# Photoinduced Reductive Elimination from Sulfur Enables a Unique Synthetic Valorization of Sulfonamides

**DOI:** 10.1021/jacs.6c07120

**Published:** 2026-08-11

**Authors:** Jaxon L. Barney, Bhargav Patel, Santino M. Servagno, Alexander J. Reif, Somnath Das, Wyatt A. Gibbs, Brian J. Levandowski, Mrinmoy Das, Elvira R. Sayfutyarova, Bryan Kudisch, Michael D. VanHeyst, Eric D. Nacsa

**Affiliations:** † Department of Chemistry, 8082 The Pennsylvania State University, University Park, Pennsylvania 16802, United States; ‡ Discovery Chemistry, Molecular Modalities Discovery, GSK, Collegeville, Pennsylvania 19426, United States; § Department of Chemistry & Biochemistry, 7823 Florida State University, Tallahassee, Florida 32306, United States; ∥ Molecular Design, GSK, Collegeville, Pennsylvania 19426, United States

## Abstract

A photochemical desulfonylation of sulfonamides has been discovered. Sulfonamides are abundant in medicinal-chemistry libraries. Strategies for their synthetic valorization remain relatively sparse, however, and their known reactions overwhelmingly retain the sulfonyl group, limiting the chemical space accessible from sulfonamides. Using this new method, an extensive array of aromatic sulfonamides bearing a selection of *N*-heteroaryl substituents was converted into the corresponding *N*-heteroaryl anilines, which are also medicinally important substructures. This reaction involves a unique photoinduced C–N reductive elimination from sulfur, which is proposed mainly to involve an excited-state, desulfonylative S-to-N aryl migration. An analogous polar aryl migration from a higher-energy, photogenerated sulfonamide tautomer was also identified, which likely serves as a minor pathway.

## Graphical abstract

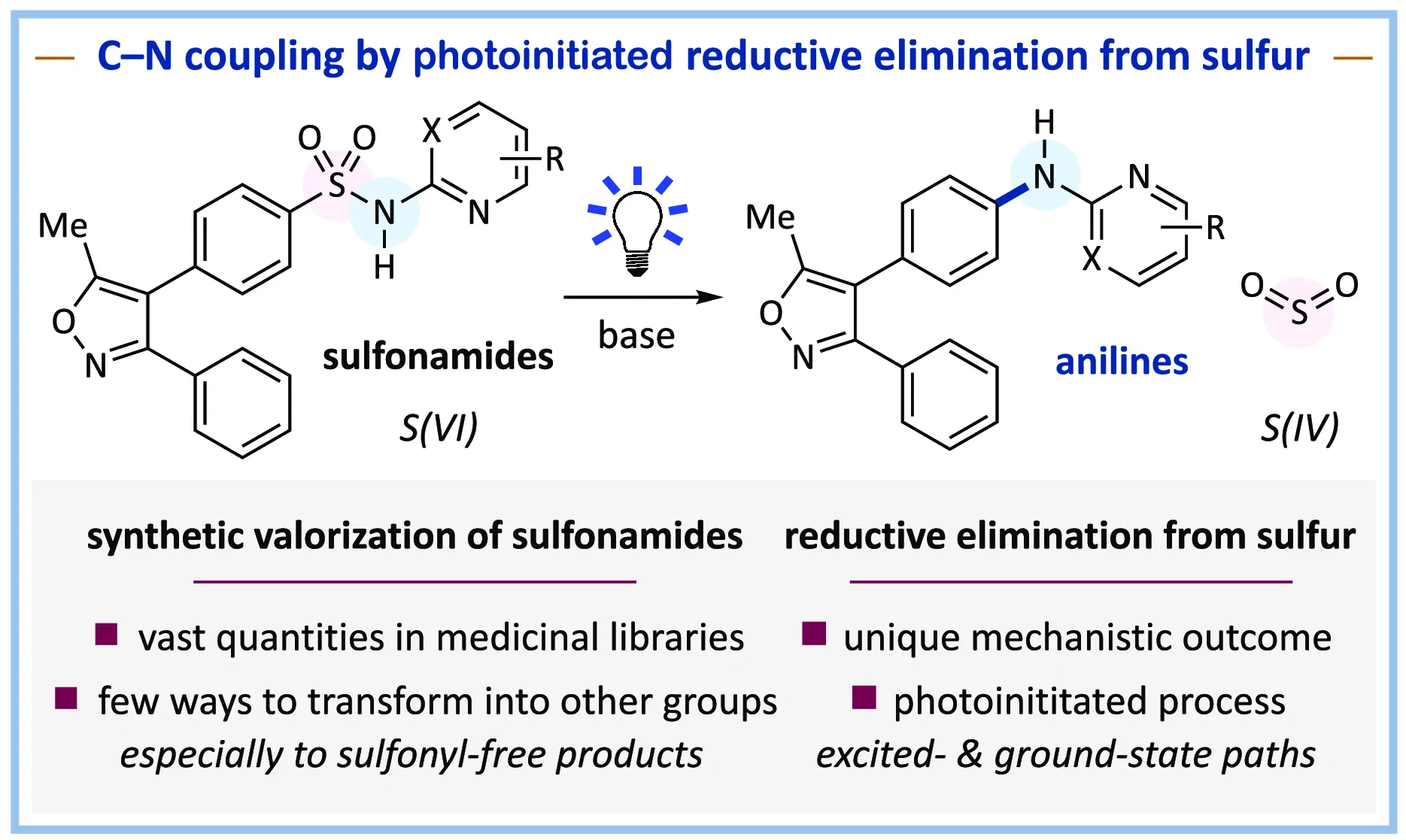

## Introduction and Background

Sulfonamides are ubiquitous in the pharmaceutical industry owing to their privileged role in medicines (Figure 1a). After C, H, O, and N, sulfur is the next-most-common atom in approved drugs,^1^ and sulfonamides represent the most-common form of sulfur in this space.^2^ Aided by recent improvements in access,^3^ companies have therefore amassed significant quantities of sulfonamides in their compound libraries, with Merck reporting in 2019 that they have “similar numbers of sulfonamides and arylboronic acids.”^4^ In contrast to the latter synthetic linchpins, however, sulfonamides are often regarded as synthetic end points rather than starting points. As a result, unless there is a demand to synthesize more of the target compounds containing sulfonamides – which is rarely the case in discovery research – these sulfonamides have limited ongoing utility.

**Figure 1:**
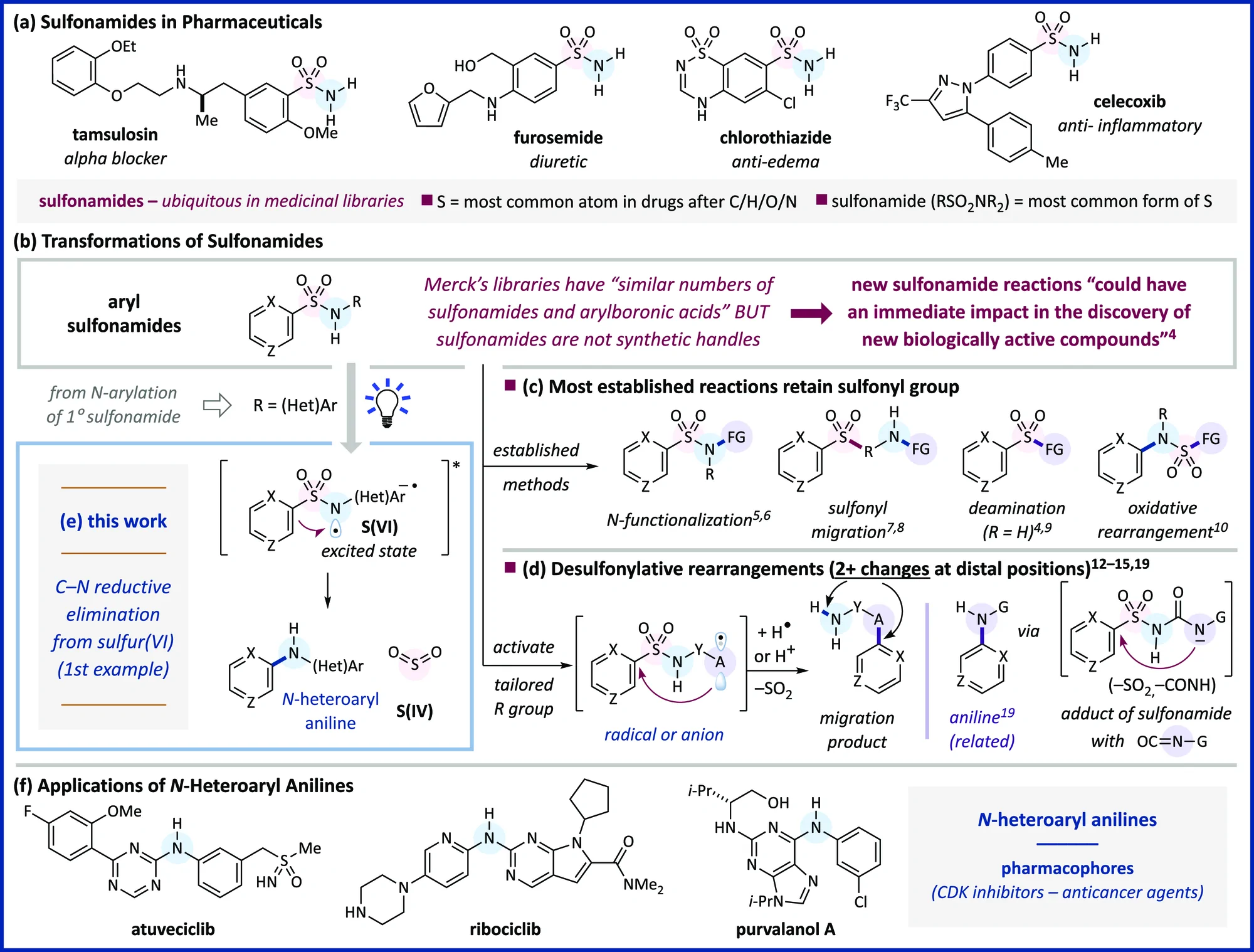
Sulfonamides in pharmaceutical compounds and libraries, their reactions, and medicines containing *N*-heteroaryl anilines.

Discovery chemists have therefore noted that new transformations of sulfonamides “could have an immediate impact in the discovery of new biologically active compounds”^4^ (Figure 1b). Sulfonamides participate in a handful of reaction classes (Figure 1c), with *N*-alkylation^5^ and *N*-arylation^6^ being well-developed. Additionally, several polar^7^ or radical^8^ sulfonyl migrations have been described, and more recently, deaminative reactions involving two-electron pathways^4,9^ or generating sulfonyl radicals have emerged,^10^ as have a selection of oxidative Hofmann-type rearrangements.^11^ These reactions all retain the sulfonyl subunit, however, restricting the chemical space they can access.

Polar^12^ and open-shell^13^ desulfonylative reactions of sulfonamides that simultaneously alter sites several atoms apart have also been studied^14,15^ (Figure 1d). These reactions have a range of synthetic uses, although they necessarily involve changes to the substrate(s) several atoms apart. Last year, for example, we (J.L.B. and E.D.N.) reported a radical-mediated amino–(hetero)­arylation of olefins that bypassed the need for directing or activating groups by using sulfonamides as sources of both (hetero)­aryl and amine groups.^13^ ^a^ A key *N*-heteroaryl substituent was developed to enable the intermolecular reaction to proceed in this system via sulfonamidyl radicals. These intermediates had previously proven unsuccessful in such settings since the sulfonamides must be *N*-protected with electron-withdrawing groups, but sulfonamidyl radicals bearing conventional *N*-acyl protecting groups decompose. This challenge also shaped pioneering work in alkene amino–arylation by Stephenson,^13^ ^b,d,e,g^ and our more recent contribution led us to the present discovery.

In this context, we now report a direct, desulfonylative C–N coupling from *N*-heteroaryl sulfonamides that complements the above-listed reactions of sulfonamides (Figure 1e). This photochemical transformation involves a novel C–N reductive elimination from sulfur^16,17^ and provides access to *N*-heteroaryl anilines, which are important motifs in medicines such as anticancer CDK inhibitors (Figure 1f).^18^ A synthetically related transformation by a different approach was also described by Manna in a 2025 preprint,^19^ wherein addition of aryl sulfonamides to isocyanates first generated *N*-(arylsulfonyl)­ureas (Figure 1d). These products then underwent a subsequent hydroxide-mediated reaction to deliver anilines^20,21^ after formal loss of SO_2_ and CONH (electron-deficient aryl groups were needed owing to the anionic Smiles rearrangement mechanism).^14^

### Reaction Discovery and Scope

During our amino–(hetero)­arylation work with sulfonamides (**1**) and sulfonamidyl radicals (**2**), we hypothesized that those reactive intermediates might undergo a desulfonylative rearrangement to the corresponding anilines (**3**, Figure 2a). Encouragingly, when we subjected (*N*-heteroaryl) aryl sulfonamide **4** to conditions we previously leveraged to generate sulfonamidyl radicals^13a^ (deprotonation with K_3_PO_4_ and oxidation with an acridinium photoredox catalyst^22^ under visible-light irradiation), *N*-heteroaryl aniline **5** was obtained in 75% yield (Figure 2b). Control experiments revealed, however, that the photocatalyst was unnecessary – 96% yield was obtained without this additive, and a different mechanism was also implied. Optimal conditions involved blue-light irradiation of the sulfonamide with K_3_PO_4_ (1 equiv) in DCE/H_2_O (1:3, 0.025 M) at ambient temperature for 48 h. Alternate inorganic bases (entries 2–7) were inferior, as were organic-soluble bases (DBU, entry 8, and Bu_4_NOH, entry 9). Replacing DCE with CH_2_Cl_2_ or adjusting the DCE/H_2_O ratio again gave lower yields (entries 10 & 11). The biphasic medium was critical, as no conversion occurred using DCE or H_2_O alone (entries 12 & 13). The reaction was usually performed under nitrogen, but it also proceeded under air (65% yield, entry 14). Heating to 70 °C in the dark did not consume any sulfonamide, pointing to a light-mediated process (entry 15).

**Figure 2:**
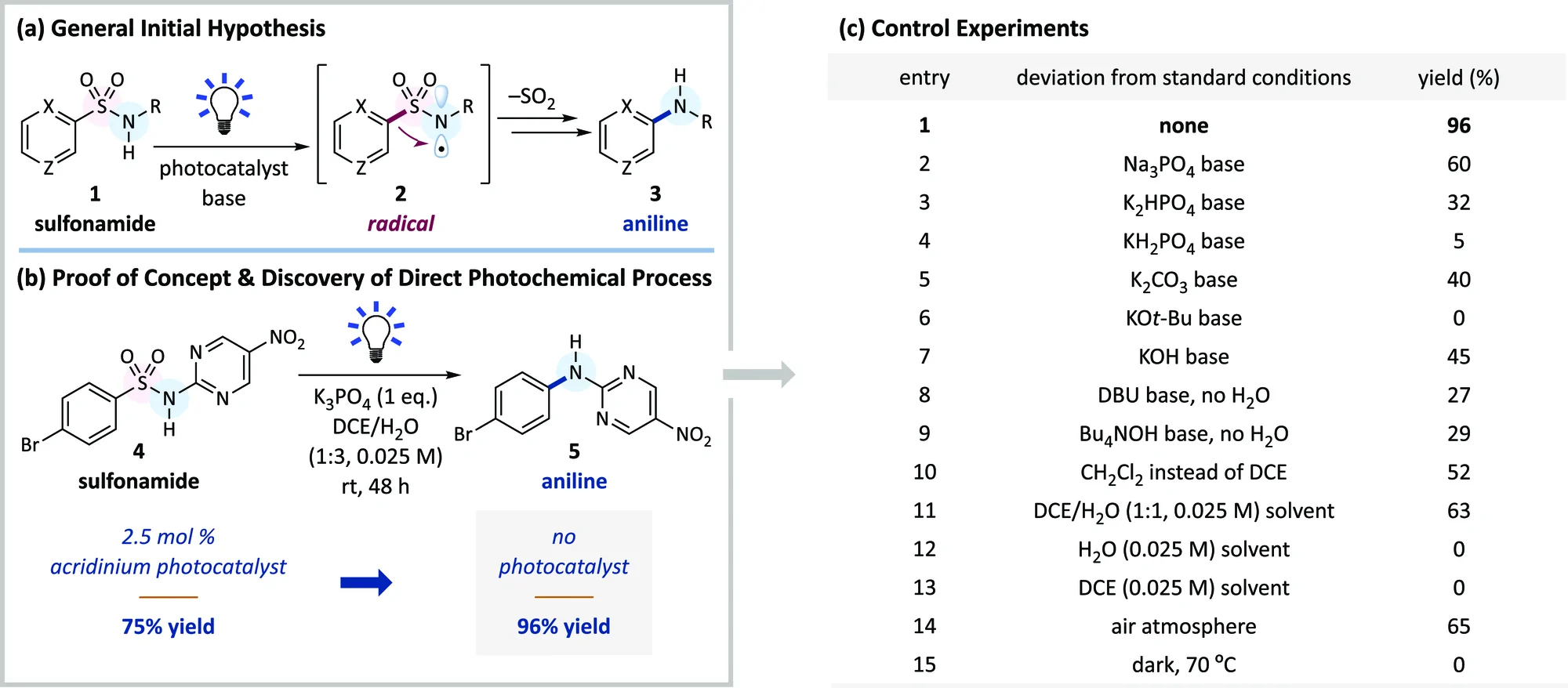
(a) Initial radical-mediated design for the desulfonylation of sulfonamides to generate anilines. (b and c) Discovery and control experiments. Conditions: sulfonamide **4** (0.1 mmol) and K_3_PO_4_ (1 equiv) were irradiated with blue LEDs in DCE/H_2_O (1:3, 0.025 M) under N_2_ near room temperature for 48 h. Deviations as noted. Yields determined by ^1^H NMR analysis.

We then evaluated the scope of this transformation (Figure 3). First, we explored heteroaryl groups (generally electron-deficient azines) as *N*-substituents in *para*-methoxyphenyl sulfonamides. Simple pyrimidine was accommodated (**6**), albeit in lower yield (37%), whereas triazines **7** & **8** and tetrazines **9** & **10** were formed in 50–99% yields. Nitropyridines **11**–**14** were accessed efficiently (65–87% yields), although CF_3_-containing analog **15** was isolated in lower 28% yield, and nitropyrimidine **16** was produced in 81% yield. Less-electron-deficient *N*-groups such as 2-pyridyl and 4-nitrophenyl were unreactive (0% yield of **17** & **18**).

**Figure 3:**
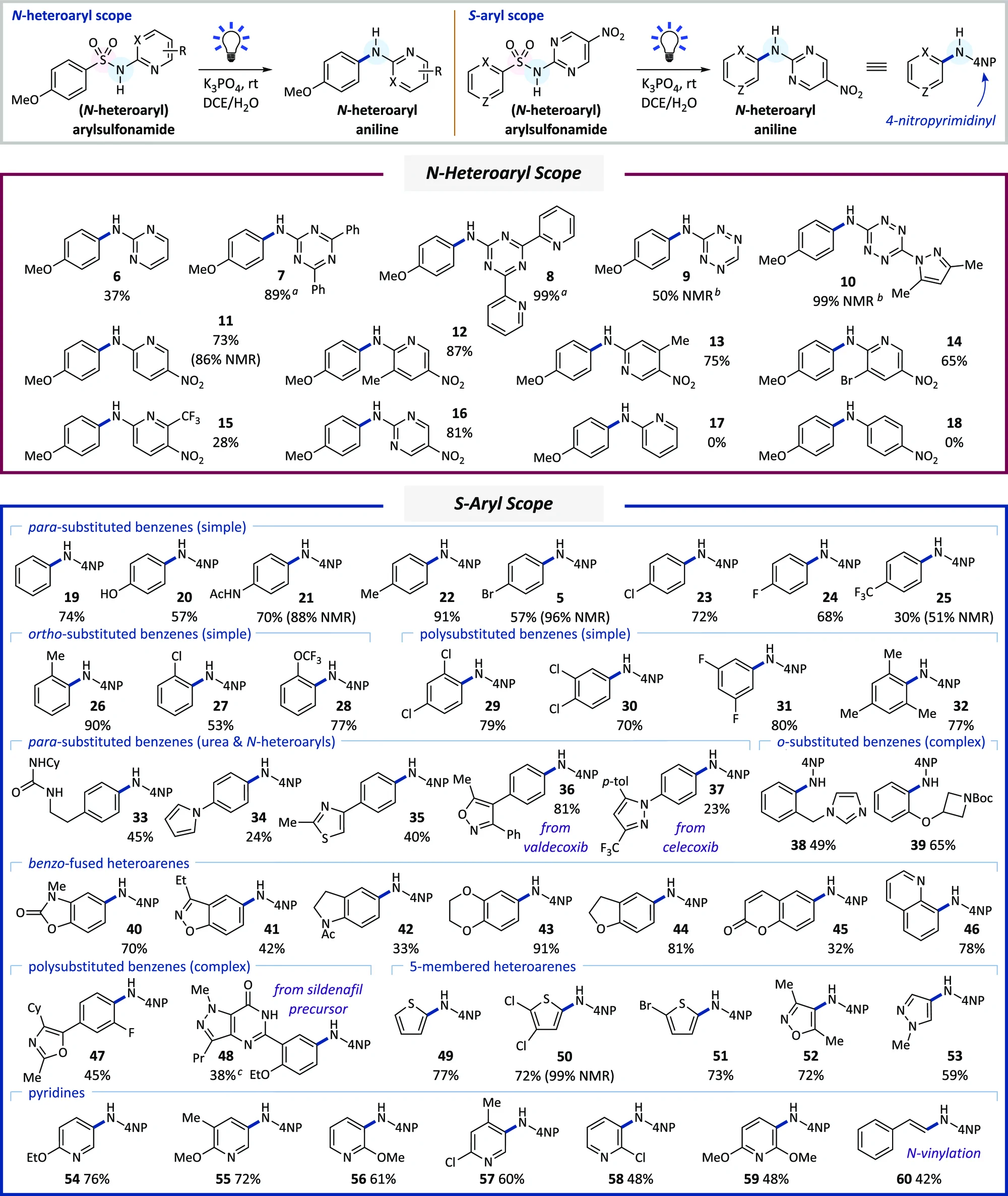
Scope for photoinitiated conversion of (*N*-heteroaryl) arylsulfonamides to *N*-heteroaryl anilines. Sulfonamide (0.1 mmol) and K_3_PO_4_ (1 equiv) were irradiated with blue LEDs in DCE/H_2_O (1:3, 0.025 M) under N_2_ near room temperature for 48 h. Isolated yields. ^*a*^ With Ir­[dFCF_3_(ppy)]_2_[4,4′-d­(CF_3_)­bpy]­PF_6_ (1 mol %), possibly as an energy-transfer catalyst. ^*b*^ Dihydrotetrazine isolated. ^*c*^ K_2_HPO_4_ for K_3_PO_4_.

Encouragingly, significant diversity was accommodated in the *S*-aryl group derived from the corresponding primary sulfonamides. Using a 4-nitro-2,6-pyrimidyl (“4NP”) *N*-substituent, phenyl and simple *para*-substituted products **5** & **19**–**24** with OH, NHAc, Me, and halogens were obtained in 57–91% yields. Increasingly electron-deficient *S*-groups reacted less efficiently, with *p*-CF_3_ product **25** isolated in 30% yield (51% NMR yield) and the *p*-NO_2_ substituent shutting down the reaction (as shown later in structure–reactivity studies). Phenyl rings with *ortho*-Me, Cl, and OCF_3_ substitution were effective (**26**–**28**, 53–90% yields), as were various difluoro and dichloro patterns (**29**–**31**, 70–80% yields) and even the hindered mesityl group (**32**, 77% yield). We then explored functionalities that are useful in medicinal chemistry but can be harder for synthetic methods to tolerate. Urea-containing product **33** was generated in 45% yield, and phenyl groups with *p*-heteroaryl substituents led to products **34**–**37** bearing pyrrole, thiazole, isoxazole, and pyrazole groups (23–81% yields, with the latter two derived from valdecoxib and celecoxib). Side chains at the *ortho*-position bearing an imidazole (**38** 49% yield) and an *N*-Boc azetidine (**39**, 65% yield) group also reacted without incident. Fused heteroaryl sulfonamides often performed well, giving products **40**–**46** containing 2-oxobenzoxazole, benzisoxazole, indoline, benzodioxane, dihydrobenzofuran, coumarin, and quinoline newly aminated at the benzo ring (32–91% yields). More-complex phenyl substitution that included an oxazole or a pyrazolopyrimidone, the latter derived from a sildenafil precursor, afforded products **47** & **48** in modest efficiency (45 and 38% yields, respectively). Five-membered heterocyclic products containing an isoxazole, a pyrazole, and thiophenes (**49**–**53**, 59–77% yields) could be obtained in good yields, and a range of pyridines (**54**–**59**, 48–76% yields) was also productive. Lastly, beyond aryl sulfonamides, a vinyl sulfonamide was converted to enamine **60** in 42% yield.

We next sought to use this transformation to access products beyond *N*-heteroaryl anilines. To this end, we were intrigued by Feng’s demonstration^23^ that the 4NP group can be cleaved from aniline **61** by hydrazine or hydroxide to reveal free aniline **62** (Figure 4a, which also involved an ester hydrolysis). To demonstrate a novel synthetic opportunity enabled by our desulfonylation, we first appended the 4NP group onto a primary sulfonamide (valdecoxib) with a S_N_Ar reaction. We then telescoped it with our photochemical desulfonylation and Feng’s 4NP cleavage (Figure 4b, a slightly higher temperature was needed herein for the last step). Therefore, starting from valdecoxib (**63**) and the requisite heteroaryl chloride (4NP–Cl, **64**), primary aniline **65** was isolated in 48% overall yield without purifying any intermediates. The individual steps proceeded in 73, 94, and 70% yields, respectively, via synthetic intermediates **66** & **35**.

**Figure 4:**
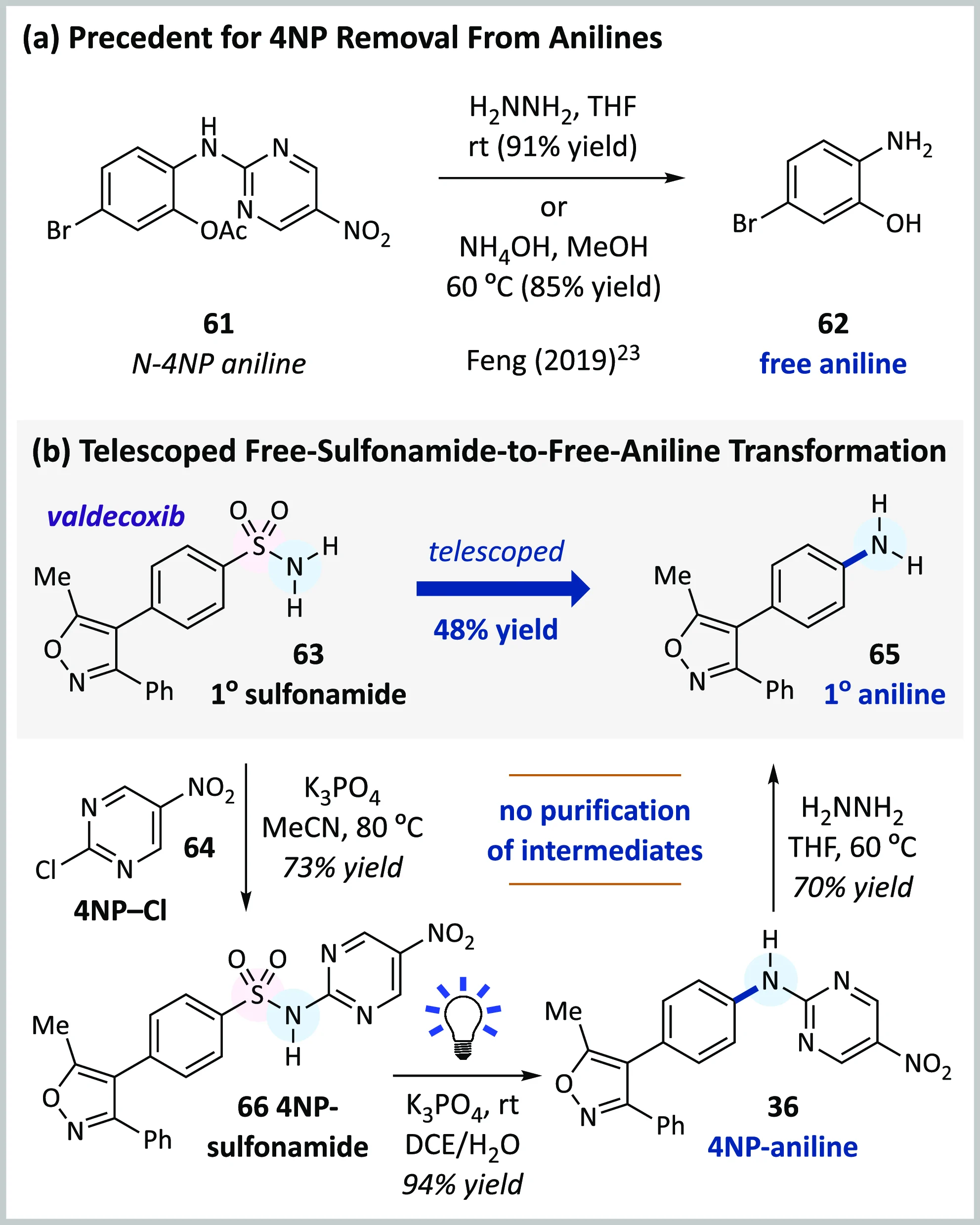
(a) Reported removal of the 4-nitropyrimidinyl (4NP) group from anilines.^23^ (b) A telescoped sequence enabling the conversion of a 1° sulfonamide to the corresponding 1° aniline.

To further explore the potential generality and utility of this desulfonylative transformation, we analyzed two libraries of primary sulfonamides (the precursors to the 4NP derivatives used to study the *S*-aryl scope in Figure 3). The first library was composed of the 228 commercial primary sulfonamides in stock (i.e., not requiring on-demand synthesis) in the eMolecules database as of June, 2026, and the second contained the 108 primary sulfonamides available internally at GSK (Figure 5, see Table S21 in the Supporting Information for the commercial structures and GSK’s compounds considered in this work).

**Figure 5:**
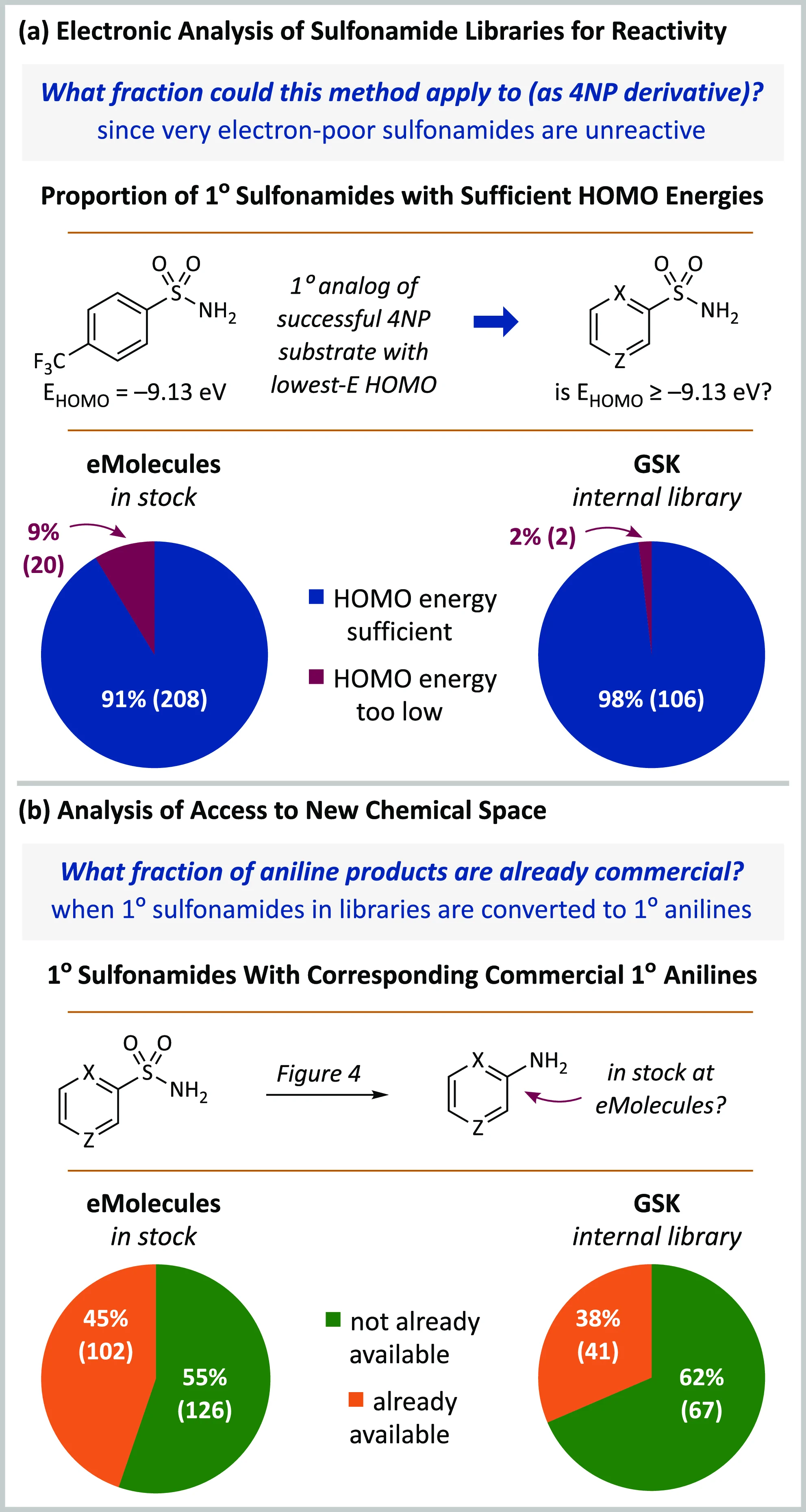
Analysis of eMolecules and GSK libraries of 1° sulfonamides by (a) HOMO energy to predict what proportion of sulfonamides would be viable in this transformation and (b) whether the corresponding 1° aniline is commercial.

Our first analysis addressed the issue that very electron-deficient *S*-aryl groups such as 4-nitrophenyl were unproductive in this reaction. We thus sought to estimate what proportion of these libraries would be expected to have enough electron density to be productive as their *N*-4NP analogs. Therefore, the HOMO of all primary sulfonamides in these libraries and the primary (des-*N*-4NP) analogs of substrates employed in Figure 3 were compared (see Table S21 in the Supporting Information for details). Among the productive *S*-aryl groups in our scope, the 4-CF_3_ analog of benzenesulfonamide that gave product **15** had the lowest-energy HOMO (−9.13 eV). We postulated that *N*-4NP analogs of primary sulfonamides with HOMO energies meeting or exceeding this threshold could be electronically capable of the desulfonylation. Encouragingly, 208 out of 228 (91%) commercial sulfonamides and 106 out of 108 (98%) among GSK’s sulfonamides met this criterion (Figure 5a).

Second, we sought to estimate the degree to which this desulfonylative process provides to new chemical space since anilines, the products of this reaction, are relatively common aromatic building blocks. To simplify this analysis that would otherwise require considering two *N*-substituents, we determined the proportion of the above-mentioned primary sulfonamide libraries, when transformed into the corresponding primary anilines (i.e., as in Figure 4), that would represent products not already commercially available (at least 9 μmol in stock at eMolecules). For both libraries, the majority of primary sulfonamides (126/228 = 55% for eMolecules and 67/108 = 62% for GSK) would generate primary anilines that are not commercial.

### Mechanistic Studies

#### Experimental Studies

To interrogate the mechanism of this new transformation, we first performed kinetic studies to assess how the electronics of the sulfonamides impacted their reactivity (Figure 6). The first plot shows that among four *para*-methoxyphenyl sulfonamides with different *N*-substituents, faster rates were associated with more electron-deficient groups. Namely, the 4-nitro-2,6-pyrimidinyl group reacted faster than the 4-nitro-2-pyridyl analog, whereas 2-pyridyl and 4-nitrophenyl groups were unproductive. A converse trend was observed for the *S*-substituent, of which five *para*-substituted phenyl groups with 4NP as the *N*-substituent were examined. In this case, reaction rates monotonically increased as the phenyl group became more electron-rich (*p*-NO_2_ < *p*-CF_3_ < *p*-H < *p*-Me < *p*-OMe). These results suggested that in the rate-limiting step, the *N*-substituent has electrophilic character, and the *S*-substituent has nucleophilic character.

**Figure 6:**
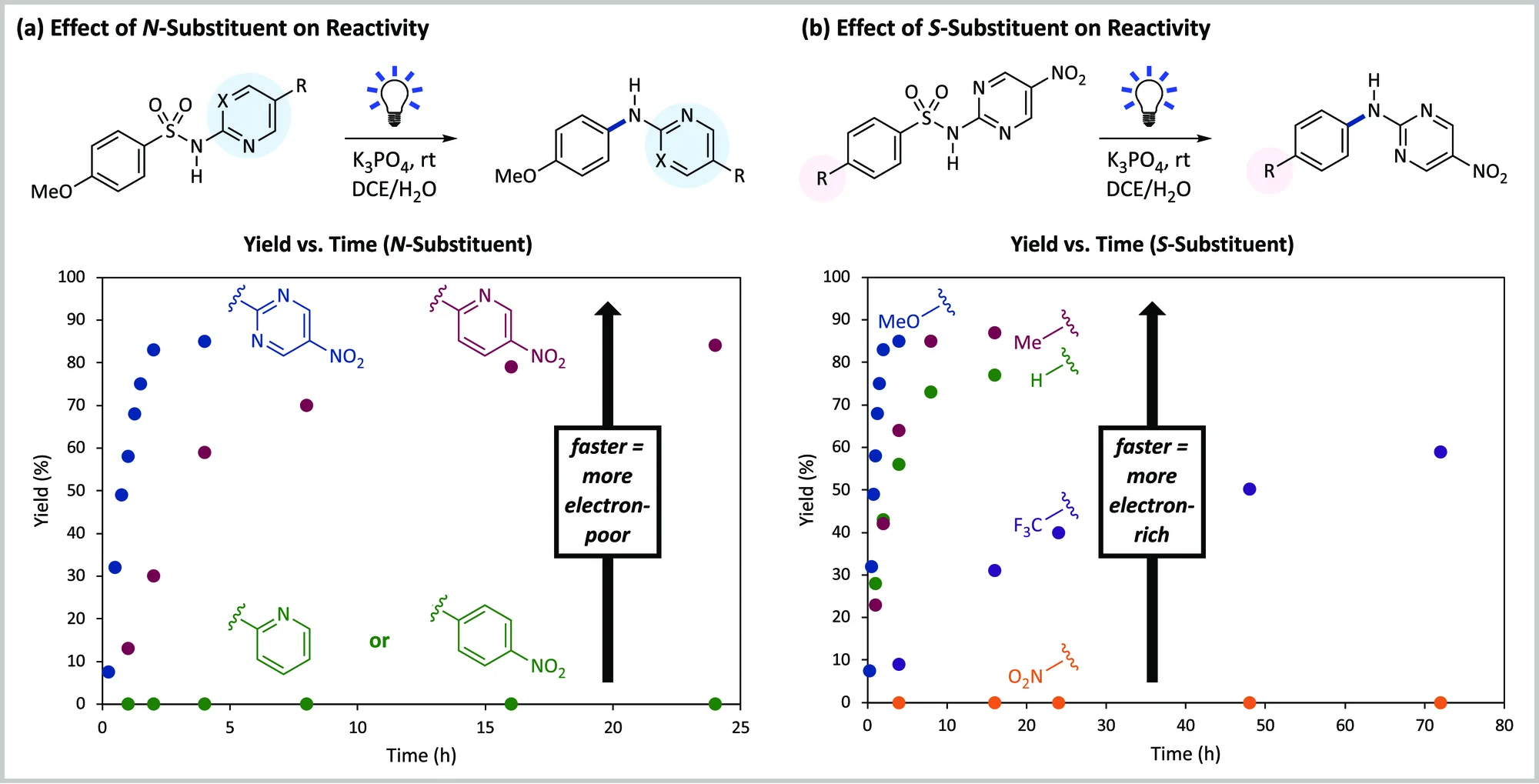
Faster reactions were observed for more electron-deficient *N*-heteroaryl groups and for more electron-rich *S*-aryl groups.

A further selection of mechanistic investigations with *p*-MeO substrate **67** is summarized in Figure 7. First, no intermediates have been observed when employing the optimal DCE/H_2_O solvent mixture, but irradiation of **67** and K_3_PO_4_ in pure H_2_O gave rise to new species (Figure 7a). We inferred that structure **68**, a phototautomer of **67** predicted to be 26.9 kcal higher in energy, was initially formed under these conditions. LC-MS and HRMS analysis gave *m*/*z* values consistent with **69**, a hydrate of **68**. Neither structure was observed in solution, however, and instead major dihydrate **70** and minor trihydrate **71** were characterized in situ by ^1^H, ^13^C, HSQC, and HMBC NMR experiments. The generation of nonhydrated **68** is likely only significant in DCE, where the concentration of H_2_O is low and the equilibrium shifts away from its hydrates. The formation of these intermediates was still usually accompanied by the formation of product **16**, which was insoluble in H_2_O and thus precipitated from the solution. Once irradiation was stopped, intermediates **70** & **71** proved relatively persistent in water, with trihydrate **71** becoming the major species after standing in the dark for 1–2 days. In contrast, the intermediates decomposed if irradiation was significantly extended after consumption of **67**.

**Figure 7:**
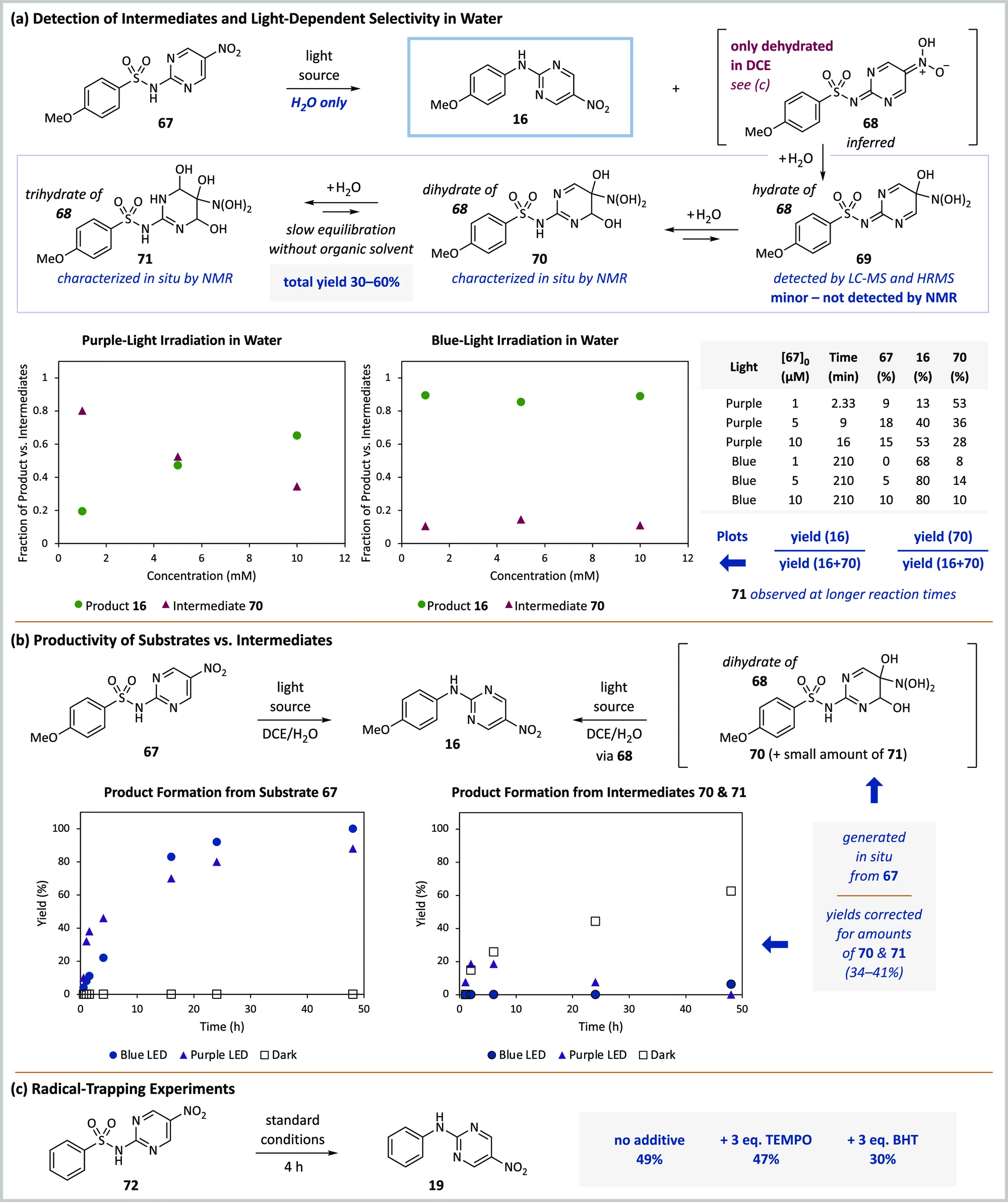
Mechanistic experiments. (a) Radical-trapping experiments. (b) Light and concentration dependence for the conversion of sulfonamide **67** to product **16** vs intermediate **70** (**71** did not form in these cases). (c) Comparison of substrate **67** vs intermediates **70** & **71** to generate product **16** using different light sources.

The selectivity between the formation of product **16** and intermediate **70** in H_2_O solvent could be controlled as a function of the light source and starting concentration of **67** (Figure 7a, **71** was not detected in these cases since reaction times were short). Using irradiation times that consumed ≥ 80% of substrate **67**, purple-light irradiation, which supplies a much-higher flux of usable photons than blue light (see Figures S16–S17 in the Supporting Information for the UV–vis absorption spectra of **67**), favored intermediate generation at high dilution (1 mM, 13% yield of **16** vs 53% yield of **70**, 20:80 ratio). An unselective outcome (47:53) was observed at 5 mM, and the product was favored (65:35) at 10 mM. Under blue-light irradiation, which should generate a lower concentration of excited states, the product was favored in 89:11, 85:15, and 89:11 ratios at the above-listed initial concentrations of **67**, respectively.

Further experiments were performed to assess the productivity of intermediates **70** & **71** under optimized solvent conditions (1:3 DCE/H_2_O, 0.025 M in sulfonamide, Figure 7b). The yield of **16** was monitored over time as a function of the light source (purple LED, blue LED, or dark) starting from either sulfonamide **67** or a mixture of intermediates **70** & **71** (generated first from **67** in pure H_2_O under purple-light irradiation before adding DCE, then degassing the mixture, and lastly applying the purple LED, blue LED, or no light source). Purple-light irradiation of substrate **67** gave a higher initial rate than blue-light irradiation (46% yield vs 22% yield after 4 h, respectively), but the purple-light yield was surpassed overnight and gave a lower final yield than with blue light (88% yield vs 99% yield after 48 h, respectively). No reaction occurred in the dark. The reactivity of intermediates **70** & **71** was distinctly different. Purple- or blue-light irradiation of these compounds was inefficient (<20% yields at all times), even after correcting for the modest conversions to **70** & **71** (34–41% in these batches), and the purple-light experiment eventually destroyed the product that had initially formed. In the dark, however, product **16** formed in 63% yield after 48 h. This result was surprising, but it may only reveal a minor pathway for product generation since it was slower than the light-mediated conversions of **67**. Moreover, the yield of **16** based on **67** in this two-step experiment was only 21% since the combined yield of **70** & **71** was 34%. Analogous intermediates were also detected by in situ NMR when the MeO group was replaced with Me or H, and they also formed the corresponding aniline products after addition of DCE and stirring in the dark, but the efficiencies were lower in these cases (see Figures S11–S12 and S14–S15 in the Supporting Information).

Lastly, in radical-trapping experiments, the addition of TEMPO (3 equiv) had an insignificant effect on the yield (47% yield of product **19** from **72** with TEMPO compared to 49% yield with no additive in 4 h, Figure 7c). Although BHT (3 equiv) modestly suppressed the yield to 30%, these results disfavored the formation of free-radical intermediates.

#### Computational Studies

Density functional theory (DFT) calculations were also performed for mechanistic studies of possible reaction pathways. (Figure 8). Using the same series of sulfonamides studied kinetically in Figure 6 (aside from the unproductive *N*-(2-pyridyl) congener), the structures, electronic energies, and free energies (when possible) were computed for the ground state (**S**_0_^–^), singlet excited state (^1^ **ES** ^–^), and triplet excited state (^3^ **ES** ^–^) of the sulfonamidyl anions. The corresponding neutral triplet excited states (^3^ **ESH**), singlet phototautomers (^1^ **SH**), and the singlet and triplet transition states for the desulfonylative [1,2]-shifts in both anionic and protonated (at the O atom of the nitro group) forms (^1^ **TS** ^–^, ^3^ **TS** ^–^, ^1^ **TSH**, and ^3^ **TSH**, respectively) were also investigated.

**Figure 8:**
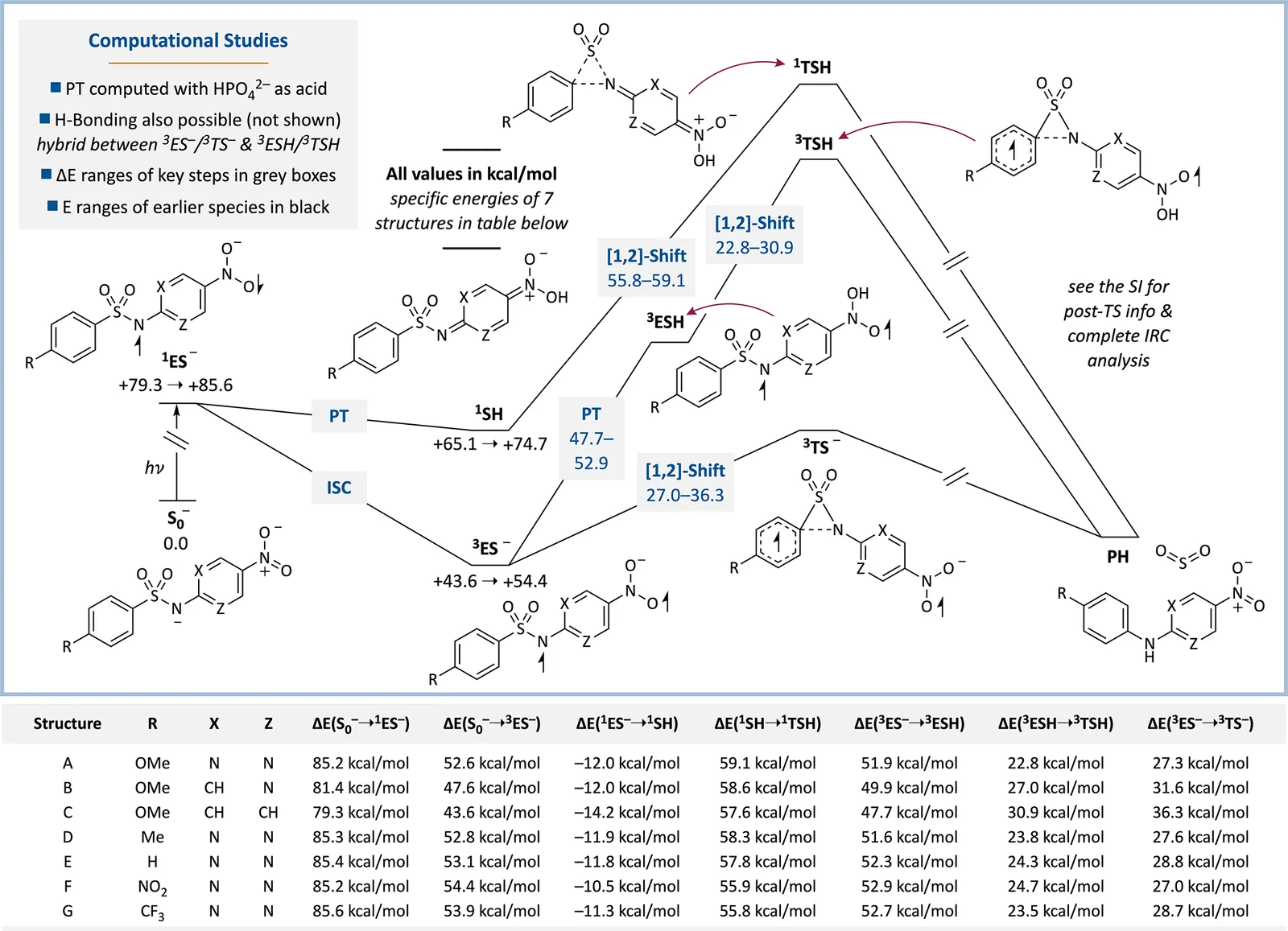
DFT analysis of possible reaction mechanisms for a series of model sulfonamides studied kinetically in Figure 6.

Calculations predicted that photoexcitation of the conjugate base (**S**_0_^–^) of the sulfonamide (we measured p*K*_a_ = 14.7 in MeCN for the *N*-4NP derivative of an aryl sulfonamide, similar to the conjugate acid of a pyridine and thus readily deprotonated by K_3_PO_4_) moves electron density from the sulfonamide nitrogen atom to the nitro group in ^1^ **ES** ^–^ & ^3^ **ES** ^–^ (see the Supporting Information). This photoinduced electron transfer can promote the formation of ^1^ **SH** (proposed intermediate **68** when R = MeO & X = Z = N) via nitro-group *O*-protonation and relaxation to the ground state, which was predicted to be 10.5–14.2 kcal/mol downhill for protonation of ^1^ **ES** ^–^ by HPO_4_^2–^. This type of phototautomerization has been characterized in many organic molecules containing both acidic and basic functional groups.^24^ High barriers (55.8–59.1 kcal/mol) were predicted for the polar, ground-state [1,2]-shift from ^1^ **SH** to ^1^ **TSH**, however. Moreover, their trends did not align with observed kinetic trends, as the lowest barriers were predicted for the least-reactive sulfonamides, while the highest barriers were predicted for the most-reactive analogs (compare to Figure 6).

Alternately from ^1^ **ES** ^–^, protonation and intersystem crossing (ISC) before relaxation could lead to ^3^ **ESH** and then ^3^ **TSH**. The barriers for the excited-state [1,2]-shifts from ^3^ **ESH** to ^3^ **TSH** were predicted to be more accessible (22.8–27.0 kcal/mol for productive compounds) than for the corresponding ground-state shifts. In addition, aside from CF_3_-containing structure G, the predicted energy barriers for this series aligned with the observed kinetic trends in Figure 6. The viability of this pathway was complicated by the prediction, however, that complete proton transfer to ^3^ **ES** ^–^ from HPO_4_^2–^, the strongest acid that should be present in solution, to be >47 kcal/mol uphill for all structures studied. Therefore, we propose that a hybrid scenario between the limiting anionic and neutral manifolds that would involve H-bonding to activate the nitro group may be operating instead.

Among the transition states for the [1,2]-shifts following directly from anionic excited states, we focused on the triplet manifold because the electronic energy barriers from ^1^ **ES** ^–^ to ^1^ **TS** ^–^ were negative and were most favored for the least-reactive sulfonamides (see Figure 6 and the Supporting Information). In contrast, the anionic triplet transition states (^3^ **TS** ^–^) were predicted to lie 27.0–31.6 kcal/mol higher than ^3^ **ES** ^–^. Among structures A–C, the calculated barriers were higher when the *N*-substituent was less electron-withdrawing, in line with Figure 6, but the observed trend of more electron-donating *S*-substituents reacting faster did not align with the predicted barriers among structures A & D–G.

## Discussion

These combined results indicated that an excited-state mechanism and a ground-state, polar mechanism could both account for the desulfonylative C–N bond formation. A condensed version of our working hypothesis is shown in Figure 9. The ground-state, polar path (top) is proposed to play a minor role under optimized conditions, as discussed below. It would involve *O*-protonation and relaxation from ^1^ **ES** ^–^ (or from ^3^ **ES** ^–^ with concomitant ISC) to ^1^ **SH**, followed by a desulfonylative polar [1,2]-shift proceeding via ^1^ **TSH** (see Figure 8). The excited-state path (bottom) would then play the major role under optimized conditions. Following ISC from ^1^ **ES** ^–^ to ^3^ **ES** ^–^, H-bond activation of the radical anion, mainly located on the nitro group, by HPO_4_^2–^ (involving complex [^3^ **ES**·HPO_4_]^3–^) would facilitate the radical-type [1,2]-shift of the *S*-aryl group. This pathway represents a hybrid of the fully anionic and neutral excited-state paths in Figure 8, although we cannot rule out those scenarios, nor analogous singlet excited-state structures.

**Figure 9:**
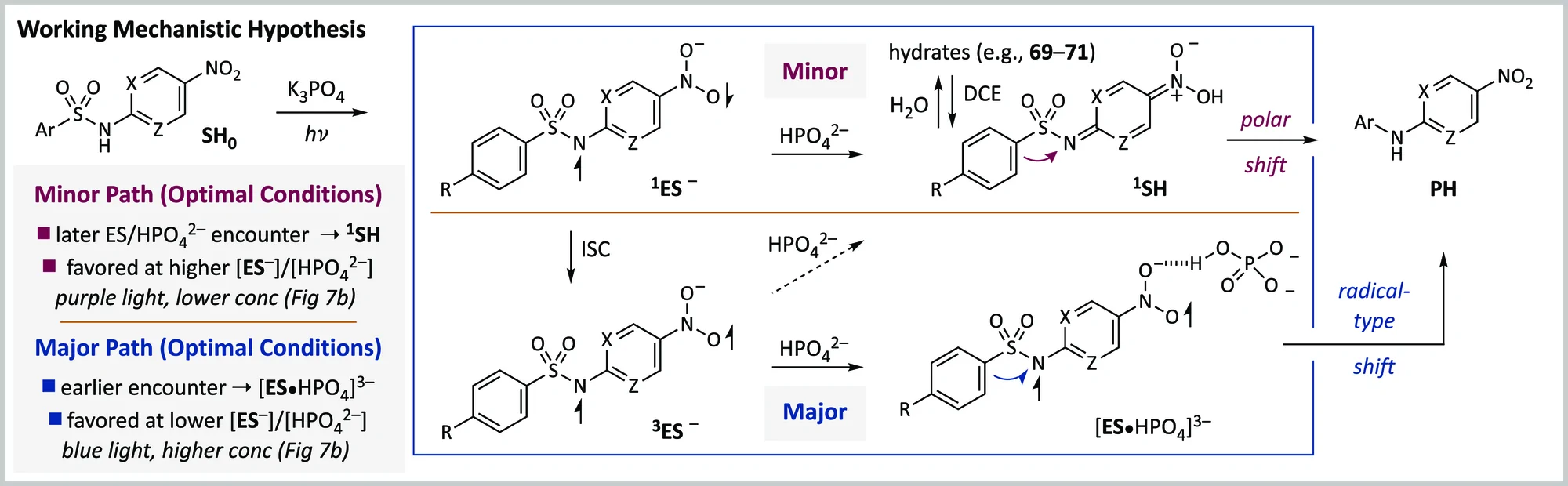
Mechanistic hypothesis based on results outlined in Figures 6–8.

We postulate that the polar, ground-state reaction occurs when intermediate ^1^ **SH** can exist in the depicted dehydrated form, wherein the reactive N atom is conjugated to the electron-withdrawing nitronic acid. This structure should only be favored in DCE, where the concentration of H_2_O is low, whereas hydrates such as **70** & **71** that lack this conjugation are favored in H_2_O solvent and thus persist when generated in DCE-free photoreactions (see Figure 7b,c). Multiple observations suggest that the ground-state [1,2]-shift from ^1^ **SH** is not significant under optimized reaction conditions, however. First, although model substrate **67** can be converted to intermediates **70** & **71** that imply the generation of **68** (i.e., ^1^ **SH**) in good efficiencies (66% yield under purple-light irradiation at 1 mM in H_2_O), this yield did not exceed 10% under blue-light irradiation (the optimal light source for product formation) at any concentration, even without DCE cosolvent (Figure 7a). Second, we have not observed these intermediates when employing optimized solvent mixtures (DCE/H_2_O). Third, although intermediates **70** & **71** were converted to product **16** in modest efficiency in the dark, this process was (a) slower than the light-mediated conversion of substrate **67** to **16** and (b) largely suppressed under blue- or purple-light irradiation (Figure 7b). The latter phenomenon, combined with the greater propensity of purple light to generate intermediates **70** & **71**, could also explain why purple-light irradiation gave a lower yield than blue light under optimized conditions even though the purple-light reaction had a greater initial rate (Figure 7b). Fourth, conversions of these intermediates to the corresponding products were much less efficient when the MeO group in substrate **67** was switched to Me or H (see the Supporting Information). Therefore, although, (*N*-(1-hydroxyalkyl)-*N*,*N*-bis­(hydroxy))­amines such as intermediates **69** have been identified as intermediates in the Nef reaction^25^ and polar [1,2]-shifts of the *S*-aryl group in sulfonamides bearing electron-withdrawing *N*-substituents have been documented in Hofmann-type rearrangements retain the sulfonyl group on nitrogen,^11,26^ our results suggest that this mechanism plays at most a minor role.

We thus favor the proposed excited-state pathway in Figure 9 as the primary productive mechanism under optimized conditions. Among the permutations explored computationally for *S*-aryl [1,2]-shifts involving singlet excited states, triplet excited states, or ground states under anionic or neutral manifolds, the ^3^ **ESH** to ^3^ **TSH** step was the only one for which the predicted energy barriers (Figure 8) mostly aligned with the observed kinetic trends (Figure 6). Since complete protonation of ^3^ **ES** ^–^ by HPO_4_^2–^ was predicted to be highly endergonic, however, H-bonded complex [^3^ **ES**·HPO_4_]^3–^ was proposed as a possible precursor to the desulfonylative migration. Compared to the pathway involving only ^3^ **ES** ^–^ and ^3^ **TS** ^–^, H-bond activation of the nitro-group-based radical anion is expected to lower the barrier to the [1,2]-shift for all structures (A–G in Figure 8), and particularly for those with more-electron-rich *S*-aryl groups. It is also likely, however, that the efficiencies of photophysical processes such of ISCs and unproductive decays at various points throughout this landscape also contribute to the experimental kinetics. Transient absorption experiments to elucidate these details and better explore the mechanism are underway.

Excited-state [1,2]-shifts were predicted to first to involve C–N bond formation, followed by C–S cleavage, and last N–S cleavage (see the Supporting Information). Significant radical character between the migrating *S*-aryl ring and the proximal S and N atoms was also predicted, although the lack of inhibition by TEMPO (Figure 7c) is consistent with the short-lived lifetimes expected for excited states compared to free radicals.

Finally, the mechanistic framework outlined in Figure 9 can also help explain the selectivity trends between the formation of product **16** vs intermediate **70** from sulfonamide **67** in H_2_O (Figure 7a). First, we ruled out the ground-state pathway for the formation of **16** in pure H_2_O since the necessary intermediate, **68**, is hydrated in this solvent and irradiation of **70** & **71** was unproductive (see above). Next, as proposed earlier, H-bond activation of anionic excited-state intermediates by HPO_4_^2–^ may be important for inducing the desulfonylative [1,2]-shift of the *S*-aryl group. Since these excited-state anions should be short-lived and the formation of complexes ([**ES**·HPO_4_]^3–^) would be bimolecular, the highest efficiencies for complex formation from any given **ES** ^–^ are expected at low ratios of [**ES** ^–^] to [HPO_4_^2–^] (we favor the triplet manifold but would expect similar trends if the singlet manifold was operating). This scenario occurs under blue-light irradiation, which provides a lower flux of usable photons than purple light to generate **ES** ^–^, and at higher initial concentrations of **67** and K_3_PO_4_ (to generate HPO_4_^2–^), as observed in Figure 7a. Conversely, we propose that less-efficient formation of [**ES**·HPO_4_]^3–^ from **ES** ^–^ leads more often to relaxation before H-bonding can assist in the productive [1,2]-shift. In these cases, simple *O*-protonation of the nitro group can occur, generating ^1^ **SH** (and thus **70** & **71** in Figure 7a). The highest efficiencies for this outcome are expected at higher ratios of [**ES** ^–^] (i.e., under purple-light irradiation) compared to [HPO_4_^2–^] (at lower initial concentrations of **67** and K_3_PO_4_), also as observed in Figure 7a.

Altogether, we believe that the experimental and computational studies are most consistent with the major and minor pathways summarized in Figure 9. Future work will focus on photophysical experiments to refine our understanding of excited-state mechanisms and whether alternate activation modes can induce similar group migrations.

## Conclusion

A novel photochemical transformation of sulfonamides has been reported. Despite the prevalence of sulfonamides in medicinal chemistry libraries, there remain limited conceptual strategies for their synthetic valorization, making such developments particularly significant. Even fewer methods allow for removal of the sulfonyl group. This approach converts a diverse range of aromatic sulfonamides, bearing various *N*-heteroaryl substituents, into the corresponding *N*-heteroaryl anilines. The process proceeds by a distinctive photoinduced C–N reductive elimination at sulfur, which is proposed mainly to involve an excited-state mechanism. A polar, ground-state mechanism from a higher-energy phototautomer was also identified, which likely serves as a minor pathway.

## Supplementary Material



## Notes

Detailed experimental procedures and NMR spectra (PDF)
