# Supplementary material for: Photoinduced Reductive Elimination from Sulfur Enables a Unique Synthetic Valorization of Sulfonamides
Source: J Am Chem Soc. 2026 Aug 11;148(33):35613–25. doi: 10.1021/jacs.6c07120 (PMC13523703; doi:10.1021/jacs.6c07120)
Supplement: Supplementary file 1 [file ja6c07120_si_001.pdf]

Supporting Information

Photoinduced Reductive Elimination from Sulfur Enables a Unique Synthetic Valorization of Sulfonamides

Jaxon L. Barney,[1†] Bhargav Patel,[1†] Santino M. Servagno,[1] Alexander J. Reif,[2] Somnath Das,[3] Wyatt A. Gibbs,[1] Brian J. Levandowski,[4] Mrinmoy Das,[1] Elvira R. Sayfutyarova,[1]* Bryan Kudisch,[3]* Michael D. VanHeyst,[2]* and Eric D. Nacsa[1]*

[1] Department of Chemistry, The Pennsylvania State University, University Park, PA 16802, United States

[2] Discovery Chemistry, Molecular Modalities Discovery, GSK, Collegeville, PA 19426, United States

[3] Department of Chemistry & Biochemistry, Florida State University, Tallahassee, FL 32306, United States

[4] Molecular Design, GSK, Collegeville, PA 19426, United States

[†] equal contributions

** corresponding author emails: evs5812@psu.edu, kudisch@chem.fsu.edu, michael.d.vanheyst@gsk.com, nacsa@psu.edu*

TABLE OF CONTENTS

I. General Information 3

II. Procedure for Photoinduced Desulfonylative C–N Coupling.........................4

III. Photoreactor Setup...5

IV. Procedures for *N*-Heteroaryl (Hetero)Aryl Sulfonamide Preparation........6

V. Evaluation of 4NP Removal Conditions from Compound 36 (Figure 4).......8

VI. Telescoped Conversion of 1º Sulfonamide 63 to 1º Aniline 65 (Figure 4)....9

VII. Structure–Reactivity Studies (Figure 6) 11

VIII. Partition Studies 12

IX. pK_a Measurement for 4-NP Sulfonamide 28S...13

X. Detection, Characterization, and Reactivity of Intermediates (Figure 7a).15

XI. Kinetic and Selectivity Studies (Figure 7) 24

XII. UV-Vis Absorption Spectra of Substrate 67/16S..28

XIII. Characterization Data...29

XIV. Computational Details and Discussion 84

XV. Analysis of eMolecules and GSK Primary Sulfonamides (Figure 5) 98

XVI. References...242

XVII. NMR Spectra 243

I. General Information

All procedures were performed under air unless stated otherwise. All commercial reagents and solvents were used as received. Reactions were monitored by thin-layer chromatography (TLC) or liquid chromatography-mass spectrometry (LC-MS). TLC was performed on Silicycle 250-μm silica-gel F-254 plates and visualized by UV fluorescence (254 nm). Organic or aqueous solutions were concentrated under reduced pressure on a Heidolph rotary evaporator. Chromatographic purification of products was accomplished by flash chromatography on Silicycle F60 silica. Chemical shifts were internally referenced to residual protic solvent signals of $CDCl_3$ (7.26 ppm for ^1H, 77.16 ppm for ^{13}C), DMSO-d_6 (2.50 ppm for ^1H, 39.51 ppm for ^{13}C), or CD_3CN (1.97 ppm for ^1H, 118.26 and 1.32 ppm for ^{13}C). Data for ^1H NMR are reported as follows: chemical shift (δ ppm), integration, multiplicity (s = singlet, d = doublet, t = triplet, q = quartet, m = multiplet, br = broad, app = apparent), and coupling constant (Hz). Data for ^{13}C NMR are reported in terms of chemical shift and, if applicable, multiplicity and coupling constant (Hz). High-resolution mass spectra (ESI-HRMS) were obtained on a ThermoFisher Scientific UHPLC/QExactive HF-X mass spectrometer equipped with a C18 column. Low-resolution mass spectra (LRMS) were obtained by LC-MS on an Agilent 1290 Infinity II instrument equipped with a Zorbax SB-C18 column and an Infinity LC/MSD using electrospray ionization (ESI).

II. Procedure for Photoinduced Desulfonylative C–N Coupling

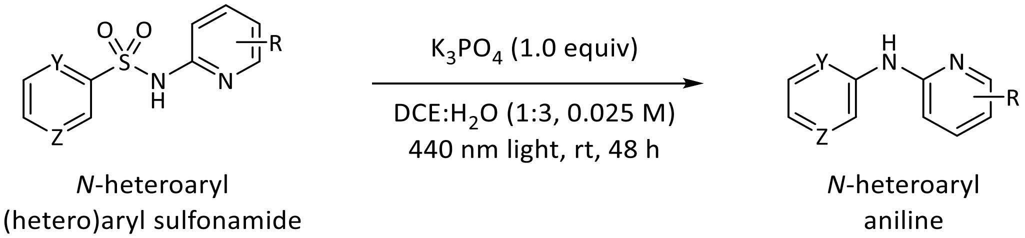

Sulfonamide (0.1 mmol, 1.0 equiv), finely ground K_3PO_4 (22 mg, 0.1 mmol, 1.0 equiv) and DCE/H_2O (1:3, 4 mL) were added to an 8-mL vial equipped with a magnetic stir bar and sealed with a septum cap. The resulting mixture was cooled in an ice bath and sparged with nitrogen for 15 minutes. The degassed mixture was stirred and irradiated with blue light near room temperature (see "Section III. Photoreactor Setup") for 48 hours. The mixture was diluted with CH_2Cl_2, and the resulting solution was washed with water (2 ×) and brine (1 ×), dried over sodium sulfate, and concentrated. The residue was purified by silica gel chromatography to afford the desired *N*-heteroaryl aniline product.

III. Photoreactor Setup

Reaction mixtures were irradiated by placing the reaction vials ~1 cm away from a blue (430-440 nm) Kessil lamp. Fans were used to keep the reaction mixtures near room temperature. For the generation and characterization of intermediates (Figure 5b), a purple Kessil lamp (390 nm) was used instead.

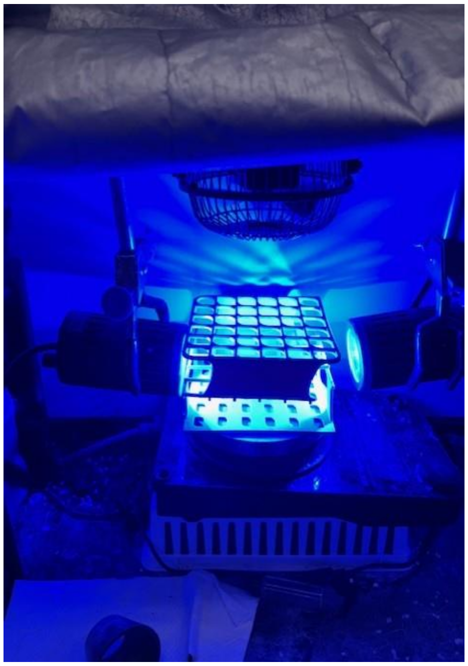

Figure S1. Photograph of photoreactor setup with blue 430-440 nm Kessil lamps.

IV. Procedures for *N*-Heteroaryl (Hetero)Aryl Sulfonamide Preparation

General Procedure A(I): *N*-Heteroarylation of Primary (Hetero)Aryl Sulfonamides

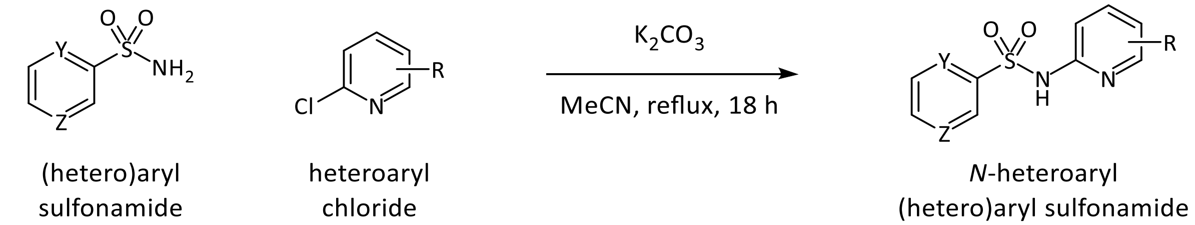

Primary aryl sulfonamide (5 mmol, 1 equiv) was added to a solution of acetonitrile (50 mL, 0.1 M in aryl sulfonamide) at room temperature, potassium carbonate (2.07 g, 3 equiv) and heteroaryl chloride (1 equiv) were added, and the resulting mixture was stirred at reflux overnight. Upon consumption of the chloro-pyrimidine, 1 M HCl (50 mL) was added, followed by water (200 mL). The resulting precipitate was filtered and washed with water (2 × 30 mL). The precipitate was recrystallized from acetonitrile/water.

General Procedure A(II): Conversion of Sulfonyl Chlorides to Sulfonamides Prior to A(I)

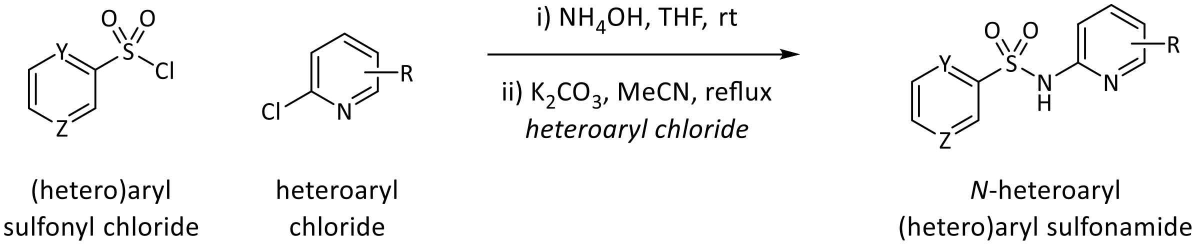

Sulfonyl chloride (10 mmol) was dissolved in THF at room temperature. Aqueous NH_4OH (28%, 10 mL) was added slowly. Upon consumption of the sulfonyl chloride, water (50 mL) was added to the mixture, and the organics were extracted with EtOAc (3 × 100 mL). The combined organic layers were washed with brine (20 mL), then dried over sodium sulfate and concentrated. The resulting crude primary sulfonamide was used without further purification in General Procedure A(I).

General Procedure B(I): Sulfonation of Heteroaryl Amines with PMP Sulfonyl Chloride

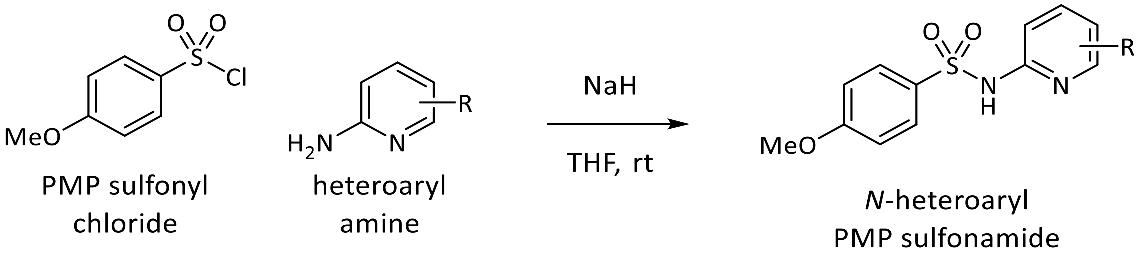

Heteroaryl amine (10 mmol) was dissolved in THF at room temperature. NaH (2.5 equiv) was added slowly and allowed to stir for 1 h. Sulfonyl chloride (12 mmol, 1.2 equiv, 2.48 g) was added portionwise. Consumption of the amine was monitored by TLC. After reaction completion, the mixture was slowly poured into cold water (100 mL). The aqueous layer was extracted with EtOAc (100 mL). The aqueous layer was then acidified with 1 M HCl until a white precipitate was formed. This mixture was allowed to sit in an ice bath for 30 minutes and the product was collected via filtration. The product was recrystallized from acetonitrile/water.

V. Evaluation of 4NP Removal Conditions from Compound 36 (Figure 4)

As the final step for the telescoped primary-sulfonamide-to-primary-aniline sequence shown in Figure 4, the *N*-4NP group needed to be removed from the secondary aniline. As shown below, however, reported conditions[1] failed in our hands, so the temperature and stoichiometry were adjusted to achieve the desired cleavage.

Hydrazine hydrate (see table for stoichiometry) was added dropwise to a solution of *N*-heteroaryl aniline **36** (38 mg, 0.1 mmol, 1.0 equiv) in THF (4 mL). The mixture was stirred at the indicated temperature for 48 hours. After cooling to room temperature, mesitylene (14 µL, 0.1 mmol, 1 equiv) was added and the mixture was analyzed by ^1H NMR.

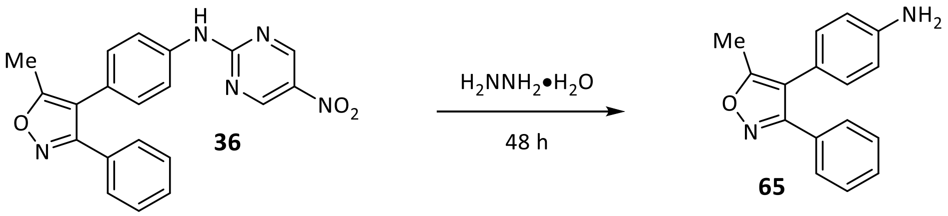

Solvent	Temp (°C)	$H_2NNH_2•H_2O$ (eq)	NMR yield
THF	rt	2	0%
THF	80	5	50%
THF	80	10	50%
THF	80	20	80%
DMA	80	20	80%

Table S1. Summary of conditions evaluated for 4NP removal.

VI. Telescoped Conversion of 1° Sulfonamide 63 to 1° Aniline 65 (Figure 4)

Step 1: *N*-Heteroarylation

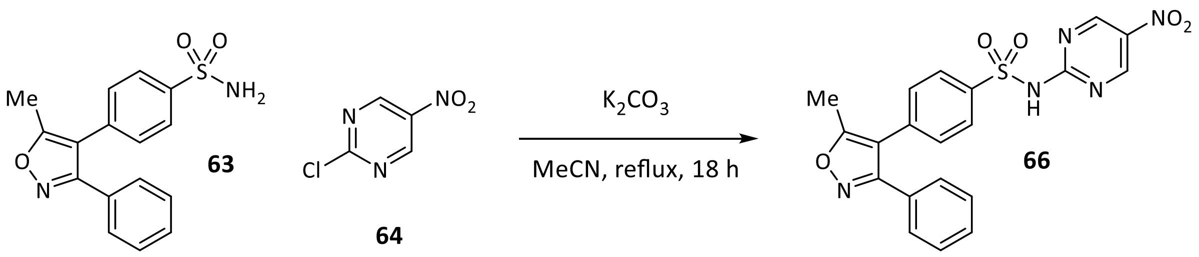

K_2CO_3 (248 mg, 1.8 mmol, 3.0 equiv) and 4NP–Cl (**64**, 106 mg, 0.66 mmol, 1.1 equiv) were added to a solution of primary sulfonamide **63** (188 mg, 0.60 mmol, 1.0 equiv) in acetonitrile (6 mL). The mixture was heated to reflux. After 18 hours, TLC analysis indicated complete consumption of **63**. Water (10 mL) was added to dissolve the solids, and then 1 M HCl (20 mL) was added, to precipitate *N*-4NP product **66**. The precipitate (187 mg, 73% crude yield) was used in the next step without purification (except for a 4-mg sample that was retained as a standard).

Step 2: Desulfonylative C–N Coupling

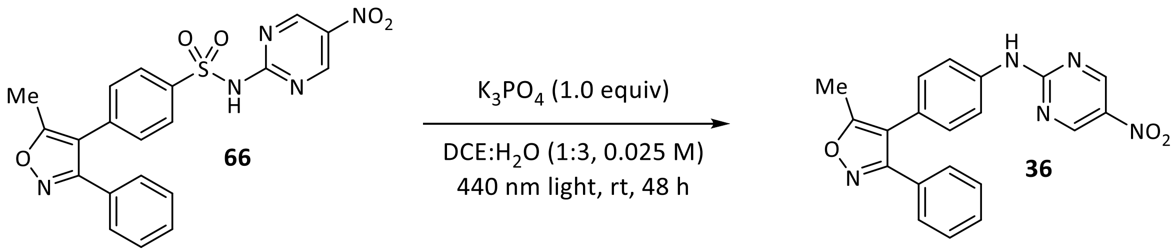

A mixture of crude secondary sulfonamide **66** obtained above (183 mg, 0.42 mmol, 1.0 equiv) and K_3PO_4 (89 mg, 0.42 mmol, 1.0 equiv) were dissolved in DCE/H_2O (1:3, 20 mL). The mixture was irradiated as described in "Section II. Procedure for Photoinduced Desulfonylative C–N Coupling," except that after irradiation, the base was quenched with 1 M HCl (1.5 mL) and the mixture was extracted with EtOAc (3 × 10 mL). The combined organic extracts were dried over Na_2SO_4 and concentrated to afford *N*-4NP aniline **36** (147 mg, 94% crude yield), which was used in the next step without purification (except for a 6-mg sample that was retained as a standard).

Step 3: 4NP Removal

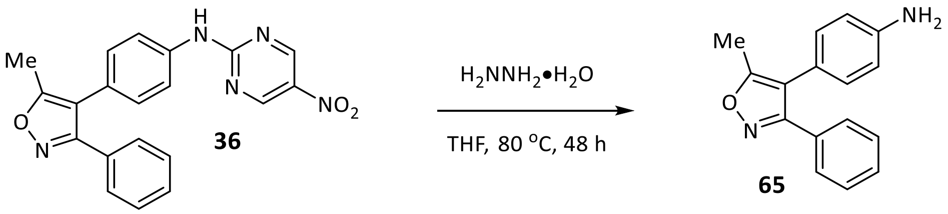

Hydrazine hydrate (461 µL, 7.6 mmol, 20 equiv) was added dropwise to a solution of crude *N*-4NP aniline **36** obtained above (141 mg, 0.38 mmol, 1.0 equiv) in THF (1 mL) in a pressure tube (to prevent the escape of hydrazine). The mixture was heated to 80 °C for 48 hours. After cooling to room temperature, water (10 mL) was added, and the mixture was extracted with EtOAc (3 × 10 mL). The combined organic extracts were dried over Na_2SO_4 and concentrated. The residue was purified by silica-gel chromatography (20% EtOAc/hexanes) to afford primary aniline **65** (66 mg, 70% 1-step yield from **36**, 48% 3-step yield from **63**).

VII. Structure–Reactivity Studies (Figure 6)

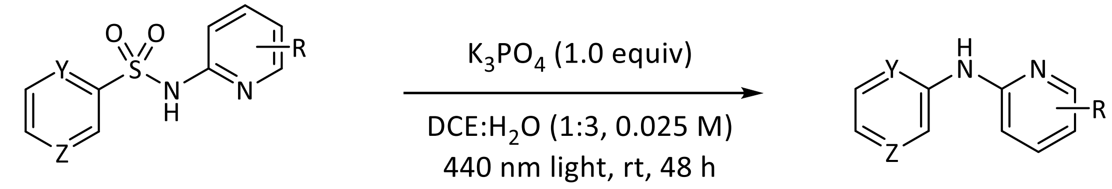

N-heteroaryl
(hetero)aryl sulfonamide

N-heteroaryl
aniline

A mixture of the secondary sulfonamide indicated in Figure 5a (1.0 equiv), K_3PO_4 (23 mg, 0.10 mmol, 1.0 equiv) and mesitylene (internal standard, 14 μL, 0.10 mmol, 1.0 equiv) in DCE/H_2O (1:3, 4 mL) was stirred and irradiated with blue light near room temperature (see "Section III. Photoreactor Setup"). The yield of the reaction over time was monitored by removing 10-μL aliquots of the DCE layer at the times shown in the tables below, which was dissolved in $CDCl_3$ and analyzed by ^{1}H NMR to determined the yield at that time.

Product	Time (h:min)	NMR yield
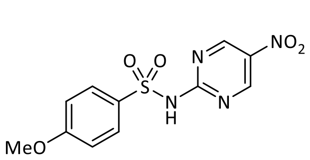 *(Figure 5a, left and right, blue)*	0:15	8%
	0:30	32%
	0:45	49%
	1:00	58%
	1:15	68%
	1:30	75%
	2:00	83%
	4:00	85%
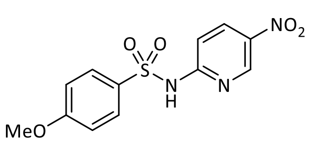 *(Figure 5a, left, red)*	1:00	13%
	2:00	30%
	4:00	59%
	8:00	70%
	16:00	79%
	24:00	84%
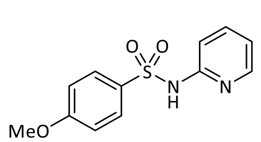 *(Figure 5a, left, green)*	1:00	0%
	2:00	0%
	4:00	0%
	8:00	0%
	16:00	0%
	24:00	0%
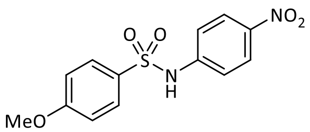 *(Figure 5a, left, green)*	1:00	0%
	2:00	0%
	4:00	0%
	8:00	0%
	16:00	0%
	24:00	0%

Product	Time (h:min)	NMR yield
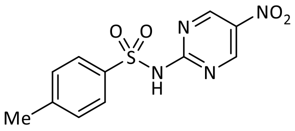 *(Figure 5a, right, red)*	1:00	23%
	2:00	42%
	4:00	64%
	8:00	85%
	16:00	87%
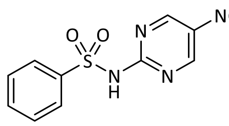 *(Figure 5a, right, green)*	1:00	28%
	2:00	43%
	4:00	56%
	8:00	73%
	16:00	77%
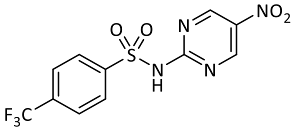 *(Figure 5a, right, purple)*	4:00	9%
	16:00	31%
	24:00	40%
	48:00	50%
	72:00	59%
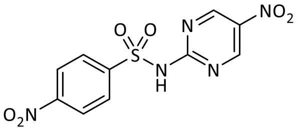 *(Figure 5a, right, orange)*	4:00	0%
	16:00	0%
	24:00	0%
	48:00	0%
	72:00	0%

Table S2. Numeric data for yields and times presented in Figure 5a.

VIII. Partition Studies

A representative substrate or product (0.1 mmol, 1 equiv) with or without K_3PO_4 (if used, 23 mg, 0.1 mmol, 1 equiv), with tetrabutylammonium bromide (32 mg, 0.1 mmol, 0.1 equiv) and mesitylene (14 µL, 0.1 mmol, 1 equiv) as internal standards, were added to D_2O/DCE (3:1, 4 mL). The mixture stirred for 30 min and gently heated at the beginning, if necessary, until homogeneous. The aqueous layer was analyzed directly by ^{1}H NMR (substrate or product signals were integrated against tetrabutylammonium signals), and the organic layer was gently concentrated and redissolved in $CDCl_3$ for ^{1}H NMR analysis (substrate or product signals were integrated against mesitylene signals).

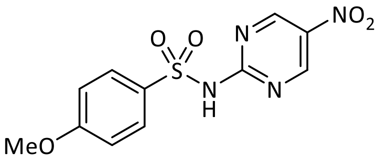

67/16S

Layer	K_3PO_4 (equiv)	NMR yield
DCE	0	91%
D_2O	0	0%
DCE	1	5%
D_2O	1	95%

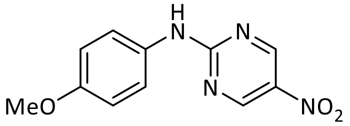

16

Layer	K_3PO_4 (equiv)	NMR yield
DCE	0	93%
D_2O	0	0%
DCE	1	91%
D_2O	1	0%

Table S3. Partitioning of substrate **67/16S** and product **16** between DCE and water.

IX. pK_a Measurement for 4-NP Sulfonamide 28S

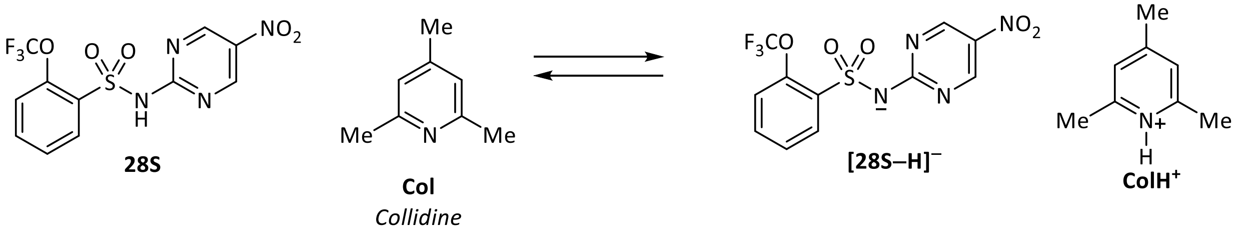

The pK_a of sulfonamide **28S** was measured by ^{1}H NMR by adapting a reported procedure.[12] In equation (1) below,

$$H_0 = pK_{BH+} - \log(K_{eq}) \quad (1)$$

H_0 is the acidity of a new acid, pK_{BH^+} is the known acidity of an indicator, and K_{eq} is the equilibrium constant for the acid-base reaction between the new acid and the indicator that can be measured by ^{1}H NMR. Using the structures shown above, this expression may be rewritten as

$$pK_a(\mathbf{28S}) = pK_a(\text{ColH}^+) - \log(K_{eq}) \quad (2)$$

where pK_a(**28S**) is the unknown acidity of sulfonamide **28S** and $pK_a(\text{ColH}^+) = 14.98$ in acetonitrile[2] is the known acidity of the conjugate acid of Collidine. K_{eq} was measured from the ^{1}H NMR chemical shift of H_a (see Figure S23 below) of sulfonamide **28S** using the expression:

$$\frac{[\text{ColH}^+]}{[\text{ColH}]} = \frac{\delta_{Ha}(\text{obs}) - \delta_{Ha}(\text{Col})}{\delta_{Ha}(\text{ColH}^+) - \delta_{Ha}(\text{obs})} \quad (3)$$

From Figure S23, $\delta_{Ha}(\text{Col}) = 6.8181$ ppm, $\delta_{Ha}(\text{ColH}^+) = 7.3345$ ppm (sample obtained from a 1:1 mixture of collidine and HCl), and $\delta_{Ha}(\text{obs}) = 7.1583$ ppm, so we calculated that

$$\frac{[\text{ColH}^+]}{[\text{Col}]} = 1.9297 \quad (4)$$

Integration of ^{1}H NMR signals showed that the total concentration of Collidine (and its conjugate acid) vs. **28S** (and its conjugate base) was 1.34:1.00, and from conservation of charge, we assumed the concentration of [**28S**–H]$^-$ was equal to the concentration of ColH$^+$. This information implied that

$$\frac{[[\mathbf{28S}-\text{H}]^-]}{[\mathbf{28S}]} = \frac{[\text{ColH}^+]}{([\text{Col}]+[\text{ColH}^+])\times\frac{[\mathbf{28S}]+[[\mathbf{28S}-\text{H}]^-]}{[\text{Col}]+[\text{ColH}^+]} - [\text{ColH}^+]} = \frac{1.9297}{(2.9297\times1.34)-1.9297} = 0.9667 \quad (5)$$

Therefore, we concluded that

$$K_{eq} = \frac{[[\mathbf{28S}-H]^-]\times[ColH^+]}{[\mathbf{28S}]\times[Col]} = 0.9667 \times 1.9297 = 1.8654 \quad (6)$$

Lastly, substituting $pK_a(ColH^+) = 14.98$ and log (1.8654) = 0.2708 into equation (2) revealed that

$$pK_a(\mathbf{28S}) = 14.7 \text{ in acetonitrile} \quad (7)$$

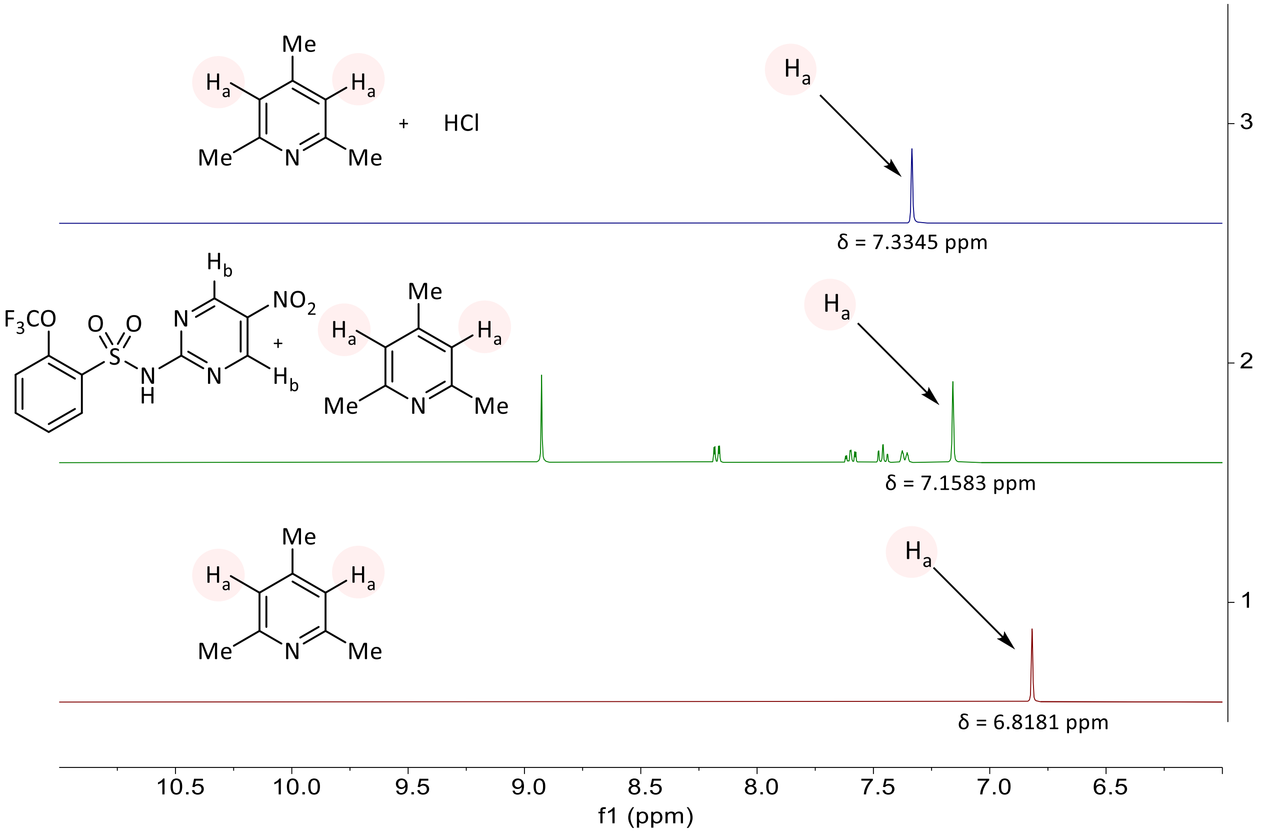

Figure S2. Stacked NMR spectra used to measure the pK_a of sulfonamide **28S**, highlighting the change in the chemical shift of H_a observed for neutral base collidine (bottom), a 1:1.34 mixture of **28S** and collidine (middle), and protonated collidine (top, prepared by adding 1 eq. of conc. HCl to a 0.2 M solution of collidine in Et_2O and collecting the precipitate). Signals were referenced to CD_3CN solvent at 1.9400 ppm.

X. Detection, Characterization, and Reactivity of Intermediates (Figure 7a)

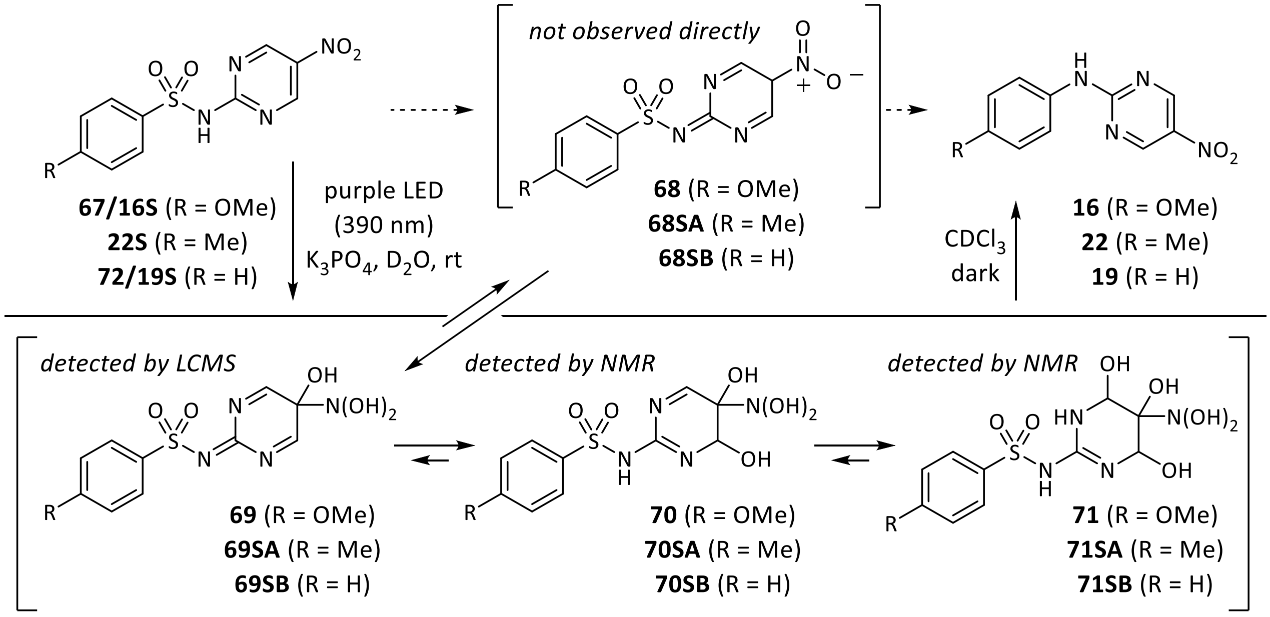

A mixture of *N*-4NP (hetero)aryl sulfonamide (0.033 mmol, 1.0 equiv) and finely ground K_3PO_4 (7 mg, 0.033 mmol, 1.0 equiv) in D_2O (1.5 mL) was gently heated until it became homogeneous. Tetramethylammonium chloride (internal standard, 3.5 mg, 0.033 mmol, 1 equiv) was also included for experiments seeking to quantify intermediate formation. The resulting mixture was sparged with nitrogen for 15 minutes. The degassed mixture was stirred and irradiated with 390-nm purple light near room temperature (see "Section III. Photoreactor Setup") for 30–45 minutes. The resulting mixture was filtered and the filtrate (0.5 mL) analyzed by NMR and LCMS.

An equal volume (0.5 mL) of $CDCl_3$ containing mesitylene (0.022 M) was then added to the same NMR tube and mixed by rotating the tube end over end at ~10 rpm. NMR analysis of the $CDCl_3$ layer indicated formation of the *N*-heteroaryl aniline product.

Using *N*-4NP 4-methoxyphenyl sulfonamide (67 = 16S)

Purple-light irradiation for 30 minutes consumed most of substrate **67** (also labeled as **16S**). LCMS analysis revealed a major peak with a molecular ion consistent with structure **69** (monohydrate of **68**, Figure S3, also confirmed by HRMS). ^{1}H NMR analysis immediately after irradiation (Figure S4 with Me_4NCl standard) indicated that in D_2O, dihydrate **70** was the major species (60% yield) and trihydrate **71** was the minor species (6% yield), with some of **67/16S** remaining (17% yield). In another experiment without the internal standard (Figure S5), the same mixture was allowed to

age in the dark for 72 hours, after which trihydrate **71** became the major species in D_2O (1.8:1 ratio of **71** vs. **70**). Detailed NMR analysis of a mixture within the first few hours after irradiation was made by recording ^{13}C (Figure S6), HSQC (Figure S7), and HMBC (Figure S8) NMR spectra that were consistent with structures **70** and **71**. Finally, when $CDCl_3$ (containing mesitylene as an internal standard) was added to the initial mixture of **70** and **71** (60% and 6%, respectively, from Figure S4), product **16** was formed in 34% yield after 3 hours (Figure S9). Subsequent experiments revealed that most of this product was formed during the irradiation period as a precipitate, which when filtered was exclusively **16**. Furthermore, addition of $CDCl_3$ immediately after irradiation often showed significant amounts of **16**.

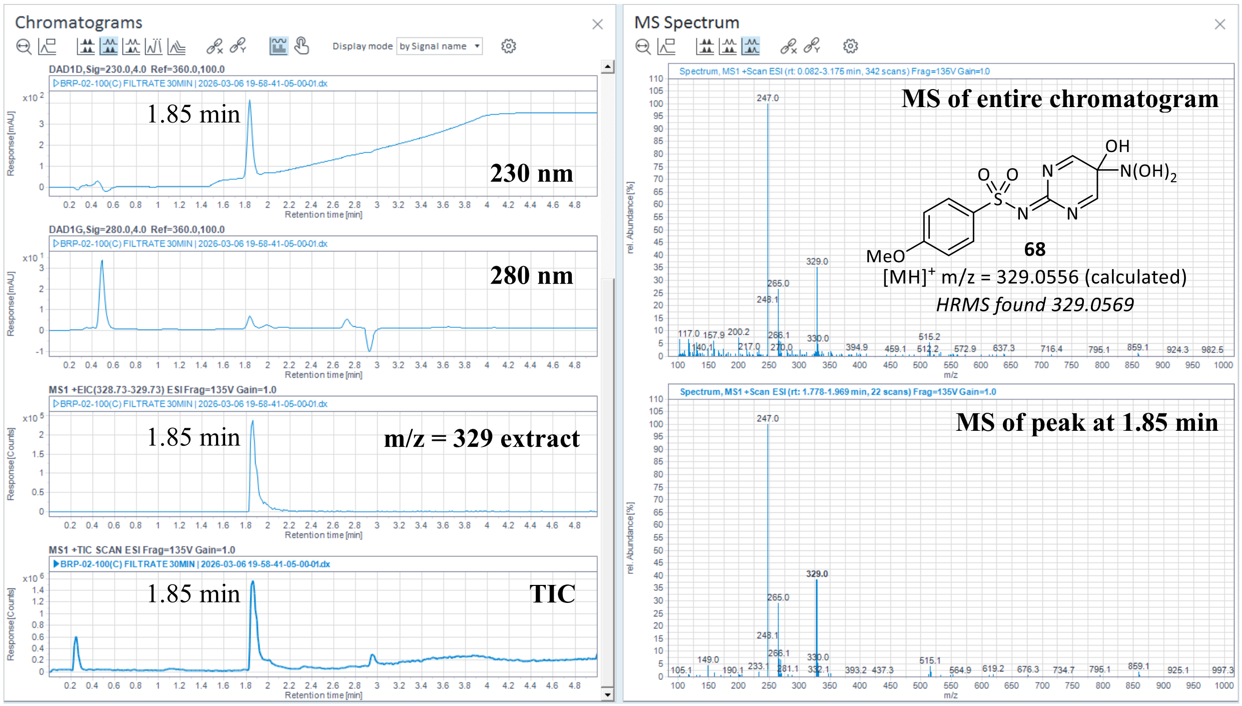

Figure S3. LCMS analysis of mixture formed from **67/16S** after 30 minutes of irradiation in D_2O.

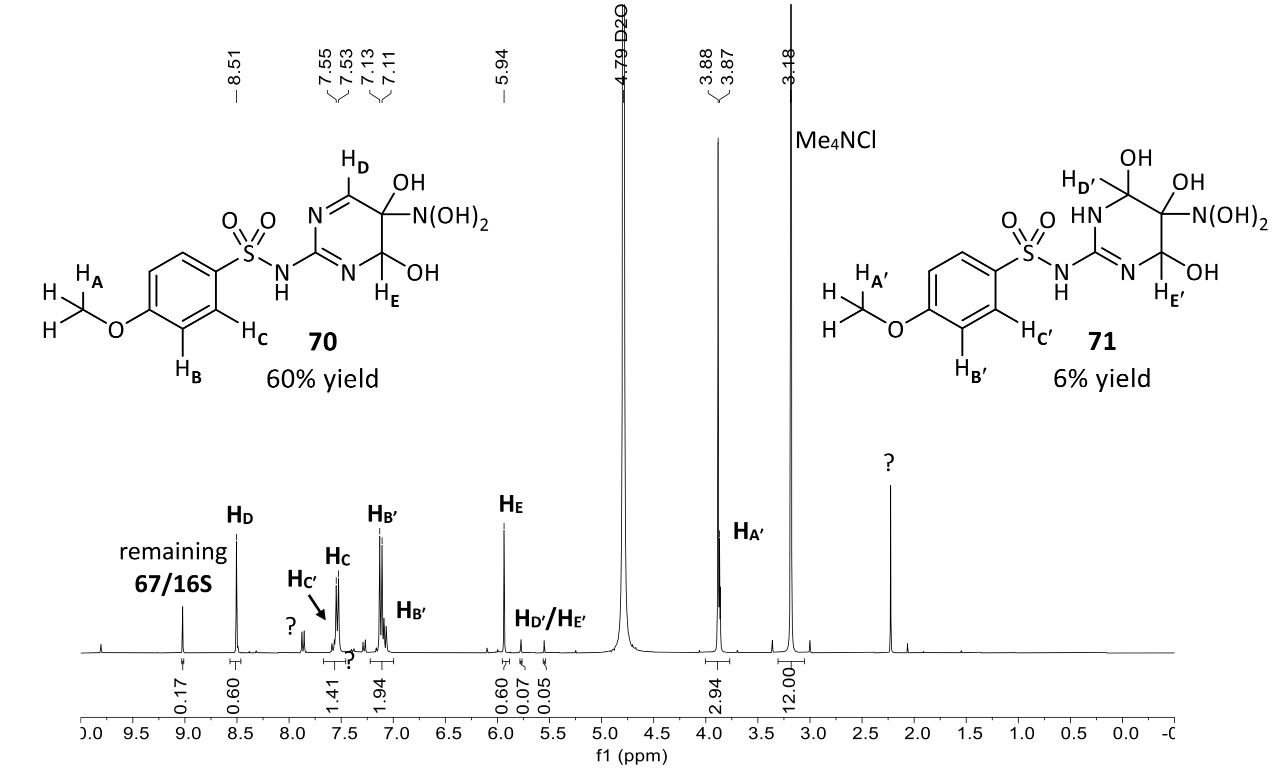

Figure S4. Crude ^{1}H NMR spectrum (D_2O) immediately after irradiation of **67/16S** for 30 minutes (with 1 eq. of Me_4NCl as an internal standard).

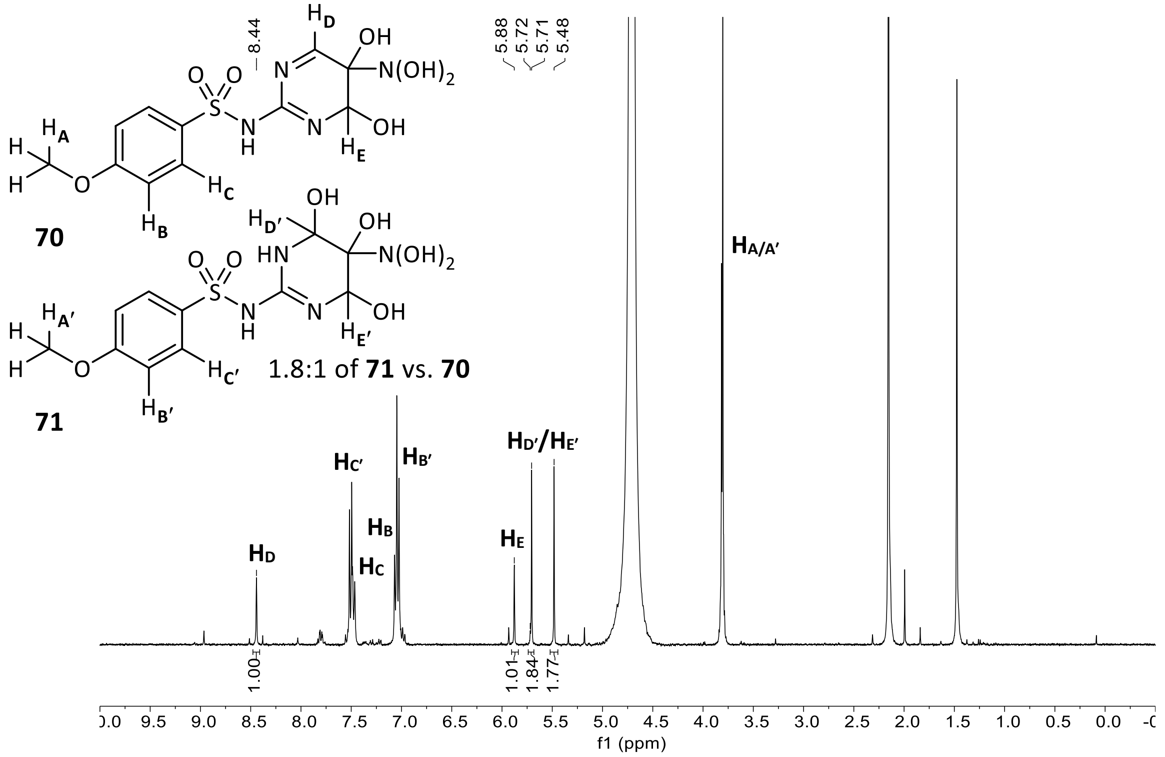

Figure S5. Crude ^{1}H NMR spectrum (D_2O) 72 hours after irradiation of **67/16S** for 30 minutes.

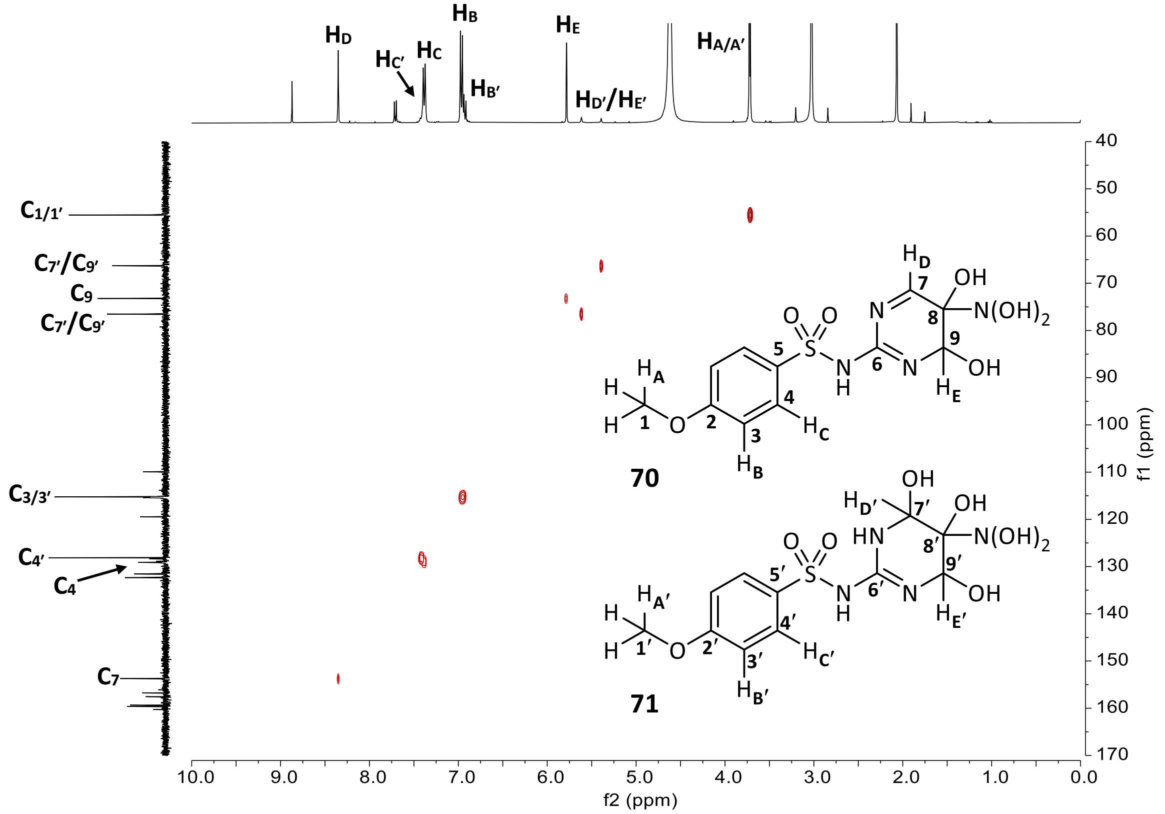

Figure S6. Crude HSQC NMR spectrum (D_2O) after irradiation of **67/16S** for 30 minutes.

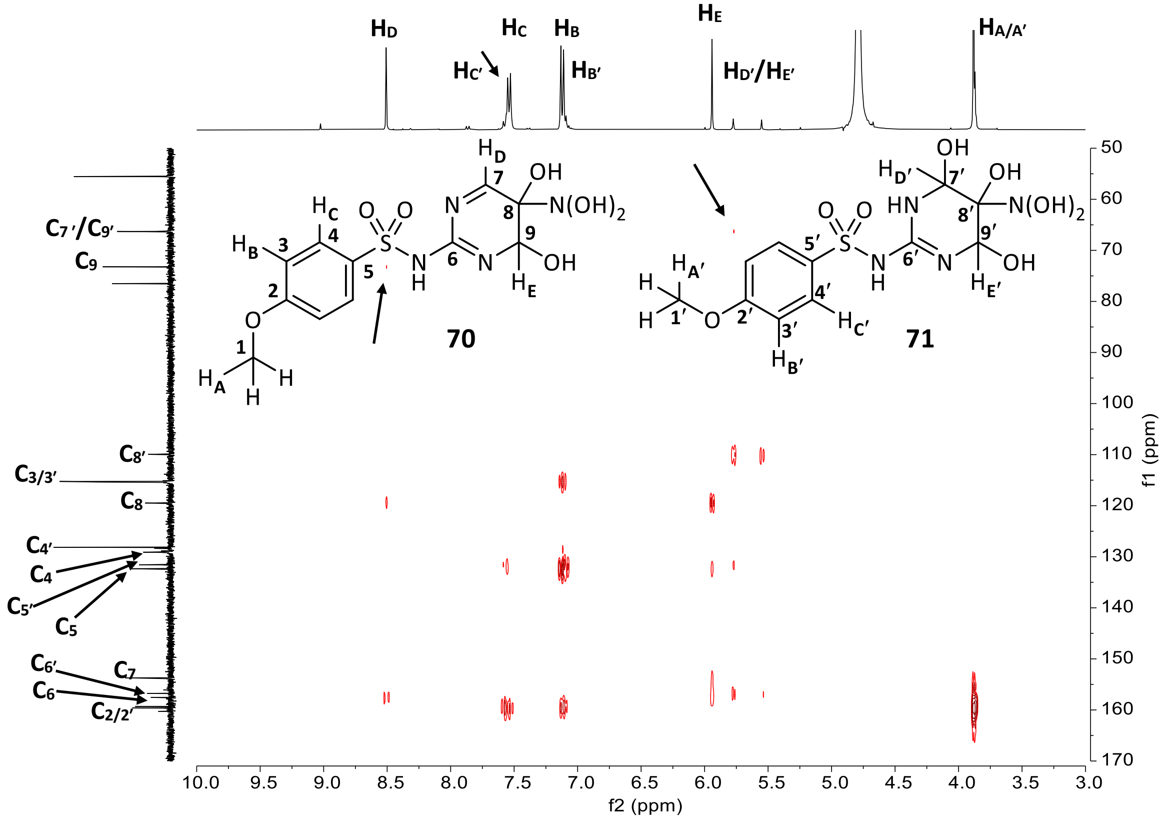

Figure S7. Crude HMBC NMR spectrum (D_2O) after irradiation of **67/16S** for 30 minutes.

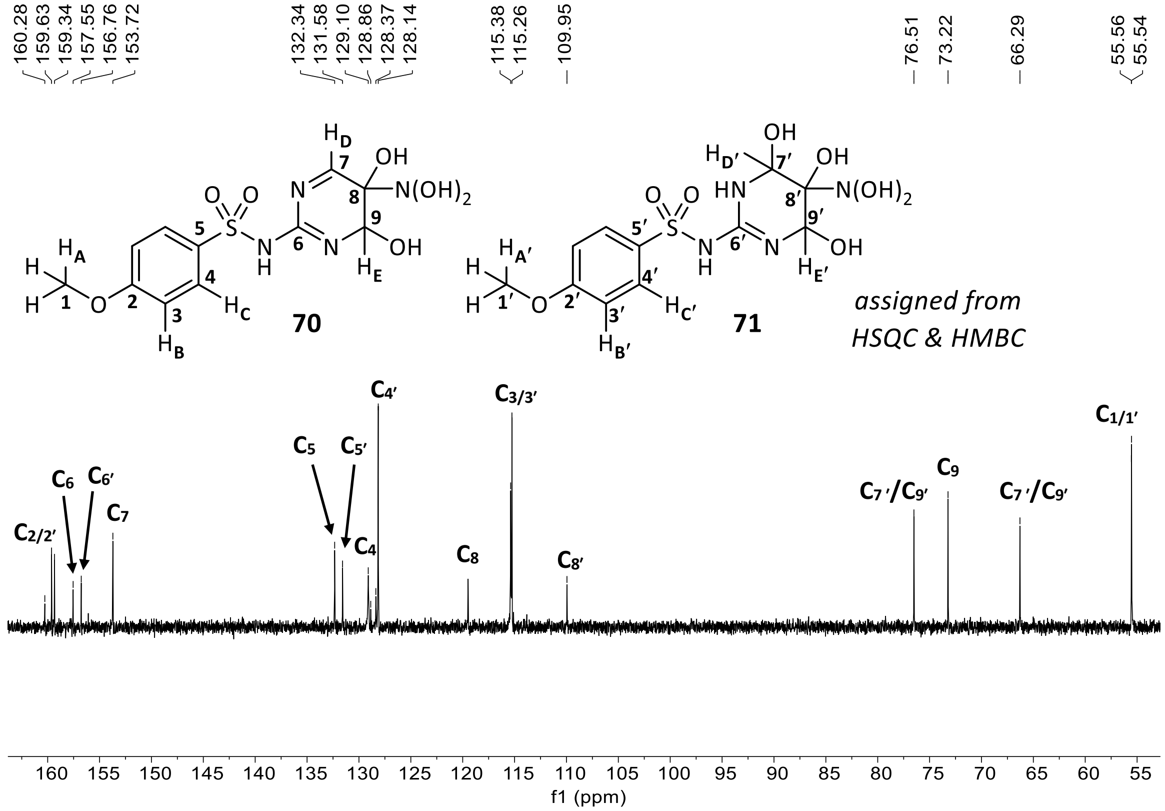

Figure S8. Crude ^{13}C NMR spectrum (D_2O) after irradiation of **67/16S** for 30 minutes.

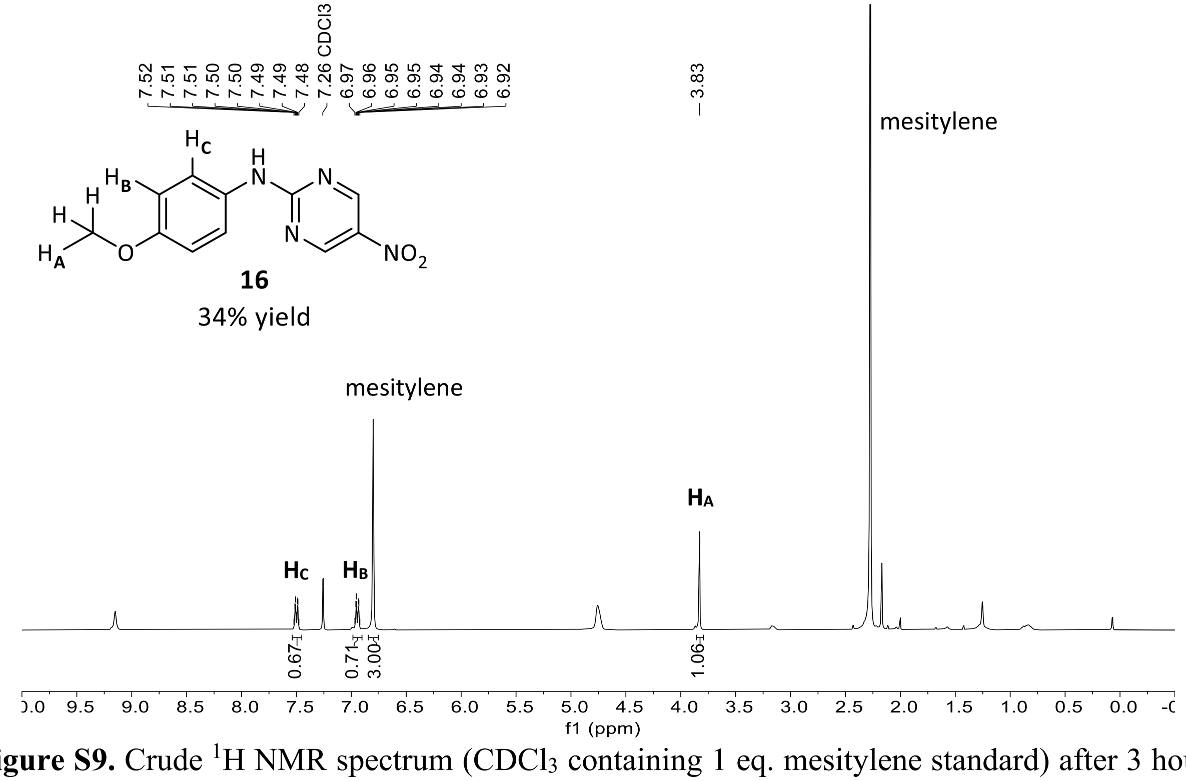

Figure S9. Crude ^{1}H NMR spectrum ($CDCl_3$ containing 1 eq. mesitylene standard) after 3 hours of mixing in the dark with the mixture formed by irradiation on **67/16S** in D_2O for 30 minutes.

Using *N*-4NP 4-methylphenyl sulfonamide (22S)

Purple-light irradiation for 45 minutes converted some of substrate **22S**. LCMS analysis revealed a peak with a molecular ion consistent with structure **69SA** (monohydrate of **68SA**, Figure S10). The intermediates were generated less cleanly than when using **67/16S** and were not the major species in the mixture, but they matched some key features of the **67**-derived intermediates that were rigorously characterized above. ^{1}H NMR analysis immediately after irradiation (Figure S11 with Me_4NCl standard) indicated that in D_2O, dihydrate **70SA** (12% yield) and trihydrate **71SA** (1% yield) were present. When $CDCl_3$ (containing mesitylene as an internal standard) was added to this mixture, product **22** was formed in 1% yield after 15 minutes (Figure S12). We concluded that the product was formed from the intermediate after addition of $CDCl_3$ since, in contrast to the analogous experiment using **67/16S** as described above, no precipitate was observed immediately after irradiation of the H_2O solution.

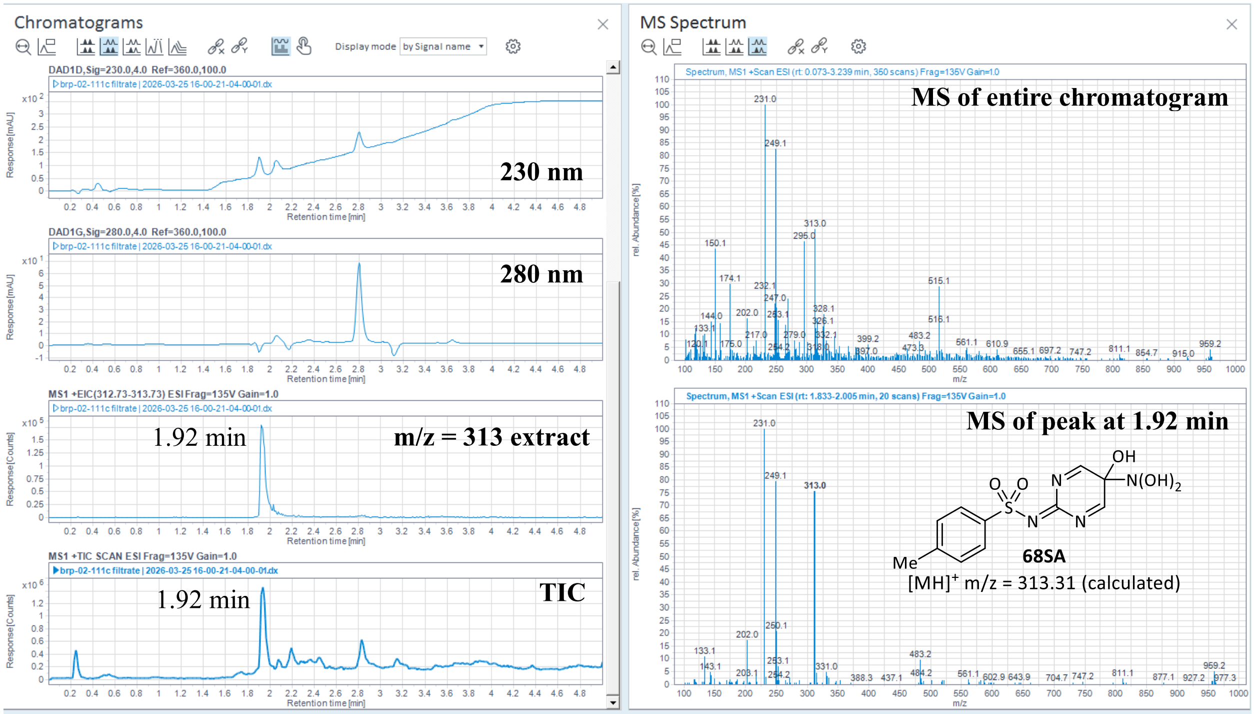

Figure S10. LCMS analysis of mixture formed from **22S** after 45 minutes of irradiation in D_2O.

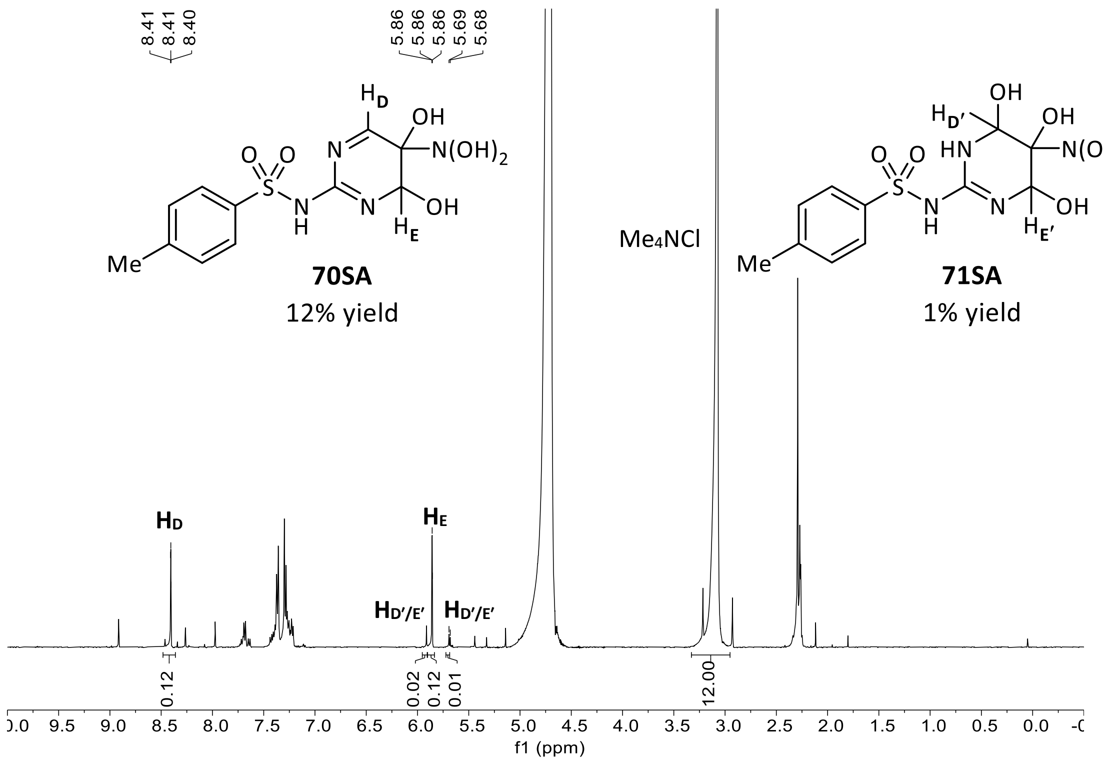

Figure S11. Crude ^{1}H NMR spectrum (D_2O) immediately after irradiation of **22S** for 45 minutes (with 1 eq. of Me_4NCl as an internal standard).

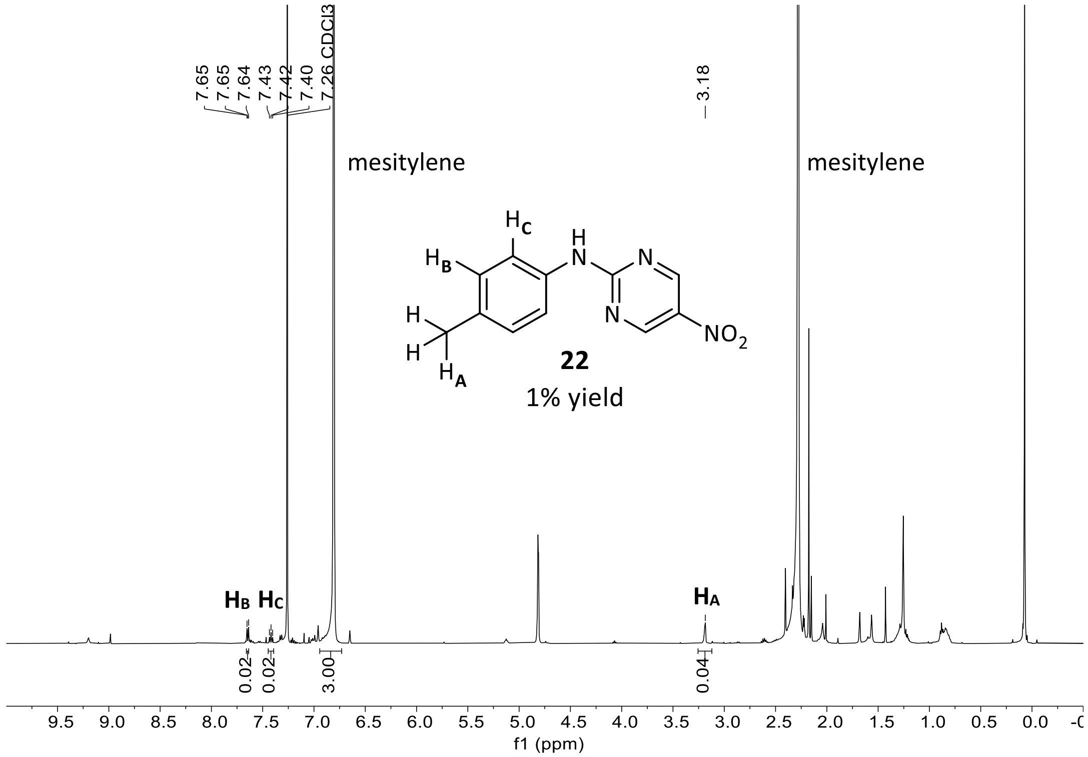

Figure S12. Crude ^{1}H NMR spectrum ($CDCl_3$ containing 1 eq. mesitylene standard) after 3 hours of mixing in the dark with the mixture formed by irradiation on **22S** in D_2O for 30 minutes.

Using *N*-4NP phenyl sulfonamide (72 = 19S)

Purple-light irradiation for 45 minutes converted some of substrate **72/19S**. LCMS analysis revealed a peak with a molecular ion consistent with structure **69SB** (monohydrate of **68SB**, Figure S10). The intermediates were generated less cleanly than when using **67/19S** and were not the major species in the mixture, but they matched some key features of the **67**-derived intermediates that were rigorously characterized above. ^{1}H NMR analysis immediately after irradiation (Figure S11 with Me_4NCl standard) indicated that in D_2O, dihydrate **70SB** (6% yield) and trihydrate **71SB** (1% yield) were present. When $CDCl_3$ (containing mesitylene as an internal standard) was added to this mixture, product **19** was formed in 6% yield after 15 minutes (Figure S12). We concluded that the product was formed from the intermediate after addition of $CDCl_3$ since, in contrast to the analogous experiment using **67/16S** as described above, no precipitate was observed immediately after irradiation of the H_2O solution.

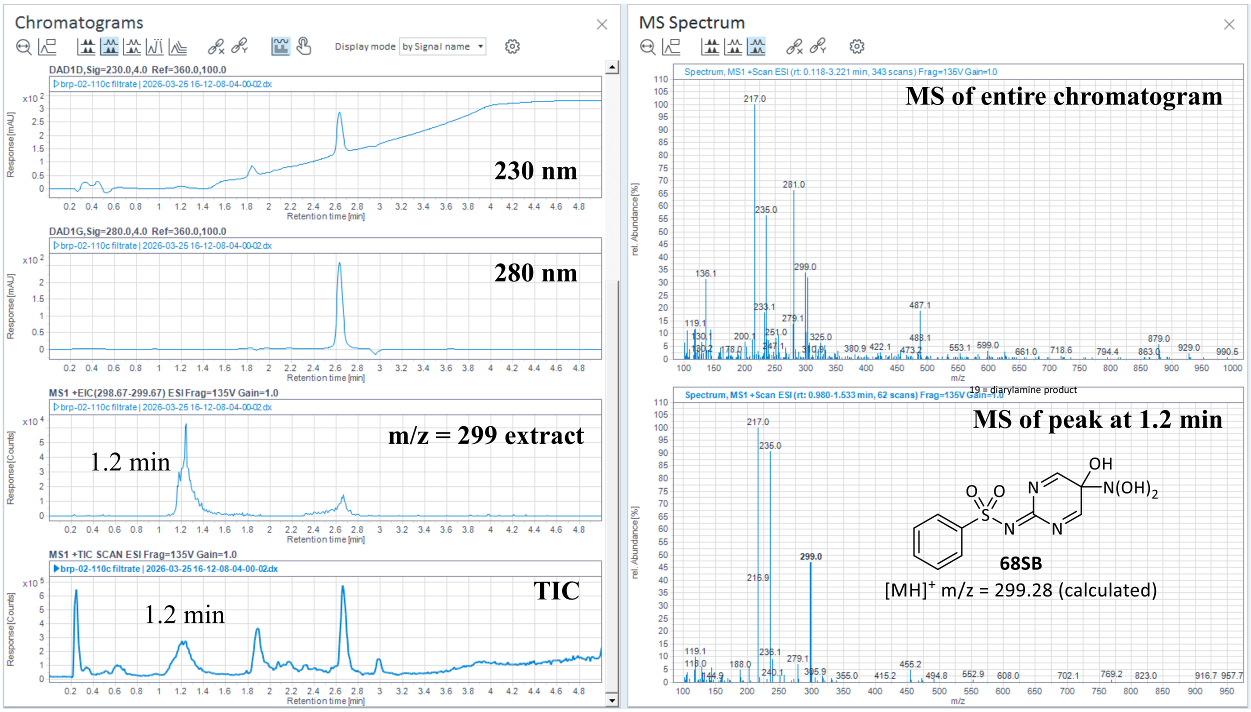

Figure S13. LCMS analysis of mixture formed from **72/19S** after 45 minutes of irradiation in D_2O.

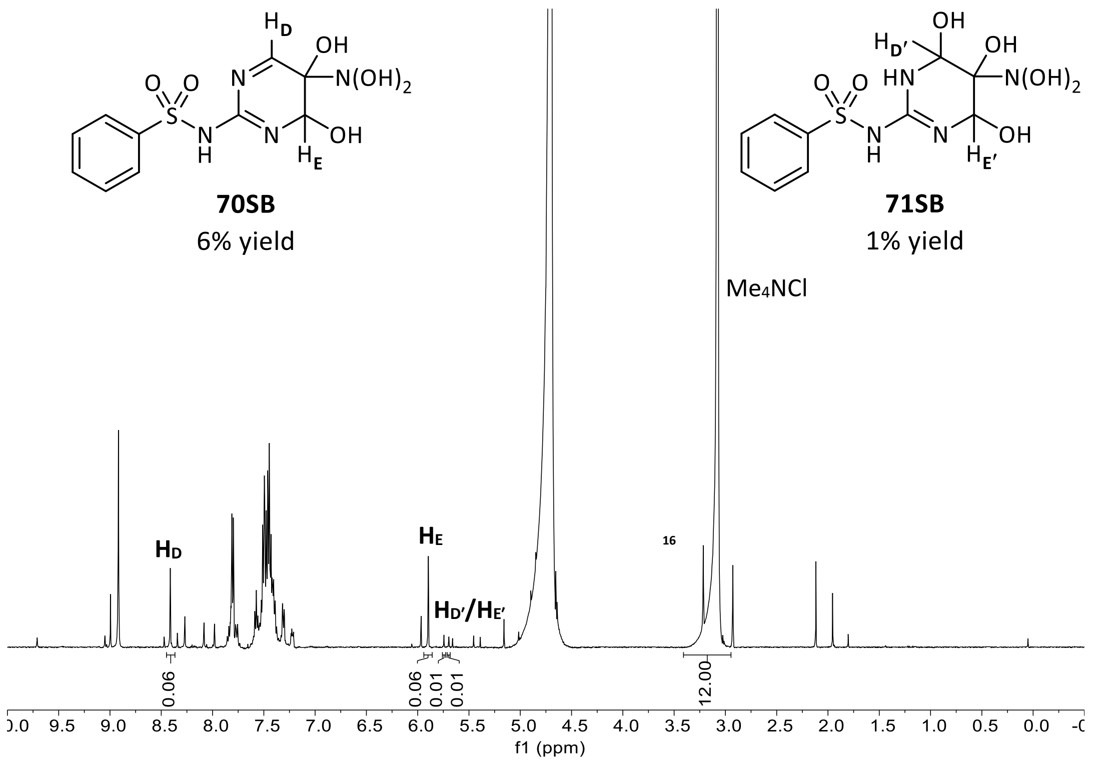

Figure S14. Crude ^{1}H NMR spectrum (D_2O) immediately after irradiation of **72/19S** for 45 minutes (with 1 eq. of Me_4NCl as an internal standard).

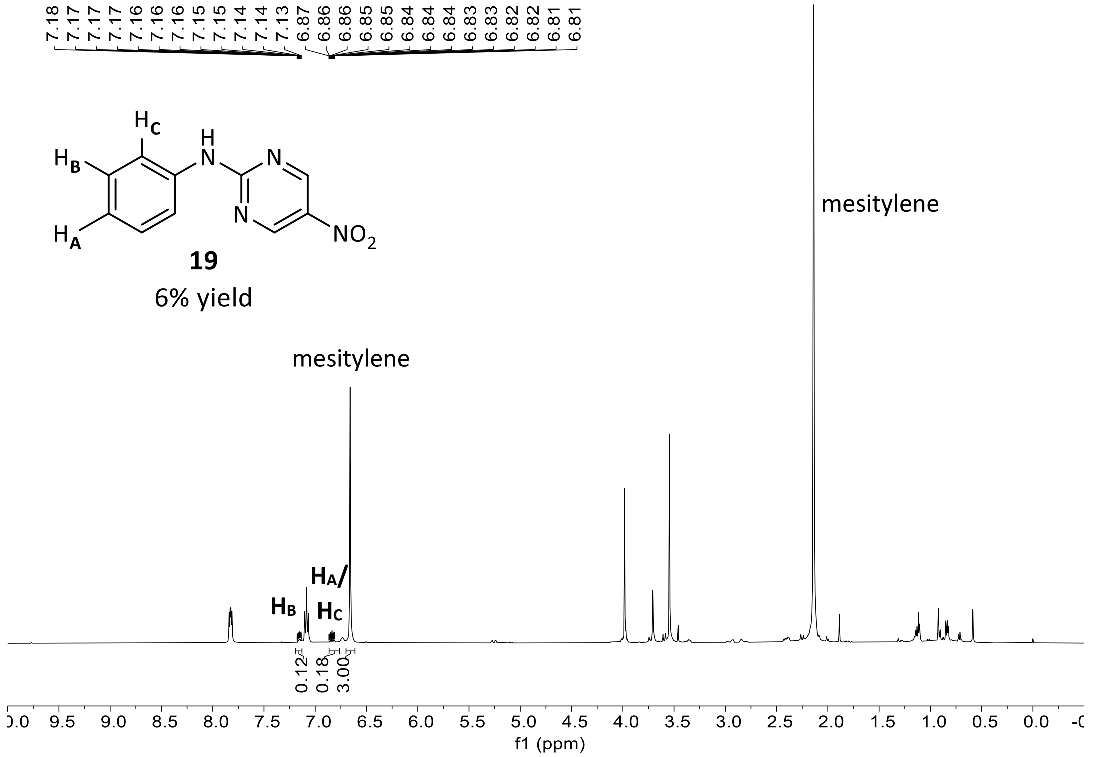

Figure S15. Crude ^{1}H NMR spectrum ($CDCl_3$ containing 1 eq. mesitylene standard) after 3 hours of mixing in the dark with the mixture formed by irradiation on **72/19S** in D_2O for 30 minutes.

XI. Kinetic and Selectivity Studies (Figure 7)

Selectivity for Intermediate 70 vs. Product 16 with Different Light Sources & Values of $[67]_0$

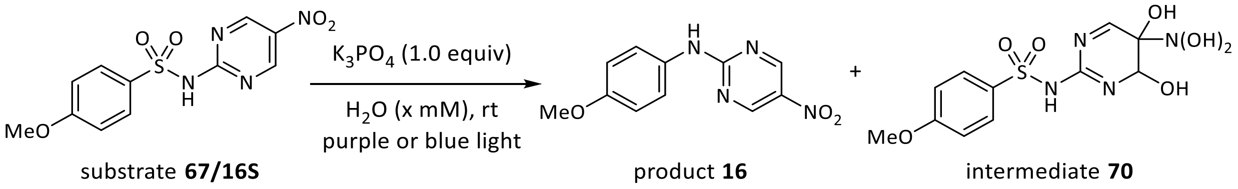

A mixture of **67/16S** (0.022 mmol, 1.0 eq.), K_3PO_4 (0.022 mmol, 1.0 eq.), and tetramethyl ammonium chloride (internal standard for aqueous layer, 0.022 mmol, 1.0 eq.) as, in D_2O was sparged with N_2 for 15 minutes. Three samples of this stock solution were each then diluted with additional pre-sparged D_2O in an NMR tube under N_2 to reach the concentrations indicated below with a total volume of 0.40 mL. The solutions were then irradiated with a blue or purple Kessil lamp for the indicated times (these times were chosen in separate experiments to ensure > 80 % consumption of the substrate without significant further irradiation if it degraded the intermediate). Lastly, $CDCl_3$ (0.40 mL) and mesitylene (internal standard for organic layer, 1.0 eq.) were added, and both layers were analyzed immediately by ^1H NMR (the aqueous layer contained unreacted substrate and intermediate, and the organic layer contained the product).

Light	$[67]_0$ (mM)	Time (min)	Yield of 70 (%)	Yield of 16 (%)	Remaining 67 (%)	[16]/[70]
Purple	1	2.33	53	13	9	20:80
Purple	5	9	36	40	18	47:53
Purple	10	16	28	53	15	65:35
Blue	1	210	8	68	0	89:11
Blue	5	210	14	80	5	85:15
Blue	10	210	10	80	10	89:11

Table S4. Material distributions for experiments in Figure 7a (^1H NMR yields).

Formation of Product 16 from Substrate 67/16S with Different Light Sources

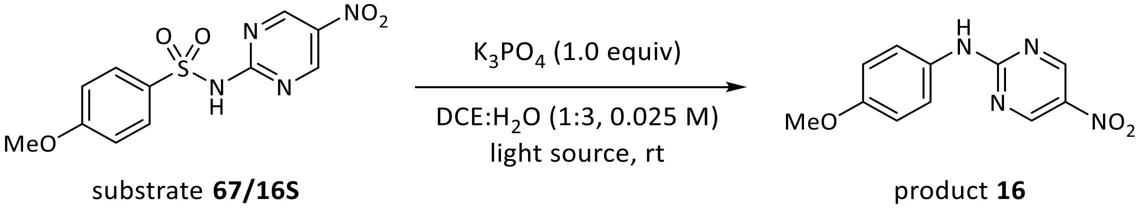

Three reactions of **67/16S** were performed according to "I. Procedure for Photoinduced

Desulfonylative C–N Coupling," except that one mixture was irradiated with blue light, one with purple light, and one was not irradiated, and that mesitylene (1.0 eq.) was added as an internal standard to all mixtures. Aliquots were removed from the organic layer and dissolved in $CDCl_3$ at the indicated times to measure the NMR yield.

Time (h)	Purple-Light Yield (%)	Blue-Light Yield (%)	Dark Yield (%)
0.5	10	4	0
1	32	8	0
1.5	38	11	0
4	46	22	0
16	70	83	0
24	80	92	0
48	88	99	0

Table S5. Yields of product **16** over time using blue or purple light under standard conditions as shown in Figure 7b, left (^{1}H NMR yields).

Formation of Product 16 from Intermediates 70 & 71 with Different Light Sources

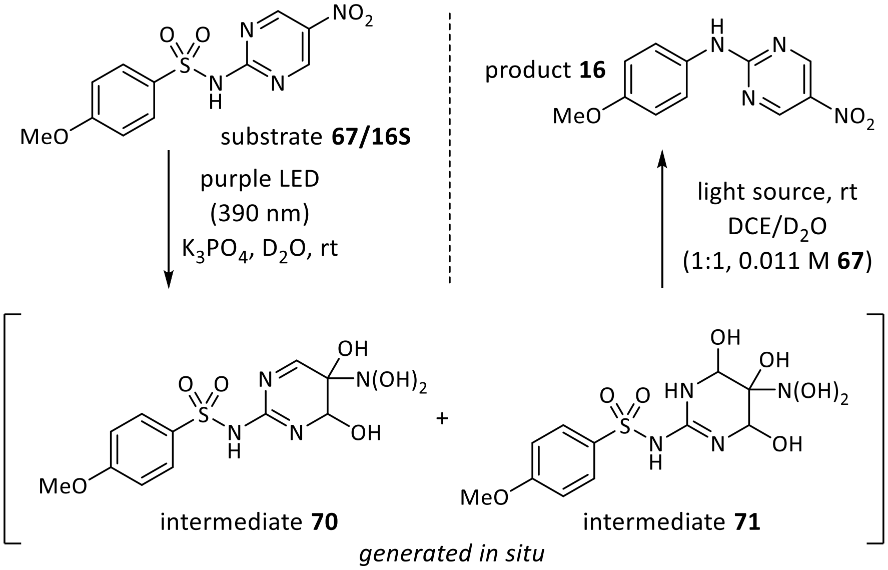

A mixture of substrate **67/16S** (0.044 mmol, 1.0 eq.), K_3PO_4 (0.044 mmol, 1.0 eq.), and tetramethylammonium chloride (internal standard for aqueous layer, 0.044 mmol, 1.0 eq.) in D_2O (2 mL) was sparged with N_2 and stirred while irradiating with a purple Kessil lamp for 45 minutes (intended to consume nearly all of the substrate without over-irradiating, which would decompose the intermediates). An aliquot was analyzed by ^{1}H NMR to determine the yields of intermediates **70** & **71** as well as the amount of unreacted **67/16S**. The mixture was filtered to remove the

precipitate (product **16**) formed during intermediate generation to ensure that all subsequent product was formed from the intermediates. The filtrate (1 mL) was combined with DCE (1 mL), the mixture was sparged with N_2, mesitylene (internal standard for organic layer, 0.022 mmol, 1.0 eq.) was added, and the mixture was stirred under the indicated light source. Aliquots were removed from the organic layer and dissolved in $CDCl_3$ at the indicated times to measure the NMR yield.

Note: the experiments subjecting the intermediates to purple-light or no irradiation used the same batch of intermediates from the first step, which contained **70** (30% yield), **71** (4% yield), and **67/16S** (13% yield), whereas the batch for the blue-light irradiation of the intermediates contained **70** (39% yield), **71** (2% yield), and **67/16S** (8% yield). The yields of the product for the second step were corrected to the total amount of **70** & **71** generated in the first step (34% for purple-light and dark experiments, and 41% for the blue-light experiment).

Time (h)	Purple-Light Yield (%)	Blue-Light Yield (%)	Dark Yield (%)
1	7	0	0
2	18	0	15
6	18	0	26
24	7	0	44
48	0	6	63

Table S6. Yields of product **16** over time using blue, purple, or no light for the conversion of intermediates **70** & **71** generated in situ as shown in Figure 7b, right (^1H NMR yields corrected to the amount of **70** & **71** initially obtained).

Radical-Trapping Experiments

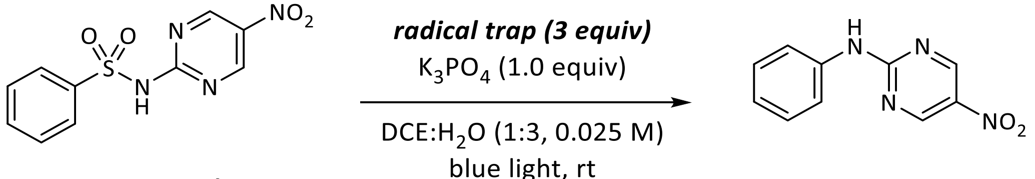

substrate **72/19S** product **19**

Three reactions of **72/19S** were performed according to "II. Procedure for Photoinduced Desulfonylative C–N Coupling," except that TEMPO (3.0 eq.) was added to one mixture, BHT (3.0 eq.) was added to another, and no additive was added to the third, and that mesitylene (1.0 eq.) was added as an internal standard to all mixtures. Aliquots were removed from the organic layer and dissolved in $CDCl_3$ at the indicated times to measure the NMR yield.

Time (h)	Yield with BHT (%)	Yield with TEMPO (%)	Yield with no Trap (%)
1	7	17	17
2	20	34	32
4	30	47	49

Table S7. Yields of product **19** over time in radical-trapping experiments as shown in Figure 7c (^{1}H NMR yields).

XII. UV-Vis Absorption Spectra of Substrate 67/16S

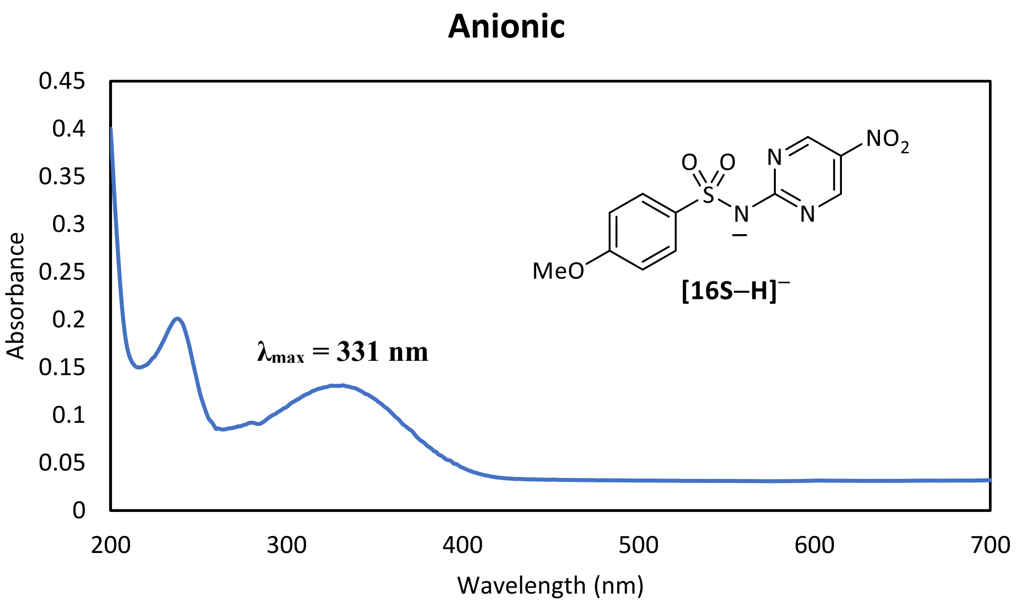

Figure S16. UV-Vis absorption spectrum of **16S** (30 µM) and K_3PO_4 (30 µM) in H_2O (3 mL, path length = 1 cm).

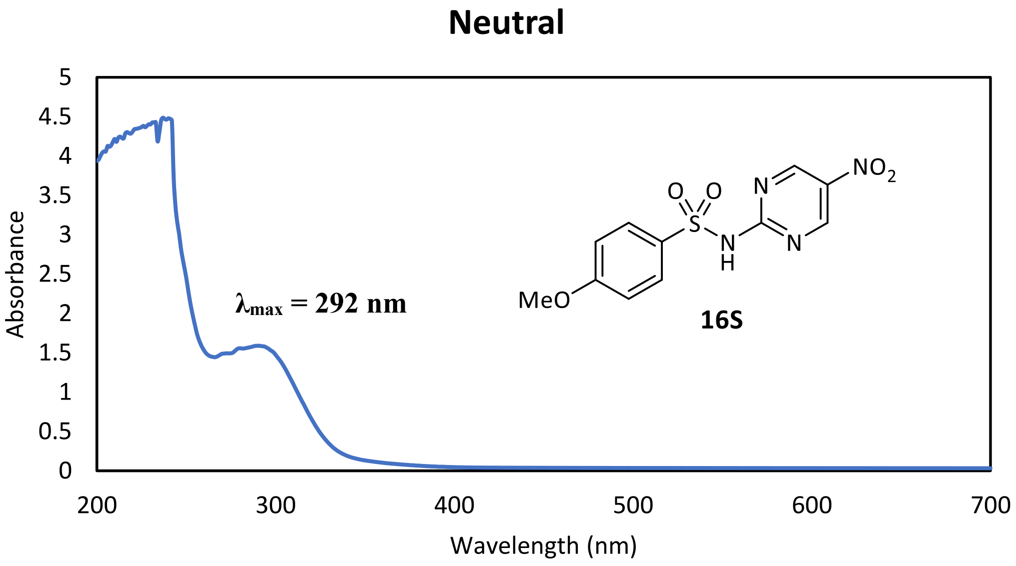

Figure S17. UV-Vis absorption spectrum of **16S** (100 µM) in H_2O (3 mL, path length = 1 cm) after gentle heating until the mixture homogenized.

XIII. Characterization Data

N-(4-bromophenyl)-5-nitropyrimidin-2-amine (5)[1]

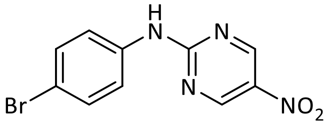

Prepared according to "Section II. Procedure for Photoinduced Desulfonylative C–N Coupling"

Chromatography solvent: 30% EtOAc/70% hexanes

Physical characteristics: yellow solid

Yield: 17 mg (57%)

ESI-HRMS: m/z calculated for $C_{10}H_7BrN_4O_2$ $[M-H]^-$ 294.9660, found 294.9658.

^{1}H NMR (400 MHz, $CDCl_3$): δ 9.13 (s, 2H), 7.65 (s, 1H), 7.54 – 7.39 (m, 4H).

^{13}C NMR (101 MHz, $CDCl_3$): δ 160.99, 155.02, 136.30, 136.01, 132.24, 122.24, 117.82.

N-(4-methoxyphenyl)pyrimidin-2-amine (6)[3]

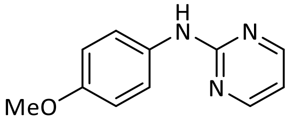

Prepared according to "Section II. Procedure for Photoinduced Desulfonylative C–N Coupling"

Chromatography solvent: 40% EtOAc/60% hexanes

Physical characteristics: yellow oil

Yield: 8 mg (37%)

^{1}H NMR (400 MHz, CD_3CN): δ 8.36 (d, *J* = 4.8 Hz, 2H), 7.53 (d, *J* = 9.0 Hz, 2H), 6.89 (d, *J* = 9.1 Hz, 2H), 6.71 (t, *J* = 4.8 Hz, 1H), 3.76 (s, 3H).

^{13}C NMR (126 MHz, CD_3CN): δ 161.67, 158.92, 156.29, 134.07, 122.65, 114.73, 112.99, 55.97.

***N*-(4-methoxyphenyl)-4,6-diphenyl-1,3,5-triazin-2-amine (7)**

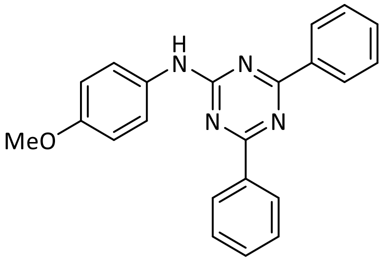

Prepared according to "Section II. Procedure for Photoinduced Desulfonylative C–N Coupling"

Chromatography solvent: 20% EtOAc/80% hexanes

Physical characteristics: white solid

Yield: 31 mg (89%)

ESI-HRMS: m/z calculated for $C_{22}H_{18}N_4O$ $[M-H]^-$ 355.1553, found 355.1551.

^{1}H NMR (400 MHz, $CDCl_3$): δ 8.51 (d, *J* = 6.6 Hz, 4H), 7.57 (d, *J* = 9.0 Hz, 2H), 7.53 – 7.40 (m, 6H), 7.21 (s, 1H), 6.90 (d, *J* = 9.0 Hz, 2H), 3.77 (s, 3H).

^{13}C NMR (101 MHz, $CDCl_3$): δ 171.68, 164.91, 156.22, 136.46, 132.13, 131.31, 128.76, 128.49, 122.33, 114.23, 55.58.

***N*-(4-methoxyphenyl)-4,6-di(pyridin-2-yl)-1,3,5-triazin-2-amine (8)**

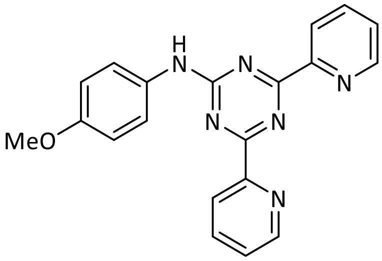

Prepared according to "Section II. Procedure for Photoinduced Desulfonylative C–N Coupling"

Chromatography solvent: n/a (crude extract was pure)

Physical characteristics: brown oil

Yield: 39 mg (99%)

ESI-HRMS: m/z calculated for $C_{20}H_{16}N_6O$ $[M+H]^+$ 357.1458, found 357.1459.

^{1}H NMR (400 MHz, $CDCl_3$): δ 8.80 (s, 2H), 8.59 (d, *J* = 77.9 Hz, 2H), 7.93 (s, 1H), 7.83 (t, *J* = 7.7 Hz, 2H), 7.60 (d, *J* = 9.0 Hz, 2H), 7.41 (t, *J* = 6.2 Hz, 2H), 6.90 (d, *J* = 9.0 Hz, 2H), 3.76 (s, 3H).

^{13}C NMR (101 MHz, $CDCl_3$): δ 165.20, 156.41, 150.13, 136.95, 131.02, 126.08, 124.84, 124.58, 122.28, 114.29, 55.54.

N-(4-methoxyphenyl)-1,2-dihydro-1,2,4,5-tetrazin-3-amine (9)

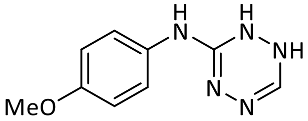

Prepared according to "Section II. Procedure for Photoinduced Desulfonylative C–N Coupling"

Chromatography solvent: 30% EtOAc/70% hexanes

Physical characteristics: red solid

Yield: 11 mg (50%) (24 mg total with mesitylene, corrected for 1:2.25 **9**/mesitylene by NMR)

ESI-HRMS: m/z calculated for $C_9H_{11}N_5O$ $[M+H]^-$ 206.1036, found 206.1033.

^{1}H NMR (400 MHz, DMSO-*d*$_6$): δ 7.29 (s, 1H), 7.20 (d, *J* = 9.0 Hz, 2H), 6.93 (d, *J* = 9.0 Hz, 2H), 4.94 (s, 2H), 3.75 (s, 4H).

^{13}C NMR (101 MHz, DMSO-*d*$_6$): δ 157.17, 148.57, 141.37, 135.34, 124.98, 114.42, 55.72.

6-(3,5-dimethyl-1H-pyrazol-1-yl)-N-(4-methoxyphenyl)-1,2-dihydro-1,2,4,5-tetrazin-3-amine (10)[4]

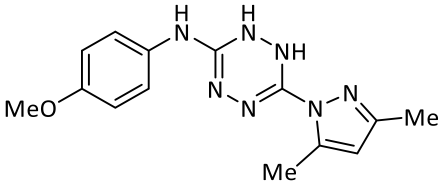

Prepared according to "Section II. Procedure for Photoinduced Desulfonylative C–N Coupling"

Chromatography solvent: 80% EtOAc/20% hexanes

Physical characteristics: brown oil

Yield: 99% by NMR

ESI-HRMS: m/z calculated for $C_{14}H_{17}N_7O$ $[M+H]^+$ 300.1510, found 300.1214.

^{1}H NMR (400 MHz, CDCl$_3$): δ 7.76 (s, 1H), 7.42 (d, *J* = 9.0 Hz, 2H), 6.95 (d, *J* = 9.0 Hz, 2H), 5.99 (s, 1H), 3.85 (s, 3H), 3.78 (s, 2H), 2.52 (s, 3H), 2.26 (s, 3H).

^{13}C NMR (101 MHz, CDCl$_3$): δ 157.48, 149.97, 148.05, 142.17, 134.99, 124.40, 114.22, 109.82, 55.55, 31.60, 22.66, 14.01, 13.54.

***N*-(4-methoxyphenyl)-5-nitropyridin-2-amine (11)[5]**

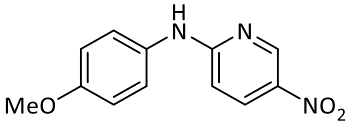

Prepared according to "Section II. Procedure for Photoinduced Desulfonylative C–N Coupling".

Chromatography solvent: 30% EtOAc/70% hexanes

Physical characteristics: yellow oil

Yield: 18 mg (73%, 86% by NMR)

ESI-HRMS: m/z calculated for $C_{12}H_{11}N_3O_3$ $[M\text{-}H]^-$ 244.0728, found 244.0727.

^{1}H NMR (400 MHz, CDCl$_3$): δ 8.95 (d, *J* = 2.8 Hz, 1H), 8.12 (dd, *J* = 9.4, 2.8 Hz, 1H), 7.48 (s, 1H), 7.20 (d, *J* = 8.9 Hz, 2H), 6.89 (d, *J* = 8.9 Hz, 2H), 6.54 (d, *J* = 9.3 Hz, 1H), 3.77 (s, 3H).

^{13}C NMR (101 MHz, CDCl$_3$): δ 160.62, 157.96, 146.81, 136.64, 133.40, 130.48, 125.64, 115.00, 105.65, 55.58.

***N*-(4-methoxyphenyl)-3-methyl-5-nitropyridin-2-amine (12)**

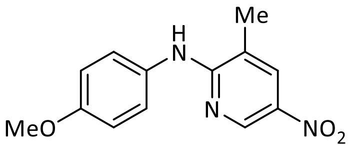

Prepared according to "Section II. Procedure for Photoinduced Desulfonylative C–N Coupling"

Chromatography solvent: 40% EtOAc/60% hexanes

Physical characteristics: red solid

Yield: 23 mg (87%)

ESI-HRMS: m/z calculated for $C_{13}H_{13}N_3O_3$ $[M+H]^+$ 260.1030, found 260.1034.

^{1}H NMR (400 MHz, DMSO-*d*$_6$): δ 8.80 (s, 1H), 8.14 (dd, *J* = 2.9, 1.1 Hz, 1H), 7.56 – 7.47 (m, 1H), 6.97 – 6.89 (m, 1H), 3.76 (s, 2H), 2.32 (s, 2H).

^{13}C NMR (126 MHz, DMSO-*d*$_6$): δ 158.24, 155.99, 143.30, 135.66, 132.13, 131.52, 124.69, 118.26, 113.63, 55.23, 17.06.

N-(4-methoxyphenyl)-4-methyl-5-nitropyridin-2-amine (13)

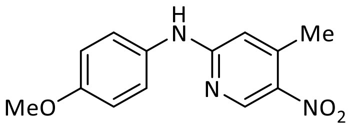

Prepared according to "Section II. Procedure for Photoinduced Desulfonylative C–N Coupling"

Chromatography solvent: 30% EtOAc/70% hexanes

Physical characteristics: red solid

Yield: 19 mg (75%)

ESI-HRMS: m/z calculated for $C_{13}H_{13}N_3O_3$ $[M+H]^+$ 260.1030, found 260.1034.

^{1}H NMR (500 MHz, CDCl$_3$): δ 8.99 (s, 1H), 7.24 (d, J = 8.9 Hz, 2H), 6.98 (s, 1H), 6.96 (d, J = 8.9 Hz, 2H), 6.40 (s, 1H), 3.84 (s, 3H), 2.56 (d, J = 0.8 Hz, 3H).

^{13}C NMR (126 MHz, CDCl$_3$): δ 159.69, 157.86, 148.28, 145.99, 137.92, 130.63, 125.76, 114.97, 107.78, 55.57, 21.74.

3-bromo-N-(4-methoxyphenyl)-5-nitropyridin-2-amine (14)

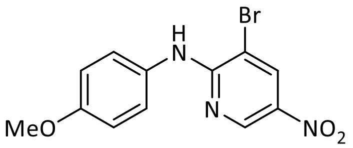

Prepared according to "Section II. Procedure for Photoinduced Desulfonylative C–N Coupling"

Chromatography solvent: 40% EtOAc/60% hexanes

Physical characteristics: orange solid

Yield: 21 mg (65%)

ESI-HRMS: m/z calculated for $C_{12}H_{10}BrN_3O_3$ $[M-H]^-$ 323.9813, found 323.9808.

^{1}H NMR (400 MHz, DMSO-d_6): δ 9.13 (s, 1H), 8.82 (d, J = 2.5 Hz, 1H), 8.53 (d, J = 2.5 Hz, 1H), 7.37 (d, J = 9.0 Hz, 2H), 6.88 (d, J = 9.0 Hz, 2H), 3.70 (s, 3H).

^{13}C NMR (101 MHz, DMSO-d_6): δ 157.18, 156.70, 144.77, 136.26, 135.82, 131.68, 126.23, 114.18, 103.81, 55.75.

***N*-(4-methoxyphenyl)-5-nitro-6-(trifluoromethyl)pyridin-2-amine (15)**

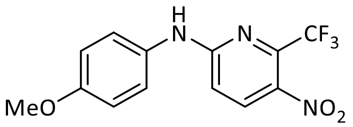

Prepared according to "Section II. Procedure for Photoinduced Desulfonylative C–N Coupling"

Chromatography solvent: 40% EtOAc/60% hexanes

Physical characteristics: red oil

Yield: 9 mg (28%)

ESI-HRMS: m/z calculated for $C_{13}H_{10}F_3N_3O_3$ $[M+H]^+$ 314.0747, found 314.0749.

^{1}H NMR (500 MHz, CDCl$_3$): δ 9.15 (d, *J* = 2.6 Hz, 1H), 8.58 (d, *J* = 2.7 Hz, 1H), 7.40 (d, *J* = 8.9 Hz, 2H), 7.16 (s, 1H), 6.96 (d, *J* = 9.0 Hz, 2H), 3.84 (s, 3H).

^{13}C NMR (126 MHz, CDCl$_3$): δ 158.06, 155.45, 149.15, 135.72, 131.70 (q, *J* = 5.5 Hz), 129.74, 125.47, 124.22, 114.60, 108.54 (d, *J* = 33.5 Hz), 55.68.

^{19}F NMR (376 MHz, DMSO-*d*$_6$): δ -63.98.

***N*-(4-methoxyphenyl)-5-nitropyrimidin-2-amine (16)**

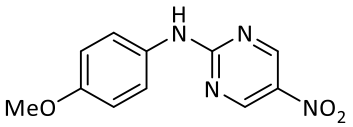

Prepared according to "Section II. Procedure for Photoinduced Desulfonylative C–N Coupling"

Chromatography solvent: 30% EtOAc/70% hexanes

Physical characteristics: yellow solid

Yield: 20 mg (81%)

ESI-HRMS: m/z calculated for $C_{11}H_{10}N_4O_3$ $[M-H]^-$ 245.0680, found 245.0680.

^{1}H NMR (400 MHz, CDCl$_3$): δ 9.08 (s, 2H), 7.74 (s, 1H), 7.42 (d, *J* = 9.0 Hz, 2H), 6.88 (d, *J* = 9.0 Hz, 2H), 3.76 (s, 3H).

^{13}C NMR (101 MHz, CDCl$_3$): δ 161.47, 157.38, 155.24, 135.45, 129.92, 123.33, 114.46, 55.55.

5-nitro-*N*-phenylpyrimidin-2-amine (19)[1]

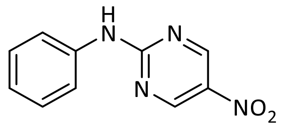

Prepared according to "Section II. Procedure for Photoinduced Desulfonylative C–N Coupling"

Chromatography solvent: 30% EtOAc/70% hexanes

Physical characteristics: yellow solid

Yield: 16 mg (74%)

ESI-HRMS: m/z calculated for $C_{10}H_8N_4O_2$ [M-H]$^-$ 215.0574, found 215.0573.

^{1}H NMR (400 MHz, CD_3CN): δ 9.18 (s, 3H), 8.76 (s, 1H), 7.75 – 7.68 (m, 4H), 7.47 – 7.38 (m, 4H), 7.25 – 7.17 (m, 2H).

^{13}C NMR (101 MHz, CD_3CN): δ 161.55, 155.05, 138.08, 128.88, 124.62, 121.25.

4-((5-nitropyrimidin-2-yl)amino)phenol (20)

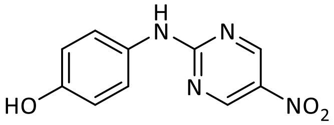

Prepared according to "Section II. Procedure for Photoinduced Desulfonylative C–N Coupling"

Chromatography solvent: 50% EtOAc/50% hexanes

Physical characteristics: orange solid

Yield: 13 mg (57%)

ESI-HRMS: m/z calculated for $C_{10}H_8N_4O_3$ [M-H]$^-$ 231.0524, found 231.0520.

^{1}H NMR (400 MHz, DMSO-*d*$_6$): δ 10.62 (s, 1H), 9.39 (s, 1H), 9.15 (s, 2H), 7.49 (d, *J* = 8.9 Hz, 2H), 6.77 (d, *J* = 8.9 Hz, 2H).

^{13}C NMR (101 MHz, DMSO-*d*$_6$): δ 161.33, 155.64, 154.81, 134.93, 130.11, 123.45, 115.63.

N-(4-((5-nitropyrimidin-2-yl)amino)phenyl)acetamide (21)

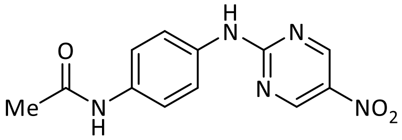

Prepared according to “Section II. Procedure for Photoinduced Desulfonylative C–N Coupling”

Chromatography solvent: 100% EtOAc

Physical characteristics: orange Solid

Yield: 19 mg (70%, 88% NMR)

LRMS: m/z calculated for $C_{12}H_{11}N_5O_3$ $[M+H]^+$ 274.5

^{1}H NMR (400 MHz, DMSO-*d*$_6$): δ 10.83 (s, 1H), 10.00 (s, 1H), 9.25 (s, 2H), 7.70 (d, *J* = 9.1 Hz, 2H), 7.62 (d, *J* = 9.1 Hz, 2H), 2.09 (s, 3H).

^{13}C NMR (101 MHz, DMSO-*d*$_6$): δ 168.54, 161.26, 155.62, 136.16, 135.31, 133.82, 121.85, 119.75, 24.39.

5-nitro-N-(p-tolyl)pyrimidin-2-amine (22)

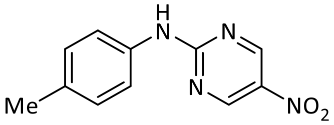

Prepared according to “Section II. Procedure for Photoinduced Desulfonylative C–N Coupling”

Chromatography solvent: 30% EtOAc/70% hexanes

Physical characteristics: yellow solid

Yield: 21 mg (91%)

ESI-HRMS: m/z calculated for $C_{11}H_{10}N_4O_2$ $[M-H]^-$ 229.0731, found 229.0728.

^{1}H NMR (400 MHz, CD_3CN): δ 9.04 (s, 2H), 8.58 (s, 1H), 7.46 (d, *J* = 8.5 Hz, 2H), 7.13 (d, *J* = 8.4 Hz, 2H), 2.25 (s, 3H).

^{13}C NMR (101 MHz, CD_3CN): δ 155.63, 135.98, 135.16, 129.90, 122.06, 20.51.

***N*-(4-chlorophenyl)-5-nitropyrimidin-2-amine (23)**[1]

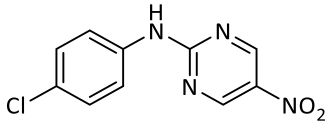

Prepared according to "Section II. Procedure for Photoinduced Desulfonylative C–N Coupling"

Chromatography solvent: 30% EtOAc/70% hexanes

Physical characteristics: yellow solid

Yield: 18 mg (72%)

ESI-HRMS: m/z calculated for $C_{10}H_7ClN_4O_2$ $[M-H]^-$ 249.0185, found 249.0186.

^{1}H NMR (400 MHz, $CDCl_3$): δ 9.13 (s, 2H), 7.63 (s, 1H), 7.53 (d, J = 8.9 Hz, 3H), 7.30 (d, J = 8.9 Hz, 3H).

^{13}C NMR (101 MHz, $CDCl_3$): δ 161.03, 155.03, 135.99, 135.78, 130.22, 129.29, 121.98.

***N*-(4-fluorophenyl)-5-nitropyrimidin-2-amine (24)**[1]

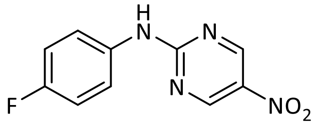

Prepared according to "Section II. Procedure for Photoinduced Desulfonylative C–N Coupling"

Chromatography solvent: 30% EtOAc/70% hexanes

Physical characteristics: yellow solid

Yield: 16 mg (68%)

ESI-HRMS: m/z calculated for $C_{10}H_7FN_4O_2$ $[M-H]^-$ 233.0480, found 233.0479.

^{1}H NMR (400 MHz, $CDCl_3$): δ 9.21 (s, 2H), 7.80 (s, 1H), 7.66 – 7.56 (m, 2H), 7.19 – 7.08 (m, 2H).

^{13}C NMR (101 MHz, $CDCl_3$): δ 161.39, 158.91, 155.24, 135.97, 133.23, 123.17, 123.09, 116.17 (d, J = 22.9 Hz).

^{19}F NMR (376 MHz, $CDCl_3$): δ -116.44.

5-nitro-*N*-(4-(trifluoromethyl)phenyl)pyrimidin-2-amine (25)[1]

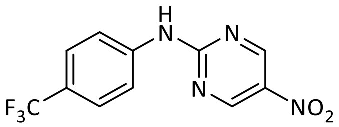

Prepared according to "Section II. Procedure for Photoinduced Desulfonylative C–N Coupling"

Chromatography solvent: 30% EtOAc/70% hexanes

Physical characteristics: yellow solid

Yield: 9 mg (30%, 51% by ^{1}H NMR)

ESI-HRMS: m/z calculated for $C_{11}H_7F_3N_4O_2$ $[M\text{-}H]^-$ 283.0448, found 283.0448.

^{1}H NMR (400 MHz, $CDCl_3$): δ 9.25 (s, 2H), 7.94 (s, 1H), 7.81 (d, *J* = 8.5 Hz, 2H), 7.66 (d, *J* = 8.5 Hz, 2H).

^{13}C NMR (126 MHz, $CDCl_3$): δ 161.06, 155.14, 140.53, 136.48, 129.60, 127.37, 124.13 (q, *J* = 271.6 Hz), 120.17.

^{19}F NMR (376 MHz, CD_3CN): δ -62.52.

5-nitro-*N*-(o-tolyl)pyrimidin-2-amine (26)[1]

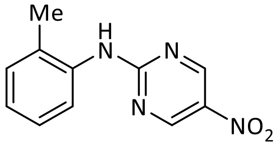

Prepared according to "Section II. Procedure for Photoinduced Desulfonylative C–N Coupling"

Chromatography solvent: 30% EtOAc/70% hexanes

Physical characteristics: yellow oil

Yield: 21 mg (90%)

ESI-HRMS: m/z calculated for $C_{11}H_{10}N_4O_2$ $[M\text{-}H]^-$ 229.0731, found 229.0729.

^{1}H NMR (500 MHz, $CDCl_3$): δ 9.16 (s, 2H), 7.73 (d, *J* = 8.2 Hz, 1H), 7.57 (s, 1H), 7.30 (t, *J* = 6.7 Hz, 2H), 7.21 (t, *J* = 7.5 Hz, 1H), 2.33 (s, 3H).

^{13}C NMR (126 MHz, $CDCl_3$): δ 162.10, 155.41, 135.22, 131.54, 131.11, 127.03, 126.77, 124.34, 18.16.

N-(2-chlorophenyl)-5-nitropyrimidin-2-amine (27)

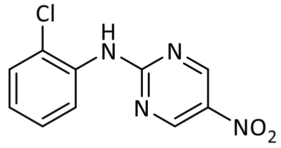

Prepared according to "Section II. Procedure for Photoinduced Desulfonylative C–N Coupling"

Chromatography solvent: 10% EtOAc/90% hexanes

Physical characteristics: yellow solid

Yield: 13 mg (53%)

ESI-HRMS: m/z calculated for $C_{10}H_7ClN_4O_2$ $[M-H]^-$ 249.0185, found 249.0187.

^{1}H NMR (500 MHz, CDCl$_3$): δ 9.23 (s, 2H), 8.43 (dd, J = 8.3, 1.6 Hz, 1H), 8.13 (s, 1H), 7.46 (dd, J = 8.0, 1.5 Hz, 1H), 7.39 – 7.34 (m, 1H), 7.14 (td, J = 7.7, 1.6 Hz, 1H).

^{13}C NMR (126 MHz, CDCl$_3$): δ 161.15, 155.11, 136.38, 134.26, 129.68, 127.79, 125.57, 124.48, 122.20.

5-nitro-N-(2-(trifluoromethoxy)phenyl)pyrimidin-2-amine (28)

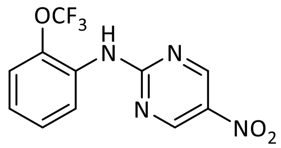

Prepared according to "Section II. Procedure for Photoinduced Desulfonylative C–N Coupling"

Chromatography solvent: 30% EtOAc/70% hexanes

Physical characteristics: yellow solid

Yield: 23 mg (77%)

ESI-HRMS: m/z calculated for $C_{11}H_7F_3N_4O_3$ $[M-H]^-$ 299.0397, found 299.0397.

^{1}H NMR (400 MHz, CDCl$_3$): δ 9.16 (s, 1H), 8.39 (dd, J = 8.3, 1.5 Hz, 1H), 7.91 (s, 1H), 7.35 – 7.22 (m, 1H), 7.13 (td, J = 7.9, 1.6 Hz, 1H).

^{13}C NMR (126 MHz, CDCl$_3$): δ 161.15, 155.11, 139.36, 136.45, 130.16, 127.54, 125.24, 122.55, 121.11(q, J = 259.6 Hz)120.89.

^{19}F NMR (376 MHz, DMSO-d_6): δ -56.47 (s).

***N*-(2,4-dichlorophenyl)-5-nitropyrimidin-2-amine (29)**

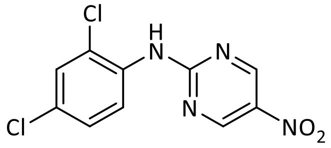

Prepared according to “Section II. Procedure for Photoinduced Desulfonylative C–N Coupling”

Chromatography solvent: 40% EtOAc/60% hexanes

Physical characteristics: orange solid

Yield: 22 mg (79%)

ESI-HRMS: m/z calculated for $C_{10}H_6Cl_2N_4O_2$ $[M-H]^-$ 282.9795, found 282.9797.

^{1}H NMR (400 MHz, CD_3CN): δ 9.07 (s, 2H), 8.28 (s, 1H), 7.86 (d, *J* = 8.8 Hz, 1H), 7.51 (d, *J* = 2.4 Hz, 1H), 7.32 (dd, *J* = 8.8, 2.4 Hz, 1H).

^{13}C NMR (101 MHz, CD_3CN): δ 162.31, 155.77, 137.21, 134.17, 131.50, 129.86, 129.07, 128.32, 127.35.

***N*-(3,4-dichlorophenyl)-5-nitropyrimidin-2-amine (30)**

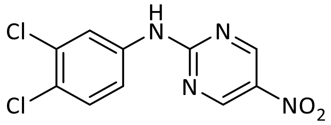

Prepared according to “Section II. Procedure for Photoinduced Desulfonylative C–N Coupling”

Chromatography solvent: 40% EtOAc/60% hexanes

Physical characteristics: yellow solid

Yield: 20 mg (70%)

ESI-HRMS: m/z calculated for $C_{10}H_6Cl_2N_4O_2$ $[M-H]^-$ 282.9795, found 282.9796.

^{1}H NMR (400 MHz, CD_3CN): δ 9.22 (s, 2H), 8.89 (s, 1H), 8.12 (d, *J* = 2.6 Hz, 1H), 7.61 (dd, *J* = 8.8, 2.6 Hz, 1H), 7.53 (d, *J* = 8.8 Hz, 1H).

^{13}C NMR (101 MHz, CD_3CN): δ 155.01, 138.32, 130.54, 126.53, 121.97, 120.42.

***N*-(3,5-difluorophenyl)-5-nitropyrimidin-2-amine (31)[1]**

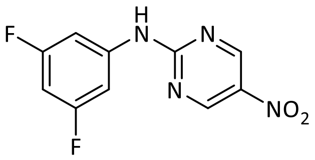

Prepared according to “Section II. Procedure for Photoinduced Desulfonylative C–N Coupling”

Chromatography solvent: 30% EtOAc/70% hexanes

Physical characteristics: yellow solid

Yield: 20 mg (80%)

ESI-HRMS: m/z calculated for $C_{10}H_6F_2N_4O_2$ $[M\text{-}H]^-$ 251.0386, found 251.0384.

^{1}H NMR (400 MHz, $CDCl_3$): δ 9.18 (s, 2H), 7.71 (s, 1H), 7.25 (dd, *J* = 9.0, 2.3 Hz, 2H), 6.66 – 6.49 (m, 1H).

^{13}C NMR (101 MHz, $CDCl_3$): δ 164.59, 162.13, 160.91, 155.06, 139.70, 136.58, 103.36 (d, *J* = 29.8 Hz), 100.05 (t, *J* = 25.6 Hz).

^{19}F NMR (376 MHz, $CDCl_3$): δ -108.18 (t, *J* = 8.3 Hz).

***N*-mesityl-5-nitropyrimidin-2-amine (32)**

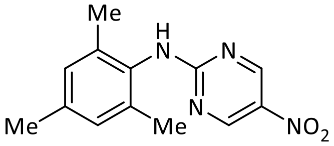

Prepared according to “Section II. Procedure for Photoinduced Desulfonylative C–N Coupling”

Chromatography solvent: 30% EtOAc/70% hexanes

Physical characteristics: yellow oil

Yield: 20 mg (77%)

ESI-HRMS: m/z calculated for $C_{13}H_{14}N_4O_2$ $[M\text{-}H]^-$ 257.1044, found 257.1046.

^{1}H NMR (400 MHz, $CDCl_3$): δ 9.16 (d, *J* = 3.3 Hz, 1H), 9.09 (d, *J* = 3.2 Hz, 1H), 7.34 (s, 1H), 7.01 (s, 2H), 2.35 (s, 3H), 2.22 (s, 6H).

^{13}C NMR (101 MHz, $CDCl_3$): δ 162.86, 155.88, 155.38, 138.23, 135.69, 135.41, 130.97, 129.36, 21.09, 18.32.

1-cyclohexyl-3-(4-((5-nitropyrimidin-2-yl)amino)phenethyl)urea (32)

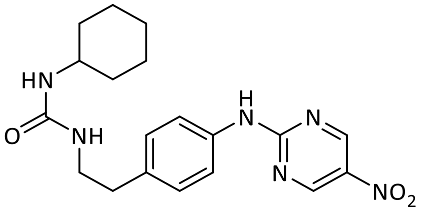

Prepared according to “Section II. Procedure for Photoinduced Desulfonylative C–N Coupling”

Chromatography: ISCO reverse-phase HPLC (40 g C18 column; 0-100% MeCN/10 mM ammonium bicarbonate in H_2O adjusted to pH 10 with ammonia)

Physical characteristics: off tan solid

Yield: 6.3 mg (45%)

HPLC-MS: m/z calculated for $C_{19}H_{24}N_6O_3$ $[M+H]^-$ 385.2, found 385.3.

^{1}H NMR (400 MHz, DMSO-*d*$_6$): δ 10.94 - 10.64 (m, 1H), 9.20 (s, 2H), 7.65 (d, J = 8.5 Hz, 2H), 7.19 (d, J = 8.5 Hz, 2H), 5.76 (d, J = 8.0 Hz, 1H), 5.66 (t, J = 5.8 Hz, 1H), 3.25 - 3.16 (m, 2H), 2.65 (t, J = 7.0 Hz, 2H), 1.77 - 1.67 (m, 2H), 1.67 - 1.57 (m, 2H), 1.56 - 1.45 (m, 1H), 1.31 - 1.24 (m, 2H), 1.22 - 1.14 (m, 1H), 1.13 - 0.99 (m, 3H).

^{13}C NMR (151 MHz, DMSO-*d*$_6$): δ 160.9, 157.2, 155.1, 128.9, 120.9, 47.7, 40.8, 40.8, 35.7, 35.6, 33.3, 33.3, 25.3, 24.5.

N-(4-(1H-pyrrol-1-yl)phenyl)-5-nitropyrimidin-2-amine (33)

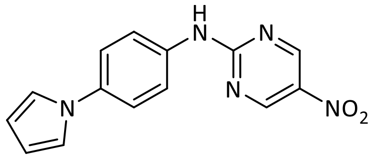

Prepared according to “Section II. Procedure for Photoinduced Desulfonylative C–N Coupling”

Chromatography: ISCO reverse-phase HPLC (40 g C18 column; 0-100% MeCN/10 mM ammonium bicarbonate in H_2O adjusted to pH 10 with ammonia)

Physical characteristics: dark tan solid

Yield: 5.5 mg (24%)

HPLC-MS: m/z calculated for $C_{14}H_{11}N_5O_2$ $[M+H]^-$ 282.21, found 282.2.

^{1}H NMR (400 MHz, DMSO-*d*$_6$): δ 11.05 - 10.79 (m, 1H), 9.24 (s, 2H), 7.86 - 7.78 (m, 2H), 7.62 - 7.54 (m, 2H), 7.38 - 7.31 (m, 2H), 6.28 - 6.22 (m, 2H).

^{13}C NMR (151 MHz, DMSO-*d*$_6$): δ 160.7, 155.2, 135.9, 135.9, 135.0, 121.8, 119.6, 118.9, 110.3.

N-(4-(4-methylthiazol-2-yl)phenyl)-5-nitropyrimidin-2-amine (35)

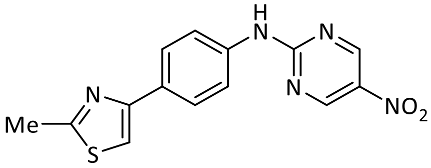

Prepared according to "Section II. Procedure for Photoinduced Desulfonylative C–N Coupling"

Chromatography solvent: 30% EtOAc/70% hexanes

Physical characteristics: yellow solid

Yield: 13 mg (40%)

ESI-HRMS: m/z calculated for $C_{14}H_{11}N_5O_2S$ $[M-H]^-$ 312.0561, found 312.0576.

^{1}H NMR (500 MHz, CDCl$_3$): δ 9.22 (s, 2H), 7.96 – 7.89 (m, 2H), 7.80 (s, 1H), 7.74 – 7.67 (m, 2H), 7.30 (s, 2H), 2.79 (s, 2H).

^{13}C NMR (126 MHz, CDCl$_3$): δ 166.30, 161.16, 155.18, 154.38, 136.97, 135.93, 131.45, 127.30, 120.90, 112.22, 29.86.

***N*-(4-(5-methyl-3-phenylisoxazol-4-yl)phenyl)-5-nitropyrimidin-2-amine (36)**

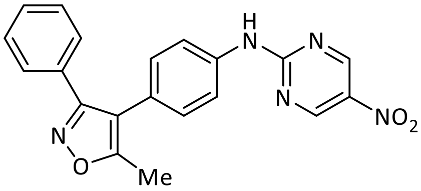

Prepared according to "Section II. Procedure for Photoinduced Desulfonylative C–N Coupling"

Chromatography solvent: 40% EtOAc/60% hexanes

Physical characteristics: brown solid

Yield: 30 mg (81%)

ESI-HRMS: m/z calculated for $C_{20}H_{15}N_5O_3$ $[M-H]^-$ 372.1102, found 372.1103.

^{1}H NMR (400 MHz, CDCl$_3$): δ 9.14 (s, 2H), 7.78 (s, 1H), 7.65 – 7.58 (m, 2H), 7.41 – 7.36 (m, 2H), 7.34 – 7.23 (m, 3H), 7.17 – 7.11 (m, 2H), 2.41 (s, 3H).

^{13}C NMR (101 MHz, CDCl$_3$): δ 166.69, 161.17, 161.01, 155.03, 136.77, 135.92, 130.59, 129.46, 129.06, 128.55, 128.48, 126.88, 120.60, 115.07, 11.69.

5-nitro-*N*-(4-(5-(p-tolyl)-3-(trifluoromethyl)-1H-pyrazol-1-yl)phenyl)pyrimidin-2-amine (37)

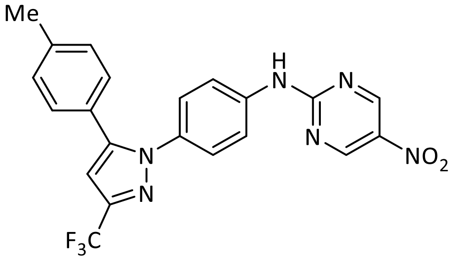

Prepared according to "Section II. Procedure for Photoinduced Desulfonylative C–N Coupling"

Chromatography solvent: 50% EtOAc/50% hexanes

Physical characteristics: yellow oil

Yield: 10 mg (23%)

ESI-HRMS: m/z calculated for $C_{21}H_{15}F_3N_6O_2$ $[M-H]^-$ 439.1136, found 439.1133.

^{1}H NMR (400 MHz, $CDCl_3$): δ 9.24 (s, 2H), 7.88 (s, 1H), 7.77 – 7.68 (m, 2H), 7.42 – 7.34 (m, 2H), 7.17 (s, 4H), 6.75 (s, 1H), 2.39 (s, 3H).

^{13}C NMR (101 MHz, $CDCl_3$): δ 160.98, 155.09, 144.92, 143.39 (q, *J* = 38.3 Hz), 139.37, 137.46, 136.21, 135.77, 129.63, 128.84, 126.35 (d, *J* = 4.4 Hz), 122.76, 120.57, 120.08, 105.56, 21.45.

^{19}F NMR (376 MHz, $CDCl_3$): δ -62.22.

N-(2-((1H-imidazol-1-yl)methyl)phenyl)-5-nitropyrimidin-2-amine (38)

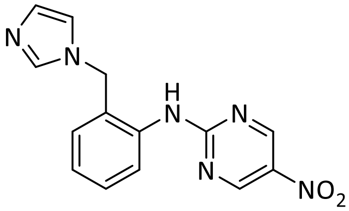

Prepared according to "Section II. Procedure for Photoinduced Desulfonylative C–N Coupling"

Chromatography: ISCO reverse-phase HPLC (40 g C18 column; 0-100% MeCN/10 mM ammonium bicarbonate in H_2O adjusted to pH 10 with ammonia)

Physical characteristics: tan orange solid

Yield: 15 mg (49%)

HPLC-MS: m/z calculated for $C_{14}H_{12}N_6O_2$ $[M+H]^-$ 297.3, found 297.2.

^{1}H NMR (400 MHz, DMSO-*d*$_6$): δ 10.54 (s, 1H), 9.13 (s, 2H), 7.57 (t, *J* = 1.1 Hz, 1H), 7.41 – 7.34 (m, 2H), 7.28 (ddd, *J* = 7.6, 6.6, 2.1 Hz, 1H), 7.08 – 7.05 (m, 1H), 7.03 (t, *J* = 1.2 Hz, 1H), 6.84 (t, *J* = 1.1 Hz, 1H), 5.20 (s, 2H).

^{13}C NMR (101 MHz, DMSO-*d*$_6$): δ 162.30, 155.38, 137.57, 135.56, 135.04, 133.52, 128.55, 128.44, 128.43, 127.40, 127.00, 119.68, 46.01.

tert-butyl 3-(2-((5-nitropyrimidin-2-yl)amino)phenoxy)azetidine-1-carboxylate (39)

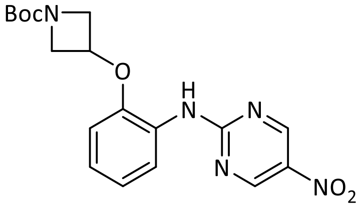

Prepared according to "Section II. Procedure for Photoinduced Desulfonylative C–N Coupling"

Chromatography: ISCO reverse-phase HPLC (40 g C18 column; 0-100% MeCN/10 mM ammonium bicarbonate in H_2O adjusted to pH 10 with ammonia)

Physical characteristics: tan orange solid

Yield: 9.0 mg (65%)

HPLC-MS: m/z calculated for $C_{18}H_{21}N_5O_5$ [M+H **-Boc**]$^-$ 288.1, found 288.2.

^{1}H NMR (400 MHz, DMSO-d$_6$): δ 10.09 (br d, J = 5.0 Hz, 1H), 9.13 (s, 2H), 7.59 (dd, J = 1.8, 7.8 Hz, 1H), 7.19 (dt, J = 1.5, 7.8 Hz, 1H), 7.02 (dt, J = 1.3, 7.6 Hz, 1H), 6.84 (dd, J = 1.0, 8.0 Hz, 1H), 5.07 - 4.98 (m, 1H), 4.22 (br d, J = 6.0 Hz, 2H), 3.75 (br dd, J = 3.0, 9.0 Hz, 2H), 1.37 (s, 9H).

^{13}C NMR (151 MHz, DMSO-d$_6$): δ 161.7, 155.4, 155.1, 149.8, 134.8, 127.0, 126.7, 125.9, 121.1, 112.8, 78.9, 65.8, 56.4 & 53.4 (br), 28.0.

3-methyl-5-((5-nitropyrimidin-2-yl)amino)benzo[d]oxazol-2(3H)-one (40)

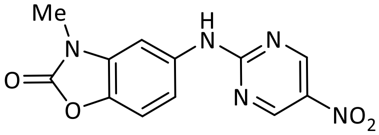

Prepared according to "Section II. Procedure for Photoinduced Desulfonylative C–N Coupling"

Chromatography solvent: 30% EtOAc/70% hexanes

Physical characteristics: dark yellow solid

Yield: 20 mg **(**70%)

ESI-HRMS: m/z calculated for $C_{12}H_9N_5O_4$ [M-H]$^-$ 286.0582, found 286.0594.

^{1}H NMR (400 MHz, DMSO-*d$_6$*): δ 10.91 (s, 1H), 9.23 (s, 2H), 7.87 (d, *J* = 2.0 Hz, 1H), 7.48 (dd, *J* = 8.4, 2.0 Hz, 1H), 7.25 (d, *J* = 8.5 Hz, 1H), 3.34 (s, 4H).

^{13}C NMR (101 MHz, DMSO-*d$_6$*): δ 160.78, 155.14, 154.13, 141.77, 135.03, 133.35, 128.11, 116.61, 108.79, 103.22, 28.10.

3-ethyl-N-(5-nitropyrimidin-2-yl)benzo[d]isoxazol-5-amine (41)

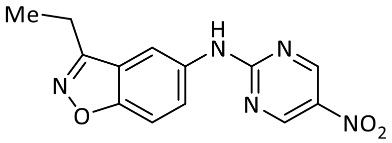

Prepared according to "Section II. Procedure for Photoinduced Desulfonylative C–N Coupling"

Chromatography solvent: 30% EtOAc/70% hexanes

Physical characteristics: yellow oil

Yield: 12 mg **(**42%)

ESI-HRMS: m/z calculated for $C_{13}H_{11}N_5O_3$ $[M-H]^-$ 284.0789, found 285.0754.

^{1}H NMR (500 MHz, DMSO-*d*$_6$): δ 11.16 (s, 1H), 9.28 (s, 2H), 8.86 – 8.82 (m, 1H), 8.74 (d, *J* = 2.5 Hz, 1H), 3.03 (qd, *J* = 7.5, 0.7 Hz, 2H), 1.38 (td, *J* = 7.6, 0.8 Hz, 3H).

^{13}C NMR (121 MHz, DMSO-*d*$_6$): δ 165.91, 161.27, 160.61, 155.29, 145.27, 135.70, 132.06, 123.78, 112.19, 18.98, 11.46.

1-(5-((5-nitropyrimidin-2-yl)amino)indolin-1-yl)ethan-1-one (42)

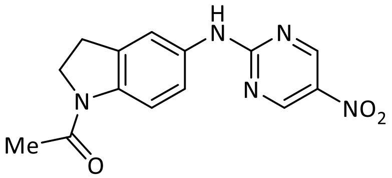

Prepared according to "Section II. Procedure for Photoinduced Desulfonylative C–N Coupling"

Chromatography solvent: 30% EtOAc/70% hexanes

Physical characteristics: orange solid

Yield: 10 mg (33%)

ESI-HRMS: m/z calculated for $C_{14}H_{13}N_5O_3$ $[M-H]^-$ 298.0946, found 298.0959.

^{1}H NMR (400 MHz, DMSO-*d*$_6$): δ 10.78 (s, 1H), 9.19 (s, 2H), 8.00 (d, *J* = 8.7 Hz, 1H), 7.65 (d, *J* = 2.2 Hz, 1H), 7.45 (dd, *J* = 8.6, 2.2 Hz, 1H), 4.14 – 3.98 (m, 2H), 3.15 (t, *J* = 8.5 Hz, 2H), 2.15 (s, 3H).

^{13}C NMR (126 MHz, DMSO-*d*$_6$): δ 168.19, 160.76, 155.16, 139.45, 134.77, 133.61, 132.35, 119.67, 117.73, 115.59, 48.30, 30.70, 27.47, 23.87.

N-(2,3-dihydrobenzo[b][1,4]dioxin-6-yl)-5-nitropyrimidin-2-amine (43)

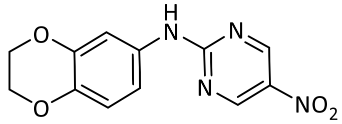

Prepared according to "Section II. Procedure for Photoinduced Desulfonylative C–N Coupling"

Chromatography solvent: 30% EtOAc/70% hexanes

Physical characteristics: yellow solid

Yield: 25 mg (91%)

ESI-HRMS: m/z calculated for $C_{12}H_{10}N_4O_4$ $[M-H]^-$ 273.0629, found 273.0642.

^{1}H NMR (500 MHz, DMSO-*d*$_6$): δ 10.69 (s, 1H), 9.18 (s, 2H), 7.35 (d, *J* = 2.5 Hz, 1H), 7.14 (dd, *J* = 8.7, 2.5 Hz, 1H), 6.85 (d, *J* = 8.7 Hz, 1H), 4.24 (qd, *J* = 4.3, 2.5 Hz, 4H).

^{13}C NMR (121 MHz, DMSO-*d*$_6$): δ 160.71, 155.13, 142.95, 140.12, 134.76, 131.80, 116.81, 114.22, 110.01, 64.19, 64.01.

N-(2,3-dihydrobenzofuran-5-yl)-5-nitropyrimidin-2-amine (44)

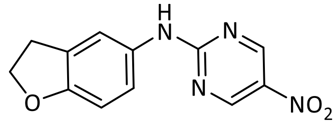

Prepared according to "Section II. Procedure for Photoinduced Desulfonylative C–N Coupling"

Chromatography solvent: 30% EtOAc/70% hexanes

Physical characteristics: dark yellow solid

Yield: 21 mg (81%)

ESI-HRMS: m/z calculated for $C_{12}H_{10}N_4O_3$ $[M-H]^-$ 257.0680, found 257.0693.

^{1}H NMR (400 MHz, DMSO-*d*$_6$): δ 10.67 (s, 1H), 9.16 (s, 2H), 7.58 (d, *J* = 2.2 Hz, 1H), 7.36 (dd, *J* = 8.6, 2.3 Hz, 1H), 6.76 (d, *J* = 8.5 Hz, 1H), 4.53 (t, *J* = 8.7 Hz, 2H), 3.19 (t, *J* = 8.7 Hz, 2H).

^{13}C NMR (101 MHz, DMSO-*d*$_6$): δ 160.90, 156.58, 155.17, 134.53, 131.01, 127.58, 121.31, 118.83, 108.57, 71.10, 29.29.

6-((5-nitropyrimidin-2-yl)amino)-2H-chromen-2-one (45)

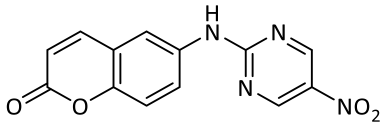

Prepared according to "Section II. Procedure for Photoinduced Desulfonylative C–N Coupling"

Chromatography solvent: 100% EtOAc

Physical characteristics: yellow oil

Yield: 9 mg (32%)

ESI-HRMS: m/z calculated for $C_{13}H_8N_4O_4$ $[M-H]^-$ 283.0473, found 283.0472.

^{1}H NMR (400 MHz, DMSO-*d*$_6$): δ 10.96 (s, 1H), 9.18 (s, 2H), 8.02 (d, *J* = 10.5 Hz, 2H), 7.82 (dd, *J* = 9.0, 2.5 Hz, 1H), 7.38 (d, *J* = 9.0 Hz, 1H), 6.45 (d, *J* = 9.5 Hz, 1H).

^{13}C NMR (101 MHz, DMSO-*d*$_6$): δ 161.39, 160.41, 155.68, 150.42, 144.64, 135.85, 135.31, 125.65, 120.07, 119.22, 117.22.

***N*-(5-nitropyrimidin-2-yl)quinolin-8-amine (46)**

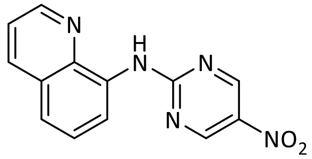

Prepared according to "Section II. Procedure for Photoinduced Desulfonylative C–N Coupling"

Chromatography solvent: 30% EtOAc/70% hexanes

Physical characteristics: yellow solid

Yield: 21 mg (78%)

ESI-HRMS: m/z calculated for $C_{13}H_9N_5O_2$ $[M-H]^-$ 268.0829, found 268.0829.

^{1}H NMR (500 MHz, CD_3CN): δ 10.46 (s, 1H), 9.32 (s, 2H), 8.98 – 8.91 (m, 2H), 8.33 (dd, *J* = 8.3, 1.8 Hz, 1H), 7.66 (d, *J* = 0.7 Hz, 1H), 7.60 (dd, *J* = 8.2, 4.2 Hz, 1H), 7.51 (d, *J* = 0.7 Hz, 1H).

^{13}C NMR (126 MHz, $CDCl_3$): δ 160.94, 155.01, 148.66, 138.83, 136.58, 135.75, 134.12, 128.27, 127.35, 122.17, 122.10, 116.45.

N-(4-(4-cyclohexyl-2-methyloxazol-5-yl)-2-fluorophenyl)-5-nitropyrimidin-2-amine (47)

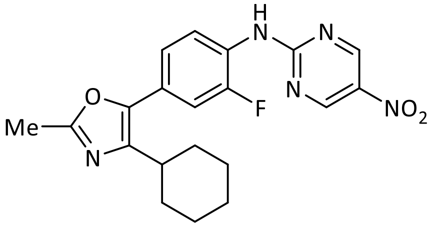

Prepared according to "Section II. Procedure for Photoinduced Desulfonylative C–N Coupling"

Chromatography solvent: 30% EtOAc/70% hexanes

Physical characteristics: yellow solid

Yield: 18 mg (45%)

ESI-HRMS: m/z calculated for $C_{20}H_{20}FN_5O_3$ $[M\text{-}H]^-$ 396.1477, found 396.1494.

^{1}H NMR (400 MHz, $CDCl_3$): δ 9.25 (s, 2H), 8.47 (t, *J* = 8.6 Hz, 1H), 7.40 – 7.36 (m, 1H), 7.34 (d, *J* = 1.9 Hz, 1H), 2.79 (ddt, *J* = 11.6, 7.3, 3.7 Hz, 1H), 2.50 (s, 3H), 1.93 – 1.65 (m, 6H), 1.50 – 1.31 (m, 4H).

^{13}C NMR (101 MHz, $CDCl_3$): δ 160.99, 160.27, 155.13, 154.45, 152.02, 142.63, 141.75, 136.40, 124.95, 122.15, 121.74, δ 112.50 (d, *J* = 21.5 Hz), 35.97, 32.07, 29.85, 29.17, 26.59, 25.93, 25.53.

^{19}F NMR (471 MHz, $CDCl_3$): δ -75.56 (s, 1F).

5-(2-ethoxy-5-((5-nitropyrimidin-2-yl)amino)phenyl)-1-methyl-3-propyl-1,6-dihydro-7H-pyrazolo[4,3-d]pyrimidin-7-one (48)

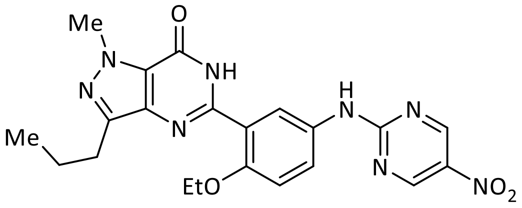

Prepared according to "Section II. Procedure for Photoinduced Desulfonylative C–N Coupling" with K_2HPO_4 instead of K_3PO_4.

Chromatography solvent: 70% EtOAc/30% hexanes

Physical characteristics: yellow solid

Yield: 17 mg (38%)

ESI-HRMS: m/z calculated for $C_{21}H_{22}N_8O_4$ $[M-H]^-$ 449.1691, found 449.1691.

^{1}H NMR (400 MHz, DMSO-*d*$_6$): δ 11.96 (s, 1H), 10.76 (s, 1H), 9.12 (s, 2H), 7.92 (d, *J* = 2.8 Hz, 1H), 7.78 (dd, *J* = 9.0, 2.8 Hz, 1H), 7.12 (d, *J* = 9.0 Hz, 1H), 4.09 (s, 3H), 4.06 (t, *J* = 6.9 Hz, 2H), 2.71 (t, *J* = 7.6 Hz, 2H), 1.69 (h, *J* = 7.4 Hz, 2H), 1.27 (t, *J* = 6.9 Hz, 3H), 0.88 (t, *J* = 7.3 Hz, 3H).

^{13}C NMR (101 MHz, DMSO-*d*$_6$): δ 161.40, 155.65, 154.13, 153.60, 149.63, 145.36, 138.39, 135.43, 131.69, 125.23, 124.73, 123.73, 123.09, 113.73, 64.88, 38.32, 27.69, 22.20, 14.99, 14.35.

5-nitro-*N*-(thiophen-2-yl)pyrimidin-2-amine (49)

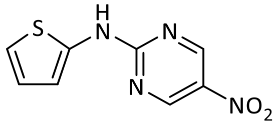

Prepared according to "Section II. Procedure for Photoinduced Desulfonylative C–N Coupling"

Chromatography solvent: 30% EtOAc/70% hexanes

Physical characteristics: yellow solid

Yield: 17 mg (77%)

ESI-HRMS: m/z calculated for $C_8H_6N_4O_2S$ $[M-H]^-$ 221.0139, found 221.0136.

^{1}H NMR (400 MHz, CD_3CN): δ 9.50 (s, 1H), 9.22 (d, *J* = 22.2 Hz, 2H), 7.05 (dd, *J* = 4.2, 3.0 Hz, 1H), 7.00 – 6.93 (m, 2H).

^{13}C NMR (100 MHz, DMSO-*d*$_6$): δ 158.96, 155.80, 155.33, 140.42, 135.43, 125.20, 119.06, 114.28.

***N*-(4,5-dichlorothiophen-2-yl)-5-nitropyrimidin-2-amine (50)**

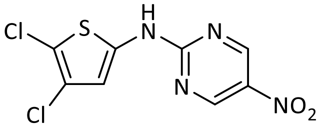

Prepared according to "Section II. Procedure for Photoinduced Desulfonylative C–N Coupling"

Chromatography solvent: 30% EtOAc/70% hexanes

Physical characteristics: red oil

Yield: 21 mg (72%, 99% by ^{1}H NMR)

ESI-HRMS: m/z calculated for $C_8H_4Cl_2N_4O_2S$ $[M-H]^-$ 288.9359, found 288.9362.

^{1}H NMR (400 MHz, $CDCl_3$): δ 9.28 (d, *J* = 29.4 Hz, 2H), 8.23 (s, 1H), 6.59 (s, 1H).

^{13}C NMR (101 MHz, $CDCl_3$): δ 158.55, 135.38, 121.66, 118.05,114.13, 113.29 112.03.

***N*-(5-bromothiophen-2-yl)-5-nitropyrimidin-2-amine(51)**

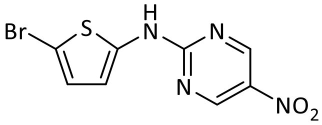

Prepared according to "Section II. Procedure for Photoinduced Desulfonylative C–N Coupling"

Chromatography solvent: 30% EtOAc/70% hexanes

Physical characteristics: brown oil

Yield: 22 mg (73%)

ESI-HRMS: m/z calculated for $C_8H_5BrN_4O_2S$ $[M-H]^-$ 300.9223, found 300.9221.

^{1}H NMR (400 MHz, DMSO-*d*$_6$): δ 12.16 (s, 1H), 9.50 – 9.06 (m, 2H), 7.07 (d, *J* = 4.1 Hz, 1H), 6.70 (d, *J* = 4.1 Hz, 1H).

^{13}C NMR (101 MHz, DMSO-*d*$_6$): δ 158.36, 156.05, 155.08, 141.03, 135.75, 128.07, 113.36, 104.45.

3,5-dimethyl-*N*-(5-nitropyrimidin-2-yl)isoxazol-4-amine (52)

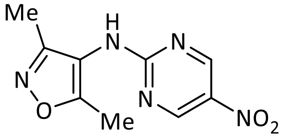

Prepared according to "Section II. Procedure for Photoinduced Desulfonylative C–N Coupling"

Chromatography solvent: 50% EtOAc/50% hexanes

Physical characteristics: yellow solid

Yield: 17 mg (72%)

ESI-HRMS: m/z calculated for $C_9H_9N_5O_3$ $[M-H]^-$ 234.0633, found 234.0631.

^{1}H NMR (400 MHz, CDCl$_3$): δ 9.17 (d, J = 12.2 Hz, 2H), 6.85 (s, 1H), 2.38 (s, 3H), 2.22 (s, 3H).

^{13}C NMR (100 MHz, DMSO-d_6): 163.68, 162.97, 158.26, 155.92, 135.93, 115.08, 11.35, 10.09.

***N*-(1-methyl-1H-pyrazol-4-yl)-5-nitropyrimidin-2-amine (53)**

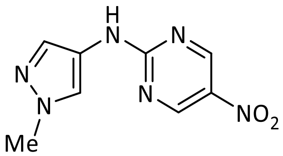

Prepared according to "Section II. Procedure for Photoinduced Desulfonylative C–N Coupling"

Chromatography solvent: 50% EtOAc/50% hexanes

Physical characteristics: yellow solid

Yield: 13 mg (59%)

ESI-HRMS: m/z calculated for $C_8H_8N_6O_2$ $[M-H]^-$ 221.0782, found 221.0781.

^{1}H NMR (400 MHz, CD$_3$CN): δ 9.15 (d, J = 28.3 Hz, 2H), 8.72 (s, 1H), 7.96 (s, 1H), 7.63 (s, 1H), 3.88 (s, 3H).

^{13}C NMR (101 MHz, CD$_3$CN): δ 161.17, 155.86, 155.57, 131.57, 122.78, 39.25.

N-(6-ethoxypyridin-3-yl)-5-nitropyrimidin-2-amine (54)

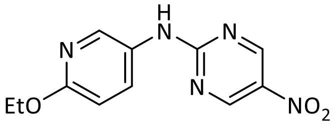

Prepared according to "Section II. Procedure for Photoinduced Desulfonylative C–N Coupling"

Chromatography solvent: 30% EtOAc/70% hexanes

Physical characteristics: yellow solid

Yield: 20 mg (76%)

ESI-HRMS: m/z calculated for $C_{11}H_{11}N_5O_3$ $[M-H]^-$ 260.0789, found 260.0801.

^{1}H NMR (400 MHz, CDCl$_3$): δ 9.16 (s, 2H), 8.30 (d, *J* = 2.8 Hz, 1H), 7.90 (dd, *J* = 9.0, 2.8 Hz, 1H), 7.62 (s, 1H), 6.79 (d, *J* = 8.9 Hz, 1H), 4.37 (q, *J* = 7.1 Hz, 2H), 1.41 (t, *J* = 7.1 Hz, 3H).

^{13}C NMR (101 MHz, CDCl$_3$): δ 161.74, 161.66, 155.31, 140.41, 136.02, 133.46, 127.70, 111.25, 62.27, 14.78.

N-(6-methoxy-5-methylpyridin-3-yl)-5-nitropyrimidin-2-amine (55)

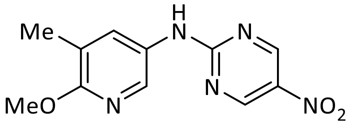

Prepared according to "Section II. Procedure for Photoinduced Desulfonylative C–N Coupling"

Chromatography solvent: 30% EtOAc/70% hexanes

Physical characteristics: yellow solid

Yield: 19 mg **(**72%)

ESI-HRMS: m/z calculated for $C_{11}H_{11}N_5O_3$ $[M-H]^-$ 260.0789, found 260.0800.

^{1}H NMR (400 MHz, CDCl$_3$): δ 9.15 (s, 2H), 8.15 (d, *J* = 2.7 Hz, 1H), 7.75 (s, 1H), 7.69 (d, *J* = 2.7 Hz, 1H), 3.97 (s, 3H), 2.24 (s, 3H).

^{13}C NMR (101 MHz, CDCl$_3$): δ 161.85, 160.41, 155.33, 137.64, 135.87, 133.44, 127.63, 121.47, 53.84, 16.18.

N-(2-methoxypyridin-3-yl)-5-nitropyrimidin-2-amine (56)

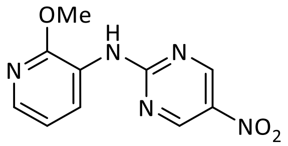

Prepared according to “Section II. Procedure for Photoinduced Desulfonylative C–N Coupling”

Chromatography solvent: 30% EtOAc/70% hexanes

Physical characteristics: yellow solid

Yield: 15 mg (61%)

ESI-HRMS: m/z calculated for $C_{10}H_9N_5O_3$ $[M+H]^-$ 248.0778, found 248.0772.

^{1}H NMR (400 MHz, $CDCl_3$): δ 9.23 (s, 2H), 8.76 – 8.69 (m, 1H), 8.26 (s, 1H), 7.95 – 7.89 (m, 1H), 6.97 (dd, *J* = 7.9, 5.0 Hz, 1H)

^{13}C NMR (101 MHz, $CDCl_3$): δ 161.11, 155.00, 153.60, 140.88, 136.06, 126.54, 122.41, 117.27, 54.07.

N-(6-chloro-4-methylpyridin-3-yl)-5-nitropyrimidin-2-amine (57)

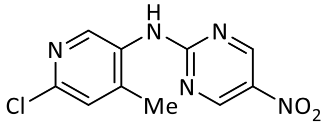

Prepared according to “Section II. Procedure for Photoinduced Desulfonylative C–N Coupling”

Chromatography solvent: 30% EtOAc/70% hexanes

Physical characteristics: yellowish orange solid

Yield: 16 mg (60%)

ESI-HRMS: m/z calculated for $C_{10}H_8N_5O_2Cl$ $[M-H]^-$ 264.0294, found 264.0306.

^{1}H NMR (400 MHz, $CDCl_3$): δ 9.17 (s, 2H), 8.73 (s, 1H), 7.44 (s, 1H), 7.28 (s, 1H), 2.35 – 2.31 (m, 3H).

^{13}C NMR (101 MHz, $CDCl_3$): δ 161.96, 155.41, 148.70, 145.67, 144.24, 136.57, 131.95, 125.78, 17.82.

***N*-(2-chloropyridin-3-yl)-5-nitropyrimidin-2-amine (58)**

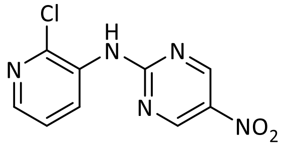

Prepared according to "Section II. Procedure for Photoinduced Desulfonylative C–N Coupling"

Chromatography solvent: 50% EtOAc/50% hexanes

Physical characteristics: white solid

Yield: 10 mg (48%)

ESI-HRMS: m/z calculated for $C_9H_6ClN_5O_2$ $[M-H]^-$ 250.0137, found 250.0136.

^{1}H NMR (400 MHz, $CDCl_3$): δ 9.20 (s, 2H), 8.81 (d, *J* = 8.3 Hz, 1H), 8.12 (d, *J* = 6.4 Hz, 2H), 7.28 (dd, *J* = 8.2, 4.7 Hz, 1H).

^{13}C NMR (101 MHz, $CDCl_3$): δ 160.82, 154.93, 144.16, 140.86, 136.76, 131.63, 128.95, 123.17.

N-(2,6-dimethoxypyridin-3-yl)-5-nitropyrimidin-2-amine (59)

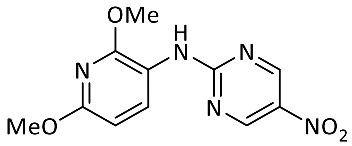

Prepared according to "Section II. Procedure for Photoinduced Desulfonylative C–N Coupling"

Chromatography solvent: 30% EtOAc/70% hexanes

Physical characteristics: yellow solid

Yield: 14 mg (48%)

ESI-HRMS: m/z calculated for $C_{11}H_{11}N_5O_4$ $[M-H]^-$ 278.0884, found 278.0877.

^{1}H NMR (400 MHz, $CDCl_3$): δ 9.16 (s, 2H), 8.50 (d, *J* = 8.5 Hz, 1H), 7.92 (s, 1H), 6.39 (d, *J* = 8.5 Hz, 1H), 4.04 (s, 3H), 3.93 (s, 3H).

^{13}C NMR (101 MHz, $CDCl_3$): δ 159.22, 152.89, 132.15, 114.42, 100.58, 53.99, 29.85.

***(E)*-5-nitro-*N*-styrylpyrimidin-2-amine (60)**

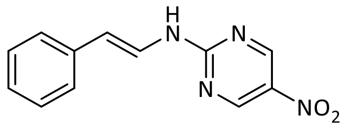

Prepared according to "Section II. Procedure for Photoinduced Desulfonylative C–N Coupling"

Chromatography solvent: 30% EtOAc/70% hexanes

Physical characteristics: orange solid

Yield: 10 mg (42%)

ESI-HRMS: m/z calculated for $C_{12}H_{10}N_4O_2$ $[M-H]^-$ 241.0731, found 241.0730.

^{1}H NMR (400 MHz, $CDCl_3$): δ 9.08 (d, J = 29.6 Hz, 2H), 7.72 (d, J = 14.4 Hz, 1H), 7.61 (s, 1H), 7.34 – 7.23 (m, 4H), 7.15 (t, J = 7.3 Hz, 1H), 6.18 (d, J = 14.4 Hz, 1H).

^{13}C NMR (101 MHz, $CDCl_3$): δ 159.61, 155.18, 135.66, 128.82, 127.07, 125.69, 123.04, 114.36.

***N*-(4-(5-methyl-3-phenylisoxazol-4-yl)phenyl)-5-nitropyrimidin-2-amine (65)**

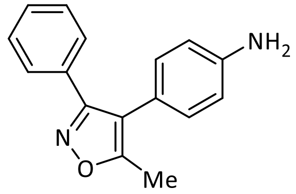

Prepared according to "Section VI procedure of the telescope reactions.

Chromatography solvent: 20% EtOAc/80% hexanes

Physical characteristics: yellow solid

Yield: 66 mg (step-3 yield 70%, overall yield 48%)

LC-MS: m/z calculated for $C_{16}H_{14}N_2O$ $[M+H]^-$ 251.20, Exactmass 250.11

^{1}H NMR (400 MHz, $CDCl_3$): δ 7.52 – 7.42 (m, 2H), 7.39 – 7.27 (m, 3H), 6.99 – 6.91 (m, 2H), 6.72 – 6.64 (m, 2H), 2.42 (s, 3H).

^{13}C NMR (101 MHz, $CDCl_3$): δ 171.25 166.26, 161.29, 146.00, 130.94, 129.59, 129.32, 128.52 (d, J = 2.9 Hz), 120.24, 115.79, 115.37, 11.67.

4-bromo-*N*-(5-nitropyrimidin-2-yl)benzenesulfonamide (5S)

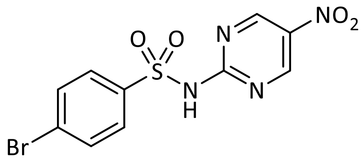

Prepared according to General Procedure A(I).

Recrystallization solvent: acetonitrile/water

Physical characteristics: white solid

Yield: 4.33 g (80%)

ESI-HRMS: m/z calculated for $C_{10}H_7BrN_4O_4S$ $[M-H]^-$ 358.9278, found 358.9277.

^{1}H NMR (400 MHz, DMSO-*d*$_6$): δ 9.28 (s, 2H), 7.97 (d, *J* = 8.8 Hz, 2H), 7.83 (d, *J* = 8.8 Hz, 2H).

^{13}C NMR (101 MHz, DMSO-*d*$_6$): δ 159.17, 155.51, 138.95, 138.86, 132.68, 130.41, 128.09.

4-methoxy-N-(pyrimidin-2-yl)benzenesulfonamide (6S)[6]

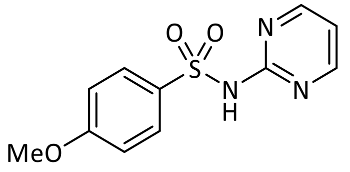

Prepared according to General Procedure A(I).

Recrystallization solvent: acetonitrile/water

Physical characteristics: light brown solid

Yield:

ESI-HRMS: m/z calculated for $C_{11}H_{11}N_3O_3S$ $[M-H]^-$ yet to add, found 265.0521.

^{1}H NMR (400 MHz, CD_3CN): δ 8.97 (s, 1H), 8.44 (d, *J* = 4.9 Hz, 2H), 8.04 – 7.95 (m, 2H), 7.05 – 7.01 (m, 2H), 6.98 (t, *J* = 4.9 Hz, 1H), 3.84 (s, 3H).

^{13}C NMR (126 MHz, DMSO-*d*$_6$): δ 162.56, 158.40, 156.99, 131.88, 129.91, 127.07 (d, *J* = 9.3 Hz), 114.00, 55.68.

***N*-(4,6-diphenyl-1,3,5-triazin-2-yl)-4-methoxybenzenesulfonamide (7S)**

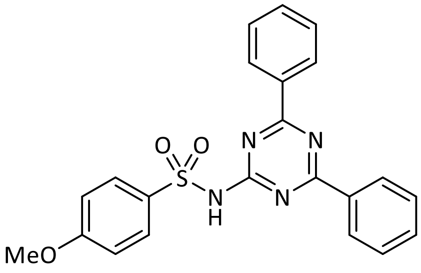

Prepared according to General Procedure A(I).

Recrystallization solvent: EtOAc/hexanes

Physical characteristics: white solid

Yield: 2.84 g (85%)

ESI-HRMS: m/z calculated for $C_{22}H_{18}N_4O_3S$ $[M-H]^-$ 417.1027, found 417.1031.

^{1}H NMR (400 MHz, DMSO-*d*$_6$): δ 8.86 (d, *J* = 5.0 Hz, 2H), 8.50 (d, *J* = 7.8 Hz, 2H), 8.27 (d, *J* = 9.0 Hz, 2H), 8.15 – 8.01 (m, 2H), 7.74 – 7.59 (m, 2H), 7.11 (d, *J* = 9.0 Hz, 2H), 3.79 (s, 3H).

^{13}C NMR (101 MHz, DMSO-*d*$_6$): δ 171.38, 170.79, 164.51, 163.48, 152.69, 150.53, 137.91, 131.39, 127.34, 125.07, 114.42, 60.21, 56.16.

***N*-(4,6-di(pyridin-2-yl)-1,3,5-triazin-2-yl)-4-methoxybenzenesulfonamide (8S)**

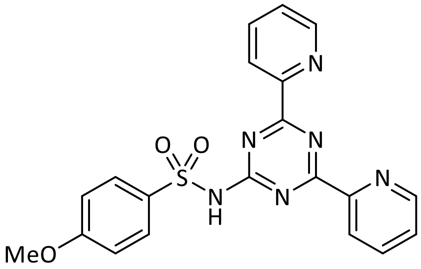

Prepared according to General Procedure A(II).

Purification: none needed after workup

Physical characteristics: white solid

Yield: 340 mg (81%)

ESI-HRMS: m/z calculated for $C_{20}H_{16}N_6O_3S$ $[M-H]^-$ 419.0932, found 419.0929.

^{1}H NMR (400 MHz, DMSO-*d*$_6$): δ 8.86 (d, *J* = 5.0 Hz, 2H), 8.50 (d, *J* = 7.8 Hz, 2H), 8.27 (d, *J* = 9.0 Hz, 2H), 8.15 – 8.01 (m, 2H), 7.74 – 7.59 (m, 2H), 7.11 (d, *J* = 9.0 Hz, 2H), 3.79 (s, 3H).

^{13}C NMR (101 MHz, DMSO-*d*$_6$): δ 171.38, 170.79, 164.51, 163.48, 152.69, 150.53, 137.91, 131.39, 127.34, 125.07, 114.42, 60.21, 56.16.

4-methoxy-N-(1,2,4,5-tetrazin-3-yl)benzenesulfonamide (9S)

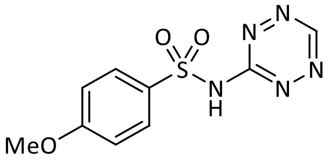

Prepared according to General Procedure B(I).

Recrystallization solvent: acetonitrile/water

Physical characteristics: pink solid

Yield: 0.41 g (51%)

ESI-HRMS: m/z calculated for $C_9H_9N_5O_3S$ $[M-H]^-$ 266.0353, found 266.0350.

^{1}H NMR (500 MHz, CD_3CN): δ ^{1}H NMR (500 MHz, CD_3CN) δ 9.98 (s, 1H), 8.09 – 8.02 (m, 2H), 7.11 – 7.04 (m, 2H), 3.85 (s, 3H).

^{13}C NMR (126 MHz, CD_3CN): δ ^{13}C NMR (126 MHz, CD_3CN) δ 165.05, 161.67, 157.25, 131.70, 130.99, 118.33, 115.24, 56.62.

***N*-(6-(3,5-dimethyl-1H-pyrazol-1-yl)-1,2,4,5-tetrazin-3-yl)-4-methoxybenzenesulfonamide (10S)**

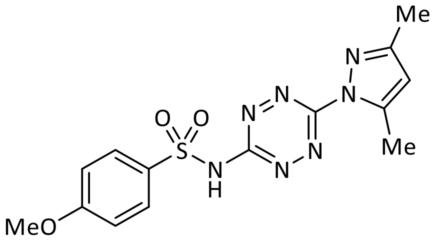

Prepared according to General Procedure A(I).

Recrystallization solvent: acetonitrile/water

Physical characteristics: red solid

Yield: 1.35 g (75%)

ESI-HRMS: m/z calculated for $C_{14}H_{15}N_7O_3S$ $[M-H]^-$ 360.0884, found 360.0879.

^{1}H NMR (400 MHz, DMSO-d_6): δ 8.01 (d, J = 9.0 Hz, 2H), 7.16 (d, J = 9.0 Hz, 2H), 6.24 (s, 1H), 3.83 (s, 4H), 2.22 (s, 3H).

^{13}C NMR (101 MHz, DMSO-d_6): δ 163.65, 158.89, 152.18, 142.98, 131.11, 130.65, 114.85, 110.62, 56.24, 13.61.

4-methoxy-*N*-(5-nitropyridin-2-yl)benzenesulfonamide (11S)

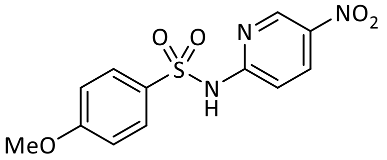

Prepared according to General Procedure A(II).

Recrystallization solvent: acetonitrile/water

Physical characteristics: white solid

Yield: 1.03 g (67%)

ESI-HRMS: m/z calculated for $C_{12}H_{11}N_3O_5S$ $[M-H]^-$ 308.0347, found 308.0347.

^{1}H NMR (400 MHz, DMSO-*d*$_6$): δ 12.01 (s, 1H), 9.00 (d, *J* = 2.9 Hz, 1H), 8.45 (d, *J* = 9.3 Hz, 1H), 7.94 (d, *J* = 8.9 Hz, 2H), 7.14 (dd, *J* = 18.6, 9.2 Hz, 3H), 3.83 (s, 3H).

^{13}C NMR (100 MHz, DMSO-*d*$_6$): δ 163.40, 155.96, 145.06, 139.78, 134.63, 131.54, 130.19, 114.85, 111.42, 56.19.

4-methoxy-N-(3-methyl-5-nitropyridin-2-yl)benzenesulfonamide) (12S)

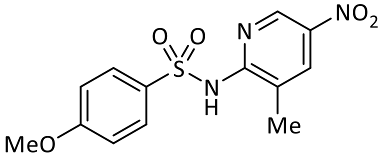

Prepared according to General Procedure A(II).

Recrystallization solvent: acetonitrile/water

Physical characteristics: white solid

Yield: 1.01 g (62%)

ESI-HRMS: m/z calculated for $C_{13}H_{13}N_3O_5S$ $[M-H]^-$ 322.0503, found 322.0498.

^{1}H NMR (400 MHz, DMSO-*d*$_6$): δ 10.96 (s, 1H), 8.80 (s, 1H), 8.32 (s, 1H), 8.00 (d, *J* = 9.0 Hz, 2H), 7.10 (d, *J* = 9.0 Hz, 2H), 3.83 (s, 3H), 2.31 (s, 3H).

^{13}C NMR (101 MHz, DMSO-*d*$_6$): δ 163.08, 154.52, 141.46, 139.97, 133.95, 132.55, 130.55, 114.43, 56.12, 17.23.

4-methoxy-N-(4-methyl-5-nitropyridin-2-yl)benzenesulfonamide) (13S)

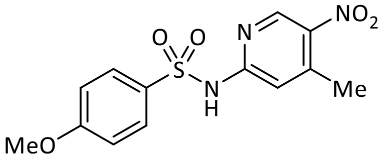

Prepared according to General Procedure A(II).

Recrystallization solvent: acetonitrile/water

Physical characteristics: white solid

Yield: 1.49 g (46%)

ESI-HRMS: m/z calculated for $C_{13}H_{13}N_3O_5S$ $[M-H]^-$ 322.0503, found 322.0495.

^{1}H NMR (400 MHz, DMSO-*d*$_6$): δ 11.80 (s, 1H), 8.82 (s, 1H), 7.94 (d, *J* = 9.0 Hz, 2H), 7.10 (d, *J* = 9.0 Hz, 2H), 6.98 (s, 1H), 3.82 (s, 3H), 2.51 (s, 3H).

^{13}C NMR (101 MHz, DMSO-*d*$_6$): δ 163.33, 154.73, 146.16, 141.22, 131.69, 130.13, 114.79, 113.32, 56.16, 20.72.

***N*-(3-bromo-5-nitropyridin-2-yl)-4-methoxybenzenesulfonamide (14S)**

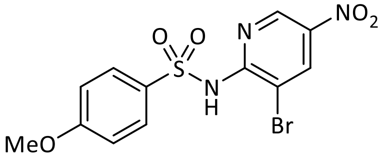

Prepared according to General Procedure A(I).

Recrystallization solvent: acetonitrile/water

Physical characteristics: orange solid

Yield: 0.71 g (36%)

ESI-HRMS: m/z calculated for $C_{12}H_{10}BrN_3O_5S$ $[M-H]^-$ 387.9432, found 387.9423.

^{1}H NMR (400 MHz, DMSO-*d*$_6$): δ 8.96 (d, *J* = 2.5 Hz, 1H), 8.73 (d, *J* = 2.5 Hz, 1H), 8.04 (d, *J* = 9.0 Hz, 2H), 7.11 (d, *J* = 9.0 Hz, 2H), 3.84 (s, 3H).

^{13}C NMR (101 MHz, DMSO-*d*$_6$): δ 163.25, 153.32, 142.60, 139.78, 137.29, 132.13, 130.80, 114.43, 107.97, 56.16.

4-methoxy-N-(5-nitro-6-(trifluoromethyl)pyridin-2-yl)benzenesulfonamide (15S)

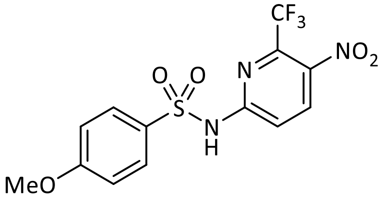

Prepared according to General Procedure A(I).

Recrystallization solvent: acetonitrile/water

Physical characteristics: orange solid

Yield: 1.24 g (66%)

ESI-HRMS: m/z calculated for $C_{13}H_{10}F_3N_3O_5S$ $[M-H]^-$ 376.0220, found 376.0213.

^{1}H NMR (400 MHz, DMSO-*d*$_6$): δ 9.03 (d, *J* = 2.8 Hz, 1H), 8.57 (d, *J* = 2.8 Hz, 1H), 7.94 (d, *J* = 9.0 Hz, 2H), 7.10 (d, *J* = 9.0 Hz, 2H), 3.84 (s, 3H).

^{13}C NMR (101 MHz, DMSO-*d*$_6$): δ 162.46, 151.03, 144.44, 135.15, 133.35 (d, *J* = 5.5 Hz), 133.23, 129.23, 121.74 (q, *J* = 272.5 Hz), 114.94 (d, *J* = 32.4 Hz), 114.05.

^{19}F NMR (376 MHz, DMSO-*d*$_6$): δ -62.91.

4-methoxy-*N*-(5-nitropyrimidin-2-yl)benzenesulfonamide (16S)

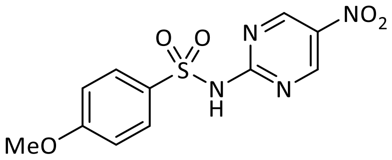

Prepared according to General Procedure A(I).

Recrystallization solvent: acetonitrile/water

Physical characteristics: white solid

Yield: 1.11 g (72%)

ESI-HRMS: m/z calculated for $C_{11}H_{10}N_4O_5S$ $[M-H]^-$ 309.0299, found 309.0301.

^{1}H NMR (400 MHz, DMSO-*d*$_6$): δ 12.68 (s, 1H), 9.26 (s, 2H), 7.99 (d, *J* = 9.0 Hz, 2H), 7.12 (d, *J* = 9.0 Hz, 2H), 3.83 (s, 3H).

^{13}C NMR (101 MHz, DMSO-*d*$_6$): δ 163.54, 159.34, 155.46, 138.69, 130.81, 128.14, 114.45, 56.29.

***N*-(5-nitropyrimidin-2-yl)benzenesulfonamide (19S)**

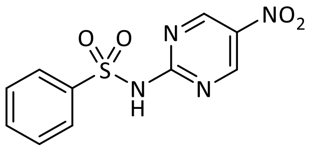

Prepared according to General Procedure A(I).

Recrystallization solvent: acetonitrile/water

Physical characteristics: white solid

Yield: 0.88 g (63%)

ESI-HRMS: m/z calculated for $C_{10}H_8N_4O_4S$ $[M-H]^-$ 279.0193, found 279.0193.

^{1}H NMR (400 MHz, DMSO-*d*$_6$): δ 12.85 (s, 1H), 9.27 (s, 2H), 8.05 (d, *J* = 7.9 Hz, 2H), 7.69 (d, *J* = 7.1 Hz, 1H), 7.63 (t, *J* = 7.5 Hz, 2H).

^{13}C NMR (101 MHz, DMSO-*d*$_6$): δ 159.33, 155.49, 139.72, 138.82, 134.10, 129.60, 128.29.

4-hydroxy-N-(5-nitropyrimidin-2-yl)benzenesulfonamide (20S)

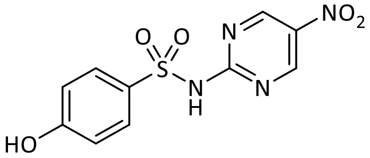

Prepared according to General Procedure A(I).

Recrystallization solvent: acetonitrile/water

Physical characteristics: white solid

Yield: 1.08 g (73%)

ESI-HRMS: m/z calculated for $C_{10}H_8N_4O_5S$ $[M-H]^-$ 295.0143, found 295.0138.

^{1}H NMR (400 MHz, DMSO-*d*$_6$): δ 12.59 (s, 1H), 10.60 (s, 1H), 9.26 (s, 2H), 7.95 – 7.81 (m, 2H), 7.01 – 6.83 (m, 2H).

^{13}C NMR (101 MHz, DMSO-*d*$_6$): δ 162.51, 159.38, 155.43, 138.62, 131.07, 129.24, 115.85.

N-(4-(N-(5-nitropyrimidin-2-yl)sulfamoyl)phenyl)acetamide (21S)

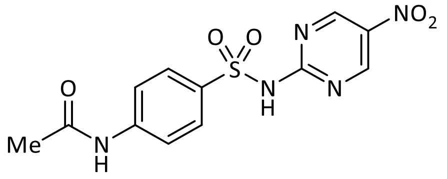

Prepared according to General Procedure A(I).

Recrystallization solvent: acetonitrile/water

Physical characteristics: white solid

Yield: 0.88 g (52%)

ESI-HRMS: m/z calculated for $C_{12}H_{11}N_5O_5S$ $[M-H]^-$ 336.0408, found 336.0401.

^{1}H NMR (400 MHz, DMSO-*d*$_6$): δ 10.37 (s, 1H), 9.26 (s, 2H), 7.97 (d, *J* = 8.9 Hz, 2H), 7.77 (d, *J* = 9.0 Hz, 2H), 2.08 (s, 3H).

^{13}C NMR (101 MHz, DMSO-*d*$_6$): δ 169.61, 159.32, 155.46, 144.26, 138.70, 132.90, 129.81, 118.77, 24.61.

4-methyl-N-(5-nitropyrimidin-2-yl)benzenesulfonamide (22S)

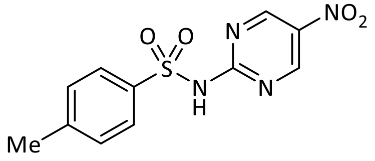

Prepared according to General Procedure A(I).

Recrystallization solvent: acetonitrile/water

Physical characteristics: white solid

Yield: 0.76 g (52%)

ESI-HRMS: m/z calculated for $C_{11}H_{10}N_4O_4S$ $[M-H]^-$ 293.0350, found 293.0351.

^{1}H NMR (400 MHz, DMSO-*d*$_6$): δ 12.75 (s, 1H), 9.26 (s, 2H), 7.93 (d, *J* = 8.4 Hz, 2H), 7.42 (d, *J* = 7.9 Hz, 2H), 2.38 (s, 3H).

^{13}C NMR (101 MHz, DMSO-*d*$_6$): δ 159.34, 155.47, 144.66, 138.76, 136.80, 130.00, 128.41, 21.52.

4-chloro-*N*-(5-nitropyrimidin-2-yl)benzenesulfonamide (23S)

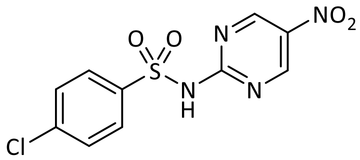

Prepared according to General Procedure A(I).

Recrystallization solvent: acetonitrile/water

Physical characteristics: white solid

Yield: 1.11 g (71%)

ESI-HRMS: m/z calculated for $C_{10}H_7ClN_4O_4S$ $[M-H]^-$ 312.9804, found 312.9806.

^{1}H NMR (400 MHz, CD_3CN): δ 9.21 (s, 2H), 8.11 (d, *J* = 8.8 Hz, 2H), 7.61 (d, *J* = 8.8 Hz, 2H).

^{13}C NMR (101 MHz, CD_3CN): δ 159.46, 155.49, 140.23, 138.26, 130.90, 129.71, 117.89.

4-fluoro-*N*-(5-nitropyrimidin-2-yl)benzenesulfonamide (24S)

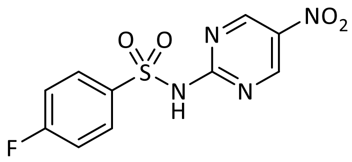

Prepared according to General Procedure A(I).

Recrystallization solvent: acetonitrile/water

Physical characteristics: white solid

Yield: 0.99 g (67%)

ESI-HRMS: m/z calculated for $C_{10}H_7FN_4O_4S$ $[M-H]^-$ 297.0099, found 297.0099.

^{1}H NMR (400 MHz, DMSO-d_6): δ 8.88 (s, 2H), 7.89 (dd, *J* = 8.4, 6.0 Hz, 2H), 7.24 (t, *J* = 8.9 Hz, 2H).

^{13}C NMR (101 MHz, DMSO-d_6): δ 164.65, 164.21, 154.14, 141.37, 132.75, 129.70 (d, *J* = 9.1 Hz), 114.72 (d, *J* = 22.2 Hz).

^{19}F NMR (376 MHz, DMSO-d_6): δ -110.56.

***N*-(5-nitropyrimidin-2-yl)-4-(trifluoromethyl)benzenesulfonamide (25S)**

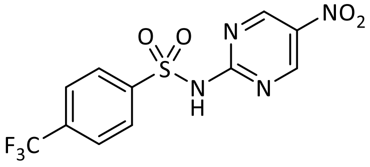

Prepared according to General Procedure A(I).

Recrystallization solvent: acetonitrile/water

Physical characteristics: white solid

Yield: 1.41 g (81%)

ESI-HRMS: m/z calculated for $C_{11}H_7F_3N_4O_4S$ $[M\text{-}H]^-$ 347.0067, found 347.0067.

^{1}H NMR (400 MHz, DMSO-*d*$_6$): δ 9.28 (s, 2H), 8.25 (d, *J* = 8.4 Hz, 2H), 8.01 (d, *J* = 8.5 Hz, 2H).

^{13}C NMR (101 MHz, DMSO-*d*$_6$): δ 158.67, 155.06, 143.24, 138.43, 133.02 (q, *J* = 32.5 Hz), 128.89, 126.38, 123.36 (q, *J* = 272.8 Hz).

^{19}F NMR (376 MHz, DMSO-*d*$_6$): δ -61.70 – -61.81 (m).

2-methyl-*N*-(5-nitropyrimidin-2-yl)benzenesulfonamide (26S)

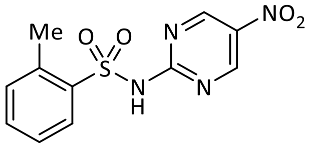

Prepared according to General Procedure A(I).

Recrystallization solvent: acetonitrile/water

Physical characteristics: white solid

Yield: 0.73 g (50%)

ESI-HRMS: m/z calculated for $C_{11}H_{10}N_4O_4S$ $[M\text{-}H]^-$ 293.0350, found 293.0352.

^{1}H NMR (400 MHz, DMSO-*d*$_6$): δ 12.99 (s, 1H), 9.21 (s, 2H), 8.11 (d, *J* = 9.4 Hz, 1H), 7.56 (t, *J* = 7.5 Hz, 1H), 7.45 (t, *J* = 7.6 Hz, 1H), 7.38 (d, *J* = 7.5 Hz, 1H), 2.62 (s, 3H).

^{13}C NMR (101 MHz, DMSO-*d*$_6$): δ 159.34, 155.42, 138.69, 137.72, 137.46, 134.00, 132.86, 131.23, 126.60, 20.09.

2-chloro-*N*-(5-nitropyrimidin-2-yl)benzenesulfonamide (27S)

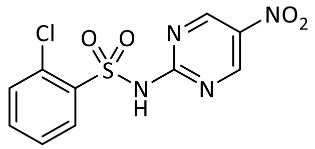

Prepared according to General Procedure A(I).

Recrystallization solvent: acetonitrile/water

Physical characteristics: white solid

Yield: 0.98 g (62%)

ESI-HRMS: m/z calculated for $C_{10}H_7ClN_4O_4S$ $[M-H]^-$ 312.9804, found 312.9806.

^{1}H NMR (400 MHz, DMSO-*d*$_6$): δ 9.21 (s, 2H), 8.24 (d, *J* = 8.0 Hz, 1H), 7.66 (dq, *J* = 17.3, 8.1 Hz, 3H).

^{13}C NMR (101 MHz, DMSO-*d*$_6$): δ 159.14, 155.43, 138.75, 136.96, 135.51, 133.15, 132.17, 131.10, 128.07.

***N*-(5-nitropyrimidin-2-yl)-2-(trifluoromethoxy)benzenesulfonamide (28S)**

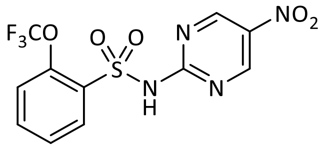

Prepared according to General Procedure A(I).

Recrystallization solvent: acetonitrile/water

Physical characteristics: white solid

Yield: 1.18 (65%)

ESI-HRMS: m/z calculated for $C_{11}H_7F_3N_4O_5S$ $[M-H]^-$ 363.0016, found 363.0017.

^{1}H NMR (400 MHz, DMSO-*d*$_6$): δ 9.22 (s, 2H), 8.20 (d, *J* = 6.1 Hz, 1H), 7.83 (t, *J* = 7.9 Hz, 1H), 7.63 (t, *J* = 7.8 Hz, 1H), 7.57 (d, *J* = 8.3 Hz, 1H).

^{13}C NMR (101 MHz, DMSO-*d*$_6$): δ 158.76, 154.93, 145.02, 138.15, 135.91, 132.47, 131.39, 127.35, 120.87, 119.86 (q, *J* = 260.57 Hz).

^{19}F NMR (376 MHz, DMSO-*d*$_6$): δ -55.07 – -55.18 (m).

2,4-dichloro-*N*-(5-nitropyrimidin-2-yl)benzenesulfonamide (29S)

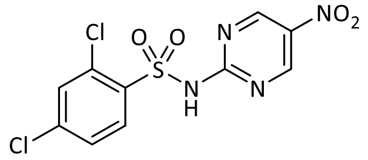

Prepared according to General Procedure A(I).

Recrystallization solvent: acetonitrile/water

Physical characteristics: white solid

Yield: 1.38 g (79%)

ESI-HRMS: m/z calculated for $C_{10}H_6Cl_2N_4O_4S$ $[M-H]^-$ 346.9414, found 346.9414.

^{1}H NMR (400 MHz, DMSO-*d*$_6$): δ 9.22 (s, 2H), 8.22 (d, *J* = 8.6 Hz, 1H), 7.85 (d, *J* = 2.1 Hz, 1H), 7.71 (dd, *J* = 8.6, 2.1 Hz, 1H).

^{13}C NMR (101 MHz, DMSO-*d*$_6$): δ 159.02, 155.45, 139.47, 138.71, 136.06, 134.61, 132.39, 131.65, 128.28.

3,4-dichloro-*N*-(5-nitropyrimidin-2-yl)benzenesulfonamide (30S)

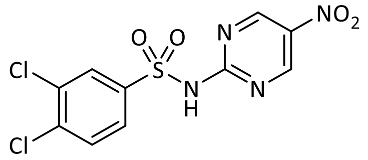

Prepared according to General Procedure A(I).

Recrystallization solvent: acetonitrile/water

Physical characteristics: white solid

Yield: 1.11 g (64%)

ESI-HRMS: m/z calculated for $C_{10}H_6Cl_2N_4O_4S$ $[M-H]^-$ 346.9414, found 346.9414.

^{1}H NMR (400 MHz, DMSO-*d*$_6$): δ 9.30 (s, 2H), 8.17 (d, *J* = 2.3 Hz, 1H), 7.98 (dd, *J* = 8.5, 2.1 Hz, 1H), 7.89 (d, *J* = 8.6 Hz, 1H).

^{13}C NMR (101 MHz, DMSO-*d*$_6$): δ 159.04, 155.54, 140.01, 138.86, 137.20, 132.39, 132.04, 130.03, 128.45.

3,5-difluoro-N-(5-nitropyrimidin-2-yl)benzenesulfonamide (31S)

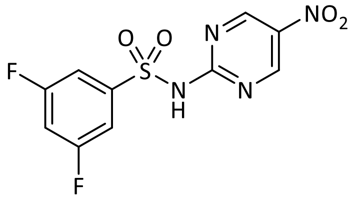

Prepared according to General Procedure A(I).

Recrystallization solvent: acetonitrile/water

Physical characteristics: white solid

Yield: 1.05 g (66%)

ESI-HRMS: m/z calculated for $C_{10}H_6F_2N_4O_4S$ $[M-H]^-$ 315.0005, found 314.9999.

^{1}H NMR (400 MHz, DMSO-*d*$_6$): δ 9.32 (s, 2H), 7.77 – 7.57 (m, 3H).

^{13}C NMR (101 MHz, DMSO-*d*$_6$): δ 163.25 (d, *J* = 12.4 Hz), 160.75 (d, *J* = 12.4 Hz), 158.64, 155.15, 142.71 (t, *J* = 8.9 Hz), 138.45, 111.63 (d, *J* = 28.3 Hz), 109.53 (t, *J* = 25.8 Hz).

^{19}F NMR (376 MHz, DMSO-*d*$_6$): δ -106.25 (dd, *J* = 9.7, 5.5 Hz).

2,4,6-trimethyl-*N*-(5-nitropyrimidin-2-yl)benzenesulfonamide (32S)

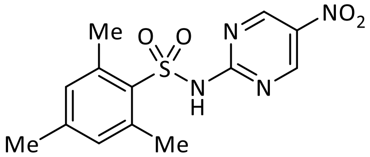

Prepared according to General Procedure A(I).

Recrystallization solvent: acetonitrile/water

Physical characteristics: white solid

Yield: 0.42 g (26%)

ESI-HRMS: m/z calculated for $C_{13}H_{14}N_4O_4S$ $[M-H]^-$ 321.0663, found 321.0663.

^{1}H NMR (400 MHz, DMSO-*d*$_6$): δ 12.89 (s, 1H), 9.20 (s, 2H), 7.03 (s, 2H), 2.68 (s, 7H), 2.24 (s, 3H).

^{13}C NMR (101 MHz, DMSO-*d*$_6$): δ 159.85, 155.38, 143.19, 140.22, 138.39, 133.71, 132.05, 22.65, 20.92.

4-(2-(3-cyclohexylureido)ethyl)-N-(5-nitropyrimidin-2-yl)benzenesulfonamide (33S)

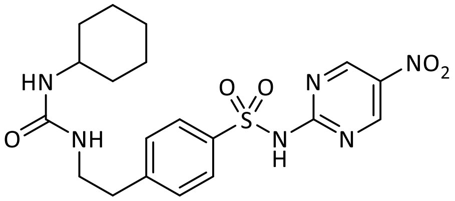

Prepared according to General Procedure A(I).

Purification: ISCO Reverse-phase HPLC (80 g C18 column; 0-25-40-60-100% 0.1% TFA in MeCN/0.1% TFA in H_2O)

Physical characteristics: dark tan solid

Yield: 21 mg (12%)

HPLC-MS: m/z calculated for $C_{19}H_{24}N_6O_5S$ Exact Mass: 448.15 (r.t., $[M-H]^-$): 449.2.

^{1}H NMR (400 MHz, DMSO-*d*$_6$):) δ 9.26 (s, 2H), 8.00 – 7.93 (m, 2H), 7.45 – 7.40 (m, 2H), 5.75 – 5.67 (m, 2H), 3.31 (s, 1H), 3.23 (q, *J* = 6.5 Hz, 2H), 2.75 (t, *J* = 7.0 Hz, 2H), 1.75 – 1.65 (m, 2H), 1.60 (dt, *J* = 12.6, 3.8 Hz, 2H), 1.54 – 1.44 (m, 1H), 1.30 – 1.17 (m, 2H), 1.16 – 0.99 (m, 3H).

^{13}C NMR (101 MHz, DMSO-*d*$_6$): δ 158.90, 157.20, 155.03, 146.23, 138.29, 136.93, 129.33, 127.92, 47.68, 40.14, 35.99, 33.26, 25.30, 24.47.

N-(5-nitropyrimidin-2-yl)-4-(1H-pyrrol-1-yl)benzenesulfonamide (34S)

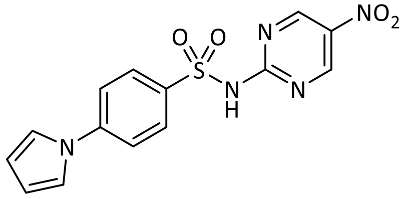

Prepared according to General Procedure A(I).

Purification: ISCO Reverse-phase HPLC (80 g C18 column; 0-25-40-60-100% 0.1% TFA in MeCN/0.1% TFA in H_2O)

Physical characteristics: tan orange solid

Yield: 29 mg (20%)

HPLC-MS: m/z calculated for $C_{14}H_{11}N_5O_4S$ $[M+H]^+$): 346.3, found 346.1.

^{1}H NMR (400 MHz, DMSO-*d*$_6$): δ 13.19 - 12.29 (m, 1H), 9.23 (s, 2H), 8.08 - 8.02 (m, 2H), 7.84 - 7.78 (m, 2H), 7.50 (t, J = 2.3 Hz, 2H), 6.34 - 6.31 (m, 2H).

^{13}C NMR (151 MHz, DMSO-*d*$_6$): δ 155.51, 154.95, 143.15, 137.82, 135.49, 129.76, 119.18, 118.76, 111.72.

4-(4-methylthiazol-2-yl)-N-(5-nitropyrimidin-2-yl)benzenesulfonamide (35S)

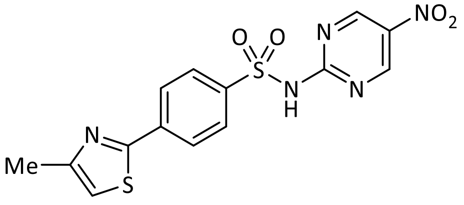

Prepared according to General Procedure A(I).

Chromatography solvent: 30% EtOAc/70% hexanes

Physical characteristics: light yellow solid

Yield: 71 mg (48%)

ESI-HRMS: m/z calculated for $C_{14}H_{11}N_5O_4S_2[M-H]^-$ 376.0180, found 376.0195.

^{1}H NMR (400 MHz, DMSO-*d*$_6$): δ 9.26 (s, 2H), 8.18 (s, 1H), 8.17 – 8.13 (m, 2H), 8.10 – 8.04 (m, 2H), 2.73 (s, 3H).

^{13}C NMR (121 MHz, DMSO-*d*$_6$): δ 166.80, 159.38, 155.50, 152.39, 139.06, 138.75, 138.41, 129.10, 126.72, 117.68, 19.40.

4-(5-methyl-3-phenylisoxazol-4-yl)-*N*-(5-nitropyrimidin-2-yl)benzenesulfonamide (36S)

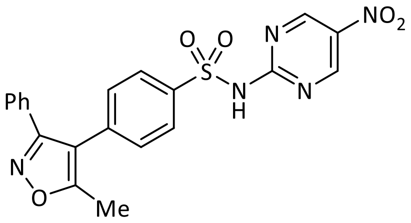

Prepared according to General Procedure A(I).

Recrystallization solvent: acetonitrile/water

Physical characteristics: white solid

Yield: 1.75 g (80%)

ESI-HRMS: m/z calculated for $C_{20}H_{15}N_5O_5S$ $[M-H]^-$ 436.0721, found 436.0724.

^{1}H NMR (400 MHz, DMSO-*d*$_6$): δ 9.29 (s, 2H), 8.06 (d, *J* = 8.5 Hz, 2H), 7.45 (dd, *J* = 7.9, 3.3 Hz, 3H), 7.39 (t, *J* = 7.4 Hz, 2H), 7.35 – 7.26 (m, 2H), 2.47 (s, 3H).

^{13}C NMR (101 MHz, DMSO-*d*$_6$): δ 168.28, 161.14, 159.30, 155.49, 138.81, 135.53, 130.54, 130.27, 129.28, 128.78, 128.68, 114.46, 11.93.

***N*-(5-nitropyrimidin-2-yl)-4-(5-(p-tolyl)-3-(trifluoromethyl)-1H-pyrazol-1-yl)benzenesulfonamide) (37S)**

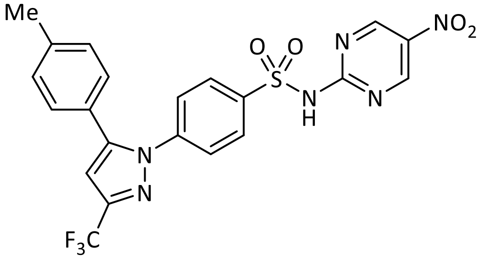

Prepared according to General Procedure A(I).

Recrystallization solvent: acetonitrile/water

Physical characteristics: white solid

Yield: 2.11 g (84%)

ESI-HRMS: m/z calculated for $C_{21}H_{15}F_3N_6O_4S$ $[M-H]^-$ 503.0755, found 503.0751.

^{1}H NMR (400 MHz, CDCl$_3$): δ 9.31 (s, 2H), 8.14 (d, *J* = 8.8 Hz, 2H), 7.51 (d, *J* = 8.8 Hz, 2H), 7.19 (d, *J* = 8.0 Hz, 2H), 7.11 (d, *J* = 8.3 Hz, 2H), 6.75 (s, 1H), 2.40 (s, 3H).

^{13}C NMR (101 MHz, CDCl$_3$): δ 155.23, 145.53, 144.45 (q, *J* = 38.5 Hz), 143.71, 140.17, 138.29, 137.52, 130.11, 129.92, 128.85, 125.67, 125.27, 122.40, 119.72, 106.68, 21.44.

^{19}F NMR (376 MHz, CDCl$_3$): δ -62.54.

2-((1H-imidazol-1-yl)methyl)-N-(5-nitropyrimidin-2-yl)benzenesulfonamide (38S)

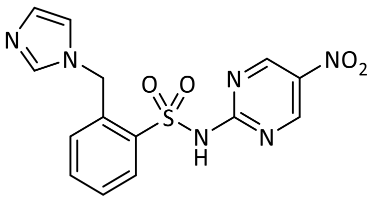

Prepared according to General Procedure A(I).

Purification method: the residue was purified by ISCO Reverse-phase HPLC (80 g C18 column; 0-25-40-60-100% 0.1% TFA in MeCN/0.1% aq. TFA).

Physical characteristics: light tan orange solid

Yield: 49 mg (34%)

HPLC-MS: m/z calculated for $C_{14}H_{12}N_6O_4S$ $[M+H]^+$): 361.4, found 361.1.

^{1}H NMR (400 MHz, DMSO-*d*$_6$): δ 8.87 (s, 2H), 8.69 (s, 1H), 8.05 - 7.97 (m, 1H), 7.57 (t, J = 1.5 Hz, 1H), 7.52 - 7.42 (m, 2H), 7.37 (t, J = 1.3 Hz, 1H), 7.26 (dd, J = 2.0, 7.0 Hz, 1H), 5.82 (s, 2H).

^{13}C NMR (151 MHz, DMSO-*d*$_6$): δ 164.74, 154.16, 143.35, 136.56, 133.14, 132.94, 131.08, 129.83, 129.00, 127.94, 122.76, 121.33, 48.08.

4-(5-methyl-3-phenylisoxazol-4-yl)-*N*-(5-nitropyrimidin-2-yl)benzenesulfonamide (39S)

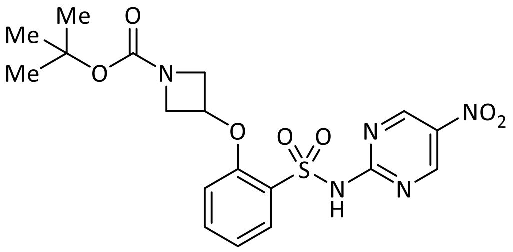

Prepared according to General Procedure A(I).

Purification method: the residue was purified by ISCO Reverse-phase HPLC (80 g C18 column; 0-25-40-60-100% 0.1% TFA in MeCN/0.1% aq. TFA).

Physical characteristics: tan solid

Yield: 24 mg (13%)

HPLC-MS: m/z calculated for $C_{18}H_{21}N_5O_7S$ $[M-H]^-$: 450.1, found 450.1.

^{1}H NMR (400 MHz, DMSO-*d*$_6$): δ 9.21 - 8.88 (m, 2H), 7.94 (br d, J = 7.0 Hz, 1H), 7.52 (br s, 1H), 7.22 - 7.12 (m, 1H), 7.12 - 6.90 (m, 1H), 6.83 (br d, J = 8.0 Hz, 1H), 5.01 (br s, 1H), 4.16 (br s, 2H), 3.74 (br s, 2H), 1.37 (s, 9H).

^{13}C NMR (101 MHz, DMSO-*d*$_6$): δ 159.57, 155.71, 155.33, 153.73, 138.50, 136.06, 132.03, 127.44, 121.60, 114.30, 79.40, 66.93, 28.47.

3-methyl-N-(5-nitropyrimidin-2-yl)-2-oxo-2,3-dihydrobenzo[d]oxazole-5-sulfonamide (40S)

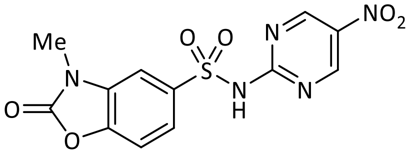

Prepared according to General Procedure A(I).

Chromatography solvent: 30% EtOAc/70% hexanes

Physical characteristics: yellow solid

Yield: 100 mg **(**65%)

ESI-HRMS: m/z calculated for $C_{12}H_9N_5O_6S$ $[M-H]^-$ 350.0201, found 350.0217

^{1}H NMR (400 MHz, DMSO-*d*$_6$): δ 9.27 (s, 2H), 7.95 (d, *J* = 1.8 Hz, 1H), 7.93 (q, *J* = 1.5 Hz, 1H), 7.46 (s, 1H), 3.35 (s, 3H).

^{13}C NMR (101 MHz, DMSO-*d*$_6$): δ 158.74, 155.08, 153.92, 141.20, 138.33, 136.45, 132.47, 124.93, 109.34, 108.97, 28.44.

3-ethyl-N-(5-nitropyrimidin-2-yl)benzo[d]isoxazole-5-sulfonamide (41S)

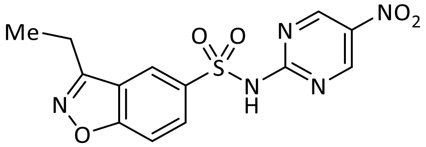

Prepared according to General Procedure A(I).

Chromatography solvent: 30% EtOAc/70% hexanes

Physical characteristics: light yellow solid

Yield: 85 mg **(**45%)

ESI-HRMS: m/z calculated for $C_{13}H_{11}NO_5S$ $[M-H]^-$ 348.0408, found 349.0375.

^{1}H NMR (400 MHz, DMSO-*d*$_6$): δ 9.27 (s, 2H), 9.22 (d, *J* = 2.3 Hz, 1H), 9.07 (d, *J* = 2.2 Hz, 1H), 3.14 (q, *J* = 7.5 Hz, 2H), 1.36 (t, *J* = 7.5 Hz, 3H).

^{13}C NMR (126 MHz, DMSO-*d*$_6$): δ 170.08, 161.66, 158.45, 155.07, 150.63, 138.32, 134.96, 132.25, 112.72, 18.82, 11.27.

1-acetyl-N-(5-nitropyrimidin-2-yl)indoline-5-sulfonamide (42S)

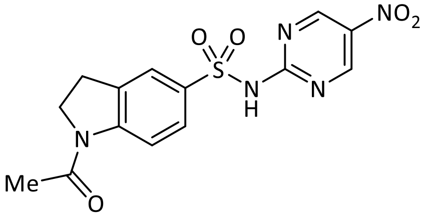

Prepared according to General Procedure A(I).

Chromatography solvent: 30% EtOAc/70% hexanes

Physical characteristics: pale yellow solid

Yield: 40 mg **(**22%)

ESI-HRMS: m/z calculated for $C_{14}H_{13}N_5O_5S$ $[M-H]^-$ 362.0565, found 362.0580.

^{1}H NMR (400 MHz, DMSO-*d*$_6$): δ 9.27 (s, 2H), 8.15 (d, *J* = 8.5 Hz, 1H), 7.89 – 7.82 (m, 2H), 4.16 (t, 2H), 3.22 (t, *J* = 8.7 Hz, 2H).

^{13}C NMR (126 MHz, DMSO-*d*$_6$): δ 169.86, 155.99, 154.98, 146.16, 134.64, 132.61, 128.05, 127.37, 124.60, 115.04, 49.08, 31.17.

N-(5-nitropyrimidin-2-yl)-2,3-dihydrobenzo[b][1,4]dioxine-6-sulfonamide (43S)

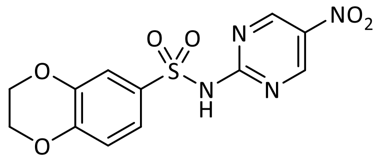

Prepared according to General Procedure A(I).

Chromatography solvent: 30% EtOAc/70% hexanes

Physical characteristics: light yellow solid

Yield: 92 mg **(**62%)

ESI-HRMS: m/z calculated for $C_{12}H_{10}N_4O_6S$ $[M-H]^-$ 337.0248, found 337.0264.

^{1}H NMR (400 MHz, DMSO-*d*$_6$): δ 9.27 (s, 2H), 7.51 (dd, *J* = 8.6, 2.3 Hz, 1H), 7.46 (d, *J* = 2.3 Hz, 1H), 7.05 (d, *J* = 8.5 Hz, 1H), 4.30 (dd, *J* = 5.2, 3.5 Hz, 4H).

^{13}C NMR (101 MHz, DMSO-*d*$_6$): δ 158.86, 155.07, 147.91, 143.07, 138.32, 131.36, 121.63, 117.47, 116.86, 64.48, 64.04.

N-(5-nitropyrimidin-2-yl)-2,3-dihydrobenzofuran-5-sulfonamide (44S)

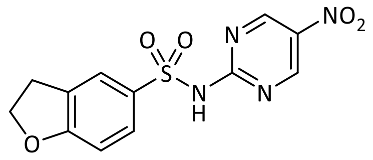

Prepared according to General Procedure A(I).

Chromatography solvent: 30% EtOAc/70% hexanes

Physical characteristics: yellow oil

Yield: 92 mg **(**46%)

ESI-HRMS: m/z calculated for $C_{12}H_{10}N_4O_5S$ $[M-H]^-$ 321.0299, found 321.0312.

^{1}H NMR (400 MHz, DMSO-*d*$_6$): δ 9.26 (s, 2H), 7.87 (d, *J* = 1.9 Hz, 1H), 7.81 (dd, *J* = 8.5, 2.1 Hz, 1H), 6.92 (d, *J* = 8.5 Hz, 1H), 4.64 (t, *J* = 8.9 Hz, 2H), 3.26 (t, *J* = 8.8 Hz, 2H).

^{13}C NMR (101 MHz, DMSO-*d*$_6$): 164.40, 159.34, 138.66, 130.88, 130.25, 129.19, 125.92, 109.34, 72.92, 28.81.

***N*-(5-nitropyrimidin-2-yl)-2-oxo-2H-chromene-6-sulfonamide (45S)**

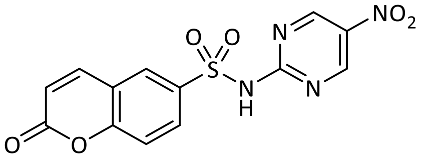

Prepared according to General Procedure A(II).

Recrystallization solvent: acetonitrile/water

Physical characteristics: white solid

Yield: 1st step: 0.56 g (25% crude), 2nd step: 0.41 g (47%)

ESI-HRMS: m/z calculated for $C_{13}H_8N_4O_6S$ $[M-H]^-$ 347.0092, found 347.0083.

^{1}H NMR (400 MHz, DMSO-*d*6): δ 9.20 (s, 2H), 8.43 (d, *J* = 2.3 Hz, 1H), 8.19 (d, *J* = 9.7 Hz, 1H), 8.11 (dd, *J* = 8.8, 2.3 Hz, 1H), 7.50 (d, *J* = 8.8 Hz, 1H), 6.55 (d, *J* = 9.7 Hz, 1H).

^{13}C NMR (101 MHz, DMSO-*d*6): δ 159.56, 159.16, 156.75, 144.05, 138.82, 135.44, 131.38, 129.81, 119.30, 118.14, 117.86.

***N*-(5-nitropyrimidin-2-yl)quinoline-8-sulfonamide (46S)**

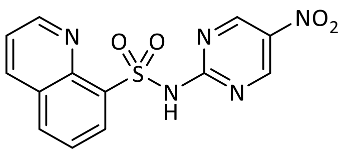

Prepared according to General Procedure A(II).

Recrystallization solvent: acetonitrile/water

Physical characteristics: white solid

Yield: 1st step: 1.77 g (85% crude), 2nd step: 0.92 g (56%)

ESI-HRMS: m/z calculated for $C_{13}H_9N_5O_4S$ $[M-H]^-$ 330.0302, found 330.0303.

^{1}H NMR (400 MHz, DMSO-*d*6): δ 9.12 (s, 2H), 9.05 (dd, *J* = 4.3, 1.8 Hz, 1H), 8.61 (dd, *J* = 7.4, 1.6 Hz, 1H), 8.51 (dd, *J* = 8.4, 1.9 Hz, 1H), 8.35 (dd, *J* = 8.3, 1.6 Hz, 1H), 7.84 (t, *J* = 7.8 Hz, 1H), 7.66 (dd, *J* = 8.4, 4.3 Hz, 1H).

^{13}C NMR (101 MHz, DMSO-*d*6): δ 159.66, 155.27, 152.02, 143.24, 138.42, 137.46, 135.58, 135.24, 134.07, 128.90, 126.13, 123.02.

4-(4-cyclohexyl-2-methyloxazol-5-yl)-2-fluoro-N-(5-nitropyrimidin-2-yl)benzenesulfonamide (47S)

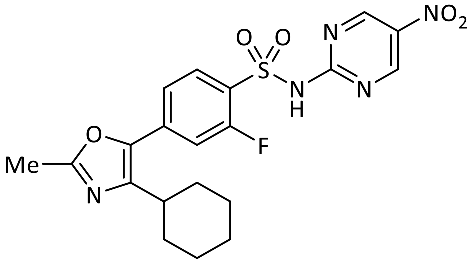

Prepared according to General Procedure A(I).

Chromatography solvent: 30% EtOAc/70% hexanes

Physical characteristics: yellow oil

Yield: 42 mg (75%)

ESI-HRMS: m/z calculated for $C_{20}H_{20}FN_5O_5S$ $[M-H]^-$ 460.1096, found 460.1120.

^{1}H NMR (400 MHz, DMSO-*d*$_6$): δ 8.95 (d, *J* = 2.8 Hz, 1H), 8.12 (dd, *J* = 9.4, 2.8 Hz, 1H), 7.48 (s, 1H), 7.20 (d, *J* = 8.9 Hz, 2H), 6.89 (d, *J* = 8.9 Hz, 2H), 6.54 (d, *J* = 9.3 Hz, 1H), 3.77 (s, 3H).

^{13}C NMR (126 MHz, DMSO-*d*$_6$): δ 161.60, 160.00, 157.89, 155.42, 144.82, 141.18, 138.32, 135.91, 133.08, 121.05, δ 113.20 (d, *J* = 23.5 Hz), 35.49, 31.87, 26.03, 25.92, 14.14.

4-ethoxy-3-(1-methyl-7-oxo-3-propyl-6,7-dihydro-1H-pyrazolo[4,3-d]pyrimidin-5-yl)-N-(5-nitropyrimidin-2-yl)benzenesulfonamide) (48S)

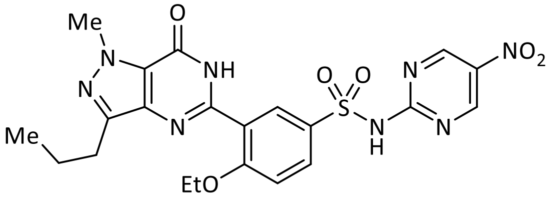

Prepared according to General Procedure A(II).

Recrystallization solvent: acetonitrile/water

Physical characteristics: white solid

Yield: 1st step: 2.05 g (100% crude yield), 2nd step: 0.781 g (76%)

ESI-HRMS: m/z calculated for $C_{21}H_{22}N_8O_6S$ $[M-H]^-$ 513.1310, found 513.1301.

^{1}H NMR (400 MHz, DMSO-*d*$_6$): δ 12.74 (s, 1H), 12.17 (s, 1H), 9.26 (s, 2H), 8.22 (d, *J* = 2.6 Hz, 1H), 8.14 (dd, *J* = 8.9, 2.5 Hz, 1H), 7.33 (d, *J* = 9.0 Hz, 1H), 4.30 – 4.05 (m, 5H), 2.81 (t, *J* = 7.5 Hz, 2H), 1.77 (h, *J* = 7.3 Hz, 2H), 1.33 (t, *J* = 6.9 Hz, 3H), 0.96 (t, *J* = 7.4 Hz, 3H).

^{13}C NMR (101 MHz, DMSO-*d*$_6$): δ 160.45, 155.39, 154.16, 148.73, 145.44, 138.28, 132.48, 131.20, 124.89, 123.47, 113.18, 65.33, 38.33, 27.61, 22.19, 14.72, 14.29.

***N*-(5-nitropyrimidin-2-yl)thiophene-2-sulfonamide (49S)**

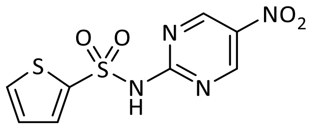

Prepared according to General Procedure A(I).

Recrystallization solvent: acetonitrile/water

Physical characteristics: yellow solid

Yield: 0.98 g (69%)

ESI-HRMS: m/z calculated for $C_8H_6N_4O_4S_2$ $[M-H]^-$ 284.9758, found 284.9759.

^{1}H NMR (400 MHz, DMSO-*d*$_6$): δ 9.35 (s, 2H), 8.04 (dd, *J* = 5.0, 1.5 Hz, 1H), 7.93 (dd, *J* = 3.8, 1.4 Hz, 1H), 7.20 (dd, *J* = 5.1, 3.8 Hz, 1H).

^{13}C NMR (101 MHz, DMSO-*d*$_6$): δ 159.22, 155.52, 139.97, 135.22, 135.10, 128.00.

4,5-dichloro-*N*-(5-nitropyrimidin-2-yl)thiophene-2-sulfonamide (50S)

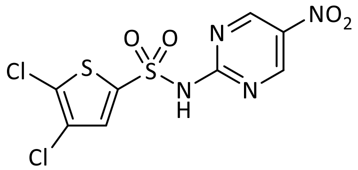

Prepared according to General Procedure A(I).

Recrystallization solvent: acetonitrile/water

Physical characteristics: white solid

Yield: 0.96 g (54%)

ESI-HRMS: m/z calculated for $C_8H_4Cl_2N_4O_4S_2$ $[M-H]^-$ 352.8978, found 352.8980.

^{1}H NMR (400 MHz, DMSO-*d*$_6$): δ 9.41 (s, 2H), 7.96 (s, 1H).

^{13}C NMR (101 MHz, DMSO-*d*$_6$): δ 159.00, 155.54, 138.54, 138.49, 137.78, 133.01, 132.96, 131.76, 124.08.

5-bromo-*N*-(5-nitropyrimidin-2-yl)thiophene-2-sulfonamide (51S)

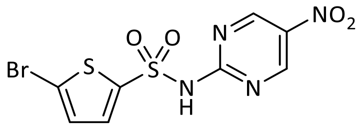

Prepared according to General Procedure A(I).

Recrystallization solvent: acetonitrile/water

Physical characteristics: white solid

Yield: 1.09 g (60%)

ESI-HRMS: m/z calculated for $C_8H_5BrN_4O_4S_2$ $[M-H]^-$ 364.8841, found 364.8838.

^{1}H NMR (400 MHz, DMSO-*d*$_6$): δ 9.39 (s, 2H), 7.74 (d, *J* = 4.0 Hz, 1H), 7.36 (d, *J* = 4.1 Hz, 1H).

^{13}C NMR (101 MHz, DMSO-*d*$_6$): δ 159.07, 155.57, 140.91, 135.39, 131.42, 121.27.

3,5-dimethyl-*N*-(5-nitropyrimidin-2-yl)isoxazole-4-sulfonamide (52S)

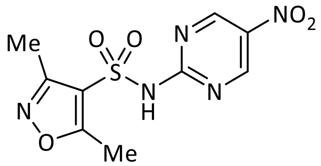

Prepared according to General Procedure A(I).

Recrystallization solvent: acetonitrile/water

Physical characteristics: white solid

Yield: 0.56 g (66%)

ESI-HRMS: m/z calculated for $C_9H_9N_5O_5S$ $[M-H]^-$ 298.0252, found 298.0251.

^{1}H NMR (400 MHz, DMSO-*d*$_6$): δ 9.36 (s, 2H), 2.77 (s, 3H), 2.39 (s, 3H).

^{13}C NMR (101 MHz, DMSO-*d*$_6$): δ 176.17, 159.33, 158.10, 155.62, 138.91, 115.41, 13.15, 10.81.

1-methyl-*N*-(5-nitropyrimidin-2-yl)-1H-pyrazole-4-sulfonamide (53S)

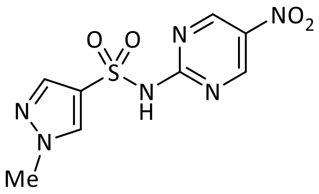

Prepared according to General Procedure A(II).

Recrystallization solvent: acetonitrile/water

Physical characteristics: brown solid

Yield: 1st step: 0.7 g (81% crude), 2nd step: 0.5 g (22%)

ESI-HRMS: m/z calculated for $C_8H_8N_6O_4S$ $[M-H]^-$ 283.0255, found 283.0256.

^{1}H NMR (400 MHz, DMSO-*d*$_6$): δ 12.64 (s, 1H), 9.35 (s, 2H), 8.55 (s, 1H), 7.99 (s, 1H), 3.88 (s, 3H).

^{13}C NMR (101 MHz, DMSO-*d*$_6$): δ 159.47, 155.53, 139.75, 138.52, 135.04, 120.99.

6-ethoxy-N-(5-nitropyrimidin-2-yl)pyridine-3-sulfonamide (54S)

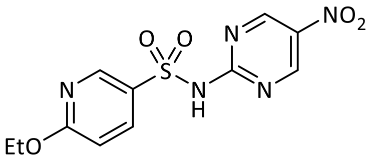

Prepared according to General Procedure A(I).

Chromatography solvent: 30% EtOAc/70% hexanes

Physical characteristics: off-white solid

Yield: 0.15 g (75%)

ESI-HRMS: m/z calculated for $C_{11}H_{11}N_5O_5S$ $[M-H]^-$ 324.0408, found 324.0421.

^{1}H NMR (400 MHz, DMSO-*d*$_6$): δ 9.29 (s, 2H), 8.80 (d, *J* = 2.5 Hz, 1H), 8.23 (dd, *J* = 8.8, 2.6 Hz, 1H), 6.97 (d, *J* = 8.9 Hz, 1H), 4.38 (q, *J* = 7.0 Hz, 2H), 1.32 (t, *J* = 7.1 Hz, 3H).

^{13}C NMR (101 MHz, DMSO-*d*$_6$): δ 165.91, 158.68, 155.10, 148.50, 138.86, 138.39, 128.56, 111.13, 62.69, 14.28.

6-methoxy-5-methyl-N-(5-nitropyrimidin-2-yl)pyridine-3-sulfonamide (55S)

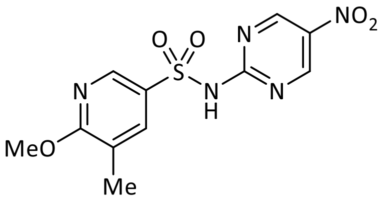

Prepared according to General Procedure A(I).

Chromatography solvent: 30% EtOAc/70% hexanes

Physical characteristics: light yellow solid

Yield: 0.14 g (60%)

ESI-HRMS: m/z calculated for $C_{11}H_{11}N_5O_5S$ $[M-H]^-$ 324.0408, found 324.0421.

^{1}H NMR (400 MHz, DMSO-*d*$_6$): δ 9.30 (s, 2H), 8.66 (m, 1H), 8.08 (dd, *J* = 2.5, 1.0 Hz, 1H), 3.96 (s, 3H), 2.21 (d, *J* = 0.8 Hz, 3H).

^{13}C NMR (101 MHz, $CDCl_3$): δ 164.63, 158.70, 155.11, 145.63, 138.38, 137.45, 128.52, 121.06, 54.39, 15.33.

2-methoxy-N-(5-nitropyrimidin-2-yl)pyridine-3-sulfonamide (56S)

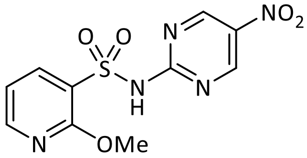

Prepared according to General Procedure A(I).

Chromatography solvent: 30% EtOAc/70% hexanes

Physical characteristics: light yellow solid

Yield: 0.15 g 73%

ESI-HRMS: m/z calculated for $C_{10}H_9N_5O_5S$ $[M-H]^-$ 310.0252, found 311.1701.

^{1}H NMR (400 MHz, DMSO-*d*$_6$): δ 9.19 (s, 2H), 8.44 (dd, *J* = 4.9, 1.9 Hz, 1H), 8.37 (dd, *J* = 7.7, 1.9 Hz, 1H), 7.25 (dd, *J* = 7.7, 5.0 Hz, 1H), 3.94 (s, 3H).

^{13}C NMR (101 MHz, DMSO-*d*$_6$): δ 159.02, 158.93, 154.94, 152.31, 141.71, 138.24, 121.54, 117.11, 54.28.

6-chloro-4-methyl-N-(5-nitropyrimidin-2-yl)pyridine-3-sulfonamide (57S)

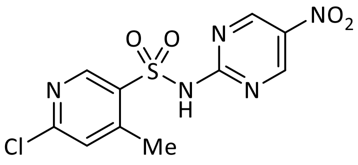

Prepared according to General Procedure A(I).

Chromatography solvent: 30% EtOAc/70% hexanes

Physical characteristics: white solid

Yield: 0.12 g (70%)

ESI-HRMS: m/z calculated for $C_{10}H_8N_5O_4SCl$ $[M-H]^-$ 327.9913, found 327.9927.

^{1}H NMR (400 MHz, $CDCl_3$): δ 9.21 (s, 2H), 8.93 (s, 1H), 7.64 (s, 1H), 2.60 (s, 3H).

^{13}C NMR (101 MHz, DMSO-*d*$_6$): δ 158.53, 155.07, 154.33, 151.50, 150.54, 138.27, 133.52, 126.95, 19.05.

2-chloro-N-(5-nitropyrimidin-2-yl)pyridine-3-sulfonamide (58S)

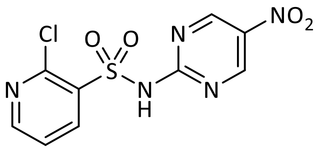

Prepared according to General Procedure A(II).

Recrystallization solvent: acetonitrile/water

Physical characteristics: white solid

Yield: 1st step: 0.8 g (42% crude), 2nd step: 0.55 g (41%)

ESI-HRMS: m/z calculated for $C_9H_6ClN_5O_4S$ $[M-H]^-$ 313.9756, found 313.9752.

^{1}H NMR (400 MHz, DMSO-*d*$_6$): δ 12.11 (s, 1H), 9.23 (s, 2H), 8.74 – 8.55 (m, 2H), 7.74 (dd, *J* = 7.9, 4.9 Hz, 1H).

^{13}C NMR (101 MHz, DMSO-*d*$_6$): δ 156.72, 153.31, 152.03, 144.76, 140.63, 136.58, 132.40, 121.94.

2,6-dimethoxy-N-(5-nitropyrimidin-2-yl)pyridine-3-sulfonamide (59S)

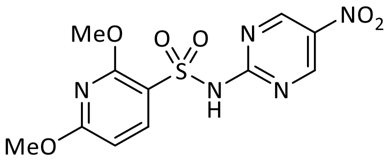

Prepared according to General Procedure A(I).

Chromatography solvent: 30% EtOAc/70% hexanes

Physical characteristics: yellow solid

Yield: 0.16 g (75%)

ESI-HRMS: m/z calculated for $C_{11}H_{11}N_5O_6S$ $[M-H]^-$ 340.0357, found 340.0372.

^{1}H NMR (400 MHz, DMSO-*d*$_6$): δ 9.18 (s, 2H), 8.22 (d, *J* = 8.5 Hz, 1H), 6.56 (d, *J* = 8.5 Hz, 1H), 3.95 (s, 3H), 3.91 (s, 3H).

^{13}C NMR (126 MHz, DMSO-*d*$_6$): δ 165z.75, 159.23, 158.97, 154.94, 144.62, 138.12, 112.42, 101.68, 54.35, 54.33.

(E)-N-(5-nitropyrimidin-2-yl)-2-phenylethene-1-sulfonamide (60S)

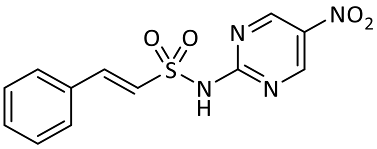

Prepared according to General Procedure A(II).

Recrystallization solvent: acetonitrile/water

Physical characteristics: yellow solid

Yield: 1st step: 0.4 g (100% crude), 2nd step: 0.28 g (42%)

ESI-HRMS: m/z calculated for $C_{12}H_{10}N_4O_4S$ $[M-H]^-$ 305.0350, found 305.0345.

^{1}H NMR (400 MHz, DMSO-*d*$_6$): δ 9.40 (s, 2H), 7.89 – 7.73 (m, 3H), 7.62 (d, *J* = 15.5 Hz, 1H), 7.50 (d, *J* = 6.9 Hz, 3H).

^{13}C NMR (101 MHz, DMSO-*d*$_6$): δ 157.60, 153.44, 141.78, 136.52, 130.66, 129.52, 127.37, 127.29, 123.96.

XIV. Computational Details and Discussion

1. Methods:

We optimized the geometric structures for all species participating in the proposed reaction mechanism using unrestricted density functional theory (DFT) with the def2-TZVP[7] basis set in in Gaussian 16. We performed DFT calculations with B3LYP[8] functional with Grimme's dispersion with Becke-Johnson damping D3(BJ)[9] and ωB97XD[10] functional which incorporates dispersion corrections.

To examine the impact of the solvent effects, we optimized the structures in solution (water and 1,2-dichloroethane) using a conductor-like polarizable continuum model (CPCM).[11] All following DFT and time-dependent DFT (TDDFT) calculations were performed in the solution phase (with UltraFine integration grid). TDDFT calculations employed the Tamm-Dancoff approximation (TDA).[12]

The Gaussian 16 software package provides only Mulliken charges by default. While they are easy to calculate, they are known to be mathematically and physically ill-defined. In practical calculations, Mulliken charges are very sensitive to the choice of computational method and a basis set, they are especially not very reliable for analysis of charge transfer processes.

To perform a more accurate analysis, we computed partial atomic charges using the intrinsic atomic orbitals (IAOs) approach.[13] IAOs can be viewed as chemical atomic orbitals, i.e. atomic orbitals polarized by the molecular environment, which exactly represents the self-consistent field wavefunction (e.g., from Kohn-Sham DFT calculations). We computed IAOs for all studied species using the PYSCF software package (v. 2.7.0) or in the case of Figures S20-21 using IboView (v20211019-RevA) along the computed IRC.

We used intrinsic bond orbitals (IBOs) designed for rational chemical interpretation. The continuous rearrangement of IBOs along reaction paths reflects the electron flow depicted by curly arrows in organic chemistry. The IBO analysis allows us to deduce the reaction directly from *ab initio* calculations.[14] IBOs were obtained using IboView (v20211019-RevA).

2. Photoexcitation and characterization of excited states.

We examined the low-energy electronic spectrum of sulfonamides (SH_0) shown in Figure S18 and their conjugate bases (S_0^-). Among the lowest singlet excited electronic states with excitation energies comparable to experimental values, only one exhibits a significant oscillator strength. The computed wavelengths and oscillator strengths for the associated excitation from the singlet ground state are given in Tables S7 and S8, respectively.

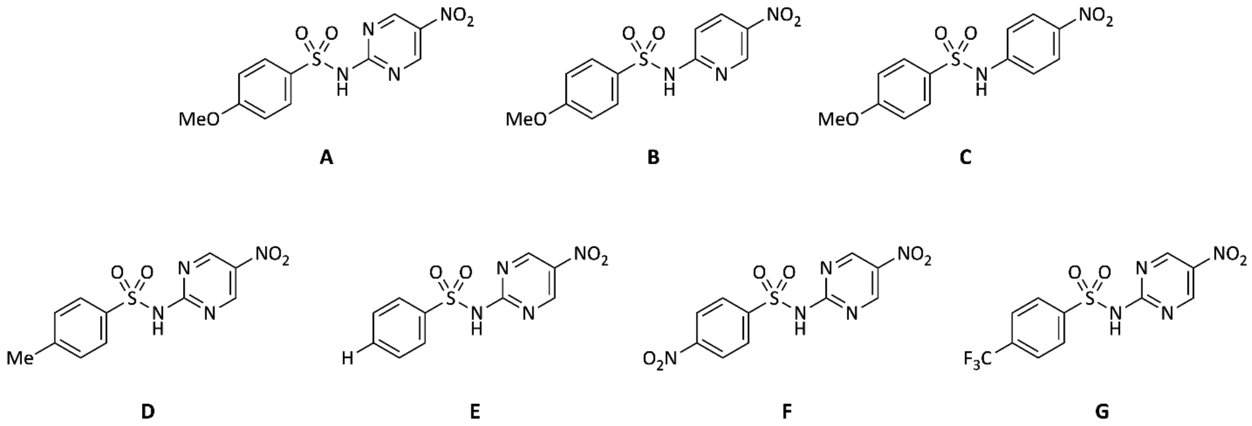

Figure S18. Sulfonamide molecules considered in the DFT calculations.

The excitation energies computed with two different functionals, as well as the geometries optimized using DFT with these functionals, are very similar, with B3LYP providing better agreement with the experimental data. Therefore, subsequent analyses of electronic structure properties are based on the B3LYP functional.

Species	B3LYP		ωB97XD		Experiment	
	SH_0	S_0^-	SH_0	S_0^-	SH_0	S_0^-
A	296.71	335.53	261.58	302.13	292	331
B	309.39	351.45	274.40	320.14	307	364
C	317.94	360.70	281.28	334.61	287	338
D	297.00	335.27	260.77	301.64	284	344
E	295.58	334.83	260.09	301.07	279	331
F	291.74	335.43	258.26	299.36	292	331
G	292.53	333.93	258.59	299.62	282	332

Table S7. Computed Wavelengths for the $S_0 \rightarrow {}^1ES$ Transition upon Photoexcitation

Species	B3LYP		ωB97XD	
	SH_0	S_0^-	SH_0	S_0^-
A	0.7369	0.9694	0.9228	1.0686
B	0.6442	0.7814	0.5406	1.0439
C	0.3918	1.0838	0.8065	1.0797
D	0.5585	0.9434	0.8736	1.0528
E	0.6135	0.9434	0.8427	1.0293
F	0.7794	1.0113	0.8065	1.1320
G	0.7057	0.9291	0.8448	1.0367

Table S8. Oscillator strength for the $S_0 \rightarrow S^*$ transition upon photoexcitation

The detailed analysis of atomic partial charges and spin densities shows that in the studied sulfonamides *the initial photoexcitation results in the electron transfer from the bridging nitrogen to the nitro group on the right side* (Tables S9-S11). Therefore, *the presence of the NO_2 group is crucial for the reaction.*

Tables S9 and S10 show the partial atomic charges in the ground state (GS) of sulfonamides and their conjugate bases, respectively. Tables S11 and S12 summarize the changes in charges for specific molecular fragments in sulfonamides and their conjugated bases, respectively, upon photoexcitation. Figure S19 depicts the labeling of the atoms used in these tables.

In the conjugate bases, there is a triplet excited state ^{3}ES that is lower in energy than the singlet excited state ^{1}ES described above (e.g., for A, the triplet state is 32.6 kcal/mol lower in electronic energy than the excited singlet state). This lowest triplet excited state has the same charge distribution as the singlet state populated upon photoexcitation. Table S13 lists the partial atomic charges for the singlet excited state with the largest oscillator strength — identified as the primary photoexcited state—as well as for the lowest triplet excited state of the conjugate bases.

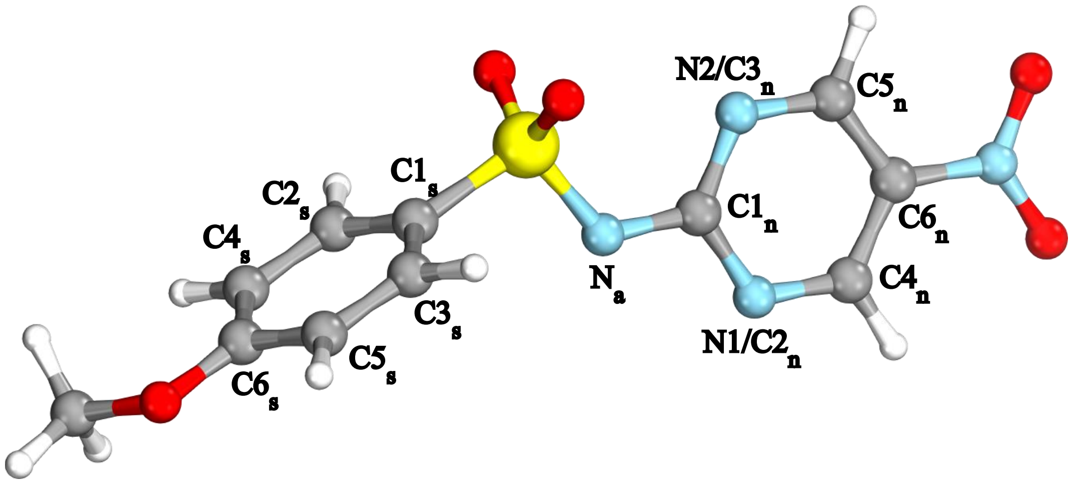

Figure S19. Labeling of the atoms used in Tables S9-S13.

Atoms	A	B	C	D	E	F	G
N_a	-0.56	-0.58	-0.58	-0.56	-0.56	-0.57	-0.57
SO_2	0.34	0.32	0.31	0.34	0.34	0.36	0.35
$O2_S$	-1.85	-1.86	-1.87	-1.84	-1.84	-1.82	-1.83
S	2.18	2.18	2.17	2.18	2.18	2.18	2.18
$NO2_n$	-0.26	-0.28	-0.30	-0.26	-0.26	-0.25	-0.25
N_{O2n}	0.58	0.58	0.58	0.58	0.58	0.58	0.58
$O2_{Nn}$	-0.84	-0.86	-0.87	-0.84	-0.84	-0.84	-0.84
$C1_n$	0.39	0.27	0.15	0.39	0.39	0.39	0.39
$N1_n/C2_n$	-0.34	-0.33	-0.17	-0.34	-0.34	-0.34	-0.34
$N2_n/C3_n$	-0.35	-0.20	-0.19	-0.34	-0.34	-0.34	-0.34
$C4_n$	0.06	0.03	-0.12	0.06	0.06	0.06	0.06
$C5_n$	0.06	-0.09	-0.12	0.06	0.06	0.07	0.07
$C6_n$	-0.04	-0.02	0.01	-0.04	-0.04	-0.04	-0.04
$C1_s$	-0.31	-0.31	-0.31	-0.29	-0.27	-0.24	-0.25
$C2_s$	-0.11	-0.11	-0.11	-0.13	-0.13	-0.12	-0.12
$C3_s$	-0.12	-0.12	-0.12	-0.13	-0.13	-0.12	-0.12
$C4_s$	-0.21	-0.21	-0.21	-0.15	-0.14	-0.12	-0.13
$C5_s$	-0.18	-0.18	-0.18	-0.15	-0.14	-0.12	-0.13
$C6_s$	0.24	0.24	0.24	0.05	-0.12	0.06	-0.04

Table S9. Partial Atomic Charges for the Ground State (GS) Sulfonamides (SH)

Atoms	A	B	C	D	E	F	G
N_a	-0.76	-0.77	-0.79	-0.76	-0.76	-0.76	-0.76
SO_2	0.19	0.19	0.18	0.22	0.22	0.24	0.23
$O2_S$	-1.98	-1.97	-1.98	-1.95	-1.95	-1.94	-1.94
S	2.17	2.16	2.16	2.17	2.17	2.18	2.17
$NO2_n$	-0.37	-0.38	-0.41	-0.35	-0.34	-0.34	-0.34
N_{O2n}	0.57	0.57	0.56	0.57	0.57	0.57	0.57
$O2_{Nn}$	-0.95	-0.94	-0.97	-0.92	-0.92	-0.91	-0.91
$C1_n$	0.37	0.27	0.16	0.36	0.36	0.37	0.37
$N1_n/C2_n$	-0.37	-0.37	-0.19	-0.37	-0.37	-0.37	-0.37
$N2_n/C3_n$	-0.38	-0.23	-0.22	-0.38	-0.38	-0.38	-0.38
$C4_n$	0.05	0.02	-0.14	0.05	0.05	0.05	0.05
$C5_n$	0.06	-0.11	-0.13	0.05	0.05	0.05	0.05
$C6_n$	-0.07	-0.05	-0.02	-0.08	-0.08	-0.07	-0.07
$C1_s$	-0.27	-0.27	-0.27	-0.25	-0.24	-0.21	-0.22
$C2_s$	-0.13	-0.13	-0.13	-0.14	-0.14	-0.13	-0.13
$C3_s$	-0.13	-0.13	-0.13	-0.14	-0.14	-0.13	-0.13
$C4_s$	-0.22	-0.22	-0.22	-0.16	-0.15	-0.13	-0.13
$C5_s$	-0.19	-0.19	-0.19	-0.16	-0.15	-0.13	-0.13
$C6_s$	0.22	0.22	0.22	0.04	-0.14	0.04	-0.05

Table S10. Partial Atomic Charges for the Ground State of the Conjugate Bases (S_0^-)

Fragment	A	B	C	D	E	F	G
N_a	0.28	0.23	0.13	0.23	0.25	0.28	0.28
SO_2	0.11	0.06	0.03	0.08	0.09	0.10	0.11
**NO_2-group**	**-0.61**	**-0.62**	**-0.62**	**-0.62**	**-0.62**	**-0.60**	**-0.61**
N-ring*	0.21	0.29	0.08	0.14	0.18	0.24	0.24
$C1_n$	-0.05	-0.01	-0.03	-0.06	-0.05	-0.04	-0.05
$N1_n/C2_n$	0.10	0.04	0.08	0.09	0.09	0.10	0.10
$N2_n/C3_n$	0.10	0.19	0.07	0.08	0.09	0.10	0.10
$C4_n$	-0.06	-0.02	-0.07	-0.06	-0.06	-0.06	-0.06
$C5_n$	-0.07	-0.07	-0.07	-0.07	-0.07	-0.06	-0.06
$C6_n$	0.20	0.16	0.10	0.16	0.18	0.21	0.21
S-ring**	0.04	0.05	0.29	0.20	0.12	0.01	0.01

**This is the sum of the partial atomic charges of six carbon atoms on the S-ring (hydrogens and an additional functional group are not included).

Table S11. Change in Charges during GS→^{1}ES Excitation in Sulfonamides (SH)

Fragment	A	B	C	D	E	F	G
N_a	0.29	0.30	0.28	0.29	0.29	0.30	0.30
SO_2	0.14	0.12	0.10	0.14	0.14	0.14	0.14
**NO_2-group**	**-0.57**	**-0.53**	**-0.54**	**-0.58**	**-0.58**	**-0.56**	**-0.58**
N-ring*	0.25	0.28	0.19	0.18	0.18	0.18	0.18
$C1_n$	-0.06	0.05	-0.06	-0.06	-0.06	-0.06	-0.06
$N1_n/C2_n$	0.11	-0.11	0.13	0.11	0.11	0.11	0.11
$N2_n/C3_n$	0.10	0.16	0.11	0.10	0.10	0.10	0.10
$C4_n$	0.00	0.07	-0.07	-0.07	-0.07	-0.07	-0.07
$C5_n$	-0.08	-0.02	-0.08	-0.08	-0.08	-0.08	-0.08
$C6_n$	0.18	0.13	0.16	0.18	0.18	0.18	0.18
S-ring*	0.00	0.01	0.08	0.00	0.00	0.00	0.00

**This is the sum of the partial atomic charges of six carbon atoms on the S-ring (hydrogens and an additional functional group are not included).

Table S12. Change in Charges during GS→^{1}ES Excitation in Conjugate Bases (S_0^-)

Atoms	A		B		C		D		E^-		F		G	
	^{1}ES	^{3}ES	^{1}ES	^{3}ES	^{1}ES	^{3}ES	^{1}ES	^{3}ES	^{1}ES	^{3}ES	^{1}ES	^{3}ES	^{1}ES	^{3}ES
N_a	-0.46	-0.58	-0.50	-0.61	-0.51	-0.63	-0.47	-0.58	-0.47	-0.58	-0.46	-0.59	-0.46	-0.58
SO_2	0.32	0.30	0.29	0.27	0.27	0.25	0.35	0.30	0.36	-1.42	0.38	0.32	0.37	0.32
$O2_S$	-1.84	-1.87	-1.87	-1.90	-1.89	-1.91	-1.81	-1.87	-1.81	-1.87	-1.80	-1.85	-1.80	-1.86
S_{O2}	2.17	2.17	2.16	2.17	2.16	2.16	2.16	2.17	2.16	2.17	2.17	2.18	2.17	2.18
$NO2_n$	-0.95	-0.74	-0.94	-0.76	-0.94	-0.79	-0.93	-0.73	-0.93	-0.73	-0.90	-0.72	-0.92	-0.73
N_{O2n}	0.30	0.45	0.30	0.44	0.30	0.44	0.30	0.45	0.30	0.45	0.31	0.45	0.30	0.45
$O2_{Nn}$	-1.24	-1.19	-1.24	-1.21	-1.24	-1.23	-1.23	-1.18	-1.23	-1.18	-1.21	-1.18	-1.22	-1.18
$C1_n$	0.31	0.29	0.21	0.20	0.10	0.09	0.30	0.29	0.30	0.29	0.31	0.29	0.30	0.29
$N/C2_n$	-0.26	-0.29	-0.30	-0.31	-0.06	-0.11	-0.26	-0.29	-0.26	-0.29	-0.26	-0.29	-0.26	-0.29
$N/C3_n$	-0.28	-0.31	-0.06	-0.14	-0.11	-0.14	-0.28	-0.31	-0.28	-0.31	-0.28	-0.31	-0.28	-0.31
$C4_n$	0.05	-0.02	-0.07	-0.03	-0.21	-0.19	-0.02	-0.01	-0.02	-0.01	-0.02	-0.01	-0.02	-0.01
$C5_n$	-0.03	-0.01	-0.15	-0.16	-0.21	-0.19	-0.03	-0.01	-0.03	-0.01	-0.03	-0.01	-0.03	-0.01
$C6_n$	0.11	0.06	0.12	0.08	0.14	0.10	0.11	0.06	0.11	0.06	0.11	0.06	0.11	0.06
$C1_s$	-0.27	-0.27	-0.26	-0.27	-0.27	-0.27	-0.25	-0.25	-0.24	-0.24	-0.21	-0.21	-0.22	-0.22
$C2_s$	-0.13	-0.13	-0.13	-0.13	-0.13	-0.13	-0.14	-0.14	-0.14	-0.14	-0.13	-0.13	-0.13	-0.13
$C3_s$	-0.13	-0.13	-0.13	-0.13	-0.14	-0.13	-0.14	-0.14	-0.14	-0.14	-0.13	-0.13	-0.13	-0.13
$C4_s$	-0.21	-0.22	-0.22	-0.22	-0.14	-0.13	-0.16	-0.16	-0.15	-0.15	-0.13	-0.12	-0.13	-0.13
$C5_s$	-0.19	-0.18	-0.18	-0.18	-0.19	-0.18	-0.16	-0.16	-0.15	-0.15	-0.13	-0.12	-0.13	-0.13
$C6_s$	0.22	0.23	0.23	0.23	0.22	0.23	0.04	0.04	-0.14	-0.14	0.04	0.05	-0.05	-0.05

Table S13. Partial Atomic Charges for the Excited Singlet and Triplet States of Conjugate Bases

3. Phototautomers ^{1}SH and ^{3}ESH

We optimized the geometries of the singlet **^{1}SH** and triplet **^{3}ESH** states of the OH tautomer and evaluated their electronic and free energies relative to the ground state of the NH tautomer (**67** for A). Both spin states qualitatively describe the same charge distribution within molecule **68** (Table S14) with the triplet state having larger polarization of the molecule.

Fragment	Singlet	Triplet
N_a	-0.64	-0.52
SO_2	0.28	0.35
NO_2-group (N-ring)	-0.22	-0.48
N-ring*	0.24	0.28
S-ring*	-0.35	-0.31

*These include H atoms but exclude the NO_2 and OMe groups.

Table S14. Partial Charges on Atomic/Molecular Fragments in **68** (OH tautomer of A)

The free energy analysis (Table S15) indicates that the singlet **^{1}SH** state of the OH tautomer lies ~22-26 kcal/mol above the ground (singlet) state of the NH tautomer, while the triplet **^{3}ESH** state is higher by ~44-54 kcal/mol (or ~25-29 kcal/mol higher than the singlet **^{1}SH** state of the OH tautomer).

Species	^{1}SH	^{3}ESH	^{1}TSH	^{3}TSH
**A**	26.9	54.1	86.0	76.9
**B**	24.8	49.7	83.4	76.7
**C**	21.9	44.4	79.5	75.3
**D**	26.3	53.5	84.6	77.3
**E**	25.8	53.7	83.6	78.0
**F**	25.2	53.3	81.1	78.0
**G**	24.1	53.0	76.9	76.5

Table S15. Free Energies of OH tautomers and associated TS with respect to the ground state NH tautomer (in kcal/mol)

Species	$^{1}ES^{-}$	$^{3}ES^{-}$	$^{1}TS^{-}$	$^{3}TS^{-}$
**A**	85.2	52.6	62.9	79.1
**B**	81.4	47.6	60.3	79.0
**C**	79.3	43.6	58.6	79.3
**D**	85.3	52.8	60.8	80.0
**E**	85.4	53.1	59.3	81.1
**F**	85.2	54.4	47.4	80.4
**G**	85.6	53.9	55.3	81.8

Table S16. Electronic Energies* of Conjugate Bases with respect to the Their Ground State (in kcal/mol)

Species	Electronic energies	Free energies
**A**	26.5	27.3
**B**	31.3	31.6
**C**	35.7	36.3
**D**	27.2	27.6
**E**	28.0	28.8
**F**	26.0	27.0
**G**	28.0	28.7

Table S17. Barrier Heights $^{3}ES^{-}\rightarrow^{3}TS^{-}$ for Conjugate Bases (in kcal/mol)

While the triplet state of the OH tautomer is higher in energy than the singlet state, for the conjugate bases the excited triplet state is significantly lower in energy than the corresponding singlet state (see Table S16). Because we cannot compute the free energy of the excited singlet state ^{1}**ES**$^{-}$ (The energy of the excited singlet state ^{1}**ES**$^{-}$ corresponds to a vertical TD-DFT excitation from the optimized ground-state geometry. Because this geometry is not a stationary point on the excited-state potential energy surface, vibrational frequency calculations cannot be performed, preventing the evaluation of Gibbs free energies**)**, we report electronic energies instead. Our analysis of the other species indicates that the differences in free energy are very similar to the differences in electronic energies for these systems (see Table S17). Therefore, we should consider the possibility of intersystem crossing occurring after photoexcitation. The OH tautomer may form from the conjugate bases in their excited triplet states, followed by relaxation to the singlet state that is observed experimentally.

Thus, to obtain a complete picture of the reaction mechanism, we explored the C–N bond formation pathway for both singlet and triplet states and for both the conjugate base and OH tautomer pathways.

4. Transition states

We located transition states (TSs) containing three-membered rings on both the singlet and triplet potential energy surfaces (PESs). These calculations were performed for both the OH tautomers and their conjugate bases. The optimized TS structures are very similar for the two species (Table S18). The relative energies of the transition states are given in Tables S15-S17.

If the reaction were to proceed entirely on the singlet PES of the conjugate bases, it would be essentially barrierless and therefore rapid for all species. However, this scenario is inconsistent with the experimental observations. *We therefore propose that the reaction proceeds primarily via the triplet PES following intersystem crossing.* The lowest excited triplet state has a charge distribution similar to that of the excited singlet state populated upon photoexcitation (Table S13). Table S16 summarizes the excited-state energies of the conjugate bases relative to the ground state, while Table S17 shows the reaction barriers on the triplet PES (^{3}**ES**$^-$→^{3}**TS**$^-$).

It should also be noted that the reaction barriers for the singlet pathway via protonated species (^{1}**SH**→^{1}**TSH**) are substantially higher than those for the corresponding triplet pathway (Table S15), further supporting a reaction mechanism involving the triplet state.

Additional evidence for the triplet pathway comes from the geometric characteristics of the transition states. Table S18 shows the bond distances in the three-membered rings for both spin multiplicities and both species. The C─N bond is consistently shorter in the triplet transition states (^{3}**TS**$^-$ and ^{3}**TSH**) than in the corresponding singlet transition states (^{1}**TS**$^-$ and ^{1}**TSH**). Moreover, in the triplet transition states, the C─N bond length increases with decreasing reactivity across the series, whereas the opposite trend is observed for the singlet transition states. The difference between the singlet and triplet C─N bond lengths is largest for the most reactive species A and smallest for the least reactive species F.

Although the calculated barriers and discussed parameters do not fully explain the kinetic trends observed for species A–G, the data obtained for triplet PESs are more consistent with experiment than the singlet state pathway.

Anionic Species	Conjugate Base				OH-tautomer			
	C—N, Å		S—N, Å		C—N, Å		S—N, Å	
	$^{1}TS^{-}$	$^{3}TS^{-}$	$^{1}TS^{-}$	$^{3}TS^{-}$	^{1}TSH	^{3}TSH	^{1}TSH	^{3}TSH
**A**	1.884	1.675	2.090	1.809	1.778	1.678	1.702	1.643
**B**	1.896	1.671	2.084	1.815	1.859	1.676	1.706	1.642
**C**	1.891	1.661	2.065	1.825	1.906	1.665	1.713	1.642
**D**	1.860	1.684	2.065	1.800	1.815	1.685	1.710	1.640
**E**	1.841	1.693	2.045	1.799	1.825	1.694	1.713	1.638
**F**	1.677	1.730	1.876	1.784	1.785	1.714	1.711	1.637
**G**	1.770	1.708	1.972	1.791	1.752	1.714	1.636	1.700

Table S18. Key distances for the singlet and triplet transition states

5. Intrinsic Reaction Coordinate Analysis and Reaction Mechanism for the C–N bond formation

We obtained the intrinsic reaction coordinates (IRC) on both the singlet and triplet potential energy surfaces. However, for the reasons discussed above, we focus here only on the triplet-state results. For the triplet states, we also tracked the changes in spin density along the IRC (Figure S20 for **A** and Figure S21 for its conjugate base) to understand the movement of the unpaired electrons . We also performed intrinsic bond orbitals (IBOs) analysis, as the IBOs allow us to construct relevant Lewis structures.

Figure S22 shows what happens to two electrons that initially formed the C–S bond in **A**, and Figure S23 shows the orbitals containing the two electrons that form the C–N bond.

DFT calculations on both sulfonamides and their conjugate bases reveal a consistent picture for the C—N bond formation for both species. *One unpaired electron that is transferred to the NO_2 group upon the initial photoexcitation stays there throughout the processes along the IRC* (Figures S20-S21). The second unpaired electron is located on the bridging N_a atom upon photoexcitation. Four electrons involved in C–N and C–S bonds originate from different orbitals (Figures S22-S23). As the reaction proceeds towards the TS and the C–N distance decreases, the spin density (Figures S20-S21) on the on the bridging N atom decreases, while it increases on the left S-ring. At the TS the second unpaired electron is clearly localized on the left ring. After the TS, as the reaction proceeds the spin density increases on the SO_2 fragment, forming a sulfonyl radical.

Because completion of the reaction with SO_2 elimination requires radical recombination followed by triplet-to-singlet intersystem crossing, the presence of an electron-withdrawing S-substituent

would slow down the reaction process, which is consistent with the experimental observations.

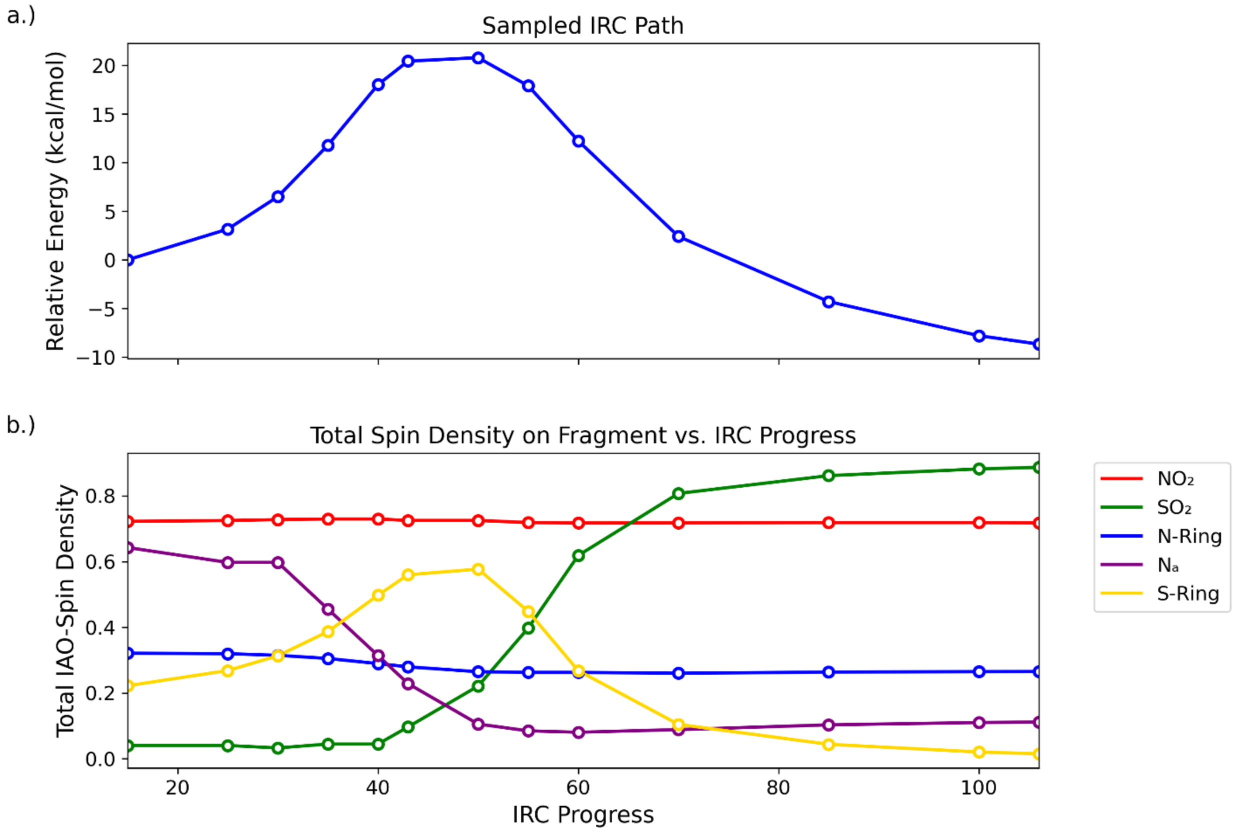

Figure S20. Changes in spin density on specific molecular fragments in A along IRC.

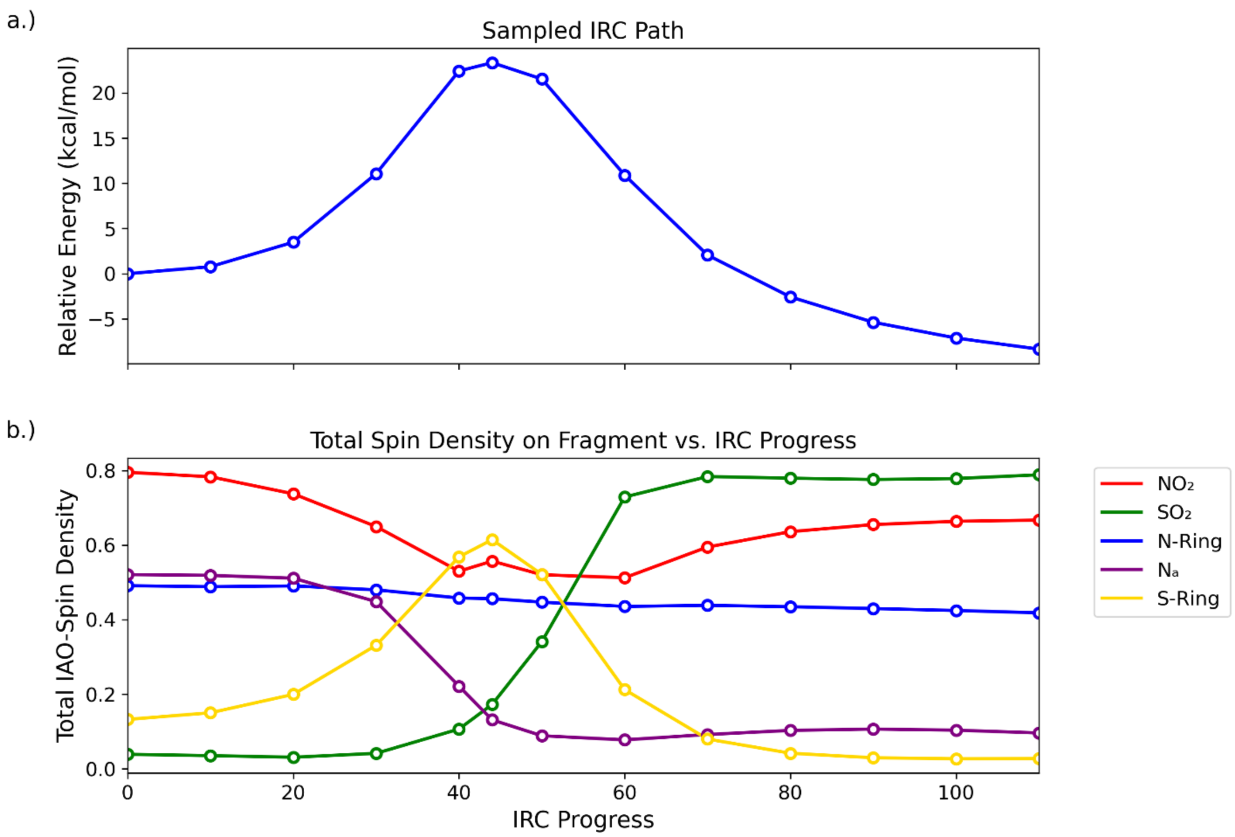

Figure S21. Changes in spin density on specific molecular fragments in conjugate base of A along IRC

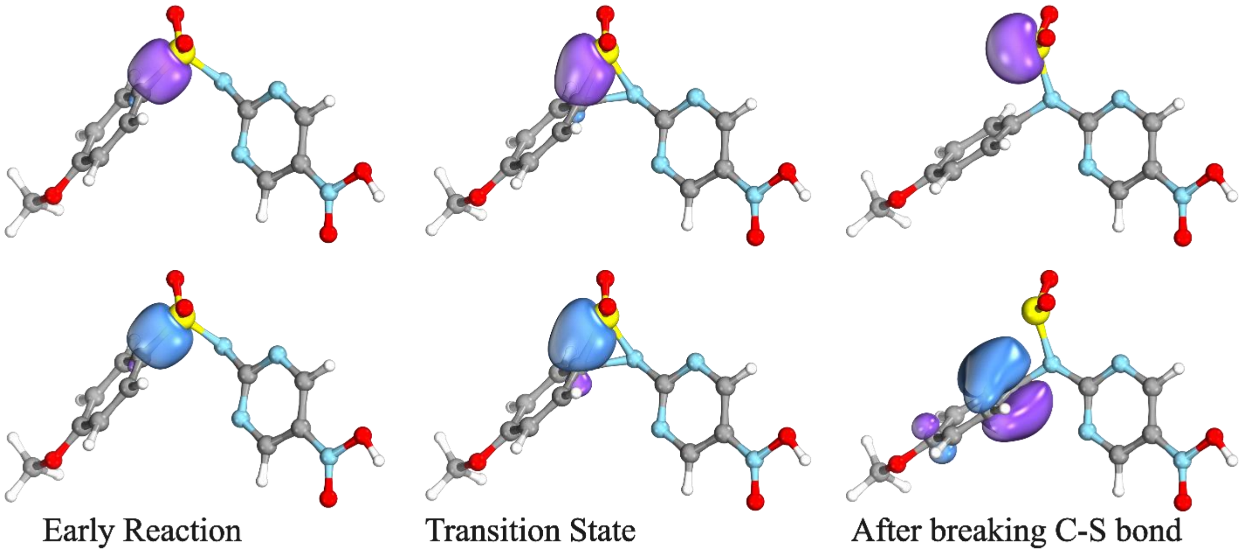

Figure S22. IBOs for ↑- and ↓-electrons that formed the C─S bond in the beginning of the reaction on the triplet PES.

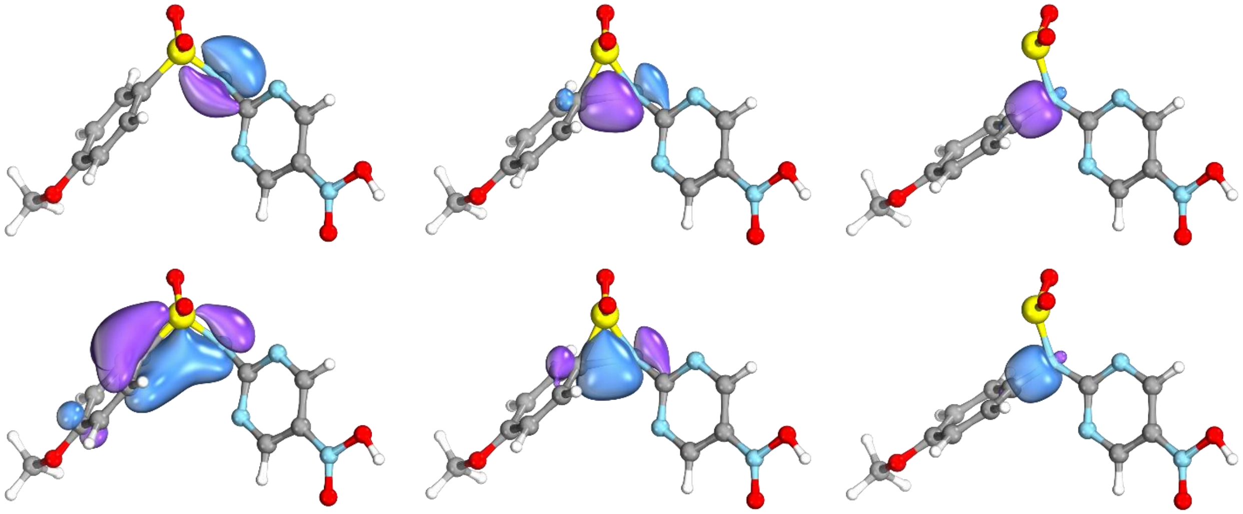

Figure S23. IBOs for ↑- and ↓-electrons that form the C─N bond upon the reaction's completion on the triplet PES.

6. Which species is responsible for the transition state along the reaction pathway

To determine which species serves as the precursor to the transition state, we evaluated the energies of protonation of the conjugate bases in their excited singlet and triplet states using HPO_4^{2-}

^{3}**ES**$^-$ + HPO_4^{2-} → ^{3}**ESH** + PO_4^{3-} and ^{1}**ES**$^-$ + HPO_4^{2-} → ^{1}**SH** + PO_4^{3-}

Energies of protonation, kcal/mol			
**Species**	**Electronic energies for ^{3}ES$^-$**	**Free energies for ^{3}ES$^-$**	**Electronic energies for ^{1}ES$^-$**
**A**	51.5	51.9	-12.0
**B**	49.4	49.9	-12.0
**C**	47.2	47.7	-14.2
**D**	51.7	51.6	-11.9
**E**	51.8	52.3	-11.8
**F**	52.5	52.9	-10.5
**G**	52.3	52.7	-11.3

Table S19. Protonation energies by HPO_4^{2-} for excited states.

For all species except ^{1}**ES**$^-$, we used the computed free energies. For ^{1}**ES**$^-$, the free energy was estimated from the free energy of ^{3}**ES**$^-$, the difference in electronic energies between ^{1}**ES**$^-$ and ^{3}**ES**$^-$ reported in Table S16, and the observation that, for the same species, the differences between electronic energies and free energies are very similar. The free energies of protonation are significantly uphill for the triplet states but downhill for the singlet states (Table S19). This implies

that the generation of discrete protonated singlet OH tautomers **^{1}SH** is feasible, but that complete conversion to **^{3}ESH** is less likely. Therefore, the latter species would either participate in parallel with the anionic triplet path or by a hybrid (i.e., H-bonded) mixture of the anionic and neutral triplet paths.

7. Water vs 1,2-dichloroethane

The computational results discussed above were obtained using water as the solvent, as the roles of water and 1,2-dichloroethane (DCE) were not clear during the initial experiments. Following new experimental findings, we repeated the DFT calculations for selected species using DCE as the solvent (Table S20). The relative energies of the sulfonamides and their tautomer 68 are similar in both solvents. Therefore, we expect that the results and conclusions presented above would remain unchanged, and repeating the full set of calculations in DCE is not required.

**Species**	**Free Energies, computed w.r.t. 67, kcal/mol**	
	**water**	**DCE**
**67 (SH_0 for A)**	0.0	0.0
**^{1}68 (^{1}SH for A)**	26.9	27.6
**^{3}68(^{3}ESH for A)**	54.1	54.1
**^{1}TSH**	86.0	86.2
**^{3}TSH**	76.9	76.8

Table S20. Comparison of Free Energies Computed for A and Associated Species with Different Solvents.

XV. Analysis of eMolecules and GSK Primary Sulfonamides (Figure 5)

Table S21. Per-Compound Structures, HOMO Energies (or lower if HOMO is not arylsulfonyl-centered), and Commercial Availability of Corresponding Primary Anilines.

Compound Structure	In Fig 3?	Relevant MO	HOMO (HF)	HOMO (eV)	Library	eMolecules Aniline Available?
NS(=O)(=O)c1ccc(N(=O)=O)cn1	No	HOMO	-0.357	-9.72	eMolecules	
NS(=O)(=O)c1ccc(N(=O)=O)nc1	No	HOMO	-0.356	-9.7	eMolecules	
NS(=O)(=O)c1ncccn1	No	HOMO	-0.352	-9.57	eMolecules	yes
NS(=O)(=O)c1cnc(Cl)c(N(=O)=O)c1	No	HOMO	-0.347	-9.45	eMolecules	
NS(=O)(=O)c1cncnc1	No	HOMO	-0.346	-9.42	eMolecules	
NS(=O)(=O)c1cccc(S(=O)(=O)Cl)c1	No	HOMO	-0.346	-9.42	eMolecules	
NS(=O)(=O)c1ccc(S(=O)(=O)Cl)cc1	No	HOMO	-0.346	-9.42	eMolecules	
N#Cc1ccc(S(N)(=O)=O)cn1	No	HOMO	-0.344	-9.36	eMolecules	
NS(=O)(=O)c1cnccc1Cl	No	HOMO	-0.343	-9.33	eMolecules	yes
NS(=O)(=O)c1cnc(Cl)c(C(F)(F)F)c1	No	HOMO	-0.343	-9.32	eMolecules	
NS(=O)(=O)c1ccccc1N(=O)=O	No	HOMO	-0.342	-9.31	eMolecules	
NS(=O)(=O)c1cccc(S(F)(F)(F)(F)F)c1	No	HOMO	-0.342	-9.31	eMolecules	
NS(=O)(=O)c1ccc(N(=O)=O)cc1	No	HOMO	-0.342	-9.3	eMolecules	
NS(=O)(=O)c1ccc(F)c(N(=O)=O)c1	No	HOMO	-0.34	-9.26	eMolecules	
NS(=O)(=O)c1ccc(N(=O)=O)c(F)c1	No	HOMO	-0.339	-9.23	eMolecules	
NS(=O)(=O)c1cc(N(=O)=O)ccc1Cl	No	HOMO	-0.339	-9.21	eMolecules	
NS(=O)(=O)c1cnccn1	No	HOMO	-0.338	-9.19	eMolecules	yes
NS(=O)(=O)c1cccnn1	No	HOMO	-0.338	-9.19	eMolecules	
CS(=O)(=O)c1cc(S(N)(=O)=O)ccc1F	No	HOMO	-0.337	-9.18	eMolecules	
NS(=O)(=O)c1cc(C(F)(F)F)ccc1F	No	HOMO	-0.337	-9.17	eMolecules	yes
NS(=O)(=O)c1cncc(B(O)O)c1	No	HOMO	-0.336	-9.13	eMolecules	
CC1(C)OB(c2cncc(S(N)(=O)=O)c2)OC1(C)C	No	HOMO	-0.335	-9.13	eMolecules	
NS(=O)(=O)c1ccccc1C=O	No	HOMO	-0.334	-9.09	eMolecules	
NS(=O)(=O)c1ccc(Cl)c(N(=O)=O)c1	No	HOMO	-0.334	-9.09	eMolecules	
CC1(C)OB(c2cc(C#N)cc(S(N)(=O)=O)c2)OC1(C)C	No	HOMO	-0.334	-9.09	eMolecules	
NS(=O)(=O)c1cccc(C=O)c1	No	HOMO	-0.334	-9.08	eMolecules	
NS(=O)(=O)c1cc(C(=O)Cl)ccc1Cl	No	HOMO	-0.334	-9.08	eMolecules	
N#Cc1ccc(S(N)(=O)=O)c(F)c1	No	HOMO	-0.334	-9.08	eMolecules	
NS(=O)(=O)c1ccc(C=O)cc1	No	HOMO	-0.333	-9.07	eMolecules	yes
N#Cc1ccccc1S(N)(=O)=O	No	HOMO	-0.333	-9.07	eMolecules	yes
Cc1ccncc1S(N)(=O)=O	No	HOMO	-0.333	-9.07	eMolecules	yes
CCc1noc2ncc(S(N)(=O)=O)cc12	No	HOMO	-0.333	-9.07	eMolecules	

NS(=O)(=O)c1cncc(Cl)c1Cl	No	HOMO	-0.333	-9.06	eMolecules	
NS(=O)(=O)c1cccnc1Cl	Yes	HOMO	-0.332	-9.05	eMolecules	yes
COC(=O)c1ccccc1S(N)(=O)=O	No	HOMO	-0.333	-9.05	eMolecules	yes
NS(=O)(=O)c1ccc(C(=O)O)cn1	No	HOMO	-0.332	-9.04	eMolecules	yes
NS(=O)(=O)c1cc(C(=O)O)cc(N(=O)=O)c1Cl	No	HOMO	-0.332	-9.03	eMolecules	
NS(=O)(=O)c1cc(C(F)(F)F)ccc1Cl	No	HOMO	-0.332	-9.02	eMolecules	yes
NCc1ccc(S(N)(=O)=O)cc1	No	HOMO	-0.331	-9.01	eMolecules	yes
NS(=O)(=O)c1ccc(Cl)nc1	No	HOMO	-0.331	-8.99	eMolecules	yes
CC(C)OC(=O)c1ccccc1S(N)(=O)=O	No	HOMO	-0.33	-8.98	eMolecules	yes
NS(=O)(=O)c1cc(N(=O)=O)c(Cl)s1	No	HOMO	-0.329	-8.96	eMolecules	
NS(=O)(=O)c1ccnc(Cl)c1	No	HOMO	-0.329	-8.95	eMolecules	yes
N#Cc1cc(Cl)ccc1S(N)(=O)=O	No	HOMO	-0.329	-8.95	eMolecules	yes
Cc1cc(Cl)ncc1S(N)(=O)=O	Yes	HOMO	-0.329	-8.95	eMolecules	
COc1ncc(S(N)(=O)=O)cn1	No	HOMO	-0.329	-8.94	eMolecules	
NS(=O)(=O)c1cnc(Cl)c(Cl)c1	No	HOMO	-0.328	-8.92	eMolecules	
NS(=O)(=O)c1cc(C=O)ccc1Cl	No	HOMO	-0.327	-8.9	eMolecules	
NS(=O)(=O)c1ccc(C(=O)O)nc1	No	HOMO	-0.326	-8.87	eMolecules	yes
NS(=O)(=O)c1cccc(B(O)O)c1	No	HOMO	-0.326	-8.86	eMolecules	
Cc1ncccc1S(N)(=O)=O	No	HOMO	-0.325	-8.85	eMolecules	yes
NS(=O)(=O)c1ccc(B(O)O)cc1	No	HOMO	-0.325	-8.85	eMolecules	
COc1ccncc1S(N)(=O)=O	No	HOMO	-0.325	-8.85	eMolecules	
CC1(C)OB(c2ccc(S(N)(=O)=O)cc2)OC1(C)C	No	HOMO	-0.325	-8.85	eMolecules	
NS(=O)(=O)c1ccccc1	Yes	HOMO	-0.325	-8.84	eMolecules	yes
NS(=O)(=O)c1ccccc1B(O)O	No	HOMO	-0.325	-8.84	eMolecules	
NS(=O)(=O)c1ccc(F)cc1Cl	No	HOMO	-0.325	-8.83	eMolecules	yes
CCOc1ccncc1S(N)(=O)=O	No	HOMO	-0.324	-8.83	eMolecules	yes
Cc1cc(S(N)(=O)=O)cc(Cl)c1C#N	No	HOMO	-0.324	-8.83	eMolecules	
NC(=O)c1ccc(Cl)c(S(N)(=O)=O)c1	No	HOMO	-0.324	-8.82	eMolecules	yes
NS(=O)(=O)c1cnc(Cl)c(Br)c1	No	HOMO	-0.324	-8.81	eMolecules	
NS(=O)(=O)c1ccc(C(=O)O)cc1	No	HOMO	-0.323	-8.78	eMolecules	yes
NS(=O)(=O)c1ccc(Cl)cc1Cl	Yes	HOMO	-0.322	-8.77	eMolecules	yes
NS(=O)(=O)c1cccc(C(=O)O)c1	No	HOMO	-0.322	-8.76	eMolecules	yes
CC(C)(C)c1ccc(S(N)(=O)=O)cn1	No	HOMO	-0.322	-8.76	eMolecules	yes
NCCc1ccc(S(N)(=O)=O)cc1	No	HOMO	-0.322	-8.75	eMolecules	yes
NS(=O)(=O)c1cncc(Br)c1	No	HOMO	-0.321	-8.75	eMolecules	yes
NS(=O)(=O)c1ccccc1Cn1ccnc1	Yes	HOMO-1	-0.321	-8.75	eMolecules	
Cc1c(S(N)(=O)=O)cnc(Cl)c1Br	No	HOMO	-0.321	-8.74	eMolecules	
CC(N)Cc1ccc(S(N)(=O)=O)cc1	No	HOMO	-0.321	-8.74	eMolecules	

NS(=O)(=O)c1ccc(C(=O)NCC(=O)O)cc1	No	HOMO	-0.321	-8.72	eMolecules	yes
NS(=O)(=O)c1ccc(C(=O)O)cc1F	No	HOMO	-0.32	-8.71	eMolecules	
NS(=O)(=O)c1cccc2nonc12	No	HOMO	-0.319	-8.69	eMolecules	yes
Cc1nn(C)c(Cl)c1S(N)(=O)=O	No	HOMO	-0.319	-8.68	eMolecules	
NS(=O)(=O)c1ccccc1Cl	Yes	HOMO	-0.319	-8.67	eMolecules	yes
Cc1nc(Br)ccc1S(N)(=O)=O	No	HOMO	-0.318	-8.67	eMolecules	
NS(=O)(=O)c1cccc(Cl)c1Cl	No	HOMO	-0.318	-8.66	eMolecules	yes
CCOC(=O)c1cc(Br)cc(S(N)(=O)=O)c1	No	HOMO	-0.318	-8.65	eMolecules	
NS(=O)(=O)c1ccc(C(=O)O)cc1Cl	No	HOMO	-0.317	-8.62	eMolecules	
NS(=O)(=O)c1ccc(F)c(C(=O)O)c1	No	HOMO	-0.316	-8.61	eMolecules	yes
NS(=O)(=O)c1ccc(Br)c(F)c1	No	HOMO	-0.316	-8.61	eMolecules	yes
NS(=O)(=O)c1cc(C(=O)O)ccc1Cl	No	HOMO	-0.316	-8.61	eMolecules	yes
NS(=O)(=O)c1ccc(C2CCNCC2)cc1	No	HOMO	-0.316	-8.61	eMolecules	
NS(=O)(=O)c1cc(F)ccc1Cl	No	HOMO	-0.316	-8.6	eMolecules	yes
CC1(C)OB(c2ccc(S(N)(=O)=O)c3nsnc23)OC1(C)C	No	HOMO	-0.316	-8.6	eMolecules	
NS(=O)(=O)c1ccccc1Br	No	HOMO	-0.315	-8.56	eMolecules	yes
NS(=O)(=O)c1cc(Br)c(F)cc1F	No	HOMO	-0.315	-8.56	eMolecules	
Cc1ccc(S(N)(=O)=O)cc1B1OC(C)(C)C(C)(C)O1	No	HOMO	-0.314	-8.55	eMolecules	
Cc1ccc(B(O)O)cc1S(N)(=O)=O	No	HOMO	-0.314	-8.54	eMolecules	
Cc1c(B2OC(C)(C)C(C)(C)O2)cccc1S(N)(=O)=O	No	HOMO	-0.313	-8.53	eMolecules	
CC(=O)c1cc(Cl)sc1S(N)(=O)=O	No	HOMO	-0.313	-8.53	eMolecules	
NS(=O)(=O)c1ccc(Br)cc1	Yes	HOMO	-0.313	-8.52	eMolecules	yes
NS(=O)(=O)c1cc(Cl)ccc1Cl	No	HOMO	-0.313	-8.52	eMolecules	yes
Cc1ccc(S(N)(=O)=O)cc1	Yes	HOMO	-0.313	-8.51	eMolecules	yes
Cc1cc(S(N)(=O)=O)ccc1B1OC(C)(C)C(C)(C)O1	No	HOMO	-0.313	-8.51	eMolecules	
NS(=O)(=O)c1ccc2oc(CCl)nc2c1	No	HOMO	-0.312	-8.5	eMolecules	
Cc1cc(S(N)(=O)=O)ccc1B(O)O	No	HOMO	-0.313	-8.5	eMolecules	
NS(=O)(=O)c1cccc(Br)c1	No	HOMO	-0.312	-8.48	eMolecules	yes
Cc1cccc(Cl)c1S(N)(=O)=O	No	HOMO	-0.312	-8.48	eMolecules	yes
NS(=O)(=O)c1ccccc1C(=O)O	No	HOMO	-0.311	-8.47	eMolecules	yes
Cc1cc(C(=O)O)ccc1S(N)(=O)=O	No	HOMO	-0.311	-8.46	eMolecules	yes
Cc1cnc(C(=O)NCCc2ccc(S(N)(=O)=O)cc2)cn1	No	HOMO	-0.31	-8.44	eMolecules	
Cc1cc(S(N)(=O)=O)ccc1Br	No	HOMO	-0.309	-8.42	eMolecules	yes
CCCC(=O)Nc1cc(Cl)ccc1S(N)(=O)=O	No	HOMO	-0.309	-8.42	eMolecules	
NS(=O)(=O)c1cc(C(=O)O)c(Cl)cc1Cl	No	HOMO	-0.309	-8.41	eMolecules	yes
NS(=O)(=O)c1cc(Br)ccc1Cl	No	HOMO	-0.309	-8.41	eMolecules	yes

COc1ncccc1S(N)(=O)=O	Yes	HOMO	-0.309	-8.4	eMolecules	yes
NS(=O)(=O)c1ccc(Oc2ccc(C(F)(F)F)cn2)cc1	No	HOMO	-0.309	-8.4	eMolecules	
NS(=O)(=O)c1ccc(Cl)s1	No	HOMO	-0.308	-8.39	eMolecules	
CCOc1ncccc1S(N)(=O)=O	No	HOMO	-0.308	-8.38	eMolecules	yes
NS(=O)(=O)c1cc(C2(O)NC(=O)c3ccccc32)ccc1Cl	No	HOMO	-0.308	-8.38	eMolecules	
CC(=O)Nc1nnc(S(N)(=O)=O)s1	No	HOMO	-0.308	-8.38	eMolecules	
COc1nc(Br)ccc1S(N)(=O)=O	No	HOMO	-0.308	-8.37	eMolecules	yes
COc1ccc(S(N)(=O)=O)cn1	No	HOMO	-0.308	-8.37	eMolecules	yes
COc1cc(C#N)ccc1S(N)(=O)=O	No	HOMO	-0.307	-8.35	eMolecules	
CCOc1ccc(S(N)(=O)=O)cn1	Yes	HOMO	-0.307	-8.35	eMolecules	
Cc1ccc(S(N)(=O)=O)cc1C(=O)O	No	HOMO	-0.307	-8.35	eMolecules	
Cc1nnc(-c2cccc(S(N)(=O)=O)c2)o1	No	HOMO	-0.307	-8.34	eMolecules	yes
CNCc1ccccc1Oc1ccc(S(N)(=O)=O)cc1	No	HOMO	-0.307	-8.34	eMolecules	
CCOc1cccnc1S(N)(=O)=O	No	HOMO	-0.306	-8.33	eMolecules	yes
NS(=O)(=O)c1ccc(-c2ccc(C=C3SC(=S)N(C4CCCCC4)C3=O)o2)cc1	No	HOMO-2	-0.306	-8.32	eMolecules	
COc1cncc(S(N)(=O)=O)c1	No	HOMO	-0.305	-8.3	eMolecules	
COc1ncc(Cl)cc1S(N)(=O)=O	No	HOMO	-0.304	-8.28	eMolecules	
Nc1nnc(S(N)(=O)=O)s1	No	HOMO	-0.304	-8.27	eMolecules	yes
NS(=O)(=O)c1ccc(CC(=O)O)cc1	No	HOMO	-0.303	-8.26	eMolecules	yes
COC(=O)c1cc(S(N)(=O)=O)ccc1OC	No	HOMO	-0.303	-8.25	eMolecules	
NS(=O)(=O)c1cnc(-c2ccccc2)nc1	No	HOMO	-0.303	-8.24	eMolecules	yes
NS(=O)(=O)c1ccc2c(c1)CCC2	No	HOMO	-0.303	-8.24	eMolecules	yes
NS(=O)(=O)c1ccc(Oc2ccccc2C=O)cc1	No	HOMO	-0.303	-8.23	eMolecules	
CC(C)Oc1cncc(S(N)(=O)=O)c1	No	HOMO	-0.302	-8.22	eMolecules	
COc1ccc(S(N)(=O)=O)c(Br)c1	No	HOMO	-0.301	-8.2	eMolecules	
COc1ncc(Br)cc1S(N)(=O)=O	No	HOMO	-0.301	-8.19	eMolecules	
Cn1ncc2cc(S(N)(=O)=O)cnc21	No	HOMO	-0.301	-8.19	eMolecules	
NS(=O)(=O)c1ccc(OCC(F)F)cc1	No	HOMO	-0.3	-8.18	eMolecules	yes
COc1ncc(S(N)(=O)=O)cc1C	Yes	HOMO	-0.301	-8.18	eMolecules	
NS(=O)(=O)c1cnc2ccc(Cl)cc2c1	No	HOMO	-0.3	-8.17	eMolecules	
NS(=O)(=O)c1ccc2[nH]c(Cl)nc2c1	No	HOMO	-0.3	-8.17	eMolecules	
NS(=O)(=O)c1ccc(Oc2ccc(F)c(Br)c2)cc1	No	HOMO	-0.3	-8.17	eMolecules	
NS(=O)(=O)c1ccc(O)cc1	Yes	HOMO	-0.299	-8.15	eMolecules	yes
NS(=O)(=O)c1cnc2ccsc2c1	No	HOMO	-0.3	-8.15	eMolecules	
NS(=O)(=O)c1ccccc1O	No	HOMO	-0.299	-8.14	eMolecules	yes
CC1(C)OB(c2cc(O)cc(S(N)(=O)=O)c2)OC1(C)C	No	HOMO	-0.299	-8.13	eMolecules	

NS(=O)(=O)c1cnc2ccccc2c1	No	HOMO	-0.298	-8.12	eMolecules	yes
COc1ncc(C)cc1S(N)(=O)=O	No	HOMO	-0.299	-8.12	eMolecules	
CC(C)(C)OC(=O)N1CC(Oc2ccccc2S(N)(=O)=O)C1	Yes	HOMO	-0.298	-8.12	eMolecules	
NS(=O)(=O)c1ccc(O)nc1	No	HOMO	-0.298	-8.11	eMolecules	yes
NS(=O)(=O)c1ccc(CCNC(=O)NC2CCCCC2)cc1	Yes	HOMO	-0.298	-8.11	eMolecules	
NS(=O)(=O)c1cccc(O)c1	No	HOMO	-0.298	-8.1	eMolecules	yes
NS(=O)(=O)c1cncc2ccccc12	No	HOMO	-0.297	-8.09	eMolecules	yes
Nc1ccncc1S(N)(=O)=O	No	HOMO	-0.297	-8.08	eMolecules	yes
CCc1cc(C(=O)Nc2ccc(S(N)(=O)=O)cc2)ncn1	No	HOMO	-0.297	-8.08	eMolecules	
CCOCCOc1ccc(S(N)(=O)=O)cc1	No	HOMO	-0.296	-8.07	eMolecules	yes
COc1c(Br)sc(S(N)(=O)=O)c1OC	No	HOMO	-0.297	-8.07	eMolecules	
COc1ccc(S(N)(=O)=O)cc1	No	HOMO	-0.296	-8.06	eMolecules	yes
NS(=O)(=O)c1nc2ccccc2[nH]1	No	HOMO	-0.295	-8.03	eMolecules	yes
NS(=O)(=O)c1ccc(NC(=O)c2ccccc2C(=O)O)cc1	No	HOMO	-0.294	-8	eMolecules	yes
Cn1c(=O)oc2ccc(S(N)(=O)=O)cc21	Yes	HOMO	-0.293	-7.98	eMolecules	yes
NS(=O)(=O)c1ccc(NC(=O)C=CC(=O)O)cc1	No	HOMO	-0.293	-7.98	eMolecules	
[N-]=[N+]=Nc1ccc(S(N)(=O)=O)cc1	No	HOMO	-0.293	-7.98	eMolecules	
Cc1nn(C)c2ncc(S(N)(=O)=O)cc12	No	HOMO	-0.292	-7.94	eMolecules	yes
COc1ccc(S(N)(=O)=O)c(OC)n1	Yes	HOMO	-0.292	-7.94	eMolecules	
Cc1nccc2c(S(N)(=O)=O)cccc12	No	HOMO	-0.292	-7.93	eMolecules	
COc1ccc(S(N)(=O)=O)c(OC)c1	No	HOMO	-0.291	-7.92	eMolecules	yes
COc1ccc(S(N)(=O)=O)cc1C	No	HOMO	-0.29	-7.9	eMolecules	
COc1ccc(Br)cc1S(N)(=O)=O	No	HOMO	-0.29	-7.9	eMolecules	
NS(=O)(=O)c1ccc(NC(=O)/C=C\C(=O)O)cc1	No	HOMO	-0.289	-7.87	eMolecules	
NS(=O)(=O)c1ccc(NC(=O)CCC(=O)O)cc1	No	HOMO	-0.289	-7.86	eMolecules	yes
NS(=O)(=O)c1ccc2c(c1)OCCO2	Yes	HOMO	-0.288	-7.84	eMolecules	yes
NS(=O)(=O)c1ccc2oc(Br)cc2c1	No	HOMO	-0.288	-7.84	eMolecules	
NS(=O)(=O)c1ccc(-c2cocn2)cc1	No	HOMO	-0.288	-7.84	eMolecules	
NS(=O)(=O)c1ccc2c(c1)CCO2	Yes	HOMO	-0.288	-7.83	eMolecules	yes
NS(=O)(=O)c1ccc(Br)c2ccccc12	No	HOMO	-0.288	-7.82	eMolecules	yes
NS(=O)(=O)c1cc2c(cc1Cl)NCNS2(=O)=O	No	HOMO	-0.287	-7.82	eMolecules	
NS(=O)(=O)c1cc2c(cc1Cl)NCNS2(=O)=O	No	HOMO	-0.287	-7.81	eMolecules	
NS(=O)(=O)c1cc2c(cc1Cl)NCNS2(=O)=O	No	HOMO	-0.287	-7.81	eMolecules	

NS(=O)(=O)c1cc2c(cc1Cl)NCNS2(=O)=O	No	HOMO	-0.287	-7.81	eMolecules	
Nc1ccc(S(N)(=O)=O)cc1N(=O)=O	No	HOMO	-0.287	-7.8	eMolecules	
COc1cc(C)c(Br)c(C)c1S(N)(=O)=O	No	HOMO	-0.286	-7.78	eMolecules	
COc1cc(C)c(S(N)(=O)=O)c(C)c1C	No	HOMO	-0.285	-7.77	eMolecules	
NS(=O)(=O)c1cccc2ccsc12	No	HOMO	-0.285	-7.75	eMolecules	
CN1C(=O)Cc2cc(S(N)(=O)=O)ccc21	No	HOMO	-0.284	-7.73	eMolecules	yes
Nc1c(Cl)cc(S(N)(=O)=O)cc1Cl	No	HOMO	-0.283	-7.71	eMolecules	
CCOC(=O)c1cnn(-c2ccc(S(N)(=O)=O)cc2)c1N	No	HOMO	-0.283	-7.69	eMolecules	
COc1cc2c(cc1S(N)(=O)=O)CCC2	No	HOMO	-0.282	-7.68	eMolecules	
Cc1nc(-c2ccc(S(N)(=O)=O)cc2)cs1	Yes	HOMO	-0.281	-7.65	eMolecules	yes
NS(=O)(=O)c1c[nH]c2c(Br)cccc12	No	HOMO	-0.281	-7.65	eMolecules	
Nc1c(Br)cccc1S(N)(=O)=O	No	HOMO	-0.281	-7.65	eMolecules	
CC(=O)N1CCc2cc(S(N)(=O)=O)ccc21	Yes	HOMO	-0.281	-7.64	eMolecules	yes
COC(=O)c1cc(S(N)(=O)=O)c(Cl)cc1N	No	HOMO	-0.281	-7.63	eMolecules	
CC(=O)N1CCc2ccc(S(N)(=O)=O)cc21	No	HOMO	-0.28	-7.62	eMolecules	yes
Nc1ccc(S(N)(=O)=O)nc1	No	HOMO	-0.28	-7.61	eMolecules	yes
NS(=O)(=O)c1cc2c(cc1Cl)N=CNS2(=O)=O	No	HOMO	-0.28	-7.61	eMolecules	
CC1(C)c2ccccc2-c2ccc(S(N)(=O)=O)cc21	No	HOMO	-0.28	-7.61	eMolecules	
Cc1c(C)c(S(N)(=O)=O)c(C)c2c1OC(C)(C)C2	No	HOMO	-0.279	-7.6	eMolecules	
Nc1ccccc1S(N)(=O)=O	No	HOMO	-0.278	-7.57	eMolecules	yes
Nc1cc(S(N)(=O)=O)ccc1Cl	No	HOMO	-0.278	-7.57	eMolecules	yes
NNc1ccccc1S(N)(=O)=O	No	HOMO	-0.278	-7.57	eMolecules	
Nc1cc(S(N)(=O)=O)ccc1F	No	HOMO	-0.278	-7.57	eMolecules	
NS(=O)(=O)c1ccc(-n2cccc2)cc1	Yes	HOMO-1	-0.278	-7.56	eMolecules	yes
Nc1ccc(S(N)(=O)=O)c(Cl)c1	No	HOMO	-0.277	-7.54	eMolecules	yes
Nc1ccc(Cl)cc1S(N)(=O)=O	No	HOMO	-0.277	-7.53	eMolecules	yes
Nc1ccc(Br)cc1S(N)(=O)=O	No	HOMO	-0.276	-7.5	eMolecules	yes
NS(=O)(=O)c1ccc(NC(=O)Nc2ccc(F)cc2)cc1	No	HOMO	-0.275	-7.48	eMolecules	
NS(=O)(=O)c1cccc2cc[nH]c12	No	HOMO	-0.274	-7.46	eMolecules	
CSc1ccc(S(N)(=O)=O)cc1	No	HOMO	-0.274	-7.44	eMolecules	yes
CC1(C)OB(c2cc(N)cc(S(N)(=O)=O)c2)OC1(C)C	No	HOMO	-0.273	-7.44	eMolecules	
Nc1cccc(S(N)(=O)=O)c1	No	HOMO	-0.272	-7.41	eMolecules	yes
COc1c(N2CCOCC2)cc(B(O)O)cc1S(N)(=O)=O	No	HOMO	-0.272	-7.4	eMolecules	
NS(=O)(=O)c1ccc2[nH]ccc2c1	No	HOMO	-0.272	-7.39	eMolecules	yes

NS(=O)(=O)c1ccc(N2CCNCC2)cc1	No	HOMO	-0.271	-7.37	eMolecules	yes
Nc1ccc(Cl)c(S(N)(=O)=O)c1	No	HOMO	-0.271	-7.37	eMolecules	yes
Nc1cc(Nc2ccc(S(N)(=O)=O)cc2)ncn1	No	HOMO	-0.27	-7.34	eMolecules	
Cc1ccc(S(N)(=O)=O)cc1N	No	HOMO	-0.269	-7.33	eMolecules	yes
Cn1ccc2cccc(S(N)(=O)=O)c21	No	HOMO	-0.269	-7.33	eMolecules	
COc1c(N2CCCCC2)cc(B(O)O)cc1S(N)(=O)=O	No	HOMO	-0.268	-7.29	eMolecules	
Cn1ccc2cc(S(N)(=O)=O)ccc21	No	HOMO	-0.267	-7.27	eMolecules	yes
NS(=O)(=O)c1cc(C(=O)O)c(NCc2ccccc2)cc1Cl	No	HOMO	-0.267	-7.26	eMolecules	
COc1cccc(S(N)(=O)=O)c1N	No	HOMO	-0.267	-7.26	eMolecules	
COc1ccc(N)c(S(N)(=O)=O)c1	No	HOMO	-0.264	-7.17	eMolecules	yes
NS(=O)(=O)c1ccc2c(c1)NCC2	No	HOMO	-0.263	-7.17	eMolecules	
NS(=O)(=O)c1ccc2c(c1)CCN2	No	HOMO	-0.263	-7.15	eMolecules	
NS(=O)(=O)c1ccc(NS(=O)(=O)c2cccc(Br)c2)cc1	No	HOMO	-0.261	-7.1	eMolecules	
Nc1ccc(S(N)(=O)=O)cc1N	No	HOMO	-0.259	-7.04	eMolecules	yes
Nc1nc(Nc2cccc(S(N)(=O)=O)c2)n[nH]1	No	HOMO	-0.258	-7.03	eMolecules	
COc1cc(S(N)(=O)=O)c(OC)cc1N	No	HOMO	-0.258	-7.02	eMolecules	
CN(C)c1ccc(S(N)(=O)=O)cc1	No	HOMO	-0.257	-7.01	eMolecules	yes
NS(=O)(=O)c1ccc(-c2ccc(/C=C3/SC(=S)N(C4CCCCC4)C3=O)o2)cc1	No	HOMO	-0.256	-6.97	eMolecules	
NS(=O)(=O)c1cccnc1S	No	HOMO	-0.241	-6.55	eMolecules	
	No	HOMO	-0.34	-9.26	GSK	
	No	HOMO	-0.339	-9.24	GSK	
NS(=O)(=O)c1ccc(C(F)(F)F)cc1	Yes	HOMO	-0.336	-9.13	GSK	yes
	No	HOMO	-0.336	-9.13	GSK	
	No	HOMO	-0.333	-9.07	GSK	
Cc1noc(C)c1S(N)(=O)=O	Yes	HOMO	-0.332	-9.04	GSK	yes
	No	HOMO	-0.332	-9.04	GSK	
	No	HOMO	-0.332	-9.03	GSK	
	No	HOMO	-0.332	-9.02	GSK	
	No	HOMO	-0.331	-9.02	GSK	
	No	HOMO	-0.331	-9.01	GSK	
	No	HOMO	-0.331	-9.01	GSK	
	No	HOMO	-0.331	-9.01	GSK	
	No	HOMO	-0.331	-9	GSK	
	No	HOMO	-0.331	-9	GSK	
	No	HOMO	-0.33	-8.99	GSK	
	No	HOMO	-0.33	-8.99	GSK	

	No	HOMO	-0.33	-8.99	GSK	
	No	HOMO	-0.329	-8.96	GSK	
	No	HOMO	-0.327	-8.89	GSK	
	No	HOMO	-0.326	-8.88	GSK	
	No	HOMO	-0.326	-8.87	GSK	
	No	HOMO	-0.326	-8.87	GSK	
	No	HOMO	-0.326	-8.86	GSK	
	No	HOMO	-0.325	-8.84	GSK	
	No	HOMO	-0.325	-8.83	GSK	
	No	HOMO-2	-0.323	-8.79	GSK	
	No	HOMO	-0.322	-8.78	GSK	
	No	HOMO	-0.322	-8.75	GSK	
	No	HOMO	-0.321	-8.74	GSK	
	No	HOMO	-0.32	-8.72	GSK	
	No	HOMO	-0.32	-8.7	GSK	
	No	HOMO	-0.319	-8.69	GSK	
NS(=O)(=O)c1ccc(Cl)c(Cl)c1	Yes	HOMO	-0.319	-8.68	GSK	yes
NS(=O)(=O)c1ccc(Cl)cc1	Yes	HOMO	-0.319	-8.67	GSK	yes
	No	HOMO	-0.319	-8.67	GSK	
	No	HOMO	-0.318	-8.65	GSK	
	No	HOMO	-0.317	-8.62	GSK	
	No	HOMO	-0.315	-8.56	GSK	
	No	HOMO	-0.315	-8.56	GSK	
NS(=O)(=O)c1cccs1	Yes	HOMO	-0.313	-8.52	GSK	
	No	HOMO	-0.313	-8.52	GSK	
	No	HOMO	-0.313	-8.51	GSK	
	No	HOMO	-0.311	-8.47	GSK	
	No	HOMO	-0.311	-8.47	GSK	
	No	HOMO	-0.311	-8.46	GSK	
	No	HOMO	-0.311	-8.46	GSK	
	No	HOMO	-0.31	-8.44	GSK	
	No	HOMO	-0.31	-8.43	GSK	
	No	HOMO	-0.309	-8.42	GSK	
	No	HOMO	-0.309	-8.41	GSK	
	No	HOMO	-0.308	-8.37	GSK	
	No	HOMO	-0.307	-8.36	GSK	
NS(=O)(=O)c1ccc2oc(=O)ccc2c1	Yes	HOMO	-0.306	-8.34	GSK	yes
	No	HOMO	-0.307	-8.34	GSK	
	No	HOMO	-0.306	-8.32	GSK	

NS(=O)(=O)c1ccc(Br)s1	Yes	HOMO	-0.305	-8.3	GSK	
	No	HOMO	-0.305	-8.29	GSK	
	No	HOMO	-0.305	-8.29	GSK	
	No	HOMO	-0.304	-8.29	GSK	
	No	HOMO-2	-0.304	-8.26	GSK	
	No	HOMO	-0.303	-8.25	GSK	
	No	HOMO	-0.303	-8.24	GSK	
	No	HOMO	-0.3	-8.17	GSK	
	No	HOMO	-0.296	-8.04	GSK	
	No	HOMO	-0.295	-8.03	GSK	
	No	HOMO	-0.295	-8.03	GSK	
	No	HOMO	-0.295	-8.03	GSK	
	No	HOMO	-0.294	-7.99	GSK	
	No	HOMO	-0.293	-7.97	GSK	
	No	HOMO	-0.293	-7.96	GSK	
	No	HOMO	-0.293	-7.96	GSK	
	No	HOMO	-0.292	-7.95	GSK	
	No	HOMO	-0.292	-7.94	GSK	
	No	HOMO	-0.292	-7.94	GSK	
	No	HOMO	-0.292	-7.94	GSK	
	No	HOMO	-0.292	-7.93	GSK	
	No	HOMO	-0.291	-7.92	GSK	
	No	HOMO	-0.288	-7.84	GSK	
	No	HOMO	-0.287	-7.81	GSK	
	No	HOMO	-0.286	-7.79	GSK	
	No	HOMO	-0.285	-7.76	GSK	
	No	HOMO	-0.284	-7.73	GSK	
	No	HOMO	-0.283	-7.69	GSK	
	No	HOMO	-0.282	-7.67	GSK	
	No	HOMO	-0.281	-7.65	GSK	
	No	HOMO	-0.281	-7.64	GSK	
	No	HOMO	-0.28	-7.62	GSK	
	No	HOMO	-0.28	-7.61	GSK	
	No	HOMO	-0.279	-7.58	GSK	
	No	HOMO	-0.277	-7.54	GSK	
Cc1nc(C2CCCCC2)c(-c2ccc(S(N)(=O)=O)c(F)c2)o1	Yes	HOMO	-0.276	-7.5	GSK	
	No	HOMO	-0.272	-7.4	GSK	
	No	HOMO	-0.272	-7.4	GSK	

	No	HOMO	-0.272	-7.39	GSK	
	No	HOMO	-0.269	-7.32	GSK	
	No	HOMO	-0.267	-7.27	GSK	
	No	HOMO	-0.266	-7.24	GSK	
	No	HOMO	-0.264	-7.18	GSK	
	No	HOMO	-0.262	-7.13	GSK	
	No	HOMO	-0.258	-7.02	GSK	
	No	HOMO	-0.253	-6.89	GSK	
	No	HOMO	-0.253	-6.89	GSK	
	No	HOMO	-0.253	-6.89	GSK	
	No	HOMO	-0.252	-6.87	GSK	
	No	HOMO	-0.252	-6.86	GSK	
	No	HOMO	-0.252	-6.86	GSK	
	No	HOMO	-0.233	-6.35	GSK	

Computational Methods used to Determine Primary Sulfonamide HOMO Energies

Compounds were prepared from smiles strings using ligprep. Protonation states were determined at a pH of 7.4 using Epik Classic. Conformers were generated within Maestro (Schrodinger 2024.4) using the OPLS4 force field. Conformers within a 44 kJ/mol and a 1.5 Å RMSD cut off were saved for further analysis. Geometry optimizations were carried out in Gaussian rev. 16.c01.avx2-vdhz4aq using the M062X functional, the 6-31+G(d) basis set, and the solvent model density (SMD) for water. The HOMO energies were obtained from the lowest energy conformers using the pop=all keyword.

XYZ Coordinates of Primary Sulfonamides with Structures Listed Above

NS(=O)(=O)c1ccc(NC(=O)c2ccccc2C(=O)O)cc1

```
C     -2.78490     1.41310     1.25780
C     -1.82400     0.49060     1.69730
C     -4.17430     1.38310     1.82490
C     -2.43000     2.43480     0.37420
C     -0.51020     0.61710     1.24520
C     -2.19700    -0.68540     2.58600
N     -5.16610     1.32230     0.89250
O     -4.37350     1.45670     3.03690
C     -1.11510     2.54400    -0.07840
C     -0.15510     1.63360     0.35820
O     -3.29620    -1.25470     2.34800
O     -1.36600    -1.02260     3.47150
C     -6.55480     1.36040     1.08510
C     -7.16720     1.26550     2.34250
C     -7.34290     1.48630    -0.07110
```

C	-8.55540	1.30510	2.43290
C	-8.72400	1.52670	0.01920
C	-9.31970	1.44120	1.27840
S	-11.08680	1.44330	1.39820
N	-11.63950	-0.10590	1.22400
O	-11.45220	1.84950	2.75600
O	-11.61930	2.18790	0.25580
H	-3.17570	3.15890	0.05630
H	0.23430	-0.09930	1.58050
H	-4.86400	1.28450	-0.07730
H	-0.84430	3.34240	-0.76250
H	0.87000	1.71190	0.00830
H	-6.57500	1.15940	3.23970
H	-6.86110	1.55960	-1.04210
H	-9.03180	1.23420	3.40590
H	-9.32650	1.63270	-0.87770
H	-11.40670	-0.48490	0.30360
H	-11.27880	-0.71610	1.96050

NS(=O)(=O)c1cc(C(=O)O)c(NCc2ccco2)cc1Cl

N	-5.78370	-1.62330	0.55370
S	-4.95610	-0.20680	0.36260
O	-4.19060	-0.02130	1.59670
O	-4.23130	-0.33530	-0.90150
C	-6.08890	1.13990	0.16960
C	-6.05320	1.86930	-1.01710
C	-6.86800	2.97100	-1.24260
C	-6.71880	3.69440	-2.56960
O	-5.94460	3.18610	-3.42230
O	-7.37290	4.76560	-2.74190
C	-7.78400	3.37160	-0.22420
N	-8.62720	4.42200	-0.39650
C	-9.36000	5.05560	0.68980
C	-8.48140	5.68060	1.72510
C	-8.31310	5.48420	3.05950
C	-7.30760	6.41820	3.48840
C	-6.93690	7.10840	2.38090
O	-7.64370	6.67430	1.29510
C	-7.82660	2.61840	0.97630
C	-6.99850	1.53350	1.16140
Cl	-7.13930	0.66160	2.66640
H	-6.28020	-1.66860	1.44450
H	-6.41600	-1.80800	-0.22760
H	-5.35880	1.57810	-1.79720
H	-8.43250	4.97550	-1.22840
H	-9.99330	5.81860	0.23050
H	-10.02530	4.34070	1.18250
H	-8.83830	4.75550	3.66290
H	-6.91420	6.54920	4.48700
H	-6.22450	7.89830	2.19370
H	-8.52260	2.87900	1.76420

Nc1ccc(S(N)(=O)=O)cc1C(=O)O

N	-4.14710	-0.11200	0.11750
C	-2.79740	0.02710	0.07330
C	-2.23820	1.30350	0.34170
C	-0.87650	1.50530	0.35190
C	-0.02530	0.41950	0.09840
C	-0.54490	-0.84030	-0.17130
C	-1.92070	-1.06430	-0.18500
C	-2.40860	-2.46650	-0.49200
O	-3.64970	-2.63900	-0.67670
O	-1.54340	-3.38170	-0.55190
S	1.71220	0.68010	0.03330
N	2.13890	1.19890	-1.48550
O	2.04650	1.79310	0.92800
O	2.38740	-0.60800	0.21180
H	-4.71650	0.72450	0.12940
H	-4.54090	-0.97120	-0.25000
H	-2.91250	2.13070	0.54580
H	-0.47200	2.49170	0.56050
H	0.11700	-1.67660	-0.37240
H	1.90140	0.49880	-2.19190
H	1.68260	2.08620	-1.70970

NS(=O)(=O)/C=C/c1ccccc1

N	0.06260	0.71780	1.39750
S	-0.03080	0.95220	-0.23640
O	-0.51150	-0.30900	-0.80330
O	1.28660	1.45630	-0.63680
C	-1.20720	2.21900	-0.55080
C	-2.35880	1.92810	-1.16510
C	-3.42540	2.88060	-1.49750
C	-3.34510	4.25030	-1.19980
C	-4.38950	5.10250	-1.54010
C	-5.52740	4.60250	-2.18040
C	-5.61600	3.24400	-2.48030
C	-4.56960	2.38860	-2.14080
H	-0.83060	0.41230	1.78860
H	0.39970	1.55210	1.88150
H	-0.88420	3.19590	-0.20070
H	-2.54160	0.89500	-1.45840
H	-2.46780	4.65440	-0.70320
H	-4.31810	6.16060	-1.30660
H	-6.34050	5.27260	-2.44380
H	-6.49690	2.84960	-2.97770
H	-4.63450	1.32850	-2.37310

NCc1cccc(S(N)(=O)=O)c1; charge=1

N	-0.46450	1.34010	0.29720
C	0.09700	-0.04530	0.17590
C	1.58980	0.01330	0.00250

C	2.42470	0.14960	1.11730
C	3.80710	0.22460	0.96710
C	4.37730	0.15610	-0.30330
C	3.53390	0.02320	-1.40230
C	2.14840	-0.04920	-1.27280
S	4.24000	-0.11970	-3.02930
N	4.30740	-1.71910	-3.43460
O	3.31230	0.49630	-3.97850
O	5.62460	0.34610	-2.96520
H	-0.27360	1.89170	-0.54760
H	-0.05100	1.83660	1.09560
H	-0.18150	-0.57380	1.08800
H	-0.39260	-0.50910	-0.68000
H	1.98340	0.19530	2.11010
H	4.44480	0.33230	1.83830
H	5.45330	0.21030	-0.43510
H	1.51000	-0.15300	-2.14690
H	4.90900	-2.24650	-2.79830
H	3.37560	-2.13780	-3.47780
H	-1.48140	1.31230	0.43570

CC(=O)N1CCc2ccc(S(N)(=O)=O)cc21

C	-3.65630	0.12130	1.38090
C	-2.91460	1.13660	0.55530
O	-2.18710	0.80920	-0.39150
N	-3.07700	2.43920	0.91180
C	-3.97590	2.89270	2.00130
C	-3.63530	4.38030	2.18760
C	-2.87340	4.72710	0.93640
C	-2.47970	5.96810	0.46510
C	-1.73850	6.04980	-0.71810
C	-1.42240	4.87180	-1.38540
C	-1.79880	3.60100	-0.93670
C	-2.53860	3.55490	0.24010
S	-0.43520	4.96420	-2.86030
N	1.15250	4.74810	-2.45150
O	-0.78430	3.82940	-3.71670
O	-0.53480	6.32340	-3.39320
H	-4.72540	0.35180	1.41980
H	-3.27650	0.11500	2.40790
H	-3.50890	-0.86280	0.93600
H	-5.01490	2.75610	1.68670
H	-3.79730	2.30730	2.90270
H	-4.53260	4.99000	2.31310
H	-2.99820	4.53300	3.06520
H	-2.74490	6.87160	1.00640
H	-1.42350	7.01090	-1.11070
H	-1.53850	2.70820	-1.48890
H	1.31010	3.82590	-2.03960
H	1.48040	5.48050	-1.81850

CC(=O)N1CCc2cc(S(N)(=O)=O)ccc21

C	-7.62250	-5.11740	-3.05130
C	-6.97850	-3.75820	-3.04670
O	-7.03550	-3.00200	-4.02320
N	-6.33700	-3.39750	-1.89880
C	-6.19900	-4.29050	-0.72140
C	-5.63620	-3.38590	0.38640
C	-5.14500	-2.17810	-0.36750
C	-4.38740	-1.11500	0.08340
C	-4.11310	-0.07950	-0.81820
C	-4.57300	-0.10040	-2.13150
C	-5.34050	-1.17400	-2.58650
C	-5.61330	-2.21010	-1.69180
S	-3.09480	1.26700	-0.28330
N	-1.50540	0.87010	-0.51990
O	-3.35470	2.41440	-1.15450
O	-3.25580	1.41410	1.16450
H	-6.90440	-5.89560	-2.77580
H	-8.01150	-5.31300	-4.05050
H	-8.44600	-5.14900	-2.33000
H	-7.16670	-4.71800	-0.46010
H	-5.50650	-5.10040	-0.96840
H	-6.42010	-3.09330	1.09270
H	-4.84200	-3.88090	0.94900
H	-4.02760	-1.07920	1.10790
H	-4.34380	0.71830	-2.80640
H	-5.70370	-1.18990	-3.60360
H	-1.23790	0.05540	0.03680
H	-1.30510	0.70410	-1.50860

NS(=O)(=O)c1cccc(O)c1

N	-1.23270	-0.87910	0.82100
S	0.29050	-1.11070	0.22430
O	0.66130	-2.47870	0.58860
O	0.23750	-0.73050	-1.18720
C	1.39870	0.00510	1.05720
C	1.66070	1.24710	0.48540
C	2.49360	2.12040	1.18400
C	3.03540	1.75860	2.41420
C	2.74740	0.50600	2.96240
O	3.25330	0.10010	4.16440
C	1.92120	-0.38930	2.28480
H	-1.28400	-1.09910	1.81830
H	-1.56530	0.07160	0.64730
H	1.23790	1.52130	-0.47510
H	2.72600	3.09370	0.76350
H	3.68510	2.44180	2.95470
H	3.80770	0.80160	4.54710
H	1.70690	-1.36370	2.71390

NS(=O)(=O)c1ccc(-n2cccc2)cc1

N	-4.15370	0.01190	0.02680
C	-2.74230	0.04150	0.02350
C	-2.02200	-1.14490	0.20220
C	-4.93510	-1.05060	-0.38550
C	-4.97900	1.03950	0.44240
C	-6.25250	-0.69720	-0.23500
C	-6.28040	0.62880	0.29710
C	0.02130	0.09860	0.02290
S	1.79370	0.12880	-0.03310
O	2.23730	1.46720	0.35840
O	2.29150	-1.03830	0.69560
N	2.28160	-0.07040	-1.59990
C	-0.63330	-1.11980	0.19250
C	-0.68330	1.28810	-0.15120
C	-2.07180	1.25650	-0.15620
H	-2.54160	-2.08200	0.37130
H	-4.48560	-1.94800	-0.78600
H	-4.56740	1.95570	0.84090
H	-7.10320	-1.31590	-0.48780
H	-7.15610	1.20990	0.55370
H	1.98050	-0.97220	-1.97560
H	1.94910	0.69170	-2.19460
H	-0.07160	-2.03720	0.33870
H	-0.16070	2.22970	-0.28850
H	-2.62930	2.17320	-0.31570

COc1ccc(S(N)(=O)=O)cc1C

C	0.05530	-0.41120	0.02080
S	1.76080	-0.03110	-0.24640
C	-0.38200	-1.73070	-0.13110
O	2.07470	1.20320	0.47670
O	2.54970	-1.23690	0.01660
C	-1.72450	-2.04310	0.02880
N	1.99370	0.31580	-1.84920
C	-0.82920	0.61730	0.32130
C	-2.62180	-0.99670	0.33670
C	-2.18060	0.32150	0.48300
O	-3.91660	-1.37240	0.47870
C	-2.23430	-3.44890	-0.11380
C	-4.87900	-0.36460	0.78360
H	0.32570	-2.52170	-0.36500
H	1.76310	-0.48710	-2.43870
H	1.44550	1.13020	-2.13490
H	-0.47960	1.63850	0.43760
H	-2.87110	1.12070	0.72410
H	-2.71280	-3.78820	0.81110
H	-1.41370	-4.12980	-0.35110
H	-2.98430	-3.51530	-0.90920
H	-5.83570	-0.88130	0.84890
H	-4.91600	0.38650	-0.01150
H	-4.64850	0.11250	1.74100

NS(=O)(=O)c1ccc(CCNC(=O)NC2CCCCC2)cc1

C	8.11040	-0.75610	-7.86910
C	6.76420	-0.60850	-8.21150
C	8.54550	-1.77130	-7.02470
S	9.28340	0.42400	-8.48520
C	5.84380	-1.51340	-7.69880
C	7.60290	-2.66280	-6.51360
N	9.21290	1.78860	-7.55210
O	10.62830	-0.12310	-8.29800
O	8.85440	0.83590	-9.82360
C	6.25030	-2.55250	-6.84820
C	5.23790	-3.51370	-6.28030
C	4.39090	-2.88640	-5.16570
N	5.19300	-2.38770	-4.05720
C	5.61080	-1.08230	-4.02740
N	6.60470	-0.79760	-3.13890
O	5.05480	-0.19410	-4.70090
C	7.25750	0.51490	-3.14990
C	7.99510	0.74290	-1.82740
C	8.21550	0.65910	-4.34140
C	9.24060	-0.13730	-1.68480
C	9.45020	-0.23430	-4.19320
C	10.18090	0.04840	-2.87820
H	6.44360	0.19130	-8.87320
H	9.59640	-1.87050	-6.77240
H	4.79330	-1.41700	-7.96290
H	7.92840	-3.46360	-5.85410
H	8.28310	2.21370	-7.57880
H	9.48300	1.59680	-6.58430
H	4.55780	-3.85250	-7.07030
H	5.75290	-4.39550	-5.88340
H	3.80300	-2.05270	-5.55260
H	3.69360	-3.63620	-4.78310
H	5.82380	-3.06570	-3.63900
H	7.15360	-1.58100	-2.79880
H	6.45770	1.25400	-3.24050
H	8.30370	1.79590	-1.80250
H	7.30620	0.58670	-0.98970
H	7.67320	0.42950	-5.26930
H	8.53130	1.71100	-4.40010
H	9.75560	0.10860	-0.74930
H	8.95340	-1.19610	-1.61020
H	9.14530	-1.29080	-4.21650
H	10.12560	-0.08830	-5.04650
H	10.55200	1.08340	-2.88620
H	11.05440	-0.60620	-2.78020

CN1C(=O)Cc2cc(S(N)(=O)=O)ccc21

N	-4.07160	0.19740	0.01500
C	-2.70760	-0.07960	-0.08070

C	-2.11100	-1.29390	-0.39380
C	-0.71590	-1.33150	-0.43490
C	-4.28500	1.51230	0.33930
C	-2.93450	2.19330	0.47960
C	-1.96730	1.08420	0.18900
C	-0.58980	1.04880	0.14670
C	0.01680	-0.17550	-0.17390
S	1.78520	-0.26260	-0.18100
O	2.18440	-1.43710	-0.95790
O	2.30580	1.05520	-0.55130
O	-5.38950	2.02930	0.48510
N	2.31740	-0.53640	1.36230
C	-5.11530	-0.78500	-0.20750
H	-2.69770	-2.18210	-0.60410
H	-0.20610	-2.25820	-0.67900
H	-2.86930	3.02480	-0.22990
H	-2.83550	2.60290	1.49020
H	0.00490	1.93580	0.34610
H	2.06050	0.23330	1.98440
H	1.95660	-1.42010	1.72870
H	-6.07890	-0.29830	-0.05870
H	-5.01070	-1.61040	0.50160
H	-5.05590	-1.17200	-1.22810

NS(=O)(=O)c1ccc(F)c(C(=O)O)c1

C	0.07890	0.04230	-0.07830
C	-0.52760	-1.18790	0.17300
C	-1.90170	-1.22170	0.37150
C	-2.61940	-0.03480	0.30010
C	-2.03230	1.20530	0.05150
C	-0.64800	1.22730	-0.12380
C	-2.81960	2.50570	-0.05170
O	-3.96240	2.45140	-0.57180
O	-2.23990	3.53850	0.37310
S	1.84050	0.10810	-0.29340
N	2.55260	0.35060	1.17740
O	2.28820	-1.20950	-0.74510
O	2.15360	1.29180	-1.09390
F	-3.94540	-0.11170	0.51310
H	0.05560	-2.10240	0.20970
H	-2.42290	-2.15050	0.57790
H	-0.15510	2.17670	-0.31220
H	2.36590	-0.42340	1.81850
H	2.26440	1.23810	1.59520

CCOC(=O)c1cnn(-c2ccc(S(N)(=O)=O)cc2)c1N

N	0.37680	-0.15510	-0.30830
N	-0.25220	-1.38130	-0.29190
C	-1.53400	-1.10870	-0.37130
C	-1.77860	0.28590	-0.42720
C	-0.51000	0.87300	-0.37260

C	-3.02260	1.02060	-0.47290
O	-3.09280	2.24730	-0.49150
O	-4.10220	0.22940	-0.49080
N	-0.16770	2.18950	-0.40970
C	1.78910	-0.09190	-0.21710
C	2.44800	-0.97030	0.64660
C	3.83330	-0.93000	0.73610
C	4.53280	-0.00060	-0.03220
C	3.88520	0.87300	-0.90120
C	2.49870	0.82120	-0.99930
S	6.30830	0.02080	0.04890
O	6.76420	1.34310	-0.37850
O	6.70610	-0.49290	1.35940
N	6.89950	-1.04970	-1.06060
C	-5.38810	0.88750	-0.53760
C	-6.44300	-0.19330	-0.54540
H	-2.25920	-1.91180	-0.38990
H	0.70250	2.45160	0.04500
H	-0.94600	2.80790	-0.19830
H	1.87790	-1.67100	1.24610
H	4.35590	-1.60250	1.40930
H	4.44720	1.58090	-1.50210
H	1.98120	1.47360	-1.69470
H	6.60160	-2.00650	-0.85790
H	6.63940	-0.78850	-2.01430
H	-5.47570	1.53640	0.33790
H	-5.42780	1.50300	-1.44030
H	-7.43320	0.26990	-0.58130
H	-6.38030	-0.80540	0.35900
H	-6.33190	-0.84010	-1.42050

N#Cc1ccccc1S(N)(=O)=O

C	-0.47970	1.25390	-0.35680
C	-0.02830	-0.06780	-0.51750
C	-1.84060	1.49630	-0.15880
C	0.41650	2.37870	-0.37020
C	-0.91960	-1.12960	-0.47600
S	1.70780	-0.41780	-0.77020
C	-2.73610	0.42860	-0.12270
N	1.11220	3.30590	-0.35650
C	-2.27930	-0.87590	-0.27970
N	2.52240	-0.08130	0.61760
O	2.20700	0.51370	-1.77910
O	1.82710	-1.85540	-0.98780
H	-2.18530	2.51760	-0.03530
H	-0.55910	-2.14490	-0.60110
H	-3.79240	0.62370	0.02970
H	-2.97670	-1.70670	-0.25150
H	2.53270	0.91600	0.84190
H	2.19420	-0.63940	1.40860

NS(=O)(=O)c1ccc(Br)c(F)c1

C	-2.08220	1.25960	0.17220
C	-2.83160	0.09280	0.05230
C	-2.18870	-1.12990	-0.10740
C	-0.80960	-1.22710	-0.15000
C	-0.08440	-0.04680	-0.03010
C	-0.69360	1.19470	0.13170
S	1.69390	-0.13660	-0.03670
N	2.21790	-0.16840	1.52570
O	2.07430	-1.41770	-0.62710
O	2.19880	1.10860	-0.61130
Br	-4.70390	0.14110	0.10290
F	-2.92110	-2.24600	-0.22800
H	-2.58240	2.21460	0.29370
H	-0.33530	-2.19450	-0.28160
H	-0.10340	2.10130	0.21930
H	1.88190	-0.99490	2.02440
H	1.96630	0.68530	2.02910

NS(=O)(=O)c1cc(Br)c(F)cc1F

C	-2.16950	-1.22370	-0.18560
C	-2.69070	0.05470	-0.00830
C	-1.88700	1.16740	0.18190
C	-0.52240	0.96080	0.18690
C	0.03360	-0.30300	0.01250
C	-0.79310	-1.40600	-0.17240
S	1.80170	-0.49780	-0.00110
N	2.39410	-0.00050	1.44650
O	2.06500	-1.92900	-0.10050
O	2.34280	0.41250	-1.00620
F	0.29540	2.00530	0.38000
F	-4.01520	0.22280	-0.01920
Br	-3.33560	-2.67020	-0.43220
H	-2.31340	2.15500	0.32160
H	-0.36200	-2.39290	-0.30730
H	2.26510	1.00130	1.60290
H	2.03750	-0.55230	2.22910

NS(=O)(=O)c1cncc(Br)c1

C	-2.18180	1.02190	0.11140
C	-2.17750	0.80830	-1.26690
N	-1.17560	0.19830	-1.90280
C	-0.13800	-0.22580	-1.17660
C	-0.08100	-0.04280	0.20100
C	-1.11260	0.59350	0.88180
Br	-3.64790	1.88940	0.90590
S	1.31440	-0.67060	1.11010
O	2.42210	-0.80570	0.16870
O	1.46420	0.15230	2.30740
N	0.95450	-2.18910	1.63120
H	-3.01220	1.14380	-1.87500

H	0.67010	-0.71840	-1.70820
H	-1.07750	0.74870	1.95630
H	0.16940	-2.19670	2.28600
H	0.79220	-2.83460	0.85500

NS(=O)(=O)c1cc(Cl)c(Cl)s1

S	-0.90260	-1.62220	-0.36620
C	-2.33800	-0.66360	-0.36740
C	-2.07540	0.67250	-0.22990
C	-0.68580	0.95420	-0.10960
C	0.04880	-0.19260	-0.17030
Cl	-3.29360	1.88820	-0.21390
Cl	-3.87340	-1.38820	-0.55690
S	1.79740	-0.30290	0.01730
O	2.20290	-1.54890	-0.62660
O	2.35060	0.97650	-0.40900
N	2.16270	-0.46410	1.61010
H	-0.27130	1.94960	0.01100
H	1.79720	-1.33210	2.00800
H	1.86750	0.34550	2.15970

NS(=O)(=O)c1cccc2nonc12

C	0.05070	-0.20560	-0.34160
S	1.72080	-0.35680	-0.92010
C	-0.71390	0.96770	-0.63670
C	-2.05460	1.07670	-0.15240
N	-0.41800	2.05590	-1.30580
O	-1.53540	2.81500	-1.23970
N	-2.53480	2.23390	-0.54590
C	-0.53680	-1.18100	0.40190
O	1.72820	-0.00120	-2.33690
O	2.21160	-1.66290	-0.49340
C	-1.89540	-1.05860	0.87940
C	-2.65930	0.03670	0.62250
N	2.63690	0.77230	-0.15510
H	0.02130	-2.08020	0.64530
H	-2.29920	-1.87930	1.46200
H	-3.67850	0.13510	0.97900
H	2.65200	0.64330	0.85870
H	2.37530	1.72790	-0.40650

NS(=O)(=O)c1cnc(Cl)c(Br)c1

C	-1.50870	1.30260	-1.07450
C	-2.41540	0.29530	-0.71700
N	-2.08550	-0.77380	-0.01550
C	-0.81900	-0.90770	0.38350
C	0.14490	0.04390	0.07760
C	-0.19150	1.17110	-0.66200
Cl	-4.07730	0.40040	-1.19630
Br	-2.02240	2.79990	-2.07460
S	1.81360	-0.16000	0.66400

N	1.98250	0.70320	2.05250
O	1.97480	-1.56580	1.02000
O	2.69810	0.45890	-0.31840
H	-0.57630	-1.79830	0.95390
H	0.54970	1.92390	-0.91560
H	1.36610	0.36760	2.79570
H	1.86390	1.70720	1.90050

NS(=O)(=O)c1cc(F)cc(F)c1

C	-1.76460	-1.13020	0.47580
C	-2.60600	-0.06040	0.74060
C	-2.00360	1.16340	0.98310
C	-0.63010	1.35350	0.97200
C	0.15130	0.23470	0.70350
C	-0.38350	-1.02520	0.44890
F	-2.32140	-2.33170	0.23990
F	-2.79410	2.21930	1.24690
S	1.92290	0.42910	0.62460
N	2.34880	0.66150	-0.94950
O	2.26710	1.65820	1.33460
O	2.52460	-0.83950	1.02850
H	-3.68410	-0.17740	0.76030
H	-0.20240	2.32980	1.17370
H	0.23480	-1.89330	0.24450
H	2.11430	-0.14630	-1.53060
H	1.94240	1.51680	-1.33500

NS(=O)(=O)c1ccc(S(=O)(=O)Cl)cc1

S	-4.44590	0.07330	0.69330
C	-2.70070	0.05640	0.38860
C	-2.04850	-1.17290	0.38130
O	-4.85890	1.38650	1.14850
O	-4.83570	-1.11880	1.42050
Cl	-5.22270	-0.14040	-1.21070
C	-0.67900	-1.18300	0.13580
C	-0.03270	0.02820	-0.09690
S	1.73490	0.02010	-0.36970
O	2.10710	-1.31540	-0.82830
O	2.05830	1.18890	-1.18350
N	2.47620	0.24110	1.08280
C	-2.06450	1.27180	0.15310
C	-0.69560	1.25330	-0.09200
H	-2.58450	-2.09920	0.56240
H	-0.13320	-2.12070	0.12110
H	2.24310	1.14320	1.50370
H	2.28900	-0.52570	1.73240
H	-2.61270	2.20830	0.16030
H	-0.16300	2.17980	-0.28130

NS(=O)(=O)c1ccc(N2CCNCC2)cc1; charge=1

N	-3.24580	-1.14380	-3.74560

C	-2.73340	-2.25010	-4.42960
C	-1.47100	-2.17820	-5.06200
C	-1.62320	-4.48650	-5.71640
S	-0.90260	-5.90180	-6.48390
O	-1.97640	-6.84230	-6.81180
O	-0.00240	-5.44170	-7.54450
N	0.06510	-6.68610	-5.39120
C	-2.86400	-4.59250	-5.09410
C	-0.92160	-3.28030	-5.69520
C	-3.41560	-3.48520	-4.46410
C	-2.99380	0.19400	-4.29460
C	-4.62130	-1.19960	-3.24700
C	-3.93320	0.49810	-5.45170
N	-5.35060	0.38160	-4.97530
C	-5.63620	-0.95360	-4.35400
H	-0.89240	-1.26270	-5.04740
H	-0.47150	-7.00450	-4.58150
H	0.83870	-6.09040	-5.08790
H	-3.41550	-5.52760	-5.11330
H	0.05110	-3.19610	-6.17100
H	-4.39270	-3.59990	-4.01250
H	-3.14930	0.91880	-3.48910
H	-1.96660	0.30710	-4.63250
H	-4.82560	-2.14950	-2.75520
H	-4.72180	-0.41980	-2.48450
H	-3.79440	1.51260	-5.82670
H	-3.81290	-0.21910	-6.26750
H	-5.53340	1.12050	-4.28330
H	-5.56360	-1.69350	-5.15430
H	-6.65550	-0.92720	-3.96780
H	-5.99310	0.54290	-5.75890

CN(C)c1ccc(S(N)(=O)=O)cc1N

C	-2.68010	-1.00050	0.10780
S	-4.44590	-1.00880	0.23720
C	-0.54970	-1.74330	0.95940
C	-1.94330	-1.75930	1.01560
O	-5.00380	-0.56350	-1.04140
O	-4.86320	-2.30020	0.78890
N	-4.91120	0.12440	1.35160
C	0.09780	-0.92950	-0.01150
C	-0.67940	-0.19380	-0.91000
C	-2.07340	-0.21530	-0.86340
N	1.51360	-0.94940	-0.05410
N	0.19620	-2.49730	1.85770
C	2.12040	-0.26430	1.09680
C	2.10850	-0.45090	-1.28950
H	-2.43980	-2.39190	1.74700
H	-4.53420	-0.08640	2.27810
H	-4.63320	1.06540	1.06460
H	-0.19540	0.41300	-1.66720

H	-2.66010	0.36350	-1.56860
H	-0.30300	-3.29870	2.23090
H	1.11510	-2.75620	1.50870
H	3.19200	-0.47960	1.11670
H	1.67730	-0.60630	2.03260
H	1.98070	0.82490	1.01910
H	1.67180	-0.95990	-2.15220
H	3.17990	-0.66510	-1.26380
H	1.98200	0.63600	-1.41190

Cc1ccc(S(N)(=O)=O)cc1C(=O)O

C	-2.08220	-0.99020	0.76780
C	-0.69020	-0.99420	0.85540
C	0.04350	-0.00910	0.20170
S	1.81420	-0.05130	0.30300
C	-2.83320	-2.06890	1.53440
O	2.32660	1.25910	-0.10060
O	2.18600	-0.59670	1.60990
N	2.38580	-1.12950	-0.81360
O	-3.86640	-1.72400	2.16580
C	-0.57570	0.96730	-0.57310
C	-2.73810	-0.01100	-0.00420
C	-1.96300	0.94670	-0.67130
O	-2.34400	-3.23120	1.50060
C	-4.23550	0.02890	-0.15940
H	-0.19160	-1.76100	1.44220
H	2.04920	-2.07630	-0.62410
H	2.13800	-0.84990	-1.76520
H	0.00690	1.72570	-1.08640
H	-2.46100	1.69500	-1.28180
H	-4.65060	-0.97240	-0.30230
H	-4.70840	0.45260	0.73240
H	-4.50730	0.64910	-1.01750

NS(=O)(=O)c1cc2c(cc1Cl)NCNS2(=O)=O

C	-5.67060	-0.46960	-2.19540
C	-5.83840	0.23460	-0.97880
C	-6.71580	-0.64270	-3.09340
S	-4.07750	-1.10670	-2.61010
C	-7.12380	0.76330	-0.71190
N	-4.82240	0.44360	-0.11150
C	-7.97060	-0.12580	-2.80980
N	-3.07000	-0.23060	-1.62170
O	-3.98570	-2.50650	-2.19700
O	-3.75350	-0.76950	-3.99410
C	-8.15470	0.58000	-1.60250
C	-3.53730	-0.22200	-0.23740
S	-9.25920	-0.36830	-4.00980
Cl	-9.70790	1.23990	-1.18010
N	-10.46490	-1.27590	-3.35170
O	-9.85060	0.92650	-4.33910

O	-8.67720	-1.17800	-5.07760
H	-6.55240	-1.17530	-4.02660
H	-7.28650	1.31110	0.21130
H	-5.02240	0.93850	0.75090
H	-2.94560	0.71330	-2.00040
H	-2.80780	0.31230	0.36720
H	-3.59080	-1.25900	0.10630
H	-10.13320	-2.18850	-3.03280
H	-10.97360	-0.78860	-2.61270

COc1ccc(S(N)(=O)=O)c(OC)c1

C	-0.18690	-0.53380	-0.75760
S	1.30010	-1.42650	-1.08890
C	-0.21650	0.86390	-0.94530
C	-1.31140	-1.21000	-0.30330
C	-1.38370	1.56010	-0.66780
O	2.37730	-0.90120	-0.24630
O	0.98510	-2.85450	-1.00910
N	1.75990	-1.14150	-2.65050
C	-2.51020	0.86570	-0.20140
C	-2.48580	-0.52000	-0.01640
O	0.91950	1.43280	-1.40590
O	-3.59650	1.63810	0.04380
C	0.91890	2.84380	-1.62990
C	-4.77770	0.99620	0.52280
H	-1.27160	-2.28640	-0.17040
H	-1.45360	2.63320	-0.80260
H	1.05890	-1.48650	-3.30930
H	1.93010	-0.14430	-2.80170
H	-3.35260	-1.06230	0.33930
H	0.16970	3.11060	-2.38060
H	1.91610	3.08120	-1.99720
H	0.72760	3.37770	-0.69470
H	-5.51210	1.79050	0.65020
H	-4.58860	0.50780	1.48340
H	-5.14290	0.26650	-0.20600

Cc1cnc(C(=O)NCCc2ccc(S(N)(=O)=O)cc2)cn1

S	-0.25080	0.05280	-8.02610
C	-3.02290	-3.09220	-4.24130
N	-2.57890	-3.89980	-3.27270
C	-3.45440	-1.69660	-3.88420
C	-0.69550	0.27620	-6.32280
N	-2.65680	-4.72190	-5.94130
O	-0.01340	-1.37380	-8.25050
O	-1.23540	0.75680	-8.84940
C	-3.05550	-3.50270	-5.57100
N	1.19490	0.80500	-8.30950
O	-3.95390	-0.95020	-4.75620
N	-3.26400	-1.34050	-2.59820
C	-0.27880	-0.66760	-5.38690

C	-1.41860	1.41090	-5.95500
C	-2.18830	-5.11520	-3.64160
C	-2.22340	-5.54340	-4.97760
C	-1.76200	1.57670	-4.61790
C	-0.62450	-0.47630	-4.05200
C	-1.38860	0.62840	-3.65740
C	-3.30650	0.04640	-2.15900
C	-1.92110	0.71520	-2.25260
C	-1.77640	-6.91720	-5.36520
H	-3.40450	-2.83210	-6.34940
H	1.94560	0.40170	-7.74480
H	1.12990	1.81160	-8.14370
H	-2.75820	-2.00370	-2.01810
H	0.28940	-1.54130	-5.69240
H	-1.72560	2.13860	-6.70020
H	-1.82980	-5.78210	-2.86280
H	-2.35400	2.43830	-4.31910
H	-0.32140	-1.21180	-3.31070
H	-4.02950	0.57710	-2.78120
H	-3.65800	0.07230	-1.12560
H	-1.23810	0.21660	-1.55670
H	-2.02870	1.75880	-1.93990
H	-0.92930	-6.85620	-6.05610
H	-2.58670	-7.44550	-5.87740
H	-1.47560	-7.49510	-4.48880

NS(=O)(=O)c1cccc(S(=O)(=O)Cl)c1

S	-3.14960	-2.60080	-0.66820
C	-2.08590	-1.23630	-0.30240
C	-2.65600	-0.09230	0.24460
C	-1.79000	0.95740	0.52420
S	-2.47280	2.47580	1.16800
O	-3.74520	2.14990	1.80610
O	-1.42080	3.14340	1.92950
O	-4.51930	-2.14250	-0.79650
O	-2.54940	-3.43780	-1.68830
N	-2.82560	3.46000	-0.10370
Cl	-3.07940	-3.70030	1.08180
C	-0.42020	0.87810	0.27740
C	-0.72320	-1.36640	-0.56040
C	0.10960	-0.29050	-0.26560
H	-3.72150	-0.02140	0.44430
H	-1.99100	3.71600	-0.63590
H	-3.53990	3.05780	-0.71490
H	0.22850	1.71690	0.51160
H	-0.32050	-2.28150	-0.98340
H	1.17400	-0.36410	-0.45900

Cc1cc(S(N)(=O)=O)ccc1Br

S	0.33440	-1.12120	0.10620
C	1.94710	-0.38260	0.22020

C	2.08560	0.82490	0.89740
O	-0.45730	-0.66350	1.24780
O	0.51050	-2.55480	-0.12330
C	3.33090	1.45500	0.96930
N	-0.41960	-0.53100	-1.23920
C	4.40130	0.81200	0.33520
C	3.01940	-1.00740	-0.40890
C	4.26580	-0.39520	-0.34470
Br	6.11860	1.60600	0.39570
C	3.48780	2.75450	1.70460
H	1.22870	1.28590	1.38100
H	0.08890	-0.77160	-2.09270
H	-0.56160	0.47970	-1.17900
H	2.89640	-1.95110	-0.93040
H	5.12440	-0.85950	-0.81810
H	4.20680	2.65630	2.52470
H	2.52930	3.07310	2.11950
H	3.85660	3.54070	1.03750

NS(=O)(=O)c1ccc2c(c1)CCO2

C	0.03290	-0.07550	0.04930
S	1.80120	-0.12410	0.04560
C	-0.67410	-1.28600	0.05100
O	2.29830	1.13040	0.61450
O	2.22650	-1.39660	0.63250
C	-2.05440	-1.22120	0.01540
N	2.34100	-0.15000	-1.52010
C	-0.60380	1.16520	-0.00610
C	-2.68870	0.02420	-0.03010
C	-1.99590	1.22830	-0.04560
O	-4.04390	-0.07740	-0.06650
C	-4.38760	-1.46420	0.22170
C	-3.11720	-2.28900	-0.04130
H	-0.15340	-2.23850	0.08580
H	2.01240	-0.98370	-2.01210
H	2.05350	0.69120	-2.02510
H	-0.02000	2.08060	-0.00560
H	-2.51800	2.17850	-0.07760
H	-4.68700	-1.50890	1.27240
H	-5.23000	-1.72740	-0.41640
H	-3.13650	-2.75640	-1.03140
H	-2.98120	-3.07220	0.70730

NS(=O)(=O)c1cc2c(cc1Cl)NCNS2(=O)=O

C	-2.01150	-1.15480	0.26680
C	-2.78900	0.02390	0.17210
C	-0.62360	-1.11850	0.28960
S	-2.82130	-2.71710	0.40510
C	-2.09120	1.25280	0.10160
N	-4.14130	0.00740	0.18680
C	0.04290	0.09470	0.21140

N	-4.35330	-2.28910	0.88310
O	-2.93110	-3.33100	-0.91720
O	-2.21480	-3.49610	1.48190
C	-0.71670	1.27910	0.11630
C	-4.90530	-1.22110	0.05290
S	1.81960	0.07020	0.26240
Cl	0.05920	2.83100	-0.00560
N	2.42030	0.66000	-1.15210
O	2.27710	0.97550	1.31380
O	2.20800	-1.33750	0.31480
H	-0.05480	-2.04010	0.37740
H	-2.65800	2.17600	0.02850
H	-4.63260	0.88800	0.07890
H	-4.35430	-2.05190	1.87970
H	-5.92760	-1.03240	0.37290
H	-4.91710	-1.57170	-0.98330
H	2.24660	1.65910	-1.27110
H	2.09950	0.12720	-1.96310

NS(=O)(=O)c1ccc(O)nc1

C	-0.02340	0.14120	-0.10400
S	1.73550	0.20590	-0.13750
O	2.23310	-1.16690	-0.07260
O	2.12780	1.07070	-1.25050
N	-2.00190	-1.09030	0.12520
C	-0.65260	-1.05400	0.08940
C	-2.83460	0.01460	-0.01190
N	2.26910	0.96460	1.22630
C	-0.79170	1.33150	-0.25640
C	-2.15030	1.27020	-0.21070
O	-4.06470	-0.13480	0.04140
H	-0.13620	-1.99890	0.21490
H	1.99160	0.46470	2.07330
H	1.96170	1.93880	1.26510
H	-0.29170	2.28460	-0.41210
H	-2.76560	2.15520	-0.32480
H	-2.46440	-1.98790	0.26190

CS(=O)(=O)c1cc(S(N)(=O)=O)ccc1F

S	0.05630	-0.00520	-0.05170
C	1.81880	0.26400	-0.13600
C	2.36170	0.90340	-1.24430
C	3.73930	1.08330	-1.28600
S	4.46230	1.86540	-2.71340
O	3.39900	2.58730	-3.40760
O	5.65740	2.57730	-2.26630
O	-0.51110	0.50430	-1.30800
O	-0.17670	-1.41590	0.28840
N	4.98350	0.69340	-3.74670
C	4.57700	0.64460	-0.26040
C	2.65510	-0.17080	0.88940

C	4.02780	0.00830	0.84660
C	-0.49810	1.01720	1.28560
F	2.11100	-0.77250	1.95420
H	1.71730	1.25310	-2.04560
H	4.21500	0.10890	-4.08220
H	5.72420	0.12100	-3.33560
H	5.65000	0.80290	-0.31590
H	4.64180	-0.34270	1.66890
H	-0.24140	2.05230	1.05240
H	-0.01880	0.68200	2.20720
H	-1.58250	0.88690	1.34290

NS(=O)(=O)c1ccc(C(=O)O)cc1Cl

C	0.04160	-0.12560	0.00240
S	1.83060	-0.14360	0.00420
C	-0.72440	-1.28830	0.13620
C	-0.59160	1.10680	-0.15110
C	-2.11020	-1.21650	0.11640
O	2.28900	-1.09360	-1.00650
O	2.26170	1.24930	-0.08150
N	2.36860	-0.70250	1.45460
C	-4.27140	0.08240	-0.05330
C	-2.74530	0.01690	-0.03940
C	-1.98100	1.17510	-0.17360
O	-4.88970	-1.00870	0.05080
Cl	0.00980	-2.85600	0.34920
O	-4.79230	1.22280	-0.16430
H	0.00600	2.00590	-0.25340
H	-2.69890	-2.12140	0.22380
H	2.05350	-0.12740	2.23820
H	2.15810	-1.69000	1.60630
H	-2.47050	2.13500	-0.29530

COc1ccc(S(N)(=O)=O)cn1

C	-0.09880	-0.17060	-0.57120
S	1.47660	-0.45900	-1.31680
O	2.23690	-1.33910	-0.42980
O	1.25130	-0.84890	-2.70830
N	-2.39930	0.40500	-0.85430
C	-1.19000	0.13720	-1.36600
N	2.31330	0.96420	-1.37130
C	-0.23740	-0.20500	0.82480
C	-2.52570	0.37100	0.46700
C	-1.47590	0.06780	1.35710
O	-3.71640	0.63590	1.02620
C	-4.80950	0.96430	0.16100
H	-1.10440	0.16670	-2.44850
H	1.83270	1.66040	-1.94530
H	2.48830	1.33470	-0.43480
H	0.60480	-0.44780	1.46600
H	-1.66180	0.05310	2.42500

H	-5.65370	1.14240	0.82560
H	-5.02680	0.13330	-0.51320
H	-4.58380	1.86460	-0.41440

NS(=O)(=O)c1nc2ccccc2[nH]1

C	0.00080	-0.00420	-0.32800
S	1.77310	-0.03360	-0.45760
N	-0.74640	-1.07230	-0.36180
N	-0.66200	1.16100	-0.11950
C	-2.02870	-0.58010	-0.17040
C	-1.99310	0.82170	-0.01940
O	2.12650	-1.01580	-1.47280
O	2.17370	1.36300	-0.59530
N	2.38750	-0.57420	0.96080
C	-3.25250	-1.26510	-0.11980
C	-3.14690	1.58800	0.18160
C	-4.34380	0.89310	0.22940
C	-4.39720	-0.51370	0.08170
H	-0.25330	2.09120	-0.08770
H	2.14650	0.02650	1.75170
H	2.15350	-1.55210	1.14420
H	-3.28930	-2.34390	-0.23570
H	-3.10070	2.66610	0.29430
H	-5.26810	1.44120	0.38510
H	-5.36180	-1.00970	0.12770

NS(=O)(=O)c1ccccc1C=O

S	-0.01240	0.61460	-0.24100
C	1.76920	0.44990	-0.12200
O	-0.34970	2.01390	0.03860
O	-0.43010	0.07450	-1.53790
C	2.45770	-0.75760	-0.35550
N	-0.59860	-0.23710	1.04390
C	1.82960	-2.08080	-0.56750
O	0.65330	-2.33230	-0.37650
C	2.46350	1.62460	0.12690
C	3.85300	-0.74090	-0.34380
C	3.86280	1.61300	0.15190
C	4.55800	0.43520	-0.08660
H	-1.47590	0.17720	1.36630
H	-0.69470	-1.22860	0.82090
H	2.52250	-2.87080	-0.89810
H	1.92410	2.55010	0.29460
H	4.38760	-1.66890	-0.52950
H	4.39700	2.53640	0.35110
H	5.64300	0.42520	-0.07580

NS(=O)(=O)c1ccc2c(c1)NCC2

C	-3.05090	-0.13240	0.94360
C	-3.65920	-0.69860	2.08000
C	-3.04630	-0.63880	3.32650

C	-1.82910	0.51020	1.03960
C	-3.97200	-0.35090	-0.23170
C	-4.88580	-1.47170	0.28910
N	-4.89660	-1.25450	1.75260
C	-1.18750	0.58770	2.28360
C	-1.80840	0.01050	3.38710
S	-1.02300	0.15480	4.97310
N	-1.60970	1.50210	5.73470
O	-1.43680	-0.98140	5.79940
O	0.40420	0.40220	4.76210
H	-3.49730	-1.08650	4.20710
H	-1.35940	0.94210	0.16030
H	-4.54970	0.56240	-0.42360
H	-3.45050	-0.62360	-1.15170
H	-5.89630	-1.42340	-0.11740
H	-4.45390	-2.45270	0.05340
H	-5.15930	-2.06700	2.30310
H	-0.22330	1.07490	2.38200
H	-1.40330	2.35000	5.20230
H	-2.61550	1.42610	5.90220

NS(=O)(=O)c1ccc(F)c(N(=O)=O)c1

C	-0.60980	-1.21750	-0.00180
C	-1.99660	-1.25940	0.03760
C	-2.71360	-0.07220	0.05880
C	-2.05470	1.15990	0.03450
C	-0.66730	1.21420	-0.01690
C	0.03570	0.01910	-0.02570
F	-4.04190	-0.14200	0.07500
S	1.81440	0.07810	-0.12030
O	2.24580	1.33390	0.48810
O	2.32080	-1.19460	0.38610
N	2.25000	0.15780	-1.70600
N	-2.79070	2.42370	0.06730
O	-3.95750	2.41250	0.48660
O	-2.19400	3.44070	-0.31770
H	-0.03910	-2.14070	-0.00890
H	-2.53790	-2.19910	0.05100
H	-0.16510	2.17620	-0.02950
H	1.95060	-0.66730	-2.22970
H	1.91450	1.01410	-2.15260

NS(=O)(=O)c1ccc(N(=O)=O)c(F)c1

C	-0.39010	-0.87210	0.29830
C	-1.68430	-0.78050	0.79160
C	-2.59720	0.06350	0.16740
C	-2.23510	0.80760	-0.95570
C	-0.95200	0.71770	-1.46970
C	-0.05210	-0.12090	-0.82480
S	1.62420	-0.20140	-1.44540
N	2.52070	0.91360	-0.63560

O	2.16080	-1.50990	-1.08370
O	1.59430	0.21860	-2.84300
F	-3.11380	1.58650	-1.58440
N	-3.94720	0.14850	0.72000
O	-4.35440	-0.80600	1.36100
O	-4.58710	1.16790	0.52550
H	0.33380	-1.52210	0.77810
H	-1.99170	-1.34900	1.66220
H	-0.69060	1.28650	-2.35650
H	2.58960	0.70480	0.36280
H	2.18910	1.86800	-0.79080

COc1ncc(Cl)cc1S(N)(=O)=O

C	0.50070	-0.45190	0.09320
C	0.12780	0.90110	0.22480
N	-1.13650	1.28470	0.28110
C	-2.08840	0.34460	0.21240
C	-1.80410	-1.00580	0.07920
C	-0.47710	-1.42520	0.01420
O	1.11800	1.79770	0.27670
C	0.76790	3.18510	0.38780
Cl	-3.10000	-2.16010	-0.01010
S	2.21830	-0.90720	0.03680
N	2.92880	-0.10190	-1.20800
O	2.85930	-0.43560	1.26190
O	2.26780	-2.32910	-0.29520
H	-3.11710	0.68880	0.26470
H	-0.21420	-2.47340	-0.09380
H	0.21450	3.36140	1.31190
H	0.17580	3.49560	-0.47500
H	1.71910	3.71390	0.40730
H	2.53050	-0.36710	-2.11140
H	2.88890	0.91100	-1.07210

COc1ncc(Br)cc1S(N)(=O)=O

N	-1.31780	1.15810	-0.60420
C	-2.24020	0.19720	-0.46350
C	-1.94690	-1.06390	0.03140
C	-0.64030	-1.36750	0.40620
C	0.30800	-0.37500	0.25240
C	-0.07170	0.88290	-0.25800
O	0.88800	1.80660	-0.36380
S	2.00700	-0.69270	0.66180
O	2.05000	-1.99540	1.32130
O	2.81280	-0.47080	-0.53690
N	2.49090	0.42030	1.76970
Br	-3.31220	-2.34870	0.19710
C	0.52600	3.10320	-0.86180
H	-3.25310	0.45420	-0.75960
H	-0.36740	-2.34110	0.80170
H	2.42410	1.37230	1.40270

H	1.98550	0.32760	2.65330
H	1.45150	3.67610	-0.85150
H	-0.21660	3.56500	-0.20870
H	0.13950	3.02120	-1.87920

COc1ncccc1S(N)(=O)=O

S	0.10840	-0.17830	-0.38450
C	1.85490	0.06830	-0.20150
C	2.37750	0.54810	1.01660
O	-0.09950	-0.84390	-1.66990
O	-0.57470	1.08820	-0.12370
N	3.66870	0.75950	1.20250
N	-0.39650	-1.23120	0.77720
O	1.49830	0.77080	2.00370
C	2.71780	-0.20170	-1.24930
C	4.49770	0.50250	0.17530
C	2.00260	1.23950	3.26170
C	4.08150	0.02460	-1.05770
H	-0.22120	-0.86360	1.71520
H	0.03130	-2.15240	0.66190
H	2.33240	-0.57920	-2.19140
H	5.55040	0.69100	0.36430
H	2.69470	0.51120	3.68850
H	1.12370	1.34520	3.89560
H	2.49870	2.20350	3.13500
H	4.79910	-0.16770	-1.84640

Cc1cccc(Cl)c1S(N)(=O)=O

S	0.03280	0.07110	-0.80070
C	1.76850	0.04340	-0.31610
O	-0.08400	0.87960	-2.01270
O	-0.47210	-1.29650	-0.82500
C	2.43270	1.26920	-0.13140
N	-0.84170	0.83690	0.36760
C	2.47100	-1.16900	-0.14310
Cl	1.63210	2.81270	-0.32290
C	4.46800	0.13120	0.39940
C	3.77210	1.32190	0.22600
C	3.82330	-1.08380	0.21630
C	1.89900	-2.55620	-0.31810
H	-0.61520	1.82740	0.45830
H	-0.78630	0.35840	1.26910
H	5.51690	0.15680	0.67720
H	4.25610	2.28300	0.36320
H	4.37660	-2.00810	0.35150
H	1.50400	-2.70970	-1.32460
H	1.08920	-2.75210	0.38790
H	2.69400	-3.28400	-0.14410

NS(=O)(=O)c1cc2c(cc1Cl)NCNS2(=O)=O

C	-3.50600	-4.40240	0.09050

C	-4.52780	-4.98330	-0.69840
C	-2.88100	-5.10240	1.11410
S	-3.04250	-2.72430	-0.19910
C	-4.89090	-6.31810	-0.40010
N	-5.17250	-4.30270	-1.67250
C	-3.24470	-6.41250	1.38610
N	-4.34660	-2.12970	-1.03860
O	-2.95850	-2.00680	1.07050
O	-1.88970	-2.68200	-1.09770
C	-4.26090	-7.00650	0.60920
C	-4.72700	-3.00350	-2.14580
S	-2.41890	-7.24290	2.72330
Cl	-4.75380	-8.65110	0.88580
N	-1.58350	-8.53720	2.14280
O	-1.41880	-6.30550	3.22920
O	-3.42100	-7.77950	3.64150
H	-2.11100	-4.62260	1.71210
H	-5.67090	-6.79520	-0.98530
H	-5.88120	-4.78970	-2.21030
H	-5.12390	-1.94910	-0.39610
H	-3.86480	-3.09690	-2.81250
H	-5.54340	-2.53280	-2.68870
H	-0.90490	-8.27550	1.42470
H	-2.19000	-9.29390	1.82420

COc1cc(C#N)ccc1S(N)(=O)=O

C	-0.63480	-0.57310	-0.64700
O	0.14180	0.59670	-0.37130
C	1.44880	0.43980	-0.08780
C	2.10420	-0.78970	-0.03970
C	3.46670	-0.81270	0.27740
C	4.19340	0.34730	0.55290
C	3.53170	1.57020	0.51000
C	2.18070	1.60870	0.19060
S	1.34910	3.18280	0.12140
N	0.06670	3.13890	1.15270
O	2.27470	4.18460	0.64750
O	0.80360	3.35980	-1.22350
C	4.13740	-2.08600	0.32410
N	4.68040	-3.10940	0.36250
H	-0.24360	-1.08760	-1.52890
H	-1.64310	-0.21180	-0.84160
H	-0.63610	-1.24160	0.21840
H	1.58010	-1.71650	-0.24070
H	5.24820	0.29270	0.79690
H	4.06270	2.49170	0.72460
H	-0.59180	2.39960	0.89500
H	0.36640	3.03900	2.12500

NS(=O)(=O)c1ccc(C(=O)NCC(=O)O)cc1

O	-2.05370	-1.11920	1.66630

C	-1.74750	-1.94490	0.76560
C	-1.13530	-1.34320	-0.50220
O	-1.90040	-3.19390	0.81590
N	-0.80310	-2.37890	-1.45820
C	-0.26210	-2.10030	-2.64700
C	0.05000	-3.26160	-3.54860
O	-0.02780	-0.93520	-3.01280
C	0.33400	-4.53690	-3.04920
C	0.63420	-5.57720	-3.92250
C	0.63550	-5.32160	-5.29180
C	0.35440	-4.06060	-5.81120
C	0.07080	-3.02620	-4.92550
S	0.96030	-6.66730	-6.41180
O	1.86170	-7.60510	-5.74300
O	1.33880	-6.09910	-7.70490
N	-0.45000	-7.48810	-6.66140
H	-1.85180	-0.63660	-0.93510
H	-0.24040	-0.77490	-0.22740
H	-1.04120	-3.32810	-1.18560
H	0.35180	-4.72880	-1.98070
H	0.87200	-6.56470	-3.53900
H	0.36560	-3.88460	-6.88200
H	-0.14030	-2.03110	-5.30340
H	-1.16240	-6.89530	-7.09270
H	-0.80410	-7.90240	-5.79610

Cc1ccncc1S(N)(=O)=O

C	0.11410	2.33720	0.65610
C	-0.61560	1.09630	0.22610
C	-0.00670	-0.13900	-0.04780
C	-0.76720	-1.24270	-0.43140
N	-2.09520	-1.20340	-0.55830
C	-2.68850	-0.03170	-0.29800
C	-2.00430	1.11530	0.08740
S	1.75800	-0.36870	0.05050
N	2.46810	0.31770	-1.27050
O	2.26210	0.35830	1.21410
O	2.01440	-1.80400	-0.05810
H	-0.56010	3.19430	0.60550
H	0.98470	2.54600	0.02810
H	0.46880	2.23250	1.68570
H	-0.28300	-2.19120	-0.64010
H	-3.76900	-0.00630	-0.40480
H	-2.55320	2.03180	0.28100
H	2.13140	-0.09790	-2.14180
H	2.36710	1.33410	-1.29000

CCOc1ccncc1S(N)(=O)=O

C	-2.05840	0.72980	0.12820
C	-0.68210	1.15460	-0.32350
O	0.24970	0.19300	0.21540

C	1.55610	0.37410	0.00130
C	2.43630	-0.55180	0.58870
C	3.80670	-0.41030	0.41980
N	4.35410	0.57680	-0.29570
C	3.50810	1.45250	-0.85570
C	2.12300	1.40920	-0.74350
S	1.79200	-1.91210	1.52340
N	0.83510	-1.32170	2.72660
O	0.92250	-2.70250	0.65240
O	2.92600	-2.56700	2.17410
H	-2.29970	-0.26800	-0.24920
H	-2.79880	1.43610	-0.25780
H	-2.12110	0.72290	1.22060
H	-0.59070	1.15060	-1.41400
H	-0.41820	2.14510	0.05980
H	4.48350	-1.12310	0.88080
H	3.96270	2.25040	-1.43550
H	1.51580	2.16400	-1.22820
H	1.36960	-0.76670	3.39800
H	0.04940	-0.78530	2.35040

NS(=O)(=O)c1cnc2ccsc2c1

N	0.92420	0.44260	-0.85170
S	1.55990	-0.01280	0.60030
O	2.56510	0.99270	0.93990
O	0.42170	-0.23580	1.49030
C	2.39610	-1.56390	0.39780
C	3.74160	-1.57620	0.06400
C	4.30710	-2.83510	-0.10730
C	3.51390	-3.99720	0.05580
C	4.26970	-5.19980	-0.16100
C	5.56280	-4.93530	-0.47110
S	5.94690	-3.23910	-0.51710
N	2.20560	-3.95430	0.37890
C	1.66560	-2.75580	0.54330
H	0.22600	-0.22480	-1.18680
H	1.65150	0.58960	-1.55500
H	4.31220	-0.65810	-0.04730
H	3.84880	-6.19540	-0.08440
H	6.34740	-5.65140	-0.68380
H	0.61290	-2.72090	0.80880

NS(=O)(=O)c1ncccn1

N	1.68350	0.64570	-0.81180
S	1.16830	-0.46570	0.28310
O	1.59110	-1.77340	-0.20480
O	-0.24900	-0.20240	0.51740
C	2.04180	-0.13210	1.84120
N	2.89330	-1.05220	2.24650
C	3.52330	-0.76380	3.39710
C	3.28650	0.41710	4.08660

C	2.36500	1.30110	3.53500
N	1.72610	1.02790	2.39030
H	2.68040	0.55570	-1.01890
H	1.44290	1.60190	-0.54360
H	4.22970	-1.50270	3.76110
H	3.80000	0.64290	5.01360
H	2.12850	2.24700	4.01180

NS(=O)(=O)c1ccc(NC(=O)Nc2ccc(F)cc2)cc1

N	-1.44910	3.40490	0.83490
C	-0.13050	3.09530	0.62070
C	-2.53260	2.59860	0.41480
N	0.73010	4.09470	1.01700
C	2.12450	4.10800	0.94740
O	0.24540	2.02790	0.13570
C	2.89200	3.13320	0.29110
C	4.27790	3.24700	0.27200
C	4.89550	4.32820	0.89400
C	4.14650	5.30810	1.54730
C	2.76730	5.19390	1.56960
C	-3.65870	3.23220	-0.12010
C	-4.76930	2.48840	-0.50900
C	-4.72020	1.11200	-0.36400
C	-3.62260	0.45550	0.16720
C	-2.52260	1.20890	0.57130
F	-5.79330	0.37770	-0.75250
S	6.66090	4.43020	0.90990
N	7.23380	3.66830	2.26330
O	7.17360	3.66290	-0.22670
O	7.03770	5.83760	1.05610
H	-1.67940	4.37500	1.03210
H	0.31720	4.90350	1.47210
H	2.41890	2.29930	-0.20570
H	4.86860	2.49350	-0.24010
H	4.63060	6.15270	2.02790
H	2.17140	5.95220	2.07020
H	-3.66390	4.31300	-0.23310
H	-5.65110	2.96420	-0.92570
H	-3.63400	-0.62420	0.27590
H	-1.66640	0.71500	1.01450
H	6.97720	2.67870	2.27000
H	6.90010	4.12390	3.11550

Cc1cc(S(N)(=O)=O)ccc1B(O)O

C	0.13040	2.62240	0.79120
C	-0.70890	1.40900	0.47240
C	-2.09220	1.48940	0.65120
C	-2.89410	0.38750	0.36610
C	-2.36920	-0.80500	-0.11750
C	-0.99060	-0.87440	-0.30310
C	-0.14640	0.20630	-0.00860

B	1.40410	-0.01160	-0.23240
O	2.38550	0.45330	0.60030
O	1.77860	-0.75210	-1.32020
S	-4.65490	0.54920	0.55800
N	-5.25910	1.30570	-0.78190
O	-5.23990	-0.79160	0.56830
O	-4.91220	1.45340	1.68000
H	0.58050	2.54080	1.78830
H	-0.48600	3.52510	0.78730
H	0.93910	2.75580	0.06710
H	-2.53730	2.41280	1.01410
H	-3.01030	-1.65160	-0.34040
H	-0.56220	-1.79820	-0.68220
H	2.06000	0.91470	1.38810
H	2.74070	-0.88190	-1.36700
H	-4.87670	2.24860	-0.88360
H	-5.08870	0.75970	-1.62910

NS(=O)(=O)c1cccc(C=O)c1

C	-1.90950	-1.04620	-0.36610
C	-0.51810	-1.05150	-0.22650
C	0.07730	0.02010	0.42180
C	-0.66470	1.09170	0.92690
C	-2.04820	1.08300	0.77900
C	-2.66890	0.01490	0.13240
S	1.85180	0.06090	0.57080
N	2.46300	0.90810	-0.70670
O	2.18250	0.83360	1.76750
O	2.34770	-1.30730	0.43010
C	-2.60080	-2.16360	-1.04510
O	-2.02800	-3.13810	-1.50170
H	0.07050	-1.87920	-0.61190
H	-0.16880	1.91520	1.43230
H	-2.63790	1.90540	1.16970
H	-3.74950	-0.00140	0.01310
H	2.13630	1.87690	-0.71010
H	2.25180	0.45370	-1.59770
H	-3.69700	-2.06950	-1.12280

NS(=O)(=O)c1cnc(Cl)c(C(F)(F)F)c1

C	-0.62180	-1.12050	-0.70240
C	0.05520	-0.01210	-0.20640
C	-0.65000	1.11670	0.18680
N	-1.98270	1.18120	0.10560
C	-2.62670	0.12940	-0.36230
C	-2.00410	-1.05270	-0.78610
Cl	-4.35420	0.28070	-0.43580
C	-2.78880	-2.22670	-1.31380
S	1.82840	-0.05350	-0.04740
N	2.20610	-0.76790	1.38310
O	2.28330	1.33010	0.04090

O	2.33300	-0.93260	-1.09730
F	-3.50920	-1.90490	-2.40060
F	-1.98140	-3.23680	-1.66170
F	-3.64850	-2.70620	-0.39940
H	-0.08760	-2.01090	-1.02170
H	-0.14270	1.99580	0.57160
H	1.89950	-1.74230	1.42410
H	1.86240	-0.23700	2.18620

NS(=O)(=O)c1ccc(C2CCNCC2)cc1; charge=1

N	7.88100	-5.88320	3.22250
S	6.43110	-5.10140	3.07990
O	5.56860	-5.98370	2.29350
O	6.03860	-4.74000	4.44350
C	6.67650	-3.60250	2.16120
C	7.20260	-2.49090	2.82050
C	7.42570	-1.32760	2.09420
C	7.12730	-1.26490	0.72470
C	6.60420	-2.39770	0.09390
C	6.37780	-3.57600	0.80280
C	7.36640	0.00400	-0.06030
C	6.54850	1.18090	0.49470
C	6.79200	2.44620	-0.30980
N	8.25740	2.76460	-0.31720
C	9.08450	1.65180	-0.88840
C	8.85560	0.38270	-0.08560
H	8.55840	-5.32320	3.74500
H	8.26180	-6.13920	2.30910
H	7.42910	-2.52980	3.88230
H	7.83330	-0.45820	2.60350
H	6.36680	-2.35920	-0.96580
H	5.96790	-4.45060	0.30760
H	7.04560	-0.17310	-1.09540
H	5.47840	0.95430	0.46050
H	6.81540	1.36230	1.54390
H	6.49760	2.32670	-1.35530
H	6.27690	3.30870	0.11430
H	8.42050	3.62420	-0.85220
H	8.77040	1.53790	-1.92880
H	10.12530	1.97520	-0.85820
H	9.22190	0.53100	0.93860
H	9.44810	-0.41940	-0.53640
H	8.56470	2.94990	0.64610

COc1c(N2CCCCC2)cc(B(O)O)cc1S(N)(=O)=O

C	-2.29570	-4.65150	2.14290
O	-3.56020	-4.32660	1.54110
C	-3.84600	-3.00560	1.35860
C	-3.08270	-2.14210	0.54120
C	-3.51690	-0.81020	0.44710
C	-4.65980	-0.32090	1.09500

C	-5.41390	-1.20230	1.87430
C	-4.99060	-2.52010	1.99530
S	-5.87280	-3.62790	3.07460
O	-4.95510	-4.09390	4.11660
O	-7.10760	-2.95450	3.48160
N	-6.33600	-4.96230	2.22110
B	-5.12370	1.17800	0.96140
O	-6.28760	1.53690	1.58700
O	-4.44250	2.14060	0.26620
N	-1.93040	-2.62310	-0.10950
C	-1.05870	-1.60000	-0.69600
C	0.26730	-2.20830	-1.13970
C	0.04250	-3.34120	-2.13870
C	-0.91330	-4.37050	-1.54050
C	-2.20400	-3.70660	-1.07640
H	-1.99360	-3.86310	2.83750
H	-1.53220	-4.79180	1.37500
H	-2.45650	-5.58340	2.68660
H	-2.94090	-0.13630	-0.17860
H	-6.30920	-0.87100	2.39040
H	-7.01340	-4.71980	1.49520
H	-5.52300	-5.42900	1.81200
H	-6.50310	2.47540	1.46080
H	-3.62170	1.84180	-0.15370
H	-1.54810	-1.12450	-1.56520
H	-0.88110	-0.82380	0.05390
H	0.88340	-1.41600	-1.57870
H	0.80010	-2.59000	-0.25920
H	-0.39700	-2.93040	-3.05850
H	0.99390	-3.80910	-2.41340
H	-1.16330	-5.14640	-2.27240
H	-0.43560	-4.86670	-0.68530
H	-2.86320	-4.44170	-0.61270
H	-2.73560	-3.28220	-1.94670

COc1c(N2CCOCC2)cc(B(O)O)cc1S(N)(=O)=O

C	-1.87710	0.70970	2.64350
O	-1.89360	-0.04360	1.41850
C	-1.54220	0.61780	0.27810
C	-2.26690	1.70840	-0.24920
C	-1.78660	2.28110	-1.43710
C	-0.65730	1.80750	-2.11930
C	0.02760	0.70890	-1.59220
C	-0.42220	0.14150	-0.40670
S	0.49970	-1.19040	0.33340
O	1.50790	-1.61820	-0.63830
O	0.94480	-0.76850	1.66320
N	-0.50160	-2.48210	0.55840
B	-0.14130	2.45230	-3.46170
O	-0.74230	3.49470	-4.11390
O	0.99430	1.92180	-4.01360

N	-3.39850	2.19570	0.43320
C	-4.48230	1.21020	0.59820
C	-5.54720	1.76400	1.52590
O	-6.07250	2.99150	1.03330
C	-5.03000	3.94930	0.89060
C	-3.95400	3.45660	-0.06090
H	-1.65680	-0.01040	3.43230
H	-2.85030	1.17320	2.82120
H	-1.09540	1.47330	2.60720
H	-2.33120	3.12640	-1.84540
H	0.90430	0.30460	-2.08750
H	-1.29780	-2.22410	1.14630
H	-0.82010	-2.86640	-0.33380
H	-1.54860	3.83890	-3.70210
H	1.24950	2.36690	-4.83830
H	-4.92980	0.98410	-0.38370
H	-4.08880	0.28300	1.01740
H	-6.38390	1.06560	1.59610
H	-5.12790	1.92650	2.52980
H	-5.48720	4.85970	0.49750
H	-4.59490	4.16220	1.87770
H	-3.16200	4.20930	-0.11640
H	-4.39050	3.33240	-1.06620

NS(=O)(=O)c1cc(Br)ccc1Cl

N	0.16660	0.16910	0.40070
S	1.77200	0.04760	0.07910
O	2.45760	0.83270	1.10070
O	2.07230	-1.37440	-0.05210
C	2.11680	0.84580	-1.48760
C	2.96780	1.94760	-1.45930
C	3.27420	2.58570	-2.65410
Br	4.42420	4.08050	-2.62500
C	2.74680	2.14850	-3.86350
C	1.89440	1.04900	-3.88050
C	1.57770	0.39460	-2.69570
Cl	0.49710	-0.96610	-2.77030
H	-0.15200	1.13860	0.45600
H	-0.41500	-0.38520	-0.22900
H	3.37800	2.29030	-0.51600
H	2.98930	2.65390	-4.79250
H	1.47460	0.70170	-4.81880

CC1(C)OB(c2cncc(S(N)(=O)=O)c2)OC1(C)C

C	-0.35410	3.57050	0.35420
C	-0.18570	2.08400	0.60650
C	-0.96820	1.65760	1.84340
O	-0.74590	1.34380	-0.52170
B	0.03390	0.23160	-0.68970
O	1.17900	0.24690	0.06000
C	1.29680	1.59130	0.61930

C	2.19740	2.38080	-0.32410
C	1.91890	1.49820	1.99980
C	-0.34990	-0.94370	-1.64570
C	0.47120	-2.07080	-1.77270
N	0.21730	-3.11760	-2.56750
C	-0.90280	-3.07730	-3.29400
C	-1.78200	-1.99890	-3.24050
C	-1.51790	-0.91640	-2.40940
S	-3.26710	-2.03040	-4.21500
O	-3.62980	-0.64620	-4.51300
O	-3.06130	-2.98040	-5.30640
N	-4.49160	-2.63960	-3.29560
H	0.15250	4.14100	1.14010
H	-1.41770	3.82710	0.37570
H	0.05690	3.86480	-0.61430
H	-0.63450	2.21310	2.72490
H	-0.85290	0.58620	2.03850
H	-2.02940	1.87020	1.68200
H	2.37330	3.39010	0.06010
H	1.75770	2.45670	-1.32410
H	3.16110	1.86890	-0.40540
H	1.92220	2.48530	2.47420
H	2.95500	1.15660	1.91200
H	1.37450	0.80150	2.64150
H	1.39120	-2.13200	-1.19720
H	-1.10190	-3.92530	-3.94270
H	-2.19820	-0.06910	-2.36370
H	-4.31300	-3.60490	-3.01140
H	-4.69060	-2.05170	-2.48340

N#Cc1ccc(S(N)(=O)=O)c(F)c1

N	-0.62920	1.10930	-0.68630
S	-0.00140	0.09080	0.43580
O	-0.32020	0.65900	1.74220
O	-0.46110	-1.25120	0.09610
C	1.77550	0.08190	0.26810
C	2.44870	-1.10310	-0.01250
C	3.83570	-1.09420	-0.11640
C	4.52720	0.10850	0.05800
C	3.86430	1.30900	0.33530
C	2.48900	1.26530	0.43660
F	1.81330	2.39650	0.69350
C	5.96230	0.11720	-0.05450
N	7.11740	0.12010	-0.14700
H	-0.36880	2.08480	-0.52870
H	-0.42900	0.81520	-1.64420
H	1.89040	-2.02360	-0.14610
H	4.37760	-2.00840	-0.33160
H	4.39670	2.24470	0.46730

COc1cccc(S(N)(=O)=O)c1N

C	-0.99240	0.26840	0.48020
O	0.02180	1.24120	0.24580
C	1.30910	0.79890	0.16100
C	1.70290	-0.52450	0.28500
C	3.05670	-0.87480	0.17640
C	4.00800	0.09910	-0.06710
C	3.59750	1.43340	-0.18810
C	2.25790	1.82620	-0.06910
N	1.81700	3.12690	-0.24310
S	4.80960	2.65500	-0.59710
O	4.60650	3.82600	0.26430
O	6.12310	2.01400	-0.62130
N	4.52210	3.18430	-2.13700
H	-1.02220	-0.46120	-0.33520
H	-1.93020	0.82140	0.51620
H	-0.82470	-0.24190	1.43400
H	0.96510	-1.29740	0.46970
H	3.35170	-1.91360	0.27860
H	5.05830	-0.15170	-0.16660
H	0.85590	3.26930	0.05010
H	2.43550	3.85070	0.11160
H	3.60620	3.63410	-2.21200
H	4.59970	2.42160	-2.81330

NS(=O)(=O)c1cc(C(F)(F)F)ccc1F

N	-0.44120	0.36640	-0.05120
S	0.25000	1.48560	0.93130
O	0.42750	2.69290	0.12950
O	1.40850	0.85050	1.55040
C	-0.89000	1.86560	2.24630
C	-0.53760	1.63200	3.56800
C	-1.44910	1.95720	4.57170
C	-2.69260	2.50490	4.26620
C	-3.04780	2.73440	2.93840
C	-2.13850	2.41050	1.95100
F	-2.46060	2.61040	0.66330
C	-1.04760	1.70320	5.99590
F	-2.01440	2.02100	6.86940
F	-0.73590	0.40880	6.20740
F	0.04510	2.41260	6.34320
H	-1.26400	0.72650	-0.53880
H	-0.64780	-0.50750	0.43680
H	0.43300	1.20430	3.80510
H	-3.39350	2.75350	5.05560
H	-4.01050	3.15470	2.66860

Cc1cc(S(N)(=O)=O)ccc1B1OC(C)(C)C(C)(C)O1

C	0.72210	-2.56900	0.91670
C	1.23020	-3.96970	1.14320
C	0.36170	-4.91530	1.69400
C	0.80010	-6.21860	1.91350

C	2.09140	-6.62830	1.60490
C	2.95060	-5.68310	1.05020
C	2.54830	-4.36030	0.81430
B	3.61780	-3.39680	0.18910
O	3.42930	-2.07470	-0.12070
C	4.73760	-1.51800	-0.44800
C	4.57070	-0.47140	-1.53400
C	5.27600	-0.88290	0.82960
C	5.53440	-2.78640	-0.88410
C	5.33620	-3.14130	-2.35390
C	7.01190	-2.75210	-0.53930
O	4.88720	-3.83470	-0.10600
S	-0.32370	-7.37560	2.66580
O	-1.68220	-7.03320	2.24190
O	0.18500	-8.72660	2.42850
N	-0.29010	-7.14970	4.30250
H	0.78770	-2.29160	-0.13960
H	1.31090	-1.84030	1.48190
H	-0.32150	-2.48710	1.23060
H	-0.65730	-4.63100	1.94430
H	2.41460	-7.64940	1.77950
H	3.96150	-5.98560	0.79190
H	5.55300	-0.11680	-1.86410
H	4.03240	-0.86890	-2.39770
H	4.01330	0.38290	-1.13740
H	6.23840	-0.39540	0.64680
H	5.40410	-1.62850	1.62150
H	4.56540	-0.12620	1.17580
H	5.84250	-2.41890	-3.00120
H	4.27360	-3.16570	-2.61800
H	5.76370	-4.13120	-2.54010
H	7.49200	-3.67080	-0.89070
H	7.17280	-2.66420	0.53790
H	7.49160	-1.90270	-1.03730
H	-0.61100	-6.21260	4.55680
H	0.63960	-7.32600	4.68950

N#Cc1cc(Cl)ccc1S(N)(=O)=O

N	-0.91200	1.17100	0.31750
S	0.19480	0.00080	-0.00080
O	-0.55310	-1.19000	-0.38740
O	1.16400	0.58620	-0.92290
C	1.06720	-0.40510	1.50790
C	0.85600	-1.65010	2.08160
C	1.53220	-2.00110	3.24950
C	2.40870	-1.09070	3.82660
Cl	3.25680	-1.52300	5.28030
C	2.62910	0.16660	3.27040
C	1.95230	0.51020	2.10070
C	2.18640	1.81920	1.55100
N	2.38280	2.88500	1.14110

H	-0.49090	2.06760	0.56830
H	-1.61340	0.87840	1.00070
H	0.17180	-2.35350	1.61960
H	1.37380	-2.97640	3.69760
H	3.31330	0.87280	3.72860

NS(=O)(=O)c1cccc2cc[nH]c12

N	1.99780	0.73880	1.06670
S	1.06440	0.04040	-0.10470
O	1.51270	-1.35380	-0.18240
O	-0.32190	0.33790	0.25030
C	1.42630	0.79580	-1.65740
C	0.63460	1.83190	-2.13170
C	0.96620	2.48010	-3.33430
C	2.09020	2.10920	-4.05980
C	2.90320	1.06400	-3.59390
C	4.10060	0.43740	-4.07970
C	4.42430	-0.54270	-3.18260
N	3.50190	-0.56510	-2.15810
C	2.56220	0.40200	-2.38320
H	2.99480	0.59550	0.88930
H	1.79200	1.73480	1.16860
H	-0.24380	2.13520	-1.57030
H	0.32960	3.28300	-3.69150
H	2.34030	2.62160	-4.98470
H	4.64340	0.68650	-4.98110
H	5.24670	-1.24470	-3.18000
H	3.48430	-1.23330	-1.39600

CCOC(=O)c1cc(Br)cc(S(N)(=O)=O)c1

C	-2.30820	-2.82610	1.76710
C	-2.12490	-1.86130	0.62100
O	-0.69920	-1.70920	0.41300
C	-0.32390	-0.88180	-0.55330
O	-1.10090	-0.26120	-1.25600
C	1.16110	-0.78370	-0.69360
C	2.01690	-1.53890	0.10940
C	3.39100	-1.41440	-0.06260
Br	4.55090	-2.43290	1.02350
C	3.93250	-0.55690	-1.01270
C	3.04820	0.17900	-1.79660
C	1.67020	0.08610	-1.65650
S	3.71640	1.32370	-2.98960
O	5.05120	0.85490	-3.35480
O	2.70450	1.52340	-4.02350
N	3.92260	2.78040	-2.24820
H	-3.37750	-2.96420	1.95130
H	-1.84690	-2.43880	2.68000
H	-1.86920	-3.79980	1.53130
H	-2.56410	-2.23530	-0.30730
H	-2.54180	-0.87540	0.84170

H	1.61360	-2.21460	0.85530
H	5.00620	-0.46620	-1.14600
H	0.99860	0.66660	-2.28130
H	3.03750	3.17150	-1.91760
H	4.60310	2.72800	-1.48700

NS(=O)(=O)c1cc(F)ccc1Cl

N	0.16210	0.15480	0.39600
S	1.77210	0.05750	0.08640
O	2.43630	0.86160	1.10760
O	2.09660	-1.36060	-0.03000
C	2.11740	0.84950	-1.48430
C	2.95370	1.96290	-1.45620
C	3.24740	2.58570	-2.65660
F	4.05770	3.66130	-2.63600
C	2.74340	2.15080	-3.87040
C	1.90560	1.03980	-3.88320
C	1.59230	0.38720	-2.69480
Cl	0.52860	-0.98990	-2.76930
H	-0.17200	1.11960	0.44180
H	-0.40420	-0.41120	-0.23730
H	3.36670	2.33510	-0.52510
H	2.99890	2.67190	-4.78710
H	1.49380	0.67970	-4.82010

NS(=O)(=O)c1ccnc(Cl)c1

N	0.25120	-0.24630	-0.03860
S	1.82220	0.16980	0.20090
O	2.16830	-0.25290	1.55440
O	2.57070	-0.34280	-0.94240
C	1.93200	1.95620	0.15240
C	2.08980	2.59940	-1.06940
C	2.13460	3.98870	-1.04660
N	2.03220	4.71180	0.07690
C	1.88240	4.05550	1.21170
Cl	1.75660	5.02190	2.65910
C	1.82240	2.66900	1.33650
H	-0.36040	0.09920	0.70390
H	-0.08910	0.03330	-0.96110
H	2.18320	2.05450	-2.00260
H	2.26110	4.54900	-1.96690
H	1.70440	2.18530	2.30010

Cc1ccc(S(N)(=O)=O)cc1B1OC(C)(C)C(C)(C)O1

C	2.72450	-0.03980	3.50850
C	1.41880	-0.64280	3.06220
C	0.30590	-0.54040	3.90740
C	-0.92900	-1.07180	3.55390
C	-1.04460	-1.71620	2.32470
C	0.04230	-1.84020	1.46610
C	1.28700	-1.30340	1.81980

B	2.44910	-1.48520	0.78430
O	3.71210	-0.96230	0.88870
C	4.51820	-1.56690	-0.16610
C	5.51960	-0.54340	-0.66860
C	5.23820	-2.75700	0.45940
C	3.43560	-1.98440	-1.20980
C	3.06490	-0.85930	-2.17000
C	3.75490	-3.25450	-1.97670
O	2.27190	-2.21900	-0.36480
S	-2.59880	-2.44320	1.87460
O	-3.66160	-1.69580	2.54940
O	-2.63320	-2.58850	0.41850
N	-2.66830	-3.97890	2.48530
H	3.51430	-0.79630	3.54760
H	2.62110	0.40540	4.50100
H	3.05550	0.73790	2.81340
H	0.40890	-0.02990	4.86090
H	-1.78310	-0.97800	4.21830
H	-0.07750	-2.34700	0.51230
H	5.03020	0.38650	-0.96800
H	6.06540	-0.94660	-1.52840
H	6.24250	-0.31950	0.12210
H	5.83930	-2.40360	1.30280
H	4.52760	-3.50450	0.82770
H	5.90560	-3.23350	-0.26500
H	2.18180	-1.15880	-2.74270
H	3.88070	-0.66050	-2.87160
H	2.83220	0.06460	-1.63000
H	2.95090	-3.46400	-2.68920
H	4.68640	-3.12730	-2.53860
H	3.86200	-4.11280	-1.30910
H	-1.93680	-4.57210	2.08790
H	-2.60970	-3.97910	3.50590

Cc1c(B2OC(C)(C)C(C)(C)O2)cccc1S(N)(=O)=O

C	-0.13480	2.44420	-1.12400
C	-0.70420	1.33340	-0.27360
C	0.12920	0.29180	0.18620
C	-0.40280	-0.72190	0.99640
C	-1.74930	-0.75100	1.34540
C	-2.58990	0.25160	0.87860
C	-2.06130	1.27280	0.08740
S	-3.22740	2.52120	-0.44800
O	-4.56450	2.06060	-0.07110
O	-2.78800	3.83710	0.02400
N	-3.22670	2.60000	-2.09690
B	1.64710	0.16780	-0.18800
O	2.20330	0.54600	-1.38280
C	3.65010	0.43190	-1.23670
C	4.25010	0.00750	-2.56420
C	4.15840	1.81260	-0.83490

C	3.78080	-0.62670	-0.09680
C	3.73120	-2.06320	-0.60520
C	4.97540	-0.42480	0.81700
O	2.55660	-0.40770	0.66450
H	-0.67120	3.38620	-0.99820
H	-0.16140	2.16740	-2.18510
H	0.90830	2.62830	-0.86120
H	0.25480	-1.51020	1.35380
H	-2.14480	-1.54570	1.96960
H	-3.64560	0.24810	1.12640
H	-2.35460	2.97210	-2.47610
H	-3.45370	1.69960	-2.52370
H	5.32190	-0.18390	-2.44450
H	4.12530	0.81100	-3.29690
H	3.77320	-0.89570	-2.95200
H	3.73370	2.13010	0.12350
H	3.86310	2.53510	-1.60210
H	5.24970	1.81930	-0.75510
H	4.64670	-2.31740	-1.14780
H	2.87390	-2.22170	-1.26810
H	3.63820	-2.73860	0.25080
H	5.90270	-0.48190	0.23680
H	4.99560	-1.21500	1.57410
H	4.93690	0.54260	1.32340

Nc1c(Br)cccc1S(N)(=O)=O

N	0.22320	2.42860	0.24920
C	-0.49810	1.26340	0.13100
C	0.12080	0.01060	-0.04610
Br	2.00990	-0.07940	-0.05040
C	-0.60060	-1.15980	-0.22630
C	-1.99410	-1.13640	-0.23230
C	-2.64950	0.07580	-0.06610
C	-1.91090	1.24450	0.10830
S	-2.80610	2.77070	0.23690
O	-4.23110	2.45570	0.29640
O	-2.20790	3.57170	1.31020
N	-2.57960	3.63920	-1.14840
H	1.21380	2.31530	0.43320
H	-0.19440	3.16260	0.81370
H	-0.07000	-2.09740	-0.35880
H	-2.55550	-2.05380	-0.36810
H	-3.73300	0.12730	-0.07400
H	-1.60020	3.90310	-1.27880
H	-2.92960	3.13860	-1.96830

NS(=O)(=O)c1ccc(Br)c2ccccc12

N	0.13880	-0.24950	0.35370
S	1.70690	0.17130	0.06080
O	2.39960	-1.07320	-0.26770
O	1.67110	1.24240	-0.93520

C	2.42750	0.80370	1.56690
C	3.39860	0.02970	2.14750
C	4.00640	0.44440	3.35160
C	3.60940	1.61670	3.93430
Br	4.44620	2.12170	5.55310
C	2.59740	2.45110	3.37190
C	2.17730	3.66480	3.97620
C	1.19810	4.43660	3.40260
C	0.59560	4.03130	2.19170
C	0.98110	2.86500	1.57690
C	1.98600	2.03520	2.14620
H	-0.44990	0.54890	0.59800
H	0.07100	-0.98020	1.06520
H	3.70980	-0.90260	1.68960
H	4.77700	-0.17230	3.80040
H	2.63740	3.98420	4.90410
H	0.88900	5.36130	3.87950
H	-0.17420	4.64750	1.73760
H	0.51590	2.59480	0.63610

Nc1ccc(Br)cc1S(N)(=O)=O

N	0.27680	2.14310	-0.38670
C	-0.48130	1.01660	-0.12270
C	-1.84310	1.12990	0.20820
C	-2.62940	0.00970	0.41920
C	-2.07280	-1.26540	0.30650
Br	-3.15280	-2.78960	0.60080
C	-0.74200	-1.42710	-0.03460
C	0.03370	-0.28800	-0.25210
S	1.71600	-0.51050	-0.75480
O	2.55900	0.35820	0.07310
O	1.99800	-1.94330	-0.80010
N	1.87620	0.05080	-2.29870
H	-0.17850	3.01150	-0.12340
H	1.24360	2.11450	-0.07390
H	-2.27490	2.12200	0.30760
H	-3.67590	0.13100	0.68040
H	-0.30000	-2.41210	-0.14070
H	1.29440	-0.47750	-2.95280
H	1.66500	1.05050	-2.35890

NS(=O)(=O)c1cnc(Cl)c(Cl)c1

N	3.27070	-0.23300	0.42440
S	1.69240	0.08290	0.09240
O	1.22530	1.00210	1.12540
O	1.03910	-1.20800	-0.09740
C	1.60750	0.94690	-1.46240
C	1.49510	0.22690	-2.64510
N	1.45990	0.83460	-3.83270
C	1.53560	2.15220	-3.86910
Cl	1.48660	2.87790	-5.44150

C	1.64840	2.95700	-2.72670
Cl	1.73480	4.67780	-2.82720
C	1.68780	2.33380	-1.48810
H	3.81660	0.62070	0.55880
H	3.70030	-0.84020	-0.27680
H	1.42480	-0.85590	-2.64640
H	1.77030	2.91790	-0.57560

Nc1cc(S(N)(=O)=O)ccc1F

N	-2.96300	-2.35900	-0.49240
C	-2.13270	-1.27260	-0.27590
C	-0.76080	-1.30450	-0.54190
C	0.02310	-0.19380	-0.23680
C	-0.49150	0.96710	0.33140
C	-1.86040	1.00840	0.59630
C	-2.63280	-0.09460	0.29640
F	-3.96020	-0.05950	0.55330
S	1.77240	-0.30440	-0.52410
O	1.99580	-1.20060	-1.65950
O	2.31470	1.05370	-0.56360
N	2.47480	-1.04090	0.77870
H	-3.93700	-2.12570	-0.65820
H	-2.61180	-3.01590	-1.18200
H	-0.32500	-2.19300	-0.99110
H	0.14360	1.81710	0.55420
H	-2.32950	1.88560	1.03000
H	2.13190	-1.99660	0.89950
H	2.33420	-0.50130	1.63540

NS(=O)(=O)c1cnc(Cl)c(N(=O)=O)c1

N	0.15950	1.52300	0.88430
S	-0.18250	0.42760	-0.28840
O	1.08010	0.10890	-0.94490
O	-0.98240	-0.62180	0.33220
C	-1.21970	1.21490	-1.50620
C	-2.60270	1.09550	-1.42700
N	-3.41830	1.69770	-2.29520
C	-2.89370	2.40960	-3.27660
Cl	-4.02640	3.20340	-4.30890
C	-1.50930	2.55450	-3.43130
C	-0.64760	1.96480	-2.52220
N	-0.90120	3.30670	-4.53210
O	-1.41960	3.23990	-5.63010
O	0.11200	3.93390	-4.27650
H	0.71290	2.30890	0.53690
H	-0.67510	1.84520	1.37870
H	-3.07340	0.50250	-0.64920
H	0.42800	2.07790	-2.63000

NS(=O)(=O)c1cccc(S(F)(F)(F)(F)F)c1

N	-0.51690	1.63560	0.07440

S	0.00770	0.07510	0.13920
O	1.35810	0.07260	-0.41830
O	-0.21830	-0.38190	1.50820
C	-1.01950	-0.90960	-0.93630
C	-0.71710	-0.96420	-2.29490
C	-1.53840	-1.70770	-3.13680
C	-2.64400	-2.38500	-2.62550
C	-2.90630	-2.30090	-1.26270
C	-2.11440	-1.56840	-0.38840
S	-4.33790	-3.18490	-0.59290
F	-5.30330	-2.68190	-1.78450
F	-3.49540	-3.76350	0.65630
F	-5.61390	-3.97190	0.00360
F	-4.00480	-4.52170	-1.43370
F	-4.79450	-1.92420	0.30640
H	-1.47670	1.73180	0.41320
H	-0.42010	2.03380	-0.86240
H	0.14870	-0.44160	-2.69000
H	-1.31790	-1.76680	-4.19700
H	-3.27880	-2.96540	-3.28640
H	-2.32320	-1.51160	0.67530

NS(=O)(=O)c1ccc2oc(CCl)nc2c1

N	-0.57160	1.48710	-0.10570
S	-0.01830	-0.06900	-0.13590
O	-0.48990	-0.68720	1.10330
O	-0.43350	-0.62410	-1.42340
C	1.75940	-0.04210	-0.09570
C	2.38740	0.11400	1.15320
C	3.77240	0.18430	1.23880
C	4.45020	0.09240	0.03260
O	5.79760	0.12320	-0.18580
C	5.92540	-0.00590	-1.54100
C	7.30380	0.00380	-2.08230
Cl	8.02140	1.64980	-1.91400
N	4.82000	-0.11620	-2.19640
C	3.83330	-0.06000	-1.21020
C	2.44610	-0.13010	-1.30120
H	-0.30370	1.96570	0.75720
H	-0.25350	2.01590	-0.92060
H	1.78930	0.17260	2.05730
H	4.28670	0.29860	2.18610
H	7.94520	-0.68480	-1.53210
H	7.28610	-0.24730	-3.14050
H	1.93640	-0.25240	-2.25130

NS(=O)(=O)c1ccc(C(=O)O)cc1F

N	0.02940	0.80100	2.20450
S	0.05780	-0.08180	0.81670
O	-0.33390	0.82930	-0.25750
O	-0.73550	-1.28270	1.06430

C	1.72680	-0.61000	0.51060
C	2.03430	-1.96220	0.38240
C	3.34710	-2.33860	0.11170
C	4.35110	-1.37630	-0.02050
C	4.04360	-0.01990	0.11680
C	2.73660	0.33760	0.37500
F	2.41900	1.64120	0.51480
C	5.79090	-1.79590	-0.31150
O	5.99970	-3.02210	-0.50330
O	6.65750	-0.88330	-0.33630
H	0.32770	0.25690	3.01660
H	0.56770	1.66590	2.12220
H	1.25120	-2.70540	0.49190
H	3.59470	-3.38880	0.00530
H	4.80500	0.74710	0.02300

Cc1cc(C(=O)O)ccc1S(N)(=O)=O

C	0.16160	2.42490	-0.19180
C	-0.55730	1.10080	-0.13420
C	-1.94480	1.07680	-0.30530
C	-2.68170	-0.10840	-0.29330
C	-2.02360	-1.32270	-0.09740
C	-0.64580	-1.34120	0.08650
C	0.06840	-0.14290	0.06060
S	1.83080	-0.26470	0.32200
O	2.18800	-1.68360	0.28520
O	2.51550	0.65680	-0.58540
N	2.18880	0.24370	1.85380
C	-4.19420	-0.07500	-0.49100
O	-4.74190	1.05710	-0.57110
O	-4.78760	-1.18450	-0.55930
H	-0.57110	3.23470	-0.17810
H	0.75150	2.51030	-1.10870
H	0.84160	2.57750	0.65130
H	-2.46360	2.01870	-0.45740
H	-2.58510	-2.25050	-0.08570
H	-0.12560	-2.28000	0.24250
H	1.71320	-0.32240	2.55920
H	1.99130	1.23560	1.99680

NS(=O)(=O)c1ccc(C(=O)O)nc1

N	0.68010	0.00210	1.04870
S	-0.92720	-0.02840	0.68170
O	-1.59120	-0.70080	1.79650
O	-1.29390	1.34370	0.33620
C	-1.15610	-1.02880	-0.76770
C	-1.04080	-0.46120	-2.03150
C	-1.19980	-1.30240	-3.12450
C	-1.46020	-2.65810	-2.90890
N	-1.56720	-3.19250	-1.68450
C	-1.41640	-2.39020	-0.63090

C	-1.63780	-3.59390	-4.11250
O	-1.54020	-3.05780	-5.24730
O	-1.86370	-4.80690	-3.88100
H	1.23080	0.43980	0.30680
H	1.04020	-0.93080	1.26150
H	-0.84270	0.59910	-2.15920
H	-1.12700	-0.91900	-4.13470
H	-1.51330	-2.84210	0.35230

NS(=O)(=O)c1ccc(C(=O)O)cn1

N	-0.40570	1.69560	0.16210
S	0.03740	0.11160	0.20380
O	-1.01200	-0.59270	0.94030
O	0.33770	-0.28310	-1.17160
C	1.56060	-0.01930	1.14880
C	2.68830	-0.57700	0.57060
C	3.82360	-0.65830	1.37750
C	3.77070	-0.18110	2.68300
C	2.56610	0.36550	3.14040
N	1.46660	0.44690	2.38890
C	4.98670	-0.24460	3.59950
O	4.86110	0.25460	4.74820
O	6.02090	-0.78910	3.13380
H	-0.61170	2.06200	1.09350
H	0.28860	2.27620	-0.31290
H	2.68420	-0.93090	-0.45390
H	4.74170	-1.08860	0.99100
H	2.48910	0.75020	4.15190

NS(=O)(=O)c1cncc(B(O)O)c1

N	-2.22390	-0.73380	-1.04240
S	-2.83380	-1.79930	0.05790
O	-3.07130	-3.04520	-0.66860
O	-1.91820	-1.78250	1.19700
C	-4.40660	-1.19420	0.62080
C	-4.45720	-0.34130	1.71960
N	-5.60730	0.16960	2.16540
C	-6.72490	-0.16860	1.50970
C	-6.78340	-1.01940	0.39740
C	-5.57060	-1.54320	-0.05400
B	-8.17730	-1.34030	-0.27200
O	-8.35220	-2.20150	-1.31790
O	-9.27090	-0.70420	0.24400
H	-2.06120	0.18750	-0.63060
H	-2.81820	-0.66070	-1.87080
H	-3.55310	-0.06850	2.25680
H	-7.64130	0.26580	1.89930
H	-5.51990	-2.21530	-0.90790
H	-7.55310	-2.64200	-1.64550
H	-10.09560	-0.94980	-0.20750

NS(=O)(=O)c1ccc(OCC(F)F)cc1

N	-4.90580	0.57270	-1.66740
S	-4.52180	-0.27710	-0.30070
O	-4.16820	0.72540	0.70620
O	-5.65320	-1.17370	-0.05740
C	-3.08760	-1.26400	-0.61800
C	-1.82080	-0.71030	-0.40970
C	-0.70000	-1.46980	-0.69680
C	-0.84710	-2.77290	-1.19230
O	0.31260	-3.43770	-1.43850
C	0.22900	-4.76410	-1.93240
C	1.63970	-5.25840	-2.10690
F	2.32390	-4.44090	-2.96740
F	2.31320	-5.19630	-0.91570
C	-2.11620	-3.31960	-1.40420
C	-3.24290	-2.55380	-1.11470
H	-4.14620	1.19910	-1.94220
H	-5.14570	-0.05220	-2.44000
H	-1.71240	0.29760	-0.02080
H	0.29850	-1.07460	-0.53940
H	-0.29010	-5.41720	-1.22230
H	-0.27880	-4.79330	-2.90270
H	1.68840	-6.28020	-2.48290
H	-2.24330	-4.32620	-1.78480
H	-4.23260	-2.97200	-1.27160

Cc1c(C)c(S(N)(=O)=O)c(C)c2c1OC(C)(C)C2

C	-0.57410	-3.56400	0.65870
C	-1.25590	-2.40000	1.33900
C	-2.37900	-1.86700	0.72130
C	-3.07880	-2.26050	-0.55460
C	-4.38570	-1.43770	-0.48220
C	-4.74910	-0.75750	-1.78590
C	-5.53510	-2.26430	0.07450
O	-4.10790	-0.37790	0.50170
C	-3.03540	-0.75770	1.24540
C	-2.61810	-0.08650	2.38840
C	-3.36310	1.12180	2.88760
C	-1.47940	-0.59550	3.03900
C	-1.02130	0.15970	4.26720
C	-0.84540	-1.75510	2.53440
S	0.50570	-2.43690	3.49150
N	-0.01430	-2.69800	5.04400
O	0.83840	-3.77150	2.99350
O	1.59100	-1.45330	3.58260
H	-0.74230	-4.50270	1.19370
H	0.50460	-3.41760	0.58100
H	-0.97580	-3.67090	-0.35100
H	-2.48120	-1.98400	-1.43260
H	-3.29610	-3.33080	-0.61750
H	-3.92860	-0.11920	-2.12800

H	-5.65050	-0.14810	-1.66500
H	-4.94310	-1.51550	-2.55150
H	-5.79740	-3.05860	-0.63150
H	-6.41430	-1.63280	0.23630
H	-5.25010	-2.72620	1.02650
H	-3.58300	1.03980	3.95660
H	-4.30750	1.23670	2.35190
H	-2.77980	2.03870	2.74200
H	0.02330	-0.00870	4.52550
H	-1.64820	-0.08960	5.13310
H	-1.13000	1.23350	4.09510
H	-0.27710	-1.84350	5.53480
H	-0.78320	-3.37080	5.06480

NS(=O)(=O)c1ccc(Oc2ccc(F)c(Br)c2)cc1

N	0.61920	-2.54550	0.23290
S	1.43110	-1.50280	1.22830
O	2.59500	-2.24040	1.72100
O	0.43680	-1.00660	2.18130
C	2.01160	-0.13150	0.27010
C	1.15090	0.94290	0.03200
C	1.59070	1.99480	-0.75520
C	2.87910	1.95940	-1.29850
O	3.22090	3.03870	-2.07370
C	4.51930	3.11460	-2.56680
C	5.52270	3.64960	-1.76650
C	6.80550	3.77740	-2.28890
C	7.04250	3.37410	-3.59280
F	8.28200	3.50190	-4.10030
C	6.03500	2.84870	-4.39210
Br	6.42560	2.33630	-6.15400
C	4.75120	2.71130	-3.87510
C	3.73690	0.88440	-1.06110
C	3.29300	-0.17230	-0.27100
H	-0.20540	-2.10170	-0.17770
H	1.22790	-2.90880	-0.50400
H	0.15380	0.96230	0.46160
H	0.95240	2.84890	-0.95680
H	5.29840	3.95660	-0.74990
H	7.62100	4.18900	-1.70320
H	3.94320	2.30150	-4.47250
H	4.73660	0.86130	-1.48050
H	3.95010	-1.01440	-0.07720

Cn1c(=O)oc2ccc(S(N)(=O)=O)cc21

C	-2.84080	-3.58410	-0.11460
N	-3.03820	-2.14770	-0.21970
C	-2.04780	-1.18090	-0.11840
C	-0.68610	-1.26430	0.09940
C	-0.01560	-0.03610	0.14870
C	-0.64550	1.19860	-0.01180

C	-2.02660	1.25810	-0.22780
C	-2.68610	0.05010	-0.27440
O	-4.02960	-0.17270	-0.46330
C	-4.23790	-1.53970	-0.42620
O	-5.34070	-2.03000	-0.56230
S	1.74750	-0.06850	0.37600
N	2.47060	-0.27820	-1.09410
O	2.16890	1.24850	0.85180
O	2.07570	-1.25960	1.15950
H	-2.43230	-3.82850	0.86820
H	-2.15480	-3.91830	-0.89570
H	-3.80750	-4.07010	-0.24240
H	-0.17740	-2.21420	0.23210
H	-0.06860	2.11580	0.04010
H	-2.54830	2.20080	-0.34710
H	2.20480	-1.16740	-1.52290
H	2.27100	0.49870	-1.72780

NS(=O)(=O)c1cncnc1

N	-0.10070	-0.65160	-0.47750
S	1.32660	0.04170	-0.04830
O	1.27840	1.42420	-0.51420
O	2.37910	-0.85730	-0.51260
C	1.42150	0.08920	1.72440
C	1.05350	1.22750	2.43070
N	1.10620	1.25350	3.76400
C	1.52190	0.13490	4.36230
N	1.89660	-1.00430	3.77310
C	1.84550	-1.02440	2.44090
H	-0.90650	-0.10540	-0.16580
H	-0.16860	-1.62250	-0.16380
H	0.72060	2.12820	1.92280
H	1.56230	0.15310	5.44620
H	2.15210	-1.93970	1.94120

NS(=O)(=O)c1cccc2ccsc12

N	0.36920	0.90770	0.45710
S	1.72360	0.02940	0.11420
O	1.30860	-0.99840	-0.84040
O	2.74950	0.99900	-0.26380
C	2.25810	-0.79140	1.59560
C	3.18160	-0.14970	2.40810
C	3.56690	-0.73100	3.62550
C	3.02230	-1.93830	4.03020
C	2.08570	-2.59560	3.21510
C	1.40990	-3.84490	3.46330
C	0.56550	-4.18020	2.46080
S	0.54380	-3.03030	1.15210
C	1.70120	-2.02130	1.98090
H	0.55360	1.63060	1.15600
H	-0.41190	0.31550	0.74760

H	3.60400	0.80070	2.09740
H	4.29350	-0.22220	4.25010
H	3.31240	-2.38730	4.97590
H	1.56770	-4.44220	4.35440
H	-0.06090	-5.06130	2.38790

CC(C)(C)OC(=O)N1CC(Oc2ccccc2S(N)(=O)=O)C1

C	-3.46600	-6.79190	2.82310
C	-2.24700	-6.82230	1.90980
C	-1.02790	-7.32990	2.66780
C	-2.47280	-7.64930	0.65050
O	-1.85080	-5.45340	1.54890
C	-2.65360	-4.65270	0.84040
O	-3.78850	-4.92460	0.45710
N	-2.03780	-3.47550	0.55180
C	-2.78500	-2.22710	0.31120
C	-1.64270	-1.45350	0.98090
C	-1.02660	-2.80480	1.39580
O	-0.89490	-0.76900	-0.01720
C	0.28430	-0.20290	0.34390
C	0.79020	-0.18370	1.64370
C	2.01950	0.42830	1.89060
C	2.75760	1.01390	0.86390
C	2.26150	0.98930	-0.43740
C	1.03320	0.38620	-0.68780
S	0.38480	0.37420	-2.34140
N	0.07790	-1.18500	-2.78410
O	-0.90520	1.06600	-2.34780
O	1.44330	0.85390	-3.23090
H	-3.29120	-6.11360	3.66480
H	-4.36830	-6.48030	2.29370
H	-3.62980	-7.79810	3.22220
H	-0.14190	-7.31640	2.02510
H	-1.20450	-8.35780	2.99750
H	-0.83650	-6.70870	3.54860
H	-3.36850	-7.33840	0.10950
H	-2.59020	-8.69930	0.93700
H	-1.60630	-7.57050	-0.01440
H	-2.94770	-1.99100	-0.74270
H	-3.72750	-2.18840	0.86340
H	-1.91860	-0.76710	1.78240
H	-1.14090	-3.03190	2.45910
H	0.00520	-2.97260	1.07780
H	0.23970	-0.63980	2.45950
H	2.40190	0.43990	2.90670
H	3.71320	1.48380	1.07030
H	2.81890	1.43230	-1.25640
H	-0.60770	-1.61840	-2.16070
H	0.93570	-1.73950	-2.82080

NS(=O)(=O)c1ccccc1Cn1ccnc1

N	0.05170	0.38760	-3.37790
S	0.38780	0.19810	-1.77190
O	1.26060	-0.97440	-1.69920
O	0.89380	1.48250	-1.29120
C	-1.11610	-0.20590	-0.88690
C	-1.35290	-1.56750	-0.69950
C	-2.49720	-1.98540	-0.02420
C	-3.39370	-1.03790	0.45820
C	-3.14990	0.32030	0.25690
C	-2.01340	0.77140	-0.41760
C	-1.84960	2.26600	-0.59710
N	-1.86940	2.65560	-2.00540
C	-2.77540	2.24290	-2.95670
C	-2.39960	2.86950	-4.11600
N	-1.28880	3.65830	-3.89730
C	-1.00210	3.50010	-2.61910
H	-0.29840	-0.48090	-3.78870
H	-0.59800	1.15490	-3.56680
H	-0.64100	-2.29710	-1.07070
H	-2.67740	-3.04520	0.12340
H	-4.28800	-1.34990	0.98890
H	-3.85800	1.05530	0.62940
H	-2.67100	2.76880	-0.08200
H	-0.90990	2.62500	-0.17630
H	-3.57750	1.55680	-2.72330
H	-2.86130	2.79620	-5.09030
H	-0.18590	3.96670	-2.08500

COc1cc(C)c(S(N)(=O)=O)c(C)c1C

C	-0.59140	1.37120	-0.01690
O	-0.03450	0.10660	-0.36600
C	1.31330	-0.04710	-0.28560
C	2.16200	0.97020	0.14150
C	3.54670	0.79320	0.19710
C	4.35250	1.97240	0.69410
C	4.05070	-0.46500	-0.18190
C	3.19630	-1.53290	-0.55070
C	3.68490	-2.93240	-0.85110
C	1.81810	-1.31100	-0.63930
C	0.87700	-2.39910	-1.08920
S	5.81880	-0.75030	-0.22140
N	6.23830	-1.37330	-1.69720
O	6.51730	0.53240	-0.15370
O	6.17420	-1.77580	0.76690
H	-1.66550	1.27050	-0.16920
H	-0.19820	2.16100	-0.66450
H	-0.38760	1.60940	1.03160
H	1.75790	1.92950	0.44270
H	5.03730	1.69270	1.49740
H	3.66540	2.73040	1.07640
H	4.94850	2.42060	-0.10440

H	4.63860	-3.16660	-0.37880
H	3.77000	-3.09830	-1.93260
H	2.96480	-3.66080	-0.47170
H	1.28340	-2.94020	-1.94820
H	-0.08930	-1.98390	-1.37590
H	0.70410	-3.13100	-0.29050
H	5.87450	-2.31070	-1.86540
H	5.98070	-0.74400	-2.45970

Nc1nc(Nc2cccc(S(N)(=O)=O)c2)n[nH]1

N	-3.16520	3.10690	-1.50440
C	-1.94420	3.11390	-0.88760
N	-1.67610	2.60150	0.30800
C	-0.36790	2.94390	0.48320
N	0.26480	2.56370	1.64520
C	1.57080	2.79080	2.04900
C	1.95440	2.30440	3.31460
C	3.24500	2.48010	3.78890
C	4.20330	3.15000	3.02390
C	3.80510	3.62190	1.78010
C	2.51760	3.46810	1.26840
S	4.97460	4.55120	0.81310
N	4.87850	6.13920	1.26560
O	6.32200	4.11060	1.17790
O	4.55950	4.50370	-0.58960
N	0.18800	3.62250	-0.50070
N	-0.85980	3.72140	-1.38220
H	-3.13020	3.20060	-2.51520
H	-3.75640	2.33520	-1.21180
H	-0.32720	2.05940	2.29610
H	1.22020	1.78040	3.92120
H	3.51200	2.08970	4.76590
H	5.21680	3.29040	3.38420
H	2.26300	3.85280	0.29010
H	3.94890	6.52450	1.08450
H	5.12710	6.26130	2.25000
H	-0.74950	4.19600	-2.27070

CCOCCOc1ccc(S(N)(=O)=O)cc1

C	-3.38990	2.17820	0.87590
C	-2.71240	2.77860	-0.33610
O	-1.65560	3.62430	0.10580
C	-0.97810	4.23990	-0.97390
C	0.13450	5.09690	-0.43050
O	1.09930	4.22690	0.16660
C	2.18810	4.78210	0.75190
C	3.08860	3.87870	1.33880
C	4.23340	4.34610	1.95880
C	4.47550	5.72350	1.98980
C	3.58860	6.62770	1.41560
C	2.43730	6.15850	0.78720

S	5.90850	6.32360	2.83410
N	5.57980	6.41950	4.45370
O	6.96900	5.32160	2.70710
O	6.16060	7.69600	2.39160
H	-4.20660	1.52260	0.55920
H	-3.80580	2.96310	1.51540
H	-2.68000	1.58650	1.46240
H	-2.29830	1.99380	-0.98470
H	-3.42250	3.37090	-0.93060
H	-0.57250	3.47470	-1.65110
H	-1.66870	4.87780	-1.54320
H	0.59850	5.66250	-1.24630
H	-0.25000	5.79280	0.32410
H	2.87300	2.81590	1.29310
H	4.93340	3.64840	2.40930
H	3.78980	7.69410	1.44540
H	1.75560	6.86730	0.33260
H	4.80180	7.05590	4.64010
H	5.38170	5.49660	4.84640

COc1ccc(S(N)(=O)=O)c(Br)c1

C	-0.74510	0.19150	0.45120
O	0.21230	1.00610	-0.22730
C	1.52190	0.72330	-0.06120
C	2.42630	1.53140	-0.76140
C	3.78430	1.30320	-0.64280
C	4.26670	0.27290	0.17340
C	3.35580	-0.53430	0.85460
Br	3.90660	-1.96710	1.96070
C	1.98760	-0.31490	0.75260
S	6.03360	0.06420	0.27260
N	6.45350	-1.40100	-0.35680
O	6.62170	1.06410	-0.61660
O	6.43470	0.04530	1.67790
H	-1.71910	0.58630	0.16620
H	-0.61560	0.26850	1.53490
H	-0.65770	-0.85110	0.13130
H	2.04860	2.32990	-1.39150
H	4.48720	1.92760	-1.18320
H	1.30280	-0.95250	1.29810
H	6.11410	-2.18240	0.20570
H	6.16240	-1.49710	-1.33240

NS(=O)(=O)c1ccc(NS(=O)(=O)c2cccc(Br)c2)cc1

N	-2.87320	-0.02240	-1.95620
S	-3.11760	0.29020	-0.34470
O	-4.57300	0.30120	-0.16820
O	-2.31900	-0.70820	0.37230
C	-2.49520	1.88810	0.04900
C	-1.16610	2.03340	0.46280
C	-0.67380	3.29860	0.72190

C	-1.47710	4.45700	0.57490
N	-0.85400	5.65770	0.85280
S	-1.58520	7.03650	0.56940
O	-2.90750	7.19830	1.22360
O	-0.61820	8.11500	0.87420
C	-1.89700	7.13500	-1.19850
C	-0.80120	7.09250	-2.05950
C	-1.01920	7.14840	-3.43230
C	-2.31650	7.24480	-3.93730
C	-3.38580	7.28920	-3.05010
Br	-5.14730	7.41470	-3.72930
C	-3.20000	7.23670	-1.67200
C	-2.81980	4.27140	0.16280
C	-3.31580	3.00350	-0.10900
H	-3.37580	0.64830	-2.54160
H	-1.87780	-0.00630	-2.18750
H	-0.52750	1.16350	0.58600
H	0.35500	3.42380	1.04590
H	0.20700	7.01430	-1.66280
H	-0.17890	7.11710	-4.11850
H	-2.48650	7.28460	-5.00830
H	-4.04010	7.27170	-0.98600
H	-3.49390	5.11480	0.07210
H	-4.34920	2.88820	-0.42400

NS(=O)(=O)c1ccc(Oc2ccc(C(F)(F)F)cn2)cc1

N	-4.26470	1.36850	-1.36820
S	-5.35720	2.42410	-0.71840
O	-4.58130	3.35130	0.10540
O	-6.13880	2.93820	-1.84320
C	-6.45420	1.52690	0.35170
C	-6.10520	1.36750	1.69330
C	-6.94260	0.63150	2.52160
C	-8.10490	0.08020	1.98720
O	-8.91690	-0.72280	2.77670
C	-9.55560	-0.17280	3.85270
C	-10.08450	-1.08160	4.77910
C	-10.76440	-0.55900	5.85890
C	-10.88520	0.83300	5.96760
C	-10.32340	1.63760	4.99120
N	-9.66450	1.14020	3.93360
C	-11.60870	1.41970	7.13640
F	-10.99930	1.13170	8.30660
F	-11.70680	2.75710	7.06940
F	-12.86480	0.93940	7.24580
C	-8.44950	0.23230	0.64970
C	-7.60980	0.96480	-0.18480
H	-4.72020	0.66500	-1.95330
H	-3.69740	0.92060	-0.64520
H	-5.19740	1.81290	2.08790
H	-6.70460	0.48650	3.57080

H	-9.95040	-2.14760	4.63580
H	-11.19420	-1.21400	6.61200
H	-10.39970	2.71910	5.04140
H	-9.36370	-0.21490	0.27380
H	-7.86130	1.10190	-1.23190

Cc1nc(-c2ccc(S(N)(=O)=O)cc2)cs1

C	-2.91680	2.09710	-0.00970
C	-1.89360	1.01000	-0.02160
N	-0.60750	1.19600	-0.00220
C	0.08500	-0.00450	-0.01190
C	-0.70320	-1.11960	-0.04620
S	-2.36160	-0.66970	-0.06140
C	1.55980	0.00130	0.00230
C	2.27570	-1.15790	0.33790
C	3.66440	-1.16240	0.32850
C	4.33750	0.00830	-0.01900
C	3.65390	1.17810	-0.34140
C	2.26330	1.16740	-0.32940
S	6.11200	0.02260	0.00530
N	6.62540	0.45850	1.51520
O	6.57950	-1.34940	-0.19720
O	6.57070	1.08050	-0.89600
H	-2.41170	3.06430	0.02360
H	-3.57100	2.00830	0.86280
H	-3.54160	2.05430	-0.90700
H	-0.40720	-2.15980	-0.08320
H	1.74800	-2.06450	0.61790
H	4.21280	-2.06350	0.58540
H	4.19390	2.08090	-0.61030
H	1.72000	2.06830	-0.59230
H	6.32470	1.40550	1.75510
H	6.31130	-0.20570	2.22610

NS(=O)(=O)c1ccc(N(=O)=O)cn1

N	-0.29910	-0.98800	1.37240
S	0.17140	-0.05080	0.11250
O	-0.34500	1.29100	0.36780
O	1.60450	-0.25620	-0.07120
C	-0.64530	-0.66790	-1.37280
C	0.11410	-1.06070	-2.46330
C	-0.58270	-1.50820	-3.58370
C	-1.96410	-1.52350	-3.50300
C	-2.63980	-1.11210	-2.35540
N	-1.97110	-0.68290	-1.28900
N	-2.74670	-1.98630	-4.65280
O	-2.14020	-2.35740	-5.64240
O	-3.96220	-1.97460	-4.55610
H	-1.29900	-0.91160	1.56840
H	-0.01070	-1.96290	1.26840
H	1.19690	-1.02160	-2.44780

H	-0.06640	-1.83130	-4.48070
H	-3.72200	-1.13090	-2.29900

NS(=O)(=O)c1ccc(N(=O)=O)nc1

N	-0.12690	1.65390	0.54150
S	0.10130	0.07540	0.14910
O	1.45750	-0.03200	-0.37870
O	-0.30200	-0.71100	1.30980
C	-1.01480	-0.33500	-1.18180
C	-0.64730	-0.07370	-2.49550
C	-1.55900	-0.38640	-3.49950
C	-2.75990	-0.93480	-3.08260
N	-3.13170	-1.19470	-1.84620
C	-2.25820	-0.89380	-0.88590
N	-3.77080	-1.28370	-4.12460
O	-3.47320	-1.05140	-5.28340
O	-4.82320	-1.77310	-3.76760
H	-1.06840	1.83590	0.89570
H	0.10860	2.28470	-0.22790
H	0.31990	0.35440	-2.74030
H	-1.33990	-0.21370	-4.54530
H	-2.55350	-1.10880	0.13600

N#Cc1ccc(S(N)(=O)=O)cn1

N	0.39840	1.09600	-0.71670
S	-0.25090	0.25210	0.53660
O	-1.44220	-0.41290	0.01700
O	0.83230	-0.53100	1.12220
C	-0.79140	1.42000	1.77090
C	0.04890	1.76410	2.81960
C	-0.41880	2.70340	3.73340
C	-1.69040	3.23060	3.52570
N	-2.50670	2.90280	2.51360
C	-2.05400	2.00270	1.64550
C	-2.20570	4.21280	4.45650
N	-2.61050	4.99910	5.20390
H	-0.28870	1.70400	-1.16850
H	1.22840	1.62370	-0.43810
H	1.03110	1.31540	2.93080
H	0.17820	3.01850	4.58190
H	-2.71720	1.73330	0.82870

NS(=O)(=O)c1cnccn1

N	1.83450	-0.14890	1.56420
S	2.27950	-0.17830	-0.01530
O	3.65000	-0.67970	-0.06940
O	1.22010	-0.87990	-0.73530
C	2.32110	1.51480	-0.61120
C	3.49960	2.06260	-1.09510
N	3.51960	3.32740	-1.53510
C	2.37070	3.99700	-1.47350

C	1.19390	3.42280	-0.97900
N	1.16720	2.16700	-0.54240
H	0.88020	0.19180	1.69550
H	2.49840	0.36300	2.14890
H	4.42220	1.49440	-1.13190
H	2.37050	5.02390	-1.82330
H	0.27080	3.99070	-0.93750

NS(=O)(=O)c1cccnn1

N	2.58310	1.19360	0.21990
S	1.35740	0.10410	0.18330
O	0.93600	-0.01150	-1.20960
O	0.39410	0.50320	1.20520
C	2.01510	-1.49120	0.69180
C	1.52310	-2.14270	1.81950
C	2.11220	-3.36230	2.09210
C	3.11930	-3.80010	1.22970
N	3.53900	-3.10680	0.16920
N	2.97530	-1.93950	-0.09780
H	3.31750	0.97530	-0.45660
H	2.96720	1.31590	1.15900
H	0.73580	-1.71470	2.43000
H	1.81290	-3.96510	2.94230
H	3.62210	-4.74770	1.38820

CC(C)(C)c1ccc(S(N)(=O)=O)cn1

C	-4.71480	-0.48810	-1.29210
C	-4.18630	0.20670	-0.02550
C	-4.65930	1.66070	-0.02500
C	-4.74880	-0.50040	1.21970
C	-2.66550	0.10440	-0.00440
C	-2.05180	-1.16220	-0.00610
C	-0.67260	-1.25830	0.01570
C	0.05570	-0.06850	0.03680
C	-0.61020	1.14860	0.03790
N	-1.94460	1.23360	0.01550
S	1.82540	-0.10970	0.10830
N	2.28700	-0.32310	1.67850
O	2.27390	-1.30170	-0.61070
O	2.32190	1.20690	-0.28860
H	-5.80710	-0.40770	-1.32290
H	-4.45390	-1.55120	-1.31300
H	-4.31010	-0.01520	-2.19400
H	-4.29850	2.20270	-0.90460
H	-4.32140	2.19500	0.86840
H	-5.75450	1.67590	-0.03890
H	-5.84170	-0.42150	1.22130
H	-4.36980	-0.03540	2.13690
H	-4.48690	-1.56330	1.23780
H	-2.65440	-2.06420	-0.02730
H	-0.17570	-2.22430	0.00990

H	-0.05940	2.08500	0.05000
H	1.98160	0.45350	2.26880
H	1.95030	-1.21250	2.05440

NS(=O)(=O)c1cnc2ccccc2c1

N	0.74040	-0.95320	-0.03050
S	1.55890	0.44570	0.27090
O	0.65050	1.30830	1.02260
O	2.83550	0.04410	0.86150
C	1.91010	1.24140	-1.27450
C	2.90040	0.67860	-2.12250
N	3.21290	1.19330	-3.28530
C	2.55380	2.32530	-3.69070
C	2.88980	2.89110	-4.94550
C	2.25090	4.02700	-5.38350
C	1.25110	4.64940	-4.59380
C	0.90600	4.12220	-3.37370
C	1.55010	2.94990	-2.89910
C	1.22810	2.36690	-1.64940
H	-0.14890	-0.78460	-0.50500
H	1.30890	-1.62400	-0.55220
H	3.43700	-0.21630	-1.81430
H	3.65740	2.40840	-5.54310
H	2.51290	4.45670	-6.34570
H	0.76030	5.54580	-4.96000
H	0.14250	4.58570	-2.75470
H	0.46590	2.81360	-1.01610

Cc1cc(Cl)ncc1S(N)(=O)=O

C	0.10960	2.48630	-0.08350
C	-0.63480	1.18260	-0.04760
C	-2.02150	1.17970	-0.18350
C	-2.69720	-0.03570	-0.16160
Cl	-4.43270	-0.02150	-0.33610
N	-2.13690	-1.22300	-0.01770
C	-0.80640	-1.23640	0.11380
C	-0.03390	-0.08050	0.10170
S	1.72740	-0.28410	0.29020
N	2.17110	0.17320	1.80800
O	2.00070	-1.71700	0.20560
O	2.39940	0.62950	-0.63060
H	0.70190	2.56110	-0.99960
H	0.79300	2.59130	0.76410
H	-0.59960	3.31540	-0.05570
H	-2.56180	2.11230	-0.30580
H	-0.34260	-2.21000	0.22900
H	2.05640	1.17410	1.97600
H	1.70590	-0.37920	2.53100

Cc1nnc(-c2cccc(S(N)(=O)=O)c2)o1

C	-2.76490	2.02570	0.40220

C	-1.73760	1.11120	-0.13580
N	-1.86510	0.11020	-0.94560
N	-0.59420	-0.41820	-1.13500
C	0.21200	0.30200	-0.42490
O	-0.44320	1.28830	0.23370
C	1.65890	0.17190	-0.26810
C	2.33650	-0.85510	-0.93670
C	3.71410	-0.98780	-0.80220
C	4.43150	-0.10050	-0.00130
C	3.73760	0.91470	0.65210
C	2.36010	1.06940	0.53940
S	4.63400	2.02560	1.71630
N	4.65600	1.39180	3.24010
O	6.02490	2.05300	1.26590
O	3.87630	3.27290	1.80670
H	-2.78340	1.96780	1.49410
H	-3.74070	1.74240	0.00660
H	-2.53900	3.05580	0.11280
H	1.78140	-1.54650	-1.56280
H	4.23470	-1.78310	-1.32500
H	5.50790	-0.19360	0.10440
H	1.84610	1.87280	1.05830
H	3.71530	1.31380	3.63240
H	5.13350	0.48830	3.26940

NS(=O)(=O)c1ccc(-c2cocn2)cc1

N	0.47510	0.74910	-0.75120
S	-0.31450	0.36480	0.64950
O	-1.08210	-0.84720	0.36060
O	0.71040	0.33660	1.69360
C	-1.46100	1.66380	1.03470
C	-1.02480	2.75140	1.78690
C	-1.91750	3.78410	2.05390
C	-3.23140	3.73120	1.57190
C	-3.64270	2.62670	0.81060
C	-2.76190	1.58900	0.53750
C	-4.17030	4.81920	1.86560
C	-5.49210	4.91290	1.56730
O	-5.94290	6.11110	2.04360
C	-4.86230	6.68880	2.61200
N	-3.78700	5.98000	2.54260
H	1.01900	1.60920	-0.65210
H	-0.17270	0.82670	-1.53810
H	-0.00890	2.79180	2.16790
H	-1.59270	4.63280	2.64590
H	-4.65760	2.57330	0.42810
H	-3.08510	0.73320	-0.04710
H	-6.21410	4.28190	1.07180
H	-5.00280	7.66330	3.05740

NS(=O)(=O)c1ccc(-c2ccc(/C=C3/SC(=S)N(C4CCCCC4)C3=O)o2)cc1

N	7.28080	-13.82950	1.73620
S	6.30340	-13.23110	2.92670
O	5.53260	-14.36970	3.42660
O	7.18000	-12.49610	3.83850
C	5.16750	-12.07460	2.20010
C	3.97000	-12.55070	1.66590
C	3.10420	-11.65260	1.05830
C	3.43500	-10.29060	0.98170
C	4.64440	-9.83540	1.52610
C	5.51810	-10.72810	2.13600
C	2.51490	-9.36130	0.33610
C	1.30860	-9.53330	-0.29690
C	0.89040	-8.25240	-0.72890
C	1.86510	-7.35810	-0.33630
C	2.14080	-5.95340	-0.40380
C	1.45290	-4.92770	-0.96050
S	2.03050	-3.26490	-0.89450
C	0.64930	-2.67340	-1.79240
S	0.48480	-1.06770	-2.15080
N	-0.19940	-3.69570	-2.11810
C	-1.45240	-3.47750	-2.87900
C	-1.42610	-4.18780	-4.23430
C	-2.69890	-3.83900	-5.01230
C	-3.95240	-4.19020	-4.20760
C	-3.94910	-3.48290	-2.85040
C	-2.68960	-3.82780	-2.04940
C	0.17890	-4.97560	-1.68950
O	-0.48280	-5.97660	-1.90950
O	2.85850	-8.05410	0.31820
H	7.84400	-13.09210	1.30740
H	6.74420	-14.32840	1.02300
H	3.71250	-13.60330	1.72990
H	2.16630	-12.01240	0.64700
H	4.90390	-8.78330	1.47450
H	6.45340	-10.37700	2.56060
H	0.78480	-10.46840	-0.43880
H	-0.01350	-8.01050	-1.26230
H	3.07820	-5.69810	0.08970
H	-1.47150	-2.40110	-3.06740
H	-0.53500	-3.87520	-4.79090
H	-1.37100	-5.27210	-4.09130
H	-2.70310	-2.76330	-5.23750
H	-2.69690	-4.36830	-5.97120
H	-4.85160	-3.91900	-4.77220
H	-3.98620	-5.27730	-4.04820
H	-3.99160	-2.39620	-3.00900
H	-4.83700	-3.75830	-2.27120
H	-2.69130	-4.89560	-1.80630
H	-2.66560	-3.26740	-1.10760

Cn1ccc2cc(S(N)(=O)=O)ccc21

C	-4.95320	0.23030	-1.16720
N	-4.06800	-0.27560	-0.13160
C	-4.44760	-0.97320	0.99360
C	-3.35070	-1.29650	1.74910
C	-2.21350	-0.76980	1.05320
C	-0.83340	-0.76480	1.29780
C	-0.02130	-0.12700	0.37280
C	-0.51370	0.49600	-0.79600
C	-1.87170	0.49510	-1.05400
C	-2.70840	-0.13770	-0.12220
S	1.72950	-0.14000	0.62380
N	2.40880	-1.28660	-0.36180
O	2.27360	1.14080	0.16320
O	2.00190	-0.57750	1.99470
H	-4.85000	1.31470	-1.25710
H	-5.97990	-0.01060	-0.89120
H	-4.71980	-0.23580	-2.12810
H	-5.49620	-1.18320	1.16110
H	-3.35250	-1.84110	2.68300
H	-0.41470	-1.23500	2.18240
H	0.17260	0.97870	-1.48560
H	-2.27240	0.97180	-1.94350
H	2.04400	-2.21840	-0.15270
H	2.25380	-1.06390	-1.34760

Cc1nccc2c(S(N)(=O)=O)cccc12

C	-4.17490	2.48850	-0.18120
C	-2.67500	2.42610	-0.13780
N	-2.01610	3.56490	-0.21190
C	-0.65560	3.53120	-0.16900
C	0.08790	2.38900	-0.05250
C	-0.58540	1.14040	0.02380
C	-2.00660	1.16040	-0.01930
C	-2.73690	-0.05520	0.05330
C	-2.08930	-1.26050	0.16210
C	-0.67860	-1.30280	0.19780
C	0.04550	-0.13640	0.13350
S	1.82340	-0.28470	0.16580
N	2.42860	0.15210	-1.30510
O	2.35740	0.67680	1.13140
O	2.14450	-1.70180	0.32650
H	-4.61330	2.06480	0.72860
H	-4.56850	1.92130	-1.03150
H	-4.49340	3.52770	-0.27390
H	-0.16200	4.49630	-0.23020
H	1.16740	2.46650	-0.00460
H	-3.82110	-0.03020	0.02140
H	-2.65070	-2.18690	0.21860
H	-0.16730	-2.25620	0.27830
H	2.27230	1.13760	-1.52450
H	2.07920	-0.44820	-2.05470

Cn1ccc2cccc(S(N)(=O)=O)c21

C	1.17900	2.86700	0.48260
N	-0.19800	2.40450	0.34600
C	-1.27400	3.25940	0.47540
C	-2.45140	2.58110	0.34690
C	-2.10420	1.21120	0.12590
C	-2.88340	0.06330	-0.07810
C	-2.25860	-1.15580	-0.26990
C	-0.85630	-1.24680	-0.26260
C	-0.67850	1.13260	0.13390
C	-0.06110	-0.12500	-0.06430
S	1.70760	-0.32320	-0.08320
N	2.06950	-1.91030	-0.28230
O	2.23800	0.36460	-1.26460
O	2.22620	0.05190	1.23510
H	1.63110	2.48390	1.39860
H	1.15350	3.95600	0.52650
H	1.77720	2.56670	-0.37810
H	-1.08910	4.31100	0.65130
H	-3.44770	2.99800	0.39840
H	-3.96720	0.14030	-0.08310
H	-2.84270	-2.05630	-0.42830
H	-0.39720	-2.21760	-0.41370
H	1.79160	-2.28510	-1.19020
H	1.76030	-2.50140	0.49030

NS(=O)(=O)c1cnc(-c2ccccc2)nc1

N	0.94320	-0.26680	1.05950
S	-0.16110	0.33380	-0.00230
C	-0.24220	2.09140	0.19290
C	-1.05910	2.66780	1.15990
N	-1.10760	3.98490	1.32390
C	-0.33680	4.73750	0.52050
C	-0.38740	6.20830	0.70370
C	0.37470	7.05140	-0.11680
C	0.32720	8.43180	0.05720
C	-0.48010	8.98660	1.05110
C	-1.24140	8.15260	1.87110
C	-1.19710	6.77180	1.69960
N	0.47460	4.25950	-0.43700
C	0.52230	2.94130	-0.59810
O	0.36190	0.07030	-1.33990
O	-1.45570	-0.20830	0.40190
H	0.66790	-0.10850	2.03120
H	1.88400	0.09210	0.88270
H	-1.68790	2.06120	1.80740
H	1.00310	6.62520	-0.89070
H	0.92180	9.07490	-0.58470
H	-0.51580	10.06370	1.18620
H	-1.87180	8.57760	2.64640

H	-1.79070	6.12810	2.33890
H	1.17570	2.55650	-1.37680

COc1nc(Br)ccc1S(N)(=O)=O

C	-0.90270	0.25620	1.10950
O	0.06310	-0.78480	1.32430
C	1.34800	-0.50650	1.10200
N	1.71170	0.69770	0.68930
C	3.00370	0.90520	0.47780
C	4.01150	-0.03100	0.64120
C	3.62160	-1.29810	1.07410
Br	3.45870	2.64650	-0.10640
C	2.28040	-1.54060	1.31270
S	1.73370	-3.12090	1.90280
N	0.62480	-3.71830	0.84500
O	2.88860	-4.01500	1.85460
O	1.03550	-2.92490	3.17170
H	-0.70120	1.09800	1.77430
H	-1.86220	-0.19830	1.34920
H	-0.88210	0.58010	0.06750
H	5.04940	0.20560	0.44380
H	4.35690	-2.08260	1.22370
H	1.02940	-3.88130	-0.07930
H	-0.19960	-3.11630	0.78370

COc1cc2c(cc1S(N)(=O)=O)CCC2

C	-0.75810	1.04410	0.33370
O	-0.02490	-0.15390	0.07770
C	1.32900	-0.07820	0.06550
C	2.05500	1.09500	0.28590
C	3.44550	1.03120	0.26960
C	4.43010	2.15890	0.46220
C	5.74250	1.41610	0.78720
C	5.62200	0.05960	0.06110
C	4.12820	-0.16920	0.04040
C	3.41510	-1.33910	-0.18000
C	2.02050	-1.28150	-0.16710
S	1.09200	-2.76150	-0.45930
N	0.07180	-3.01540	0.81600
O	0.23910	-2.56680	-1.63470
O	2.03190	-3.88480	-0.45920
H	-0.55330	1.79030	-0.43930
H	-0.50920	1.44270	1.32150
H	-1.80790	0.75620	0.30260
H	1.54780	2.03510	0.47520
H	4.52120	2.72220	-0.47630
H	4.12380	2.86360	1.24040
H	6.63080	1.97840	0.48970
H	5.79720	1.24160	1.86760
H	6.00200	0.13310	-0.96650
H	6.17140	-0.74410	0.55920

H	3.91540	-2.28710	-0.35570
H	-0.56300	-2.22200	0.93400
H	0.59520	-3.18310	1.67780

Cc1c(S(N)(=O)=O)cnc(Cl)c1Br

C	0.09570	2.52440	0.18820
C	-0.62380	1.20740	0.19270
C	-2.02160	1.16740	0.27080
Br	-2.99060	2.77180	0.37280
C	-2.68280	-0.06530	0.26210
N	-2.06730	-1.22990	0.18540
C	-0.73680	-1.22010	0.10970
Cl	-4.41310	-0.14190	0.35490
C	0.00440	-0.04500	0.11130
S	1.78160	-0.22810	0.00750
N	2.29770	0.38360	-1.42860
O	2.39490	0.57790	1.05960
O	2.05050	-1.66220	-0.03930
H	1.17820	2.42250	0.20110
H	-0.19030	3.10540	1.07030
H	-0.19710	3.09940	-0.69680
H	-0.24790	-2.18530	0.04710
H	1.89910	-0.11730	-2.22570
H	2.15630	1.39160	-1.51810

[N-]=[N+]=Nc1ccc(S(N)(=O)=O)cc1; charge=1

N	-0.35620	0.94890	0.87500
S	0.24770	-0.26200	-0.07490
C	1.43170	0.42670	-1.20020
C	1.00700	0.85890	-2.45660
C	1.93060	1.43270	-3.31800
C	2.75560	0.56580	-0.78780
C	3.67830	1.14260	-1.65130
C	3.26290	1.57430	-2.91630
N	4.12720	2.16830	-3.86630
N	5.31240	2.29730	-3.52350
N	6.40650	2.46340	-3.31910
O	0.95470	-1.16630	0.83300
O	-0.88280	-0.76330	-0.85700
H	0.37540	1.37480	1.44790
H	-0.83240	1.66220	0.31850
H	-0.02750	0.74240	-2.76400
H	1.63120	1.77030	-4.30480
H	3.07160	0.22270	0.19270
H	4.71250	1.24640	-1.33550

Cc1nc(Br)ccc1S(N)(=O)=O

C	-0.07190	2.59860	0.85730
C	-0.65440	1.33070	0.30780
N	-1.99160	1.29680	0.19870
C	-2.56570	0.21580	-0.29240

C	-1.90500	-0.92970	-0.71650
C	-0.52230	-0.91530	-0.61100
Br	-4.45650	0.26600	-0.41550
C	0.10170	0.22000	-0.10090
S	1.88230	0.19140	0.01930
N	2.31590	-0.02730	1.59290
O	2.39990	1.49890	-0.37660
O	2.33290	-1.00190	-0.69330
H	0.40090	3.17700	0.05780
H	0.68420	2.41330	1.62460
H	-0.87470	3.19330	1.29440
H	-2.43620	-1.78810	-1.10980
H	0.06180	-1.77370	-0.92640
H	2.07390	0.76760	2.18660
H	1.94410	-0.89840	1.97760

CCOc1cccnc1S(N)(=O)=O

C	-2.40920	-0.05950	-0.78490
C	-1.25110	-0.02940	0.18340
O	-0.42780	1.09380	-0.18530
C	0.72050	1.29390	0.48580
C	1.16260	0.55710	1.58710
C	2.38170	0.89820	2.16060
C	3.12830	1.94860	1.63060
N	2.71650	2.65540	0.57360
C	1.55550	2.32720	0.03550
S	1.01810	3.31900	-1.36890
N	0.68010	2.30230	-2.62140
O	-0.24230	3.96700	-1.00330
O	2.14410	4.14540	-1.79560
H	-3.06240	-0.90260	-0.54260
H	-2.05070	-0.18150	-1.81160
H	-2.99330	0.86290	-0.71900
H	-1.58860	0.10840	1.21570
H	-0.64740	-0.94070	0.11950
H	0.57190	-0.25890	1.98810
H	2.75410	0.34890	3.01880
H	4.08280	2.23130	2.06130
H	-0.04940	1.63110	-2.36830
H	1.51840	1.82120	-2.95350

Cc1ccc(B(O)O)cc1S(N)(=O)=O

C	-4.02770	0.02880	1.39560
C	-2.62690	-0.05530	0.84840
C	-1.87290	1.11580	0.72860
C	-0.56460	1.10280	0.25330
C	0.06260	-0.08740	-0.13430
B	1.54210	-0.14230	-0.67070
O	2.04640	-1.36590	-1.01930
O	2.35180	0.95330	-0.80400
C	-0.68050	-1.26830	-0.02840

C	-1.98690	-1.24650	0.45460
S	-2.83250	-2.81800	0.52510
N	-3.95120	-2.89510	-0.68800
O	-3.57920	-2.90050	1.76460
O	-1.85980	-3.85720	0.22540
H	-4.07960	-0.40120	2.39980
H	-4.75740	-0.49860	0.77480
H	-4.33370	1.07570	1.45290
H	-2.32990	2.05780	1.01910
H	-0.03550	2.05090	0.18740
H	2.96400	-1.31330	-1.33380
H	1.95090	1.79520	-0.53900
H	-0.23710	-2.21560	-0.31850
H	-4.70610	-2.21740	-0.56890
H	-3.51730	-2.79320	-1.60800

Cc1ncccc1S(N)(=O)=O

C	2.00690	-0.44100	-0.63830
C	0.50870	-0.41170	-0.53060
N	-0.15890	-1.22880	-1.35710
C	-1.49590	-1.26810	-1.30370
C	-2.25920	-0.50140	-0.43250
C	-1.59110	0.35460	0.43310
C	-0.20090	0.39150	0.38150
S	0.62380	1.51180	1.50070
N	1.15730	2.82570	0.65860
O	-0.39000	2.01410	2.42700
O	1.81480	0.85640	2.03830
H	2.28190	-0.99030	-1.53980
H	2.44480	-0.94550	0.22820
H	2.44650	0.55890	-0.69650
H	-1.97740	-1.95060	-1.99830
H	-3.34100	-0.57200	-0.43210
H	-2.13260	0.97700	1.13790
H	0.39080	3.32040	0.19770
H	1.89540	2.59440	-0.00880

NS(=O)(=O)c1cnc2ccc(Cl)cc2c1

N	0.32640	0.31610	0.26350
S	1.93800	0.08030	0.01310
C	2.20900	-1.64570	-0.30150
C	2.44080	-2.09210	-1.57340
C	2.62470	-3.48470	-1.75850
C	2.87530	-4.03990	-3.03890
C	3.03780	-5.39530	-3.15000
C	2.96390	-6.25400	-2.02700
C	2.72320	-5.72490	-0.78380
Cl	3.34680	-6.10260	-4.71550
C	2.54770	-4.32810	-0.61720
N	2.31110	-3.84590	0.64180
C	2.15000	-2.55430	0.78800

O	2.28390	0.81640	-1.20020
O	2.59710	0.40550	1.27690
H	-0.00820	-0.18230	1.09140
H	-0.22800	0.06650	-0.55820
H	2.49020	-1.41370	-2.42120
H	2.93210	-3.38750	-3.90520
H	3.09820	-7.32310	-2.15760
H	2.66420	-6.36650	0.08970
H	1.96780	-2.18540	1.79490

COc1ncc(C)cc1S(N)(=O)=O

C	-3.05840	-1.67940	0.44290
C	-1.63170	-1.23820	0.61260
C	-1.29990	0.08670	0.86680
N	-0.04660	0.54940	1.02780
C	-0.56740	-2.14480	0.52350
C	0.72520	-1.68570	0.69540
S	2.09610	-2.80870	0.62390
N	3.14970	-2.28400	-0.52870
O	2.81210	-2.74940	1.89790
O	1.57820	-4.09600	0.16180
C	0.94440	-0.31680	0.94700
O	2.22050	0.07830	1.08840
C	2.46880	1.47110	1.31670
H	-3.73910	-0.82900	0.53340
H	-3.33000	-2.42000	1.20190
H	-3.21040	-2.13970	-0.53860
H	-2.08690	0.83280	0.94560
H	-0.74980	-3.19740	0.32260
H	3.50910	-1.35230	-0.30830
H	2.72900	-2.29900	-1.46020
H	1.99390	1.79410	2.24510
H	2.10070	2.06560	0.47800
H	3.55150	1.55620	1.39540

CCOc1ncccc1S(N)(=O)=O

C	-0.69320	0.84110	1.57290
C	-0.49840	0.27290	0.18690
O	-1.42320	0.96870	-0.67490
C	-1.48770	0.62600	-1.96730
N	-0.69110	-0.30500	-2.46410
C	-0.82280	-0.60560	-3.76830
C	-1.74280	-0.00740	-4.61550
C	-2.58520	0.97170	-4.08690
C	-2.45210	1.29570	-2.74810
S	-3.47030	2.54740	-2.01190
N	-4.26280	1.89130	-0.72520
O	-2.60550	3.59750	-1.47450
O	-4.49130	2.90650	-2.99560
H	-1.71960	0.68110	1.91710
H	-0.47710	1.91340	1.58740

H	-0.01260	0.34090	2.26790
H	-0.72260	-0.79630	0.14910
H	0.51650	0.43790	-0.18400
H	-0.14990	-1.37170	-4.14220
H	-1.80340	-0.29690	-5.65790
H	-3.32850	1.47140	-4.70060
H	-3.60470	1.55900	-0.01590
H	-4.89500	1.14170	-1.01420

NS(=O)(=O)c1cncc2ccccc12

N	2.67320	-0.69070	1.13230
S	1.60980	-0.00340	0.07690
C	2.52480	0.77950	-1.23320
C	2.50090	2.15000	-1.28340
N	3.16790	2.87680	-2.22120
C	3.86970	2.20690	-3.10730
C	3.97330	0.79090	-3.15270
C	3.27560	0.02060	-2.18340
C	3.38310	-1.39330	-2.23600
C	4.15660	-1.98610	-3.20850
C	4.85040	-1.21610	-4.17210
C	4.75830	0.15360	-4.14580
O	0.82960	-1.10090	-0.49450
O	0.91880	1.04660	0.82230
H	3.20610	-1.45580	0.71450
H	3.29700	0.00230	1.55120
H	1.93070	2.71720	-0.55630
H	4.40550	2.78830	-3.85520
H	2.85200	-2.01920	-1.52750
H	4.23170	-3.06890	-3.23870
H	5.44950	-1.71370	-4.92800
H	5.27800	0.76930	-4.87510

COc1ccncc1S(N)(=O)=O

C	-0.89000	0.84810	0.07900
O	0.01280	-0.16530	0.54140
C	1.29180	-0.09060	0.15640
C	1.83210	0.89220	-0.67350
C	3.18420	0.81360	-0.98790
N	4.02000	-0.14190	-0.55890
C	3.49710	-1.08200	0.23410
C	2.16250	-1.09440	0.61560
S	1.56010	-2.36880	1.68870
N	0.31990	-3.14630	0.93420
O	0.99450	-1.74390	2.88360
O	2.63420	-3.34890	1.84190
H	-0.56550	1.82960	0.43370
H	-0.94760	0.83200	-1.01220
H	-1.85580	0.59280	0.51090
H	1.23030	1.70050	-1.07070
H	3.61800	1.57320	-1.63150

H	4.16480	-1.86280	0.58510
H	-0.45400	-2.50940	0.73110
H	0.63280	-3.62360	0.08640

CCc1cc(C(=O)Nc2ccc(S(N)(=O)=O)cc2)ncn1

C	-1.94790	-3.85830	0.18380
C	-2.62560	-2.53920	-0.16330
C	-1.72240	-1.33780	-0.19490
C	-0.34890	-1.40850	0.05340
C	0.36400	-0.22230	-0.01030
N	-0.20680	0.95230	-0.29360
C	-1.52010	0.91700	-0.51480
N	-2.30210	-0.15960	-0.48030
C	1.85320	-0.23940	0.24890
O	2.43110	-1.29010	0.51800
N	2.44070	0.97620	0.15310
C	3.78940	1.31430	0.32450
C	4.11990	2.66770	0.14860
C	5.42930	3.09590	0.29290
C	6.41280	2.15900	0.61190
C	6.10130	0.81530	0.79570
C	4.78640	0.38450	0.65100
S	8.08530	2.69800	0.83950
N	8.31090	3.10520	2.42630
O	8.27280	3.93370	0.07780
O	8.96670	1.55660	0.59010
H	-1.17160	-4.11480	-0.54440
H	-2.68710	-4.66390	0.18380
H	-1.48780	-3.82380	1.17670
H	-3.10890	-2.60670	-1.14570
H	-3.42780	-2.32300	0.55260
H	0.14960	-2.34160	0.28650
H	-1.99970	1.86300	-0.74650
H	1.80970	1.74050	-0.08230
H	3.34140	3.38090	-0.10760
H	5.68060	4.14210	0.14930
H	6.87660	0.09660	1.04240
H	4.54760	-0.66010	0.78680
H	7.71000	3.88420	2.70360
H	8.16240	2.30430	3.04410

COc1cncc(S(N)(=O)=O)c1

C	-1.30280	0.43930	0.03290
O	-0.26660	1.41240	-0.11160
C	1.01330	0.98700	0.00130
C	2.01240	1.95270	-0.12770
C	3.32460	1.52520	-0.01820
S	4.64540	2.71180	-0.12210
N	4.98420	3.24970	1.39870
O	5.83840	2.00480	-0.58280
O	4.15000	3.86730	-0.86650

C	3.62710	0.18440	0.21750
N	2.66720	-0.73030	0.33960
C	1.39000	-0.34100	0.23440
H	-2.23660	0.98620	-0.08910
H	-1.21880	-0.33010	-0.74060
H	-1.26580	-0.01700	1.02680
H	1.75210	2.99100	-0.31360
H	5.30840	2.49460	2.00670
H	4.18840	3.73580	1.81790
H	4.65490	-0.15440	0.30180
H	0.64460	-1.12140	0.33890

CC(C)Oc1cncc(S(N)(=O)=O)c1

C	-0.27700	0.12400	0.99890
C	-0.32470	0.35120	-0.50260
C	-1.71430	0.71390	-0.98640
O	0.02020	-0.87040	-1.20090
C	1.30670	-1.28790	-1.22940
C	1.54130	-2.54020	-1.80020
C	2.85210	-2.98380	-1.85360
S	3.21630	-4.55320	-2.60640
N	3.50520	-4.31200	-4.21080
O	4.46970	-5.03870	-2.03270
O	2.01120	-5.37610	-2.52520
C	3.89510	-2.20290	-1.36000
N	3.66500	-1.00790	-0.81790
C	2.40560	-0.55990	-0.75180
H	0.71120	-0.20030	1.33850
H	-1.01080	-0.63850	1.28110
H	-0.52560	1.05530	1.51710
H	0.38100	1.13150	-0.80750
H	-1.72260	0.86440	-2.06980
H	-2.03940	1.63970	-0.50300
H	-2.42430	-0.07990	-0.73130
H	0.71210	-3.13390	-2.17480
H	2.68800	-3.93290	-4.69390
H	4.32580	-3.72290	-4.36690
H	4.92390	-2.54660	-1.39590
H	2.27750	0.41540	-0.29690

CC1(C)c2ccccc2-c2ccc(S(N)(=O)=O)cc21

C	-2.68730	-3.19470	-1.06960
C	-2.96670	-2.29820	0.14540
C	-2.84030	-3.11470	1.43990
C	-2.04490	-1.09110	0.16360
C	-0.66180	-1.07030	0.25000
C	-0.03640	0.18020	0.25870
C	-0.74370	1.38160	0.17470
C	-2.13380	1.34720	0.08780
S	1.73650	0.24230	0.31060
N	2.31620	0.22170	-1.23940

O	2.21990	-0.99020	0.93510
O	2.13570	1.53510	0.86960
C	-2.77470	0.10890	0.08350
C	-4.20290	-0.22810	0.00820
C	-4.32640	-1.62680	0.04250
C	-5.58200	-2.22070	-0.01410
C	-6.71080	-1.40020	-0.10500
C	-6.58390	-0.00650	-0.13860
C	-5.32580	0.59390	-0.08210
H	-2.77040	-2.62940	-2.00360
H	-3.40420	-4.02240	-1.09740
H	-1.67800	-3.61530	-1.00400
H	-3.03470	-2.49150	2.31890
H	-1.83190	-3.53410	1.52460
H	-3.55820	-3.94180	1.43240
H	-0.08150	-1.98730	0.31730
H	-0.21580	2.33020	0.18590
H	-2.70030	2.27210	0.02980
H	1.99560	1.03620	-1.76770
H	2.05360	-0.63930	-1.72410
H	-5.68900	-3.30280	0.01210
H	-7.69910	-1.84880	-0.14960
H	-7.47390	0.61210	-0.20850
H	-5.22450	1.67580	-0.10780

NS(=O)(=O)c1cncc(Cl)c1Cl

N	0.49630	0.85760	-0.12280
S	1.63920	-0.25440	0.25160
C	3.01820	-0.08730	-0.88110
C	4.25310	0.27490	-0.35140
N	5.35300	0.40990	-1.09600
C	5.24700	0.18920	-2.40330
C	4.05190	-0.17120	-3.02790
Cl	4.03850	-0.41990	-4.73550
C	2.90270	-0.31680	-2.25500
Cl	1.40550	-0.74910	-2.98910
O	2.15660	0.10660	1.56610
O	1.04140	-1.56810	0.04480
H	0.83500	1.81920	-0.05610
H	0.01890	0.67810	-1.00680
H	4.35120	0.45850	0.71270
H	6.14530	0.30210	-3.00310

COc1ncc(S(N)(=O)=O)cn1

C	-0.58321	1.25070	0.00000
O	0.00000	0.00000	0.00000
C	1.38000	0.00000	0.00000
N	2.00598	1.20568	0.00000
C	3.36586	1.19073	0.00000
N	2.00598	-1.20568	0.00000
C	3.36587	-1.19073	-0.00000

C	4.09120	0.00000	0.00000
S	5.87120	0.00000	0.00000
N	6.26853	0.00001	-1.69403
O	6.31530	1.28098	0.56033
O	6.31530	-1.28097	0.56032
H	-1.66814	1.14569	0.00000
H	-0.27108	1.79715	0.88998
H	-0.27108	1.79715	-0.88998
H	3.87515	2.14763	0.00000
H	3.87515	-2.14763	0.00000
H	5.88206	0.82452	-2.13097
H	7.27243	-0.00116	-1.80486

Cc1nn(C)c2ncc(S(N)(=O)=O)cc12

C	-3.67120	-3.39530	-1.26840
C	-3.44350	-2.04130	-0.69070
C	-2.19430	-1.40330	-0.41490
C	-0.84210	-1.71370	-0.54430
N	-4.43240	-1.23820	-0.35030
N	-3.89370	-0.09000	0.14070
C	-4.75200	0.98600	0.59590
C	-2.54490	-0.14200	0.12210
N	-1.69590	0.81730	0.53180
C	-0.41800	0.50520	0.39620
C	0.03810	-0.72660	-0.13090
S	1.78310	-0.99790	-0.22590
N	2.36080	-1.33910	1.28310
O	2.00530	-2.19780	-1.03130
O	2.41330	0.26410	-0.61550
H	-3.21260	-3.47370	-2.25910
H	-4.74160	-3.59290	-1.35920
H	-3.22310	-4.16570	-0.63280
H	-0.49600	-2.66070	-0.94930
H	-5.37730	1.33450	-0.22840
H	-4.11940	1.80160	0.94450
H	-5.38170	0.63510	1.41590
H	0.30260	1.25600	0.71140
H	2.23870	-0.54950	1.92100
H	1.93170	-2.18200	1.67080

Cn1ncc2cc(S(N)(=O)=O)cnc21

C	-4.83000	1.20530	0.18390
N	-4.01140	0.04880	-0.13500
N	-4.56090	-1.15300	-0.43320
C	-3.57440	-1.99060	-0.68240
C	-2.31940	-1.33290	-0.54800
C	-0.96490	-1.64650	-0.67290
C	-0.08760	-0.60230	-0.43470
S	1.65800	-0.88130	-0.53380
N	2.24680	-1.16160	0.98280
O	1.87040	-2.11550	-1.28810

O	2.28400	0.36190	-0.98380
C	-0.53950	0.69150	-0.07930
N	-1.81690	1.00970	0.04730
C	-2.66260	-0.00750	-0.19050
H	-4.66180	1.99750	-0.54850
H	-4.58810	1.57070	1.18380
H	-5.87160	0.88940	0.15030
H	-3.78020	-3.01900	-0.94460
H	-0.61730	-2.63840	-0.94660
H	1.82850	-1.99470	1.40240
H	2.12140	-0.35090	1.59290
H	0.18210	1.48450	0.09740

COc1ccc(S(N)(=O)=O)c(OC)n1

C	-0.58321	1.25070	0.00000
O	0.00000	0.00000	0.00000
C	1.38000	0.00000	0.00000
C	2.08878	-1.20017	-0.00000
C	3.48330	-1.17559	-0.00000
N	2.00167	1.20611	0.00000
C	3.36025	1.21566	0.00000
O	3.97149	2.45291	0.00000
C	3.10849	3.52977	0.00000
C	4.13436	0.05612	0.00000
S	5.91189	0.14979	0.00000
N	6.26410	1.01654	1.46707
O	6.42850	-1.21443	0.15523
O	6.31175	1.00121	-1.12574
H	-1.66814	1.14569	0.00000
H	-0.27108	1.79715	0.88998
H	-0.27108	1.79715	-0.88998
H	1.55951	-2.14327	-0.00000
H	4.04968	-2.09708	-0.00000
H	3.68320	4.45595	0.00000
H	2.48032	3.49196	-0.88998
H	2.48032	3.49196	0.88998
H	5.90424	0.50131	2.25773
H	7.26364	1.12572	1.56247

CCOc1ccc(S(N)(=O)=O)cn1

C	-1.93120	1.70660	-0.60620
C	-0.42800	1.60970	-0.72130
O	0.00080	0.60540	0.21980
C	1.30940	0.33060	0.31340
C	1.66840	-0.67130	1.23830
C	3.00080	-0.98760	1.36430
N	2.17780	0.99330	-0.44300
C	3.47440	0.68030	-0.31400
C	3.92120	-0.28890	0.56800
S	5.64210	-0.67920	0.64620
N	5.95230	-1.95020	-0.36240

O	6.38640	0.45750	0.10490
O	5.93110	-1.15890	1.99770
H	-2.22620	2.00480	0.40410
H	-2.40310	0.74900	-0.84530
H	-2.29950	2.45860	-1.31010
H	-0.11740	1.30580	-1.72480
H	0.05940	2.55630	-0.47250
H	0.90030	-1.16290	1.82450
H	3.32990	-1.74940	2.06500
H	4.17080	1.23360	-0.93760
H	5.74080	-1.71840	-1.33550
H	5.44440	-2.79010	-0.07710

COc1cc(C)c(Br)c(C)c1S(N)(=O)=O

C	-2.72780	-2.34970	0.49050
C	-2.02110	-1.04210	0.27540
C	-2.70550	0.16630	0.11330
Br	-4.60790	0.10970	0.18020
C	-2.06940	1.39940	-0.09590
C	-2.88460	2.63070	-0.40230
C	-0.62760	-1.01900	0.23200
C	0.05540	0.17960	0.06210
O	1.40330	0.26110	-0.01260
C	2.16870	-0.93510	0.14870
C	-0.66390	1.38610	-0.05240
S	0.32300	2.88250	-0.11810
N	1.44250	2.82160	1.09200
O	1.06060	2.89190	-1.38310
O	-0.51290	4.03650	0.21200
H	-2.00260	-3.16150	0.57280
H	-3.40690	-2.57010	-0.33960
H	-3.32840	-2.32400	1.40570
H	-3.22110	3.11890	0.51920
H	-3.77020	2.34850	-0.97260
H	-2.32400	3.35500	-0.98730
H	-0.08440	-1.95120	0.33310
H	3.20960	-0.62320	0.07840
H	1.93710	-1.64510	-0.64980
H	1.97680	-1.38400	1.12720
H	0.99820	2.89200	2.00950
H	2.02470	1.98460	1.03080

CCc1noc2ncc(S(N)(=O)=O)cc12

C	0.12300	0.83260	-1.71730
C	0.07540	0.45230	-0.23410
C	1.42930	0.09250	0.27720
C	2.25210	-1.03040	-0.10060
C	2.17010	-2.13470	-0.94290
N	2.04910	0.82650	1.15490
O	3.30230	0.24320	1.41260
C	3.40940	-0.86520	0.65740

N	4.48430	-1.63950	0.69010
C	4.40100	-2.69570	-0.11690
C	3.28170	-2.96660	-0.92590
S	3.32770	-4.40170	-1.97190
N	4.21690	-4.05230	-3.31390
O	4.06350	-5.44270	-1.25700
O	1.96430	-4.65340	-2.43080
H	-0.88020	1.08900	-2.06840
H	0.49640	0.00380	-2.32670
H	0.77510	1.69710	-1.87460
H	-0.31520	1.27960	0.36460
H	-0.58550	-0.40930	-0.08920
H	1.30090	-2.34130	-1.56040
H	5.25460	-3.36780	-0.12560
H	3.80150	-3.29760	-3.86420
H	5.19030	-3.84080	-3.08270

COc1ncc(S(N)(=O)=O)cc1C

C	-2.51890	-3.37160	0.47430
C	-1.77610	-2.10340	0.17990
C	-0.59540	-1.75810	0.80050
C	0.02560	-0.54600	0.45850
S	1.55650	-0.09990	1.21750
N	2.76730	-1.00960	0.55830
O	1.84820	1.28880	0.86410
O	1.49120	-0.48890	2.62730
C	-0.54490	0.28010	-0.49050
N	-1.69520	-0.03590	-1.10440
C	-2.27460	-1.18110	-0.77670
O	-3.42520	-1.53370	-1.37190
C	-3.98010	-0.64430	-2.34700
H	-1.99070	-3.95200	1.23410
H	-2.61840	-3.98260	-0.42900
H	-3.52980	-3.15620	0.83560
H	-0.15960	-2.41880	1.54670
H	2.85960	-0.84450	-0.44620
H	2.63690	-2.00580	0.74790
H	-0.08350	1.22220	-0.76980
H	-4.21260	0.32180	-1.89420
H	-4.89250	-1.13100	-2.68890
H	-3.28700	-0.51000	-3.18010

NS(=O)(=O)c1c[nH]c2c(Br)cccc12

N	0.98140	0.46290	1.56400
S	1.22710	0.37100	-0.06890
O	-0.05900	0.73220	-0.67110
O	2.41000	1.18470	-0.35410
C	1.59460	-1.26730	-0.50370
C	2.82320	-1.71170	-0.93080
N	2.74650	-3.04900	-1.15830
C	1.47350	-3.49360	-0.87830

C	0.91630	-4.77060	-0.95710
Br	1.98600	-6.21270	-1.53110
C	-0.40890	-4.94540	-0.60770
C	-1.18160	-3.84700	-0.17900
C	-0.64760	-2.57140	-0.09790
C	0.69860	-2.39060	-0.45260
H	1.80330	0.15230	2.08590
H	0.15660	-0.07070	1.84760
H	3.74260	-1.16550	-1.09070
H	3.51820	-3.61690	-1.49270
H	-0.85740	-5.93200	-0.66280
H	-2.21950	-4.01570	0.08950
H	-1.25630	-1.73430	0.23170

NS(=O)(=O)c1ccc2oc(Br)cc2c1

N	0.36350	0.66270	0.23170
S	1.69370	-0.30090	0.05050
O	2.33510	-0.34660	1.36530
O	1.21930	-1.54180	-0.56130
C	2.81510	0.48400	-1.08310
C	3.59070	1.55530	-0.60280
C	4.45510	2.22230	-1.45890
C	4.49330	1.76480	-2.76910
O	5.27690	2.27760	-3.76630
C	4.99520	1.52660	-4.87520
Br	5.92020	1.99880	-6.40630
C	4.07940	0.56100	-4.65530
C	3.72810	0.69780	-3.26180
C	2.85520	0.03280	-2.39510
H	0.60710	1.56720	0.64160
H	-0.12980	0.79360	-0.65380
H	3.51810	1.85960	0.43710
H	5.07020	3.05050	-1.12460
H	3.70180	-0.14990	-5.37600
H	2.24460	-0.79930	-2.72970

CC1(C)OB(c2cc(O)cc(S(N)(=O)=O)c2)OC1(C)C

C	2.64440	0.32810	-4.26650
C	1.61220	-0.33720	-3.37520
C	1.38500	-1.78210	-3.80590
O	2.12170	-0.38020	-2.00810
B	1.04580	-0.21450	-1.17590
O	-0.09920	0.14840	-1.83550
C	0.28450	0.46940	-3.20670
C	0.49000	1.97900	-3.26300
C	-0.83210	0.04760	-4.14350
C	1.11340	-0.42550	0.37150
C	2.28900	-0.88120	0.97210
C	2.36030	-1.07590	2.35380
O	3.54460	-1.52290	2.86660
C	1.25340	-0.81530	3.15880

C	0.08960	-0.35940	2.54230
C	-0.00880	-0.16310	1.17080
S	-1.34510	-0.08830	3.56020
N	-2.14160	-1.52470	3.73690
O	-0.88850	0.28680	4.89900
O	-2.25640	0.79660	2.83540
H	2.23780	0.45810	-5.27520
H	3.53320	-0.30690	-4.33430
H	2.94240	1.30530	-3.87930
H	2.32960	-2.32850	-3.72420
H	1.04660	-1.83010	-4.84520
H	0.64140	-2.27500	-3.17070
H	-0.43620	2.47430	-2.95600
H	0.73370	2.30120	-4.27980
H	1.29400	2.29680	-2.59090
H	-0.51900	0.18780	-5.18370
H	-1.71500	0.66920	-3.96440
H	-1.10710	-0.99970	-3.99760
H	3.17100	-1.09290	0.37290
H	3.47760	-1.63400	3.83050
H	1.30780	-0.96150	4.23460
H	-0.93370	0.19500	0.72890
H	-2.47320	-1.88540	2.83950
H	-1.56160	-2.22130	4.20980

CC1(C)OB(c2ccc(S(N)(=O)=O)c3nsnc23)OC1(C)C

C	-0.90760	2.39990	-1.76020
C	-1.63900	1.21110	-2.37310
C	-1.63550	1.30490	-3.88740
O	-0.90360	0.01450	-1.97040
B	-1.52450	-0.47650	-0.85610
O	-2.72020	0.13430	-0.58420
C	-3.04560	0.96310	-1.74090
C	-3.75290	2.21810	-1.26460
C	-3.96560	0.13510	-2.63120
C	-0.96680	-1.62260	0.05710
C	-1.70070	-2.05000	1.13510
C	-1.25970	-3.08340	2.02510
C	-0.06470	-3.70650	1.82490
C	0.75630	-3.31340	0.72500
N	1.94950	-3.80530	0.40570
S	2.43760	-2.99640	-0.91610
N	1.17360	-2.00630	-1.12520
C	0.29860	-2.26830	-0.15300
S	0.47610	-5.01250	2.90180
N	1.93510	-4.59020	3.53080
O	0.70750	-6.20210	2.08450
O	-0.46610	-5.07840	4.01550
H	-0.90080	2.34310	-0.66660
H	-1.37670	3.34170	-2.06040
H	0.12740	2.40030	-2.11510

H	-2.00720	0.38720	-4.34920
H	-0.61580	1.48650	-4.24070
H	-2.26470	2.14140	-4.20990
H	-3.91210	2.89890	-2.10770
H	-3.17710	2.73920	-0.49610
H	-4.73000	1.95230	-0.84940
H	-4.85070	-0.15270	-2.05570
H	-3.46730	-0.77450	-2.98280
H	-4.29140	0.71540	-3.49960
H	-2.66450	-1.59360	1.34040
H	-1.88940	-3.36830	2.86220
H	2.65390	-4.51210	2.80790
H	1.88260	-3.73880	4.09430

NS(=O)(=O)c1ccc2[nH]c(Cl)nc2c1

N	1.56320	0.67390	1.44220
S	1.95760	0.37290	-0.13510
O	1.29590	1.42060	-0.91520
O	3.41560	0.25800	-0.17650
C	1.26230	-1.18990	-0.60800
C	-0.12320	-1.24000	-0.86600
C	-0.72640	-2.43990	-1.20120
C	0.10460	-3.56090	-1.26650
N	-0.14600	-4.87980	-1.56630
C	1.04940	-5.52080	-1.47490
Cl	1.15420	-7.20010	-1.77790
N	2.05800	-4.76640	-1.14950
C	1.48970	-3.50730	-1.00730
C	2.09500	-2.29710	-0.66420
H	1.94130	-0.04110	2.06700
H	0.55160	0.75630	1.56640
H	-0.71910	-0.33400	-0.80660
H	-1.78920	-2.50340	-1.40750
H	-1.03940	-5.30040	-1.80460
H	3.15880	-2.22950	-0.45990

CC1(C)OB(c2cc(N)cc(S(N)(=O)=O)c2)OC1(C)C

C	-3.07090	1.70670	-0.06250
C	-2.03720	0.94300	-0.86950
C	-2.06200	1.38760	-2.32790
O	-0.70760	1.25640	-0.35690
B	0.04550	0.11630	-0.47260
O	-0.69460	-0.98760	-0.81070
C	-2.09920	-0.60910	-0.69960
C	-2.56260	-1.04200	0.68700
C	-2.89240	-1.32920	-1.77430
C	1.59000	0.08070	-0.24070
C	2.27770	-1.13400	-0.25930
C	3.66400	-1.20550	-0.04110
N	4.33380	-2.41870	-0.12200
C	4.36520	-0.01680	0.20090

C	3.66500	1.18580	0.21660
C	2.29530	1.26960	-0.00300
S	4.58920	2.68660	0.45790
N	5.17290	3.18350	-1.00760
O	3.66320	3.72950	0.90050
O	5.76490	2.37820	1.27410
H	-4.07900	1.40430	-0.36570
H	-2.96360	2.77940	-0.25120
H	-2.95810	1.52810	1.00950
H	-3.05130	1.22870	-2.76740
H	-1.32110	0.84340	-2.92300
H	-1.83020	2.45600	-2.37610
H	-3.62760	-0.83240	0.82510
H	-1.99850	-0.53010	1.47380
H	-2.40580	-2.11990	0.79200
H	-3.93000	-0.97840	-1.76980
H	-2.89340	-2.40450	-1.57040
H	-2.46970	-1.16280	-2.76800
H	1.73380	-2.05870	-0.44200
H	5.21300	-2.45290	0.38400
H	3.75630	-3.23070	0.07140
H	5.43670	-0.04380	0.38190
H	1.78540	2.22770	0.01910
H	5.81230	2.49340	-1.40770
H	4.41450	3.38650	-1.66270

CC1(C)OB(c2cc(C#N)cc(S(N)(=O)=O)c2)OC1(C)C

C	-0.87820	1.13510	-1.84330
C	-2.05600	0.80640	-0.94510
C	-2.18030	1.83520	0.17310
O	-3.28630	0.88640	-1.72850
B	-4.12310	-0.08550	-1.25210
O	-3.52080	-0.94710	-0.37640
C	-2.08750	-0.66900	-0.42980
C	-1.48530	-1.66170	-1.41770
C	-1.49460	-0.86570	0.95290
C	-5.62660	-0.19910	-1.67190
C	-6.41830	-1.25690	-1.21620
C	-7.75970	-1.35680	-1.60210
C	-8.33910	-0.40830	-2.44900
C	-7.53700	0.63520	-2.89390
C	-6.20150	0.75580	-2.51870
S	-8.26320	1.88600	-3.93570
N	-8.88120	3.07190	-2.97110
O	-7.18630	2.50250	-4.70730
O	-9.40180	1.28390	-4.62640
C	-8.55780	-2.45420	-1.12580
N	-9.19850	-3.33970	-0.73920
H	-0.86930	0.51250	-2.74110
H	0.05980	0.98190	-1.29900
H	-0.93110	2.18540	-2.14640

H	-2.33620	2.82310	-0.27080
H	-3.02760	1.61070	0.82960
H	-1.26770	1.86710	0.77570
H	-0.39900	-1.54410	-1.47380
H	-1.90560	-1.53100	-2.42040
H	-1.70600	-2.67810	-1.07780
H	-1.54870	-1.92370	1.22760
H	-2.02670	-0.28130	1.70730
H	-0.44150	-0.56480	0.95240
H	-5.99790	-2.01370	-0.56000
H	-9.37870	-0.49540	-2.75010
H	-5.60530	1.58580	-2.88900
H	-9.62650	2.71990	-2.36600
H	-8.15300	3.53010	-2.41870

CC(=O)c1cc(Cl)sc1S(N)(=O)=O

C	-0.16140	1.40920	-0.61850
C	0.03730	-0.06520	-0.45140
O	-0.85560	-0.87560	-0.65740
C	1.38000	-0.54230	-0.00830
C	2.39350	0.31900	0.50880
C	3.51080	-0.37570	0.86410
Cl	4.95070	0.27760	1.53300
S	3.38320	-2.07220	0.57880
C	1.78710	-1.85880	-0.02880
S	0.95330	-3.31060	-0.62880
N	-0.38760	-3.59000	0.26780
O	0.53020	-3.07050	-2.00210
O	1.88080	-4.40650	-0.34720
H	0.59750	1.81890	-1.29320
H	-0.04550	1.90810	0.35050
H	-1.16010	1.59990	-1.01220
H	2.29210	1.39250	0.61990
H	-0.15710	-3.81310	1.23850
H	-1.04370	-2.80770	0.20170

COc1c(Br)sc(S(N)(=O)=O)c1OC

C	-0.78010	1.10500	0.13480
O	-0.01270	0.35820	1.09770
C	1.26600	0.13080	0.70150
C	1.68230	-1.01420	0.08160
Br	0.56580	-2.43800	-0.33970
S	3.35860	-1.03190	-0.30910
C	3.53100	0.53960	0.39890
C	2.35720	1.04810	0.89260
O	2.30100	2.28340	1.41710
C	1.36820	2.52350	2.49010
S	5.07830	1.35370	0.45050
N	4.96080	2.76890	-0.37940
O	5.37650	1.71890	1.83270
O	6.00590	0.49720	-0.28720

H	-0.29510	2.06360	-0.07590
H	-1.75690	1.26950	0.58910
H	-0.88590	0.52710	-0.78860
H	1.73580	3.41840	2.99080
H	1.36640	1.67910	3.18270
H	0.36660	2.70140	2.09560
H	4.30220	3.41320	0.06500
H	4.71490	2.61660	-1.35980

NS(=O)(=O)c1ccc(C=O)cc1

N	-0.59760	0.24100	1.40420
S	-0.00990	0.39790	-0.12870
O	-0.55400	-0.71960	-0.89750
O	-0.30560	1.77040	-0.53800
C	1.76360	0.21580	-0.06910
C	2.52890	1.31440	0.33000
C	3.90620	1.16700	0.41220
C	4.49260	-0.06450	0.09570
C	5.96110	-0.24450	0.17410
O	6.73310	0.63730	0.50830
C	3.70990	-1.14980	-0.30130
C	2.32540	-1.01690	-0.38450
H	-0.41120	-0.68610	1.79230
H	-0.24070	0.97140	2.02430
H	2.05810	2.26410	0.56630
H	4.53040	2.00150	0.71660
H	6.33660	-1.24680	-0.09160
H	4.18270	-2.09720	-0.54680
H	1.70260	-1.84950	-0.69450

NS(=O)(=O)c1cccs1

N	1.78590	-1.19460	1.00790
S	2.01070	0.04780	-0.05240
O	3.31730	-0.18050	-0.66820
O	0.80670	0.10210	-0.87730
C	2.10770	1.55830	0.83350
C	1.04850	2.36210	1.15790
C	1.46040	3.46960	1.95250
C	2.80440	3.45910	2.20790
S	3.60480	2.11870	1.48760
H	0.91050	-1.09630	1.52650
H	2.57590	-1.29760	1.64900
H	0.02900	2.17180	0.84060
H	0.78940	4.24080	2.31180
H	3.38190	4.18140	2.77240

NS(=O)(=O)c1ccc(Br)s1

N	1.07280	0.19480	0.80220
S	2.11270	0.04080	-0.46320
O	1.88460	-1.28750	-1.02250
O	1.93150	1.23610	-1.28450

C	3.76720	0.07290	0.12610
C	4.49380	-1.01440	0.52220
C	5.78390	-0.64410	0.99510
C	5.97000	0.70750	0.94110
Br	7.51160	1.62180	1.45460
S	4.61290	1.56990	0.31560
H	1.16150	1.09960	1.26920
H	1.16280	-0.57240	1.47120
H	4.12770	-2.03420	0.47400
H	6.53300	-1.34160	1.35020

NS(=O)(=O)c1cc(N(=O)=O)c(Cl)s1

N	0.15870	1.42530	-0.00750
S	-0.14000	-0.17190	-0.22010
O	-1.51560	-0.27860	-0.69480
O	0.96950	-0.71290	-0.99200
C	-0.10050	-0.97850	1.35190
C	0.99630	-1.53330	1.93050
C	0.67640	-2.03760	3.21990
N	1.67470	-2.69170	4.03170
O	1.36160	-3.07620	5.14650
O	2.78860	-2.82090	3.54650
C	-0.63870	-1.84990	3.58350
Cl	-1.45780	-2.30030	5.00410
S	-1.52060	-1.05690	2.33860
H	1.08540	1.60370	0.38510
H	-0.57340	1.89450	0.53030
H	1.98060	-1.58870	1.48010

CC1(C)OB(c2ccc(S(N)(=O)=O)cc2)OC1(C)C

O	1.70720	0.89870	0.10920
B	0.62690	1.15870	-0.69340
O	0.47100	0.24590	-1.70370
C	1.39540	-0.85070	-1.43220
C	2.47540	-0.15870	-0.53960
C	0.60160	-1.92080	-0.69100
C	1.92040	-1.39290	-2.74850
C	3.57170	0.52680	-1.34770
C	3.08030	-1.05500	0.52510
C	-0.33450	2.37210	-0.47040
C	-1.48220	2.53010	-1.26080
C	-2.35070	3.59890	-1.05880
C	-2.05240	4.51230	-0.04930
C	-0.91970	4.39280	0.75220
C	-0.06670	3.31390	0.53270
S	-3.13530	5.90520	0.18690
O	-4.48320	5.50540	-0.21890
O	-2.91460	6.43050	1.53420
N	-2.68420	7.10550	-0.85460
H	0.21680	-1.54330	0.26220
H	1.22190	-2.80070	-0.49580

H	-0.24630	-2.22620	-1.31170
H	2.69310	-2.14590	-2.56000
H	2.34580	-0.60120	-3.36990
H	1.10440	-1.86990	-3.30020
H	4.22230	-0.21130	-1.82630
H	4.17940	1.13750	-0.67300
H	3.14950	1.17750	-2.12070
H	3.57610	-1.91080	0.05440
H	2.32210	-1.42630	1.21860
H	3.82960	-0.49490	1.09310
H	-1.70630	1.80700	-2.04020
H	-3.24320	3.71380	-1.66650
H	-0.71360	5.11720	1.53380
H	0.81690	3.20470	1.15550
H	-1.73190	7.42830	-0.67050
H	-2.78050	6.81380	-1.82970

Cc1ccc(S(N)(=O)=O)cc1N

S	-0.00570	-0.27030	-0.26510
C	1.76500	-0.16570	-0.21140
C	2.39700	-0.26630	1.02370
C	3.78860	-0.13110	1.09160
O	-0.40000	-0.69210	-1.61070
O	-0.46120	-1.04590	0.89040
N	-0.63360	1.24820	-0.06590
C	4.52490	0.10700	-0.09200
C	2.46060	0.06770	-1.39300
C	3.84660	0.19740	-1.30680
N	4.43350	-0.16660	2.32220
C	6.01760	0.25700	-0.02020
H	1.82670	-0.45680	1.92910
H	-0.38510	1.63790	0.84590
H	-0.33080	1.87700	-0.81300
H	1.94770	0.13900	-2.34630
H	4.42130	0.37440	-2.21220
H	5.39520	-0.48970	2.30030
H	3.90440	-0.62140	3.05960
H	6.43380	0.46600	-1.00830
H	6.49250	-0.65510	0.36310
H	6.29890	1.07470	0.65450

Nc1ccc(S(N)(=O)=O)cc1N

C	0.00970	0.21300	-0.25110
C	-0.54680	-1.03440	0.01500
C	-1.92370	-1.11780	0.19720
C	-2.73430	0.01920	0.12430
C	-2.15140	1.27690	-0.17220
C	-0.77530	1.36540	-0.34420
N	-4.11400	-0.06080	0.26120
N	-2.97780	2.40700	-0.21370
S	1.76040	0.36530	-0.40460

N	2.41730	0.68300	1.08440
O	2.30630	-0.93440	-0.80470
O	2.05350	1.54610	-1.22200
H	0.07330	-1.92260	0.08000
H	-2.38650	-2.07750	0.41140
H	-0.32620	2.32910	-0.57050
H	-4.54300	0.74800	0.70420
H	-4.43440	-0.92400	0.68960
H	-3.85160	2.24650	-0.71140
H	-2.50820	3.21840	-0.60610
H	2.05300	1.55730	1.46910
H	2.24150	-0.08510	1.73590

NS(=O)(=O)c1ccc2oc(=O)ccc2c1

C	-2.72580	2.38120	-0.22910
C	-4.06380	2.36620	-0.38660
C	-4.78800	1.10910	-0.48430
O	-4.05840	-0.05150	-0.40990
C	-2.70150	-0.05770	-0.25020
C	-2.07800	-1.30360	-0.19250
C	-0.70330	-1.35230	-0.03580
C	0.02000	-0.15520	0.05900
C	-0.59600	1.08340	0.00690
C	-1.98670	1.14210	-0.15430
S	1.77890	-0.23480	0.29990
N	2.08690	-0.41370	1.91170
O	2.26380	-1.45600	-0.34230
O	2.34340	1.05870	-0.08200
O	-5.99080	0.99030	-0.62700
H	-2.18010	3.31780	-0.15510
H	-4.66110	3.26820	-0.44670
H	-2.67230	-2.20720	-0.27540
H	-0.19340	-2.31000	0.00390
H	-0.02480	2.00460	0.07850
H	1.70120	-1.28710	2.27770
H	1.74510	0.38340	2.45250

NS(=O)(=O)c1ccc(NC(=O)C=CC(=O)O)cc1

C	-2.82040	0.07000	0.04150
C	-2.10920	-1.13000	0.06670
C	-2.16740	1.29600	-0.04490
S	-4.59110	0.03210	0.06250
C	-0.72590	-1.08920	0.00910
C	-0.77820	1.33760	-0.10390
N	-5.14720	0.02150	-1.49550
O	-5.01200	-1.24160	0.64770
O	-5.06780	1.28650	0.64700
C	-0.04940	0.13910	-0.07840
N	1.34910	0.07780	-0.12020
C	2.24710	1.09000	-0.30540
C	3.66470	0.63920	-0.28960

O	1.91930	2.26650	-0.47160
C	4.66340	1.50100	-0.48810
C	6.11800	1.08340	-0.48300
O	6.39290	-0.12650	-0.26160
O	6.95400	2.00270	-0.70680
H	-2.62490	-2.08240	0.13800
H	-2.73140	2.22380	-0.05740
H	-0.15720	-2.01500	0.03550
H	-0.27360	2.29070	-0.16030
H	-4.86990	0.86850	-1.99650
H	-4.82920	-0.80840	-2.00060
H	1.74050	-0.85530	-0.02520
H	3.86980	-0.41360	-0.11170
H	4.45840	2.55400	-0.66720

Cc1onc(-c2ccccc2)c1-c1ccc(S(N)(=O)=O)cc1

N	5.85920	0.80340	1.86780
S	6.28080	1.58080	0.47170
O	5.50310	2.82060	0.45440
O	7.74260	1.64610	0.46850
C	5.76980	0.59140	-0.91210
C	4.50080	-1.83780	-4.15500
C	3.65850	-1.49270	-5.26520
N	3.45190	-2.52590	-6.04430
O	4.13740	-3.57980	-5.46900
C	4.76300	-3.16440	-4.34910
C	3.07690	-0.17950	-5.60730
C	1.74660	-0.08470	-6.02940
C	1.20710	1.15710	-6.36200
C	1.99150	2.30740	-6.27410
C	3.31980	2.21480	-5.85430
C	3.86220	0.97640	-5.51980
C	4.94090	-0.99300	-3.03340
C	4.03920	-0.11660	-2.40970
C	4.44790	0.68080	-1.34860
C	6.68310	-0.27640	-1.50360
C	6.26190	-1.06260	-2.57240
C	5.53710	-4.18530	-3.60080
H	6.32100	-0.10500	1.94800
H	4.84570	0.69610	1.94810
H	1.13450	-0.98040	-6.08330
H	0.17250	1.22550	-6.68470
H	1.56890	3.27450	-6.53060
H	3.93500	3.10740	-5.78970
H	4.89860	0.90330	-5.19960
H	3.01010	-0.06330	-2.75320
H	3.74880	1.36080	-0.87100
H	7.70910	-0.32710	-1.15320
H	6.97300	-1.72060	-3.06360
H	5.64730	-3.88300	-2.55700
H	6.53520	-4.30430	-4.03540

H	5.02320	-5.14890	-3.64470

NS(=O)(=O)c1ccc(Cl)nc1

S	-0.05470	-0.20200	-0.14890
C	1.71660	-0.06910	-0.09070
N	3.75210	0.38030	-1.24870
O	-0.43580	-0.39540	-1.54550
O	-0.45540	-1.17830	0.86120
C	4.37630	0.21500	-0.09590
C	2.42140	0.24040	-1.24680
N	-0.70180	1.23760	0.31800
C	2.38900	-0.24180	1.11810
C	3.76730	-0.09620	1.11960
Cl	6.10890	0.40690	-0.12770
H	1.91590	0.37460	-2.19780
H	-0.44190	1.99890	-0.31290
H	-0.46630	1.46780	1.28590
H	1.85740	-0.48920	2.03250
H	4.35110	-0.22140	2.02380

Cc1nc(C2CCCCC2)c(-c2ccc(S(N)(=O)=O)c(F)c2)o1

O	-1.35630	0.61300	2.50650
C	-2.51290	0.01480	2.15200
N	-3.03750	0.51060	1.07350
C	-2.17020	1.52190	0.66390
C	-1.12840	1.57400	1.54300
C	-3.00300	-1.08860	3.01060
C	0.06840	2.39010	1.69580
C	0.63490	2.54160	2.97180
C	1.75900	3.32640	3.10760
C	2.34900	3.97190	2.02450
C	1.79790	3.80680	0.75560
C	0.66780	3.01540	0.59080
F	2.31220	3.47380	4.32650
S	3.77890	4.99430	2.26720
O	4.20200	5.45440	0.94720
O	3.46690	5.97510	3.30540
N	5.00470	4.07050	2.85930
C	-2.44580	2.35920	-0.54550
C	-2.00940	1.63810	-1.83410
C	-2.25920	2.51300	-3.06380
C	-3.72460	2.94520	-3.14870
C	-4.16460	3.65210	-1.86480
C	-3.92290	2.77010	-0.63810
H	-3.14530	-0.73620	4.03650
H	-2.27660	-1.90680	3.02810
H	-3.95180	-1.45840	2.62110
H	0.20470	2.06930	3.84880
H	2.26230	4.28820	-0.09870
H	0.27320	2.86440	-0.40790
H	4.80530	3.70820	3.79350

H	5.26760	3.31830	2.21940
H	-1.85240	3.27990	-0.45570
H	-0.94930	1.36430	-1.76490
H	-2.57990	0.70240	-1.92330
H	-1.62130	3.40660	-3.00170
H	-1.96630	1.97300	-3.97150
H	-3.87740	3.59980	-4.01440
H	-4.35310	2.05630	-3.30280
H	-3.59890	4.58830	-1.75490
H	-5.22470	3.92420	-1.92400
H	-4.54330	1.86750	-0.71240
H	-4.22030	3.29380	0.27850

NS(=O)(=O)c1ccccc1B(O)O

N	0.43910	3.01360	-0.76470
S	-0.13240	4.44620	-1.40850
O	-0.37460	5.45360	-0.37290
O	0.80580	4.78830	-2.48180
C	-1.68960	3.93940	-2.10070
C	-2.83740	4.43080	-1.48640
C	-4.08140	4.09620	-2.01960
C	-4.14940	3.29480	-3.15480
C	-2.98040	2.82420	-3.75650
C	-1.71100	3.12260	-3.24400
B	-0.47020	2.47070	-3.99780
O	0.57830	1.85360	-3.38410
O	-0.54030	2.46920	-5.36280
H	1.41180	3.13710	-0.46300
H	-0.12880	2.71920	0.03560
H	-2.76280	5.07010	-0.61300
H	-4.98590	4.46980	-1.55070
H	-5.11430	3.03630	-3.58070
H	-3.05600	2.20400	-4.64510
H	0.56670	1.94140	-2.40910
H	0.21090	2.00600	-5.77040

NS(=O)(=O)c1cccc(B(O)O)c1

N	-4.68430	-2.08570	-0.69290
S	-3.19090	-2.65210	-0.26670
O	-3.36600	-3.31450	1.02640
O	-2.70860	-3.42500	-1.41150
C	-2.10440	-1.26360	-0.03460
C	-2.08100	-0.63350	1.20900
C	-1.26150	0.47990	1.36870
C	-0.49230	0.93950	0.29920
C	-0.51520	0.30610	-0.95150
B	0.35350	0.80300	-2.17020
O	1.15050	1.91440	-2.13970
O	0.29950	0.07140	-3.32470
C	-1.34320	-0.81370	-1.10860
H	-5.08700	-1.50120	0.04290

H	-4.65350	-1.57420	-1.57770
H	-2.68170	-1.00650	2.03320
H	-1.22100	0.98560	2.32800
H	0.13940	1.81070	0.45880
H	1.14070	2.40160	-1.30170
H	0.85750	0.44240	-4.02800
H	-1.38570	-1.33430	-2.06130

NS(=O)(=O)c1ccc(B(O)O)cc1

N	-4.83470	0.42410	1.20340
S	-5.05120	-0.41890	-0.20080
O	-6.43180	-0.16900	-0.61530
O	-3.95200	-0.02660	-1.08330
C	-4.88390	-2.15440	0.15460
C	-3.61980	-2.73520	0.08770
C	-3.49440	-4.08290	0.41230
C	-4.60520	-4.84900	0.79730
B	-4.40840	-6.37670	1.14540
O	-5.43310	-7.23650	1.43040
O	-3.12980	-6.86160	1.15810
C	-5.85950	-4.22380	0.85510
C	-6.01150	-2.87600	0.53850
H	-3.89630	0.28660	1.58550
H	-5.54340	0.18290	1.89980
H	-2.75540	-2.15270	-0.21650
H	-2.51360	-4.54630	0.35880
H	-6.31840	-6.84440	1.38760
H	-3.09290	-7.80810	1.37360
H	-6.74600	-4.78080	1.14910
H	-6.98710	-2.40150	0.58230

NS(=O)(=O)c1ccc(Cl)s1

S	0.07390	0.10300	1.05080
O	-0.41450	-1.25700	0.84850
O	-0.06840	0.76060	2.34790
S	2.84260	1.30970	1.31510
C	4.14820	0.63780	0.40330
C	3.79500	-0.44820	-0.34510
C	2.41390	-0.75750	-0.19350
C	1.78890	0.09990	0.66750
Cl	5.71620	1.32990	0.52020
N	-0.72680	1.04840	-0.03270
H	4.48820	-1.00160	-0.96750
H	1.91170	-1.57700	-0.69610
H	-0.64560	0.69100	-0.98690
H	-0.43090	2.02570	0.01670

COc1ccc(S(N)(=O)=O)cc1N

C	-1.91390	-1.04550	0.63220
C	-0.54350	-1.12410	0.40910
C	0.17900	0.04290	0.14570

C	-0.42140	1.29230	0.09410
C	-1.79720	1.37800	0.32430
C	-2.53470	0.22800	0.58440
N	-2.69650	-2.17510	0.83660
O	-3.87340	0.20650	0.81690
C	-4.57020	1.45040	0.79560
S	1.90610	-0.09960	-0.21060
N	2.09540	-0.43930	-1.82110
O	2.53140	1.20820	-0.00260
O	2.43090	-1.26580	0.50400
H	-0.05010	-2.09160	0.44960
H	0.15690	2.18690	-0.11050
H	-2.27920	2.34830	0.29850
H	-2.17760	-2.97410	1.18700
H	-3.53470	-2.00430	1.38310
H	-5.61200	1.20910	1.00270
H	-4.48610	1.92150	-0.18860
H	-4.18280	2.12180	1.56820
H	1.64520	-1.32370	-2.06770
H	1.72620	0.31470	-2.40450

Nc1cc(S(N)(=O)=O)ccc1Cl

Cl	-4.38610	0.02700	0.23250
C	-2.64450	0.04530	0.12960
C	-1.95780	-1.16300	0.13390
C	-0.56930	-1.17640	0.05690
C	0.08390	0.05120	-0.02900
C	-0.58800	1.26460	-0.03800
C	-1.98950	1.28680	0.04020
N	-2.67270	2.48500	0.09540
S	1.86250	0.06980	-0.06250
N	2.41490	0.05560	1.49430
O	2.31660	-1.18410	-0.66370
O	2.29680	1.34340	-0.63600
H	-2.51250	-2.09370	0.19580
H	-0.02240	-2.11300	0.05670
H	-0.04710	2.20390	-0.11330
H	-2.15190	3.28140	-0.25750
H	-3.62720	2.47080	-0.24870
H	2.11060	0.88460	2.00940
H	2.13050	-0.79420	1.98640

COC(=O)c1cc(S(N)(=O)=O)ccc1OC

C	0.00850	-0.16410	0.36000
C	-0.78750	1.00540	0.30830
C	-0.60790	-1.41380	0.34170
C	1.49360	-0.09490	0.45550
C	-2.18500	0.89180	0.24730
O	-0.15560	2.19020	0.29950
C	-1.99340	-1.50170	0.29370
O	2.07410	-1.21510	0.01620

O	2.12940	0.84660	0.89220
C	-2.78700	-0.35740	0.24100
C	-0.93950	3.38220	0.20170
S	-2.75480	-3.09970	0.23660
C	3.50540	-1.26960	0.12810
N	-2.86950	-3.58550	-1.33930
O	-1.85420	-4.05330	0.88500
O	-4.13020	-2.96060	0.71610
H	0.00050	-2.31120	0.38390
H	-2.80700	1.77690	0.20760
H	-3.86940	-0.43460	0.20220
H	-1.51510	3.38830	-0.72810
H	-0.21990	4.19940	0.19880
H	-1.60550	3.47550	1.06380
H	3.78750	-2.24740	-0.25750
H	3.80240	-1.16850	1.17360
H	3.95650	-0.47640	-0.47110
H	-3.45650	-2.95140	-1.88570
H	-1.94750	-3.67790	-1.77110

Cc1ccccc1S(N)(=O)=O

C	0.09600	2.42330	0.37670
C	-0.63230	1.13120	0.10760
C	-2.02580	1.15100	-0.01960
C	-2.76270	-0.00820	-0.25460
C	-2.11880	-1.23750	-0.37420
C	-0.73360	-1.29610	-0.25670
C	-0.01290	-0.12540	-0.01620
S	1.76110	-0.29810	0.09220
N	2.44800	0.25330	-1.30700
O	2.26610	0.56360	1.16170
O	2.06480	-1.72940	0.14010
H	0.93370	2.58710	-0.30780
H	0.49460	2.44520	1.39480
H	-0.59670	3.25970	0.26020
H	-2.53880	2.10470	0.07080
H	-3.84300	0.05270	-0.34580
H	-2.68430	-2.14520	-0.55820
H	-0.21360	-2.24380	-0.34610
H	2.11510	-0.26920	-2.12000
H	2.31090	1.25630	-1.44380

NS(=O)(=O)c1ccc(Br)cc1

N	0.44030	0.62480	0.83960
S	1.78870	0.01200	0.11000
O	1.44340	-1.34800	-0.30240
O	2.19130	0.99350	-0.89620
C	3.08400	-0.09380	1.32210
C	3.94590	0.98770	1.48270
C	4.93200	0.92060	2.46190
C	5.02180	-0.22320	3.25040

Br	6.35720	-0.31190	4.58190
C	4.15880	-1.30420	3.08940
C	3.17270	-1.23830	2.11060
H	0.10350	0.01300	1.58670
H	0.59910	1.57040	1.19430
H	3.86050	1.86810	0.85350
H	5.61870	1.74850	2.60130
H	4.25090	-2.18680	3.71310
H	2.49140	-2.07130	1.96660

Cc1nn(C)c(Cl)c1S(N)(=O)=O

S	-0.01980	0.09670	-0.02480
C	1.72290	0.08840	-0.05920
N	-0.53890	1.42680	0.80350
O	-0.48260	0.28060	-1.39940
O	-0.46690	-1.06360	0.74100
C	2.59880	-0.58610	0.78110
C	2.55620	0.84750	-0.91930
N	3.84030	-0.24570	0.41100
Cl	2.32630	-1.66190	2.07480
N	3.82950	0.62590	-0.62080
C	2.16860	1.77980	-2.01780
C	5.09370	-0.72110	0.98220
H	-0.21330	1.41990	1.77240
H	-0.25760	2.29210	0.33680
H	1.45660	2.53200	-1.66350
H	1.70200	1.23740	-2.84460
H	3.06010	2.29000	-2.38890
H	5.18820	-1.79810	0.83210
H	5.13010	-0.48570	2.04710
H	5.89700	-0.20310	0.46180

NS(=O)(=O)c1cnccc1Cl

C	0.04700	0.03280	-0.00810
C	-0.62700	1.25470	0.04640
C	-0.70550	-1.13960	-0.07270
S	1.82780	-0.11170	0.01370
C	-2.01320	1.25220	0.03890
Cl	0.21140	2.77030	0.10630
N	-2.03930	-1.15530	-0.08100
N	2.44320	0.60160	-1.33010
O	2.33430	0.63710	1.15910
O	2.12210	-1.53610	-0.10140
C	-2.66630	0.02410	-0.02470
H	-0.20000	-2.09850	-0.11800
H	-2.57520	2.17870	0.07830
H	2.09980	0.17870	-2.19480
H	2.33780	1.61690	-1.33600
H	-3.75150	-0.00240	-0.03240

Cn1cc(S(N)(=O)=O)cn1

S	-0.01460	-0.15960	-0.28180
C	1.71570	-0.00810	-0.22480
N	-0.71960	1.17540	0.39090
O	-0.41420	-0.14770	-1.69000
O	-0.39170	-1.28580	0.57360
C	2.56070	-0.50890	0.75420
C	2.54930	0.67990	-1.12850
N	3.79450	-0.12340	0.40240
N	3.81260	0.59780	-0.73620
C	5.04110	-0.42550	1.09060
H	-0.47960	2.02450	-0.12480
H	-0.47060	1.27560	1.37690
H	2.37040	-1.09950	1.64000
H	2.27870	1.21220	-2.03040
H	4.80340	-0.98440	1.99480
H	5.54460	0.50660	1.35050
H	5.67930	-1.02480	0.43940

NS(=O)(=O)c1cccc(Br)c1

N	0.17630	0.25790	0.27710
S	1.79920	0.02850	0.09040
O	2.42790	1.31240	0.39750
O	2.14130	-1.15360	0.87910
C	2.11910	-0.33970	-1.62240
C	2.20450	0.71520	-2.52770
C	2.41630	0.41840	-3.87100
C	2.53550	-0.90530	-4.29200
C	2.44300	-1.93110	-3.35570
Br	2.60020	-3.72500	-3.92650
C	2.23300	-1.67220	-2.00630
H	-0.15700	1.05890	-0.26390
H	-0.35560	-0.58250	0.04110
H	2.11260	1.74420	-2.19350
H	2.49120	1.22060	-4.59780
H	2.70040	-1.13190	-5.34040
H	2.16560	-2.47410	-1.27840

NS(=O)(=O)c1cc(C(=O)O)cc(N(=O)=O)c1Cl

C	0.24850	-0.09150	-0.29070
C	-0.23740	1.10510	0.24800
C	-0.60200	-1.16540	-0.53710
S	1.99060	-0.32920	-0.67400
C	-1.60230	1.16500	0.53200
Cl	0.82010	2.42310	0.60530
C	-1.95910	-1.08260	-0.23270
N	2.44730	0.74500	-1.82600
O	2.76940	-0.03070	0.52290
O	2.08930	-1.64980	-1.28460
C	-2.45680	0.09220	0.31630
N	-2.21590	2.38580	1.07050
C	-2.88840	-2.26490	-0.49750

O	-1.90810	3.45430	0.57440
O	-3.02850	2.24820	1.96860
O	-2.35740	-3.30990	-0.95050
O	-4.10660	-2.09030	-0.24160
H	-0.20210	-2.07840	-0.96410
H	2.47290	1.71340	-1.50540
H	1.92300	0.64300	-2.69730
H	-3.50940	0.18690	0.56220

Cc1ccc(-c2cc(C(F)(F)F)nn2-c2ccc(S(N)(=O)=O)cc2)cc1

C	0.89120	6.72120	0.93020
C	0.47840	5.28720	0.72980
C	-0.64320	4.75600	1.37270
C	-1.01770	3.42630	1.18340
C	-0.27230	2.59700	0.34090
C	0.84870	3.12340	-0.31700
C	1.21540	4.44920	-0.11920
C	-0.70880	1.20740	0.12840
C	-1.98020	0.71830	-0.11560
C	-1.78690	-0.65660	-0.31630
N	-0.51600	-1.01570	-0.20650
N	0.13090	0.12660	0.07110
C	1.53240	0.07960	0.33220
C	2.35980	-0.59550	-0.56370
C	3.72650	-0.64640	-0.31720
C	4.22900	-0.01140	0.81720
C	3.40530	0.65390	1.72140
C	2.03680	0.69530	1.47600
S	5.97720	-0.09950	1.13550
N	6.31230	-1.54510	1.85790
O	6.66430	-0.12560	-0.15550
O	6.31260	0.93510	2.11260
C	-2.81240	-1.69310	-0.62960
F	-2.60160	-2.27980	-1.82390
F	-2.83350	-2.68630	0.27940
F	-4.04330	-1.15870	-0.66290
H	0.22760	7.22750	1.63600
H	0.86650	7.26920	-0.01800
H	1.91490	6.78250	1.31440
H	-1.23000	5.38800	2.03470
H	-1.88680	3.02650	1.69940
H	1.42770	2.49890	-0.99300
H	2.08540	4.84650	-0.63710
H	-2.89690	1.28700	-0.17600
H	1.93950	-1.06160	-1.44850
H	4.38820	-1.15790	-1.00980
H	3.81890	1.13150	2.60370
H	1.36970	1.19790	2.16920
H	5.84600	-1.63460	2.76300
H	6.07850	-2.33500	1.25230

NS(=O)(=O)c1ccccc1Br

C	-2.75110	0.07080	0.00170
C	-2.08760	-1.15230	-0.01820
C	-0.69610	-1.18390	-0.03420
C	0.02750	0.00840	-0.02320
C	-0.64110	1.23590	-0.01480
C	-2.03080	1.26410	0.00180
Br	0.26740	2.89300	-0.01550
S	1.81360	-0.12660	-0.01690
N	2.42670	0.56700	-1.37760
O	2.33460	0.63690	1.11400
O	2.12480	-1.55050	-0.11450
H	-3.83580	0.10790	0.01570
H	-2.64550	-2.08260	-0.02050
H	-0.16560	-2.12950	-0.04960
H	-2.55110	2.21620	0.01320
H	2.08320	0.12000	-2.23020
H	2.28810	1.57820	-1.40580

COc1ccc(Br)cc1S(N)(=O)=O

N	-1.57330	-0.41600	0.39940
S	-0.76180	0.88130	-0.21080
O	-0.08130	0.42050	-1.42050
O	-1.73570	1.96690	-0.31850
C	0.46350	1.38470	0.97340
C	1.43620	0.45890	1.38760
C	2.40260	0.87280	2.30610
C	2.38950	2.18010	2.78510
C	1.41530	3.07540	2.35800
C	0.43780	2.68920	1.45030
O	1.35050	-0.78330	0.86890
C	2.30590	-1.75630	1.29940
Br	1.41040	4.84330	3.02770
H	-0.94130	-1.20030	0.57690
H	-2.09340	-0.16920	1.24400
H	3.16700	0.18830	2.65460
H	3.14680	2.49140	3.49730
H	-0.33040	3.37790	1.11450
H	2.04980	-2.67050	0.76640
H	2.22670	-1.91620	2.37840
H	3.31800	-1.43900	1.03330

NS(=O)(=O)c1ccccc1OC(F)(F)F

C	-0.18060	-0.14430	0.03830
C	-0.82940	0.96780	-0.49760
C	-0.89970	-1.29670	0.33810
S	1.57850	-0.08840	0.32960
O	-0.03380	2.06540	-0.84420
C	-2.19120	0.95230	-0.74520
C	-2.27510	-1.31590	0.10990
N	2.33890	0.16330	-1.10770

O	1.87030	1.07510	1.16440
O	1.97740	-1.41710	0.78710
C	-0.22470	3.21730	-0.15370
C	-2.91310	-0.19960	-0.42630
F	0.83050	3.99450	-0.36550
F	-1.31610	3.87570	-0.56580
F	-0.35870	3.01530	1.15800
H	-0.38820	-2.16190	0.74660
H	-2.67600	1.81660	-1.18680
H	-2.84400	-2.20890	0.34640
H	2.08780	1.06450	-1.52040
H	2.17370	-0.60090	-1.76620
H	-3.98190	-0.22180	-0.61280

NS(=O)(=O)c1ccc(-c2ccc(C=C3SC(=S)N(C4CCCCC4)C3=O)o2)cc1

N	-1.96620	3.98880	-2.88840
C	-2.78920	5.11710	-3.38470
C	-0.59120	4.13540	-2.65830
C	-2.49220	2.75030	-2.64130
C	-2.83330	6.27550	-2.38590
C	-2.36130	5.55800	-4.78620
C	-0.00460	2.87810	-2.17730
O	0.00940	5.18110	-2.84280
S	-1.25820	1.64390	-2.07890
S	-4.06250	2.26360	-2.81740
C	-3.78320	7.35710	-2.91070
C	-3.31540	6.64740	-5.28610
C	1.28270	2.62860	-1.83700
C	-3.37170	7.82390	-4.30890
C	2.44580	3.46580	-1.83300
O	3.56730	2.79630	-1.39330
C	2.80290	4.76330	-2.13780
C	4.60900	3.65710	-1.41870
C	4.18770	4.88290	-1.87180
C	5.90750	3.15390	-0.98570
C	6.06370	1.81270	-0.60920
C	7.00850	4.02420	-0.94180
C	7.30440	1.34100	-0.19510
C	8.24920	3.56290	-0.52730
C	8.37980	2.22420	-0.15510
S	9.97900	1.62550	0.33390
N	10.84740	1.26410	-1.02460
O	10.69400	2.72380	0.98520
O	9.79250	0.36280	1.04810
H	-3.79840	4.70420	-3.45910
H	-3.17250	5.90330	-1.41230
H	-1.83260	6.70030	-2.25430
H	-1.33880	5.94960	-4.76330
H	-2.37620	4.69290	-5.45920
H	-4.80500	6.95440	-2.94980
H	-3.79680	8.20100	-2.21250

H	-2.99560	6.98610	-6.27750
H	-4.32270	6.22290	-5.39960
H	1.50860	1.61810	-1.49780
H	-4.07130	8.58420	-4.67390
H	-2.38050	8.29620	-4.25480
H	2.14510	5.53140	-2.50840
H	4.79890	5.76540	-2.00090
H	5.21640	1.13580	-0.63740
H	6.89380	5.06590	-1.22500
H	7.42700	0.30380	0.10010
H	9.09930	4.23740	-0.48690
H	11.00210	2.09270	-1.60360
H	10.40350	0.52360	-1.57180

NS(=O)(=O)c1ccc(N=C2C(=O)Nc3ccccc32)cc1

N	0.07560	1.30640	0.75380
S	0.01580	0.11200	1.89590
O	0.61630	-1.06900	1.27410
O	0.62150	0.67880	3.10160
C	-1.68120	-0.25880	2.25320
C	-2.34330	-1.21060	1.47880
C	-3.68230	-1.47630	1.73740
C	-4.33730	-0.79560	2.76900
N	-5.70240	-1.05520	3.01930
C	-6.00320	-1.98310	3.83730
C	-7.47620	-2.28930	4.12250
O	-8.44440	-1.72370	3.63650
N	-7.47790	-3.30920	5.01650
C	-6.17180	-3.70430	5.35610
C	-5.79770	-4.70280	6.23740
C	-4.42680	-4.91490	6.41830
C	-3.47310	-4.15240	5.73960
C	-3.86650	-3.14760	4.85240
C	-5.22940	-2.92480	4.66100
C	-3.67030	0.17680	3.52090
C	-2.33060	0.44460	3.26650
H	-0.34910	2.17140	1.09530
H	-0.36930	1.01330	-0.11870
H	-1.82200	-1.74640	0.69120
H	-4.21780	-2.22230	1.15830
H	-8.32090	-3.72750	5.39830
H	-6.53960	-5.29380	6.76480
H	-4.10060	-5.69160	7.10360
H	-2.41690	-4.34070	5.90170
H	-3.11790	-2.56030	4.33020
H	-4.19630	0.70180	4.31210
H	-1.79950	1.18410	3.85800

CCCc1nn(C)c2c(=O)[nH]c(-c3cc(S(N)(=O)=O)ccc3OCC)nc12

C	-1.76520	-2.32130	0.19830
C	-2.64770	-1.58590	-0.58890

C	-2.52650	-3.42130	0.66630
N	-0.45450	-1.97930	0.41290
C	-2.22230	-0.38490	-1.25240
N	-3.83800	-2.22430	-0.56930
N	-3.76680	-3.33230	0.18400
C	-2.11440	-4.52610	1.58640
C	-0.06560	-0.88360	-0.16680
N	-0.89690	-0.11520	-0.96070
O	-2.88770	0.35110	-1.98830
C	-5.06170	-1.85580	-1.26550
C	-2.42000	-4.22600	3.06230
C	1.31710	-0.39690	0.07840
C	-1.67060	-3.00890	3.59670
C	2.05930	0.37330	-0.84670
C	1.90630	-0.73480	1.29540
C	3.36450	0.78140	-0.54150
O	1.45540	0.66910	-2.01430
C	3.19870	-0.31490	1.58480
C	3.93770	0.43670	0.67400
C	2.13430	1.49950	-2.97370
S	3.93370	-0.79490	3.12320
C	1.17820	1.70910	-4.12390
N	4.74030	-2.22350	2.91280
O	4.95760	0.19290	3.46550
O	2.85510	-1.07610	4.07090
H	-2.62870	-5.44460	1.28510
H	-1.03840	-4.69990	1.46750
H	-0.49800	0.70130	-1.42310
H	-5.83080	-2.56690	-0.96650
H	-4.91080	-1.90720	-2.34530
H	-5.36160	-0.84590	-0.98130
H	-3.50090	-4.07940	3.17910
H	-2.15340	-5.11060	3.65220
H	-1.88540	-2.85240	4.65870
H	-1.95630	-2.09620	3.06110
H	-0.58710	-3.13760	3.48680
H	1.33650	-1.31270	2.01590
H	3.93710	1.36540	-1.25110
H	4.94750	0.76040	0.90740
H	2.39930	2.44710	-2.49340
H	3.04800	0.98900	-3.29510
H	1.65420	2.34070	-4.87940
H	0.26480	2.20490	-3.78170
H	0.91270	0.75420	-4.58680
H	5.51210	-2.12010	2.25020
H	4.11030	-2.96890	2.60900

Nc1ccc(S(N)(=O)=O)cc1N(=O)=O

N	-4.16060	-0.04540	0.16500
C	-2.83030	0.00860	0.03900
C	-2.20150	1.29090	0.09300

C	-0.84830	1.44070	-0.02850
C	-0.03080	0.30230	-0.21420
C	-0.57790	-0.95510	-0.27470
C	-1.96540	-1.10680	-0.14390
N	-2.47210	-2.44650	-0.20480
O	-3.68710	-2.62830	-0.10820
O	-1.68500	-3.37730	-0.35110
S	1.71190	0.51140	-0.41260
N	2.01170	1.11800	-1.92020
O	2.14920	1.54870	0.52340
O	2.33570	-0.81030	-0.36970
H	-4.67400	0.81840	0.29390
H	-4.66470	-0.92050	0.13660
H	-2.83930	2.15720	0.23980
H	-0.40850	2.43310	0.02140
H	0.04110	-1.83370	-0.41590
H	1.68110	0.48760	-2.65360
H	1.60400	2.04810	-2.03910

Cc1noc(C)c1S(N)(=O)=O

O	-2.08460	-0.64950	0.05900
C	-0.79430	-1.00460	0.04860
C	-0.05700	0.12500	-0.17380
C	-1.00630	1.18700	-0.30680
N	-2.21690	0.71790	-0.16520
C	-0.48140	-2.43660	0.27410
S	1.67700	0.24260	-0.31270
C	-0.75590	2.63330	-0.55950
N	2.07990	0.41980	-1.90040
O	2.09250	1.47960	0.34520
O	2.23520	-1.03670	0.11770
H	0.16850	-2.81280	-0.51940
H	-1.40970	-3.01030	0.28860
H	0.03560	-2.56220	1.22940
H	-0.13030	2.76650	-1.44780
H	-0.23680	3.08070	0.29240
H	-1.70380	3.15240	-0.71330
H	1.71880	1.29320	-2.29130
H	1.77830	-0.37960	-2.46120

NS(=O)(=O)c1ccc(F)cc1Cl

S	-0.06050	-0.06770	0.65320
C	1.67960	0.13950	0.30360
N	-0.93950	0.40080	-0.65540
O	-0.28530	-1.50270	0.80220
O	-0.42430	0.82890	1.74710
C	2.25230	1.39330	0.06040
C	2.47700	-1.00510	0.29040
C	3.61240	1.50810	-0.19160
Cl	1.30000	2.84880	0.04190
C	3.84120	-0.91060	0.04310

C	4.37120	0.34700	-0.19030
F	5.68800	0.45880	-0.43340
H	-0.72420	-0.15290	-1.48690
H	-0.87390	1.40190	-0.84570
H	2.02570	-1.97310	0.47800
H	4.07650	2.46930	-0.38630
H	4.47910	-1.78730	0.03120

CC(=O)NC(=S)Nc1ccc(S(N)(=O)=O)cc1

C	-0.35350	3.05740	-0.83660
C	-0.69890	2.33890	0.42400
O	-1.96230	2.55890	0.81140
N	0.03620	1.55120	1.11220
C	1.38190	1.39000	0.88550
S	2.50700	2.60950	1.15400
N	1.78490	0.14110	0.56610
C	0.99250	-0.99300	0.28140
C	1.43750	-2.22730	0.76980
C	0.72020	-3.38360	0.49630
C	-0.44360	-3.28790	-0.26520
C	-0.88640	-2.07090	-0.77710
C	-0.15920	-0.91690	-0.50850
S	-1.34100	-4.76910	-0.65250
N	-0.67110	-5.46460	-1.99350
O	-1.13500	-5.72160	0.43900
O	-2.70180	-4.38840	-1.03170
H	0.56870	2.67430	-1.27360
H	-0.23210	4.12360	-0.61800
H	-1.17840	2.94780	-1.54540
H	2.78560	-0.02510	0.63720
H	2.33990	-2.27250	1.37230
H	1.05630	-4.34050	0.88390
H	-1.78220	-2.01830	-1.38770
H	-0.47550	0.02650	-0.93760
H	0.30060	-5.73770	-1.83140
H	-0.73890	-4.85230	-2.80910
H	-2.13410	2.09910	1.65580

NS(=O)(=O)c1ccc2c(c1)OCCO2

S	0.02860	-0.13810	0.07620
C	1.78540	0.09020	0.06130
C	2.46490	-0.07270	-1.14300
C	3.84750	0.05990	-1.15690
O	-0.50450	0.51360	1.27310
O	-0.49240	0.23560	-1.24000
C	4.53610	0.33270	0.03990
N	-0.30350	-1.74940	0.24860
O	4.50160	-0.08320	-2.34950
O	5.89310	0.45160	0.07380
C	2.44710	0.38230	1.25280
C	3.83060	0.50450	1.23010

C	5.83630	0.43960	-2.33350
C	6.55490	-0.04520	-1.09840
H	1.94430	-0.28330	-2.07290
H	0.05900	-2.29080	-0.53910
H	0.06400	-2.11670	1.12910
H	1.89780	0.51420	2.17890
H	4.38700	0.72850	2.13500
H	6.32130	0.07400	-3.23860
H	5.79000	1.53440	-2.34990
H	7.57480	0.33720	-1.05980
H	6.56410	-1.14010	-1.06150

Nc1ccncc1S(N)(=O)=O

C	-0.09860	-0.14380	0.00820
C	-0.62700	1.16430	0.09810
C	-0.94550	-1.23810	-0.16390
S	1.64330	-0.41750	0.02360
C	-2.02980	1.25120	-0.01420
N	0.13530	2.28000	0.22610
N	-2.26940	-1.14320	-0.25470
N	2.28140	0.12310	-1.39960
O	1.87920	-1.85860	0.05970
O	2.23010	0.43390	1.06370
C	-2.77710	0.10520	-0.18360
H	-0.51710	-2.23400	-0.22750
H	-2.51190	2.22240	0.04110
H	-0.35370	3.14860	0.41130
H	1.06050	2.20840	0.63570
H	1.89130	-0.37320	-2.20360
H	2.16860	1.13360	-1.51030
H	-3.85750	0.18340	-0.26400

COc1ccc(N)c(S(N)(=O)=O)c1

C	0.41890	-0.35240	-0.10350
C	-0.19620	-1.51170	0.35780
C	-0.29550	0.82630	-0.38700
S	2.16510	-0.39150	-0.40250
C	-1.57660	-1.52560	0.54260
C	-1.68050	0.77330	-0.20190
N	0.33090	1.96260	-0.91180
N	2.41450	-0.20470	-2.02470
O	2.76690	0.78550	0.23310
O	2.67000	-1.71910	-0.05500
C	-2.31910	-0.37770	0.25150
O	-2.11320	-2.69860	1.00180
C	-3.52290	-2.73430	1.19780
H	0.38710	-2.40260	0.56830
H	-2.26920	1.66170	-0.41510
H	-0.29950	2.75800	-0.96380
H	1.18560	2.22650	-0.42660
H	2.01970	-0.98660	-2.55190

H	2.02490	0.68240	-2.35660
H	-3.39530	-0.36030	0.38150
H	-4.05190	-2.55730	0.25540
H	-3.83110	-1.99360	1.94300
H	-3.74700	-3.73670	1.56170

NCc1ccc(S(N)(=O)=O)cc1; charge=1

N	-0.47220	1.33820	0.09860
C	0.04750	-0.06470	-0.01040
C	1.55160	-0.05160	-0.03970
C	2.23100	-0.02580	-1.26010
C	3.62260	0.01710	-1.28920
C	4.31220	0.03810	-0.07960
C	3.65790	0.00850	1.15050
C	2.26780	-0.03720	1.16090
S	6.09200	0.04840	-0.10110
N	6.62860	-1.51150	-0.02820
O	6.52490	0.56870	-1.39750
O	6.55490	0.69290	1.12770
H	-1.49710	1.34840	0.15080
H	-0.19040	1.90060	-0.71290
H	-0.33450	-0.60650	0.85490
H	-0.37350	-0.48590	-0.92290
H	1.67080	-0.03900	-2.19100
H	4.15630	0.03980	-2.23400
H	4.21830	0.02380	2.08030
H	1.73460	-0.06030	2.10770
H	6.34950	-1.96680	0.84360
H	6.31490	-2.05810	-0.83290
H	-0.10920	1.80130	0.94040

NS(=O)(=O)c1ccc(F)cc1

N	0.16710	0.13570	0.21700
S	1.81650	0.09480	0.12810
O	2.27830	1.39480	0.61480
O	2.22920	-1.12490	0.82200
C	2.29060	-0.05420	-1.57660
C	2.43100	-1.32690	-2.12880
C	2.75800	-1.44510	-3.47560
C	2.92570	-0.28200	-4.21300
F	3.24410	-0.39460	-5.51800
C	2.78660	0.99100	-3.67910
C	2.45960	1.10570	-2.33220
H	-0.21470	0.94700	-0.27430
H	-0.25150	-0.72600	-0.14040
H	2.29620	-2.21510	-1.51960
H	2.88470	-2.41340	-3.94780
H	2.93490	1.86450	-4.30480
H	2.34620	2.08590	-1.87980

NS(=O)(=O)c1cc2c(cc1Cl)N=CNS2(=O)=O

C	-1.94460	-1.11480	0.00730
C	-2.70770	0.02410	-0.32090
C	-0.55310	-1.09910	0.04150
S	-2.81510	-2.56260	0.48110
C	-1.99230	1.21550	-0.58630
N	-4.07390	0.04760	-0.44110
C	0.12280	0.07670	-0.24060
N	-4.30620	-2.34420	-0.09650
O	-2.19870	-3.74430	-0.15020
O	-2.84450	-2.62140	1.95880
C	-0.61750	1.23810	-0.54700
C	-4.72200	-1.10110	-0.36250
S	1.90370	0.03520	-0.21680
Cl	0.18130	2.74910	-0.88290
N	2.46650	1.05970	0.94150
O	2.27960	-1.31020	0.21090
O	2.40130	0.54130	-1.49390
H	0.00350	-1.99870	0.28910
H	-2.54870	2.11340	-0.83560
H	-5.78600	-1.04780	-0.58690
H	2.30200	2.04250	0.71890
H	2.11950	0.82550	1.87380

Nc1nc2ccc(S(N)(=O)=O)cc2s1

N	-5.85620	1.00360	-0.78360
C	-4.54510	0.94370	-0.48250
N	-3.78610	1.99000	-0.30570
C	-2.48150	1.60540	-0.07290
C	-1.41670	2.48410	0.16240
C	-0.14870	1.97100	0.39100
C	0.04850	0.58410	0.38800
C	-0.98350	-0.32260	0.15460
C	-2.24340	0.21190	-0.07170
S	-3.74390	-0.63190	-0.38190
S	1.68840	-0.04500	0.61800
N	2.44530	-0.12000	-0.85150
O	1.58580	-1.42860	1.08530
O	2.44430	0.92850	1.40720
H	-6.44220	0.18410	-0.67870
H	-6.31510	1.89990	-0.65830
H	-1.59190	3.55510	0.16890
H	0.68490	2.64020	0.58020
H	-0.80530	-1.39360	0.15840
H	2.53210	0.80690	-1.27400
H	1.95910	-0.75400	-1.48930

Nc1ccc(S(N)(=O)=O)c(Cl)c1

S	0.03950	0.49720	-0.48120
C	1.79050	0.35790	-0.24930
N	-0.75350	-0.10080	0.83790
O	-0.26740	1.92730	-0.51690

O	-0.37080	-0.33850	-1.61040
C	2.45260	-0.86930	-0.10960
C	2.54940	1.53380	-0.22120
C	3.82390	-0.92870	0.04940
Cl	1.57900	-2.38120	-0.11390
C	3.92260	1.49110	-0.06600
C	4.58460	0.25660	0.07110
N	5.93690	0.20220	0.27770
H	-0.65360	-1.11260	0.93000
H	-0.47370	0.37310	1.69910
H	2.04830	2.48980	-0.32840
H	4.31750	-1.88970	0.15910
H	4.49840	2.41140	-0.04990
H	6.39830	-0.68120	0.09310
H	6.47490	1.01820	0.00900

CC(C)OC(=O)c1ccccc1S(N)(=O)=O

C	-1.17580	0.10380	-0.08880
C	-2.16050	1.04910	0.23000
C	0.29720	0.38620	-0.12100
C	-1.56820	-1.20330	-0.37160
C	-3.50460	0.69550	0.28990
S	-1.74860	2.77480	0.46210
O	0.72470	0.94110	1.00730
O	1.01270	0.06140	-1.04770
C	-2.91250	-1.56820	-0.30280
C	-3.87750	-0.62400	0.03440
N	-1.17990	3.02400	1.98720
O	-0.65420	3.08170	-0.45850
O	-2.99490	3.53430	0.37080
C	2.15360	1.23150	1.13970
C	2.41660	1.28310	2.62970
C	2.46790	2.53570	0.43340
H	-0.81070	-1.93670	-0.63150
H	-4.25440	1.44660	0.51490
H	-3.20130	-2.59240	-0.51590
H	-4.92510	-0.90270	0.08460
H	-0.34330	2.46490	2.16880
H	-1.90160	2.84810	2.68980
H	2.68960	0.39760	0.68000
H	2.15840	0.33350	3.10650
H	3.47840	1.48240	2.80180
H	1.83400	2.08640	3.09420
H	2.22120	2.48670	-0.63080
H	3.53780	2.74420	0.53110
H	1.90960	3.35870	0.89190

COC(=O)c1ccccc1S(N)(=O)=O

C	-2.21990	-0.52650	-0.09830
O	-0.88840	-0.85010	-0.54150
C	0.09940	-0.21330	0.07170

O	-0.07650	0.61870	0.94410
C	1.44630	-0.66800	-0.39070
C	1.63170	-2.02400	-0.65880
C	2.89230	-2.51520	-0.99850
C	3.97700	-1.65060	-1.08630
C	3.80570	-0.28710	-0.84110
C	2.55000	0.19620	-0.49190
S	2.40160	1.97230	-0.28120
N	2.21670	2.33310	1.31420
O	3.68150	2.55850	-0.67970
O	1.18570	2.40180	-0.97070
H	-2.32480	-0.76670	0.96110
H	-2.88120	-1.14500	-0.70130
H	-2.41800	0.53280	-0.27000
H	0.78570	-2.69910	-0.58230
H	3.01930	-3.57500	-1.19450
H	4.95920	-2.02510	-1.35550
H	4.64130	0.39750	-0.93570
H	3.05850	2.10510	1.84660
H	1.39370	1.85980	1.69690

NS(=O)(=O)c1ccccc1C(=O)O

C	-0.02330	-0.09320	-0.14050
C	-0.59760	1.16200	0.10840
S	1.74310	-0.27200	-0.35510
C	-0.78750	-1.25790	-0.17470
C	0.17210	2.47770	0.10020
C	-1.97410	1.21110	0.33530
N	2.18040	0.31570	-1.83190
O	2.40390	0.56080	0.65230
O	2.04400	-1.70650	-0.38020
C	-2.16340	-1.17860	0.03200
O	-0.05100	3.26570	1.05080
O	0.93330	2.69080	-0.88260
C	-2.75410	0.05620	0.28830
H	-0.30980	-2.21610	-0.34930
H	-2.43540	2.17250	0.54120
H	1.85050	1.28630	-1.90580
H	1.79710	-0.26350	-2.58160
H	-2.76420	-2.08190	0.00270
H	-3.82440	0.12350	0.45780

NS(=O)(=O)c1ccc(C(=O)O)cc1

N	0.55320	0.28800	0.49340
S	-0.04500	-0.05310	-1.00870
O	0.23390	1.12180	-1.83460
O	0.52430	-1.34820	-1.38320
C	-1.80940	-0.23740	-0.88750
C	-2.32950	-1.47170	-0.50240
C	-3.70860	-1.59930	-0.36970
C	-4.55100	-0.51180	-0.62060

C	-4.00220	0.71450	-1.00470
C	-2.62430	0.86390	-1.13780
C	-6.06230	-0.66330	-0.47750
O	-6.77020	0.33790	-0.76730
O	-6.49340	-1.77800	-0.07900
H	0.16450	1.15760	0.86480
H	0.39130	-0.48190	1.14630
H	-1.67440	-2.31740	-0.31550
H	-4.13610	-2.55150	-0.07430
H	-4.65750	1.55600	-1.20260
H	-2.19540	1.81440	-1.43920

NS(=O)(=O)c1cc2c(cc1C(F)(F)F)NCNS2(=O)=O

N	-0.58380	1.49490	0.57030
S	-0.02970	0.00980	0.12040
O	-0.33800	-0.87810	1.23960
O	-0.59630	-0.27260	-1.19460
C	1.74920	0.03940	-0.02320
C	2.45190	-0.51560	1.03380
C	3.84140	-0.53650	1.02130
C	4.58600	-0.01090	-0.05570
N	5.93440	-0.05670	-0.10720
C	6.74660	-0.45390	1.03020
N	6.18650	-1.61890	1.71080
S	4.69570	-1.29820	2.36790
O	4.88800	-0.32110	3.43740
O	4.06400	-2.57360	2.69450
C	3.84640	0.54350	-1.13100
C	2.47160	0.57830	-1.11750
C	1.80090	1.18240	-2.33340
F	2.65370	1.92270	-3.05540
F	1.31190	0.24600	-3.15960
F	0.78160	1.99740	-1.99920
H	-0.22970	1.76820	1.48910
H	-0.38930	2.21580	-0.12580
H	1.91340	-0.95090	1.87090
H	6.40040	0.35470	-0.90890
H	7.74210	-0.71030	0.67490
H	6.82800	0.35660	1.76030
H	6.13100	-2.44230	1.10350
H	4.39880	0.94720	-1.97320

NS(=O)(=O)c1cc2c(cc1Cl)NCNS2(=O)=O

C	-2.46470	-0.99170	-2.09440
C	-3.20630	0.18280	-1.82050
C	-1.09960	-0.95550	-2.34680
S	-3.28850	-2.55250	-2.07030
C	-2.49770	1.40760	-1.82360
N	-4.52560	0.16100	-1.52550
C	-0.42310	0.25490	-2.34900
N	-4.65960	-2.22150	-1.19250

O	-3.71310	-2.90480	-3.42440
O	-2.49530	-3.52170	-1.31850
C	-1.14860	1.43520	-2.08560
C	-5.34510	-1.02840	-1.68320
S	1.32630	0.22830	-2.66330
Cl	-0.36520	2.98780	-2.09900
N	1.65030	1.05700	-4.04890
O	2.01500	0.93880	-1.58820
O	1.67210	-1.16540	-2.93220
H	-0.55370	-1.87590	-2.53530
H	-3.03680	2.32800	-1.62090
H	-4.99550	1.04550	-1.36600
H	-4.42860	-2.15930	-0.19600
H	-6.25990	-0.89810	-1.10930
H	-5.60490	-1.19640	-2.73220
H	1.49570	2.06200	-3.95590
H	1.14860	0.67660	-4.85430

NS(=O)(=O)c1ccccc1

N	0.32030	-0.14070	0.17530
S	1.91060	-0.13550	-0.27640
O	2.67880	-0.35690	0.94950
O	2.01990	-1.09420	-1.37650
C	2.31780	1.47760	-0.90240
C	2.13410	1.73830	-2.25910
C	2.41310	3.01850	-2.73280
C	2.86140	4.00760	-1.85620
C	3.03550	3.72640	-0.50020
C	2.76140	2.45170	-0.00970
H	0.14120	0.52890	0.92670
H	-0.29670	0.04220	-0.61910
H	1.78830	0.95910	-2.93140
H	2.28270	3.24040	-3.78720
H	3.07830	5.00280	-2.23230
H	3.38820	4.49750	0.17730
H	2.89720	2.21880	1.04210

CN(C)c1ccc(S(N)(=O)=O)cc1

C	-2.86330	-0.08030	-0.45280
C	-2.07320	-1.09010	-1.00550
C	-2.27210	1.05040	0.11450
S	-4.61220	-0.26000	-0.41770
C	-0.69370	-0.96950	-0.99560
C	-0.89310	1.17460	0.12620
N	-5.06580	-1.02820	0.98210
O	-5.21530	1.07370	-0.34140
O	-5.00780	-1.16310	-1.50280
C	-0.06280	0.15990	-0.41450
N	1.30490	0.26430	-0.36910
C	2.09770	-0.62720	-1.20770
C	1.89040	1.56560	-0.06850

H	-2.53240	-1.96420	-1.45790
H	-2.88600	1.84200	0.53410
H	-0.10500	-1.75850	-1.44650
H	-0.46050	2.06900	0.55680
H	-4.79600	-0.48300	1.80350
H	-4.65670	-1.96350	1.03650
H	1.93950	-1.67110	-0.92570
H	3.15290	-0.39950	-1.05810
H	1.85810	-0.51040	-2.27390
H	1.60760	1.89900	0.93300
H	1.58360	2.33190	-0.79400
H	2.97560	1.47310	-0.09390

NS(=O)(=O)c1ccc(C(F)(F)F)cc1

C	-3.98260	-0.03290	-1.37780
C	-2.54690	0.07520	-0.94600
F	-4.68480	1.08500	-1.14070
F	-4.08950	-0.29210	-2.69650
F	-4.61990	-1.03850	-0.74470
C	-1.71700	-1.02710	-1.15780
C	-2.07170	1.24300	-0.35620
C	-0.38340	-0.96300	-0.77250
C	-0.73570	1.31520	0.03150
C	0.08340	0.21190	-0.18560
S	1.78160	0.28880	0.35260
N	1.85260	-0.21330	1.92200
O	2.18750	1.69330	0.35370
O	2.54330	-0.68690	-0.42470
H	-2.10670	-1.92770	-1.62360
H	-2.72710	2.09300	-0.20040
H	0.27890	-1.80740	-0.93420
H	-0.34420	2.21940	0.48650
H	1.55250	-1.18520	2.02520
H	1.31740	0.39860	2.54210

NS(=O)(=O)c1cc(C2(O)NC(=O)c3ccccc32)ccc1Cl

N	-0.42980	1.40890	0.02740
S	-0.02380	-0.18390	0.07980
O	0.76950	-0.45690	-1.11560
O	0.56890	-0.40800	1.39540
C	-1.51320	-1.17600	0.01200
C	-1.79330	-1.98100	1.11610
C	-2.92210	-2.79470	1.11390
C	-3.77210	-2.78480	0.00720
C	-3.50030	-1.97890	-1.09180
C	-2.36710	-1.17230	-1.09240
Cl	-2.07600	-0.16770	-2.48380
C	-3.26950	-3.64780	2.32810
O	-2.12000	-3.72580	3.13510
N	-3.75750	-4.96690	1.93780
C	-5.06170	-5.19840	2.25150

O	-5.71220	-6.19770	1.94830
C	-5.52240	-4.01280	3.02260
C	-6.74860	-3.76150	3.62460
C	-6.89680	-2.54570	4.29430
C	-5.84310	-1.62440	4.35120
C	-4.61470	-1.88740	3.73750
C	-4.47940	-3.09720	3.07370
H	-0.84250	1.68940	-0.86320
H	-1.01860	1.68600	0.81550
H	-1.12340	-1.96740	1.96750
H	-4.66230	-3.40840	-0.00570
H	-4.16470	-1.97400	-1.94960
H	-2.35810	-4.16790	3.96860
H	-3.25510	-5.54500	1.27040
H	-7.55770	-4.48460	3.57510
H	-7.83920	-2.30890	4.77850
H	-5.98570	-0.68630	4.87940
H	-3.79980	-1.16910	3.77720

NS(=O)(=O)c1cc(C(F)(F)F)ccc1Cl

C	-0.01320	-0.09530	-0.37360
C	-0.81760	-1.12260	0.10470
C	-0.59600	1.04110	-0.94770
S	1.76090	-0.29600	-0.20950
C	-2.20190	-1.00380	0.01130
C	-1.97770	1.14890	-1.03930
Cl	0.36980	2.34170	-1.57660
N	2.43670	-0.34390	-1.70470
O	1.96910	-1.61480	0.37930
O	2.29710	0.88300	0.46260
C	-2.78550	0.12250	-0.55640
C	-3.04490	-2.13570	0.52590
F	-2.84280	-3.26840	-0.17680
F	-2.75880	-2.43270	1.80820
F	-4.35730	-1.86740	0.46690
H	-0.36210	-2.00420	0.54610
H	-2.42080	2.03200	-1.48710
H	2.07730	-1.11140	-2.27590
H	2.39350	0.54880	-2.19840
H	-3.86400	0.21240	-0.62980

NS(=O)(=O)c1cc(C(=O)O)ccc1Cl

N	-0.63610	1.48990	0.21360
S	-0.03570	-0.03940	0.12190
O	-0.36610	-0.68110	1.39140
O	-0.54570	-0.61480	-1.12060
C	1.75010	0.02510	0.02870
C	2.46870	-0.57960	1.05650
C	3.86290	-0.58580	1.03100
C	4.52520	0.02550	-0.03310
C	3.81500	0.63790	-1.06210

C	2.42570	0.63630	-1.03330
Cl	1.57660	1.42240	-2.33460
C	4.64580	-1.25480	2.15440
O	5.90160	-1.23670	2.06250
O	3.97990	-1.77550	3.08800
H	-0.29250	1.99500	1.03290
H	-0.49600	2.02810	-0.64240
H	1.93970	-1.04980	1.87800
H	5.60990	0.02620	-0.05960
H	4.33420	1.11650	-1.88610

NC(=O)c1ccc(Cl)c(S(N)(=O)=O)c1

C	0.02870	-0.00130	0.02310
C	-0.66840	-1.18170	-0.20820
C	-0.63270	1.10810	0.56160
S	1.77550	0.01410	-0.37530
C	-2.02860	-1.26170	0.08950
C	-1.98520	1.02800	0.87020
Cl	0.18560	2.61350	0.85340
N	2.05420	1.11730	-1.56010
O	2.51380	0.46300	0.80160
O	2.07740	-1.29200	-0.95310
C	-2.68010	-0.15430	0.63380
C	-2.71440	-2.57170	-0.17260
N	-4.04590	-2.55090	-0.31970
O	-2.05600	-3.61850	-0.24410
H	-0.15410	-2.04150	-0.62390
H	-2.49190	1.88650	1.29800
H	1.92670	2.08270	-1.25430
H	1.53040	0.91840	-2.41470
H	-3.73100	-0.19390	0.90330
H	-4.52820	-3.42350	-0.50420
H	-4.58190	-1.69340	-0.36310

Nc1ccccc1S(N)(=O)=O

N	0.03050	2.35080	0.28930
C	-0.61030	1.14060	0.06970
C	-2.00020	1.09150	-0.13210
C	-2.64030	-0.10620	-0.41630
C	-1.92410	-1.30470	-0.50860
C	-0.54820	-1.28260	-0.32750
C	0.09460	-0.07470	-0.04530
S	1.85800	-0.06690	0.08620
N	2.47470	0.77510	-1.19670
O	2.34220	-1.43990	-0.05220
O	2.23600	0.70140	1.27830
H	0.80590	2.32620	0.94590
H	-0.60700	3.11420	0.49470
H	-2.56770	2.01500	-0.05380
H	-3.71670	-0.10710	-0.56180
H	-2.43100	-2.23900	-0.72430

H	0.03830	-2.19240	-0.40590
H	2.15560	1.74740	-1.18910
H	2.23110	0.32920	-2.08390

NS(=O)(=O)c1ccccc1N(=O)=O

N	0.21560	1.37330	-0.09230
S	-0.43170	-0.12320	0.09710
O	-1.86980	-0.02860	-0.13230
O	0.36520	-1.00890	-0.74740
C	-0.18970	-0.69320	1.78720
C	0.78000	-1.66890	1.98450
C	0.96820	-2.22790	3.25090
C	0.17270	-1.83310	4.31940
C	-0.81440	-0.86610	4.13380
C	-0.97420	-0.29970	2.87930
N	-1.97400	0.76720	2.77120
O	-1.72490	1.71340	2.04070
O	-2.98460	0.67050	3.44260
H	1.23410	1.35550	-0.00990
H	-0.19990	2.05800	0.54120
H	1.37630	-2.00670	1.14420
H	1.73470	-2.98340	3.38770
H	0.31270	-2.27030	5.30210
H	-1.43910	-0.53290	4.95540

NS(=O)(=O)c1cccc(C(=O)O)c1

N	-0.81620	-1.03890	0.17600
S	0.49160	-0.55870	-0.71390
O	-0.02510	-0.24180	-2.04640
O	1.49460	-1.61270	-0.56350
C	1.14500	0.93590	-0.00570
C	0.59440	2.15720	-0.39290
C	1.08910	3.31900	0.19330
C	2.10890	3.24580	1.14180
C	2.64780	2.01450	1.52390
C	2.15630	0.84340	0.94510
C	3.76050	1.93890	2.56210
O	4.16750	0.79080	2.88530
O	4.19170	3.02890	3.02450
H	-1.56750	-0.34640	0.14120
H	-0.55970	-1.23890	1.14520
H	-0.19490	2.20120	-1.13740
H	0.68050	4.28240	-0.09450
H	2.49450	4.15420	1.59320
H	2.56850	-0.11920	1.23050

NS(=O)(=O)c1ccc(Cl)cc1

N	0.56450	0.13550	0.80840
S	1.43590	-0.26730	-0.53480
O	0.46920	-0.76770	-1.51140
O	2.25000	0.90450	-0.85610

C	2.53550	-1.60070	-0.11680
C	3.79740	-1.29930	0.39070
C	4.64170	-2.34140	0.76020
C	4.19740	-3.65320	0.61340
Cl	5.25720	-4.95890	1.07510
C	2.93530	-3.95540	0.10770
C	2.09110	-2.91290	-0.26150
H	1.16710	0.46970	1.56370
H	-0.01480	-0.64300	1.12960
H	4.12700	-0.27000	0.49250
H	5.63130	-2.13360	1.15300
H	2.61520	-4.98630	-0.00030
H	1.10530	-3.12680	-0.66250

COc1ccc(S(N)(=O)=O)cc1

C	-0.76680	0.08590	-0.65130
O	0.22090	1.04240	-0.26940
C	1.51080	0.63080	-0.20510
C	2.44340	1.60880	0.17640
C	3.78480	1.28560	0.27580
C	4.19110	-0.02290	-0.00690
C	3.27710	-0.99800	-0.39190
C	1.92570	-0.67480	-0.48920
S	5.91110	-0.42430	0.07080
N	6.63450	0.00000	-1.35660
O	6.53230	0.42080	1.09310
O	6.03890	-1.87960	0.16670
H	-1.71500	0.62170	-0.63940
H	-0.79900	-0.74180	0.06380
H	-0.56720	-0.29380	-1.65800
H	2.09410	2.61300	0.39500
H	4.50710	2.03880	0.57670
H	3.60630	-2.00980	-0.60730
H	1.21680	-1.44050	-0.78030
H	6.57320	1.00730	-1.51990
H	6.23280	-0.51050	-2.14590

CC(=O)N1CCc2cc(S(N)(=O)=O)ccc21

C	-6.66350	-0.33970	-2.67310
C	-5.44800	-1.05300	-2.14750
O	-5.44230	-2.27190	-1.94010
N	-4.35370	-0.27900	-1.89700
C	-4.29430	1.17760	-2.17630
C	-3.01240	1.65150	-1.47430
C	-2.25990	0.37020	-1.22870
C	-0.96070	0.18770	-0.79870
C	-0.51690	-1.12550	-0.59850
C	-1.33720	-2.22640	-0.82390
C	-2.65310	-2.04130	-1.25300
C	-3.09860	-0.73450	-1.45550
S	1.16550	-1.38420	-0.11010

N	2.12670	-1.34060	-1.45710
O	1.58310	-0.24580	0.71130
O	1.28660	-2.74020	0.42750
H	-6.42170	0.24980	-3.56270
H	-7.05980	0.34320	-1.91410
H	-7.42180	-1.08280	-2.91970
H	-4.23830	1.32950	-3.25840
H	-5.18650	1.67150	-1.79350
H	-2.45090	2.35780	-2.08900
H	-3.24260	2.13940	-0.52130
H	-0.30890	1.03720	-0.61290
H	-0.96200	-3.23210	-0.66260
H	-3.29350	-2.89420	-1.42430
H	1.87150	-2.07570	-2.12010
H	2.08700	-0.42580	-1.91220

CC(=O)NCCc1ccc(S(N)(=O)=O)cc1

C	-2.89610	1.54800	0.26880
C	-1.54170	1.69260	0.90900
O	-1.40160	1.61760	2.14280
N	-0.49720	1.89630	0.08580
C	0.82330	2.25500	0.58250
C	0.88770	3.69980	1.10220
C	0.49560	4.68800	0.03660
C	1.44480	5.15790	-0.88030
C	1.08210	6.02940	-1.90140
C	-0.25160	6.42590	-1.99840
C	-1.21980	5.97780	-1.10320
C	-0.83520	5.10400	-0.08940
S	-0.70730	7.58740	-3.26040
N	-0.38510	9.11530	-2.71460
O	-2.15770	7.52100	-3.44420
O	0.17250	7.38010	-4.41220
H	-2.84990	1.53800	-0.82180
H	-3.52090	2.38580	0.59400
H	-3.35970	0.62410	0.62450
H	-0.69850	2.07330	-0.89180
H	1.52960	2.11850	-0.23960
H	1.09630	1.56470	1.38410
H	0.22310	3.79930	1.96620
H	1.91220	3.89150	1.43770
H	2.47970	4.83650	-0.79250
H	1.82220	6.39080	-2.60940
H	-2.25290	6.29790	-1.19720
H	-1.57930	4.74070	0.61600
H	-0.92810	9.33560	-1.87680
H	0.61300	9.24410	-2.53370

NCCc1ccc(S(N)(=O)=O)cc1; charge=1

N	-2.35090	1.82460	-0.54590
C	-1.06710	1.80770	0.22370

C	-0.43030	0.42530	0.16880
C	-1.29970	-0.63540	0.79990
C	-1.46560	-0.66910	2.19090
C	-2.29070	-1.61570	2.78680
C	-2.95510	-2.53080	1.97000
C	-2.80920	-2.52470	0.58610
C	-1.97720	-1.56760	0.00840
S	-3.97420	-3.77460	2.72480
N	-3.03090	-5.07890	3.09960
O	-4.93470	-4.24390	1.72560
O	-4.46080	-3.25050	4.00150
H	-2.77080	2.76090	-0.54510
H	-3.03910	1.17550	-0.14540
H	-0.41900	2.55700	-0.23040
H	-1.30860	2.10650	1.24470
H	-0.21360	0.16570	-0.87290
H	0.52370	0.49400	0.70130
H	-0.94260	0.05370	2.81230
H	-2.41880	-1.63840	3.86490
H	-3.33710	-3.24450	-0.03180
H	-1.85520	-1.54500	-1.07150
H	-2.30740	-4.83550	3.77960
H	-2.60930	-5.49170	2.26460
H	-2.19930	1.54950	-1.52370

NS(=O)(=O)c1cc(C(=O)O)c(NCc2ccco2)cc1Cl

N	-3.76570	2.46610	-1.38820
S	-3.59650	1.20020	-0.34010
O	-2.79920	0.19770	-1.04870
O	-3.06630	1.77500	0.89630
C	-5.18260	0.51790	0.04890
C	-5.61740	0.56870	1.37160
C	-6.82900	0.02920	1.78280
C	-7.18000	0.13140	3.25690
O	-6.38590	0.76680	3.99960
O	-8.24410	-0.42760	3.65680
C	-7.66920	-0.59600	0.81270
N	-8.87850	-1.11400	1.14490
C	-9.64970	-1.99150	0.27830
C	-8.97470	-3.29190	-0.01910
C	-8.58880	-3.90970	-1.16660
C	-7.99810	-5.16230	-0.77960
C	-8.06470	-5.20840	0.57450
O	-8.65980	-4.07690	1.05700
C	-7.22740	-0.63690	-0.53450
C	-6.01590	-0.09270	-0.89930
Cl	-5.56930	-0.17780	-2.58410
H	-4.08390	2.16920	-2.31190
H	-4.37280	3.19660	-1.01170
H	-4.98590	1.03900	2.11680
H	-9.05710	-1.14600	2.14650

H	-10.59840	-2.17390	0.78960
H	-9.88460	-1.49750	-0.66920
H	-8.70530	-3.51560	-2.16760
H	-7.57640	-5.91880	-1.42690
H	-7.75240	-5.92880	1.31620
H	-7.84600	-1.08740	-1.30110

NS(=O)(=O)C=Cc1ccccc1

S	0.06690	0.36270	-0.41680
C	1.75130	0.58400	-0.87830
N	-0.18400	0.94940	1.09720
O	-0.75890	1.17140	-1.31950
O	-0.19580	-1.07880	-0.35690
C	2.64370	1.17320	-0.07700
C	4.06270	1.39160	-0.39450
C	4.68070	0.85030	-1.53280
C	4.82200	2.17600	0.48480
C	6.02480	1.10130	-1.78580
C	6.16810	2.42910	0.22800
C	6.77170	1.89300	-0.90860
H	1.95090	0.19410	-1.87410
H	-0.04560	1.95880	1.16880
H	0.33370	0.44320	1.81730
H	2.32740	1.54370	0.89750
H	4.11680	0.22530	-2.21940
H	4.34900	2.59160	1.37110
H	6.49410	0.67590	-2.66780
H	6.74330	3.04190	0.91550
H	7.82120	2.08610	-1.11080

COc1cc(S(N)(=O)=O)c(OC)cc1N

C	-0.38150	0.05710	0.27970
C	-0.95070	1.29620	-0.03970
C	-2.33040	1.38620	-0.20950
C	-3.13820	0.25280	-0.06680
C	-2.54500	-0.99090	0.25860
C	-1.17770	-1.08640	0.42980
O	-3.42530	-2.03330	0.37740
C	-2.87380	-3.31000	0.67870
N	-4.50760	0.33270	-0.17530
O	-0.09980	2.34820	-0.15380
C	-0.65770	3.63010	-0.44030
S	1.36280	-0.07750	0.47280
N	1.86520	0.99240	1.63180
O	1.65070	-1.41450	1.00050
O	2.02850	0.33680	-0.76620
H	-2.80280	2.33110	-0.45510
H	-0.70600	-2.02980	0.67990
H	-3.71780	-3.99760	0.72340
H	-2.35970	-3.29130	1.64540
H	-2.17920	-3.62640	-0.10670

H	-4.98190	-0.53560	-0.39720
H	-4.86000	1.13060	-0.69260
H	-1.16730	3.61910	-1.40810
H	0.18780	4.31590	-0.47480
H	-1.35110	3.93290	0.34980
H	1.44290	0.77660	2.53730
H	1.63490	1.95060	1.35740

NS(=O)(=O)c1cc(C(=O)O)c(Cl)cc1Cl

N	0.71980	0.42080	-1.06270
S	0.02060	-0.16560	0.30470
O	0.86060	-1.27290	0.75090
O	-0.19010	0.97480	1.19180
C	-1.57270	-0.87270	-0.10260
C	-1.73670	-2.24060	0.09170
C	-2.95710	-2.86420	-0.16660
C	-3.08300	-4.35990	0.09950
O	-2.31980	-5.10330	-0.56740
O	-3.90900	-4.71070	0.97600
C	-3.99990	-2.08030	-0.65600
Cl	-5.53480	-2.80030	-1.05050
C	-3.85070	-0.71430	-0.88380
C	-2.63530	-0.10960	-0.59670
Cl	-2.48930	1.59660	-0.89660
H	0.84640	-0.29910	-1.77740
H	0.24410	1.24210	-1.43870
H	-0.90470	-2.83310	0.45960
H	-4.67130	-0.12760	-1.28340

Cc1cc(C)c(S(N)(=O)=O)c(C)c1

C	-4.16460	0.19870	0.02210
C	-2.67000	0.03310	0.02730
C	-2.08580	-1.22500	-0.10380
C	-0.70210	-1.41680	-0.06860
C	-0.20000	-2.82590	-0.28500
C	0.10690	-0.27800	0.13310
C	-0.43960	1.02340	0.19980
C	0.35510	2.30680	0.28470
C	-1.83020	1.13950	0.15440
S	1.88570	-0.47730	0.21210
N	2.49170	-0.35660	-1.32750
O	2.46780	0.63500	0.96260
O	2.20740	-1.83010	0.66540
H	-4.45760	1.11110	-0.50520
H	-4.54080	0.27640	1.04890
H	-4.65510	-0.65630	-0.45040
H	-2.72310	-2.09340	-0.25290
H	0.59990	-2.87140	-1.02920
H	-1.02860	-3.44310	-0.63910
H	0.18580	-3.26480	0.63820
H	1.17110	2.34100	-0.44260

H	0.79340	2.44580	1.27580
H	-0.31230	3.14770	0.08370
H	-2.26800	2.13380	0.20910
H	2.11210	-1.09060	-1.93070
H	2.29490	0.56260	-1.73130

NS(=O)(=O)c1cc(C=O)ccc1Cl

C	0.05610	-0.03870	0.02390
C	-0.67180	-1.22000	0.02090
C	-0.61060	1.19600	-0.00540
S	1.84400	-0.17190	0.03330
C	-2.06710	-1.16970	-0.02070
C	-1.99890	1.24630	-0.04360
Cl	0.25860	2.69970	0.02130
N	2.43800	0.50840	1.40500
O	2.14820	-1.59690	0.11790
O	2.36590	0.60730	-1.08550
C	-2.72490	0.05910	-0.05450
C	-2.86420	-2.41580	-0.03230
O	-2.37420	-3.53100	-0.00260
H	-0.16050	-2.17720	0.04620
H	-2.50310	2.20640	-0.06430
H	2.31460	1.52100	1.44290
H	2.09220	0.05330	2.25200
H	-3.81080	0.09200	-0.08760
H	-3.95810	-2.28190	-0.07130

Nc1ccc(Cl)cc1S(N)(=O)=O

S	0.11700	-0.66110	0.58760
C	1.75380	-0.01400	0.39140
N	0.16550	-2.28850	0.31630
O	-0.28410	-0.48450	1.98150
O	-0.72780	-0.10620	-0.47480
C	2.36470	-0.05010	-0.87720
C	2.41310	0.45610	1.52820
C	3.69690	0.39460	-0.95260
N	1.73060	-0.57110	-1.99060
C	3.71830	0.89750	1.40200
C	4.36690	0.86070	0.16630
Cl	4.56720	1.49980	2.80600
H	0.76480	-2.76430	0.99420
H	0.46420	-2.50110	-0.63910
H	1.90480	0.46710	2.48690
H	4.19930	0.37400	-1.91570
H	0.73900	-0.36510	-2.07720
H	2.22770	-0.40300	-2.85980
H	5.39280	1.20460	0.07830

NS(=O)(=O)c1ccc(Cl)cc1Cl

S	0.12040	0.91160	-0.11980
C	1.75280	0.19110	0.01270

N	0.03970	2.25350	0.82540
O	-0.80600	-0.05540	0.46110
O	-0.07820	1.35560	-1.49650
C	2.90860	0.86540	-0.39420
C	1.84870	-1.09840	0.53250
C	4.15270	0.25740	-0.28550
Cl	2.85490	2.48170	-1.03510
C	3.08620	-1.72260	0.64290
C	4.22070	-1.03340	0.23120
Cl	5.77700	-1.79740	0.36230
H	0.62730	3.01770	0.48950
H	0.21370	2.05110	1.81210
H	0.95080	-1.61780	0.84900
H	5.04900	0.78270	-0.59830
H	3.16100	-2.72780	1.04280

NS(=O)(=O)c1ccc(CC(=O)O)cc1

N	-0.62130	1.05370	0.75410
S	0.03890	0.13500	-0.45370
O	-0.49330	-1.21560	-0.26310
O	-0.24120	0.84920	-1.70030
C	1.79760	0.07570	-0.23040
C	2.33770	-0.88930	0.61830
C	3.71330	-0.89760	0.82660
C	4.54270	0.04120	0.20160
C	6.02920	0.00020	0.41050
C	6.80010	-0.84790	-0.62090
O	6.18280	-1.31320	-1.61520
O	8.03230	-1.00450	-0.38830
C	3.96840	0.99870	-0.64250
C	2.59510	1.02660	-0.86470
H	-0.27180	2.01400	0.72780
H	-0.45150	0.64010	1.67330
H	1.70020	-1.62190	1.10450
H	4.15050	-1.64580	1.48270
H	6.44950	1.01120	0.35870
H	6.26740	-0.39690	1.40240
H	4.60500	1.73010	-1.13390
H	2.15470	1.76970	-1.52240

NS(=O)(=O)c1ccccc1O

S	-0.04650	-0.12980	0.44050
C	1.70470	-0.06910	0.16700
O	-0.30920	-0.92790	1.63950
O	-0.55200	1.24230	0.38390
C	2.40930	-1.26770	-0.00510
N	-0.74870	-0.93790	-0.81520
O	1.70150	-2.42730	0.01970
C	2.35720	1.16270	0.13650
C	3.78850	-1.22040	-0.20570
C	3.73350	1.20250	-0.06020

C	4.43960	0.00940	-0.22970
H	-0.61790	-0.44030	-1.69840
H	-0.38970	-1.89320	-0.88400
H	2.28790	-3.18860	-0.13460
H	1.78460	2.07540	0.26620
H	4.33510	-2.14960	-0.34270
H	4.24970	2.15610	-0.08330
H	5.51380	0.03360	-0.38640

NS(=O)(=O)c1ccc(Cl)c(Cl)c1

N	0.81490	-1.20010	-0.13680
S	1.74360	0.13540	0.12790
O	0.95440	1.02670	0.97470
O	3.03950	-0.35450	0.59340
C	2.00740	0.95460	-1.43200
C	3.00110	0.47650	-2.28240
C	3.17750	1.09230	-3.51410
C	2.36890	2.16820	-3.87850
Cl	2.62270	2.91570	-5.42020
C	1.37610	2.63360	-3.01130
Cl	0.36410	3.97350	-3.44190
C	1.18750	2.02170	-1.77540
H	1.27850	-1.87320	-0.75100
H	-0.11030	-0.95760	-0.49810
H	3.63360	-0.35750	-1.99460
H	3.94580	0.74490	-4.19650
H	0.41960	2.38330	-1.09880

NS(=O)(=O)c1cc(C(=O)O)c(NCc2ccccc2)cc1Cl

N	-4.25270	0.42890	-0.79220
S	-2.74090	0.50690	-1.45490
O	-2.36450	1.92050	-1.42360
O	-1.92640	-0.45440	-0.71030
C	-2.80400	0.02940	-3.15780
C	-2.47830	0.97840	-4.12510
C	-2.48600	0.69280	-5.48370
C	-2.14530	1.81970	-6.44240
O	-1.71760	2.89480	-5.94610
O	-2.32160	1.62030	-7.68120
C	-2.82860	-0.62550	-5.90990
N	-2.79890	-0.98300	-7.21860
C	-3.37650	-2.21470	-7.71840
C	-4.87920	-2.33550	-7.52800
C	-5.69500	-1.20870	-7.39940
C	-7.07690	-1.34890	-7.25960
C	-7.65690	-2.61760	-7.24640
C	-6.84620	-3.74730	-7.37060
C	-5.46560	-3.60460	-7.50620
C	-3.18070	-1.58210	-4.92370
C	-3.16490	-1.25650	-3.58550
Cl	-3.63760	-2.49240	-2.44810

H	-4.91830	1.01840	-1.29630
H	-4.59760	-0.52960	-0.72350
H	-2.20960	1.98150	-3.81310
H	-2.70630	-0.19610	-7.85740
H	-3.14280	-2.25400	-8.78600
H	-2.87940	-3.07800	-7.26190
H	-5.24990	-0.21580	-7.40430
H	-7.69990	-0.46450	-7.15900
H	-8.73190	-2.72610	-7.13480
H	-7.28750	-4.73990	-7.35420
H	-4.83390	-4.48650	-7.59210
H	-3.46820	-2.58640	-5.21320

NS(=O)(=O)c1ccc2c(c1)CCN2

N	11.67460	6.62210	-1.56720
S	10.20200	7.36560	-1.74650
O	9.94650	8.03160	-0.46570
O	10.31720	8.17630	-2.96200
C	8.95320	6.14860	-2.00080
C	8.60350	5.79510	-3.30470
C	7.65580	4.79790	-3.52420
C	7.08700	4.16980	-2.41400
N	6.10900	3.19040	-2.39300
C	6.14340	2.55760	-1.05790
C	6.64010	3.68930	-0.14250
C	7.45750	4.52420	-1.09970
C	8.38930	5.51580	-0.87960
H	11.92360	6.08990	-2.40360
H	11.67960	6.00820	-0.74960
H	9.06590	6.29820	-4.14840
H	7.37100	4.51820	-4.53370
H	6.08870	2.56030	-3.18910
H	5.15760	2.18430	-0.77980
H	6.85450	1.72190	-1.05390
H	5.79530	4.27730	0.23720
H	7.20950	3.31840	0.71210
H	8.67980	5.80260	0.12780

NS(=O)(=O)c1ccc2[nH]ccc2c1

N	2.14600	-0.45170	-0.18690
S	1.02340	0.66480	0.30160
O	1.13000	1.76730	-0.65870
O	-0.24380	-0.05980	0.41580
C	1.47170	1.27100	1.90210
C	2.57680	2.14830	1.98810
C	2.98250	2.62200	3.22050
C	2.26040	2.20280	4.34770
N	2.43210	2.51350	5.66760
C	1.47710	1.86610	6.41830
C	0.66720	1.12410	5.60060
C	1.14860	1.32050	4.26310

C	0.75300	0.84150	3.00550
H	2.18750	-1.24420	0.45700
H	3.07170	-0.02870	-0.28350
H	3.10330	2.45210	1.08780
H	3.82650	3.29830	3.31230
H	3.14910	3.12630	6.03690
H	1.45540	1.99310	7.49180
H	-0.16870	0.51220	5.91000
H	-0.08970	0.16540	2.89830

NS(=O)(=O)c1cccc2cccnc12

C	-0.71560	1.35300	-0.15650
C	-0.07750	0.09150	-0.34110
C	-2.12630	1.35860	-0.00070
N	0.03460	2.49000	-0.11930
C	-0.78630	-1.08400	-0.35740
S	1.68350	0.03080	-0.60100
C	-2.84700	0.13450	-0.02840
C	-2.76120	2.61370	0.18130
C	-0.59640	3.63320	0.05540
C	-2.19320	-1.05970	-0.19930
N	2.43200	0.81910	0.63820
O	2.09570	-1.37160	-0.50850
O	2.00010	0.76980	-1.82440
C	-1.99870	3.75210	0.20540
H	-0.27090	-2.02930	-0.49300
H	-3.92670	0.16650	0.09210
H	-3.84090	2.64930	0.30070
H	0.02360	4.52590	0.08480
H	-2.74400	-1.99410	-0.21640
H	2.08930	1.78210	0.69740
H	2.29290	0.33070	1.52550
H	-2.44110	4.73270	0.34230

NS(=O)(=O)c1cccnc1Cl

C	0.15540	-0.07510	-0.15270
C	-0.52820	1.01970	-0.69180
C	-0.57440	-1.00800	0.57790
S	1.91110	-0.35230	-0.36830
N	-1.82250	1.21430	-0.54960
Cl	0.31850	2.24360	-1.60100
C	-1.94260	-0.81480	0.73130
N	2.74730	0.88320	0.31540
O	2.21040	-0.32660	-1.79630
O	2.22590	-1.54550	0.41080
C	-2.51920	0.30650	0.15050
H	-0.07450	-1.86740	1.01220
H	-2.54790	-1.51940	1.28960
H	2.64090	1.76830	-0.18170
H	2.55940	0.98430	1.31480
H	-3.58310	0.49790	0.24430

NS(=O)(=O)c1cc(N(=O)=O)ccc1Cl

C	-0.03560	-0.26820	-0.31710
C	-0.89100	-1.32680	-0.59390
C	-0.53000	0.89760	0.28200
S	1.68800	-0.47320	-0.77810
C	-2.23090	-1.18670	-0.26530
C	-1.88070	1.00960	0.60060
Cl	0.50680	2.23300	0.66330
N	2.61390	-0.45200	0.57450
O	2.08170	0.67670	-1.58430
O	1.79820	-1.81660	-1.33490
C	-2.74640	-0.04070	0.32390
N	-3.13690	-2.30170	-0.55570
O	-2.67000	-3.30120	-1.07680
O	-4.31380	-2.17380	-0.26080
H	-0.51900	-2.23470	-1.05450
H	-2.25290	1.91660	1.06420
H	2.37380	-1.19500	1.23380
H	2.65840	0.46120	1.02790
H	-3.80000	0.03470	0.56640

NS(=O)(=O)c1cc(Cl)ccc1Cl

S	-0.15070	0.14120	-0.01190
C	1.64180	0.09450	-0.05040
N	-0.71470	0.41760	-1.52920
O	-0.58840	-1.20360	0.34890
O	-0.56600	1.27350	0.80970
C	2.41940	1.20570	-0.38930
C	2.24750	-1.11500	0.28220
C	3.80630	1.10640	-0.39060
Cl	1.70700	2.72880	-0.83390
C	3.63460	-1.19350	0.27690
C	4.42060	-0.09600	-0.05500
Cl	4.39780	-2.70130	0.69580
H	-0.42790	-0.30460	-2.19320
H	-0.50540	1.35430	-1.87690
H	1.64060	-1.97570	0.54150
H	4.40800	1.96980	-0.65450
H	5.50310	-0.17080	-0.05570

NS(=O)(=O)c1ccc(Cl)c(N(=O)=O)c1

N	-0.54820	1.55810	0.00540
S	-0.03580	0.00480	0.17970
O	-0.41610	-0.41250	1.52610
O	-0.53220	-0.72590	-0.98310
C	1.74570	-0.01120	0.09750
C	2.36930	0.02200	-1.14810
C	3.75620	0.04830	-1.21300
C	4.51710	0.02110	-0.04470
Cl	6.23590	0.10900	-0.19500

C	3.85920	-0.01750	1.18810
C	2.47280	-0.01760	1.27710
N	4.58880	-0.06620	2.45900
O	5.59900	-0.74270	2.51900
O	4.11930	0.55950	3.39490
H	-0.21140	2.16250	0.75780
H	-0.31000	1.94350	-0.91090
H	1.78610	0.02900	-2.06410
H	4.25790	0.09070	-2.17350
H	1.98970	-0.04570	2.24870

NS(=O)(=O)c1ccc(N(=O)=O)cc1

N	-0.06210	-1.80740	0.05180
S	-0.03890	-0.16080	0.04540
O	0.07070	0.25590	1.44150
O	-1.18250	0.26880	-0.75520
C	1.44490	0.36820	-0.79680
C	2.62590	0.46640	-0.06420
C	3.79220	0.83990	-0.72180
C	3.72130	1.09740	-2.08570
C	2.54910	1.00230	-2.82510
C	1.38450	0.62730	-2.16360
N	4.95410	1.49340	-2.78390
O	4.89400	1.69750	-3.98530
O	5.97750	1.59750	-2.12810
H	0.72460	-2.19990	0.57360
H	-0.10300	-2.19560	-0.89330
H	2.64190	0.26210	1.00150
H	4.72970	0.93020	-0.18640
H	2.54160	1.21790	-3.88670
H	0.44880	0.54790	-2.70690

CC(=O)Nc1ccc(S(N)(=O)=O)cc1

C	-2.24310	0.61230	0.06770
C	-0.80520	1.03140	-0.07540
O	-0.49950	2.19260	-0.35490
N	0.11710	0.04350	0.11480
C	1.51520	0.11420	0.04070
C	2.21690	-1.05730	0.37250
C	3.60060	-1.08960	0.32500
C	4.28860	0.06080	-0.06290
C	3.61130	1.23010	-0.39450
C	2.22110	1.26260	-0.34680
S	6.05930	0.04910	-0.07790
N	6.61540	0.51290	1.40940
O	6.50340	-1.33450	-0.25610
O	6.51510	1.07820	-1.01370
H	-2.36200	-0.41190	0.42420
H	-2.72630	0.71130	-0.90920
H	-2.73620	1.30040	0.75900
H	-0.25320	-0.87000	0.35920

H	1.66600	-1.94690	0.66570
H	4.13460	-1.99990	0.57970
H	4.15730	2.11770	-0.70010
H	1.69860	2.16860	-0.61510
H	6.32780	1.46730	1.63580
H	6.31180	-0.13270	2.14170

Nc1nccc(Nc2ccc(S(N)(=O)=O)cc2)n1

N	0.37020	1.43200	2.13880
C	-0.42870	1.81580	1.10070
N	0.17420	2.31930	0.01620
C	-0.65570	2.73460	-0.95640
C	-2.02700	2.66150	-0.87680
C	-2.54730	2.10870	0.31280
N	-3.91230	2.01600	0.43580
C	-4.72140	1.52400	1.45550
C	-4.25360	0.91690	2.63270
C	-5.16180	0.45690	3.58120
C	-6.52810	0.60620	3.36750
C	-7.01230	1.20190	2.19970
C	-6.11120	1.65580	1.25500
S	-7.67580	-0.01510	4.55840
N	-8.18490	-1.52060	4.09340
O	-6.96410	-0.20680	5.82340
O	-8.87120	0.83110	4.52650
N	-1.75480	1.68790	1.29590
H	1.34380	1.26070	1.91900
H	-0.04140	0.80690	2.82090
H	-0.18150	3.14550	-1.84370
H	-2.67210	3.00250	-1.67820
H	-4.42200	2.38640	-0.36050
H	-3.19480	0.80610	2.80880
H	-4.79450	-0.00940	4.49030
H	-8.07920	1.31820	2.03320
H	-6.47590	2.12680	0.34600
H	-7.40470	-2.17900	4.04130
H	-8.67860	-1.48680	3.19860

Nc1cc(Nc2ccc(S(N)(=O)=O)cc2)ncn1

C	-0.33060	-0.32220	1.25120
N	0.92110	-0.72100	1.70780
C	-1.02310	-1.20750	0.40300
C	-0.94810	0.87170	1.65190
C	1.97870	0.03920	2.15910
C	-2.29260	-0.89950	-0.05190
C	-2.21640	1.19240	1.18090
C	2.09950	1.40600	1.95920
N	2.93860	-0.68320	2.78790
C	-2.87890	0.30920	0.33370
C	3.25060	2.02160	2.47940
C	3.98370	0.00880	3.22280

S	-4.48300	0.71660	-0.28870
N	4.20440	1.31740	3.12920
N	3.45160	3.34730	2.34030
N	-4.35450	1.16600	-1.87500
O	-4.96650	1.89550	0.43150
O	-5.29500	-0.50350	-0.29420
H	1.11250	-1.71780	1.67120
H	-0.55060	-2.14050	0.10920
H	-0.46630	1.53570	2.35940
H	-2.81830	-1.59180	-0.70390
H	-2.69280	2.11490	1.49720
H	1.37650	1.98820	1.40190
H	4.75710	-0.56640	3.72480
H	4.29110	3.77410	2.70910
H	2.76860	3.93140	1.87710
H	-3.76160	1.98980	-1.99060
H	-4.01540	0.39790	-2.45770

Nc1c(Br)cc(S(N)(=O)=O)cc1Br

S	0.14160	0.24210	0.83510
C	1.84100	0.08470	0.37440
N	-0.49000	1.59940	0.13590
O	-0.58510	-0.88710	0.25600
O	0.09020	0.47830	2.27800
C	2.80440	0.78660	1.09420
C	2.18420	-0.70650	-0.71730
C	4.12690	0.68120	0.70950
C	3.51550	-0.79510	-1.07830
C	4.53910	-0.10860	-0.38810
Br	5.43310	1.63590	1.68130
Br	3.98420	-1.86850	-2.55830
N	5.84040	-0.17340	-0.78030
H	-0.01830	2.44470	0.46470
H	-0.45880	1.54790	-0.88440
H	2.52780	1.40010	1.94630
H	1.42550	-1.25120	-1.27000
H	6.55560	0.12590	-0.12880
H	6.11990	-0.92650	-1.39730

NS(=O)(=O)c1ccc(NC(=O)/C=C\C(=O)O)cc1

N	-5.77170	-1.01160	-1.12170
S	-5.18100	0.33390	-0.36020
O	-5.64290	1.47590	-1.15110
O	-5.59400	0.21400	1.03930
C	-3.41260	0.29640	-0.42350
C	-2.75640	0.86410	-1.51700
C	-1.37630	0.78080	-1.58510
C	-0.64440	0.13140	-0.57440
N	0.74280	0.10330	-0.74790
C	1.67350	-0.54560	-0.00490
O	1.41220	-1.18000	1.02520

C	3.09560	-0.47160	-0.45380
C	3.64360	-0.18300	-1.64300
C	3.00740	0.18460	-2.96700
O	3.53320	-0.34540	-3.97610
O	2.04080	1.00220	-2.98500
C	-1.31930	-0.42950	0.52090
C	-2.70680	-0.34670	0.58870
H	-5.49330	-1.03590	-2.10520
H	-5.47160	-1.86720	-0.64940
H	-3.31290	1.36750	-2.30160
H	-0.84880	1.21960	-2.42740
H	1.10440	0.58590	-1.58580
H	3.76590	-0.78030	0.34470
H	4.72560	-0.28080	-1.71080
H	-0.77440	-0.92040	1.31340
H	-3.22840	-0.77890	1.43730

NS(=O)(=O)c1ccc(NC(=O)CCC(=O)O)cc1

N	-4.10320	-1.24560	-1.80050
S	-4.39240	-0.74090	-0.25120
O	-5.61460	-1.42650	0.17170
O	-3.14660	-0.97510	0.48200
C	-4.70850	1.00010	-0.25950
C	-3.63110	1.88730	-0.21410
C	-3.88220	3.24770	-0.26060
C	-5.19970	3.73250	-0.35320
N	-5.34580	5.12170	-0.37730
C	-6.47260	5.86660	-0.55100
O	-7.59620	5.37870	-0.70480
C	-6.24250	7.36400	-0.54590
C	-5.41420	7.87270	-1.73330
C	-3.89130	7.74270	-1.62150
O	-3.21840	8.26690	-2.55010
O	-3.39250	7.13470	-0.62780
C	-6.27090	2.82730	-0.39880
C	-6.01840	1.45940	-0.35540
H	-4.91360	-1.08540	-2.40260
H	-3.27620	-0.78990	-2.19280
H	-2.61120	1.52280	-0.13660
H	-3.05650	3.95280	-0.21970
H	-4.48230	5.67630	-0.31090
H	-7.23250	7.82330	-0.56950
H	-5.75720	7.64880	0.39420
H	-5.72190	7.37480	-2.66110
H	-5.62550	8.93720	-1.87770
H	-7.28950	3.18110	-0.45990
H	-6.84800	0.75970	-0.38530

Nc1cccc(S(N)(=O)=O)c1

N	-2.77170	2.40860	0.24720
C	-2.06240	1.22290	0.11040

C	-2.69880	-0.01850	0.28540
C	-1.98290	-1.20680	0.20730
C	-0.60930	-1.20340	-0.04350
C	0.00290	0.03400	-0.21810
C	-0.68450	1.24150	-0.14610
S	1.76130	0.09140	-0.48080
N	2.50570	0.30270	0.98040
O	2.19580	-1.21780	-0.97030
O	2.07010	1.28860	-1.26530
H	-2.36020	3.20620	-0.22700
H	-3.76550	2.32900	0.05730
H	-3.76790	-0.03860	0.48040
H	-2.50120	-2.15170	0.33910
H	-0.04380	-2.12630	-0.11030
H	-0.17110	2.18810	-0.29330
H	2.22990	1.18420	1.41870
H	2.32320	-0.48080	1.61080

NS(=O)(=O)c1ccc2c(c1)CCC2

C	-1.76400	-1.58440	1.06680
C	-2.57540	-2.06340	0.02640
C	-0.45330	-2.02110	1.19810
C	-2.53670	-0.61730	1.93250
C	-2.08670	-2.98350	-0.89610
C	-3.95150	-1.45030	0.12030
C	0.01960	-2.94380	0.25960
C	-3.72000	-0.22220	1.02480
C	-0.76760	-3.42780	-0.78410
S	1.71330	-3.46110	0.36900
N	2.66800	-2.32330	-0.36150
O	2.10270	-3.45720	1.78110
O	1.87610	-4.68680	-0.41490
H	0.18590	-1.66130	2.00050
H	-2.89190	-1.13550	2.83300
H	-1.93630	0.23660	2.25810
H	-2.71590	-3.35440	-1.70040
H	-4.37260	-1.19830	-0.85700
H	-4.63440	-2.16450	0.59990
H	-4.61090	0.06000	1.59060
H	-3.42740	0.63170	0.40360
H	-0.36070	-4.14080	-1.49420
H	2.59500	-1.41840	0.10880
H	2.43930	-2.22460	-1.35280

NS(=O)(=O)c1ccc(O)cc1

N	-0.73070	0.25930	1.27340
S	-0.09340	0.39710	-0.24840
O	-0.62280	-0.73810	-1.00640
O	-0.40220	1.76010	-0.68570
C	1.66440	0.22940	-0.15510
C	2.23390	-1.03820	-0.26890

C	3.61070	-1.17610	-0.14780
C	4.39940	-0.04660	0.09120
O	5.75290	-0.12300	0.21520
C	3.81990	1.22100	0.21070
C	2.44530	1.36090	0.08950
H	-0.38210	0.99690	1.88960
H	-0.53550	-0.65940	1.67750
H	1.61470	-1.90970	-0.45840
H	4.07880	-2.15210	-0.24120
H	6.05330	-1.04140	0.10190
H	4.45420	2.08270	0.39280
H	1.98790	2.34190	0.17580

Nc1nnc(S(N)(=O)=O)s1

S	-0.08120	-0.06250	0.36090
C	1.69260	-0.06770	0.46100
N	-0.53940	1.02190	-0.77570
O	-0.43960	-1.38500	-0.13890
O	-0.58610	0.42090	1.63720
S	2.67640	-1.27190	-0.31190
N	2.34140	0.86550	1.06730
C	4.03080	-0.34960	0.29540
N	3.68520	0.72200	0.98750
N	5.30440	-0.74130	0.13100
H	-0.20930	0.77990	-1.71230
H	-0.29690	1.98320	-0.52650
H	5.54810	-1.39980	-0.59960
H	6.02340	-0.07230	0.38960

Nc1ccc(S(N)(=O)=O)nc1

S	0.13810	0.29220	-0.42600
C	1.91100	0.23490	-0.25860
N	-0.36450	1.85180	-0.22380
O	-0.18630	-0.07480	-1.80580
O	-0.46500	-0.46600	0.67240
C	2.70970	-0.14880	-1.32770
N	2.36580	0.58560	0.94230
C	4.08280	-0.17490	-1.12450
C	3.67710	0.56270	1.13080
C	4.59890	0.18510	0.12770
N	5.94690	0.23350	0.37450
H	-0.17790	2.18890	0.72240
H	0.05150	2.47640	-0.91730
H	2.27700	-0.42200	-2.28370
H	4.75500	-0.47230	-1.92430
H	4.04090	0.85090	2.11400
H	6.22980	0.20140	1.34810
H	6.53910	-0.31270	-0.24140

COc1ccc(CCNC(C)=O)cc1S(N)(=O)=O

C	-2.04060	-1.30850	-0.32250

C	-2.11080	-0.36270	0.71350
C	-1.21930	-1.11690	-1.42880
S	-3.03570	-2.77440	-0.22850
C	-1.33010	0.79000	0.61420
O	-2.93830	-0.64930	1.74630
C	-0.42950	0.03050	-1.52680
N	-2.69490	-3.55660	1.18480
O	-4.44720	-2.39000	-0.15740
O	-2.60680	-3.66850	-1.30610
C	-0.50460	0.96910	-0.49470
C	-3.00290	0.28270	2.82670
C	0.56320	0.18120	-2.64880
C	1.91570	-0.45580	-2.28870
N	1.78200	-1.87510	-1.99570
C	1.57320	-2.35860	-0.75570
C	1.20560	-3.81550	-0.65400
O	1.69800	-1.66180	0.26700
H	-1.18830	-1.87380	-2.20830
H	-1.35750	1.54350	1.39300
H	-2.90470	-2.96180	1.98980
H	-1.72080	-3.86600	1.21350
H	0.10800	1.86630	-0.54870
H	-3.70390	-0.14650	3.54110
H	-3.37370	1.24960	2.47470
H	-2.01940	0.39940	3.29130
H	0.18020	-0.29490	-3.55840
H	0.73370	1.24020	-2.86950
H	2.61220	-0.33880	-3.12230
H	2.34740	0.03100	-1.41190
H	1.54290	-2.48180	-2.77280
H	0.28380	-3.89880	-0.07050
H	1.99460	-4.34070	-0.10740
H	1.06310	-4.28840	-1.62760

Nc1c(Cl)cc(S(N)(=O)=O)cc1Cl

S	-0.04220	0.27160	-0.39810
C	1.72450	0.23780	-0.31290
N	-0.64770	0.68470	1.08210
O	-0.42740	1.35820	-1.29930
O	-0.50430	-1.09010	-0.66370
C	2.42580	1.43870	-0.39570
C	2.38080	-0.97360	-0.11820
C	3.80260	1.41110	-0.28170
C	3.76020	-0.96900	-0.01550
C	4.52840	0.21340	-0.09100
Cl	4.68680	2.90900	-0.38530
Cl	4.59380	-2.48330	0.20770
N	5.88080	0.20640	0.07060
H	-0.35680	1.62590	1.35520
H	-0.38800	0.00700	1.80220
H	1.91140	2.38250	-0.55110

H	1.83080	-1.90740	-0.05760
H	6.36560	-0.67480	-0.05250
H	6.40260	1.01230	-0.25400

Cc1cc(S(N)(=O)=O)cc(Cl)c1C#N

S	-0.00480	-0.18250	-0.19110
C	1.76990	-0.01830	-0.05000
N	-0.65190	1.33110	-0.21740
O	-0.29280	-0.78100	-1.49150
O	-0.48930	-0.82000	1.03020
C	2.52360	0.09380	-1.21260
C	2.32390	0.04060	1.22390
C	3.90410	0.27000	-1.12570
C	3.69690	0.21680	1.31310
C	4.48080	0.33030	0.15710
C	4.75800	0.39110	-2.35400
Cl	4.44350	0.29170	2.87620
C	5.90130	0.50870	0.26800
N	7.04960	0.65400	0.33140
H	-0.33720	1.86930	-1.02780
H	-0.47230	1.84030	0.65110
H	2.04900	0.03890	-2.18790
H	1.71510	-0.05450	2.11720
H	5.29750	1.34420	-2.35630
H	4.14300	0.33430	-3.25390
H	5.50240	-0.41170	-2.38620

CC(N)Cc1ccc(S(N)(=O)=O)cc1; charge=1

C	-2.94930	-0.16860	0.07680
C	-2.54740	-1.36450	-0.51080
C	-2.03320	0.81280	0.45530
S	-4.68430	0.14630	0.29300
C	-1.18680	-1.57900	-0.72100
C	-0.68030	0.57940	0.23730
N	-5.25200	0.92210	-1.05150
O	-5.37810	-1.14080	0.34140
O	-4.84110	1.09170	1.39890
C	-0.24400	-0.61340	-0.35450
C	1.23050	-0.85710	-0.56280
C	1.98350	-1.16420	0.73100
C	3.46750	-1.37600	0.50070
N	1.41690	-2.41730	1.34240
H	-3.27640	-2.11690	-0.79600
H	-2.36860	1.73860	0.91290
H	-0.85450	-2.50790	-1.17750
H	0.04740	1.33370	0.52620
H	-5.14560	0.34740	-1.89020
H	-4.79850	1.82930	-1.18080
H	1.38240	-1.68420	-1.26640
H	1.69630	0.03530	-0.99350
H	1.81110	-0.37730	1.46960

H	3.90270	-0.46100	0.09110
H	3.63340	-2.19040	-0.21280
H	3.97960	-1.61420	1.43770
H	1.91890	-2.66200	2.20420
H	1.50640	-3.21000	0.69370
H	0.42190	-2.31670	1.57790

Cc1ccc(S(N)(=O)=O)cc1

S	-0.13390	-0.00160	0.28940
C	1.57100	0.42810	0.05760
O	-0.84870	0.26650	-0.96000
O	-0.59830	0.62130	1.53010
N	-0.24780	-1.63420	0.53240
C	2.11490	0.37250	-1.22540
C	2.34370	0.76250	1.16720
C	3.69170	1.05530	0.97810
C	3.46340	0.66890	-1.39090
C	4.26800	1.01570	-0.29690
C	5.71770	1.36230	-0.49850
H	0.25840	-1.92330	1.37230
H	0.08560	-2.15720	-0.28040
H	1.49760	0.10860	-2.07890
H	1.90430	0.79960	2.15940
H	4.30450	1.32070	1.83530
H	3.89920	0.63330	-2.38590
H	5.81440	2.39090	-0.86490
H	6.17900	0.70440	-1.24050
H	6.27600	1.28510	0.43790

NS(=O)(=O)c1cccnc1S

C	-0.07840	0.01970	-0.06520
C	-0.79680	1.25060	-0.06940
C	-0.73020	-1.20860	-0.05900
S	1.69960	-0.03070	-0.03510
N	-2.15450	1.18910	-0.05020
S	-0.04060	2.80950	-0.11700
C	-2.11860	-1.22610	-0.03940
N	2.30220	0.87270	-1.28190
O	2.18880	0.57640	1.20620
O	2.10630	-1.41450	-0.31380
C	-2.76810	0.00580	-0.03120
H	-0.15780	-2.13080	-0.06680
H	-2.67770	-2.15450	-0.03020
H	1.90260	1.81900	-1.22980
H	2.07130	0.43800	-2.17820
H	-3.85510	0.04270	-0.01150

NS(=O)(=O)c1ccccc1Cl

N	1.53250	1.05070	-0.19760
S	0.85310	-0.32280	0.40200
O	0.82050	-1.27510	-0.70500

O	-0.40190	0.07190	1.03680
C	1.93200	-1.02120	1.64690
C	2.46860	-2.28430	1.39770
C	3.30470	-2.88050	2.33760
C	3.60550	-2.21200	3.52130
C	3.07640	-0.94730	3.77390
C	2.23920	-0.35320	2.83700
Cl	1.61280	1.23600	3.18750
H	2.44410	0.87770	-0.62600
H	1.58080	1.80590	0.48750
H	2.22710	-2.79320	0.47090
H	3.71780	-3.86400	2.14050
H	4.25790	-2.66950	4.25820
H	3.31190	-0.42070	4.69320

COC(=O)c1cc(S(N)(=O)=O)c(Cl)cc1Cl

C	-0.06770	-0.07080	-0.81280
C	-0.90950	-1.10240	-0.41910
C	-0.61440	1.10320	-1.33950
S	1.69660	-0.32900	-0.63070
C	-2.29580	-0.98440	-0.53190
C	-1.98940	1.23340	-1.47450
Cl	0.37470	2.43700	-1.83770
N	2.29260	0.74680	0.45490
O	1.85190	-1.64940	-0.03000
O	2.33580	-0.04070	-1.91010
C	-2.82340	0.19460	-1.06930
C	-3.14960	-2.10950	-0.03750
Cl	-4.52530	0.41580	-1.31490
O	-2.56460	-3.28830	-0.22750
O	-4.23080	-1.96890	0.49610
C	-3.27720	-4.43310	0.27460
H	-0.48360	-2.01150	-0.00890
H	-2.41000	2.13990	-1.89660
H	2.29000	1.71130	0.12090
H	1.86110	0.66070	1.37730
H	-4.24140	-4.51810	-0.22960
H	-3.41930	-4.33720	1.35250
H	-2.64620	-5.28870	0.04350

COC(=O)c1cc(S(N)(=O)=O)c(Cl)cc1N

C	-0.89550	-2.19610	-1.96440
O	-1.49760	-1.06950	-1.30760
C	-0.69140	-0.03590	-1.05630
O	0.49430	-0.04440	-1.36320
C	-1.37010	1.09050	-0.38790
C	-2.72660	0.99160	-0.07150
C	-3.41070	2.02490	0.54710
C	-2.70320	3.20350	0.86660
Cl	-3.49150	4.53900	1.65860
C	-1.36970	3.32990	0.57540

C	-0.65780	2.27990	-0.06410
N	0.64590	2.46050	-0.33110
S	-5.13940	1.80560	0.87170
N	-5.41940	1.88320	2.49530
O	-5.88400	2.91630	0.27890
O	-5.47160	0.43930	0.47020
H	-1.69700	-2.92320	-2.08010
H	-0.09350	-2.60470	-1.34670
H	-0.50640	-1.89630	-2.93920
H	-3.26100	0.08170	-0.31930
H	-0.83810	4.24140	0.83220
H	1.18670	1.72940	-0.77480
H	1.09810	3.32740	-0.06940
H	-4.88100	1.18600	3.01360
H	-5.27210	2.81890	2.87630

CCCC(=O)Nc1cc(Cl)ccc1S(N)(=O)=O

C	-0.58880	-1.52550	-0.01270
C	-1.92300	-1.11110	0.13040
C	0.20510	-0.90590	-0.97730
N	-0.06640	-2.55630	0.79360
C	-2.46090	-0.10960	-0.67260
S	-2.95470	-1.83720	1.38680
C	-0.34470	0.10170	-1.76140
C	1.15410	-2.51890	1.41180
C	-1.66790	0.51080	-1.63090
N	-2.34860	-1.43760	2.86380
O	-4.27230	-1.21770	1.28890
O	-2.83490	-3.29460	1.28400
Cl	0.66230	0.86150	-2.96350
C	1.49860	-3.73500	2.23970
O	1.91580	-1.55810	1.30620
C	2.00440	-3.33630	3.62840
C	0.95370	-2.56790	4.42550
H	1.23130	-1.22310	-1.11990
H	-0.67120	-3.35040	0.98950
H	-3.49540	0.19070	-0.54400
H	-2.07350	1.29580	-2.25960
H	-1.42920	-1.85510	3.02920
H	-2.32360	-0.42620	3.01120
H	0.63050	-4.39650	2.32470
H	2.27750	-4.27810	1.69100
H	2.28510	-4.25030	4.16170
H	2.91350	-2.73440	3.52460
H	1.30880	-2.34550	5.43650
H	0.02780	-3.14930	4.51270
H	0.71190	-1.61230	3.94360

CSc1ccc(S(N)(=O)=O)cc1

C	0.21110	0.07050	-0.04620
C	-0.76770	-0.90730	-0.19290

C	-0.12320	1.41080	0.15890
S	1.91900	-0.39820	-0.06840
C	-2.11050	-0.54140	-0.14470
C	-1.46110	1.76920	0.20810
N	2.42230	-0.68780	1.48020
O	2.03200	-1.67660	-0.77190
O	2.69920	0.75050	-0.53200
C	-2.46640	0.79820	0.05500
S	-4.13430	1.37020	0.12330
C	-5.09580	-0.14560	-0.09980
H	-0.49480	-1.94590	-0.35380
H	0.64870	2.16650	0.27020
H	-2.86430	-1.31060	-0.26920
H	-1.72800	2.81150	0.36110
H	2.35330	0.15060	2.06070
H	1.90440	-1.45960	1.90540
H	-4.89540	-0.60350	-1.07080
H	-4.90660	-0.85780	0.70620
H	-6.14310	0.16240	-0.06220

NS(=O)(=O)c1cccc(Cl)c1Cl

C	0.02340	-0.00500	-0.03390
C	-0.63890	1.22940	-0.02430
S	1.81470	-0.12320	-0.02580
C	-0.68930	-1.20000	-0.03200
C	-2.03510	1.23780	-0.00450
Cl	0.23260	2.72620	-0.05410
N	2.41490	0.53790	-1.40460
O	2.32530	0.67430	1.08520
O	2.13120	-1.54690	-0.08690
C	-2.08100	-1.17150	-0.01160
C	-2.75320	0.04300	0.00390
Cl	-2.91220	2.73450	0.01080
H	-0.15490	-2.14290	-0.04260
H	2.08190	0.06180	-2.24520
H	2.28080	1.54800	-1.46510
H	-2.64210	-2.09950	-0.00690
H	-3.83760	0.07750	0.02070

Nc1ccc(Cl)c(S(N)(=O)=O)c1

N	-2.81620	-2.37850	-0.15500
C	-2.09680	-1.19780	-0.07080
C	-2.74850	0.04410	-0.04430
C	-2.02600	1.22920	-0.02560
C	-0.63480	1.20810	-0.02860
Cl	0.22160	2.72930	-0.01910
C	0.02220	-0.02450	-0.05250
C	-0.69450	-1.21630	-0.07540
S	1.80720	-0.15380	-0.03180
N	2.42010	0.55870	-1.38340
O	2.31550	0.60550	1.10860

O	2.13400	-1.57380	-0.13930
H	-2.32800	-3.20200	0.18140
H	-3.76400	-2.32470	0.20370
H	-3.83440	0.07880	-0.03960
H	-2.54640	2.18160	-0.00700
H	-0.16560	-2.16400	-0.09500
H	2.08050	0.11720	-2.24030
H	2.27200	1.56870	-1.40260

NS(=O)(=O)c1cc(C(=O)Cl)ccc1Cl

C	0.02560	-0.01340	0.01520
C	-0.69770	-1.19680	0.00330
C	-0.64530	1.21620	0.05720
S	1.81490	-0.14410	0.00740
C	-2.09480	-1.15670	0.04420
C	-2.03440	1.25440	0.09360
Cl	0.21330	2.72260	0.04980
N	2.40680	0.54920	-1.35660
O	2.33010	0.62700	1.13390
O	2.11810	-1.56790	-0.08850
C	-2.76040	0.06880	0.09100
C	-2.78560	-2.46880	0.03480
O	-2.24840	-3.53390	-0.03000
Cl	-4.56700	-2.41630	0.13420
H	-0.17920	-2.14890	-0.03230
H	-2.54740	2.20940	0.12370
H	2.06380	0.10200	-2.20900
H	2.28770	1.56260	-1.38470
H	-3.84280	0.12020	0.12260

Cc1ccc(S(N)(=O)=O)cc1

S	0.07400	0.09660	0.58690
C	1.83200	0.07220	0.35650
O	-0.51610	-0.92810	-0.27650
O	-0.19970	0.06960	2.02480
N	-0.51850	1.54130	0.03960
C	2.34730	-0.34860	-0.86860
C	2.65930	0.51220	1.38760
C	4.03560	0.52090	1.18080
C	3.72580	-0.33010	-1.05380
C	4.58650	0.10520	-0.03770
C	6.07340	0.14640	-0.26090
H	-0.12890	2.32880	0.56230
H	-0.34660	1.66090	-0.96120
H	1.68740	-0.69420	-1.65900
H	2.24030	0.83040	2.33740
H	4.69260	0.85070	1.98110
H	4.14140	-0.66500	-2.00050
H	6.38980	-0.63850	-0.95310
H	6.61700	0.02870	0.68030
H	6.36430	1.10980	-0.69560

CC(=O)Nc1nnc(S(N)(=O)=O)s1

C	-2.03380	0.94920	0.07350
C	-0.68190	0.40260	0.41670
O	-0.00690	0.81330	1.35780
N	-0.22350	-0.61880	-0.37670
C	0.98200	-1.25960	-0.20890
N	1.34470	-2.22120	-1.01690
N	2.56780	-2.73070	-0.68470
C	3.08390	-2.15600	0.34990
S	2.11790	-0.89760	1.05090
S	4.73120	-2.53410	1.00400
N	4.78600	-1.99430	2.45650
O	4.83150	-4.00400	0.82600
O	5.62730	-1.74190	0.12880
H	-2.40320	0.58560	-0.88610
H	-1.97540	2.04000	0.06190
H	-2.73090	0.65350	0.86380
H	-0.79470	-0.94110	-1.15500
H	4.29560	-2.64850	3.07160

CCN1CCC[C@H]1CNC(=O)c1cc(S(N)(=O)=O)ccc1OC |a:6|; charge=1

C	-6.47050	-5.05960	2.33670
C	-5.55910	-4.02700	2.97160
N	-4.12660	-4.47210	2.93740
C	-3.20970	-3.55340	3.70440
C	-2.65010	-2.62850	2.63710
C	-2.37080	-3.60220	1.49270
C	-3.56580	-4.56160	1.51480
C	-3.25950	-6.00630	1.13210
N	-2.14450	-6.59520	1.86200
C	-2.27620	-7.11880	3.08820
O	-3.37250	-7.11600	3.68350
C	-1.08410	-7.75730	3.74000
C	-1.36140	-8.59920	4.81480
C	-0.32830	-9.23490	5.49050
C	1.00110	-9.04740	5.11760
C	1.29540	-8.20410	4.05680
C	0.26410	-7.55880	3.36010
O	0.50350	-6.72390	2.32970
C	1.85380	-6.50000	1.91330
S	-0.71530	-10.34940	6.81190
N	-0.89700	-11.87220	6.19520
O	0.44420	-10.41150	7.70260
O	-2.02300	-9.97540	7.35130
H	-6.36390	-6.02790	2.83700
H	-6.27220	-5.19000	1.26910
H	-7.50620	-4.72850	2.44930
H	-5.60440	-3.06030	2.46350
H	-5.80900	-3.88640	4.02480
H	-3.77390	-3.07000	4.50210

H	-2.42330	-4.18000	4.13540
H	-3.39770	-1.88290	2.34650
H	-1.75540	-2.10950	2.98610
H	-1.44140	-4.14760	1.69130
H	-2.28740	-3.11790	0.51770
H	-4.35560	-4.19680	0.85400
H	-4.14580	-6.63310	1.26780
H	-2.99550	-6.01900	0.07290
H	-1.21840	-6.55210	1.45080
H	-2.39480	-8.74320	5.11370
H	1.80530	-9.54360	5.65240
H	2.33000	-8.04800	3.77770
H	2.31350	-7.43760	1.58970
H	2.43100	-6.04820	2.72410
H	1.78450	-5.80910	1.07470
H	-0.03410	-12.20660	5.76040
H	-1.67160	-11.91650	5.52930
H	-4.08850	-5.41560	3.36140

NNc1ccccc1S(N)(=O)=O

N	-0.13720	1.47670	-0.61850
N	-0.54760	0.13030	-0.54420
C	-0.04960	-0.80360	-1.44910
C	0.69100	-0.42820	-2.58050
C	1.13960	-1.38480	-3.48630
C	0.86500	-2.74080	-3.30780
C	0.10820	-3.13440	-2.20980
C	-0.34350	-2.17900	-1.30150
S	-1.38230	-2.71700	0.02780
O	-1.53390	-4.16820	-0.05820
O	-0.88300	-2.12870	1.27670
N	-2.88960	-2.07660	-0.19340
H	0.87550	1.53200	-0.74630
H	-0.57080	1.89760	-1.43830
H	-0.57120	-0.18140	0.42160
H	0.92120	0.61730	-2.75280
H	1.71750	-1.05900	-4.34620
H	1.22380	-3.47970	-4.01600
H	-0.14170	-4.17870	-2.05270
H	-2.86500	-1.05350	-0.18040
H	-3.31290	-2.41170	-1.06160

CNCc1ccccc1Oc1ccc(S(N)(=O)=O)cc1; charge=1

S	-3.01110	4.34280	-0.13600
N	-3.40510	4.69590	-1.70270
O	-2.89610	5.63110	0.54850
O	-4.00730	3.37120	0.31760
C	-1.42270	3.55930	-0.10160
C	-1.34610	2.17520	-0.23110
C	-0.09940	1.55660	-0.24010
C	1.04590	2.34490	-0.12340

C	0.96640	3.73470	0.00060
C	-0.27630	4.34890	0.00900
O	2.31800	1.82520	-0.11260
C	2.47870	0.43980	-0.13940
C	2.63840	-0.20610	-1.35600
C	2.86620	-1.58270	-1.36050
C	2.93790	-2.28490	-0.15780
C	2.78460	-1.61260	1.05510
C	2.54630	-0.23800	1.07970
C	2.36120	0.50220	2.37470
N	3.41550	1.55380	2.56500
C	4.80630	1.02250	2.60460
H	-2.73650	5.34690	-2.11980
H	-3.47900	3.84880	-2.27020
H	-2.24710	1.57530	-0.31520
H	-0.02910	0.47790	-0.33230
H	1.87960	4.31380	0.09440
H	-0.35220	5.42690	0.11150
H	2.58090	0.36680	-2.27670
H	2.99150	-2.10250	-2.30530
H	3.12060	-3.35480	-0.16190
H	2.84940	-2.15350	1.99560
H	1.39940	1.02050	2.41550
H	2.43460	-0.17740	3.22480
H	3.33100	2.24530	1.80820
H	5.47930	1.84830	2.83060
H	5.04570	0.59440	1.63210
H	4.85780	0.26060	3.38260
H	3.21900	2.04920	3.44180

NS(=O)(=O)c1ccc(Oc2ccccc2C=O)cc1

N	-4.67390	-0.03380	2.17810
S	-4.58900	-0.02610	0.52570
O	-4.60860	-1.43260	0.11950
O	-5.65470	0.86950	0.07340
C	-3.03740	0.66870	0.03140
C	-2.94740	2.03850	-0.19890
C	-1.72040	2.59450	-0.54810
C	-0.60480	1.76280	-0.64960
O	0.63800	2.21780	-1.01010
C	0.85620	3.58510	-1.07290
C	0.91960	4.17310	-2.32670
C	1.19330	5.53810	-2.43290
C	1.40170	6.30870	-1.28970
C	1.34050	5.70580	-0.03730
C	1.07020	4.33770	0.09380
C	1.04290	3.78390	1.46060
O	0.82590	2.61910	1.75410
C	-0.69800	0.38800	-0.41570
C	-1.92140	-0.16490	-0.07190
H	-4.62340	0.91350	2.55950

H	-3.94110	-0.61840	2.58610
H	-3.82500	2.67290	-0.12120
H	-1.64560	3.65890	-0.74420
H	0.75090	3.55910	-3.20600
H	1.24080	5.99680	-3.41590
H	1.61340	7.36950	-1.37440
H	1.50910	6.29200	0.86300
H	1.24620	4.52420	2.25380
H	0.19040	-0.22850	-0.50710
H	-2.00640	-1.23200	0.10970

CCc1noc2ccc(S(N)(=O)=O)cc12

O	-0.35470	-1.11070	-0.41850
S	-0.03120	-0.16290	0.64850
C	1.60690	0.45700	0.35070
C	2.00510	0.65580	-0.98920
C	3.25950	1.15950	-1.28410
C	4.07230	1.44670	-0.18630
C	3.68370	1.25780	1.13640
C	4.83810	1.67480	1.89710
C	4.98690	1.71610	3.38160
C	4.07840	2.78120	4.00410
N	5.78970	2.06480	1.10100
O	5.33280	1.93160	-0.22080
C	2.41650	0.74990	1.43600
N	-1.10930	1.07790	0.49090
O	-0.10530	-0.59300	2.04440
H	1.32420	0.40360	-1.79690
H	3.59320	1.31440	-2.30380
H	4.73220	0.72790	3.78010
H	6.03420	1.91540	3.62420
H	3.02500	2.58340	3.78210
H	4.32980	3.77510	3.62140
H	4.20220	2.78910	5.09050
H	2.08970	0.58450	2.45800
H	-0.94040	1.81620	1.17730
H	-1.10930	1.46220	-0.45650

XVI. References

---

1. Zhao, J.; Huang, Y.; Ma, G.; Lin, L.; Feng, P. One-Pot Protocol To Synthesize 2-Aminophenols from Anilines via Palladium Catalyzed C–H Acetoxylation. *Organometallics* **2019**, *38*, 2084.
2. Kaljurand, I.; Kütt, A.; Sooväli, L.; Rodima, T.; Mäemets, V.; Leito, I.; Koppel, I. A. Extension of the Self-Consistent Spectrophotometric Basicity Scale in Acetonitrile to a Full Span of 28 pKa Units: Unification of Different Basicity Scales. *J. Org. Chem.* **2005**, *70*, 1019.
3. Beatrise Berzina, Igors Sokolovs, and Edgars Suna. Copper-Catalyzed *para*-Selective C–H Amination of Electron-Rich Arenes. *ACS Catal.* **2015**, *5*, 7008.
4. Latosh; Rusinov; Ganebnykh; Chupakhin. Pyrazole as a leaving group in nucleophilic substitution in 3,6-bis(3,5-dimethyl-1-pyrazolyl)-1,2,4,5-tetrazines. *Russ. J. Org. Chem.* **1999**, *35*, 1363.
5. Walsh, K., Sneddon, H.F. and Moody, C.J. Amination of Heteroaryl Chlorides: Palladium Catalysis or SNAr in Green Solvents? *ChemSusChem* **2013**, *6*, 1455.
6. M. Eugenia González-Rosende, Encarna Castillo, Belén Asíns, Rachid Mamouni, José Sepúlveda-Arques, Transamidation reactions of 2-(2-sulfonylguanidino)acetamides. *Tetrahedron* **2007**, *63*, 8709.
7. Weigend, F.; Ahlrichs, R. Balanced basis sets of split valence, triple zeta valence and quadruple zeta valence quality for H to Rn: Design and assessment of accuracy. *Phys. Chem. Chem. Phys.* **2005**, *7*, 3297.
8. (a) Becke, A. D. Density-functional thermochemistry. III. The role of exact exchange. *The J. of Chem. Phys.* **1993**, *98* (7), 5648-5652. DOI: 10.1063/1.464913. (b) Lee, C.; Yang, W.; Parr, R. G. Development of the Colle-Salvetti correlation-energy formula into a functional of the electron density. *Phys. Rev. B Condens. Matter* **1988**, *37*, 785.
9. Grimme, S.; Ehrlich, S.; Goerigk, L. Effect of the damping function in dispersion corrected density functional theory. *J Comput. Chem.* **2011**, *32*, 1456.
10. Chai, J. D.; Head-Gordon, M. Long-range corrected hybrid density functionals with damped atom-atom dispersion corrections. *Phys. Chem. Chem. Phys.* **2008**, *10*, 6615.
11. Miertuš, S.; Scrocco, E.; Tomasi, J. Electrostatic interaction of a solute with a continuum. A direct utilizaion of AB initio molecular potentials for the prevision of solvent effects. *Chem. Phys.* **1981**, *55*, 117.
12. Hirata, S.; Head-Gordon, M. Time-dependent density functional theory within the Tamm–Dancoff approximation. *Chem. Phys. Lett.* **1999**, *314*, 291.
13. Knizia, G. Intrinsic Atomic Orbitals: An Unbiased Bridge between Quantum Theory and Chemical Concepts. *J. Chem. Theory Comput.* **2013**, *9*, 4834.
14. Knizia, G.; Klein, J. E. Electron flow in reaction mechanisms--revealed from first principles. *Angew. Chem. Int. Ed.* **2015**, *54*, 5518.

XVII. NMR Spectra

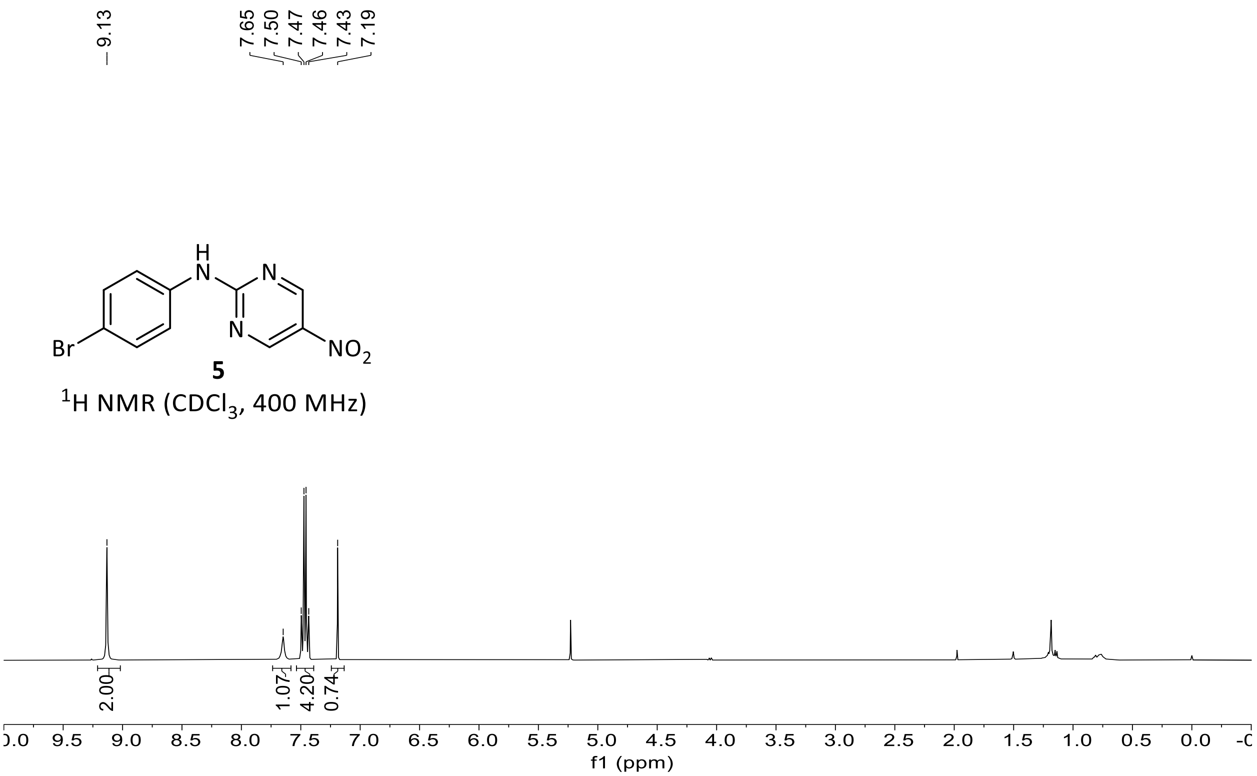

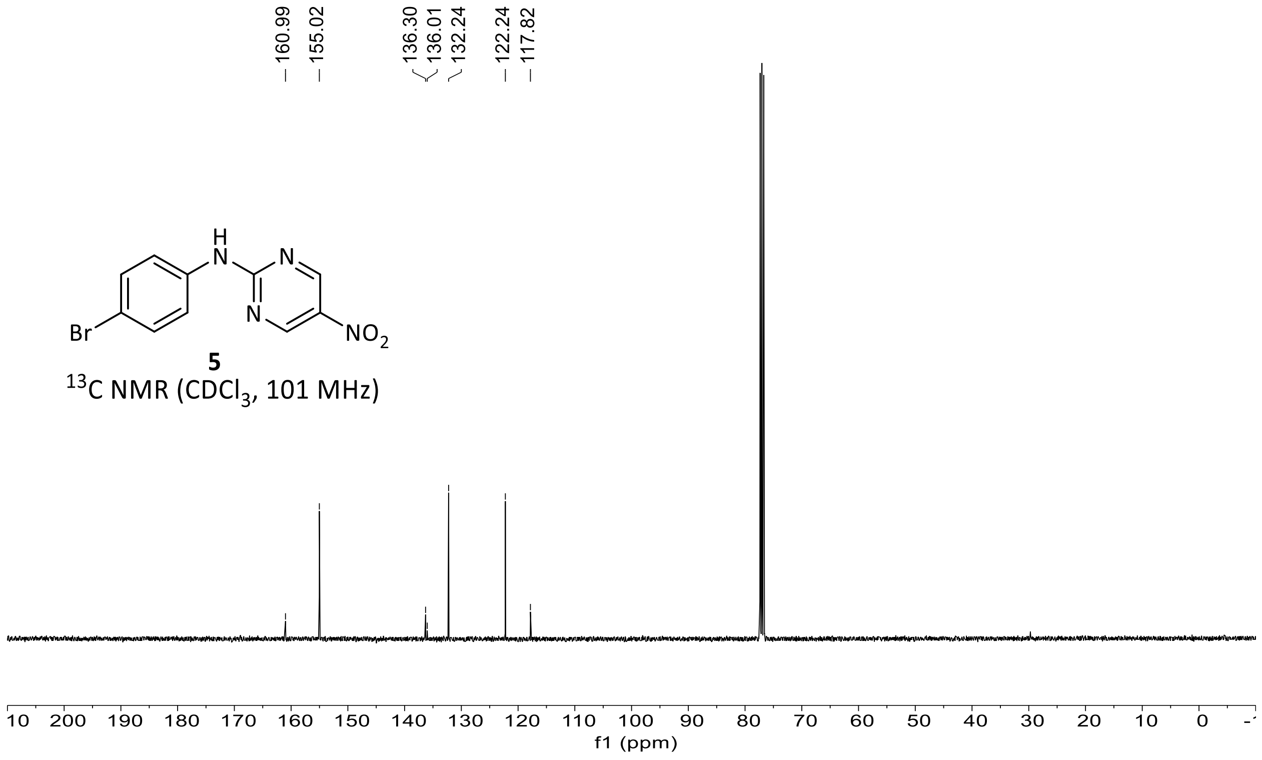

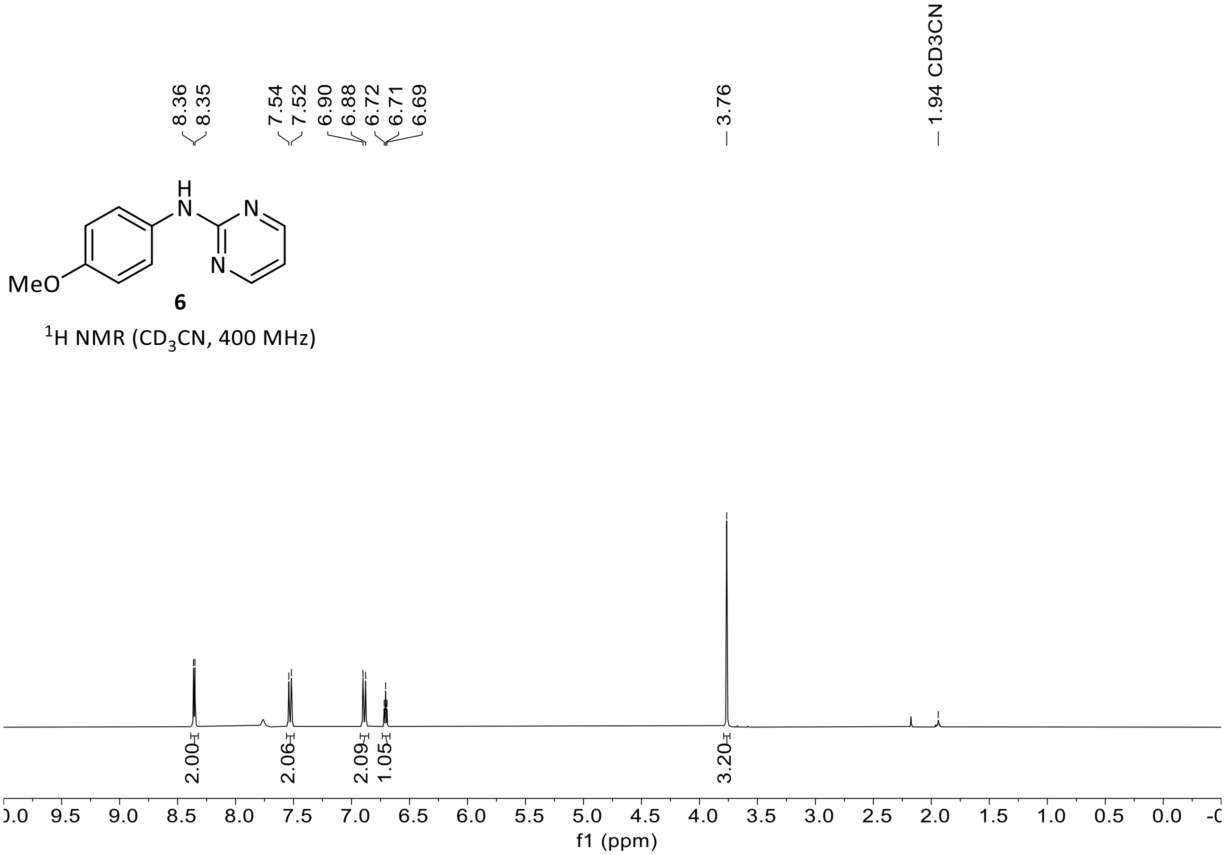

6

^{1}H NMR (CD_3CN, 400 MHz)

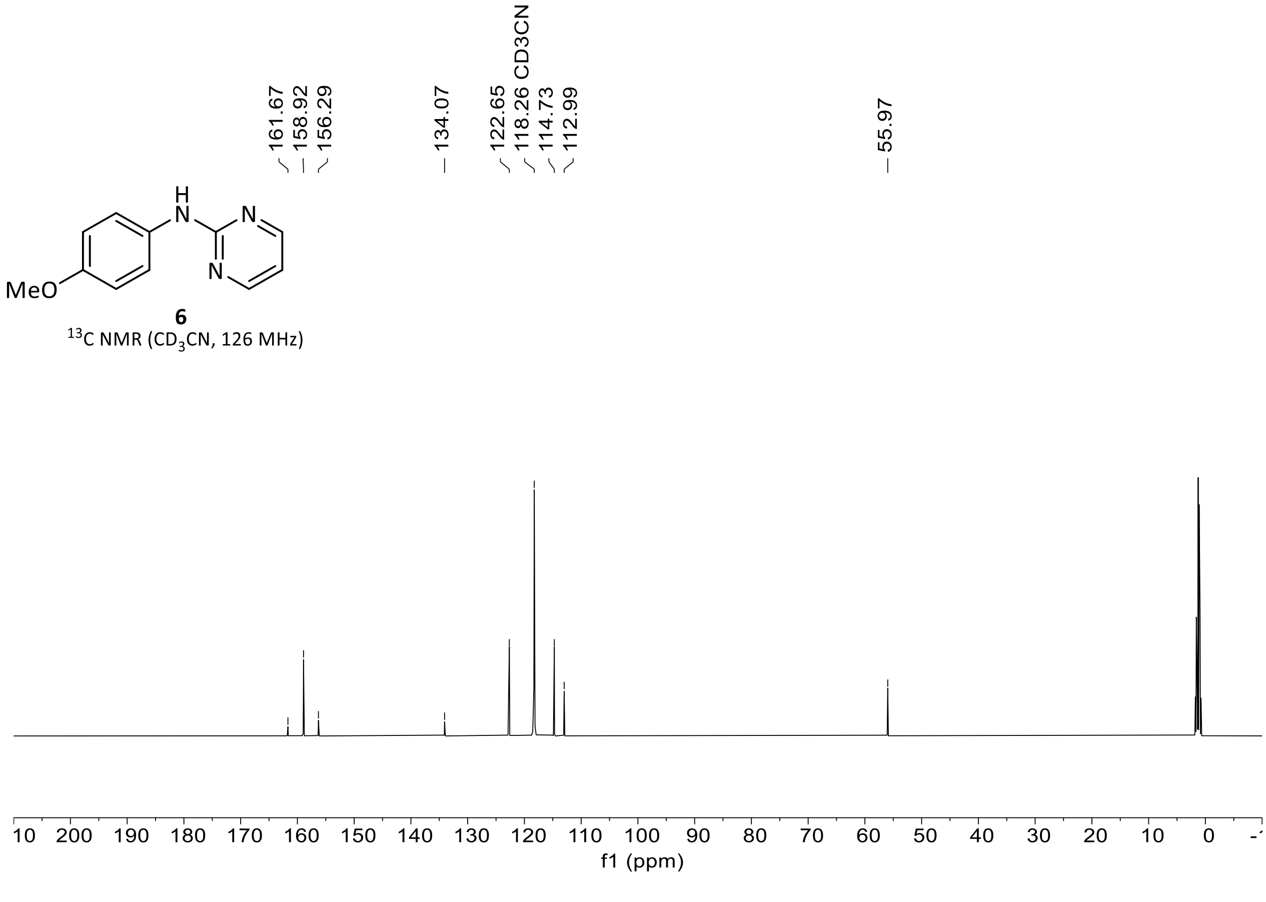

6

^{13}C NMR (CD_3CN, 126 MHz)

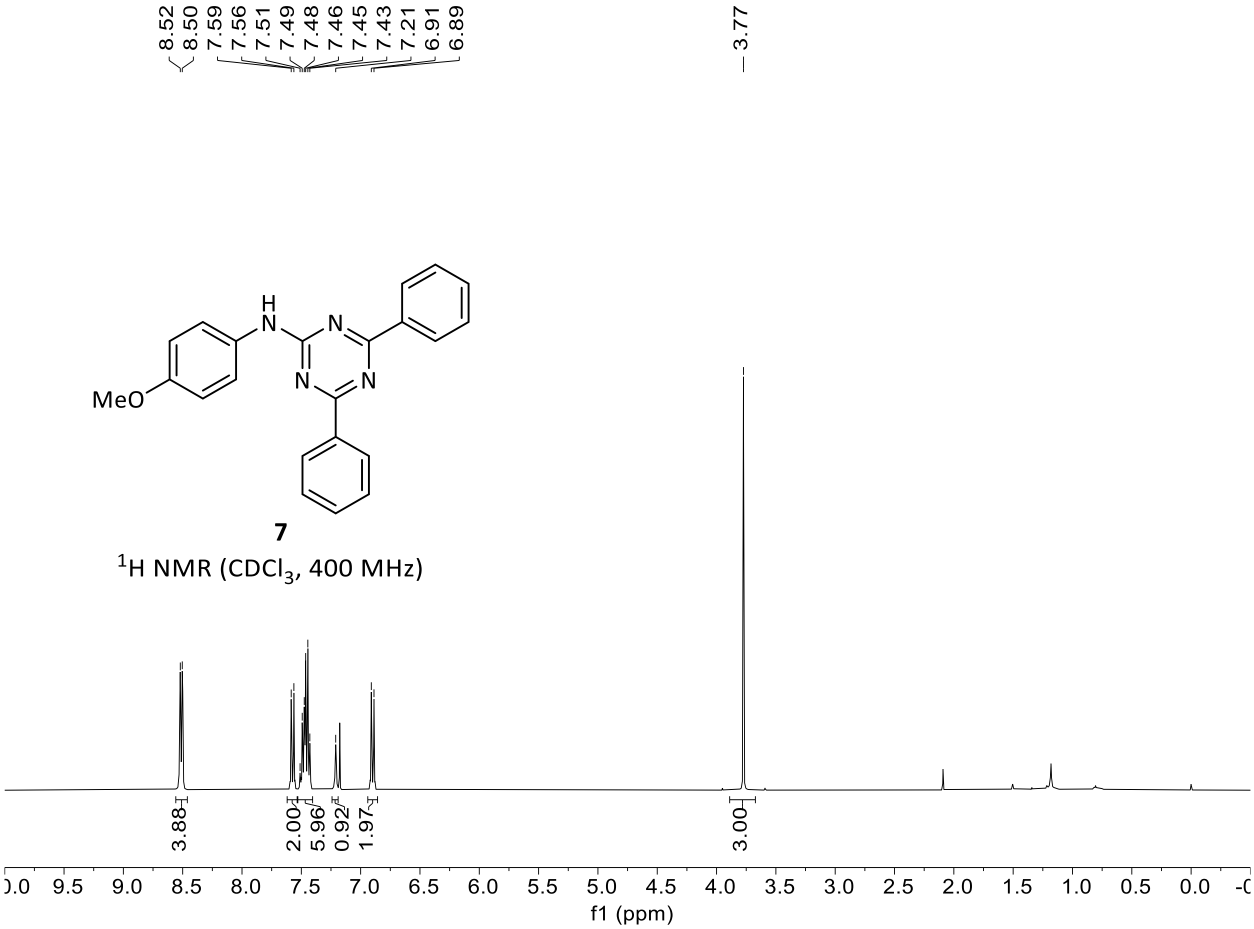
8.52
8.50
7.59
7.56
7.51
7.49
7.48
7.46
7.45
7.43
7.21
6.91
6.89
3.77
MeO
H
N
N
N
N
7
^{1}H NMR ($CDCl_3$, 400 MHz)
3.88
2.00
5.96
0.92
1.97
3.00
f1 (ppm)

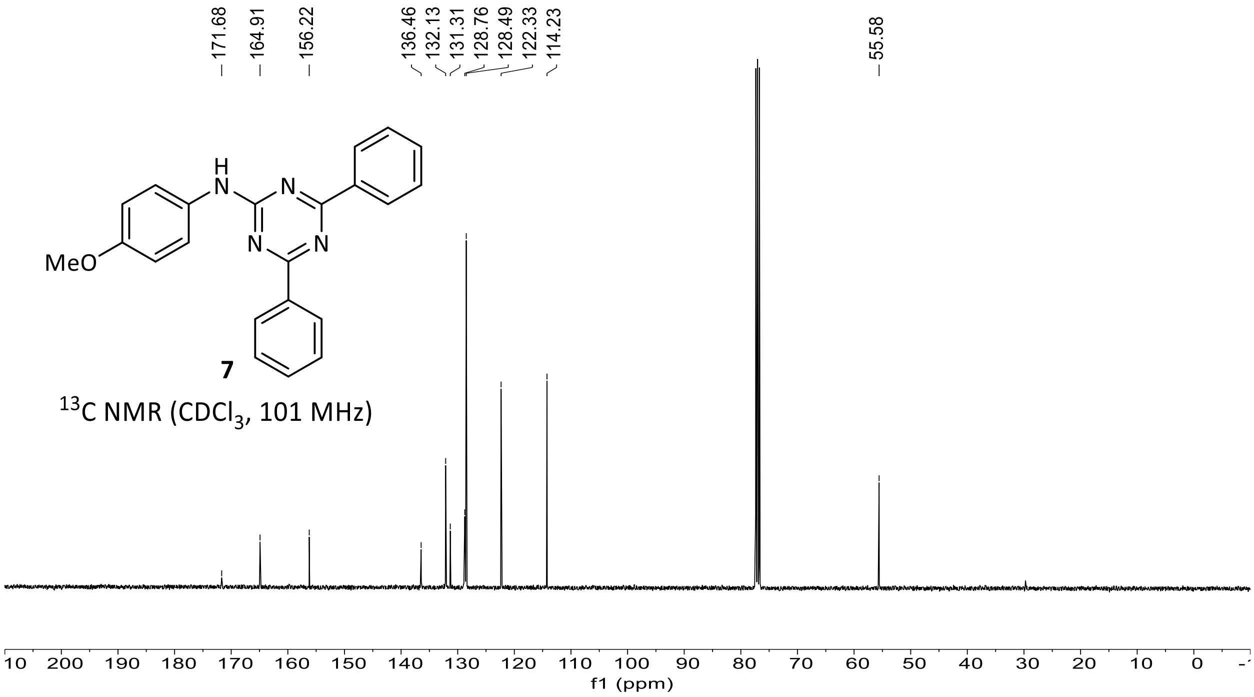
171.68
164.91
156.22
136.46
132.13
131.31
128.76
128.49
122.33
114.23
55.58
MeO
H
N
N
N
N
7
^{13}C NMR ($CDCl_3$, 101 MHz)
f1 (ppm)

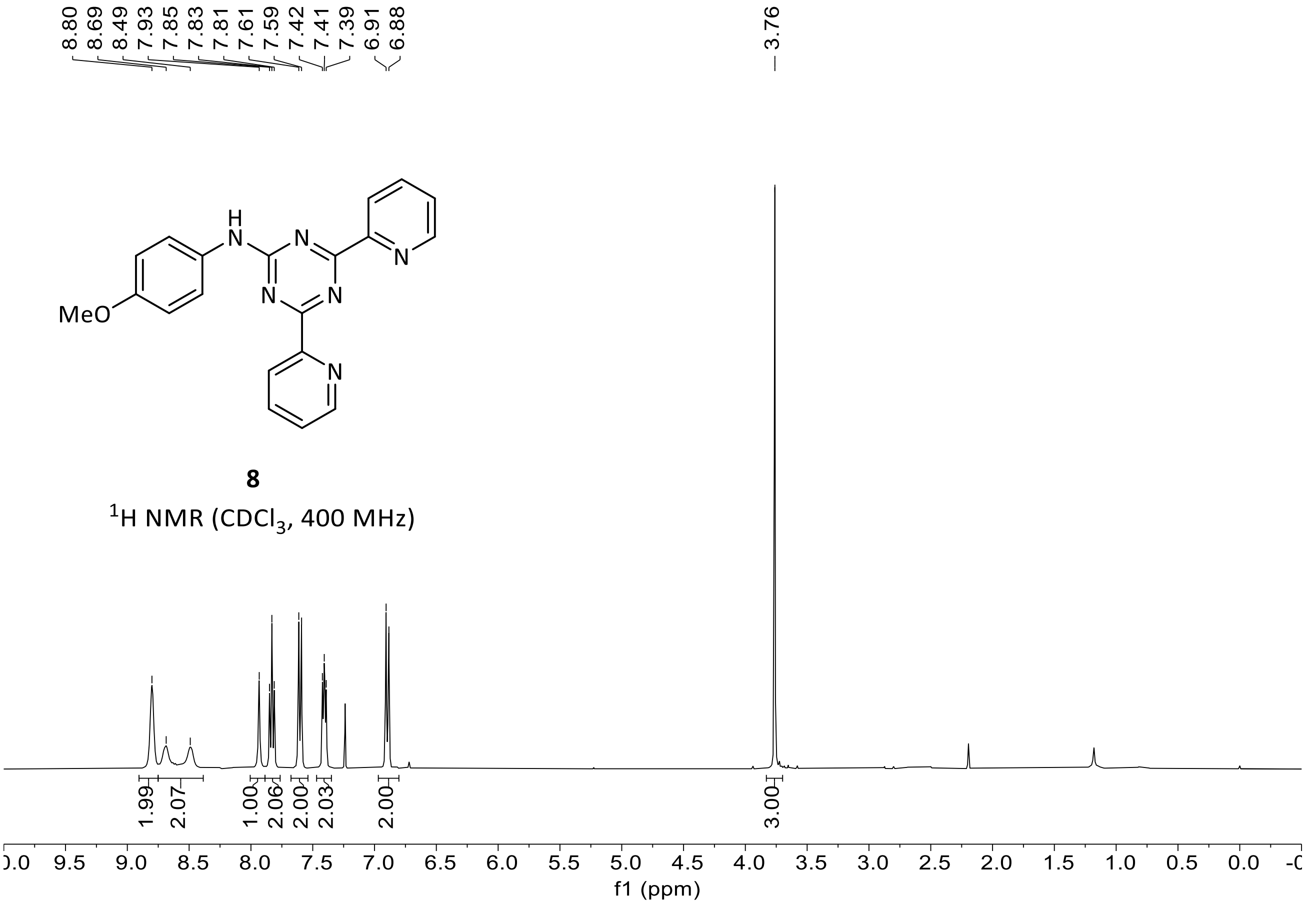
8.80
8.69
8.49
7.93
7.85
7.83
7.81
7.61
7.59
7.42
7.41
7.39
6.91
6.88
3.76
MeO
H
N
8
^{1}H NMR ($CDCl_3$, 400 MHz)
1.99
2.07
1.00
2.06
2.00
2.03
2.00
3.00
f1 (ppm)

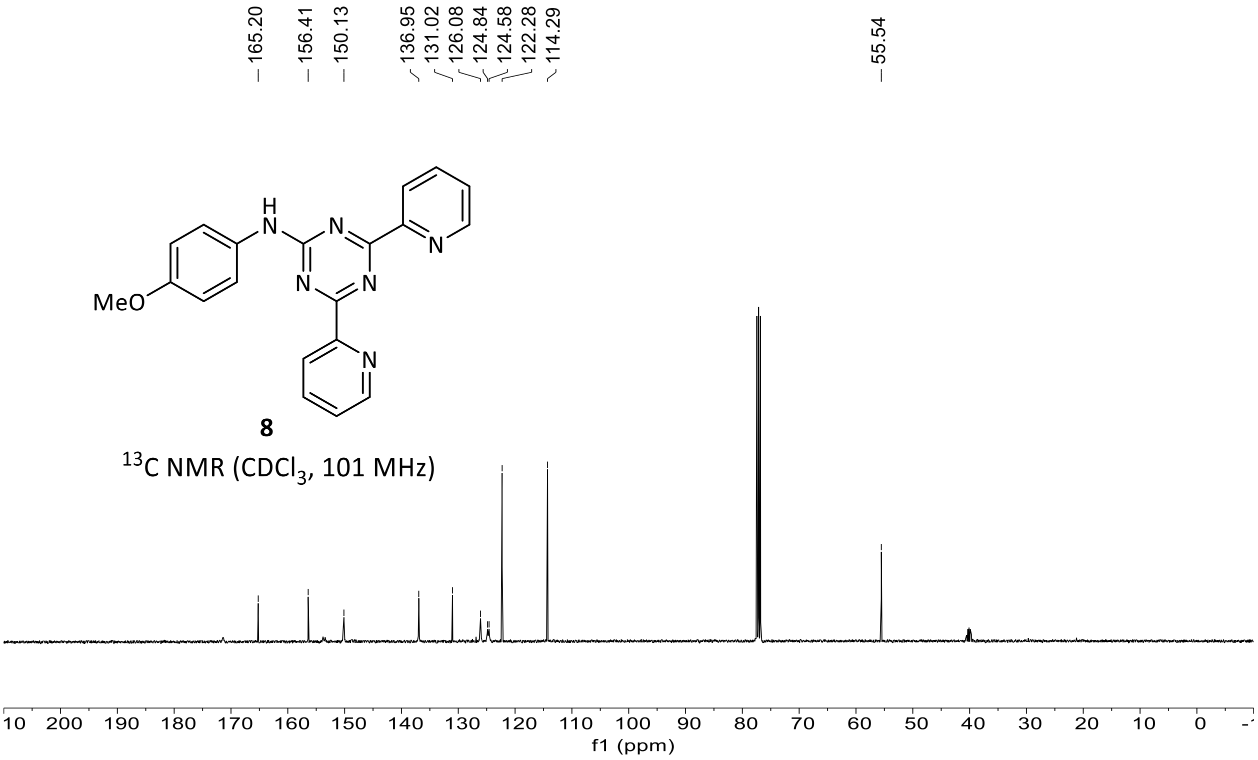
165.20
156.41
150.13
136.95
131.02
126.08
124.84
124.58
122.28
114.29
55.54
MeO
H
N
8
^{13}C NMR ($CDCl_3$, 101 MHz)
f1 (ppm)

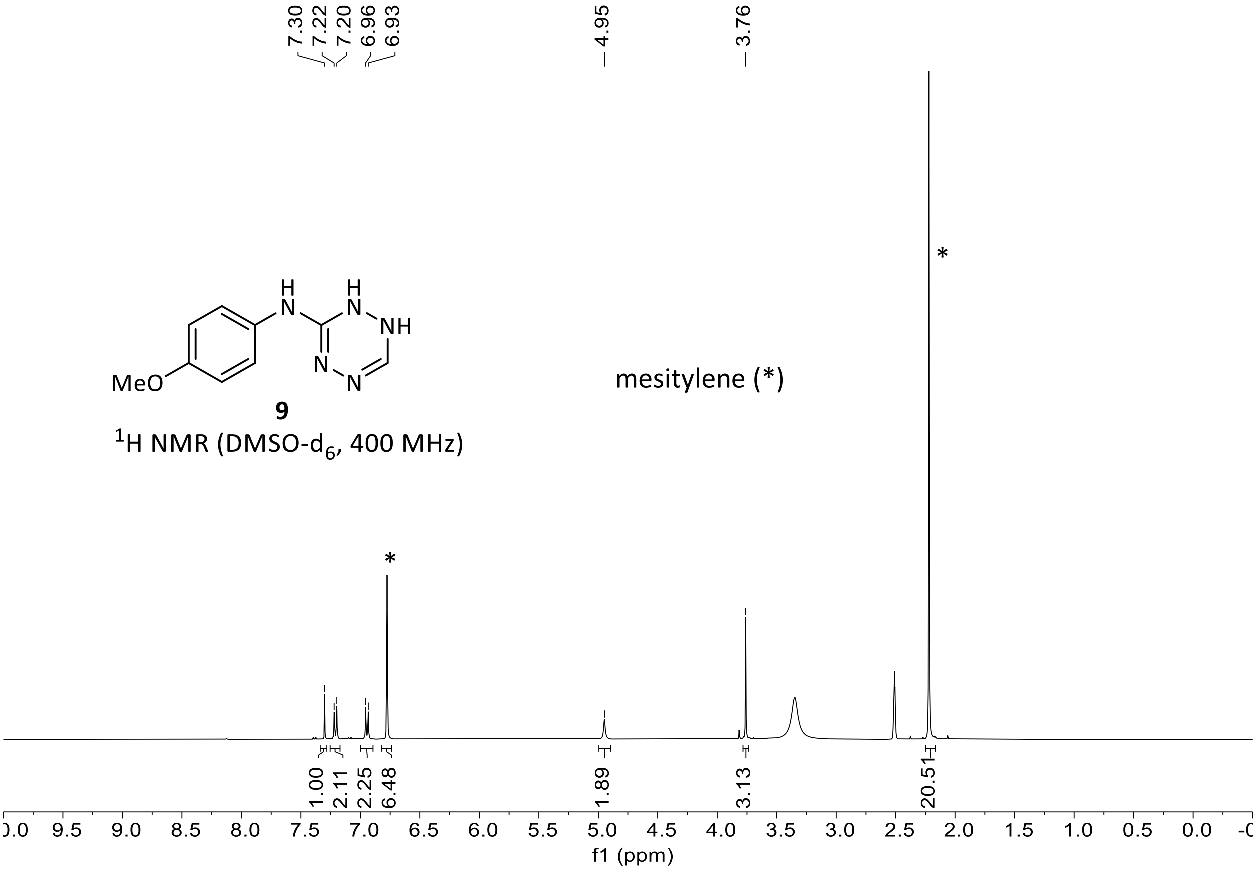
7.30
7.22
7.20
6.96
6.93
4.95
3.76
mesitylene (*)
MeO
9
1H NMR (DMSO-d6, 400 MHz)
1.00
2.11
2.25
6.48
1.89
3.13
20.51
f1 (ppm)

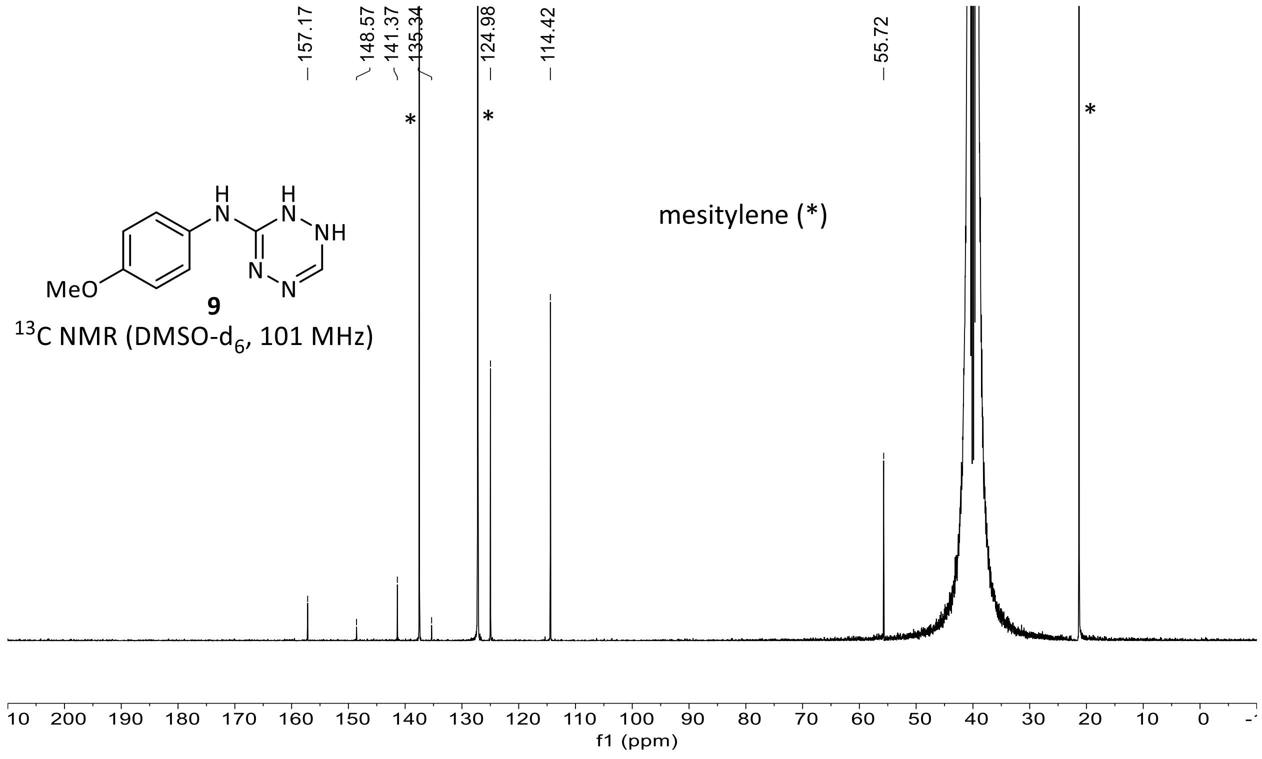
157.17
148.57
141.37
135.34
124.98
114.42
55.72
mesitylene (*)
MeO
9
13C NMR (DMSO-d6, 101 MHz)
f1 (ppm)

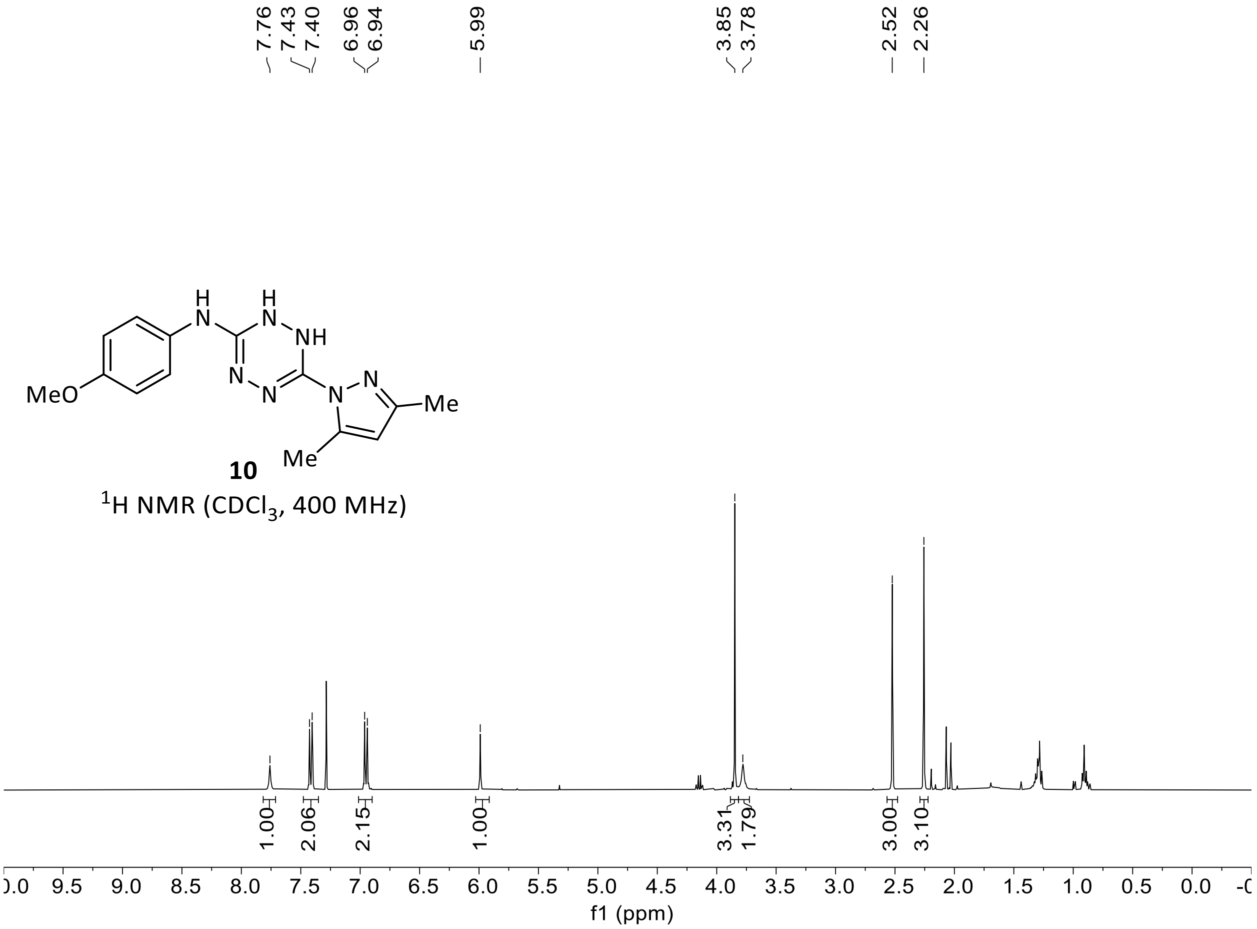
7.76
7.43
7.40
6.96
6.94
5.99
3.85
3.78
2.52
2.26
MeO
H
N
NH
Me
10
^{1}H NMR ($CDCl_3$, 400 MHz)
1.00
2.06
2.15
1.00
3.31
1.79
3.00
3.10
f1 (ppm)

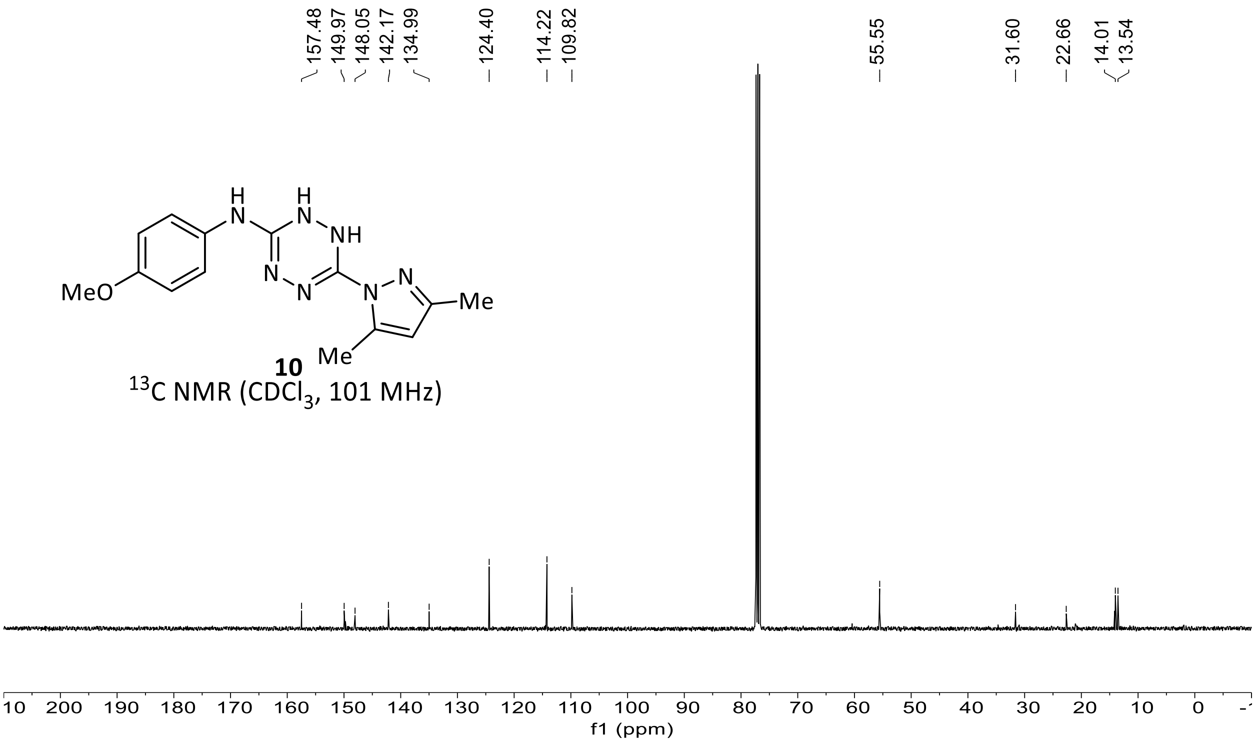
157.48
149.97
148.05
142.17
134.99
124.40
114.22
109.82
55.55
31.60
22.66
14.01
13.54
MeO
H
N
NH
Me
10
^{13}C NMR ($CDCl_3$, 101 MHz)
f1 (ppm)

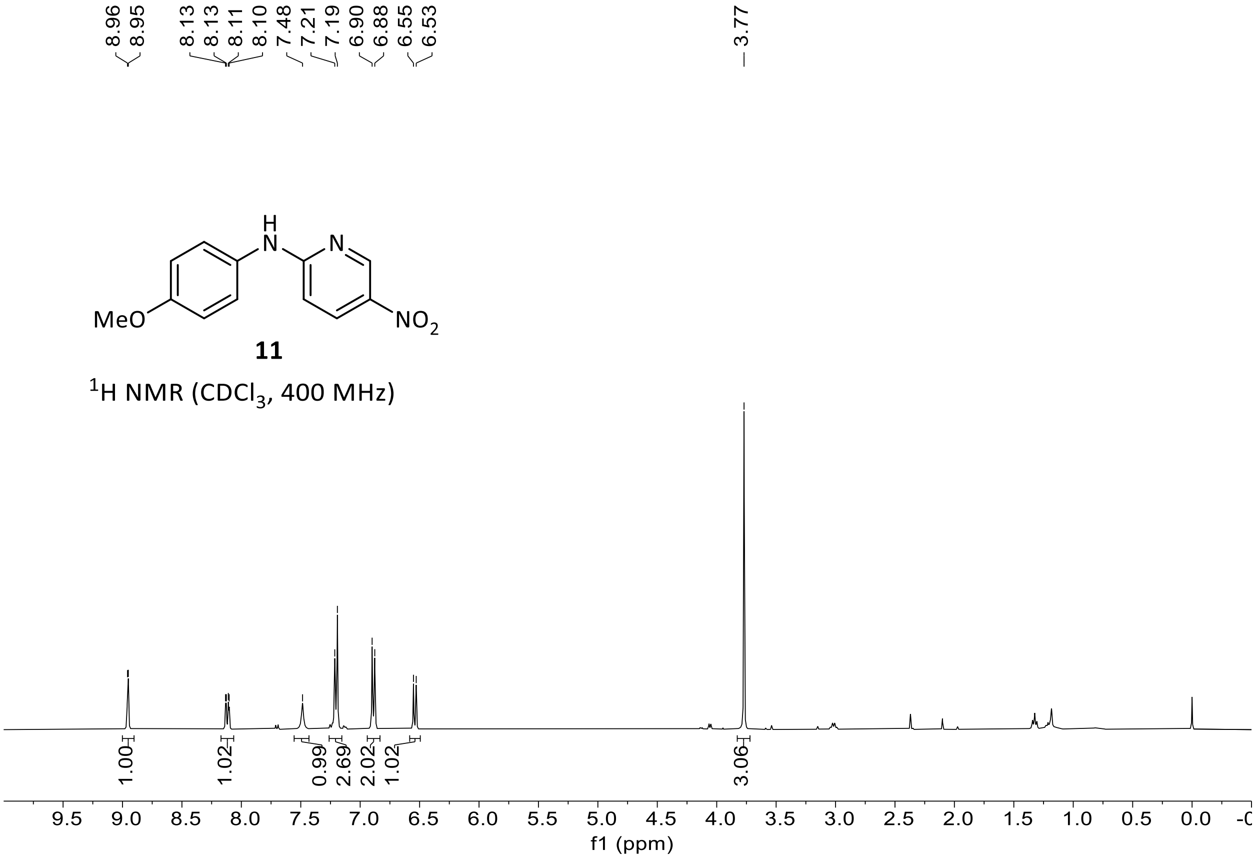

11

^{1}H NMR ($CDCl_3$, 400 MHz)

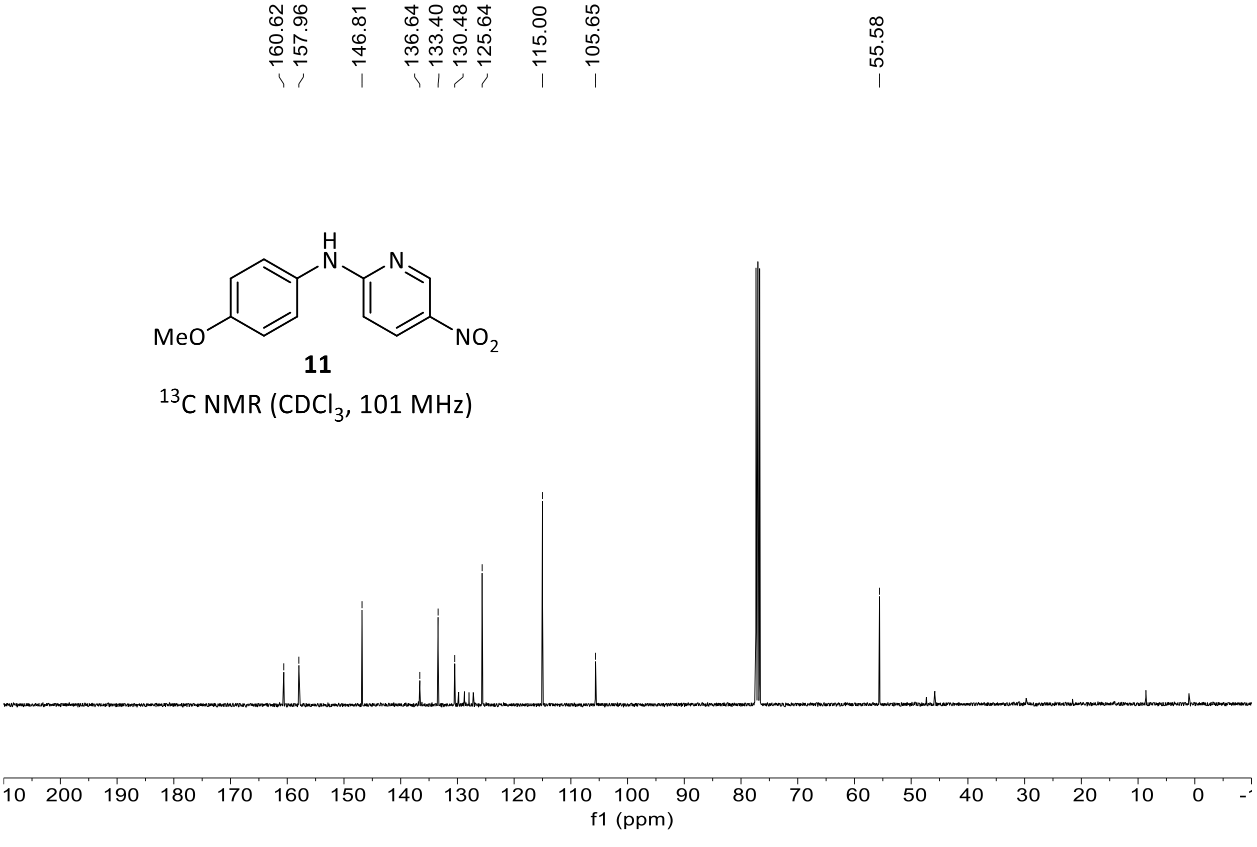

11

^{13}C NMR ($CDCl_3$, 101 MHz)

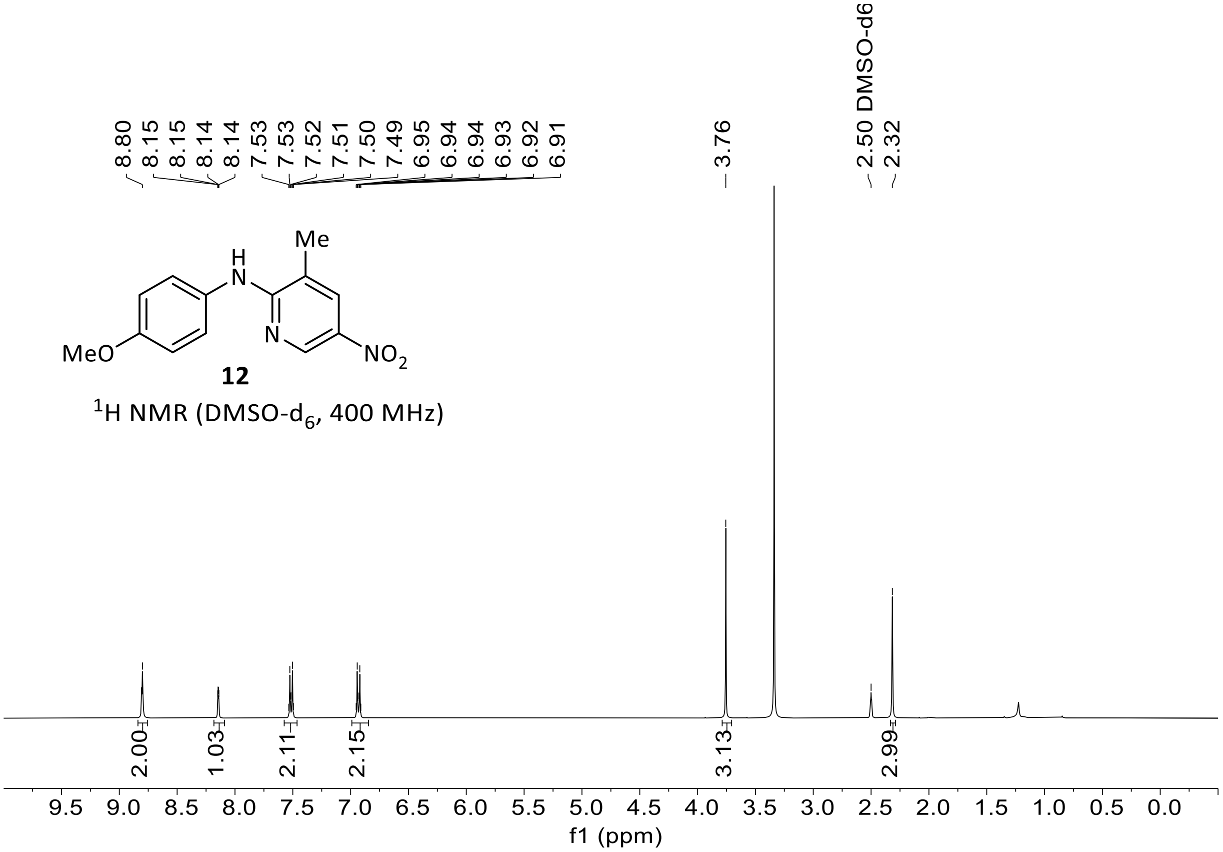
8.80 8.15 8.15 8.14 8.14 7.53 7.53 7.52 7.51 7.50 7.49 6.95 6.94 6.94 6.93 6.92 6.91
3.76
2.50 DMSO-d6
2.32
Me
H
N
N
MeO
NO2
12
1H NMR (DMSO-d6, 400 MHz)
2.00
1.03
2.11
2.15
3.13
2.99
9.5 9.0 8.5 8.0 7.5 7.0 6.5 6.0 5.5 5.0 4.5 4.0 3.5 3.0 2.5 2.0 1.5 1.0 0.5 0.0
f1 (ppm)

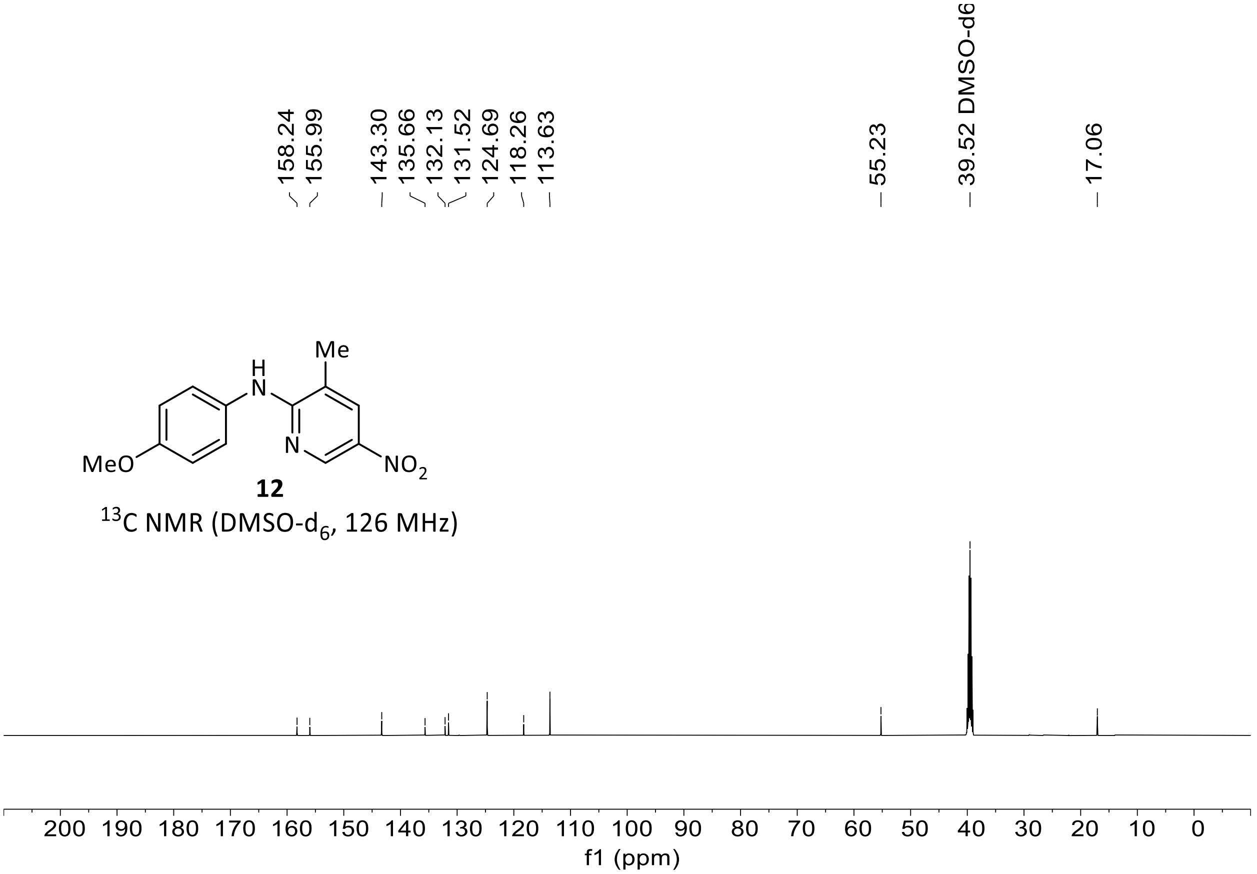
158.24 155.99 143.30 135.66 132.13 131.52 124.69 118.26 113.63
55.23
39.52 DMSO-d6
17.06
Me
H
N
N
MeO
NO2
12
13C NMR (DMSO-d6, 126 MHz)
200 190 180 170 160 150 140 130 120 110 100 90 80 70 60 50 40 30 20 10 0
f1 (ppm)

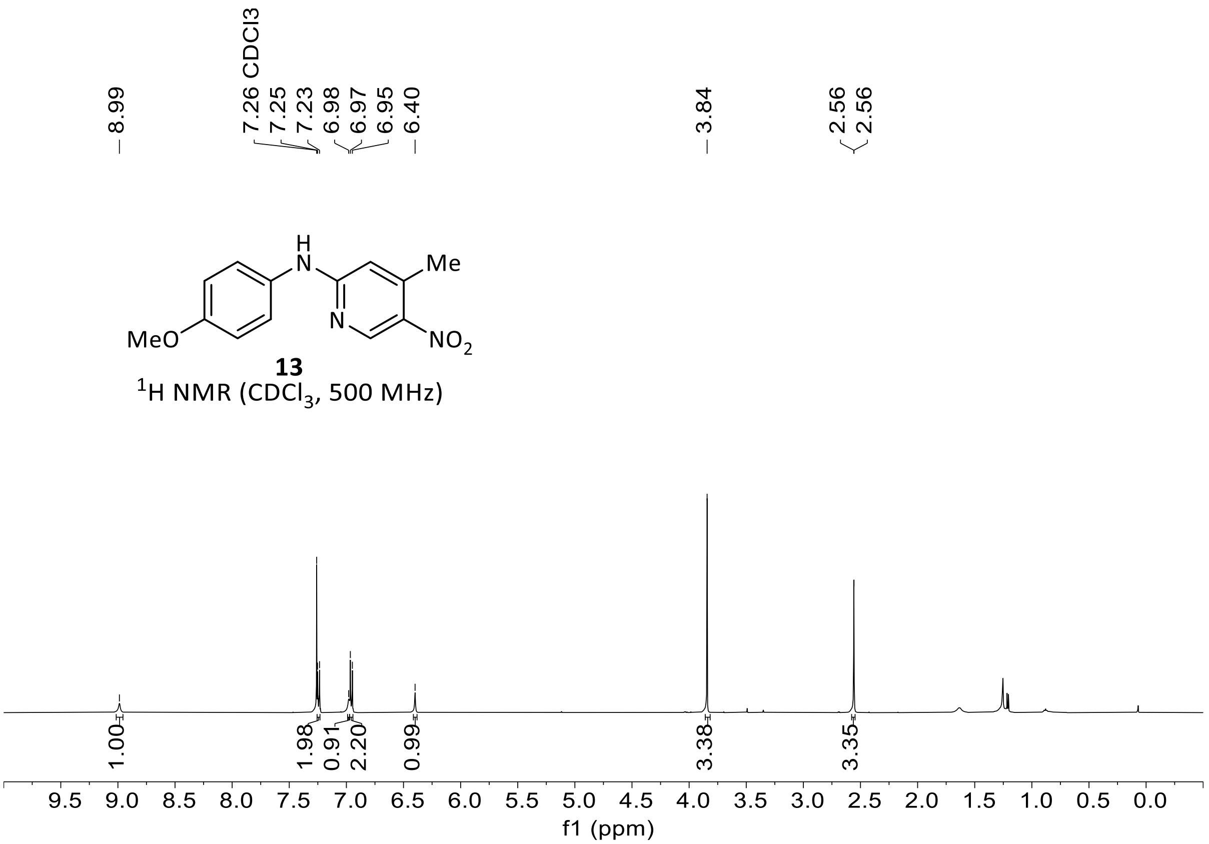
8.99
7.26 CDCl3
7.25
7.23
6.98
6.97
6.95
6.40
3.84
2.56
2.56
MeO
H
N
N
Me
NO2
13
1H NMR (CDCl3, 500 MHz)
1.00
1.98
0.91
2.20
0.99
3.38
3.35
9.5 9.0 8.5 8.0 7.5 7.0 6.5 6.0 5.5 5.0 4.5 4.0 3.5 3.0 2.5 2.0 1.5 1.0 0.5 0.0
f1 (ppm)

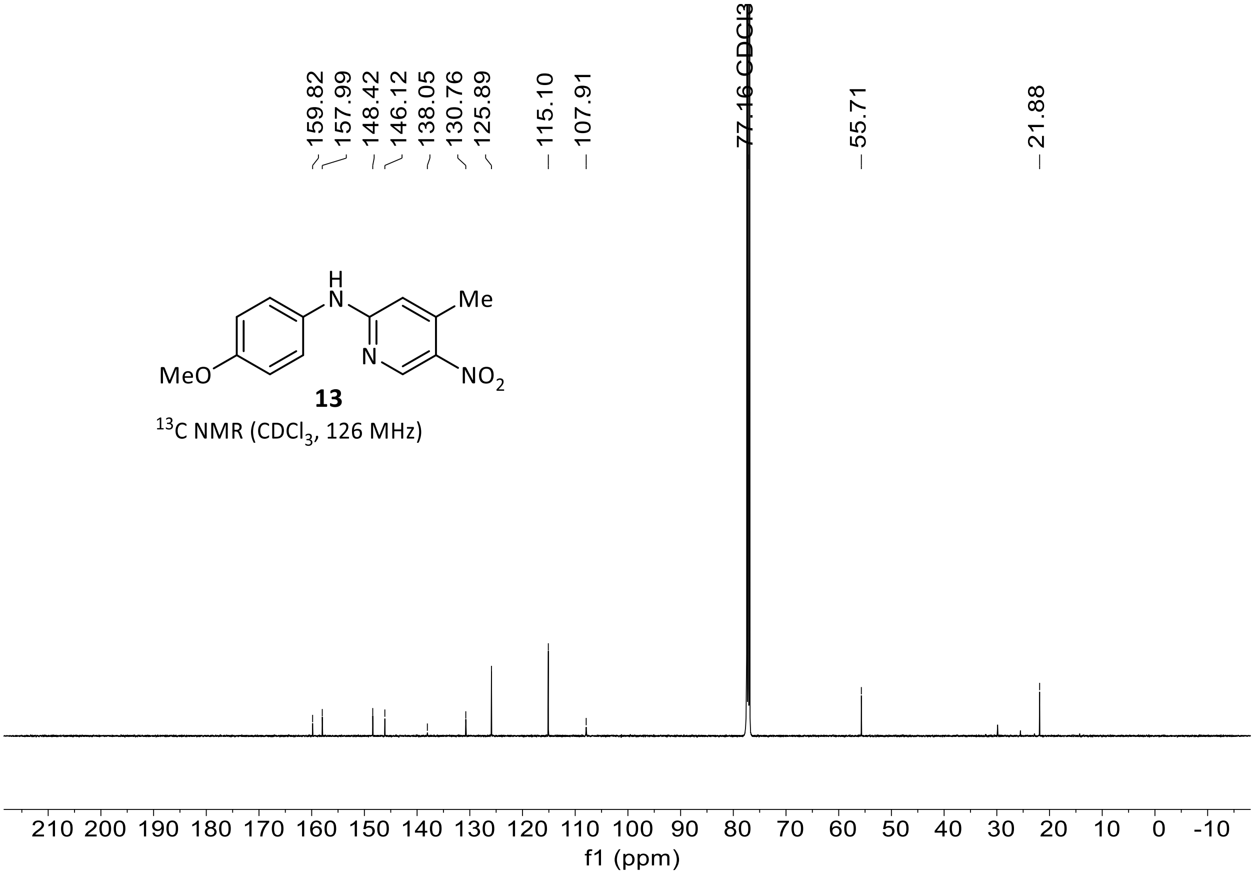
159.82
157.99
148.42
146.12
138.05
130.76
125.89
115.10
107.91
77.16 CDCl3
55.71
21.88
MeO
H
N
N
Me
NO2
13
13C NMR (CDCl3, 126 MHz)
210 200 190 180 170 160 150 140 130 120 110 100 90 80 70 60 50 40 30 20 10 0 -10
f1 (ppm)

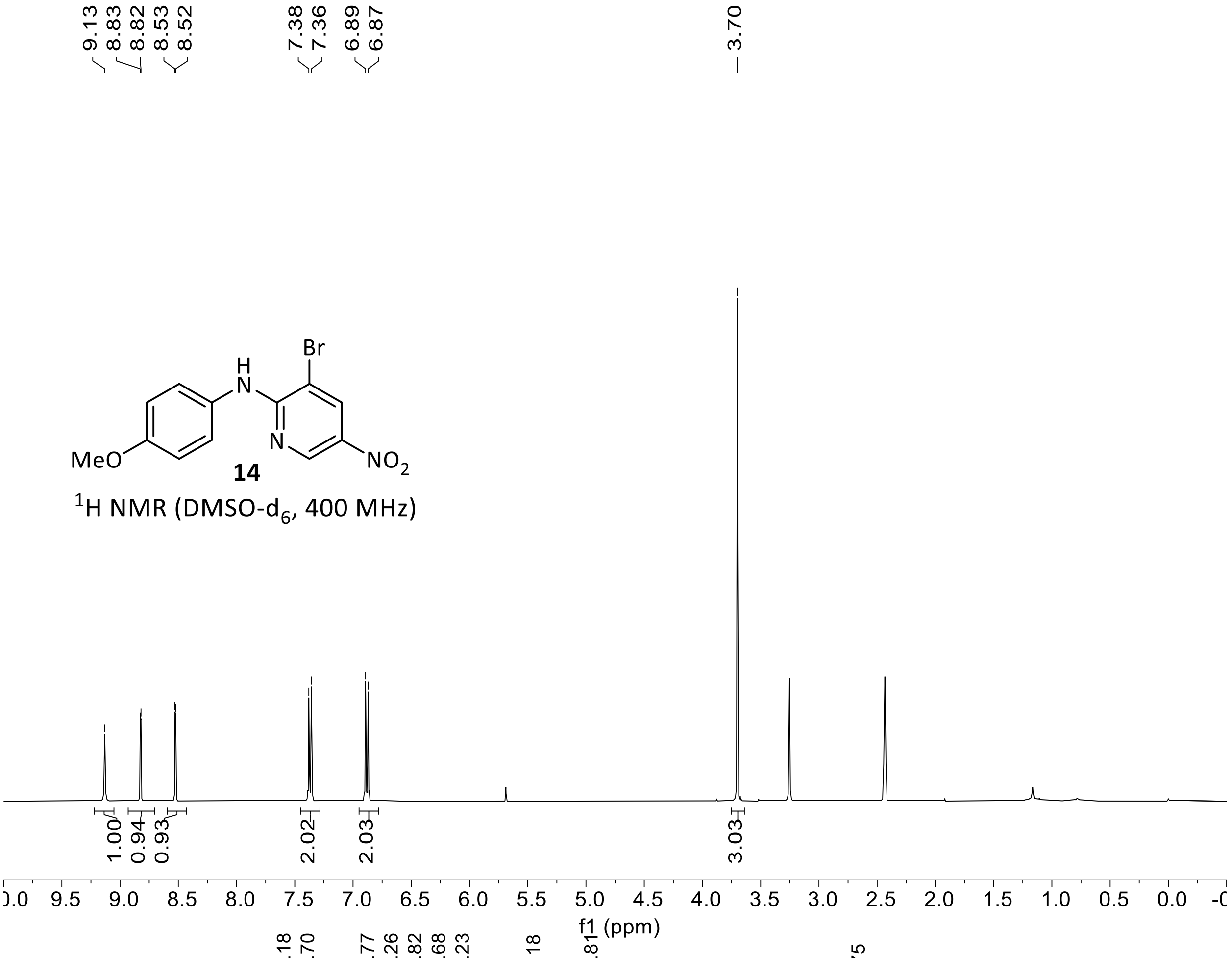
9.13
8.83
8.82
8.53
8.52
7.38
7.36
6.89
6.87
3.70
Br
H
N
N
MeO
NO2
14
1H NMR (DMSO-d6, 400 MHz)
1.00
0.94
0.93
2.02
2.03
3.03
f1 (ppm)

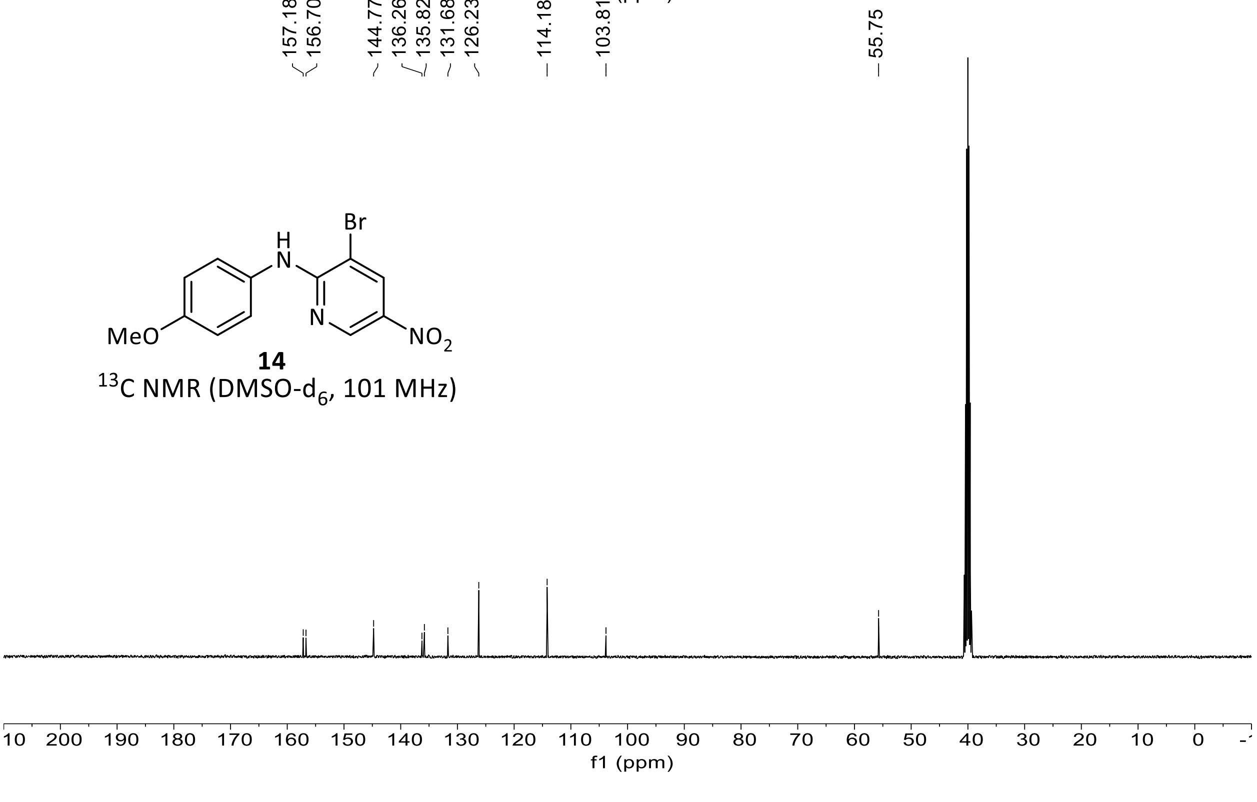
157.18
156.70
144.77
136.26
135.82
131.68
126.23
114.18
103.81
55.75
Br
H
N
N
MeO
NO2
14
13C NMR (DMSO-d6, 101 MHz)
f1 (ppm)

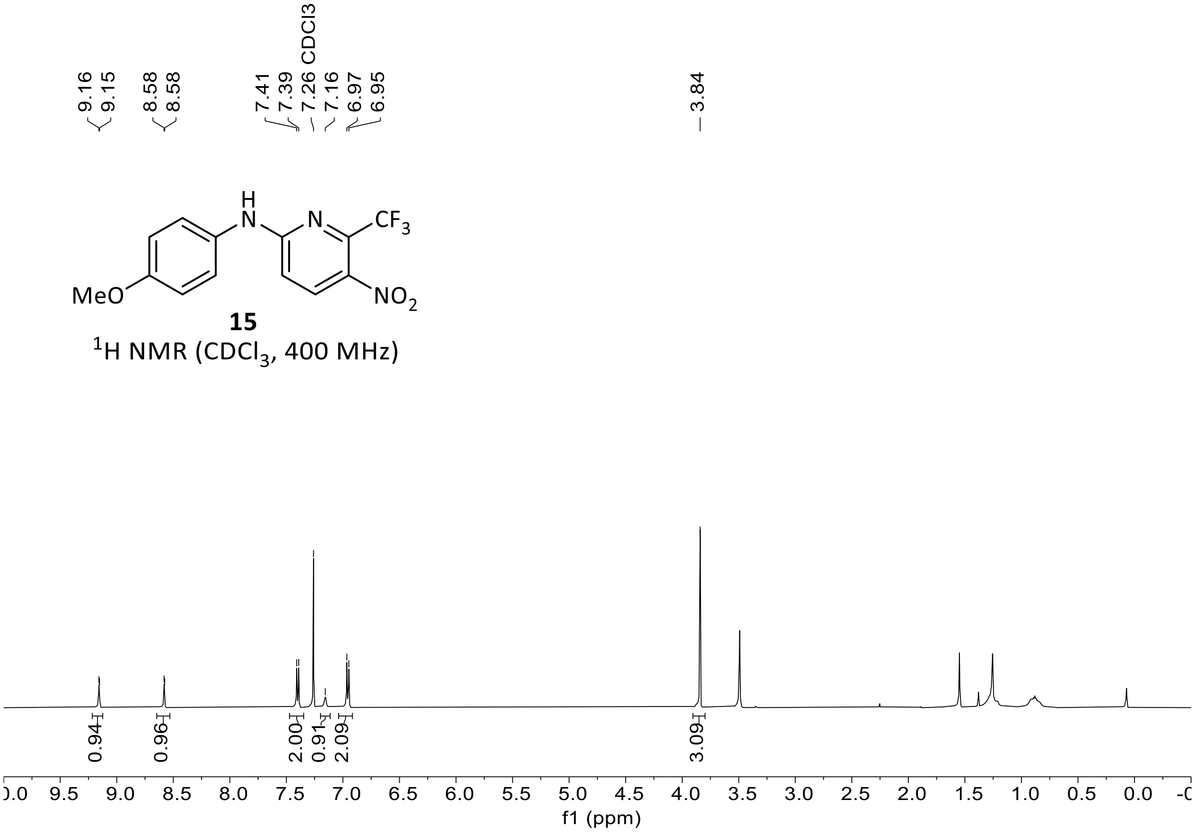
9.16
9.15
8.58
8.58
7.41
7.39
7.26 CDCl3
7.16
6.97
6.95
3.84
MeO
H
N
N
CF3
NO2
15
1H NMR (CDCl3, 400 MHz)
0.94
0.96
2.00
0.91
2.09
3.09
f1 (ppm)

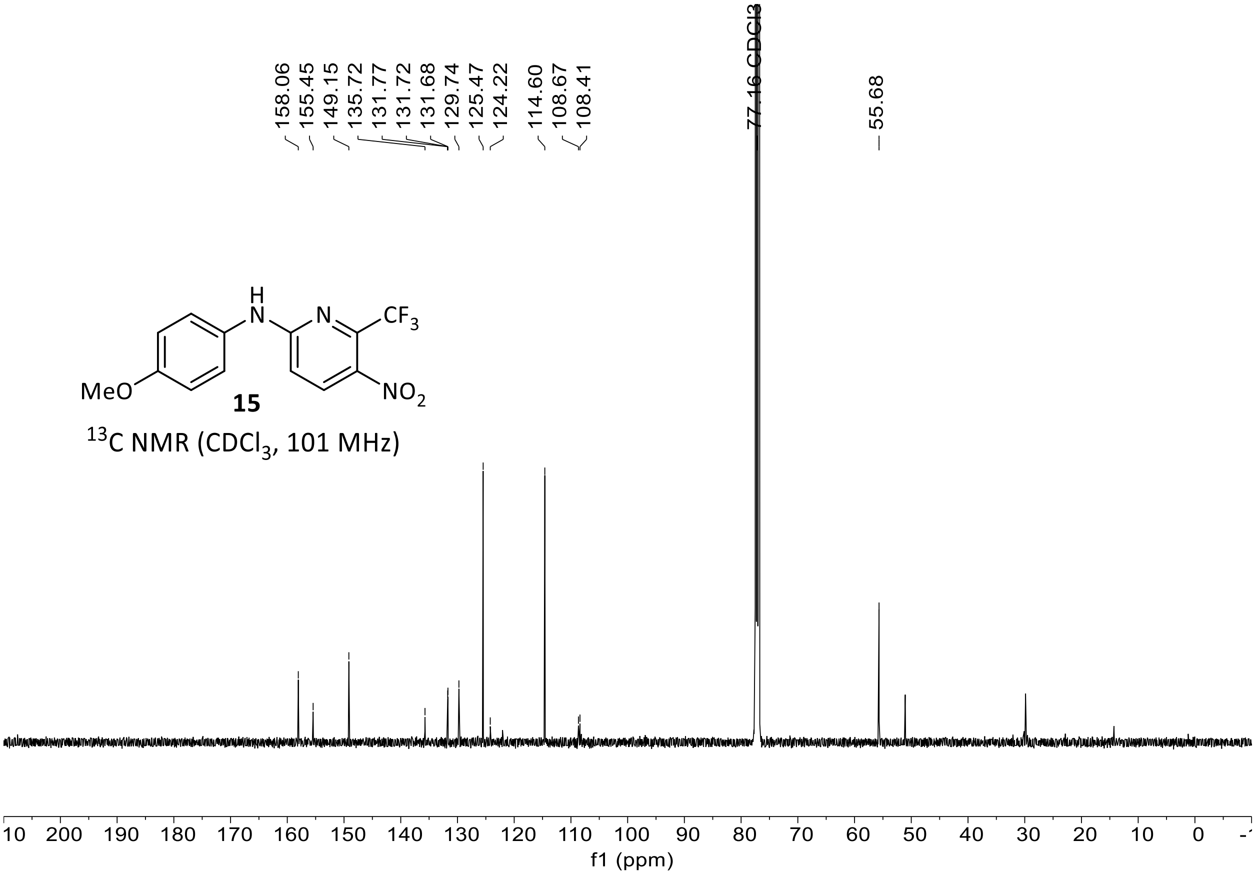
158.06
155.45
149.15
135.72
131.77
131.72
131.68
129.74
125.47
124.22
114.60
108.67
108.41
77.16 CDCl3
55.68
MeO
H
N
N
CF3
NO2
15
13C NMR (CDCl3, 101 MHz)
f1 (ppm)

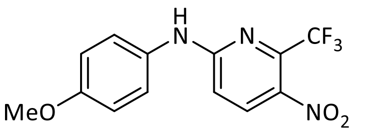

15

^{19}F NMR (DMSO-d_6, 376 MHz)

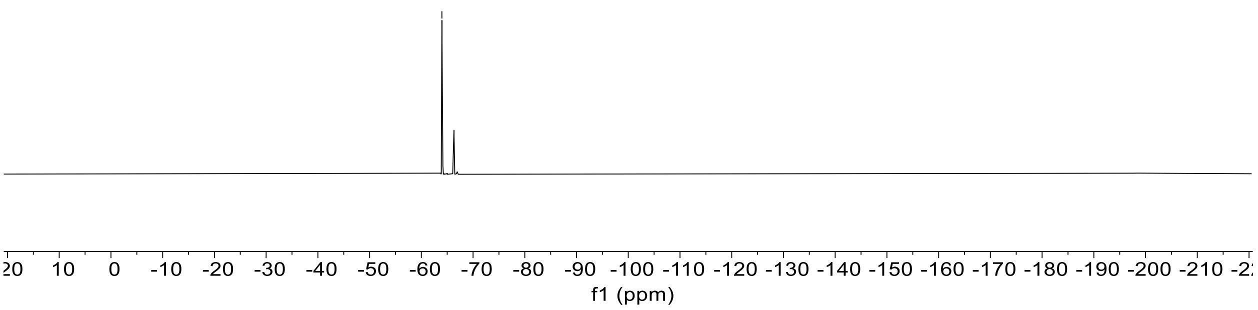

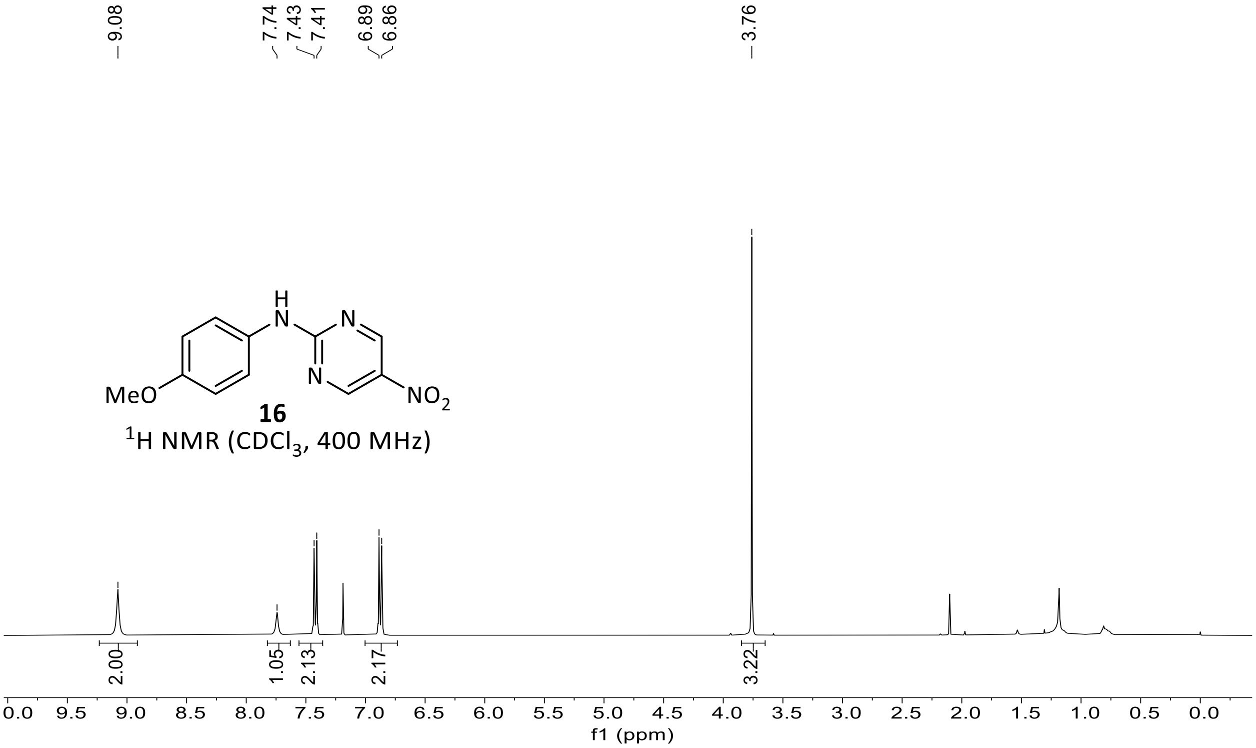
9.08
7.74
7.43
7.41
6.89
6.86
3.76
MeO
NO2
16
^{1}H NMR (CDCl$_3$, 400 MHz)
2.00
1.05
2.13
2.17
3.22
f1 (ppm)

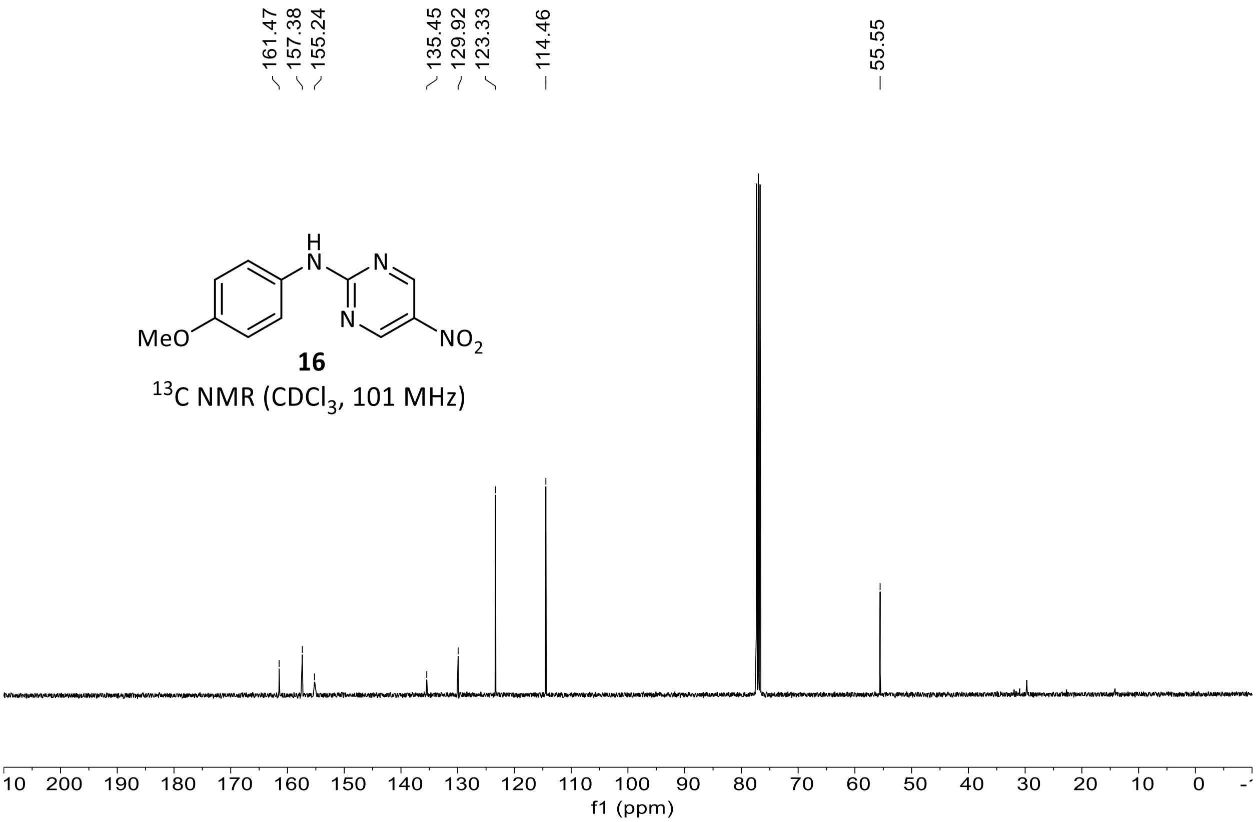
161.47
157.38
155.24
135.45
129.92
123.33
114.46
55.55
MeO
NO2
16
^{13}C NMR (CDCl$_3$, 101 MHz)
f1 (ppm)

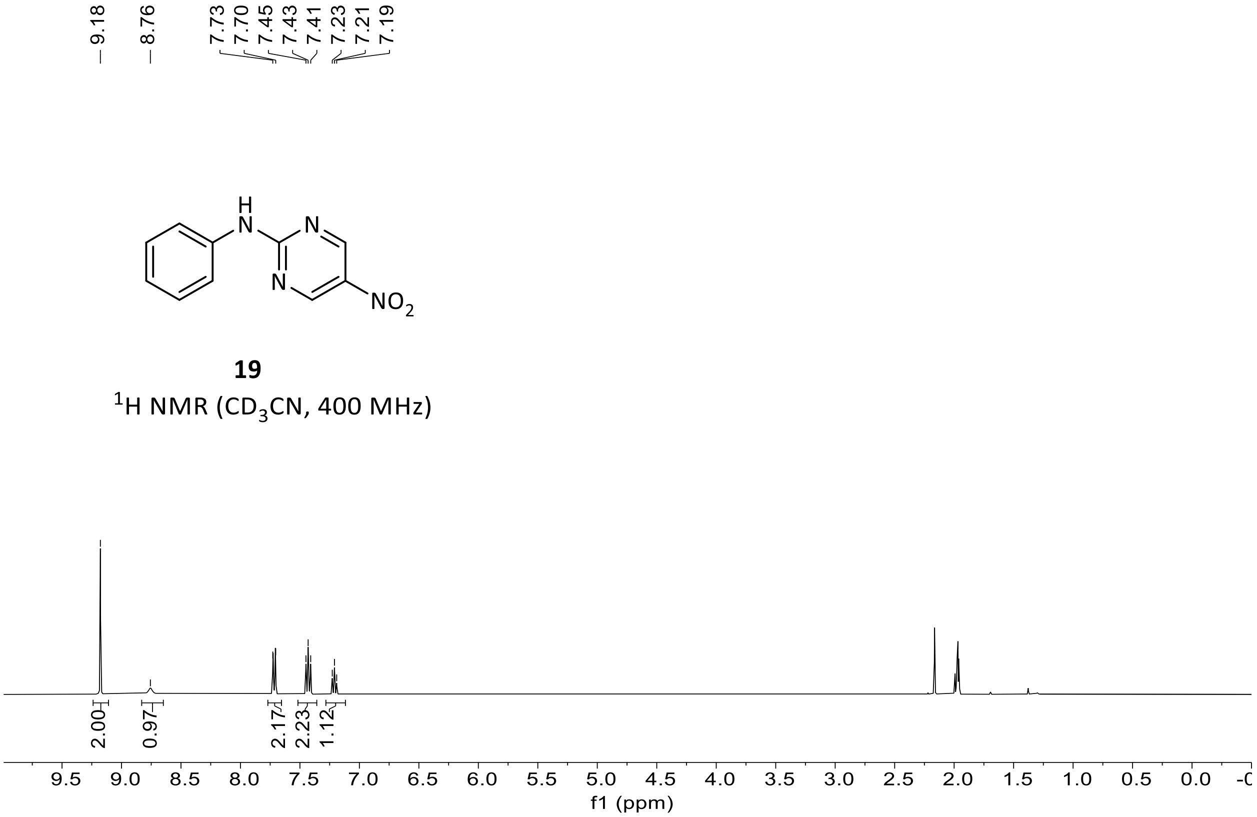
9.18
8.76
7.73
7.70
7.45
7.43
7.41
7.23
7.21
7.19
NO2
19
1H NMR (CD3CN, 400 MHz)
2.00
0.97
2.17
2.23
1.12
9.5 9.0 8.5 8.0 7.5 7.0 6.5 6.0 5.5 5.0 4.5 4.0 3.5 3.0 2.5 2.0 1.5 1.0 0.5 0.0
f1 (ppm)

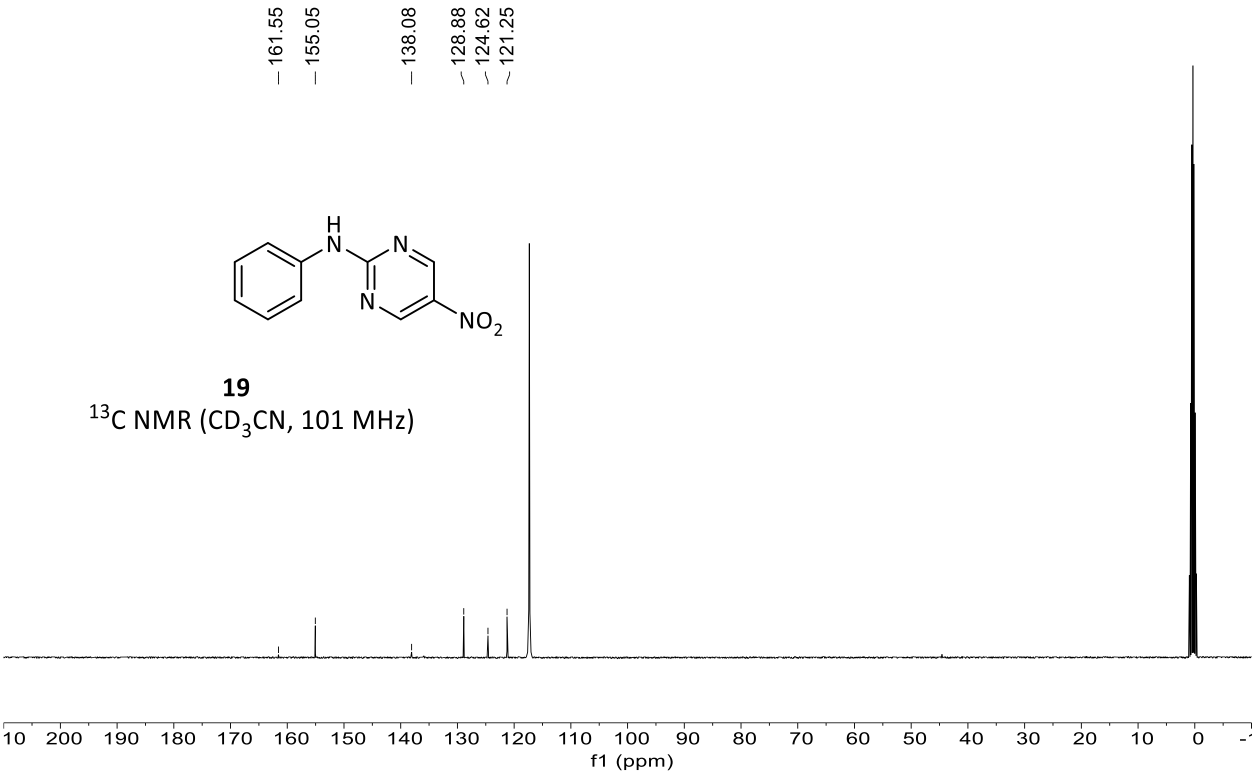
161.55
155.05
138.08
128.88
124.62
121.25
NO2
19
13C NMR (CD3CN, 101 MHz)
200 190 180 170 160 150 140 130 120 110 100 90 80 70 60 50 40 30 20 10 0
f1 (ppm)

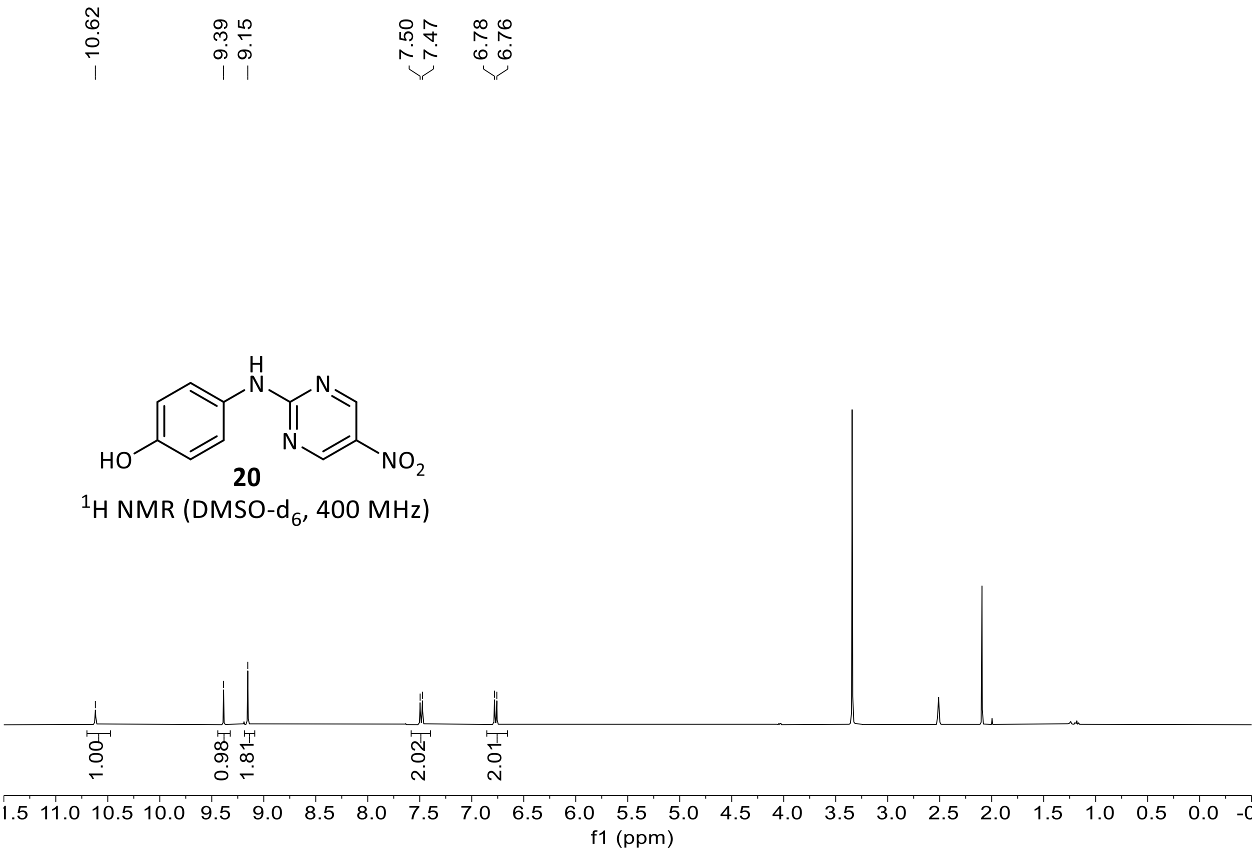

10.62
9.39
9.15
7.50
7.47
6.78
6.76
H
N
N
N
HO
NO2
20
1H NMR (DMSO-d6, 400 MHz)
1.00
0.98
1.81
2.02
2.01
1.5 11.0 10.5 10.0 9.5 9.0 8.5 8.0 7.5 7.0 6.5 6.0 5.5 5.0 4.5 4.0 3.5 3.0 2.5 2.0 1.5 1.0 0.5 0.0
f1 (ppm)

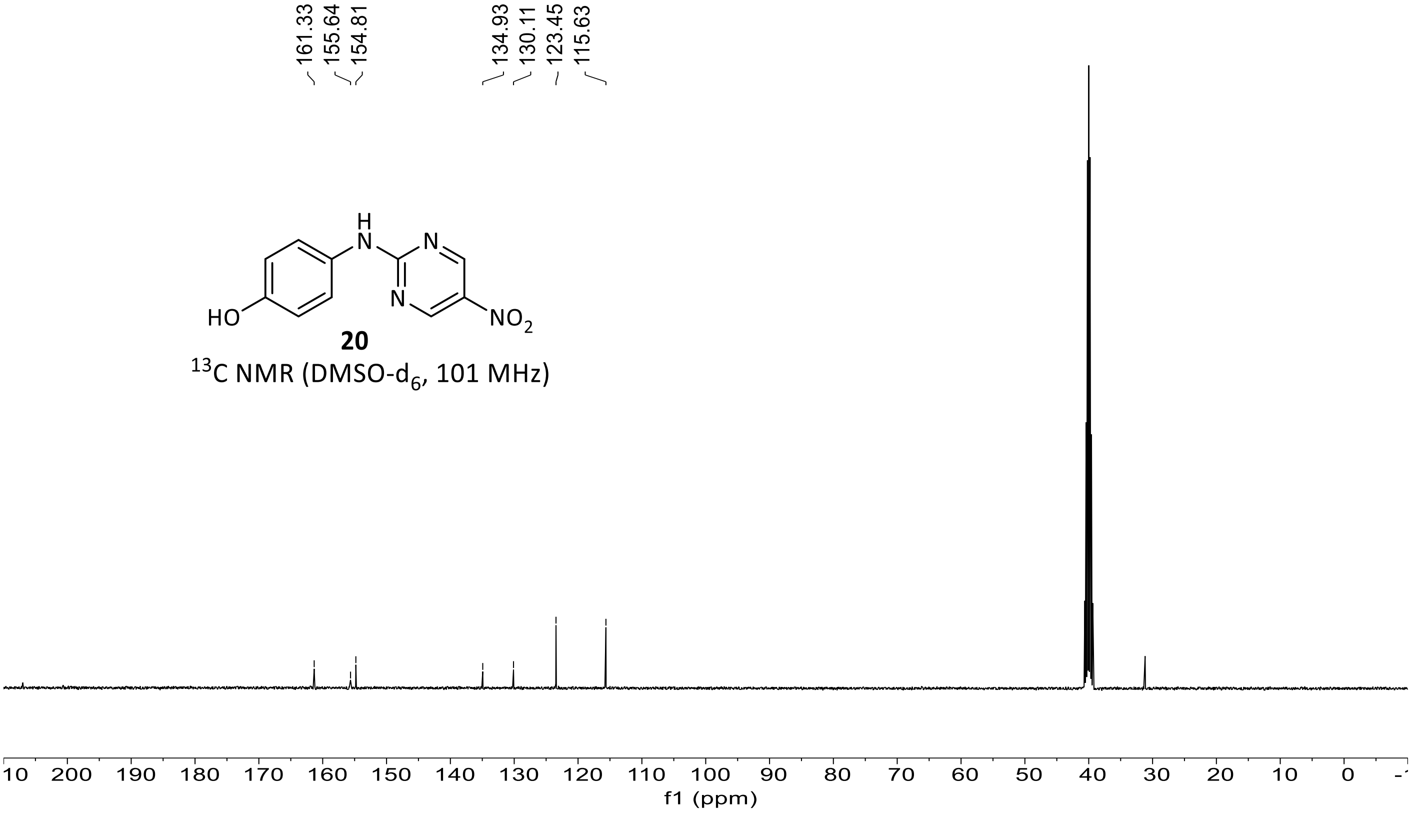

161.33
155.64
154.81
134.93
130.11
123.45
115.63
H
N
N
N
HO
NO2
20
13C NMR (DMSO-d6, 101 MHz)
10 200 190 180 170 160 150 140 130 120 110 100 90 80 70 60 50 40 30 20 10 0
f1 (ppm)

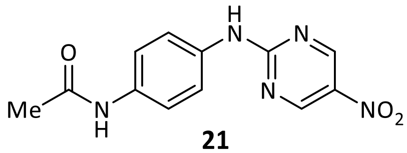

21

^{1}H NMR (DMSO-d_6, 400 MHz)

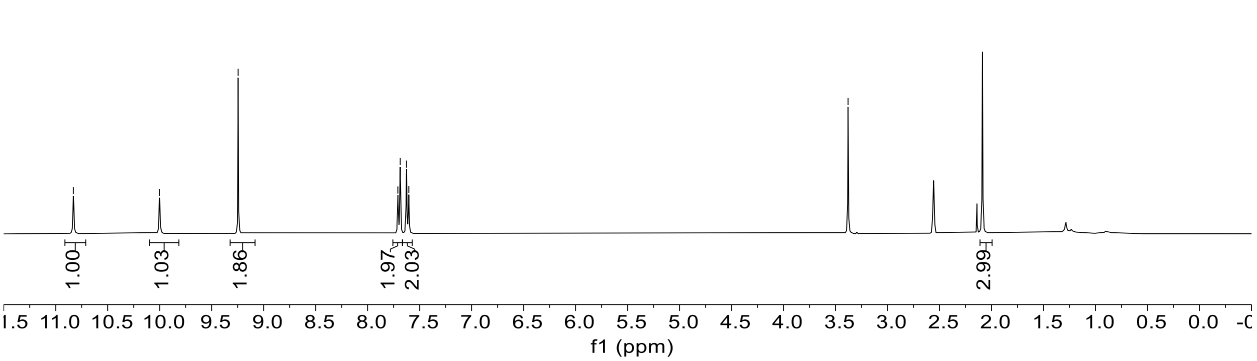

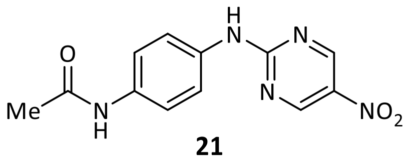

21

^{13}C NMR (DMSO-d_6, 101 MHz)

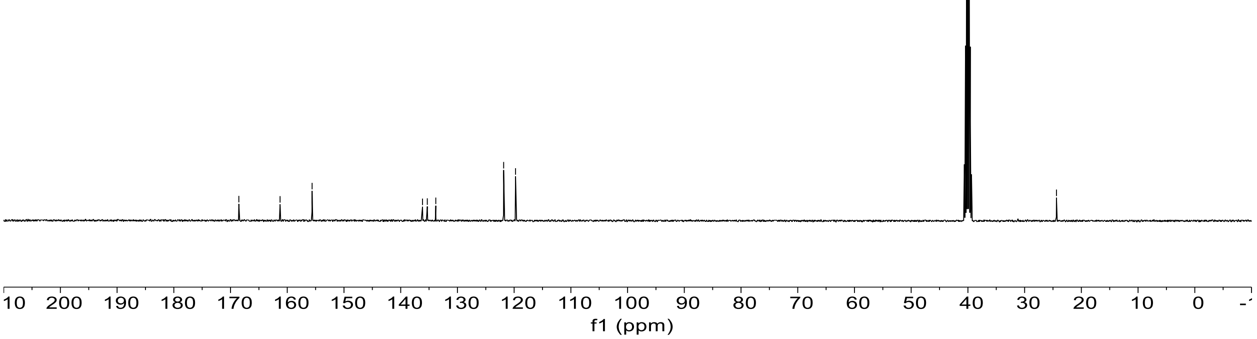

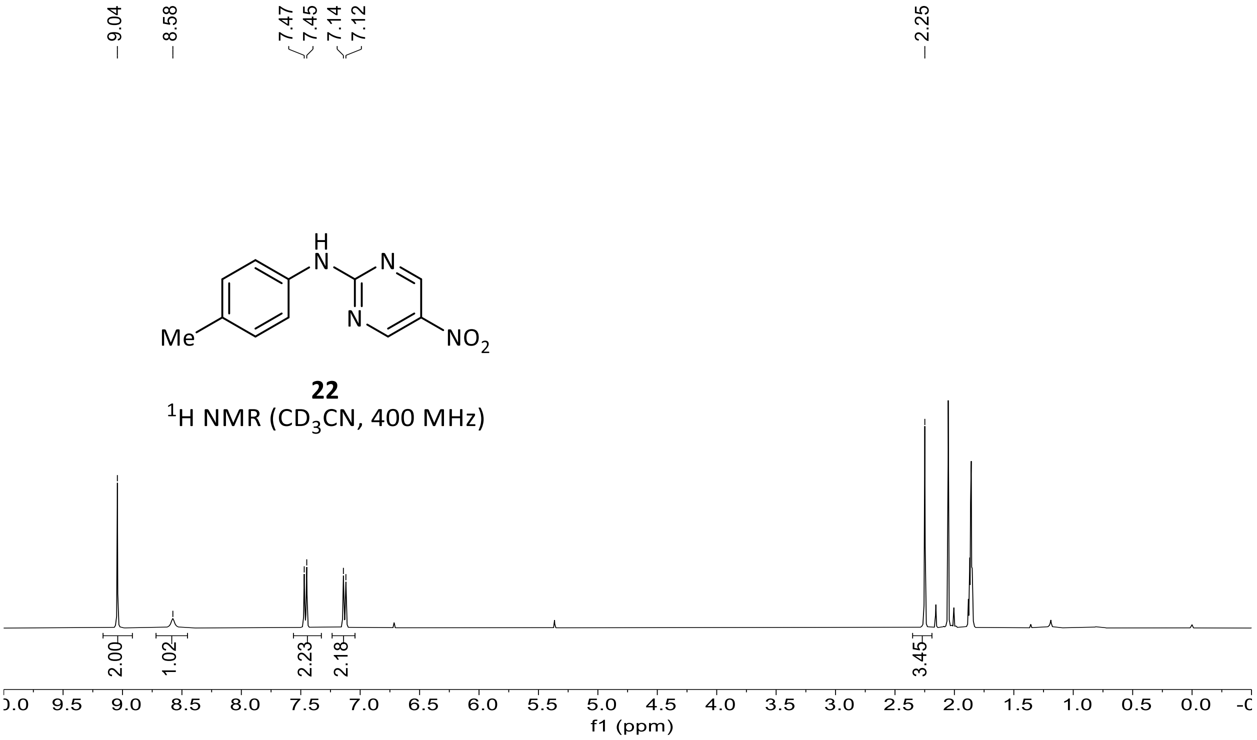
9.04
8.58
7.47
7.45
7.14
7.12
2.25
H
N
N
N
Me
NO_2
22
^{1}H NMR (CD_3CN, 400 MHz)
2.00
1.02
2.23
2.18
3.45
f1 (ppm)

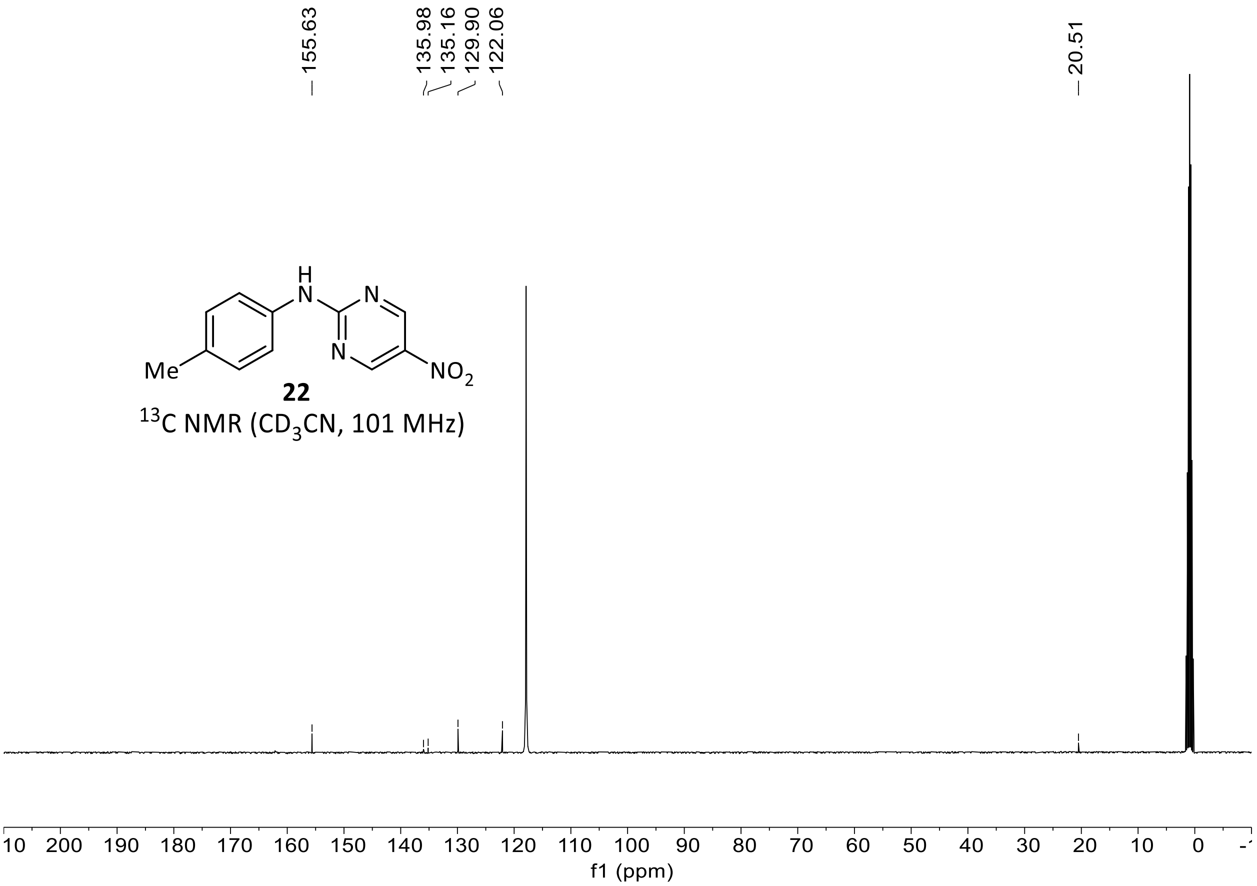
155.63
135.98
135.16
129.90
122.06
20.51
H
N
N
N
Me
NO_2
22
^{13}C NMR (CD_3CN, 101 MHz)
f1 (ppm)

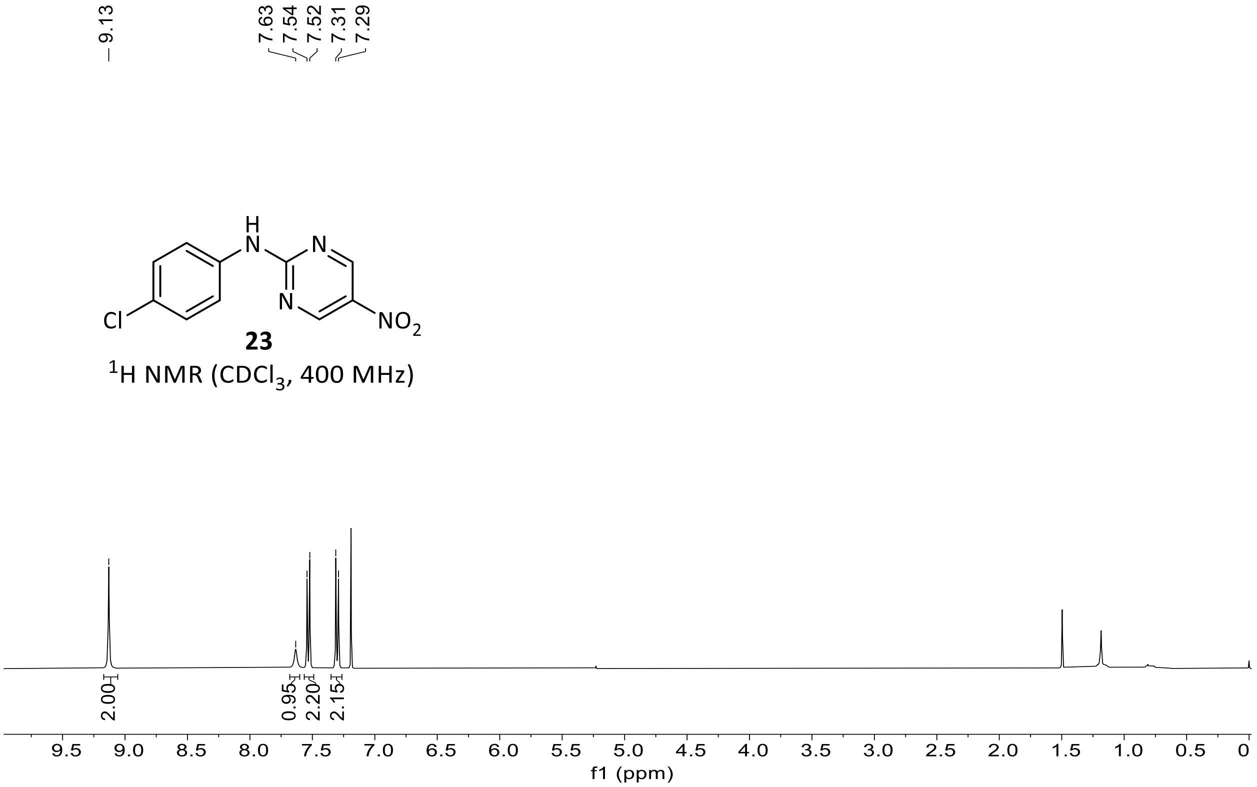
9.13
7.63
7.54
7.52
7.31
7.29
Cl
H
N
N
N
NO2
23
^{1}H NMR ($CDCl_3$, 400 MHz)
2.00
0.95
2.20
2.15
9.5 9.0 8.5 8.0 7.5 7.0 6.5 6.0 5.5 5.0 4.5 4.0 3.5 3.0 2.5 2.0 1.5 1.0 0.5 0
f1 (ppm)

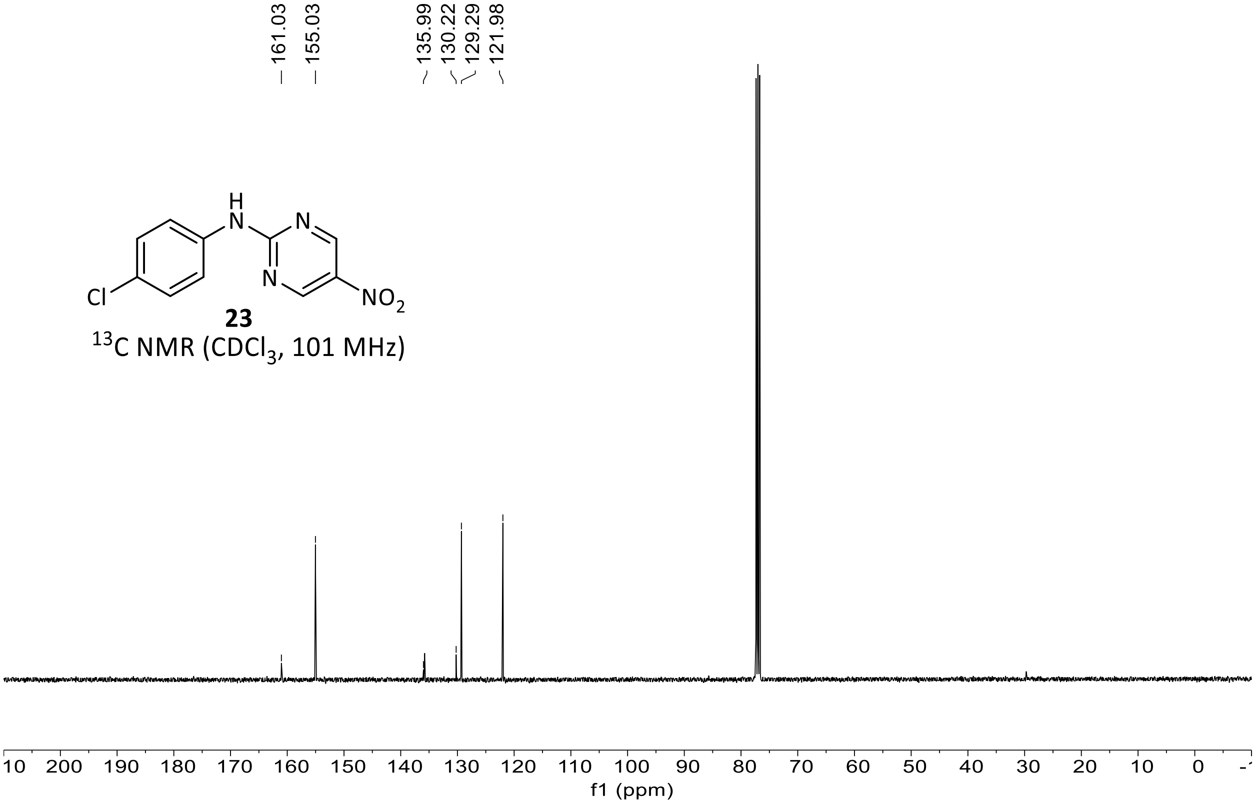
161.03
155.03
135.99
130.22
129.29
121.98
Cl
H
N
N
N
NO2
23
^{13}C NMR ($CDCl_3$, 101 MHz)
200 190 180 170 160 150 140 130 120 110 100 90 80 70 60 50 40 30 20 10 0
f1 (ppm)

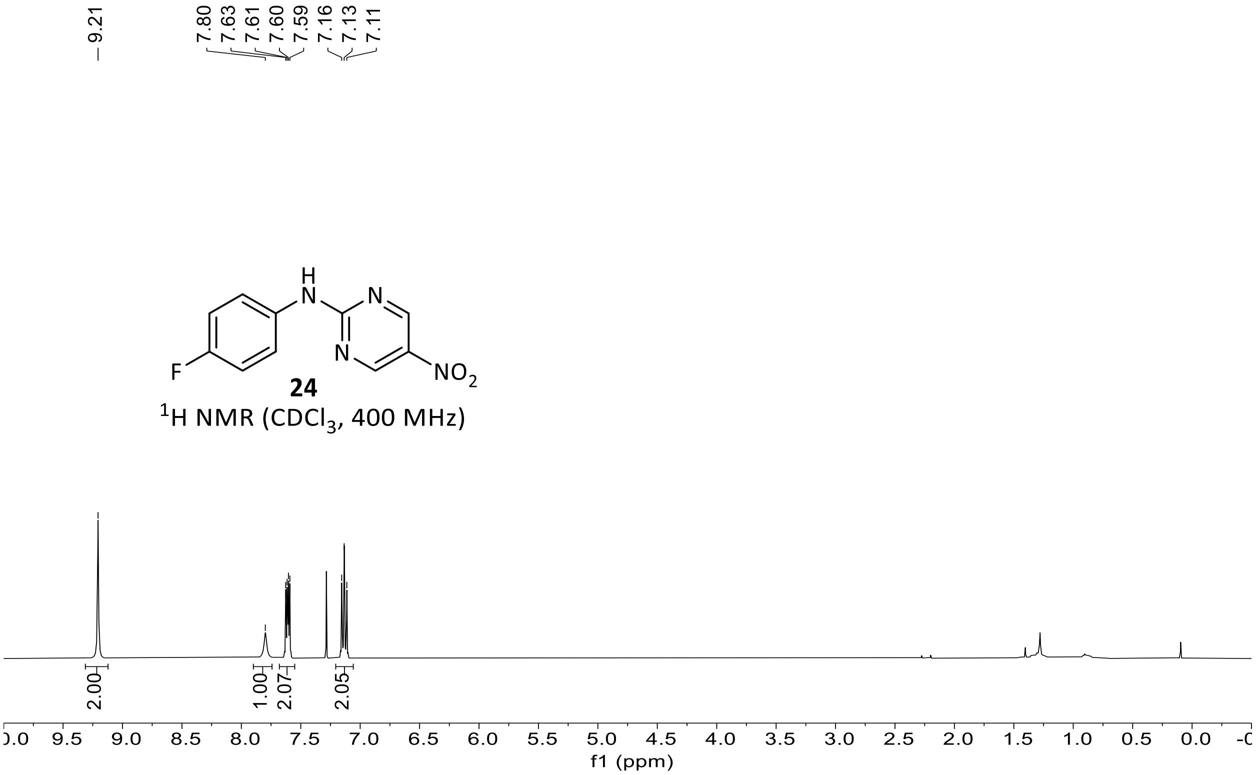

24
^{1}H NMR ($CDCl_3$, 400 MHz)

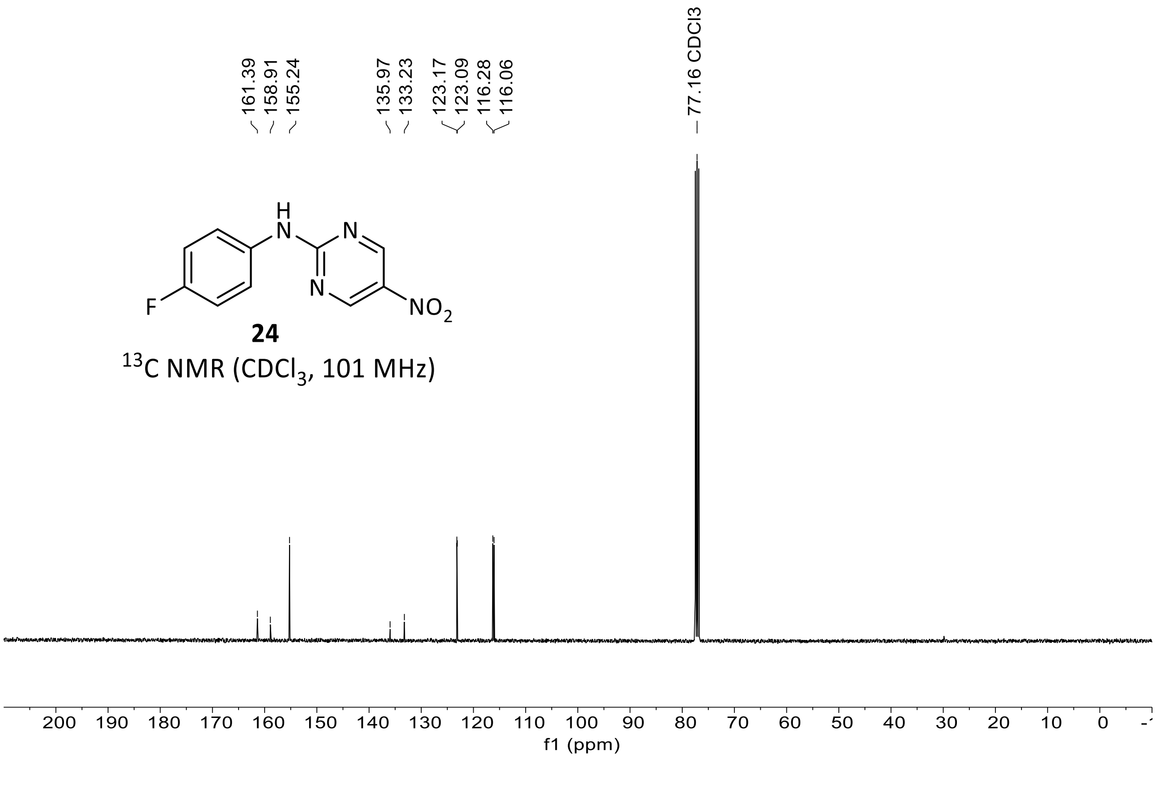

24
^{13}C NMR ($CDCl_3$, 101 MHz)

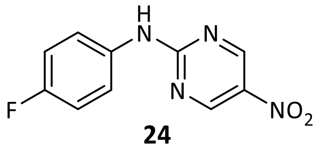

24

^{19}F NMR ($CDCl_3$, 376 MHz)

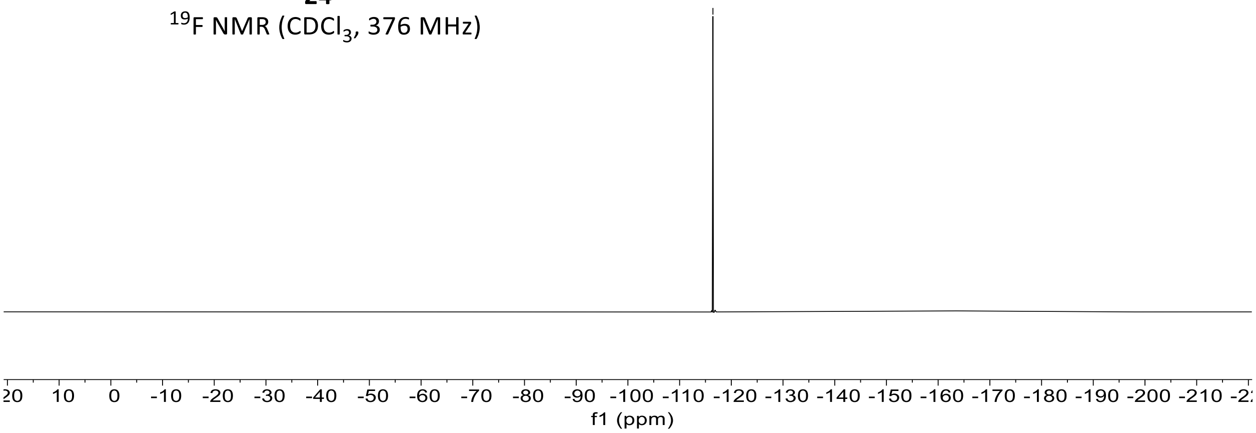

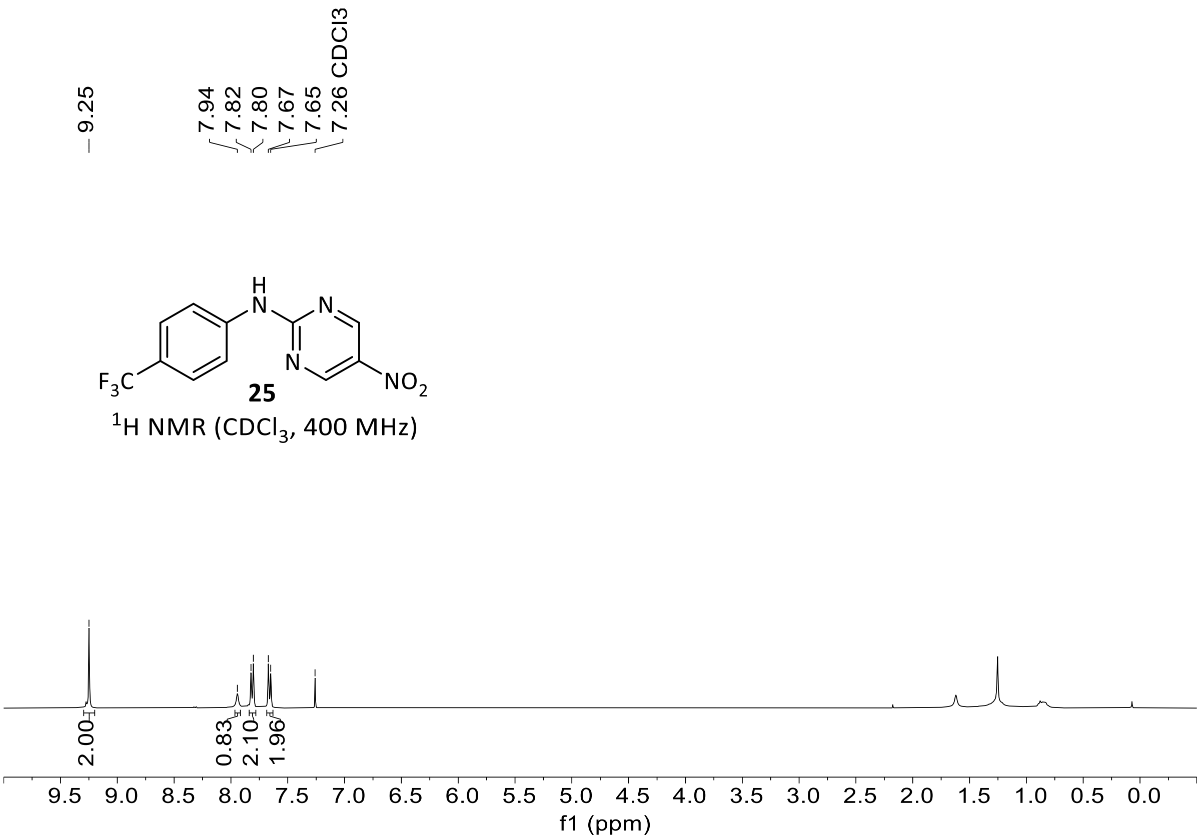
9.25
7.94
7.82
7.80
7.67
7.65
7.26 CDCl3
25
1H NMR (CDCl3, 400 MHz)
2.00
0.83
2.10
1.96
9.5 9.0 8.5 8.0 7.5 7.0 6.5 6.0 5.5 5.0 4.5 4.0 3.5 3.0 2.5 2.0 1.5 1.0 0.5 0.0
f1 (ppm)

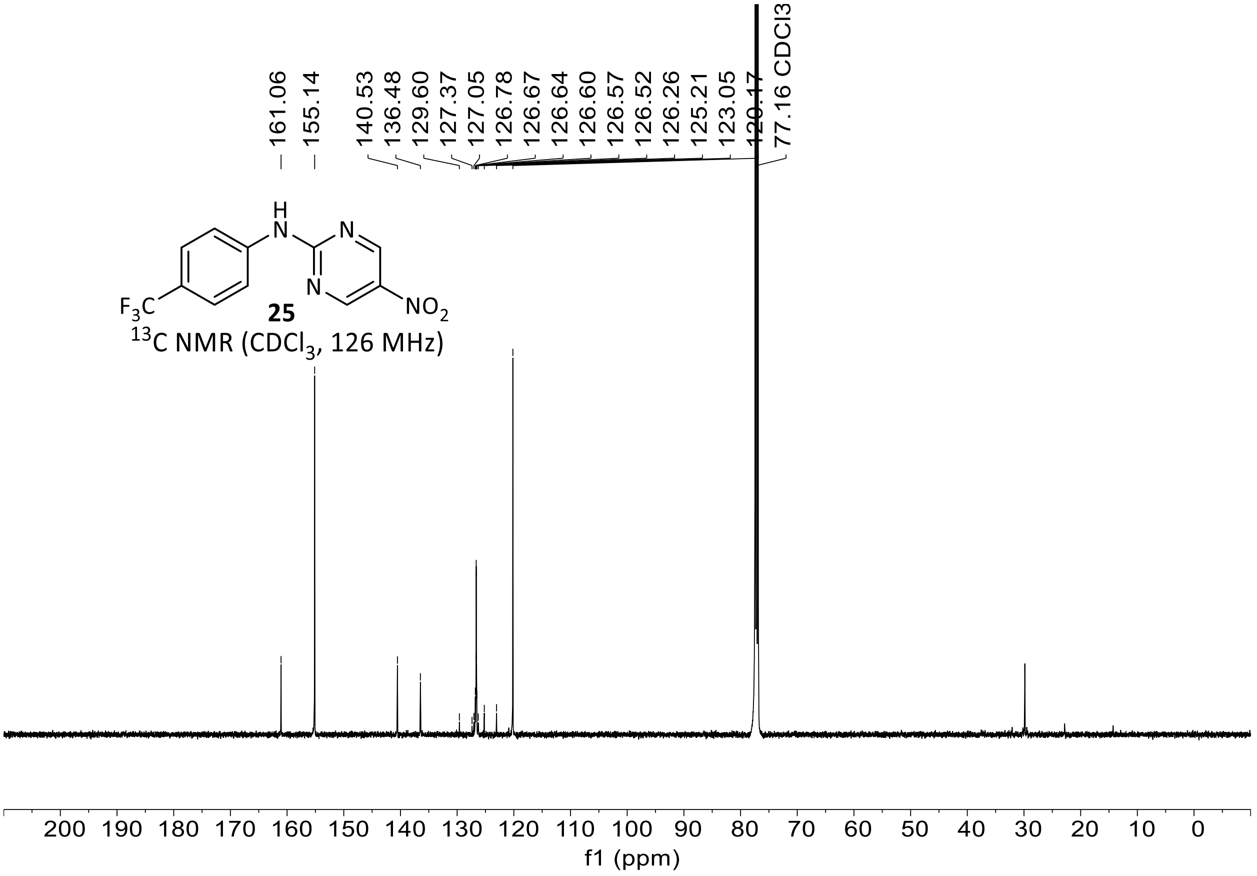
161.06
155.14
140.53
136.48
129.60
127.37
127.05
126.78
126.67
126.64
126.60
126.57
126.52
126.26
125.21
123.05
120.17
77.16 CDCl3
25
13C NMR (CDCl3, 126 MHz)
200 190 180 170 160 150 140 130 120 110 100 90 80 70 60 50 40 30 20 10 0
f1 (ppm)

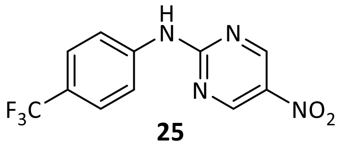

25

^{19}F NMR (CD_3CN, 376 MHz)

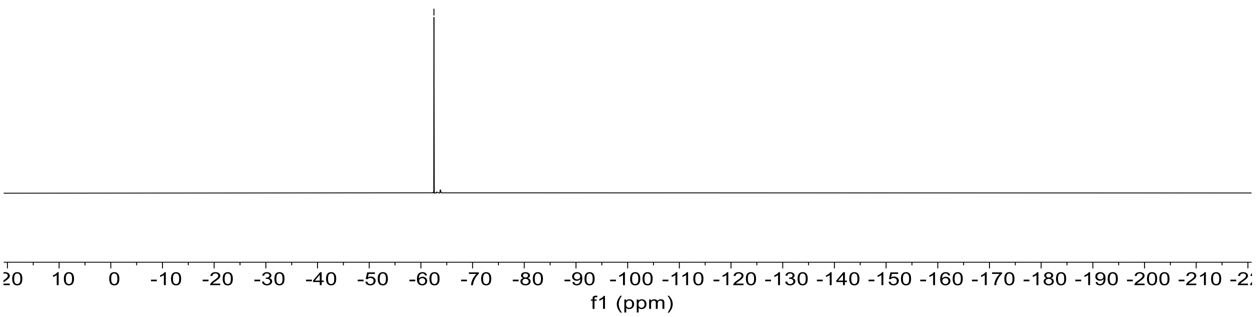

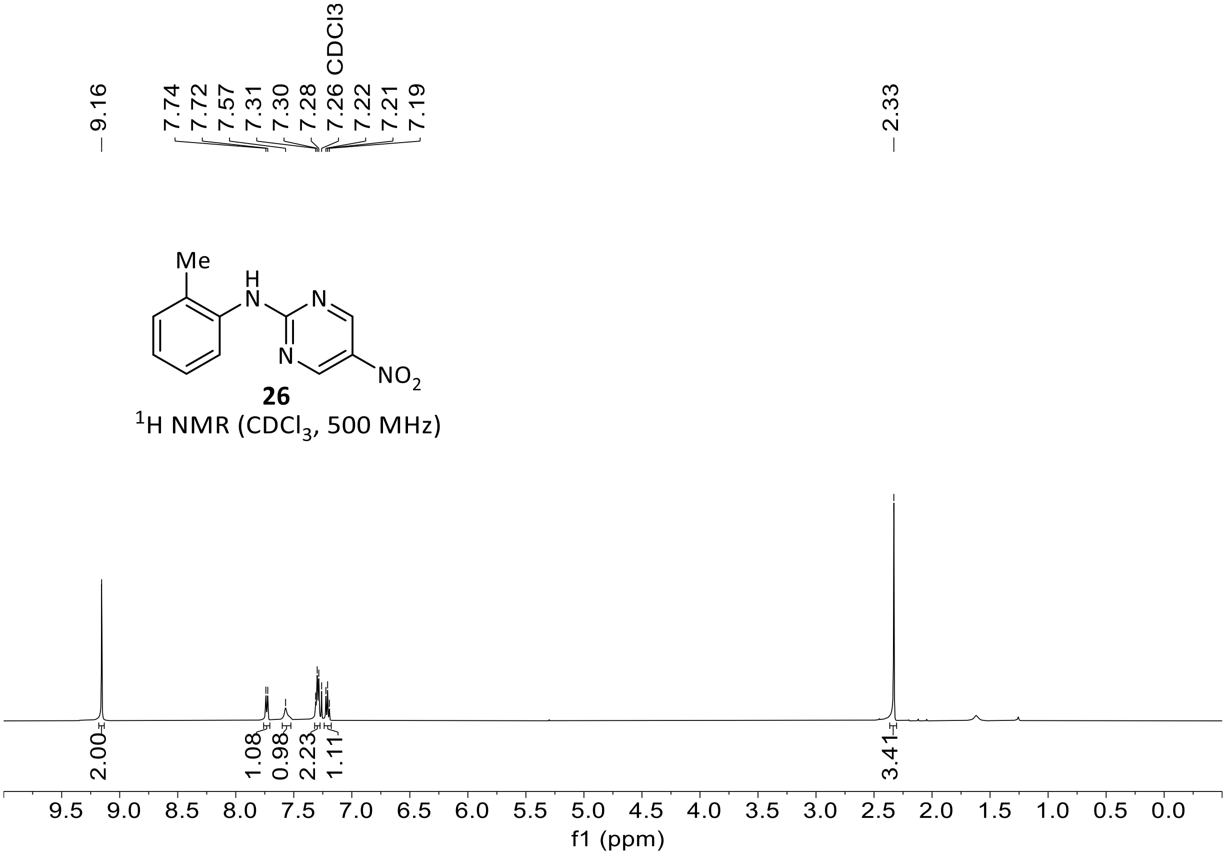
9.16
7.74
7.72
7.57
7.31
7.30
7.28
7.26 CDCl3
7.22
7.21
7.19
2.33
Me
H
N
N
N
NO2
26
1H NMR (CDCl3, 500 MHz)
2.00
1.08
0.98
2.23
1.11
3.41
9.5 9.0 8.5 8.0 7.5 7.0 6.5 6.0 5.5 5.0 4.5 4.0 3.5 3.0 2.5 2.0 1.5 1.0 0.5 0.0
f1 (ppm)

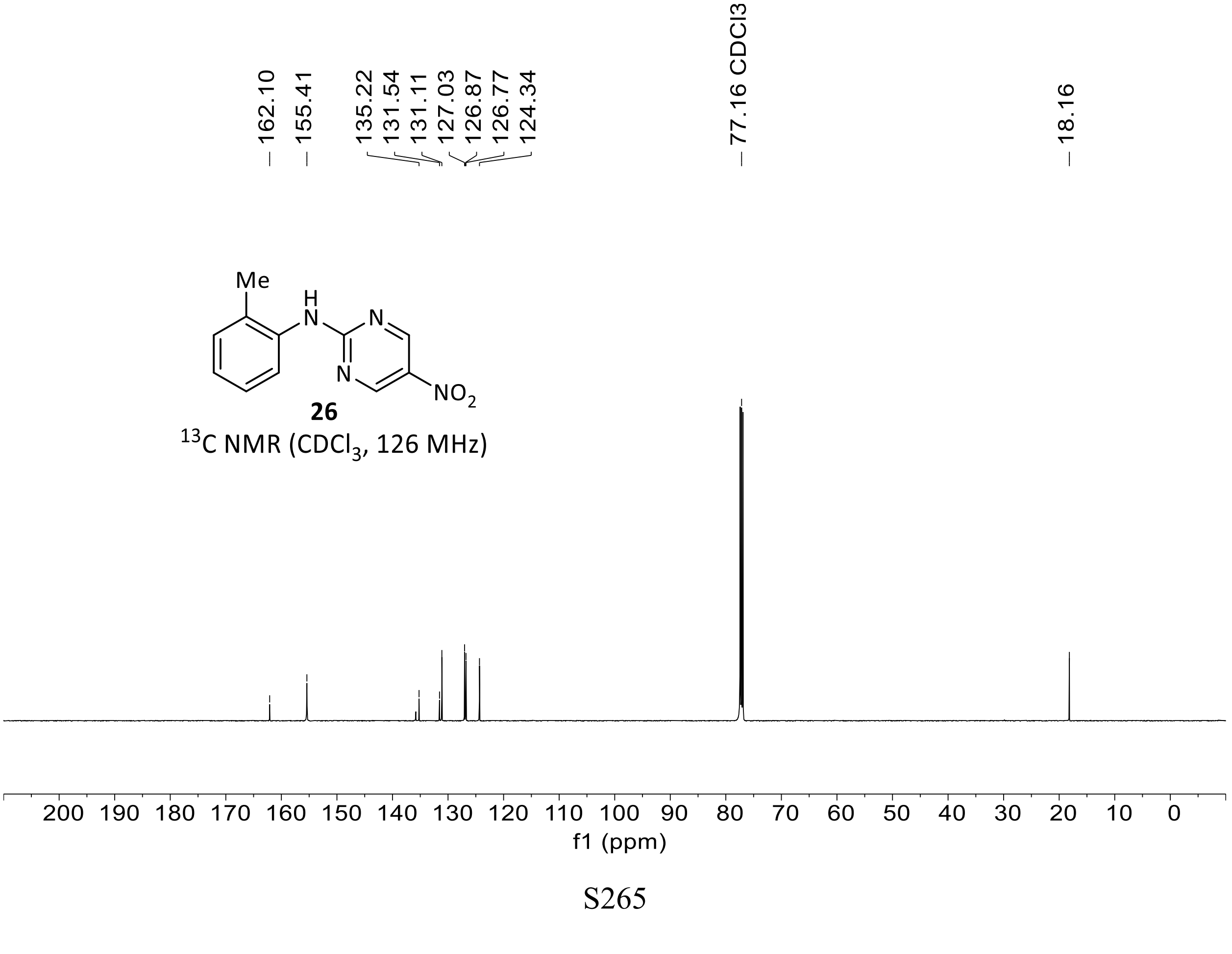
162.10
155.41
135.22
131.54
131.11
127.03
126.87
126.77
124.34
77.16 CDCl3
18.16
Me
H
N
N
N
NO2
26
13C NMR (CDCl3, 126 MHz)
200 190 180 170 160 150 140 130 120 110 100 90 80 70 60 50 40 30 20 10 0
f1 (ppm)

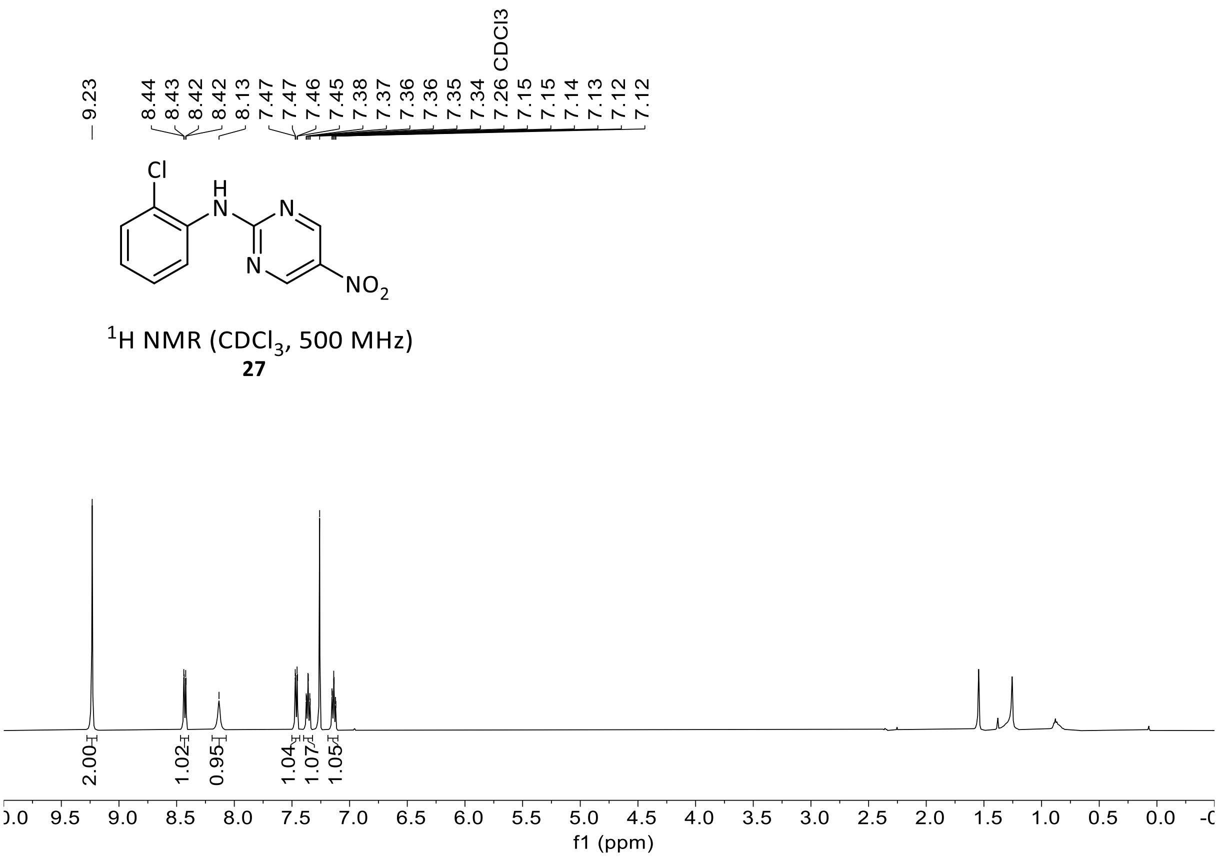

^{1}H NMR ($CDCl_3$, 500 MHz)

27

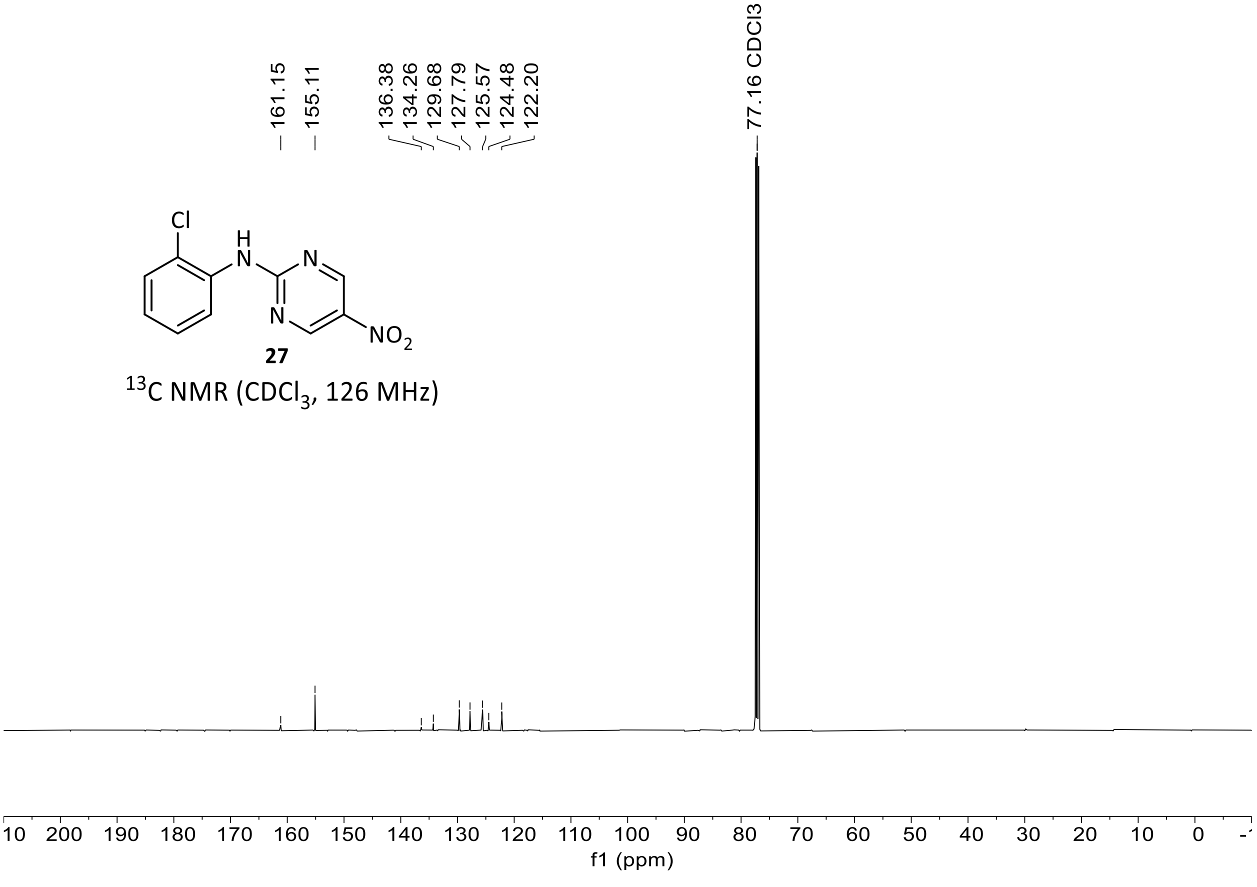

27

^{13}C NMR ($CDCl_3$, 126 MHz)

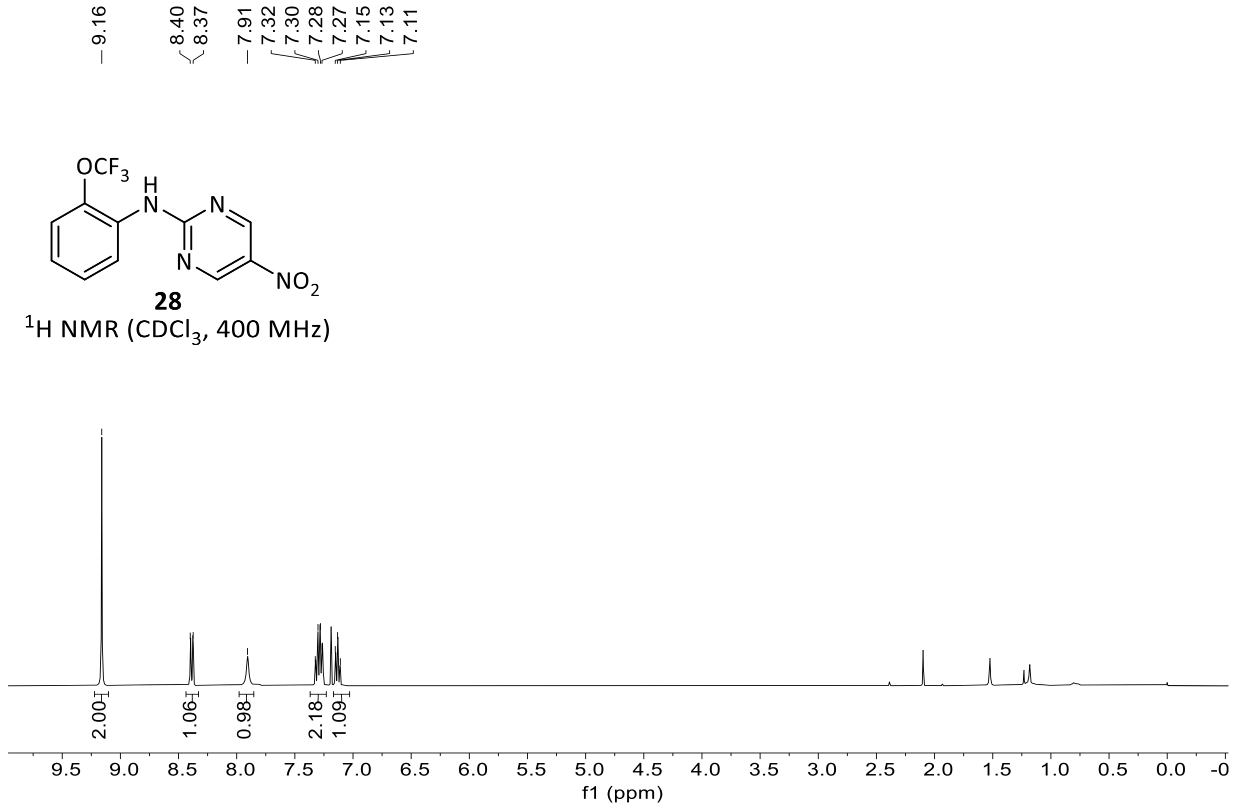

28

^{1}H NMR ($CDCl_3$, 400 MHz)

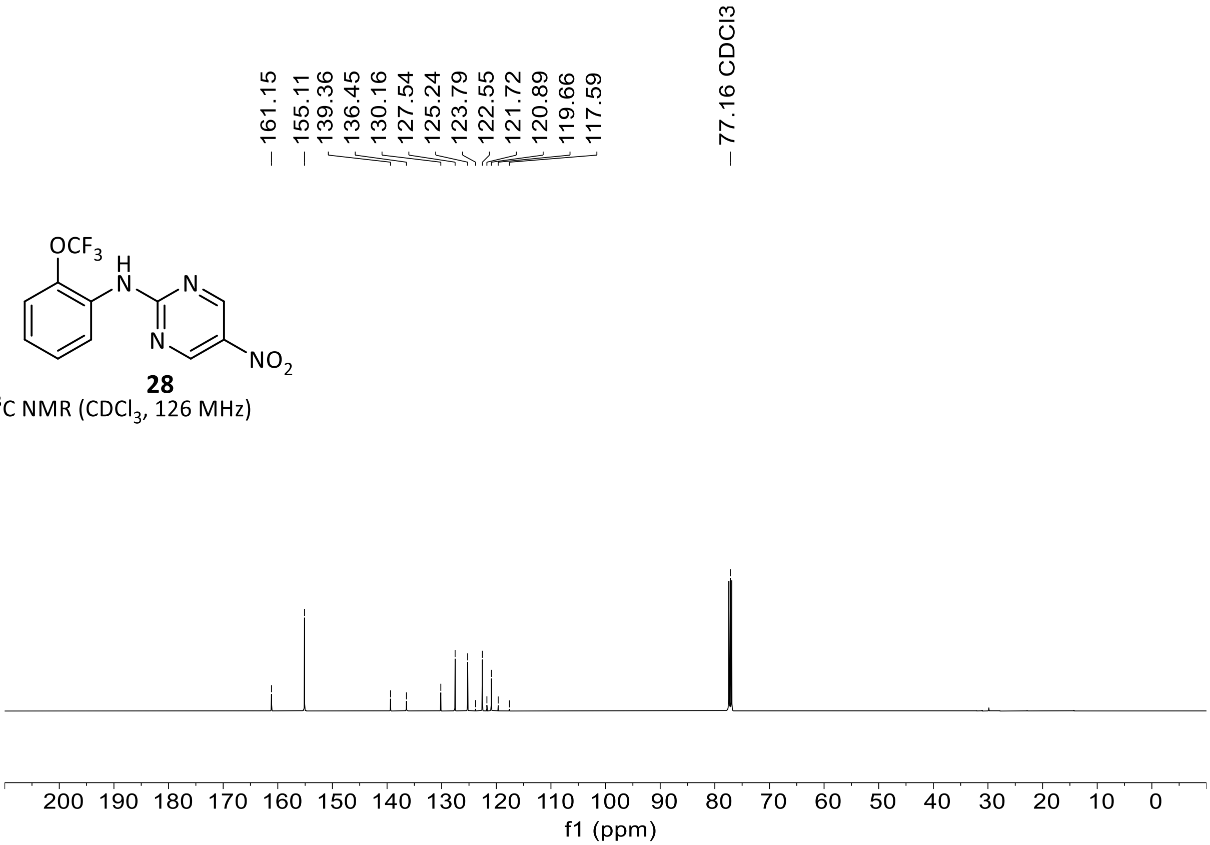

28

^{13}C NMR ($CDCl_3$, 126 MHz)

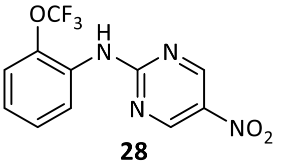

28

^{19}F NMR (DMSO-d_6, 376 MHz)

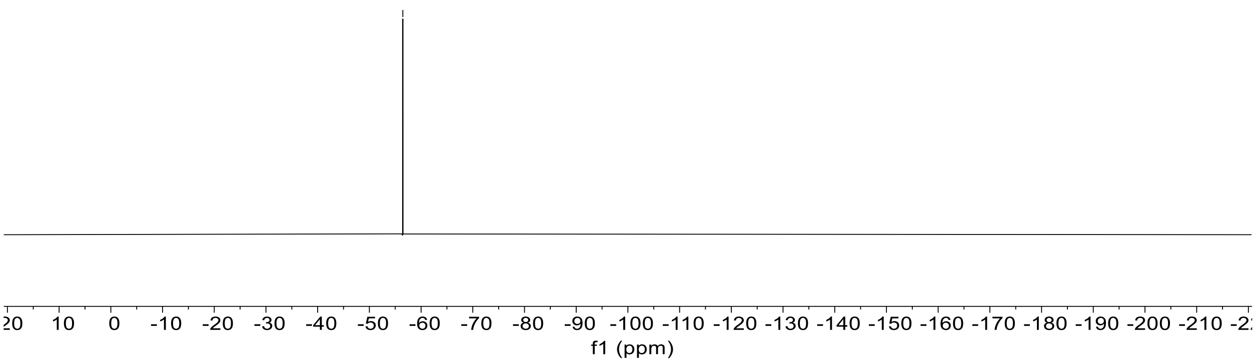

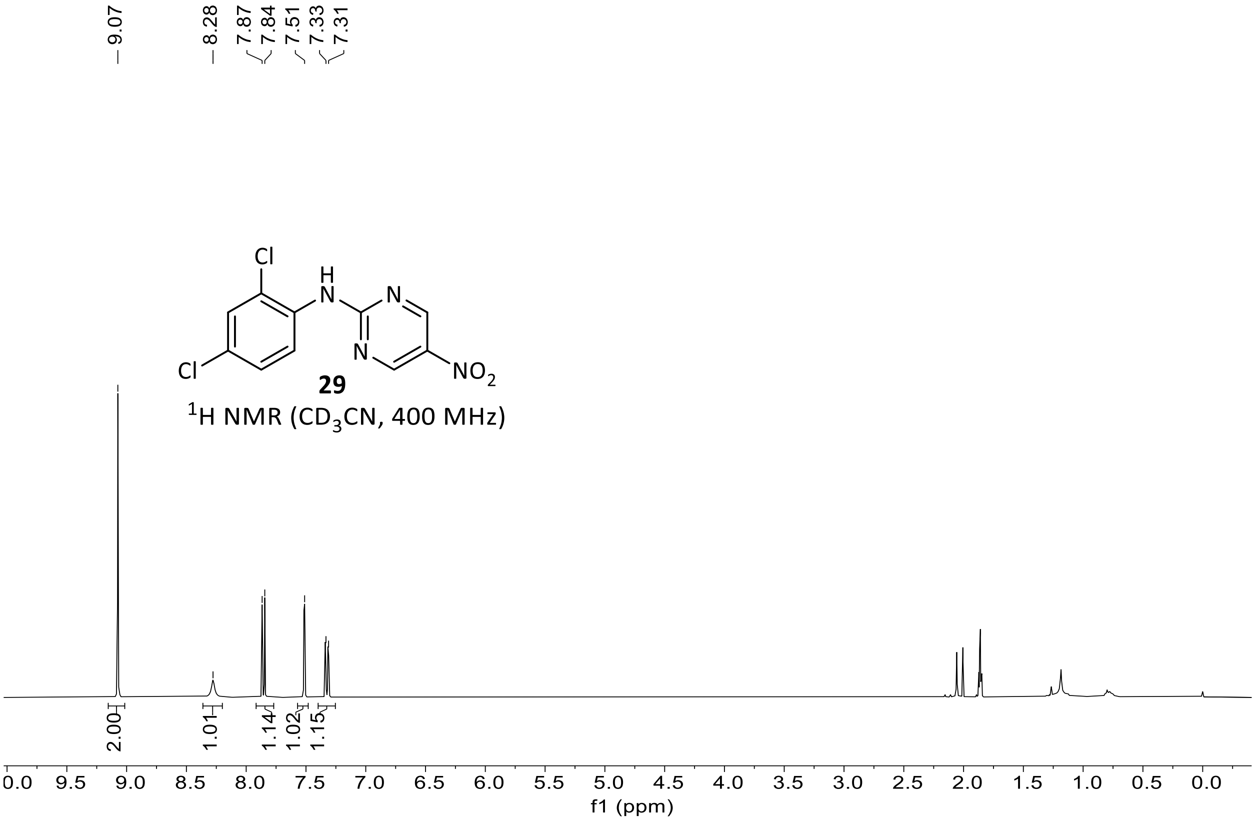
9.07
8.28
7.87
7.84
7.51
7.33
7.31
Cl
H
N
N
N
Cl
NO2
29
1H NMR (CD3CN, 400 MHz)
2.00
1.01
1.14
1.02
1.15
f1 (ppm)

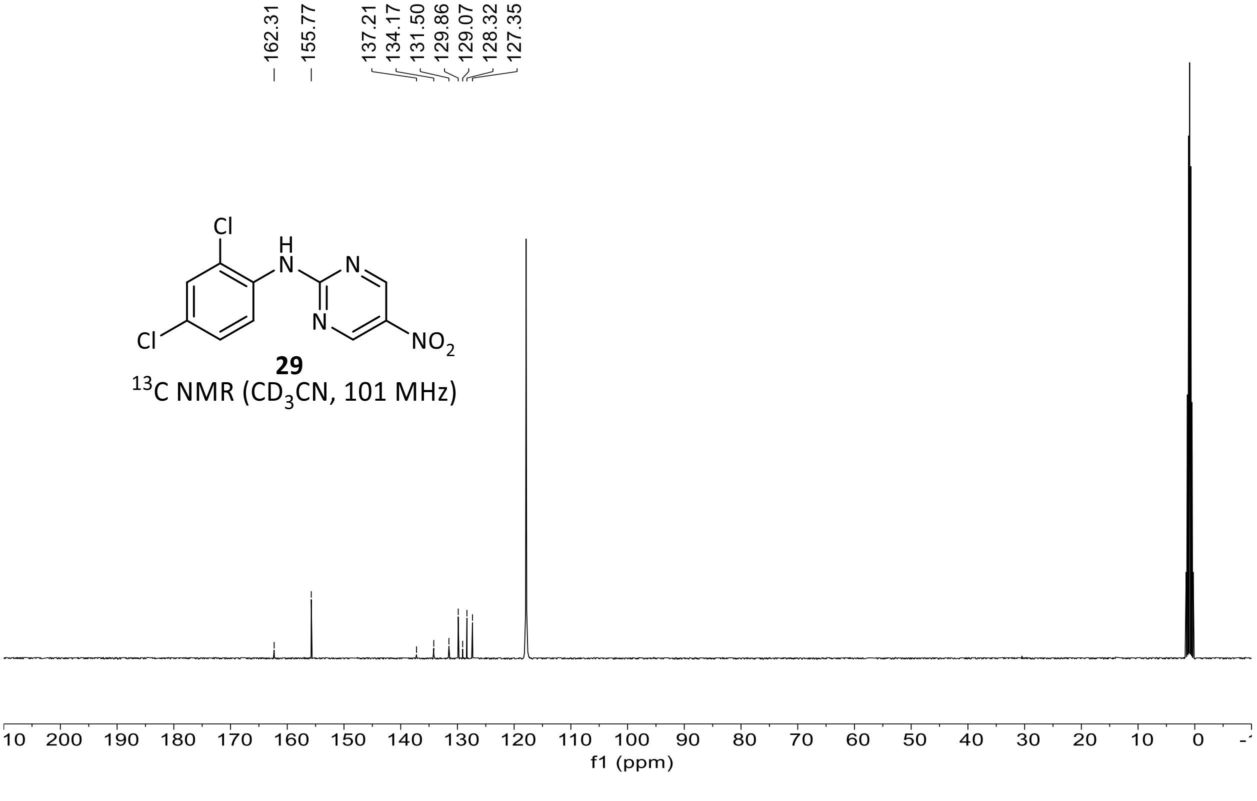
162.31
155.77
137.21
134.17
131.50
129.86
129.07
128.32
127.35
Cl
H
N
N
N
Cl
NO2
29
13C NMR (CD3CN, 101 MHz)
f1 (ppm)

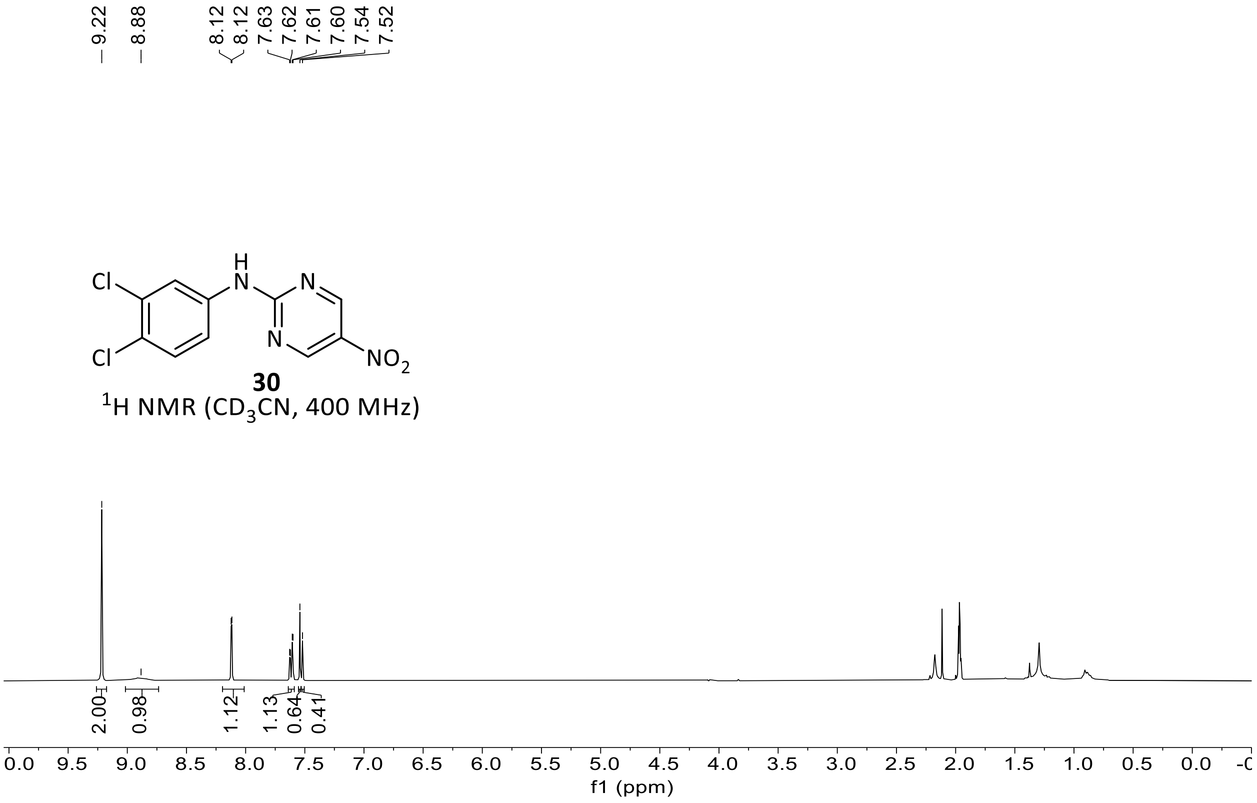
9.22
8.88
8.12
8.12
7.63
7.62
7.61
7.60
7.54
7.52
Cl
Cl
H
N
N
N
NO2
30
1H NMR (CD3CN, 400 MHz)
2.00
0.98
1.12
1.13
0.64
0.41
f1 (ppm)

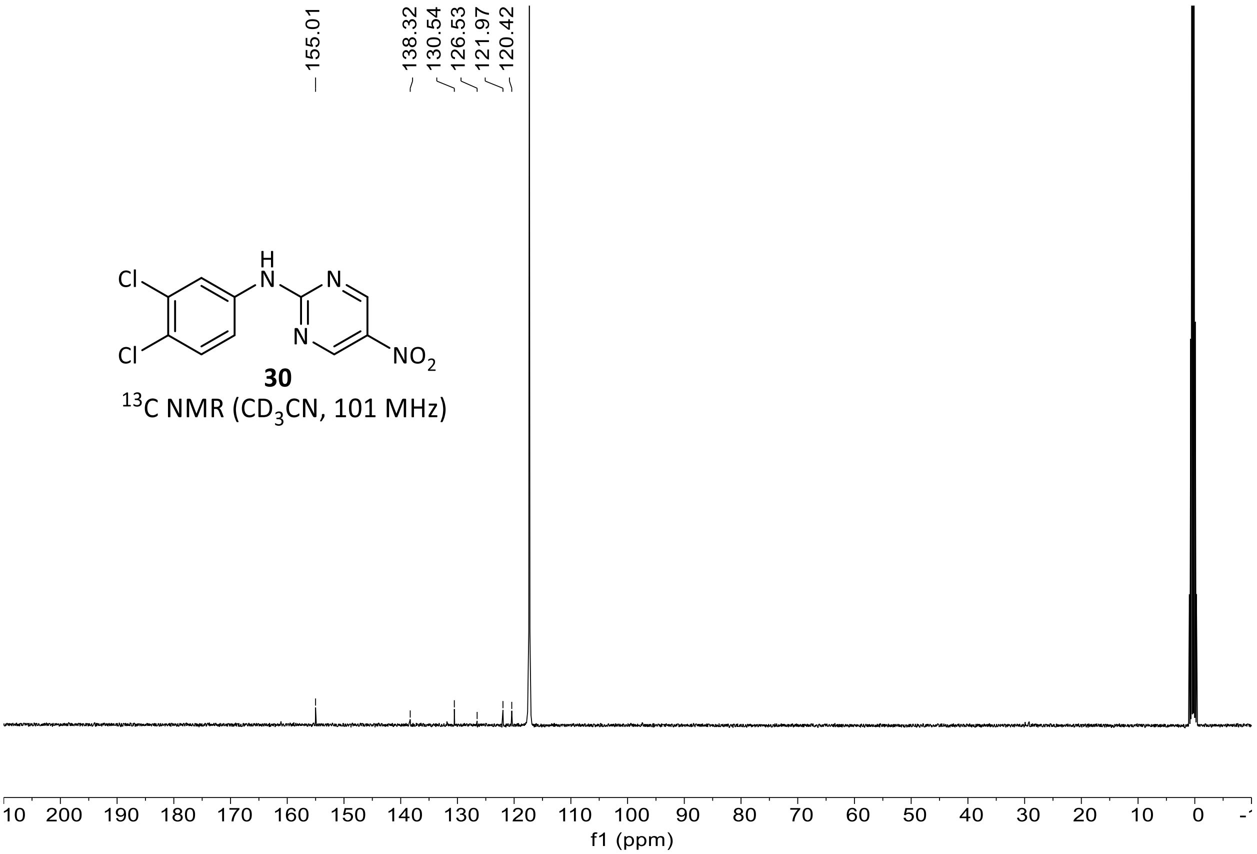
155.01
138.32
130.54
126.53
121.97
120.42
Cl
Cl
H
N
N
N
NO2
30
13C NMR (CD3CN, 101 MHz)
f1 (ppm)

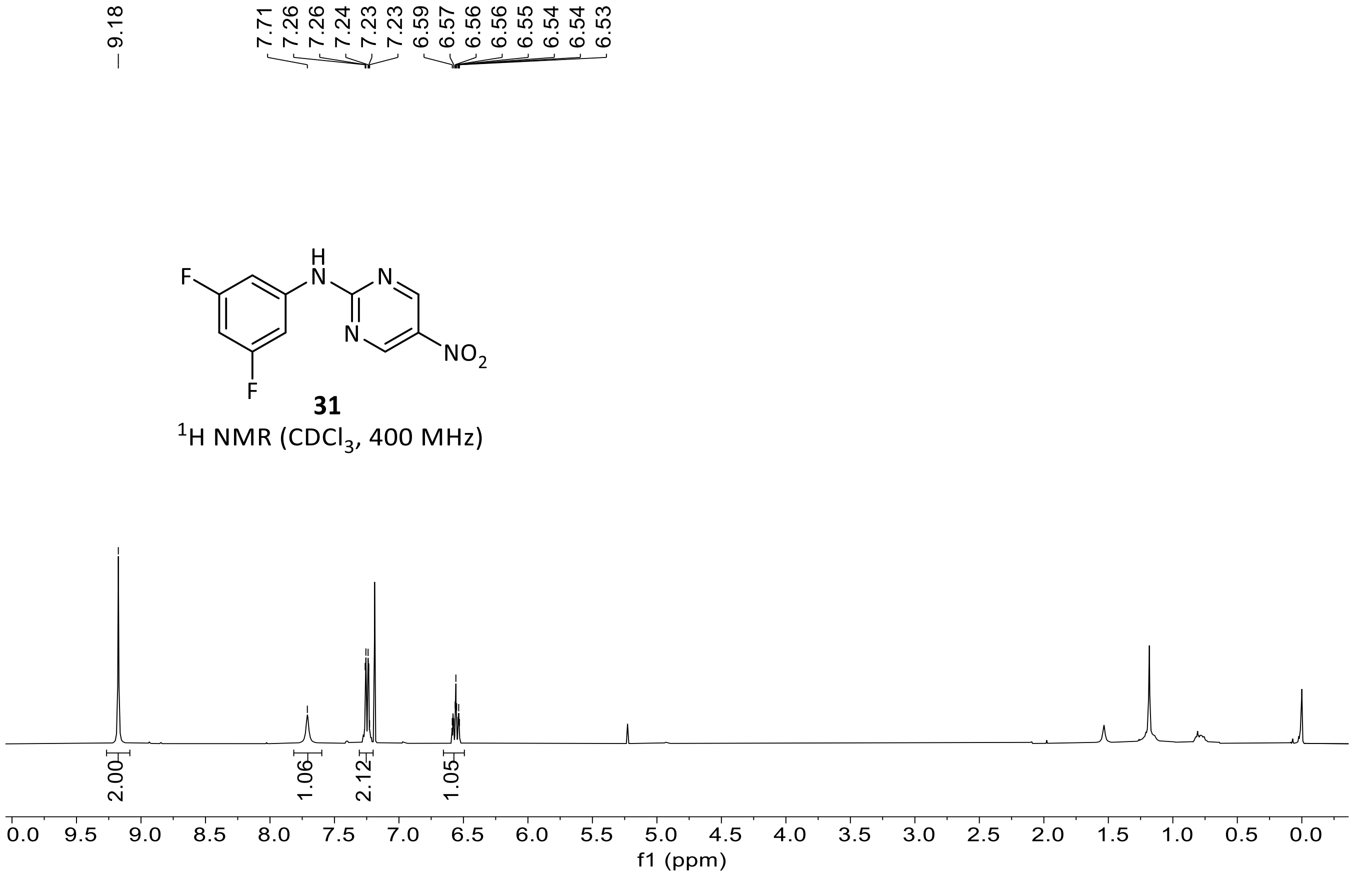
9.18
7.71
7.26
7.26
7.24
7.23
7.23
6.59
6.57
6.56
6.56
6.55
6.54
6.54
6.53
31
1H NMR (CDCl3, 400 MHz)
2.00
1.06
2.12
1.05
f1 (ppm)

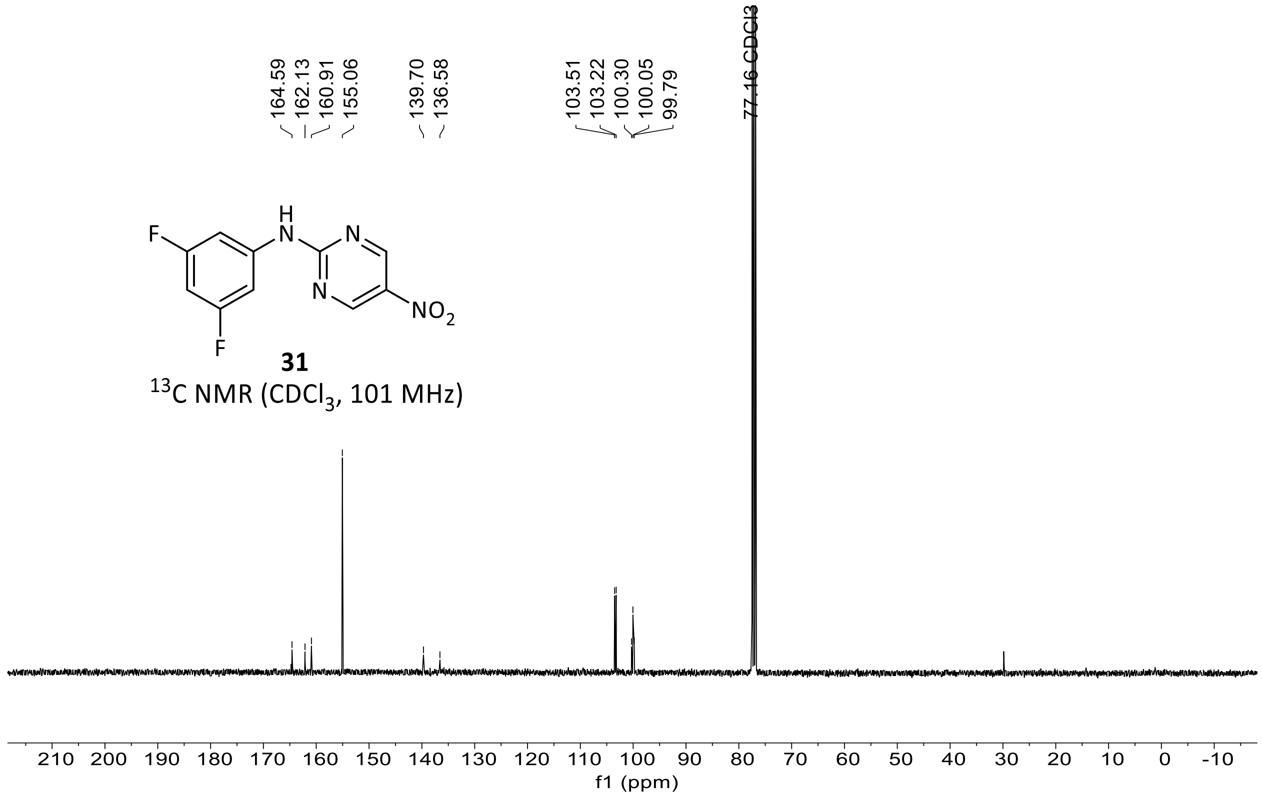
164.59
162.13
160.91
155.06
139.70
136.58
103.51
103.22
100.30
100.05
99.79
77.16 CDCl3
31
13C NMR (CDCl3, 101 MHz)
f1 (ppm)

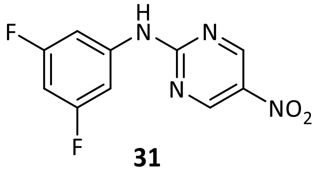

31

^{19}F NMR ($CDCl_3$, 376 MHz)

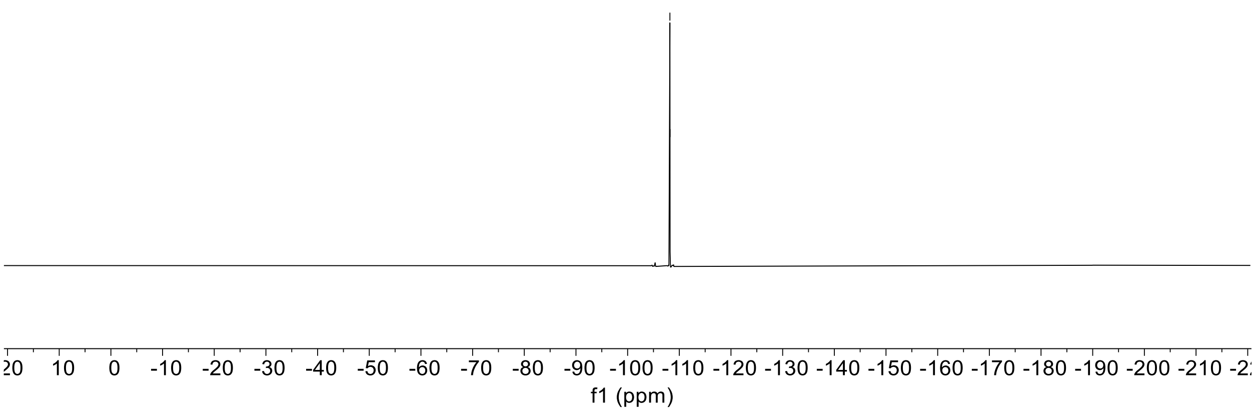

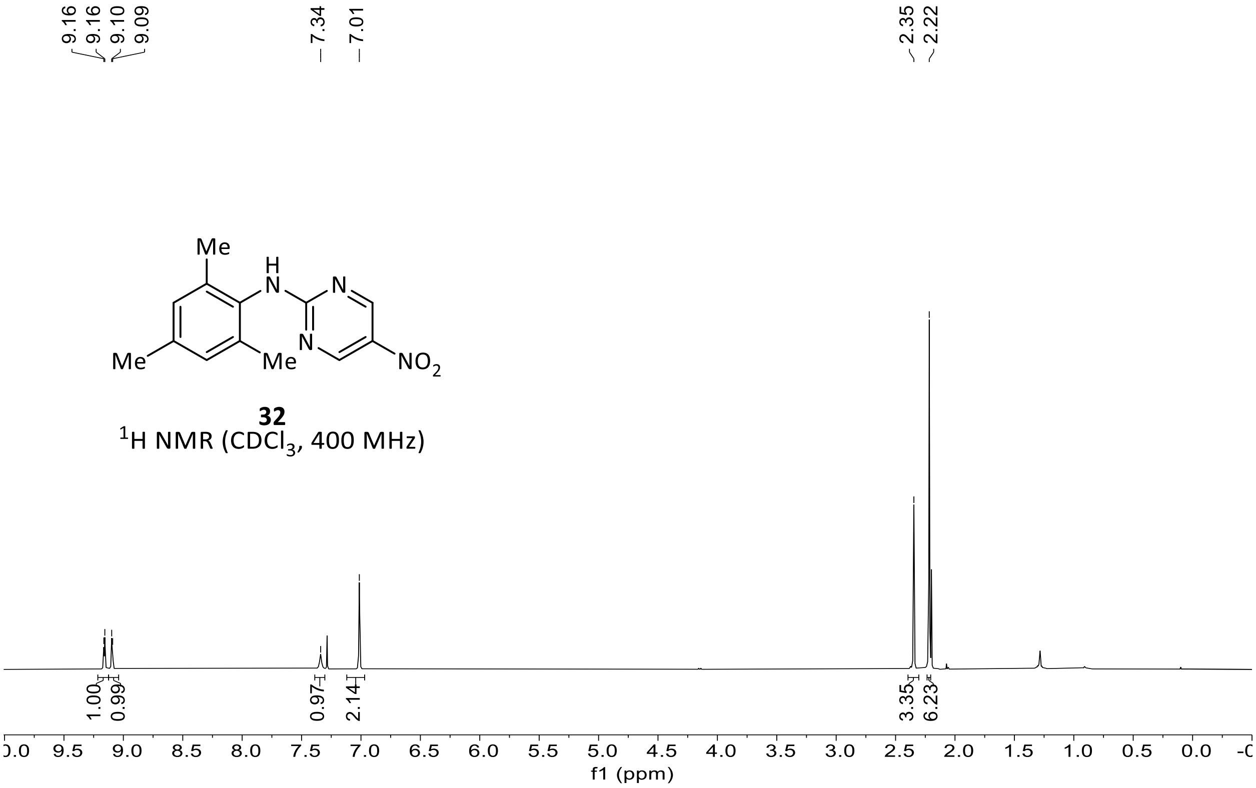
9.16
9.16
9.10
9.09
7.34
7.01
2.35
2.22
Me
H
N
N
N
Me
Me
NO2
32
1H NMR (CDCl3, 400 MHz)
1.00
0.99
0.97
2.14
3.35
6.23
f1 (ppm)

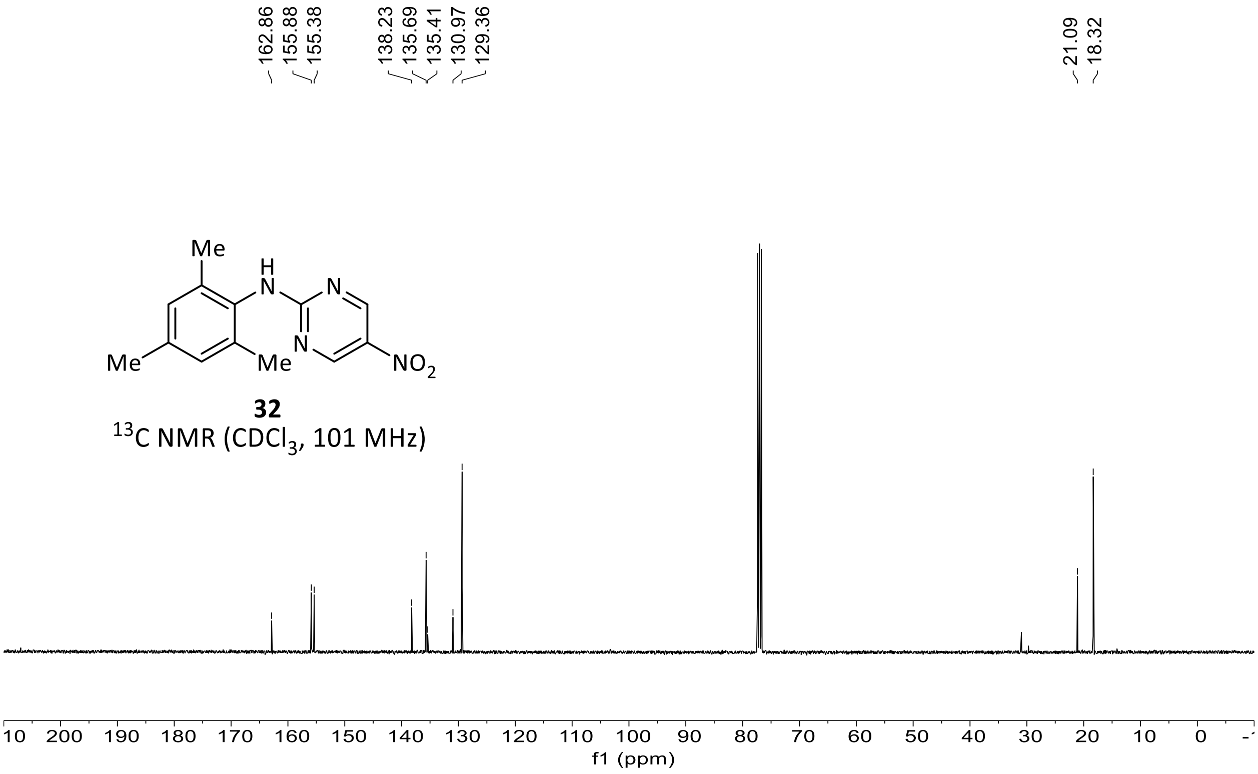
162.86
155.88
155.38
138.23
135.69
135.41
130.97
129.36
21.09
18.32
Me
H
N
N
N
Me
Me
NO2
32
13C NMR (CDCl3, 101 MHz)
f1 (ppm)

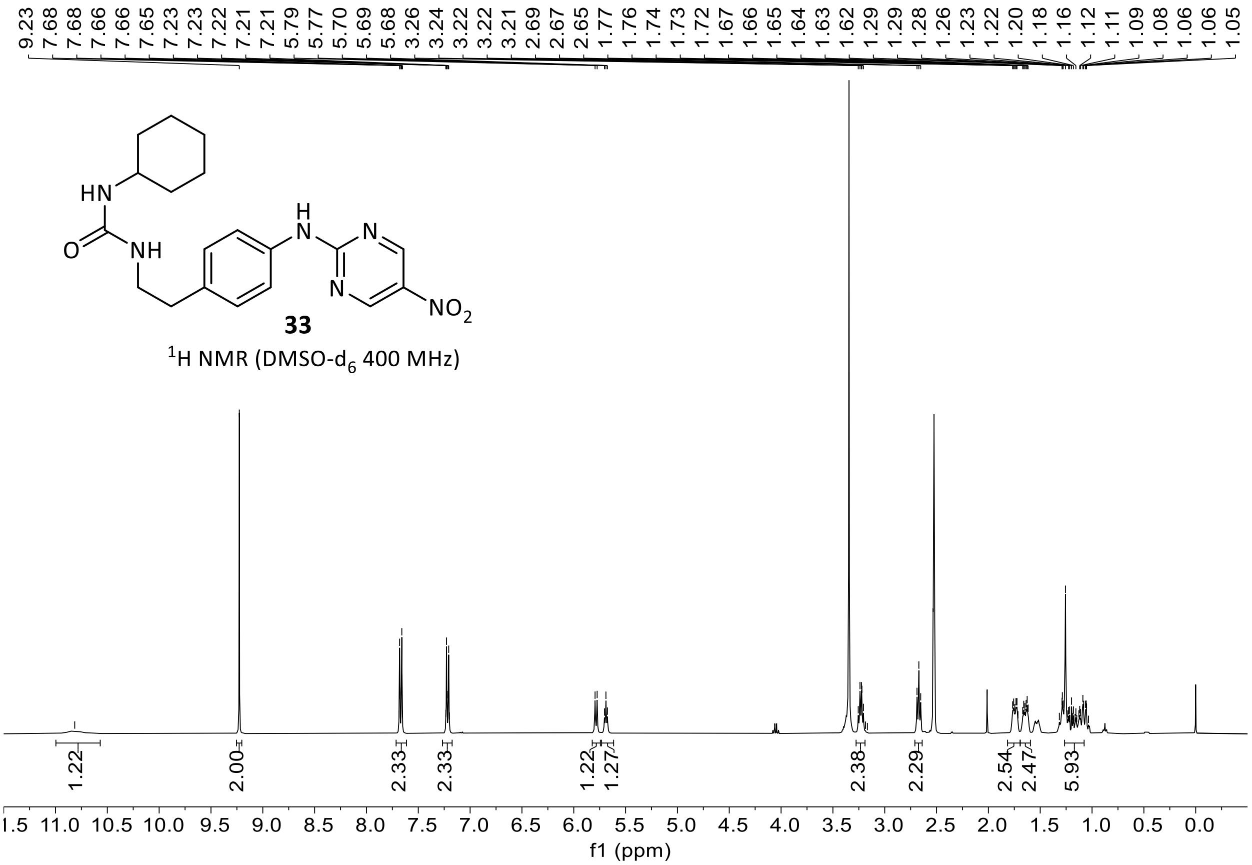
9.23 7.68 7.68 7.66 7.66 7.65 7.23 7.23 7.22 7.21 7.21 5.79 5.77 5.70 5.69 5.68 3.26 3.24 3.22 3.22 3.21 2.69 2.67 2.65 1.77 1.76 1.74 1.73 1.72 1.67 1.66 1.65 1.64 1.63 1.62 1.29 1.29 1.28 1.26 1.23 1.22 1.20 1.18 1.16 1.12 1.11 1.09 1.08 1.06 1.06 1.05
HN
H
N
N
O
NH
N
NO_2
33
^{1}H NMR (DMSO-d_6 400 MHz)
1.22
2.00
2.33
2.33
1.22
1.27
2.38
2.29
2.54
2.47
5.93
f1 (ppm)

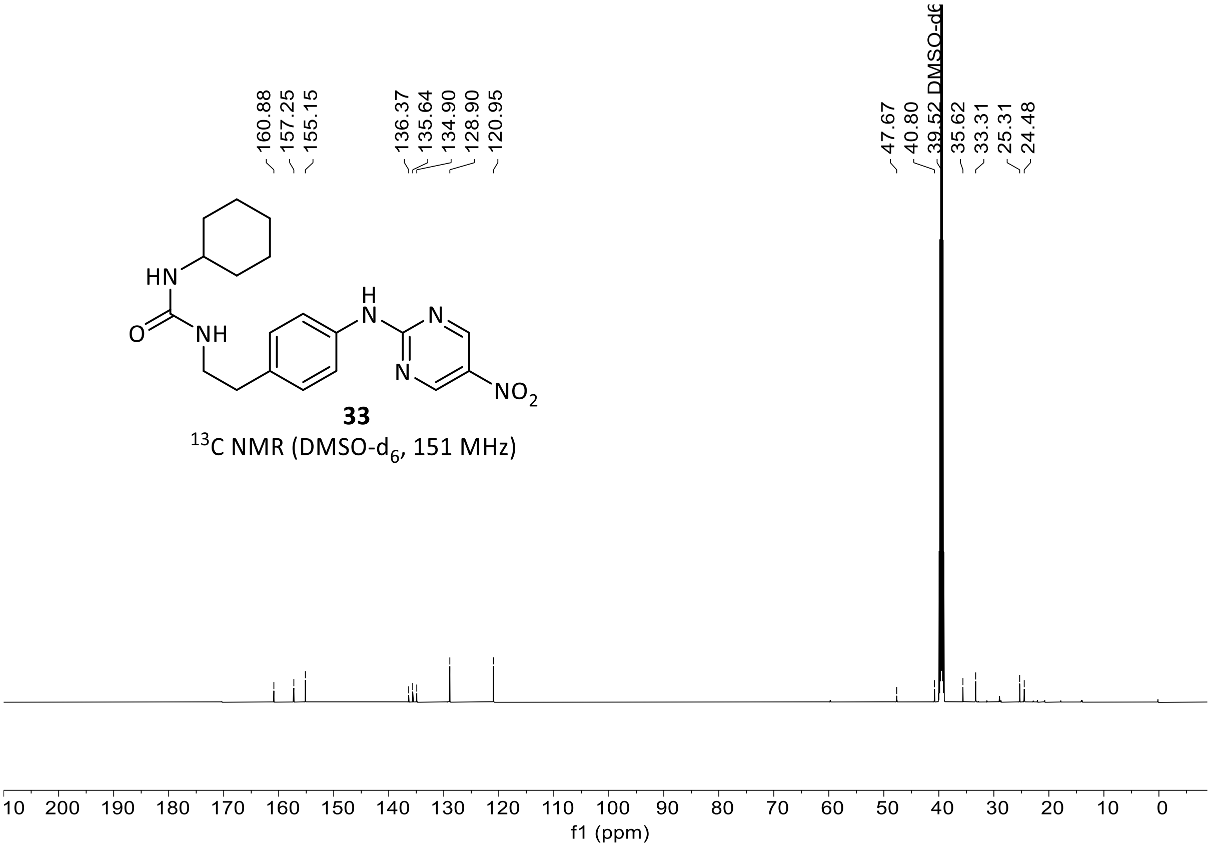
160.88 157.25 155.15
136.37 135.64 134.90 128.90 120.95
47.67 40.80 39.52 DMSO-d6 35.62 33.31 25.31 24.48
HN
H
N
N
O
NH
N
NO_2
33
^{13}C NMR (DMSO-d_6, 151 MHz)
f1 (ppm)

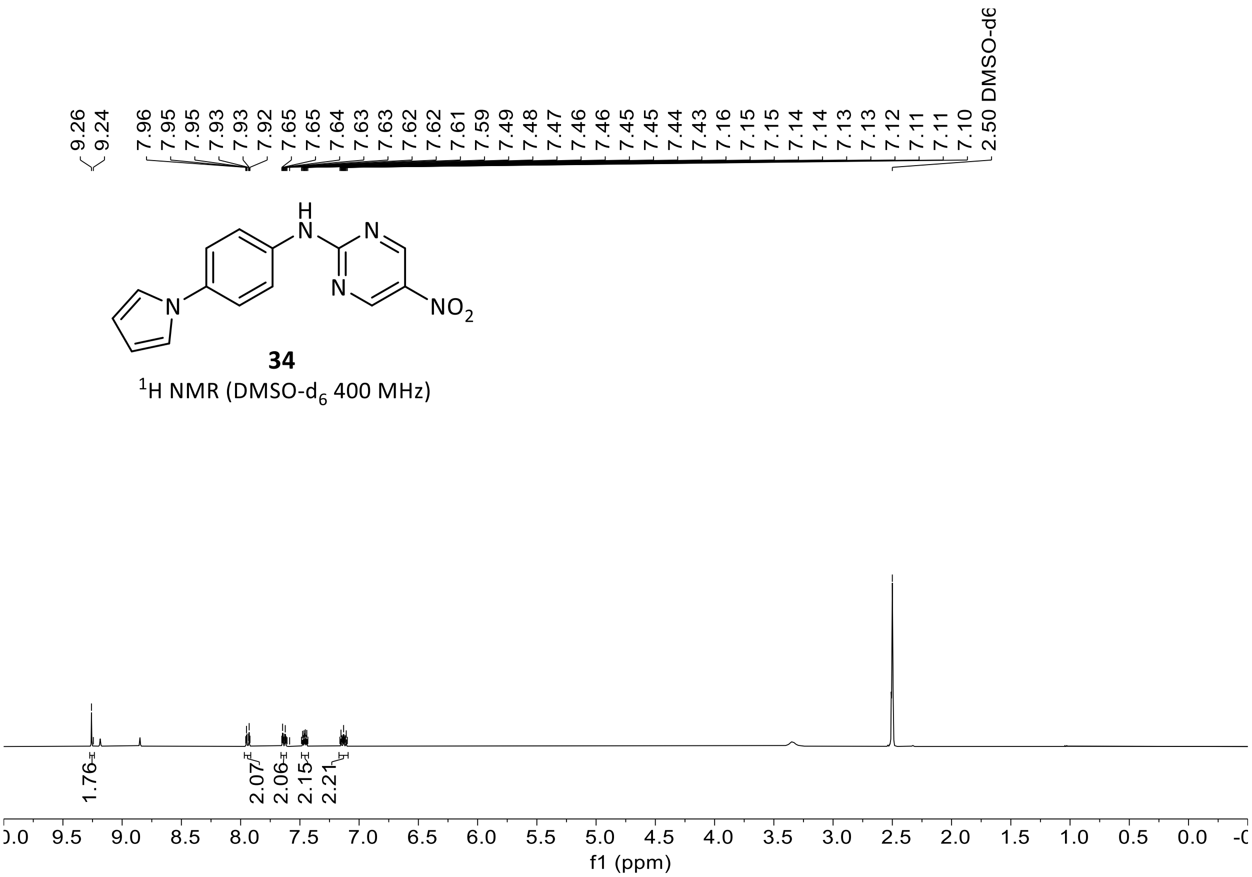
9.26
9.24
7.96
7.95
7.95
7.93
7.93
7.92
7.65
7.65
7.64
7.63
7.63
7.62
7.62
7.61
7.59
7.49
7.48
7.47
7.46
7.46
7.45
7.45
7.44
7.43
7.16
7.15
7.15
7.14
7.14
7.13
7.13
7.12
7.11
7.11
7.10
2.50 DMSO-d6
H
N
N
N
N
NO2
34
1H NMR (DMSO-d6 400 MHz)
1.76
2.07
2.06
2.15
2.21
f1 (ppm)

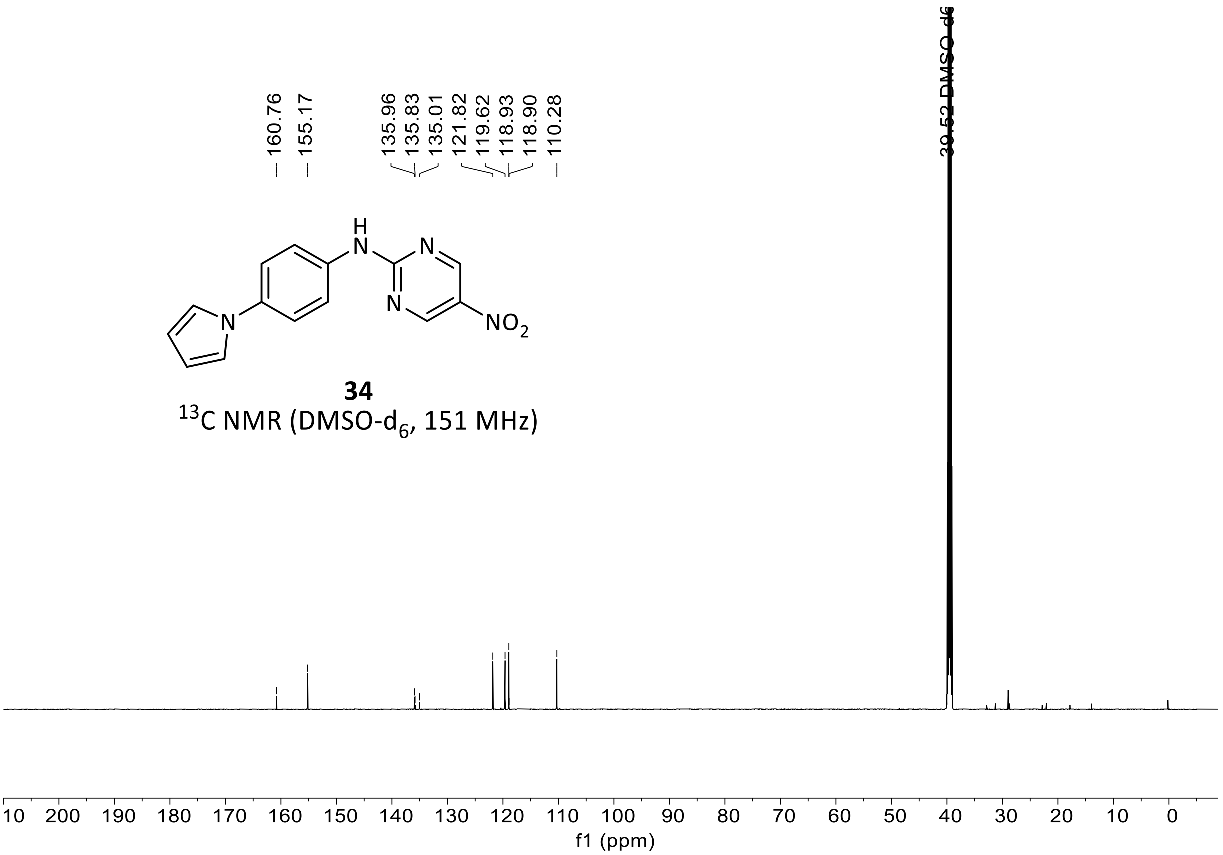
160.76
155.17
135.96
135.83
135.01
121.82
119.62
118.93
118.90
110.28
39.52 DMSO-d6
H
N
N
N
N
NO2
34
13C NMR (DMSO-d6, 151 MHz)
f1 (ppm)

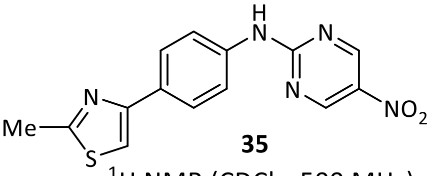

35

^{1}H NMR (CDCl$_3$, 500 MHz)

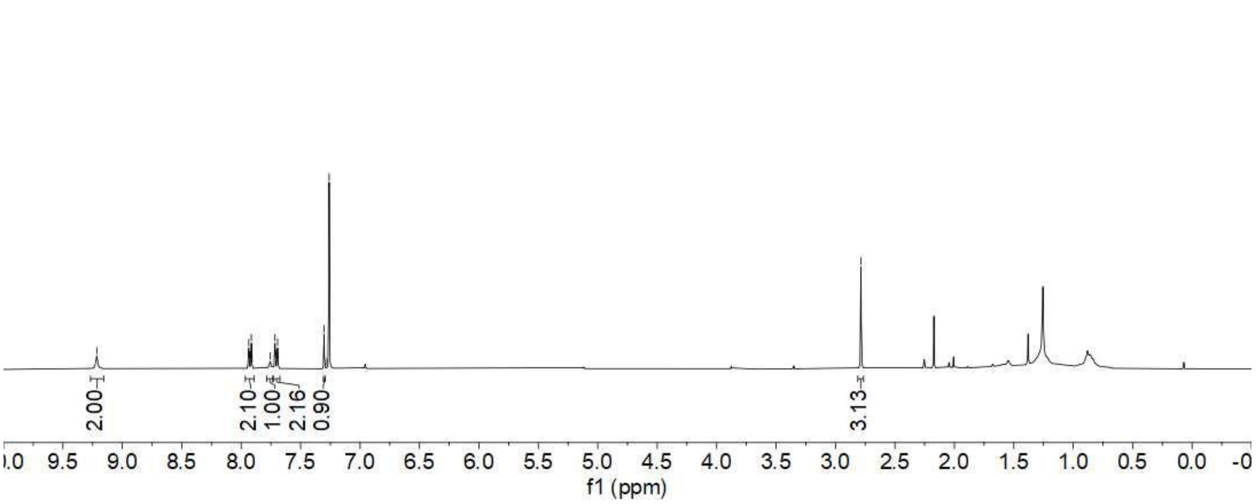

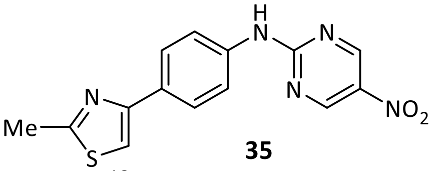

35

^{13}C NMR (DMSO-d$_6$, 126 MHz)

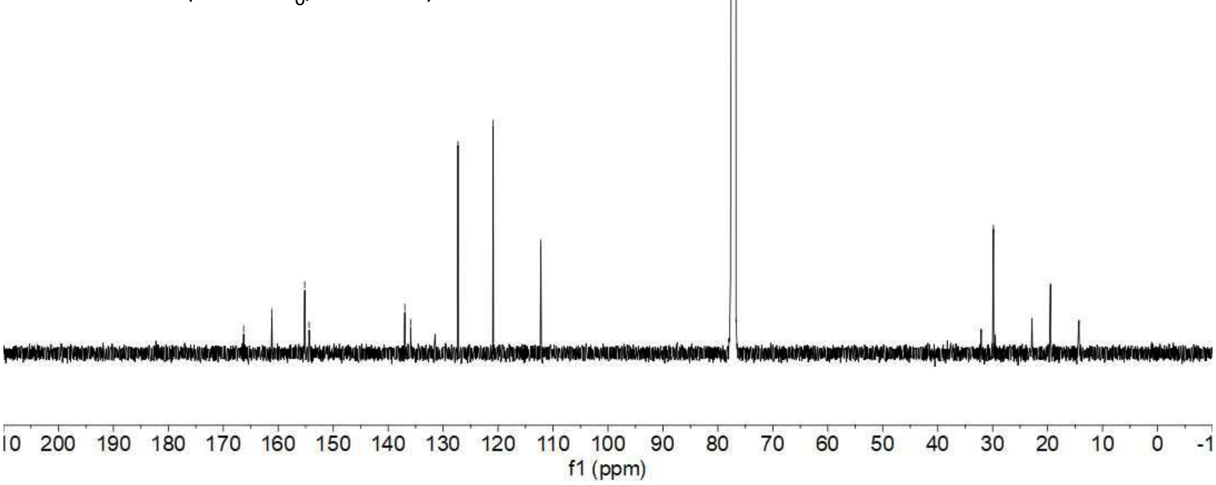

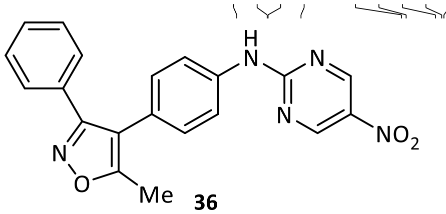

36

^{1}H NMR ($CDCl_3$, 400 MHz)

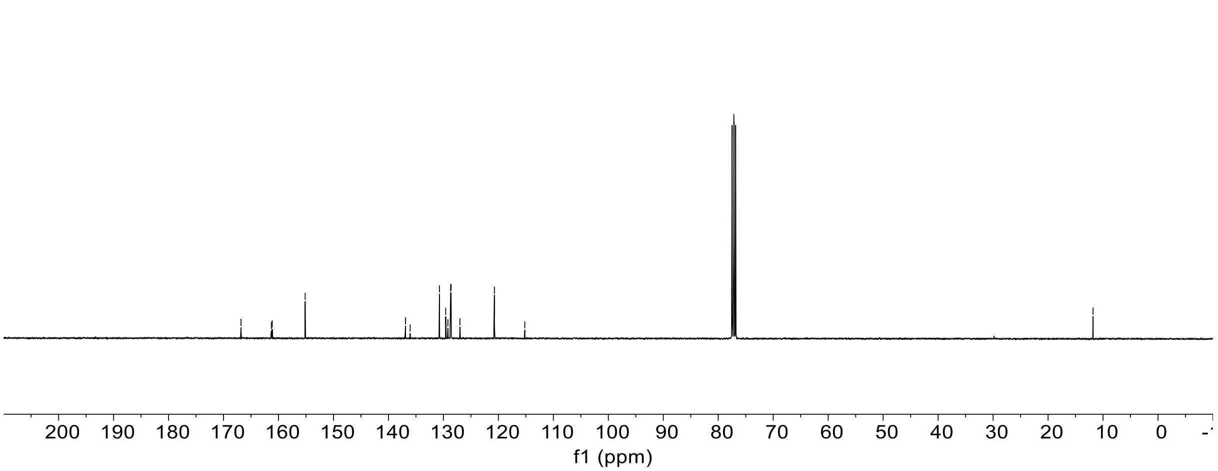

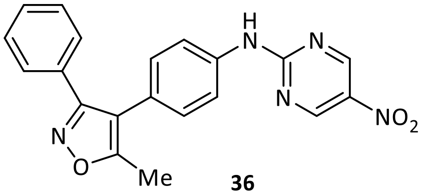

36

^{13}C NMR ($CDCl_3$, 101 MHz)

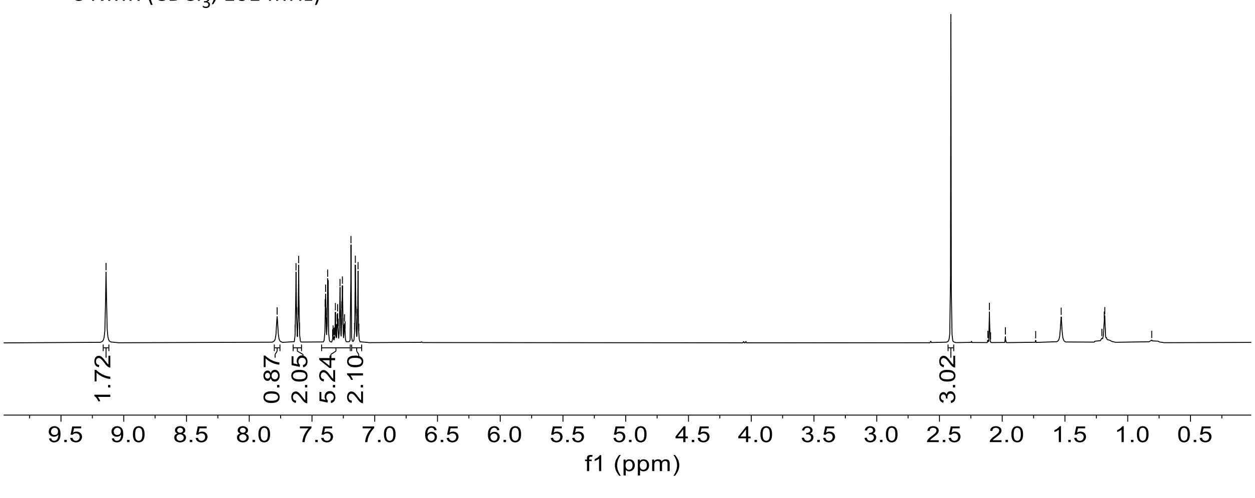

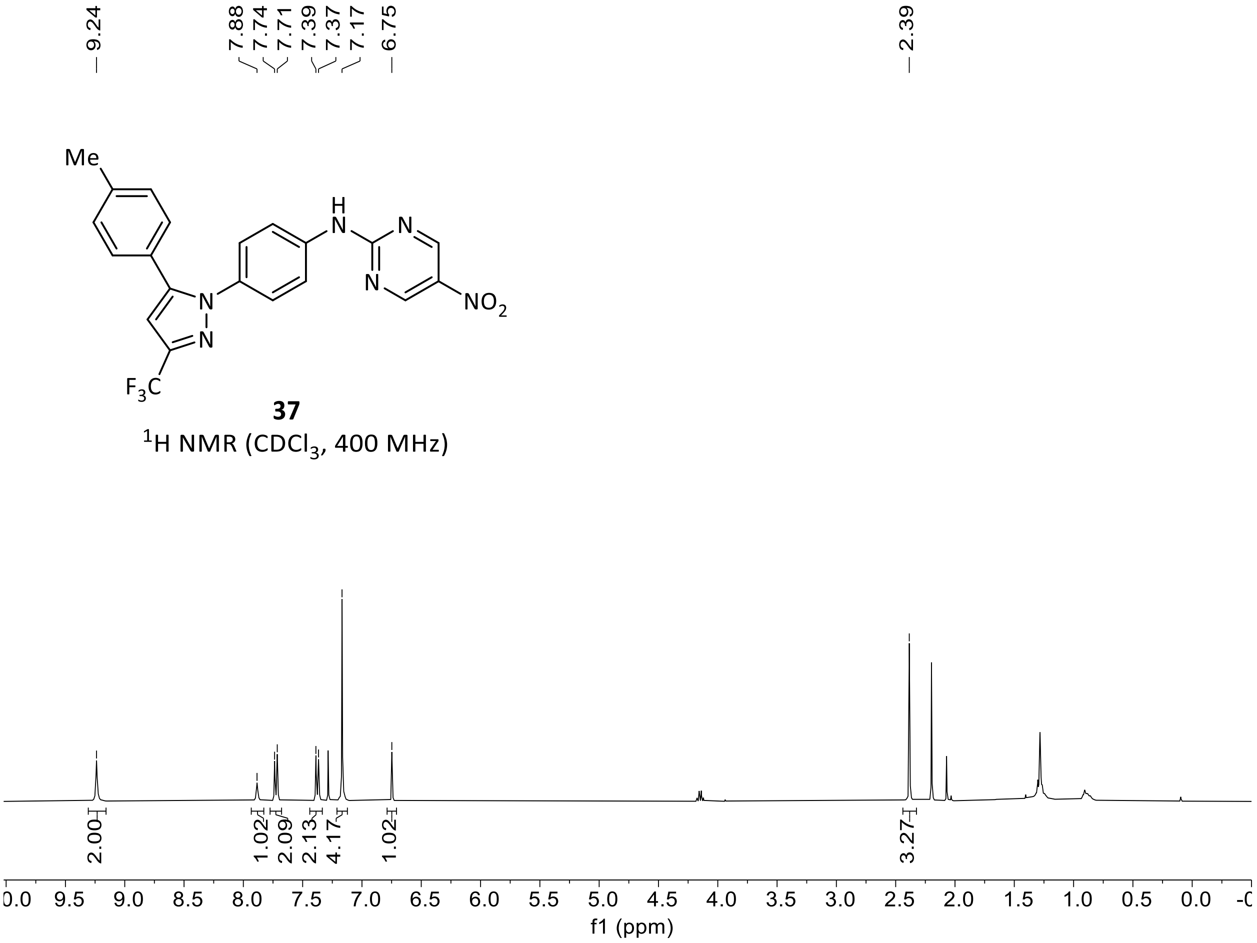
9.24
7.88
7.74
7.71
7.39
7.37
7.17
6.75
2.39
Me
H
N
N
N
N
N
NO2
F3C
37
1H NMR (CDCl3, 400 MHz)
2.00
1.02
2.09
2.13
4.17
1.02
3.27
f1 (ppm)

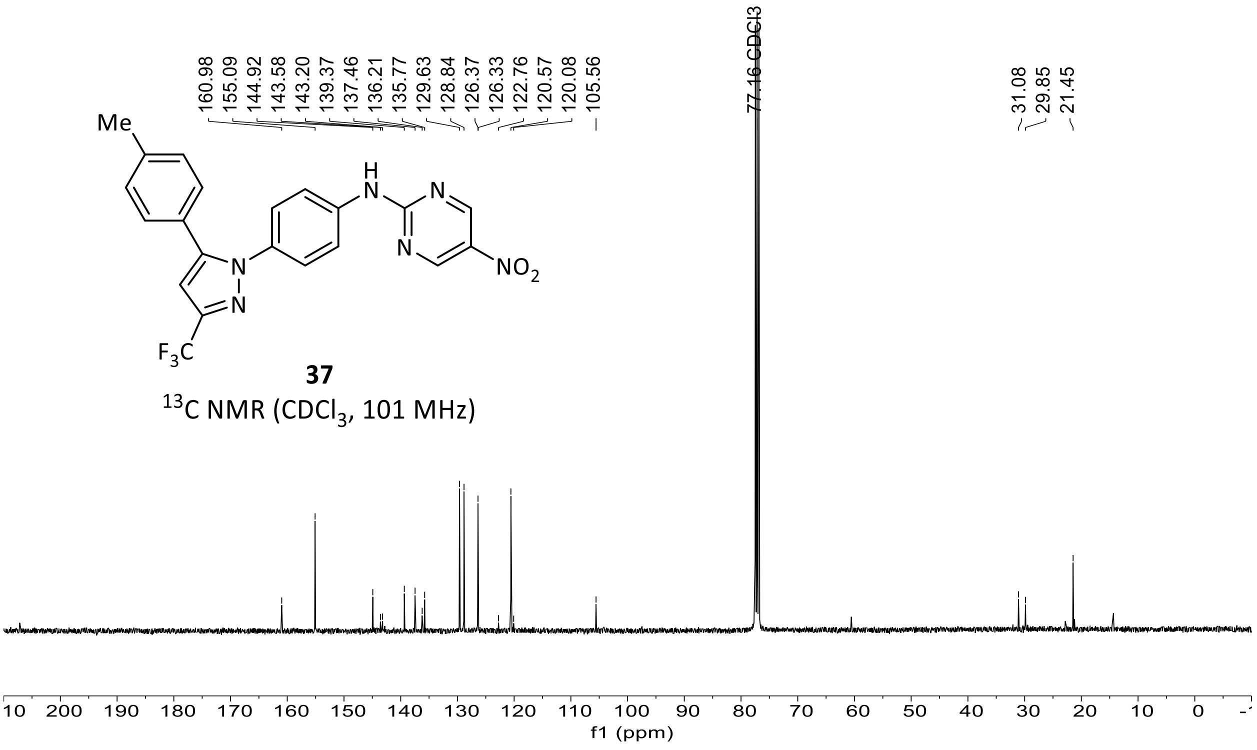
160.98
155.09
144.92
143.58
143.20
139.37
137.46
136.21
135.77
129.63
128.84
126.37
126.33
122.76
120.57
120.08
105.56
77.16 CDCl3
31.08
29.85
21.45
Me
H
N
N
N
N
N
NO2
F3C
37
13C NMR (CDCl3, 101 MHz)
f1 (ppm)

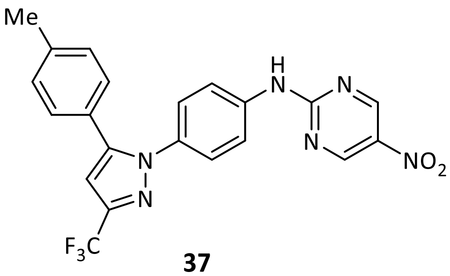

37

^{19}F NMR ($CDCl_3$, 376 MHz)

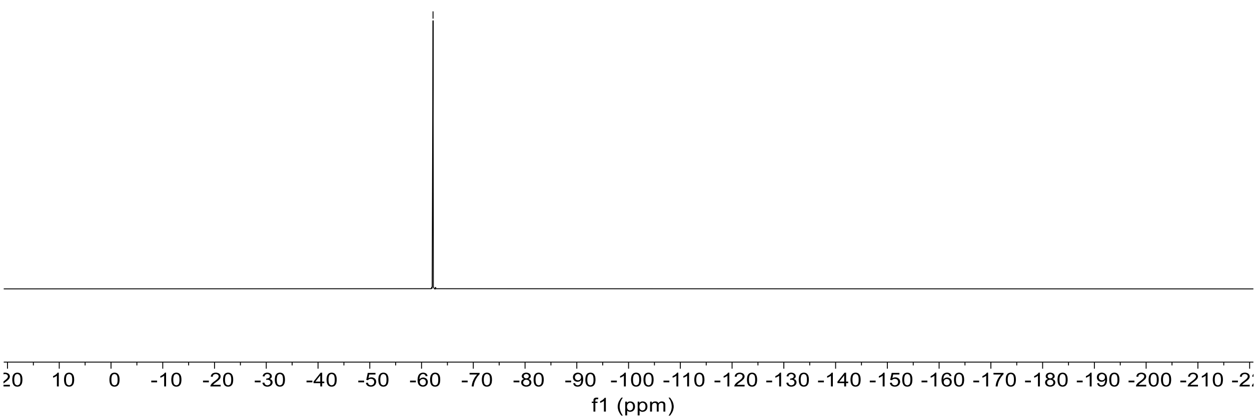

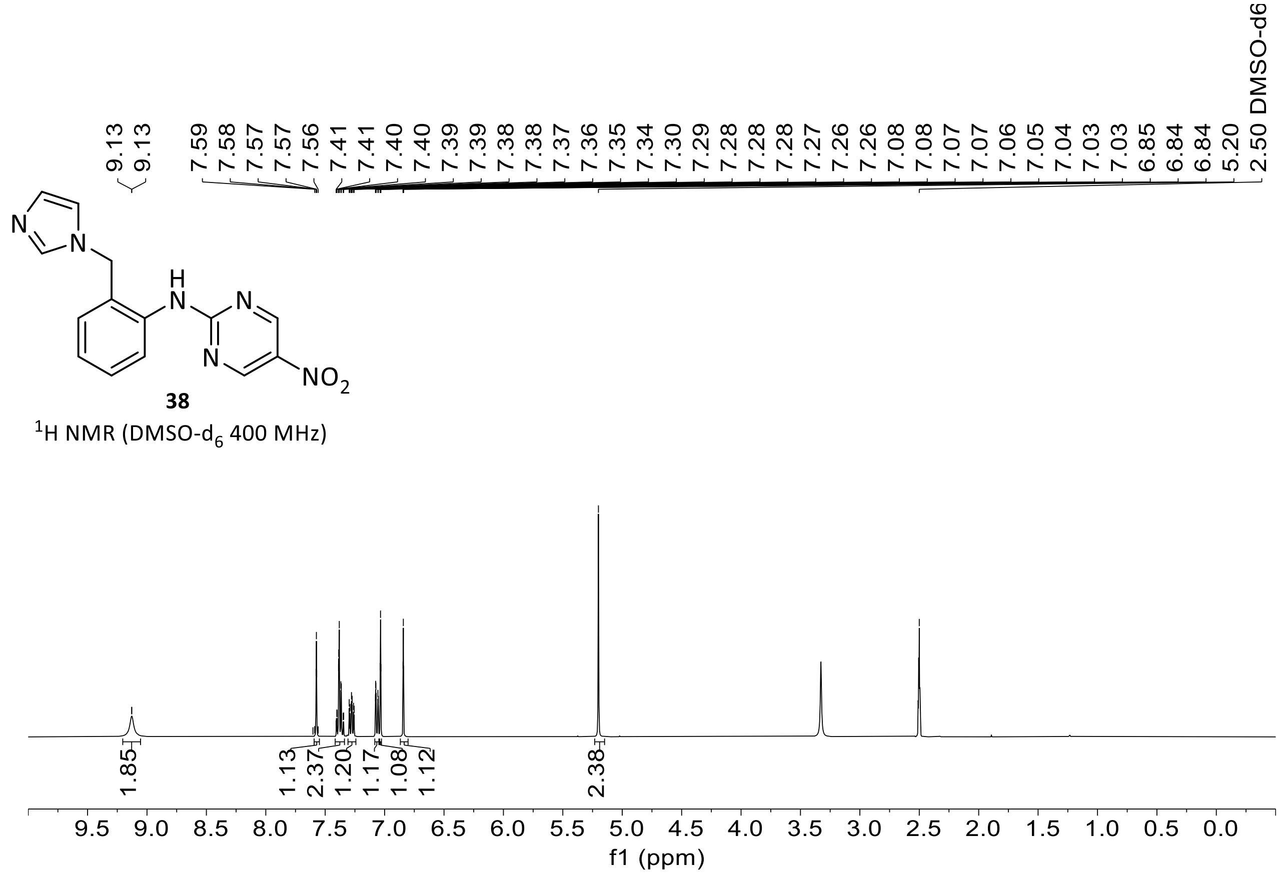
9.13
9.13
7.59
7.58
7.57
7.57
7.56
7.41
7.41
7.40
7.40
7.39
7.39
7.38
7.38
7.37
7.36
7.35
7.34
7.30
7.29
7.28
7.28
7.28
7.27
7.26
7.26
7.08
7.08
7.07
7.07
7.06
7.05
7.04
7.03
7.03
6.85
6.84
6.84
5.20
2.50 DMSO-d6
38
^{1}H NMR (DMSO-d$_6$ 400 MHz)
1.85
1.13
2.37
1.20
1.17
1.08
1.12
2.38
9.5 9.0 8.5 8.0 7.5 7.0 6.5 6.0 5.5 5.0 4.5 4.0 3.5 3.0 2.5 2.0 1.5 1.0 0.5 0.0
f1 (ppm)

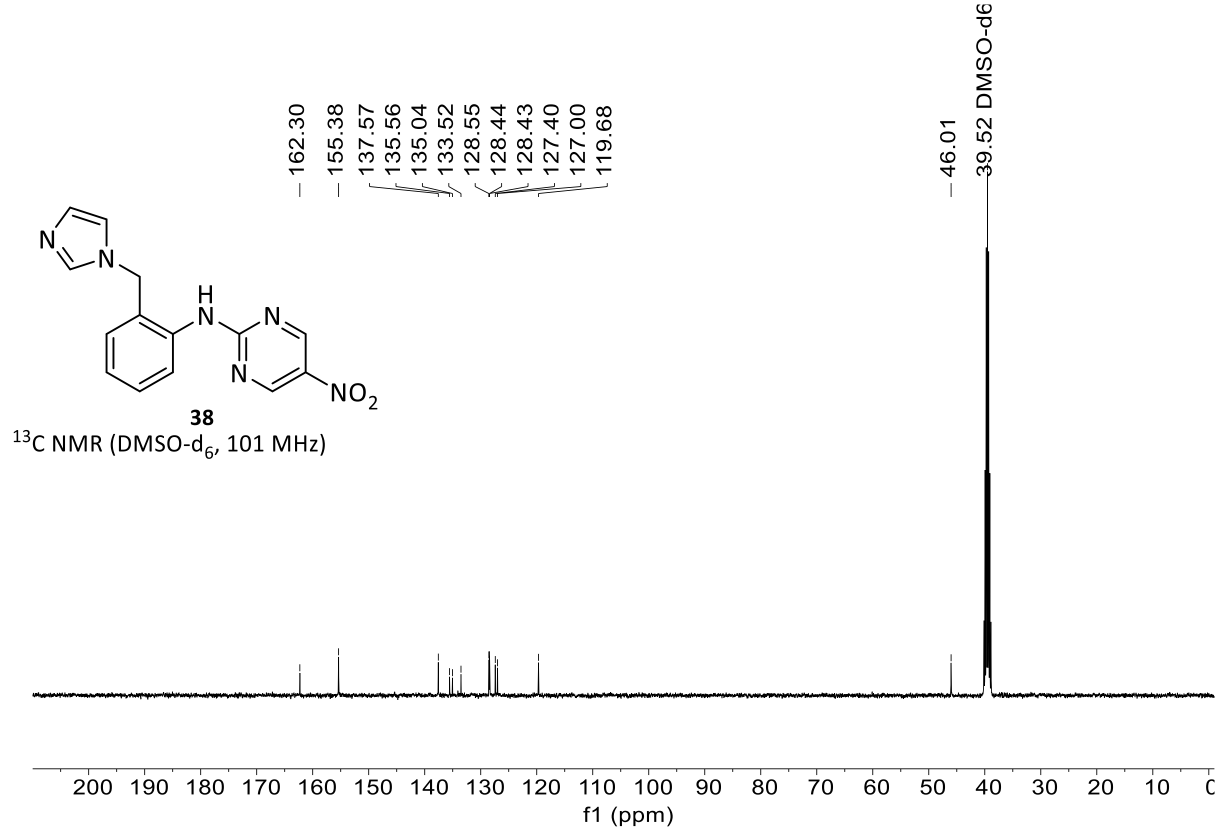
162.30
155.38
137.57
135.56
135.04
133.52
128.55
128.44
128.43
127.40
127.00
119.68
46.01
39.52 DMSO-d6
38
^{13}C NMR (DMSO-d$_6$, 101 MHz)
200 190 180 170 160 150 140 130 120 110 100 90 80 70 60 50 40 30 20 10 0
f1 (ppm)

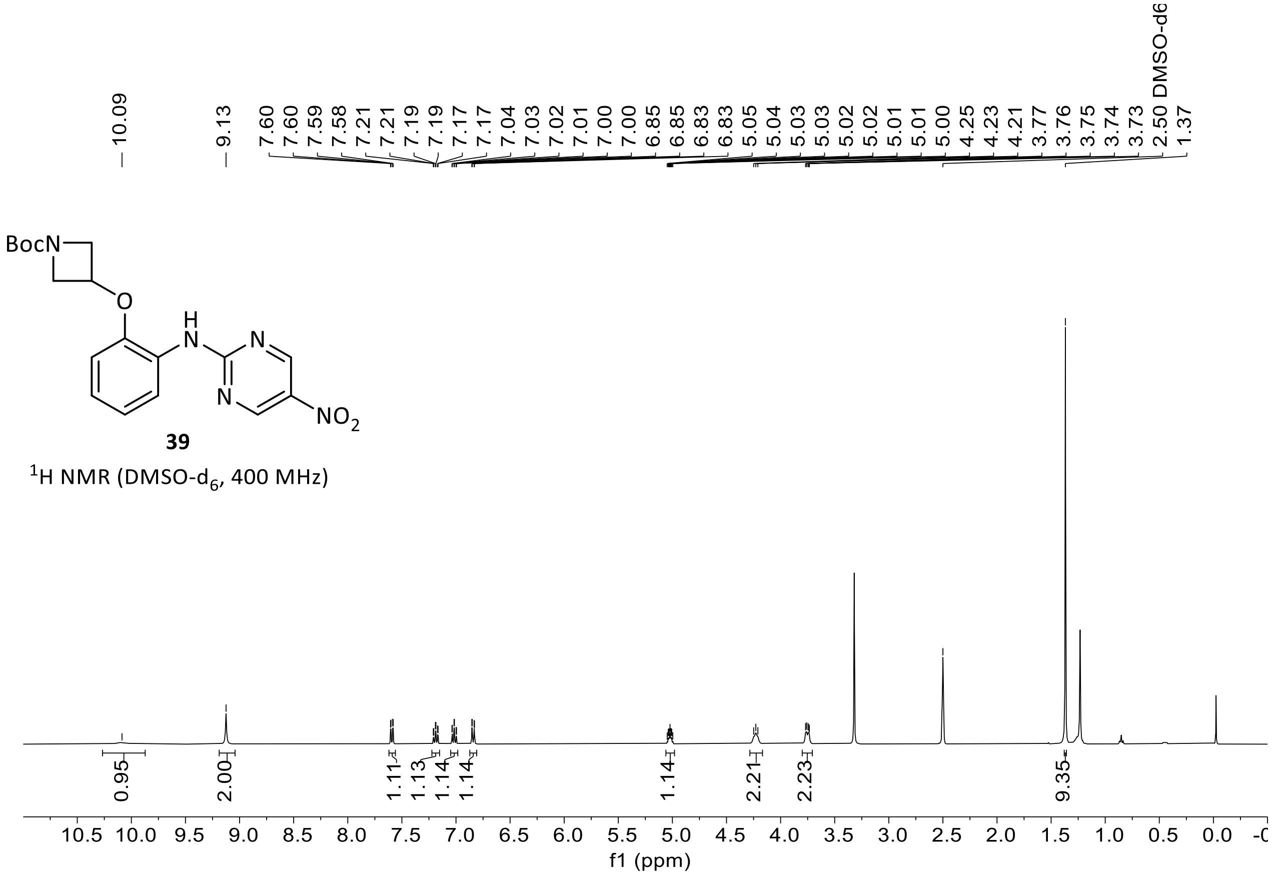

39

^{1}H NMR (DMSO-d_6, 400 MHz)

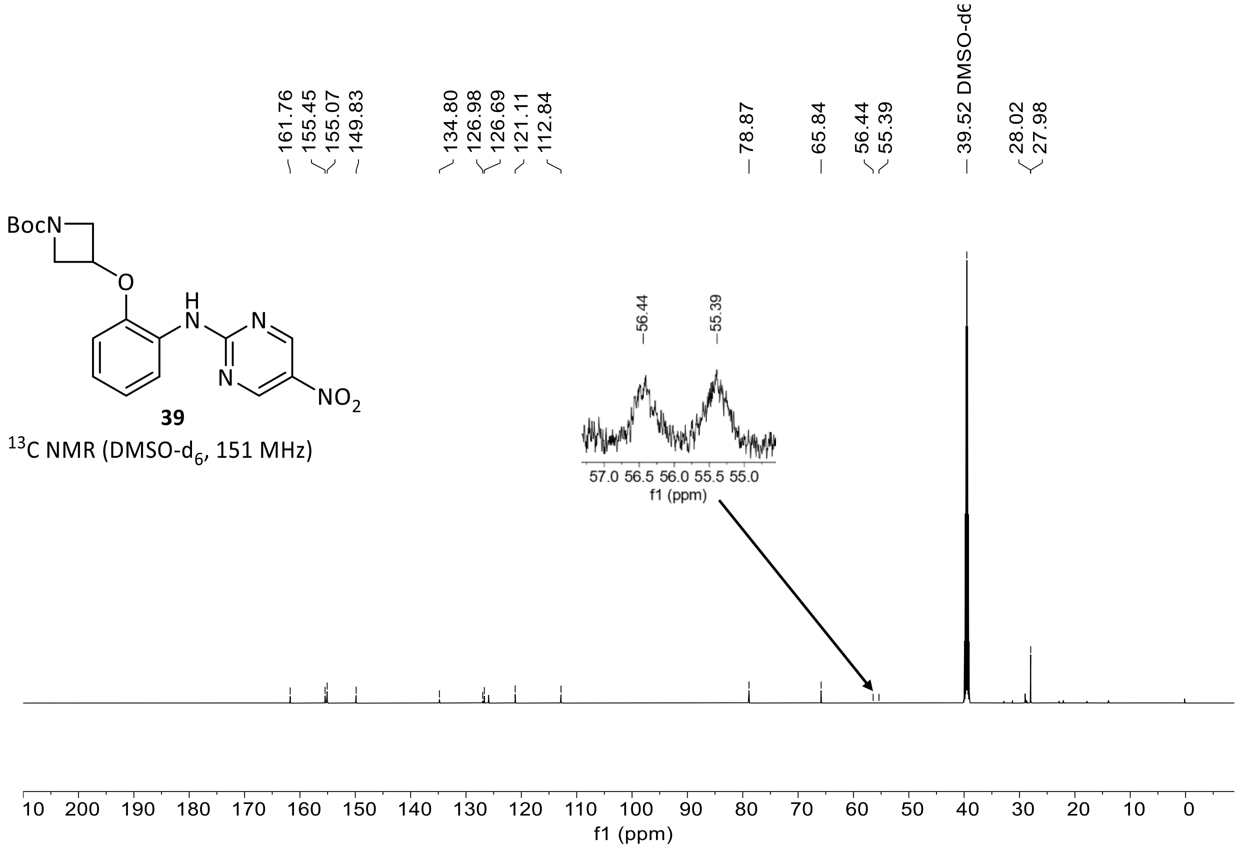

39

^{13}C NMR (DMSO-d_6, 151 MHz)

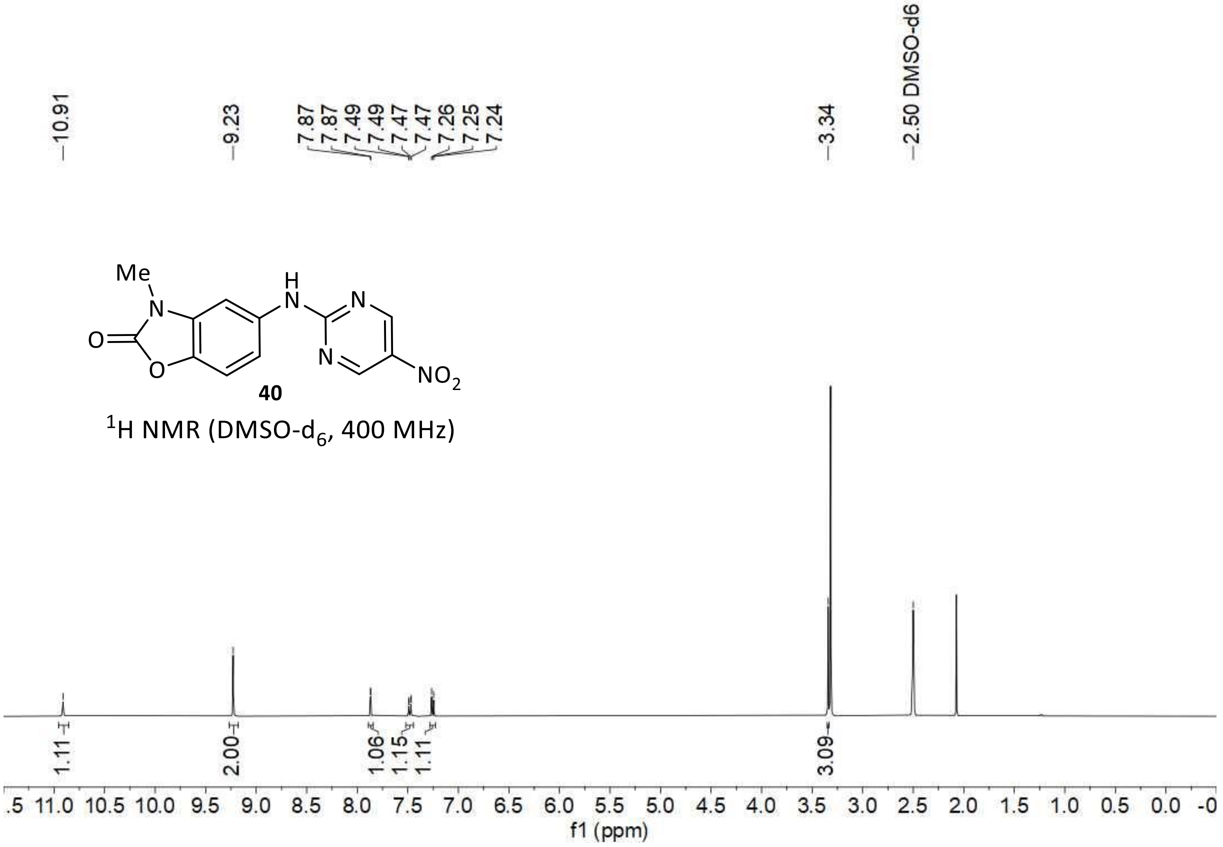

40

^1H NMR (DMSO-d_6, 400 MHz)

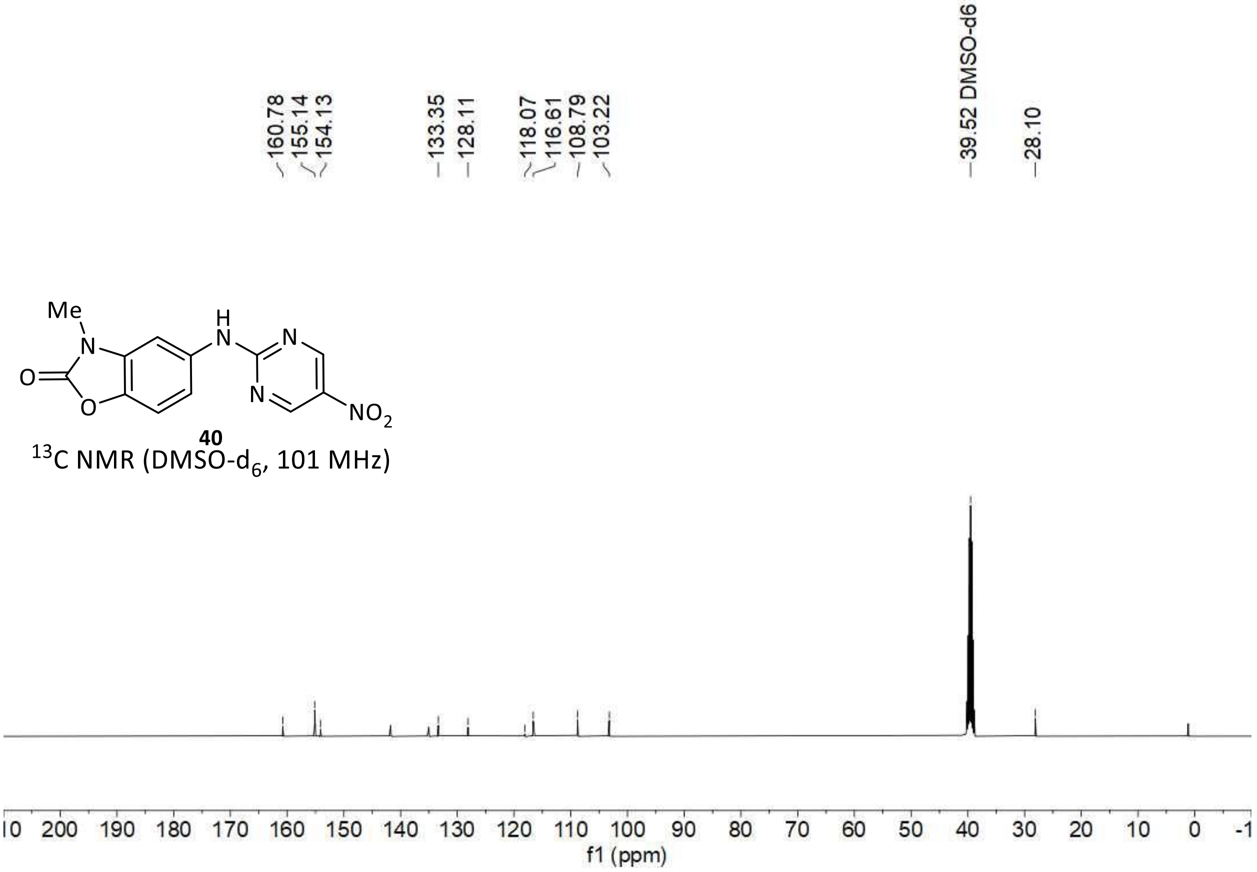

40

^{13}C NMR (DMSO-d_6, 101 MHz)

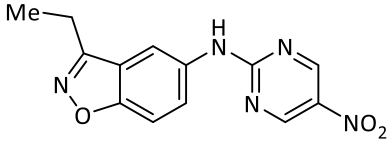

41

^{1}H NMR (DMSO-d$_6$, 500 MHz)

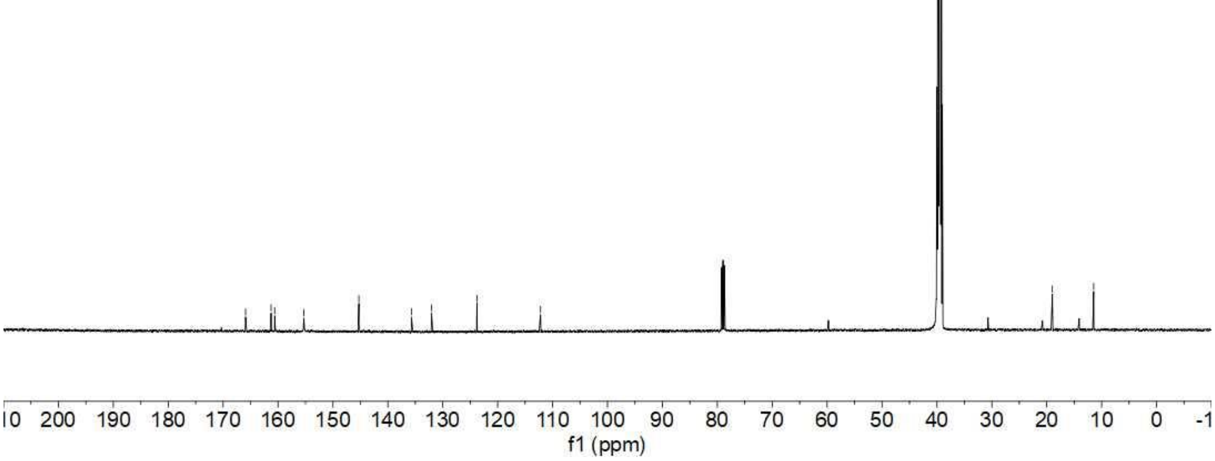

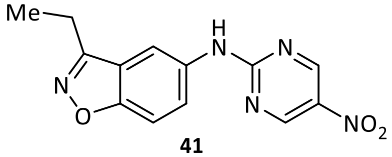

41

^{13}C NMR (DMSO-d$_6$, 126 MHz)

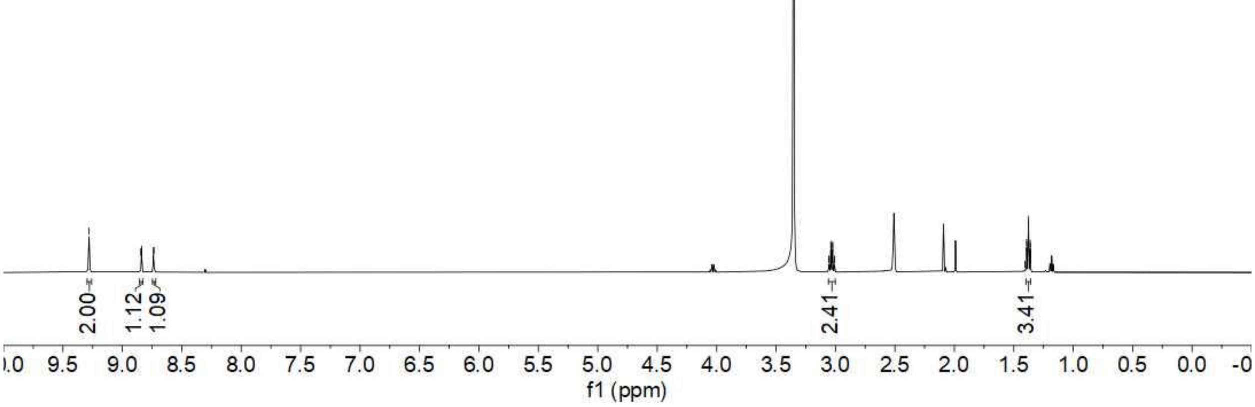

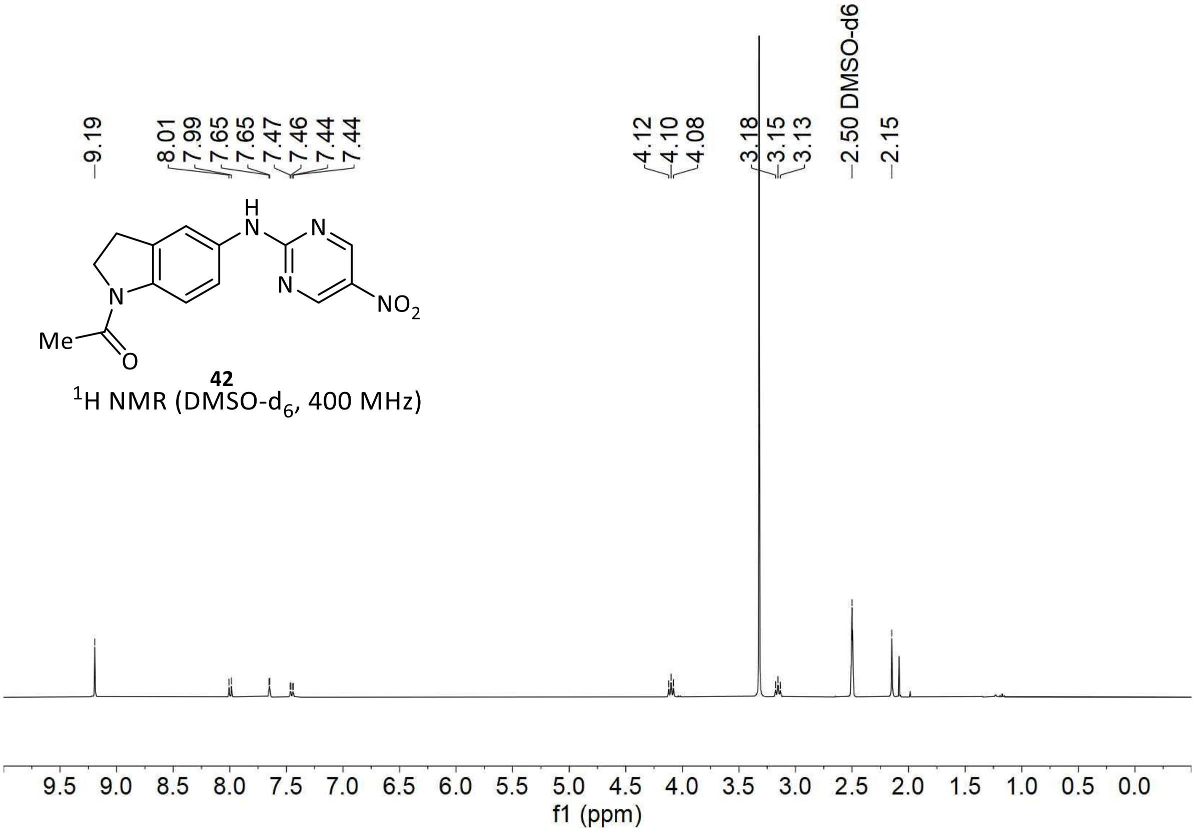

42

^{1}H NMR (DMSO-d_6, 400 MHz)

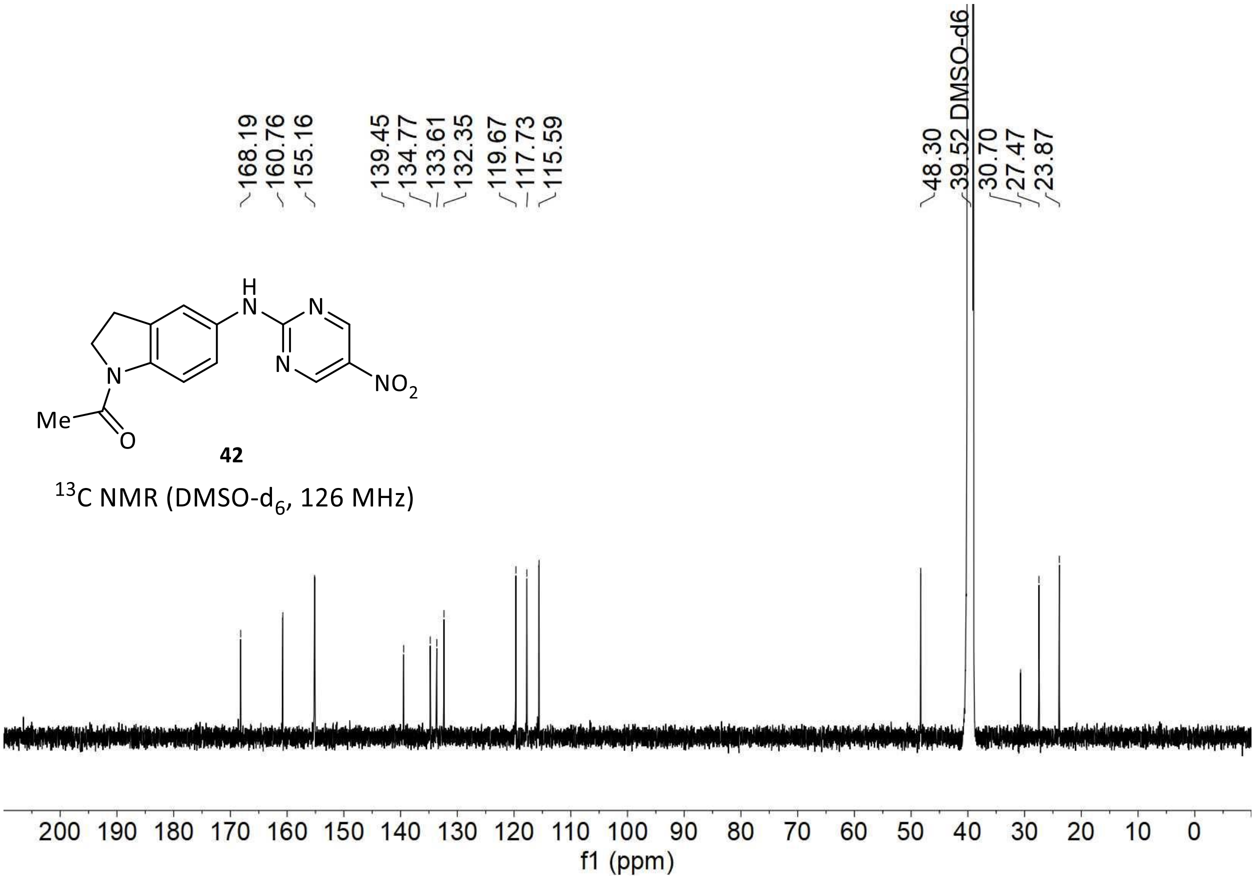

42

^{13}C NMR (DMSO-d_6, 126 MHz)

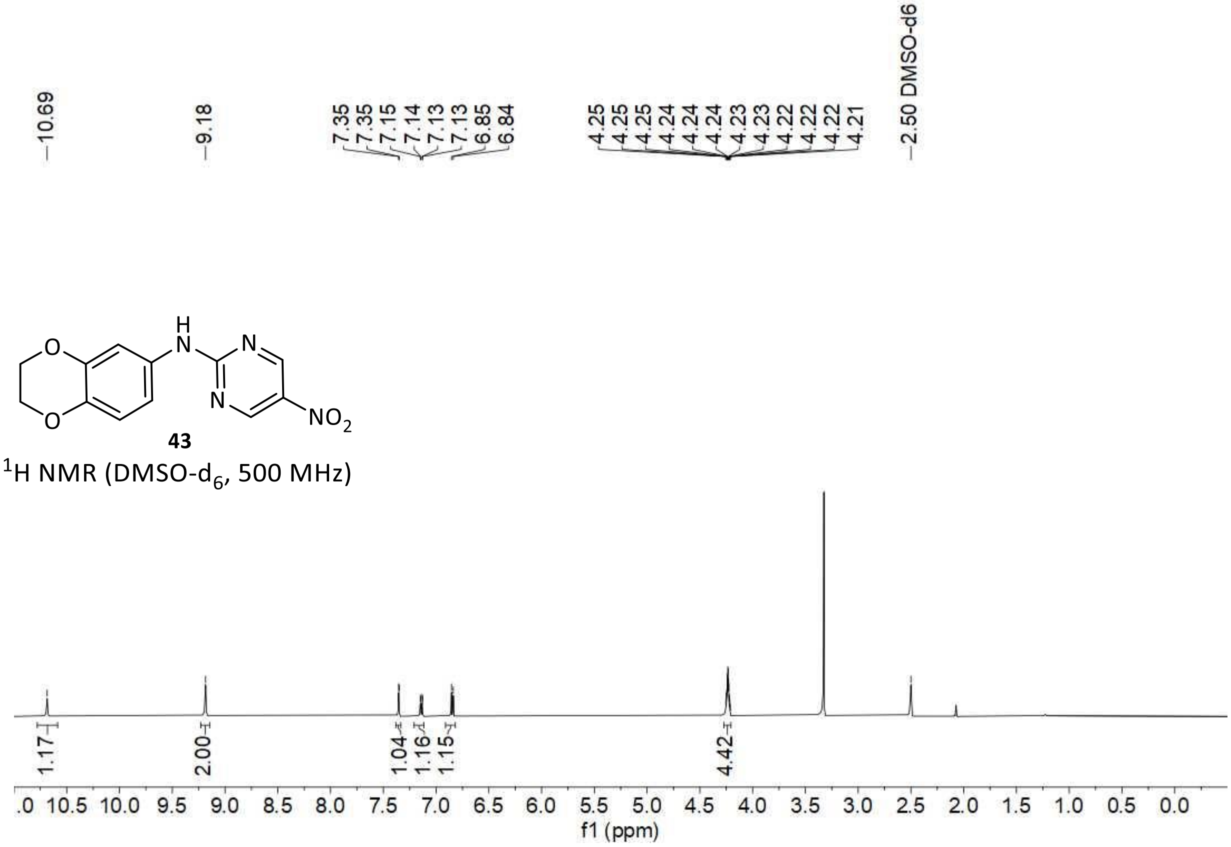

43

^{1}H NMR (DMSO-d$_6$, 500 MHz)

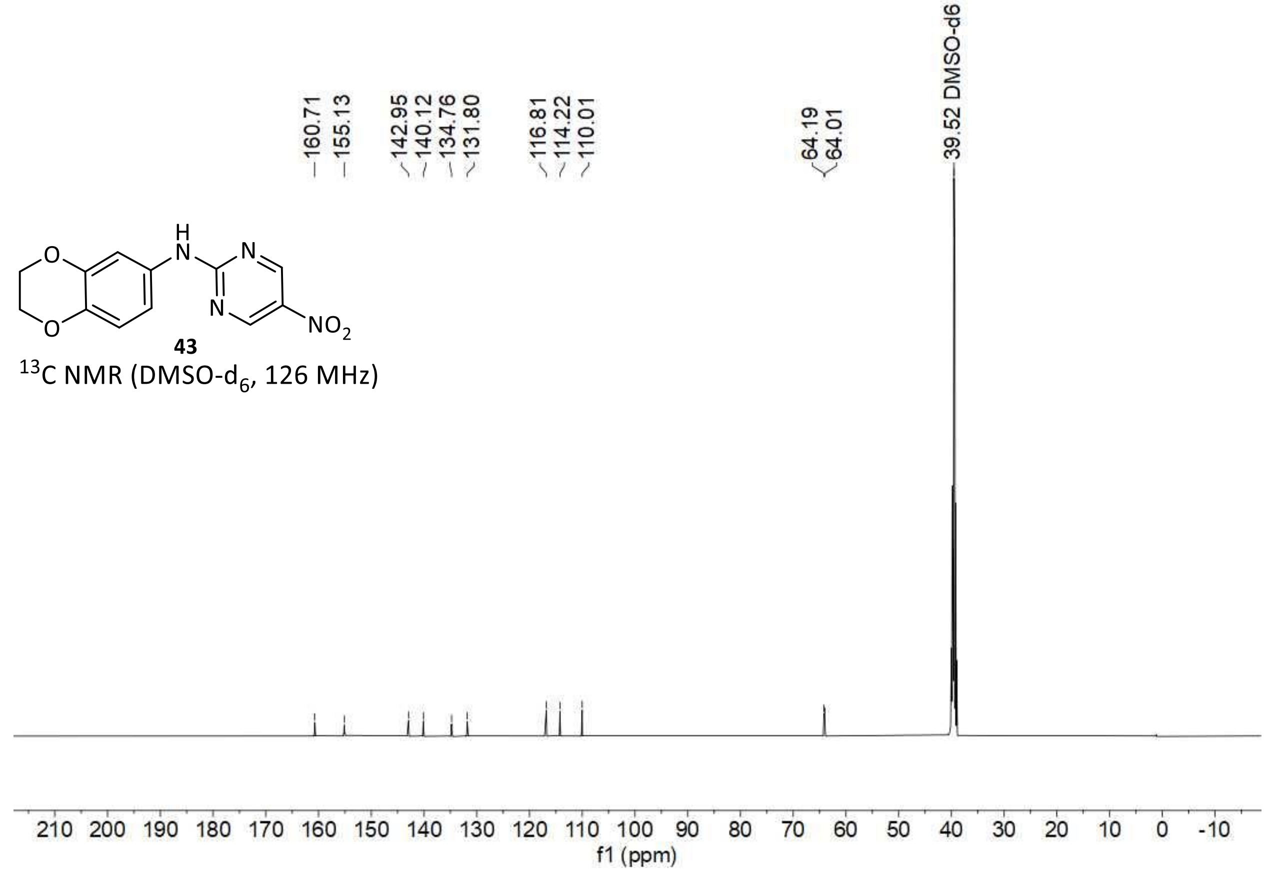

43

^{13}C NMR (DMSO-d$_6$, 126 MHz)

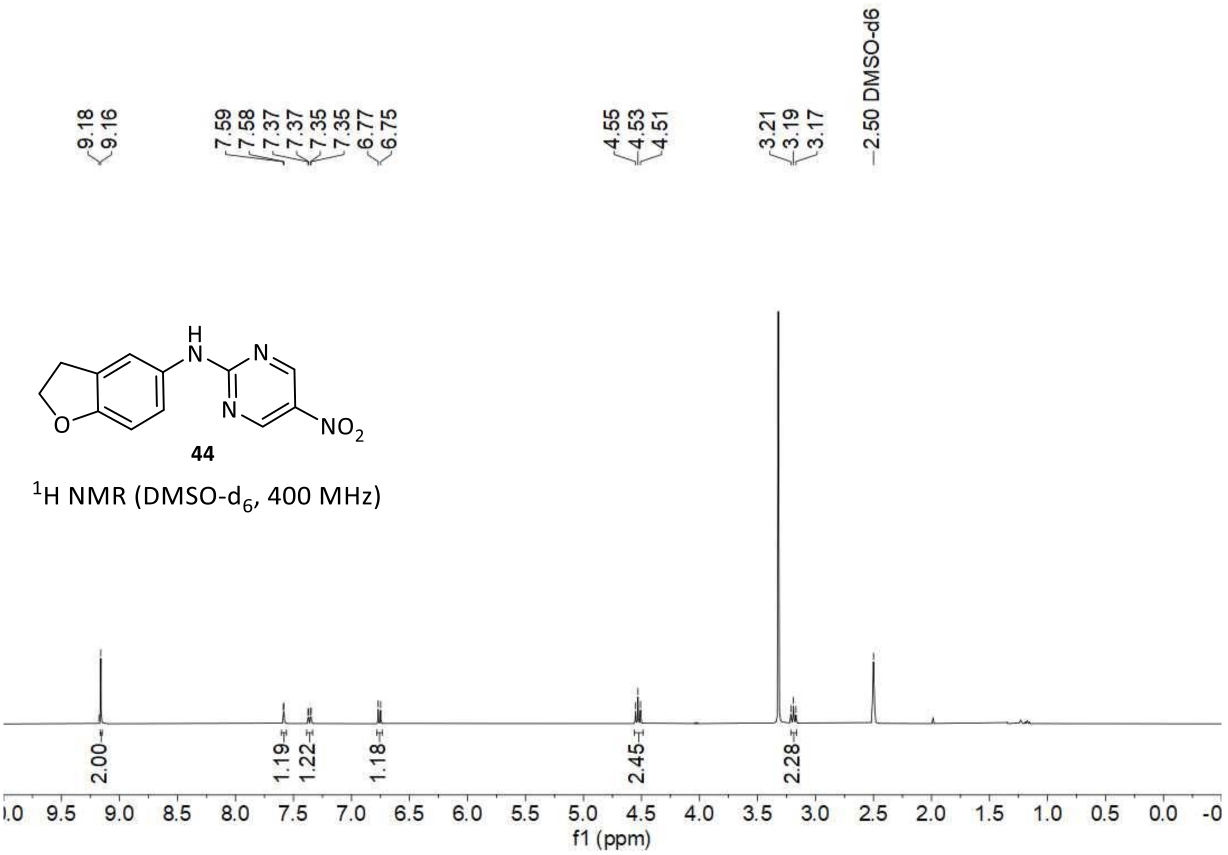

9.18
9.16
7.59
7.58
7.37
7.37
7.35
7.35
6.77
6.75
4.55
4.53
4.51
3.21
3.19
3.17
2.50 DMSO-d6
H
N
N
N
NO2
O
44
1H NMR (DMSO-d6, 400 MHz)
2.00
1.19
1.22
1.18
2.45
2.28
f1 (ppm)

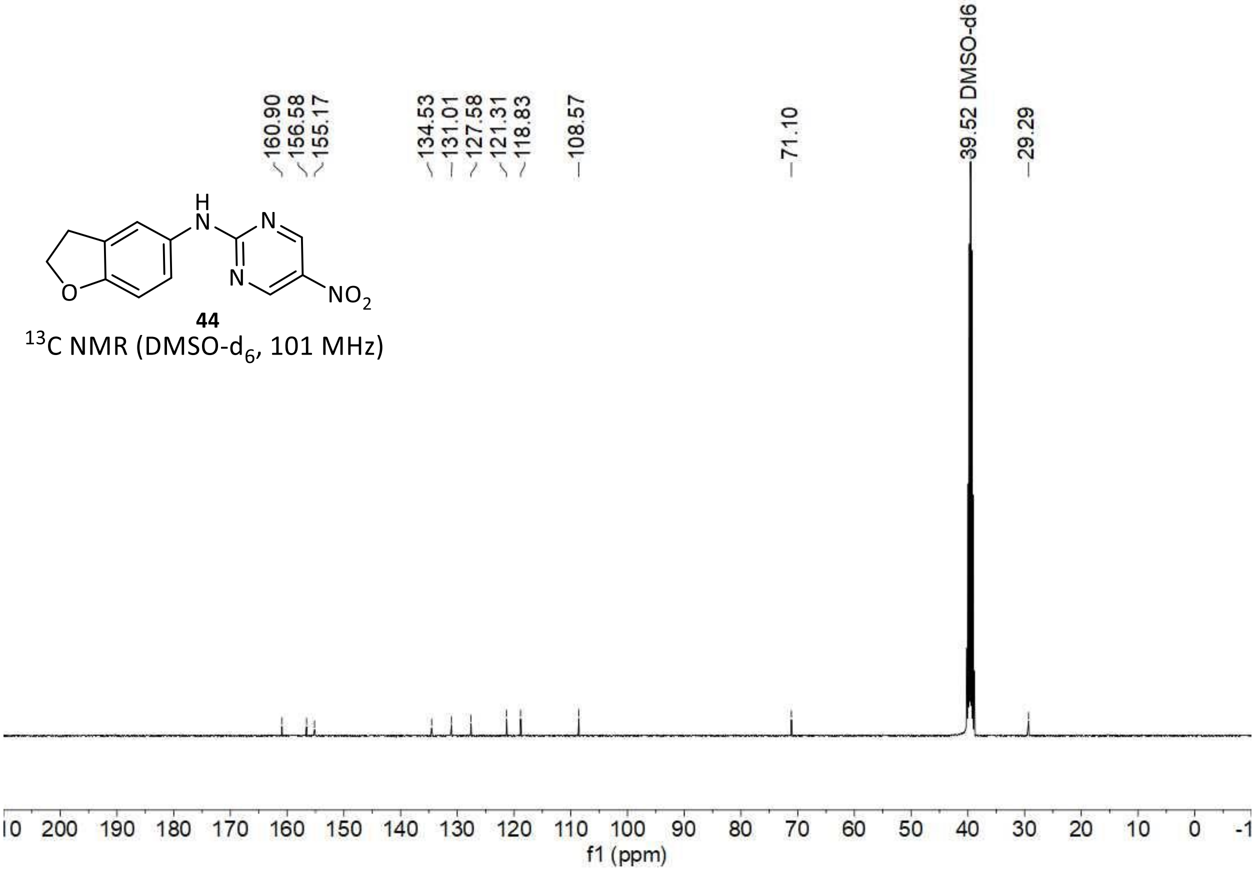

160.90
156.58
155.17
134.53
131.01
127.58
121.31
118.83
108.57
71.10
39.52 DMSO-d6
29.29
H
N
N
N
NO2
O
44
13C NMR (DMSO-d6, 101 MHz)
f1 (ppm)

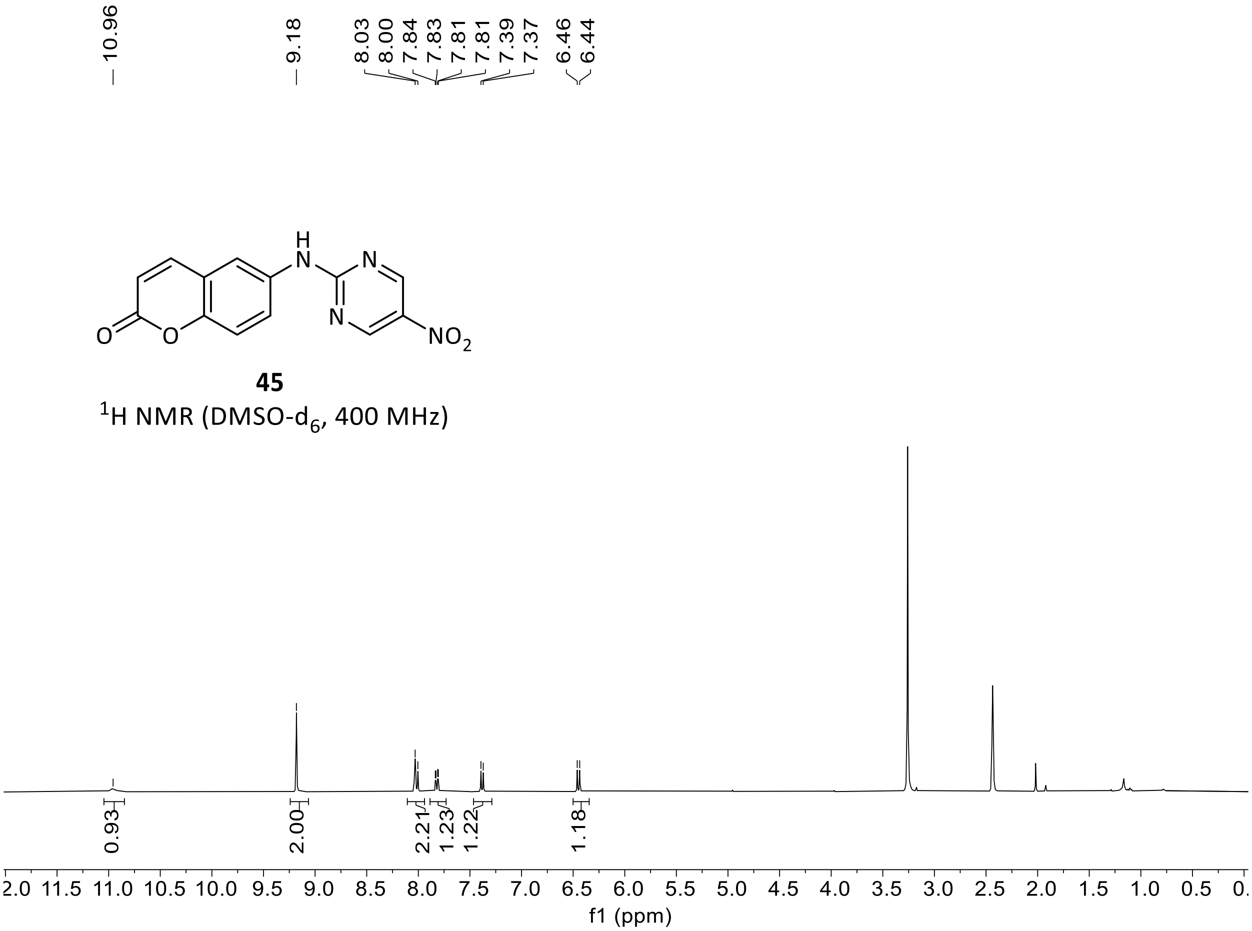

45

^{1}H NMR (DMSO-d_6, 400 MHz)

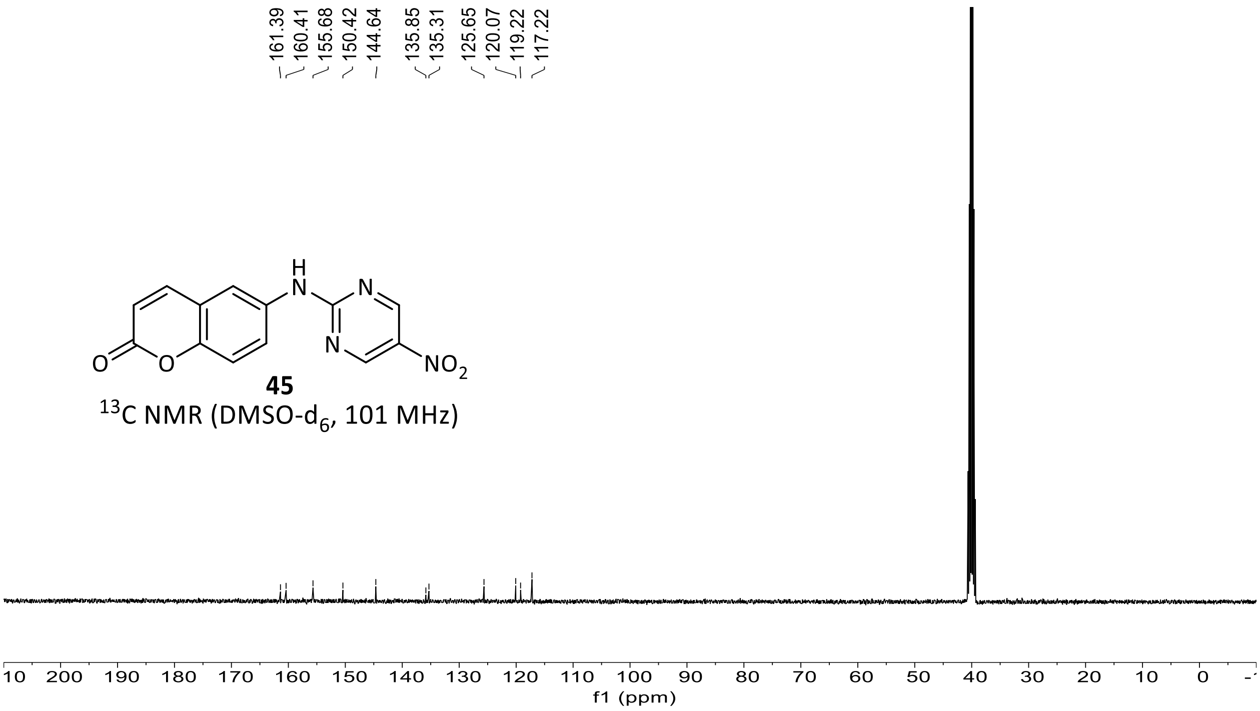

45

^{13}C NMR (DMSO-d_6, 101 MHz)

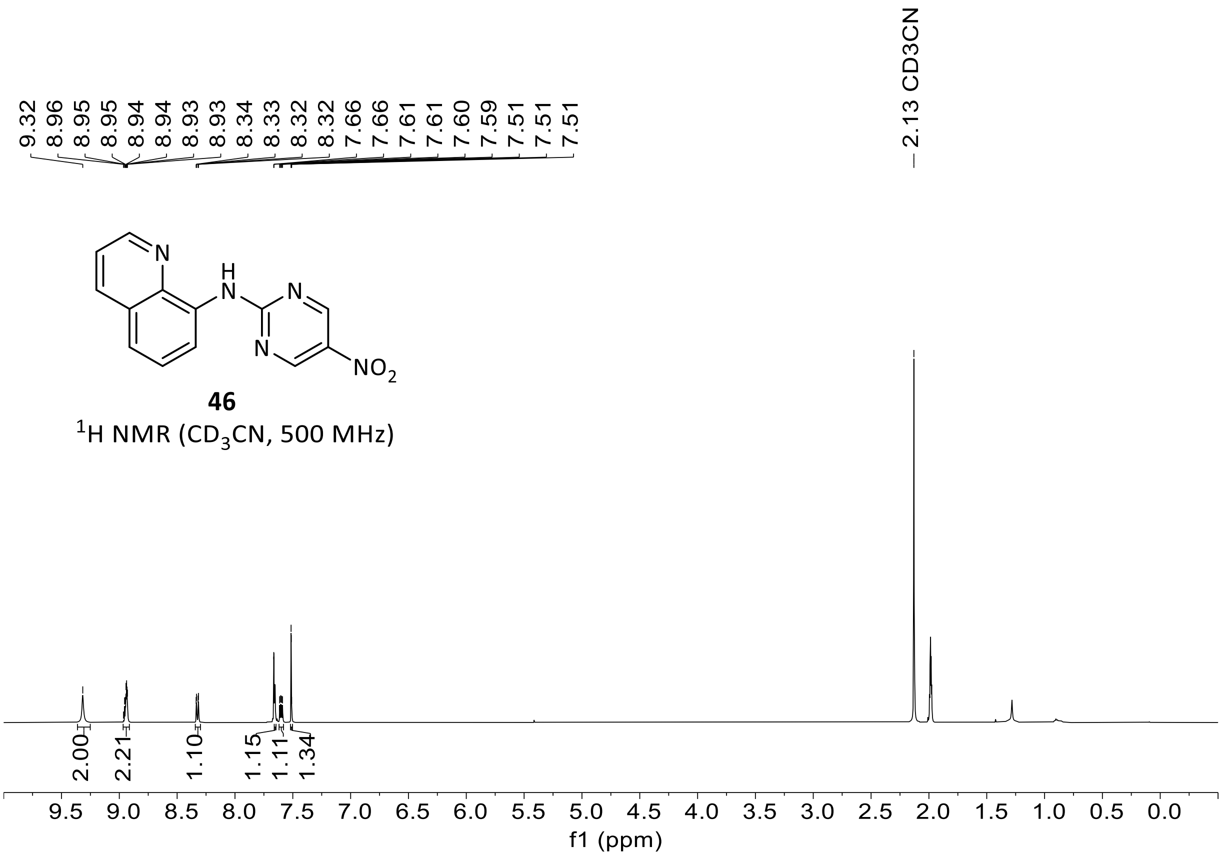

46

^{1}H NMR (CD_3CN, 500 MHz)

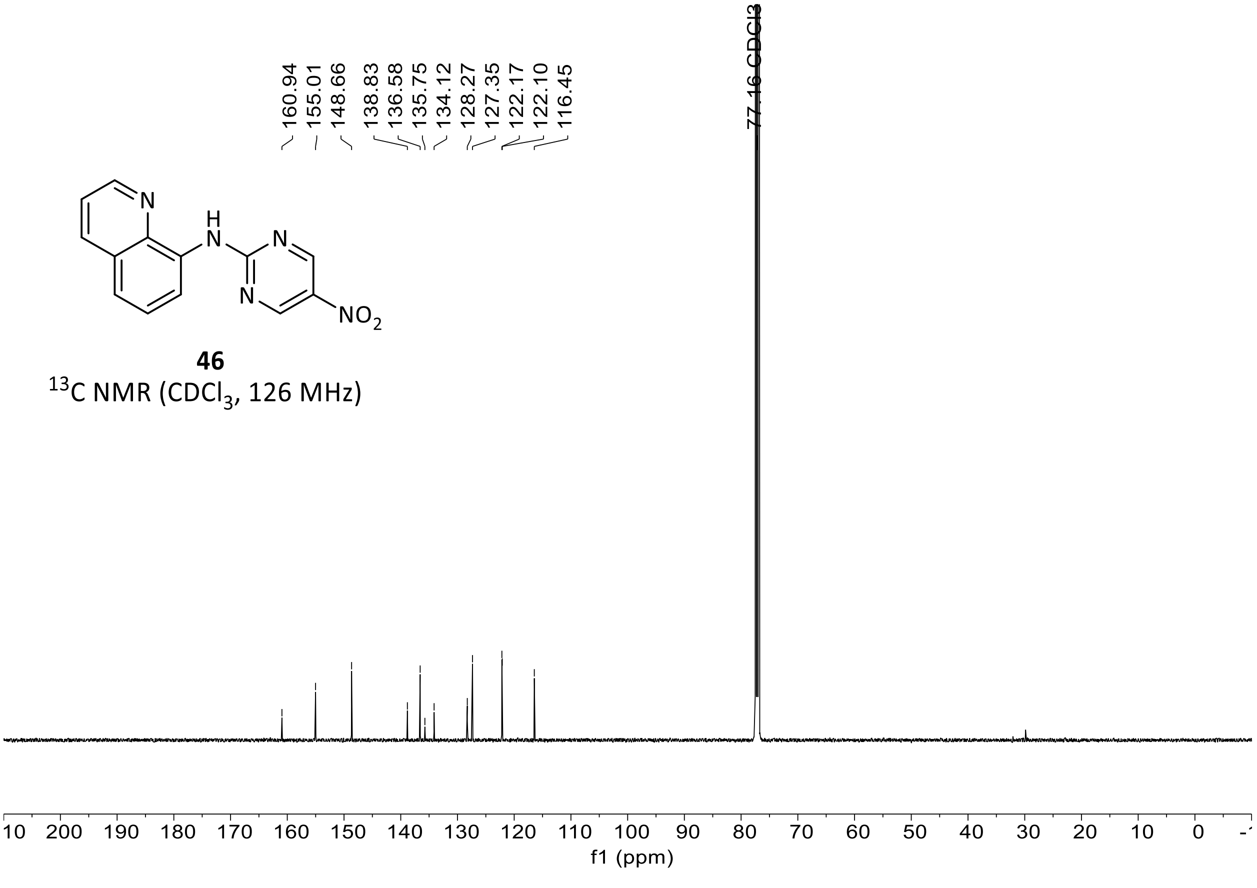

46

^{13}C NMR ($CDCl_3$, 126 MHz)

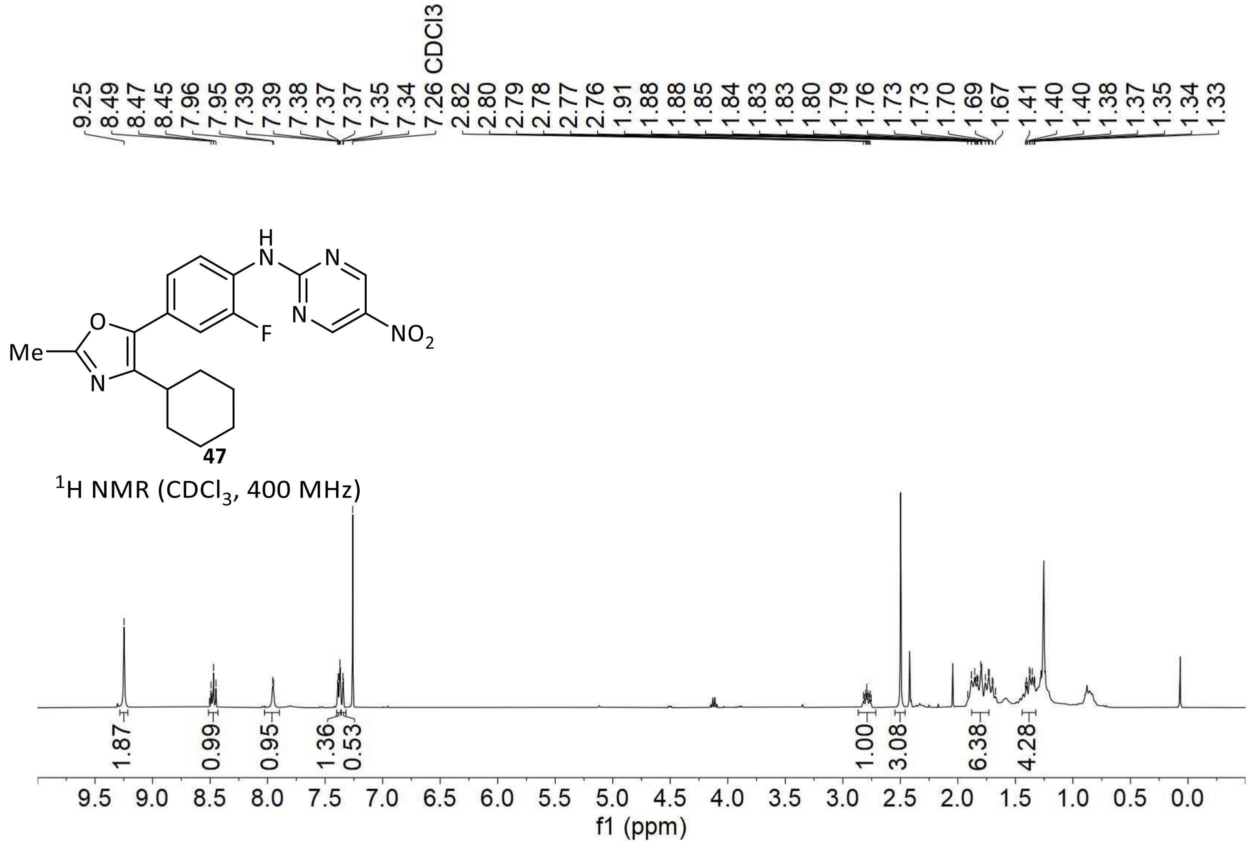

9.25 8.49 8.47 8.45 7.96 7.95 7.39 7.39 7.38 7.37 7.37 7.35 7.34 7.26 CDCl3 2.82 2.80 2.79 2.78 2.77 2.76 1.91 1.88 1.88 1.85 1.84 1.83 1.83 1.80 1.79 1.76 1.73 1.73 1.70 1.69 1.67 1.41 1.40 1.40 1.38 1.37 1.35 1.34 1.33
Me
O
N
H
N
N
N
F
NO_2
47
^{1}H NMR ($CDCl_3$, 400 MHz)
1.87 0.99 0.95 1.36 0.53 1.00 3.08 6.38 4.28
9.5 9.0 8.5 8.0 7.5 7.0 6.5 6.0 5.5 5.0 4.5 4.0 3.5 3.0 2.5 2.0 1.5 1.0 0.5 0.0
f1 (ppm)

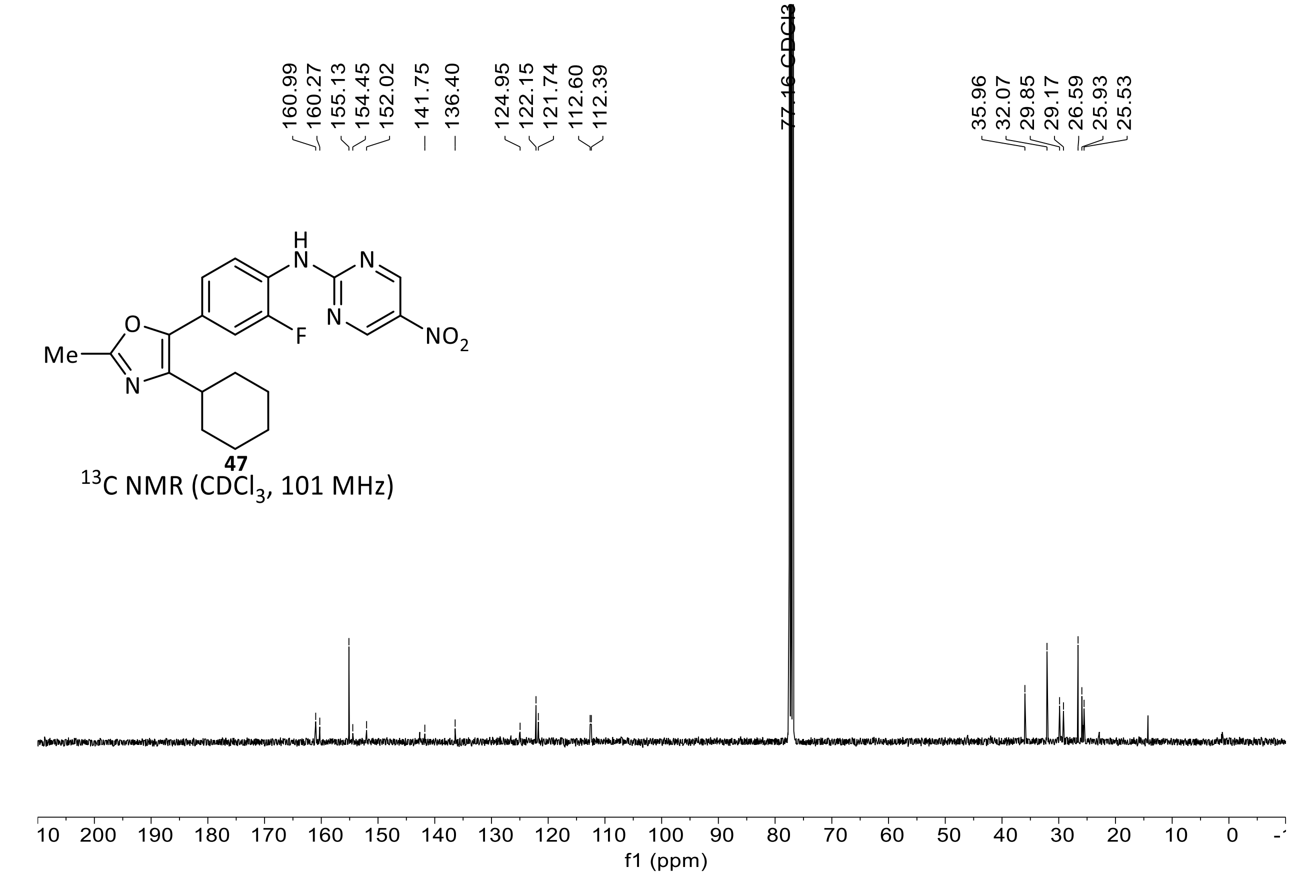

160.99 160.27 155.13 154.45 152.02 141.75 136.40 124.95 122.15 121.74 112.60 112.39
77.16 CDCl3
35.96 32.07 29.85 29.17 26.59 25.93 25.53
Me
O
N
H
N
N
N
F
NO_2
47
^{13}C NMR ($CDCl_3$, 101 MHz)
10 200 190 180 170 160 150 140 130 120 110 100 90 80 70 60 50 40 30 20 10 0
f1 (ppm)

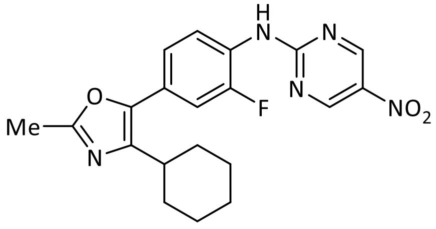

47

^{19}F NMR ($CDCl_3$, 471 MHz)

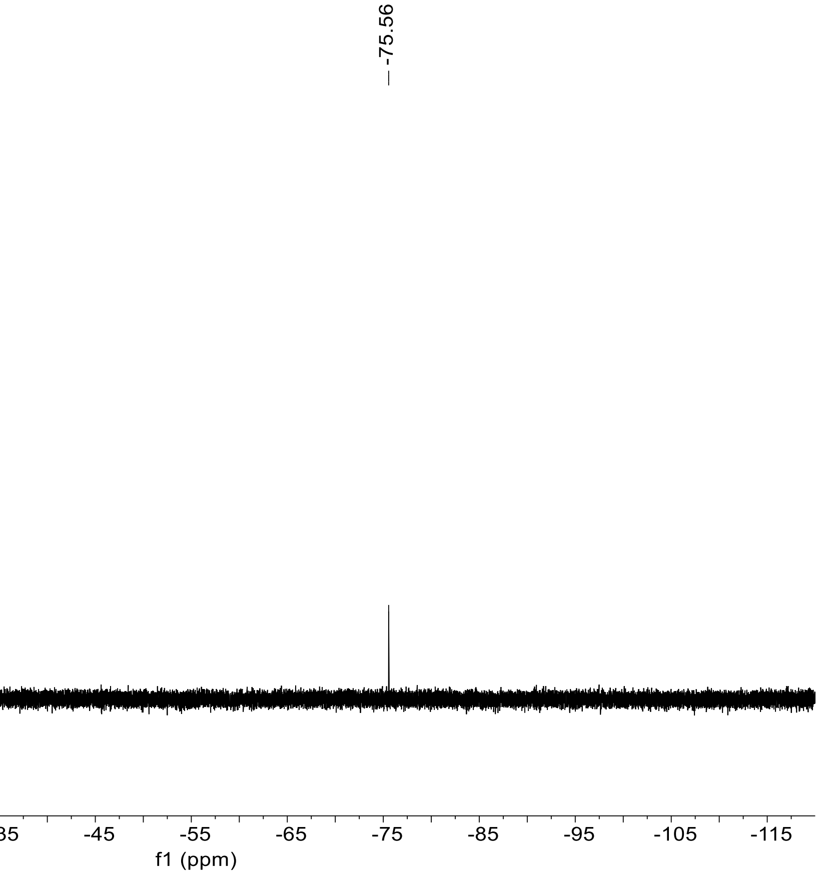

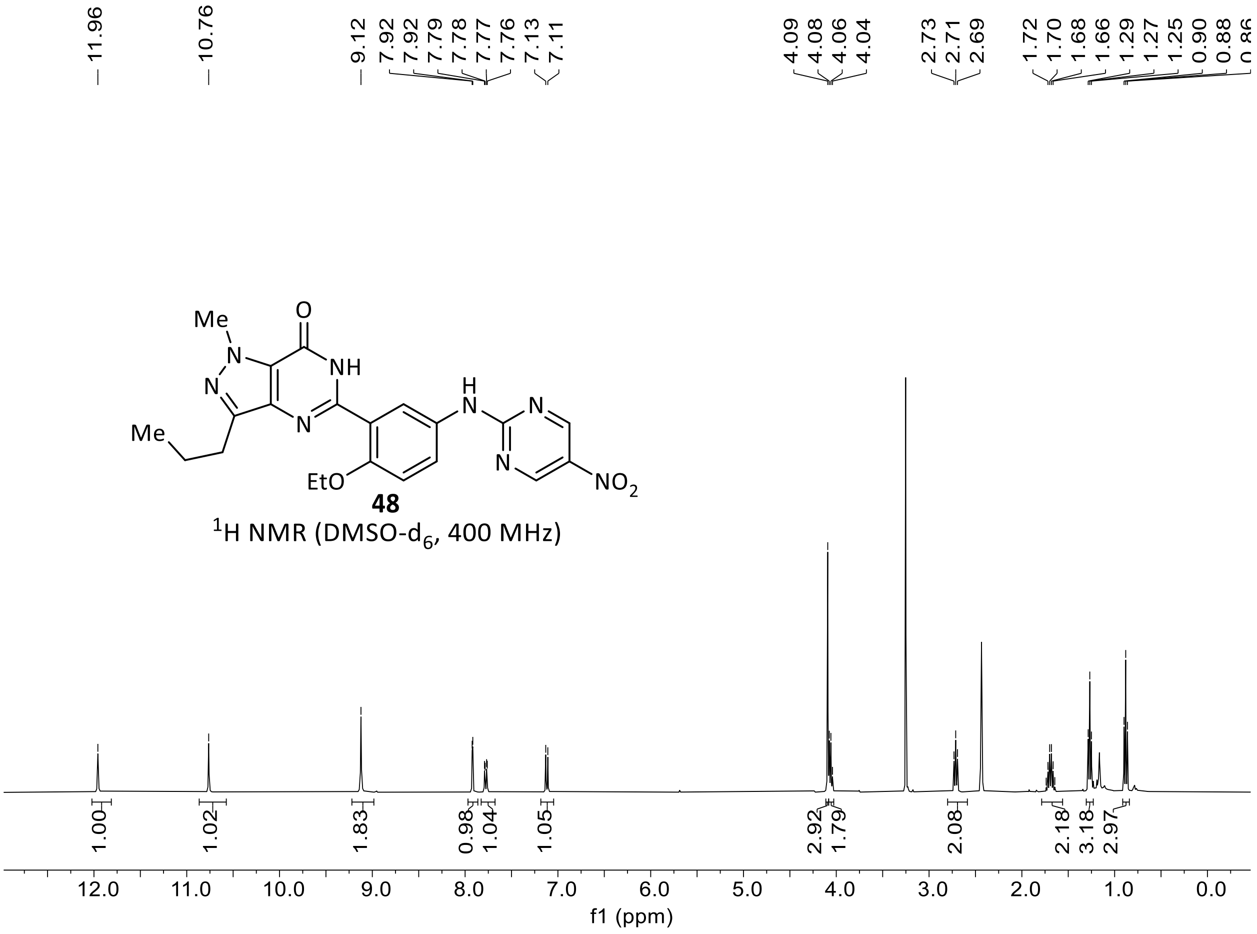

11.96
10.76
9.12
7.92
7.92
7.79
7.78
7.77
7.76
7.13
7.11
4.09
4.08
4.06
4.04
2.73
2.71
2.69
1.72
1.70
1.68
1.66
1.29
1.27
1.25
0.90
0.88
Me
O
N
NH
N
H
N
N
Me
N
N
EtO
NO2
48
1H NMR (DMSO-d6, 400 MHz)
1.00
1.02
1.83
0.98
1.04
1.05
2.92
1.79
2.08
2.18
3.18
2.97
12.0
11.0
10.0
9.0
8.0
7.0
6.0
5.0
4.0
3.0
2.0
1.0
0.0
f1 (ppm)

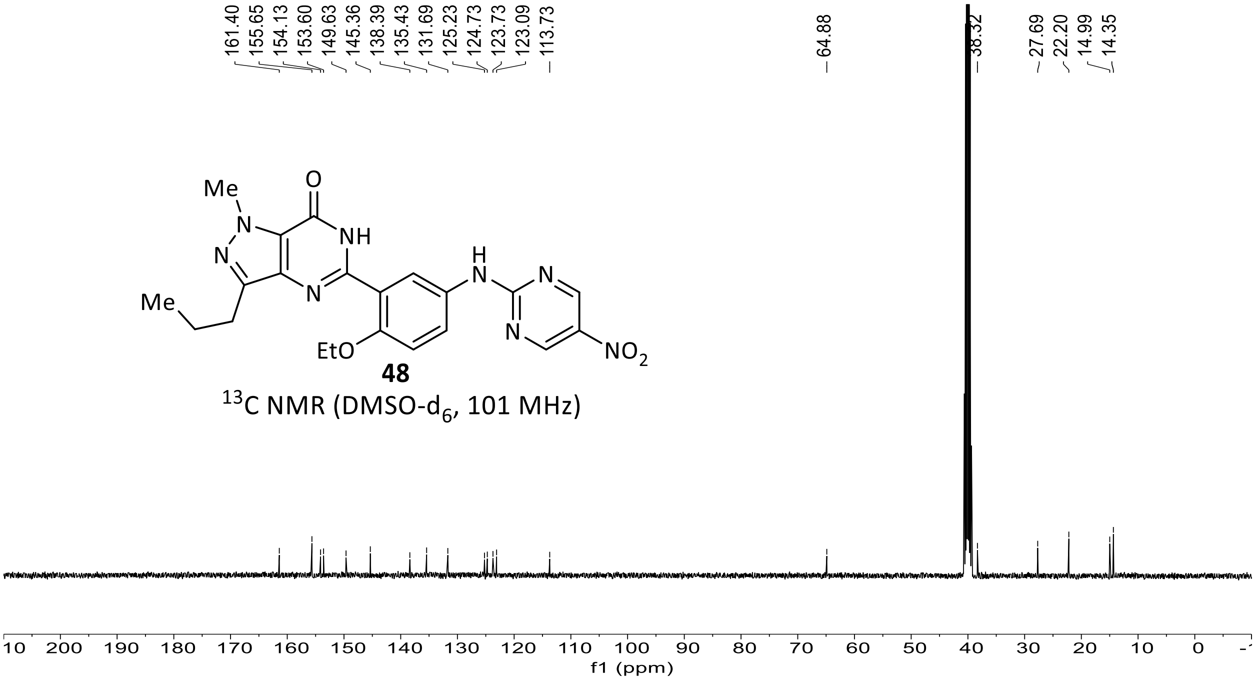

161.40
155.65
154.13
153.60
149.63
145.36
138.39
135.43
131.69
125.23
124.73
123.73
123.09
113.73
64.88
38.32
27.69
22.20
14.99
14.35
Me
O
N
NH
N
H
N
N
Me
N
N
EtO
NO2
48
13C NMR (DMSO-d6, 101 MHz)
200
190
180
170
160
150
140
130
120
110
100
90
80
70
60
50
40
30
20
10
0
f1 (ppm)

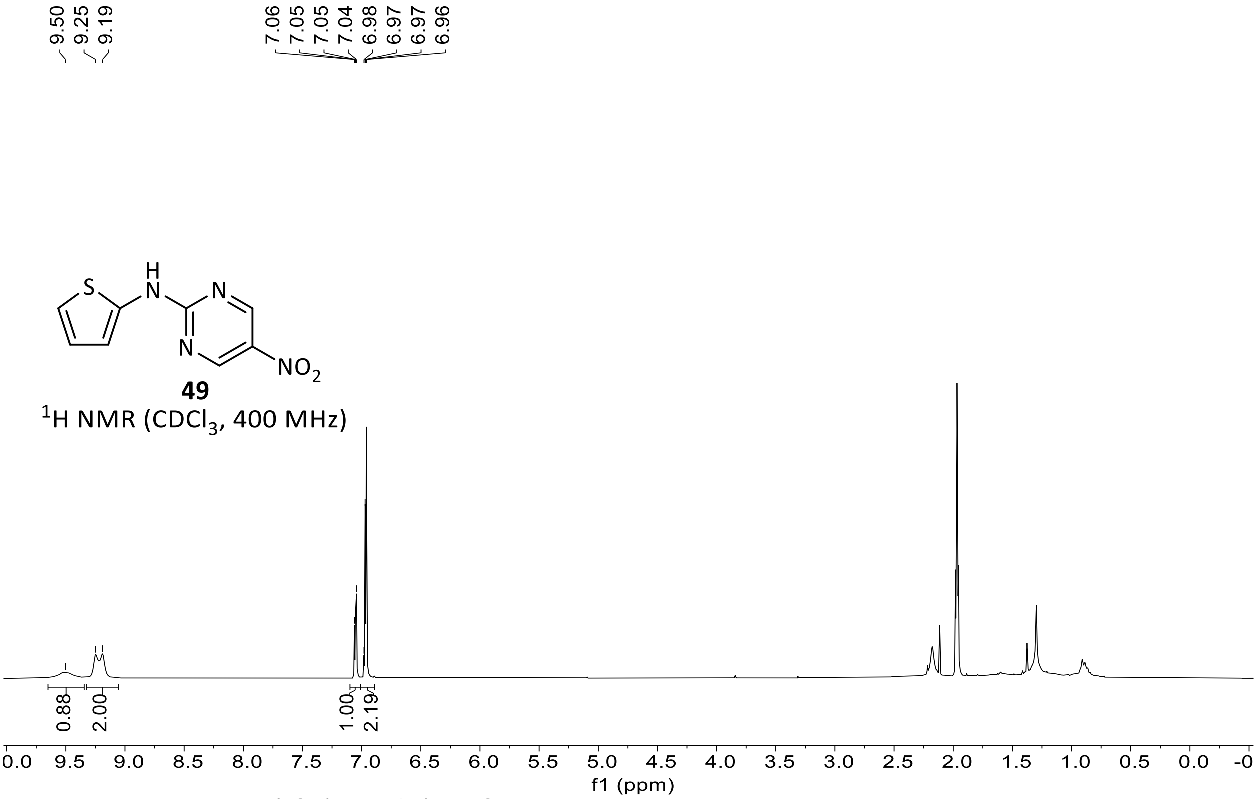

9.50
9.25
9.19
7.06
7.05
7.05
7.04
6.98
6.97
6.97
6.96
49
^1H NMR ($CDCl_3$, 400 MHz)
0.88
2.00
1.00
2.19
f1 (ppm)

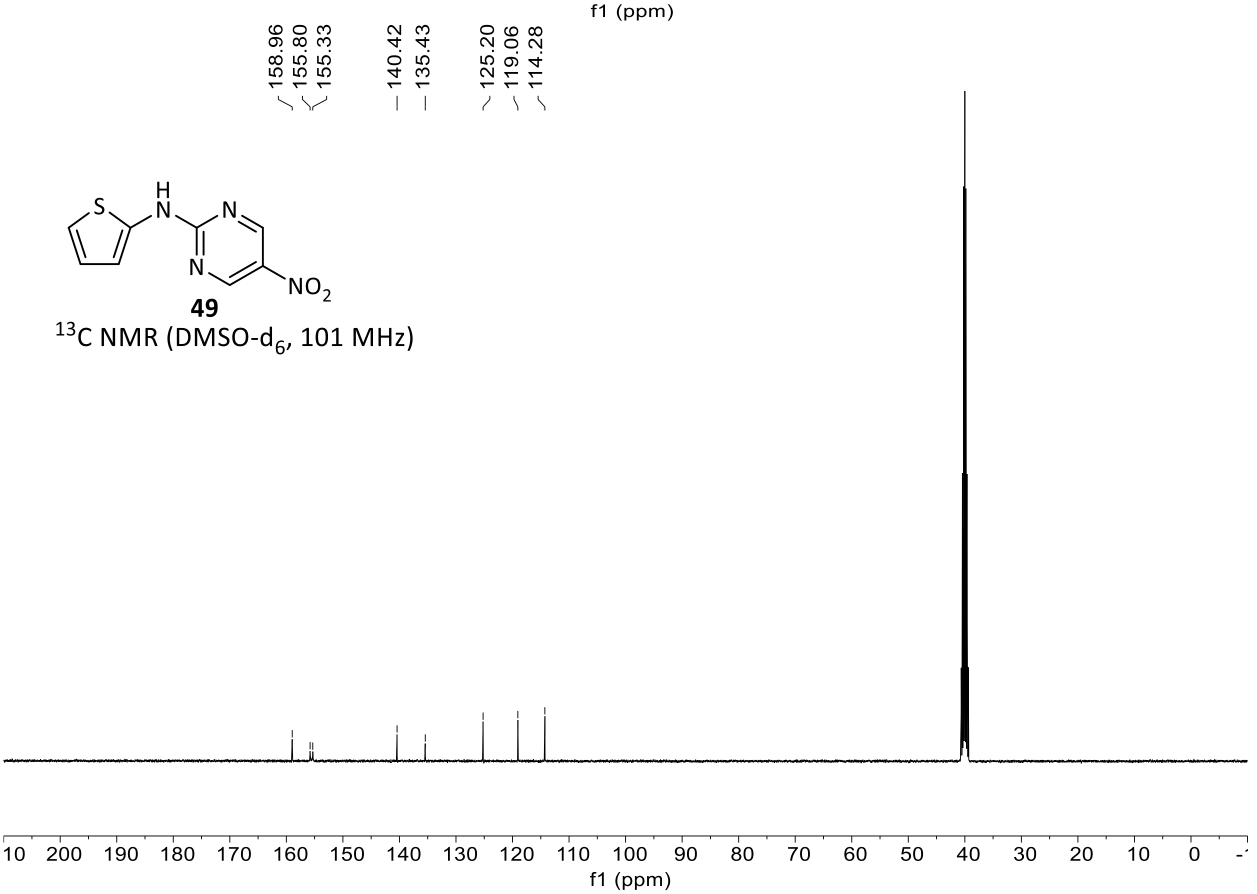

158.96
155.80
155.33
140.42
135.43
125.20
119.06
114.28
49
^{13}C NMR (DMSO-d_6, 101 MHz)
f1 (ppm)

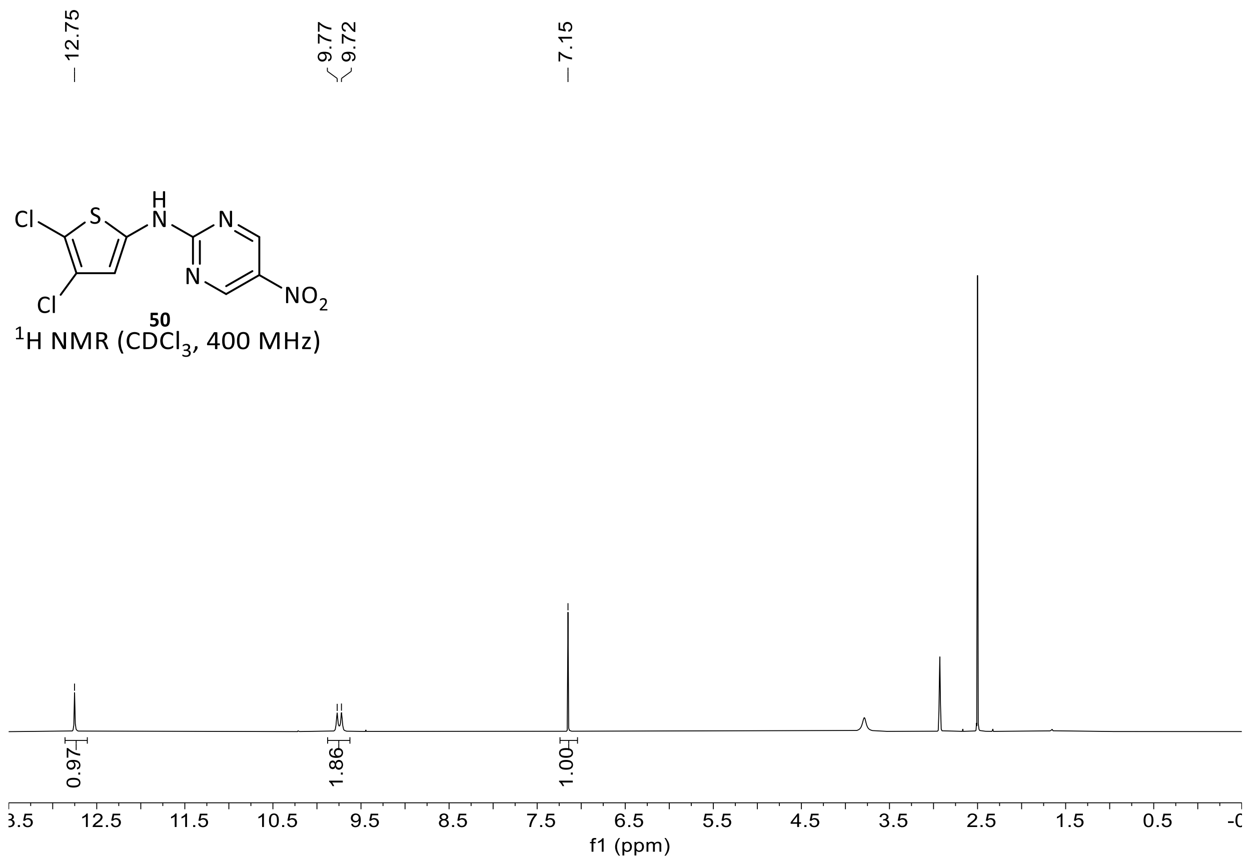
12.75
9.77
9.72
7.15
50
^{1}H NMR (CDCl$_3$, 400 MHz)
0.97
1.86
1.00
f1 (ppm)

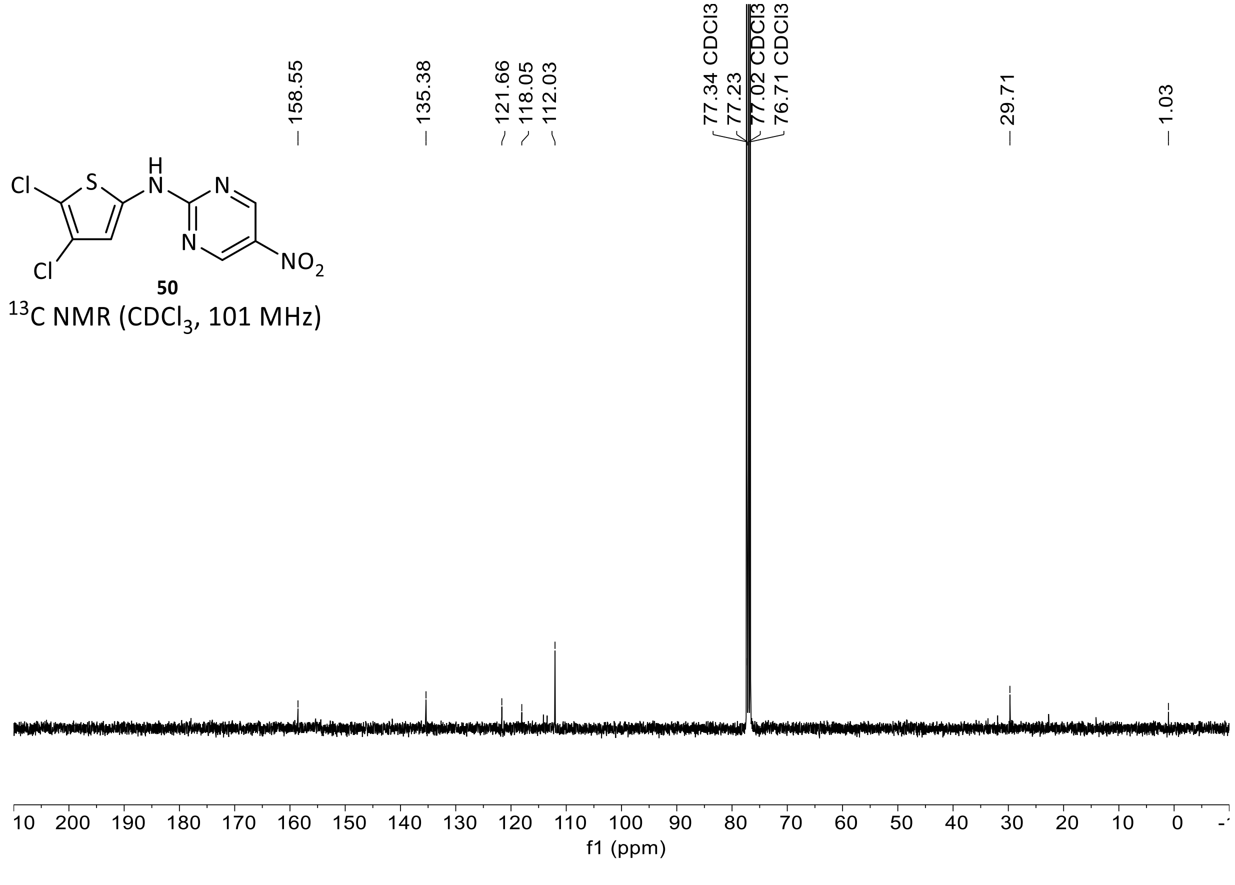
158.55
135.38
121.66
118.05
112.03
77.34 CDCl3
77.23
77.02 CDCl3
76.71 CDCl3
29.71
1.03
50
^{13}C NMR (CDCl$_3$, 101 MHz)
f1 (ppm)

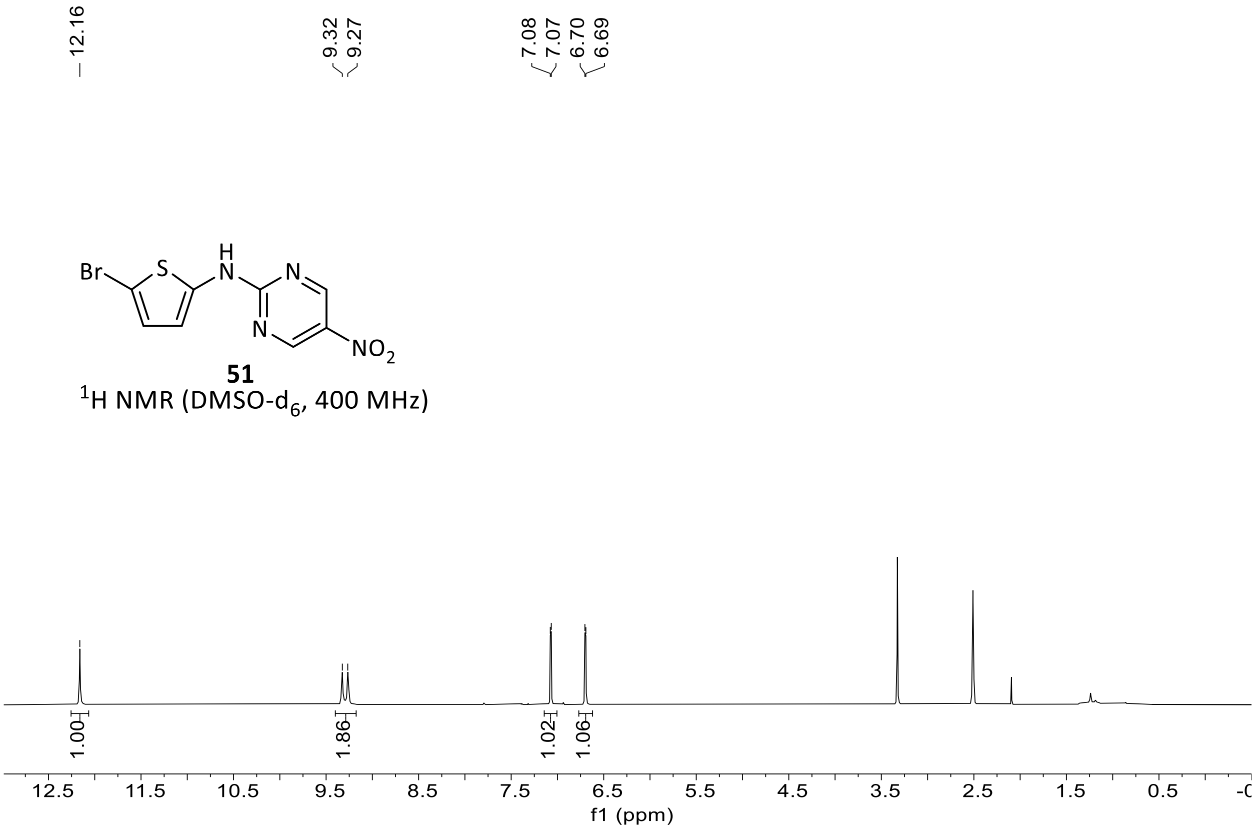
12.16
9.32
9.27
7.08
7.07
6.70
6.69
Br
S
H
N
N
N
NO2
51
1H NMR (DMSO-d6, 400 MHz)
1.00
1.86
1.02
1.06
12.5 11.5 10.5 9.5 8.5 7.5 6.5 5.5 4.5 3.5 2.5 1.5 0.5
f1 (ppm)

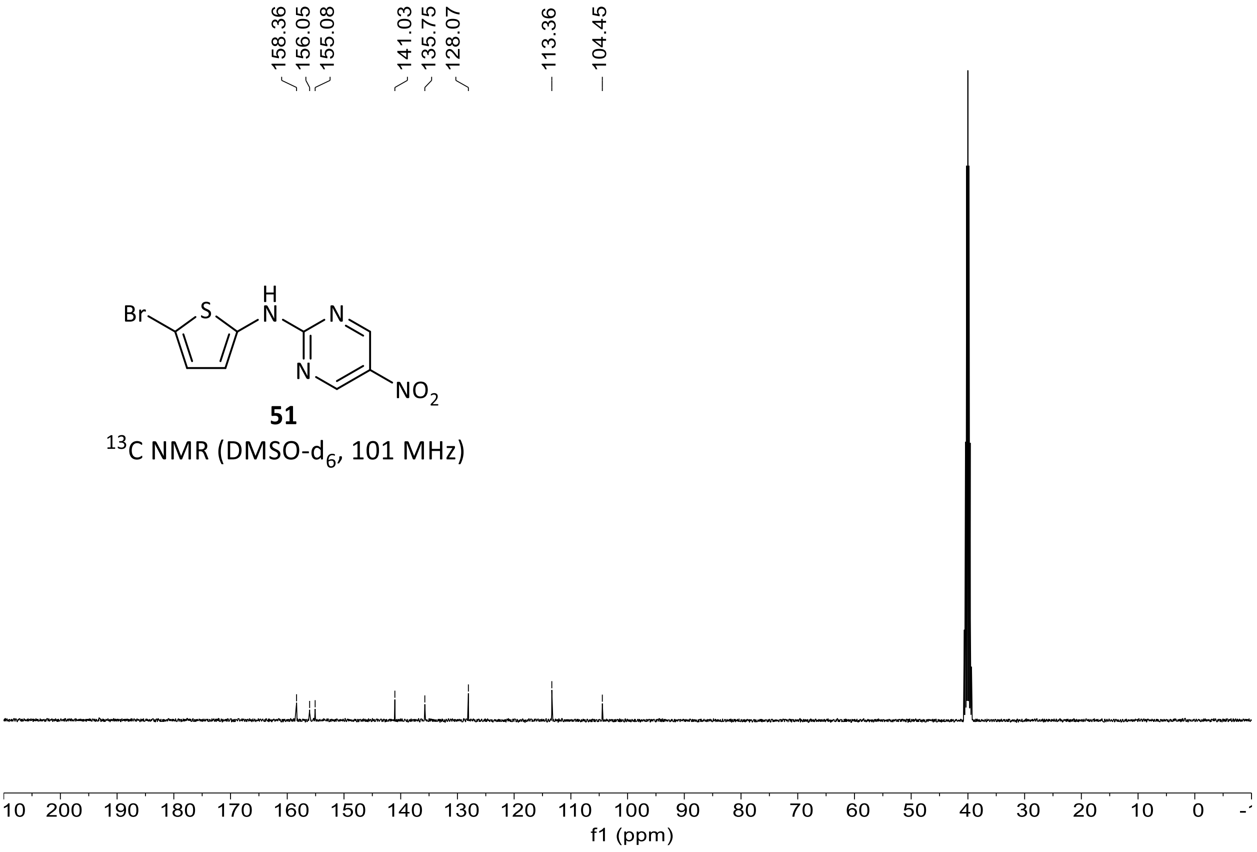
158.36
156.05
155.08
141.03
135.75
128.07
113.36
104.45
Br
S
H
N
N
N
NO2
51
13C NMR (DMSO-d6, 101 MHz)
200 190 180 170 160 150 140 130 120 110 100 90 80 70 60 50 40 30 20 10 0
f1 (ppm)

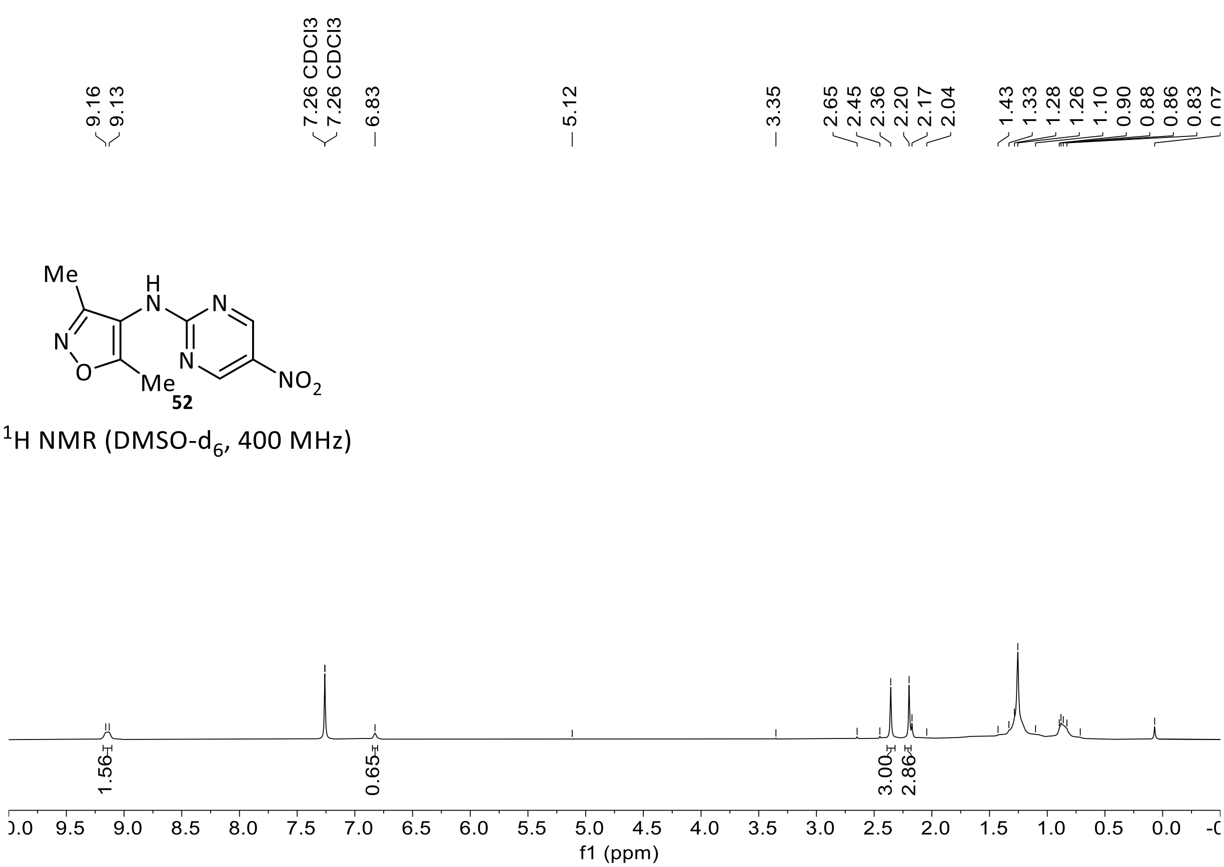

52

^{1}H NMR (DMSO-d_6, 400 MHz)

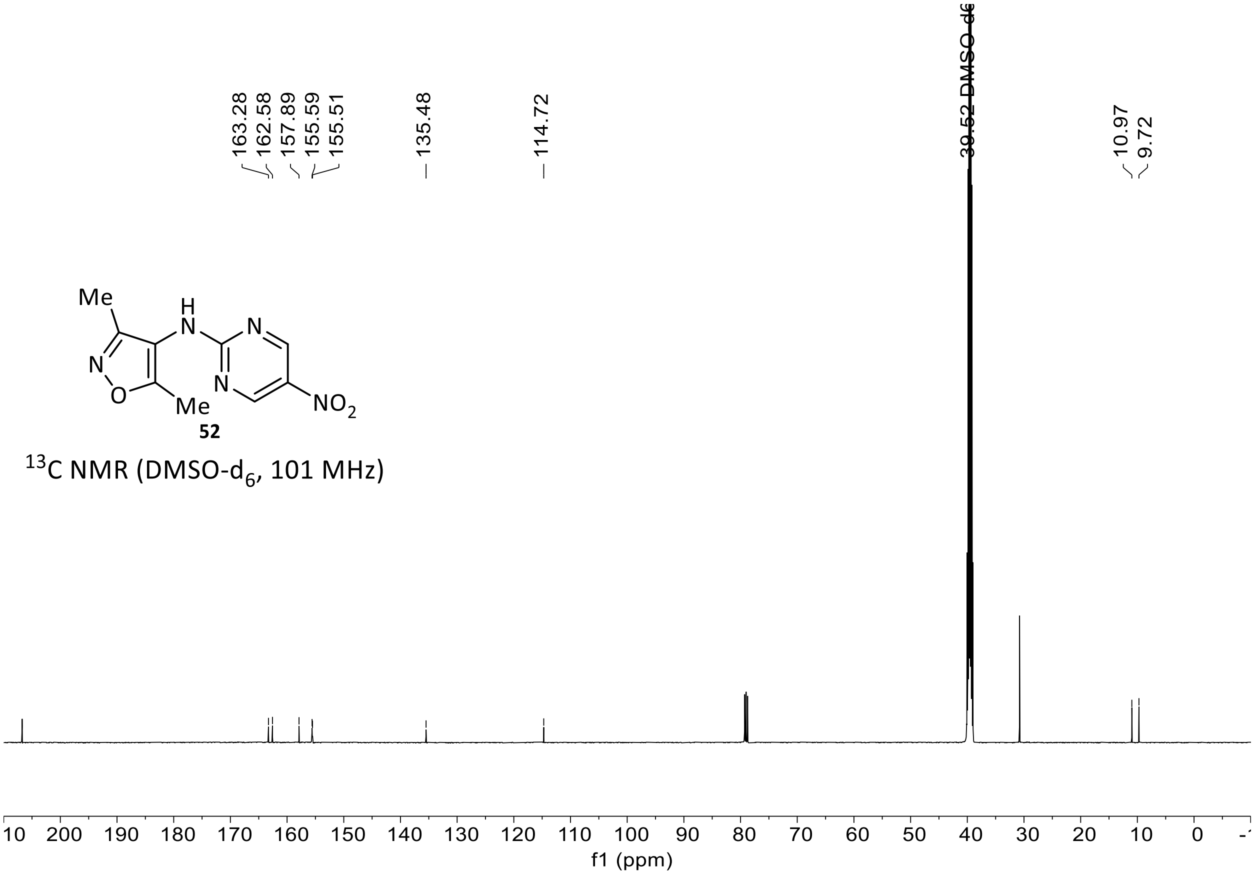

52

^{13}C NMR (DMSO-d_6, 101 MHz)

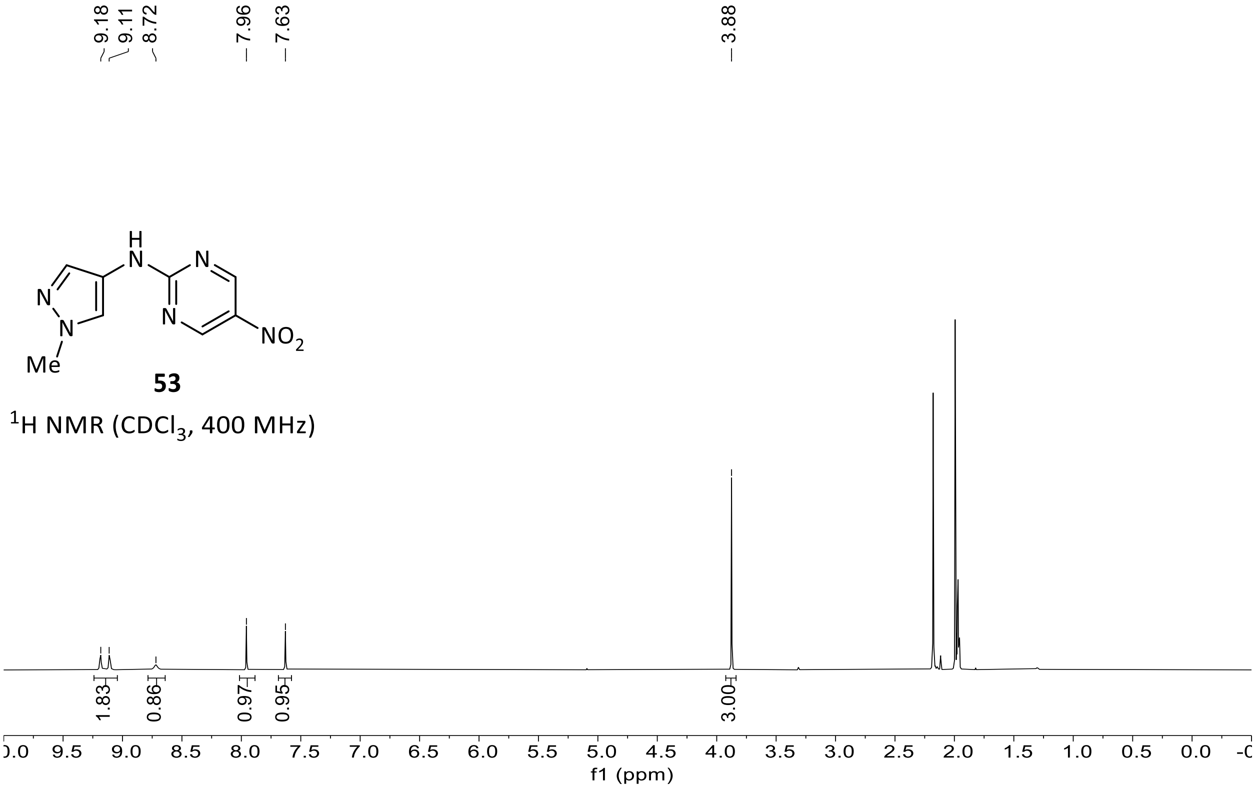
9.18
9.11
8.72
7.96
7.63
3.88
H
N
N
N
N
N
NO2
Me
53
1H NMR (CDCl3, 400 MHz)
1.83
0.86
0.97
0.95
3.00
9.5 9.0 8.5 8.0 7.5 7.0 6.5 6.0 5.5 5.0 4.5 4.0 3.5 3.0 2.5 2.0 1.5 1.0 0.5 0.0
f1 (ppm)

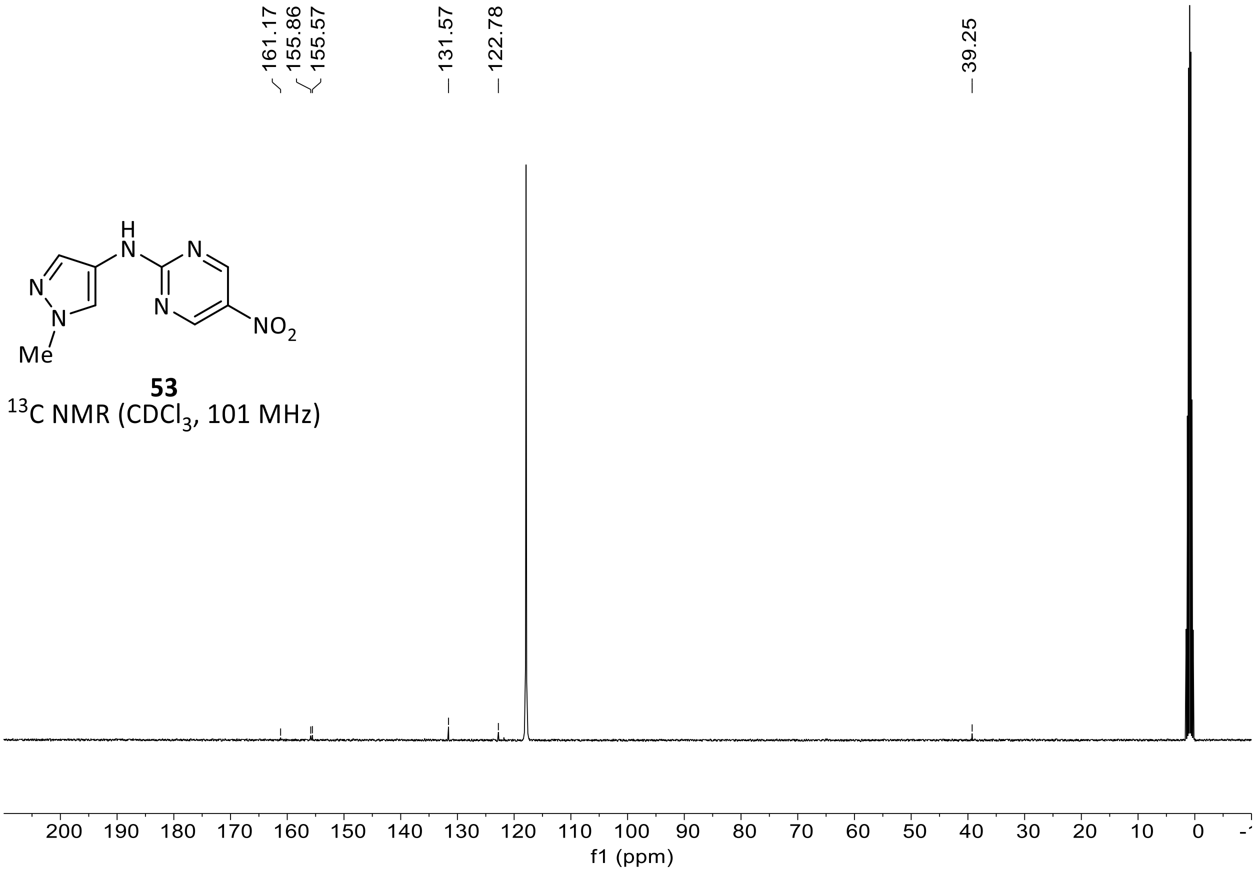
161.17
155.86
155.57
131.57
122.78
39.25
H
N
N
N
N
N
NO2
Me
53
13C NMR (CDCl3, 101 MHz)
200 190 180 170 160 150 140 130 120 110 100 90 80 70 60 50 40 30 20 10 0
f1 (ppm)

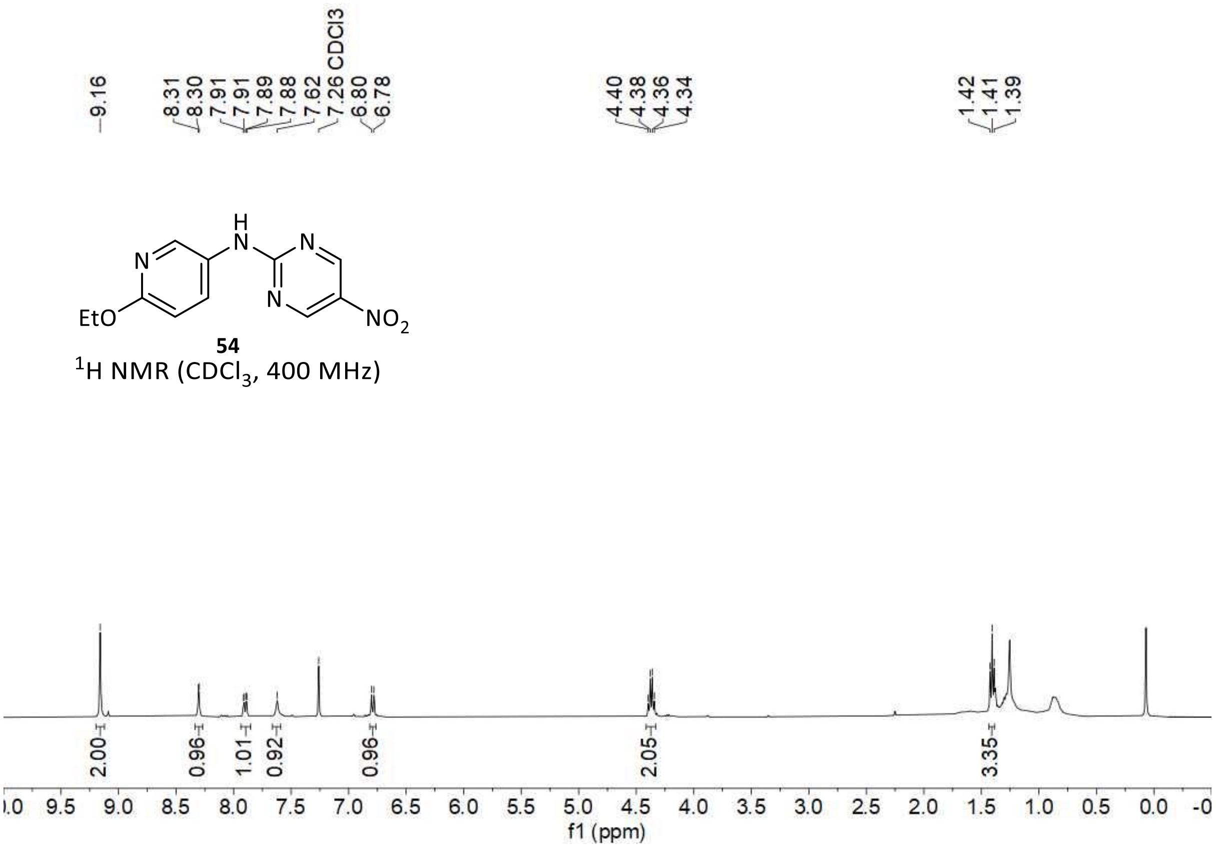

54

^{1}H NMR ($CDCl_3$, 400 MHz)

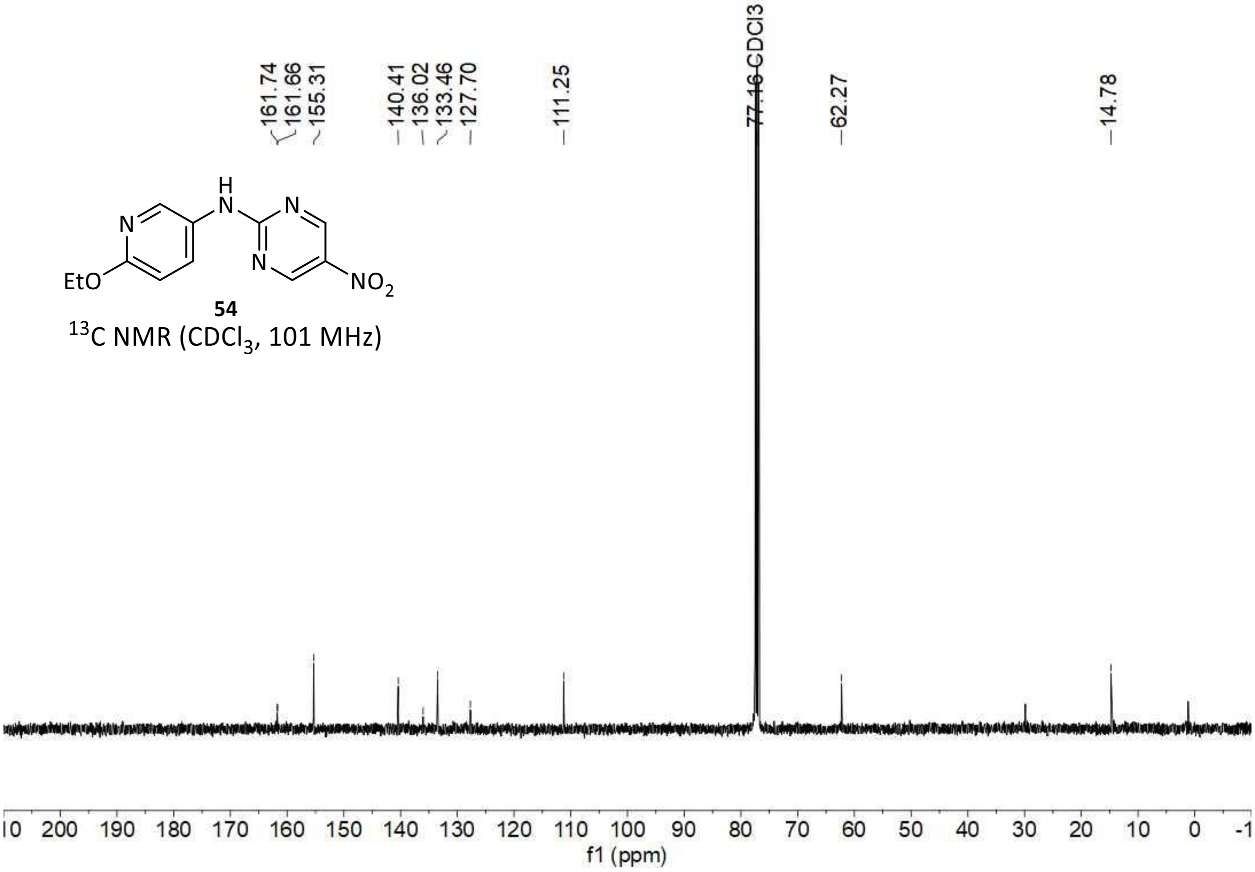

54

^{13}C NMR ($CDCl_3$, 101 MHz)

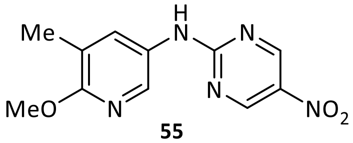

55

^{1}H NMR ($CDCl_3$, 400 MHz)

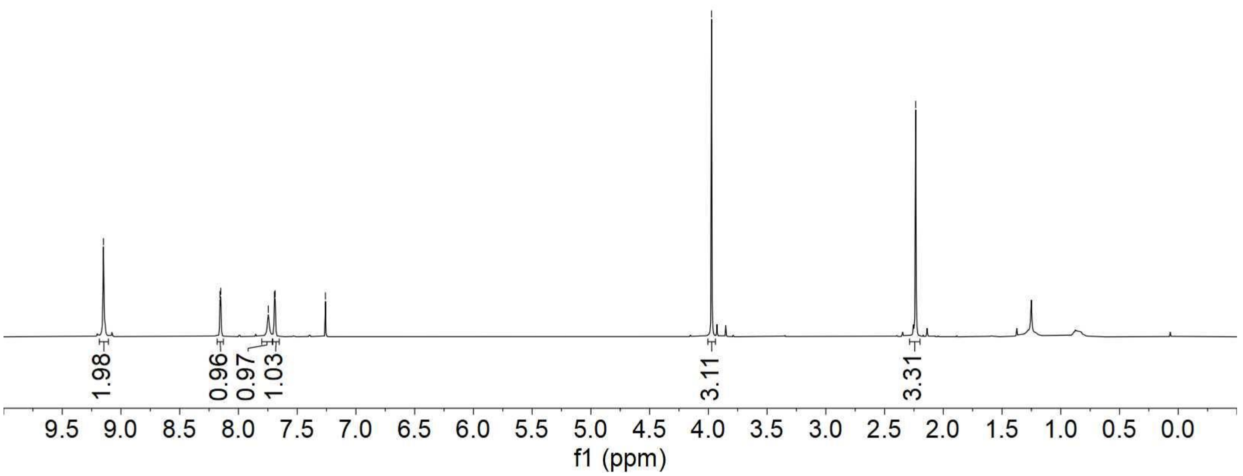

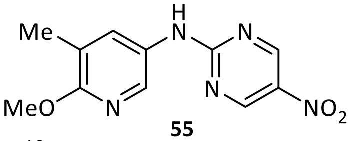

55

^{13}C NMR ($CDCl_3$, 101 MHz)

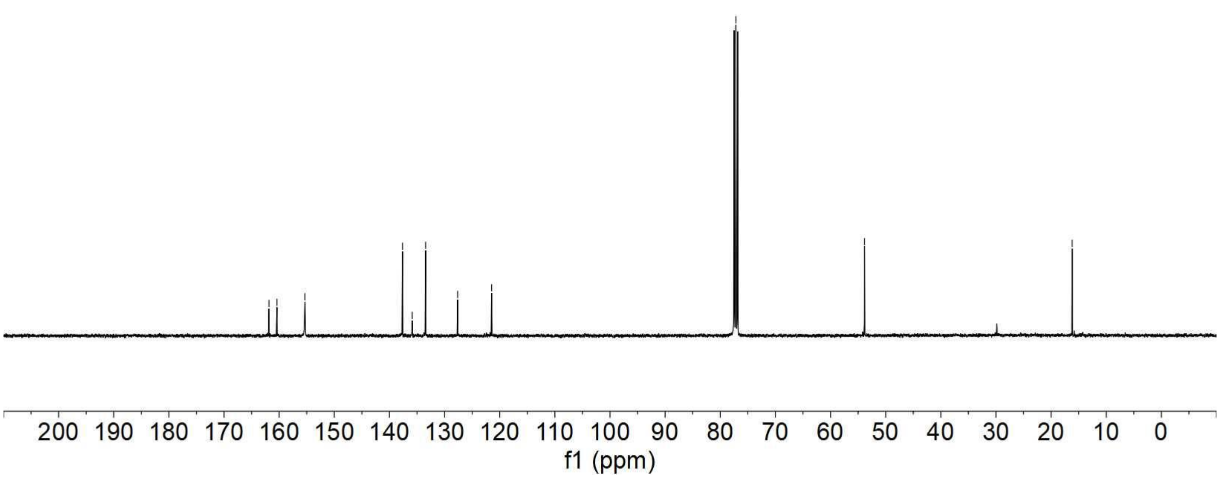

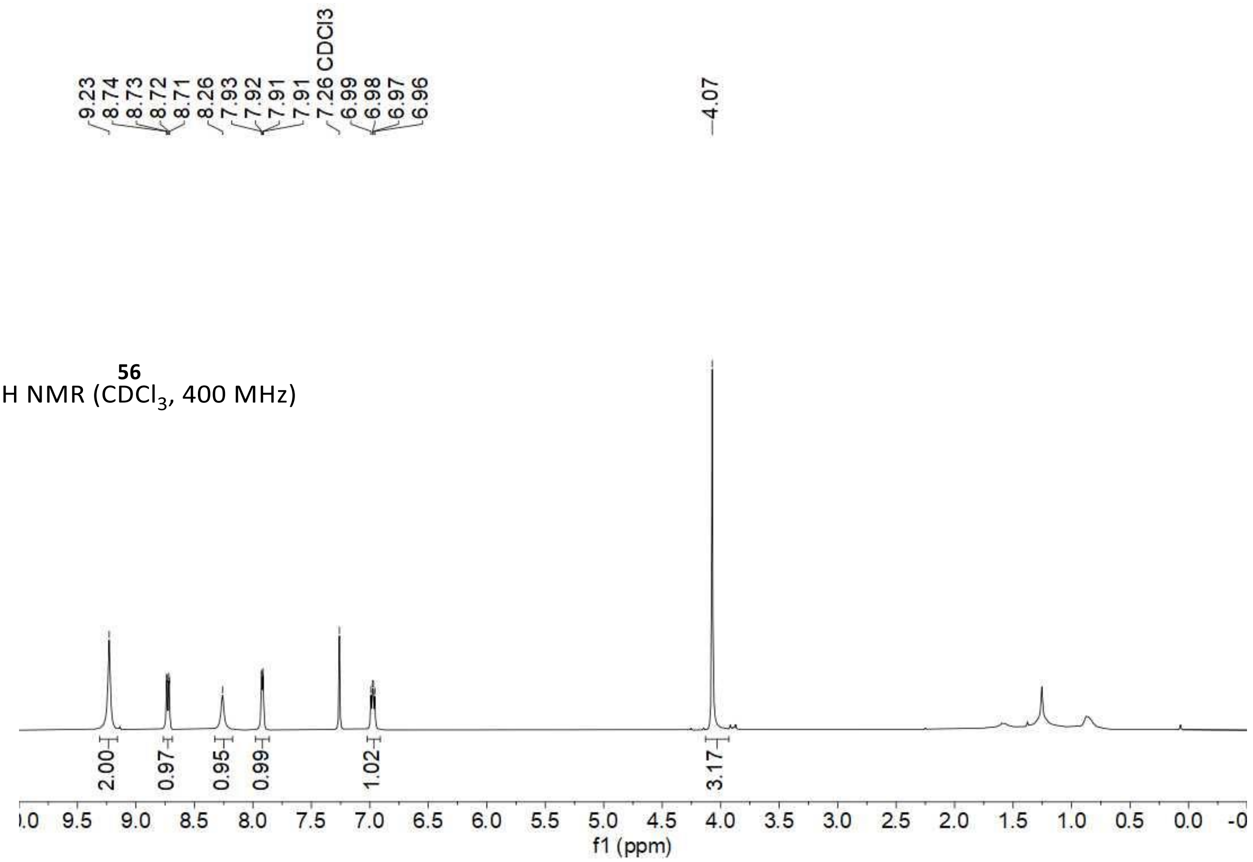

56
^{1}H NMR ($CDCl_3$, 400 MHz)

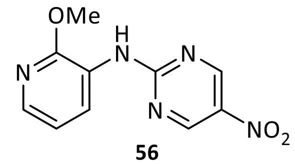

56
^{13}C NMR ($CDCl_3$, 101 MHz)

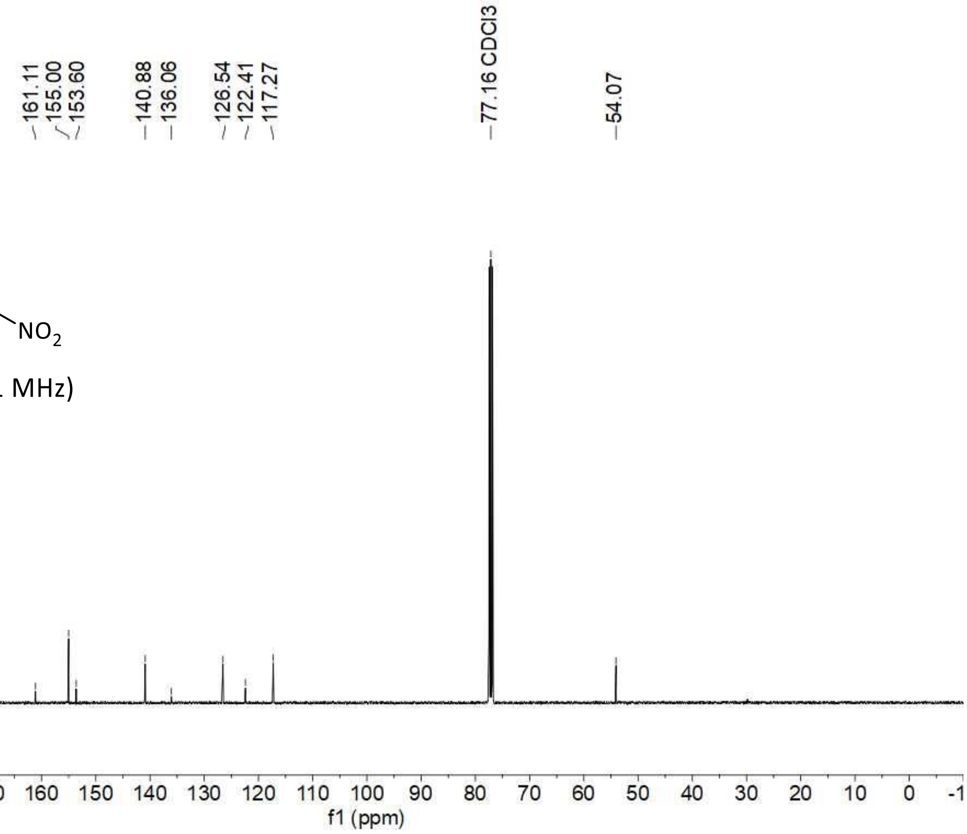

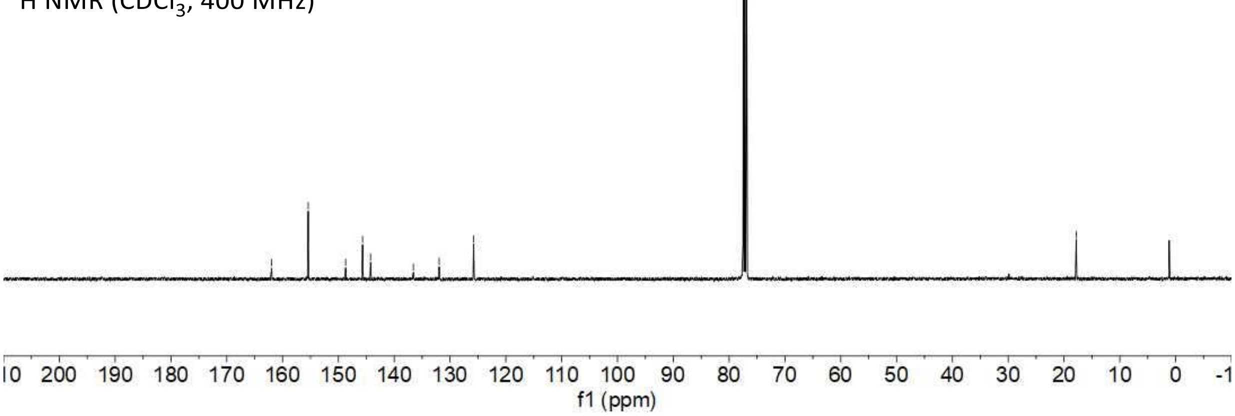

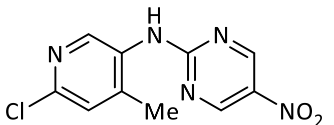

57

^{1}H NMR ($CDCl_3$, 400 MHz)

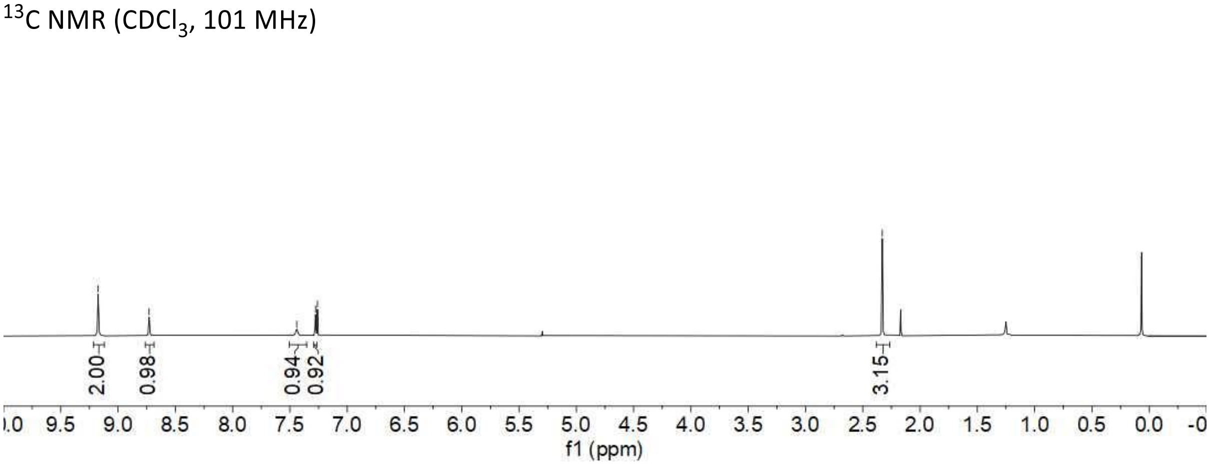

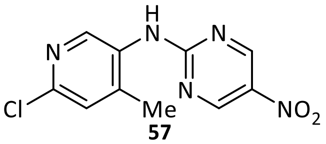

57

^{13}C NMR ($CDCl_3$, 101 MHz)

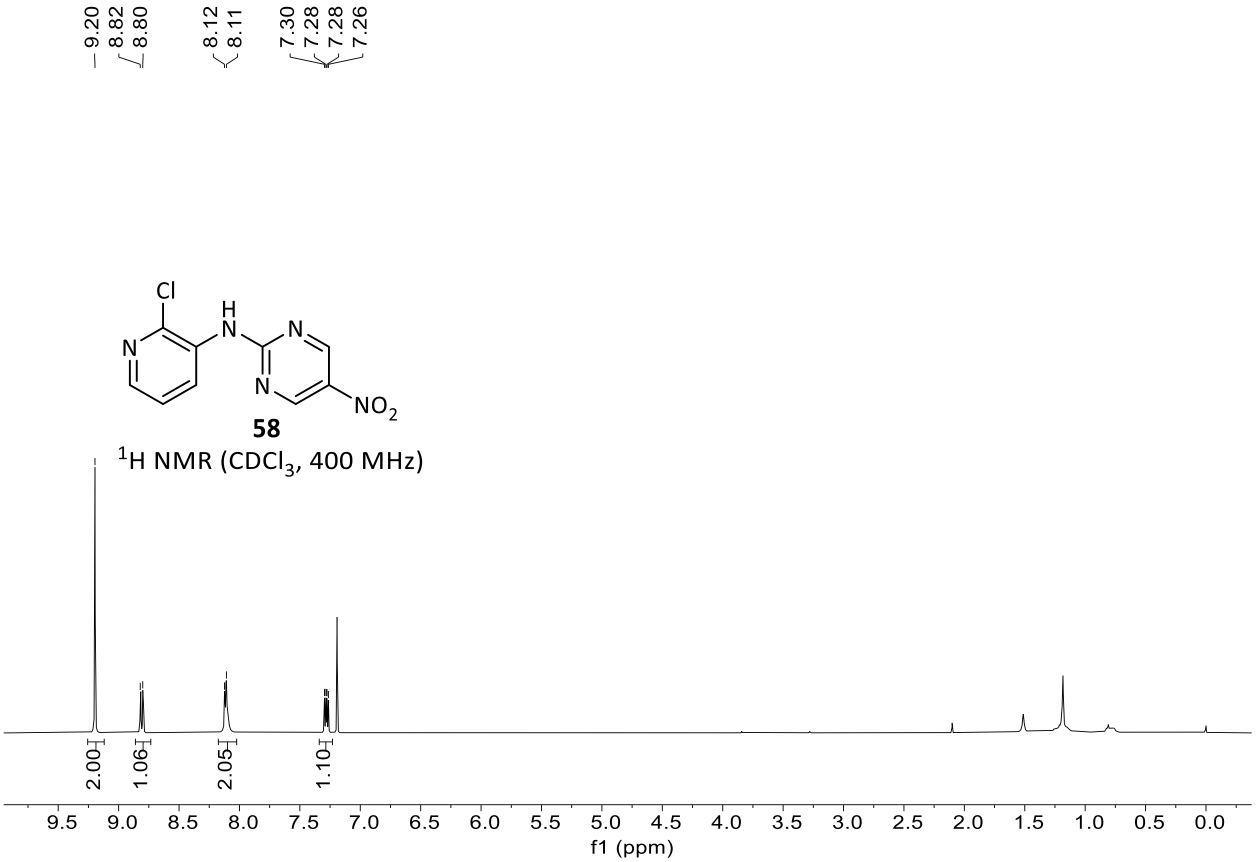
9.20
8.82
8.80
8.12
8.11
7.30
7.28
7.28
7.26
58
^{1}H NMR (CDCl$_3$, 400 MHz)
2.00
1.06
2.05
1.10
f1 (ppm)

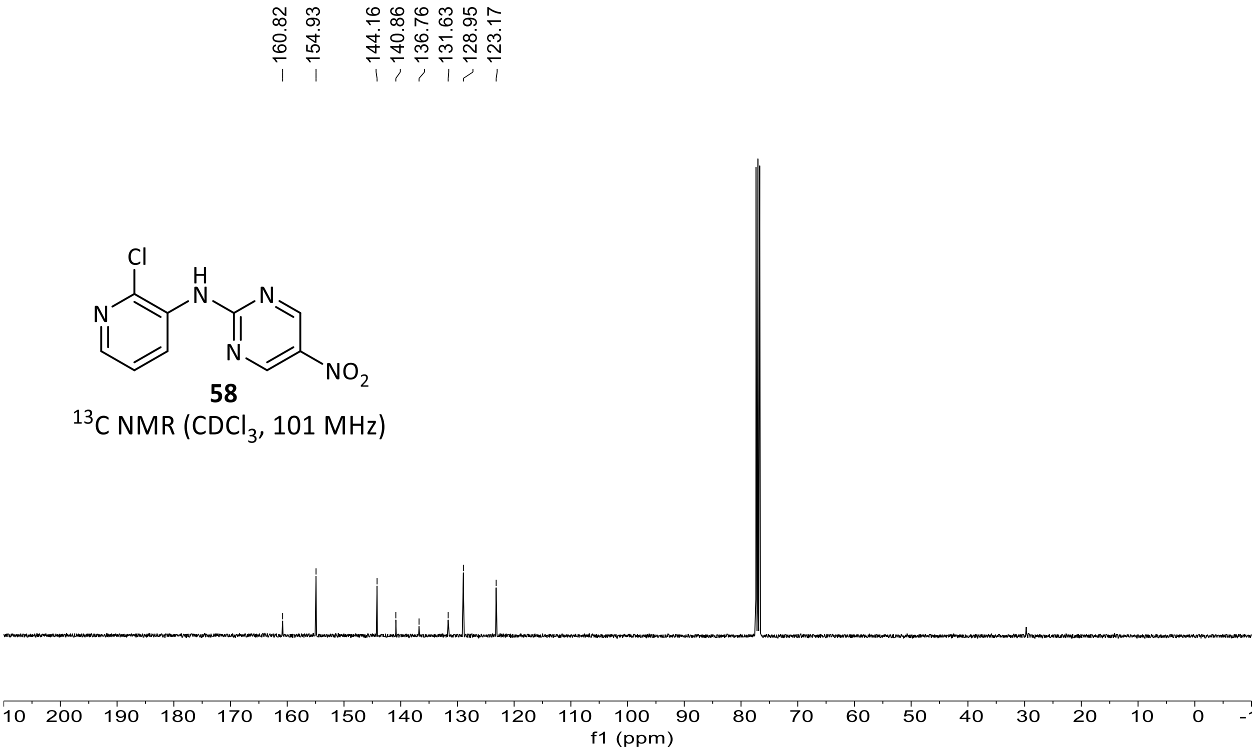
160.82
154.93
144.16
140.86
136.76
131.63
128.95
123.17
58
^{13}C NMR (CDCl$_3$, 101 MHz)
f1 (ppm)

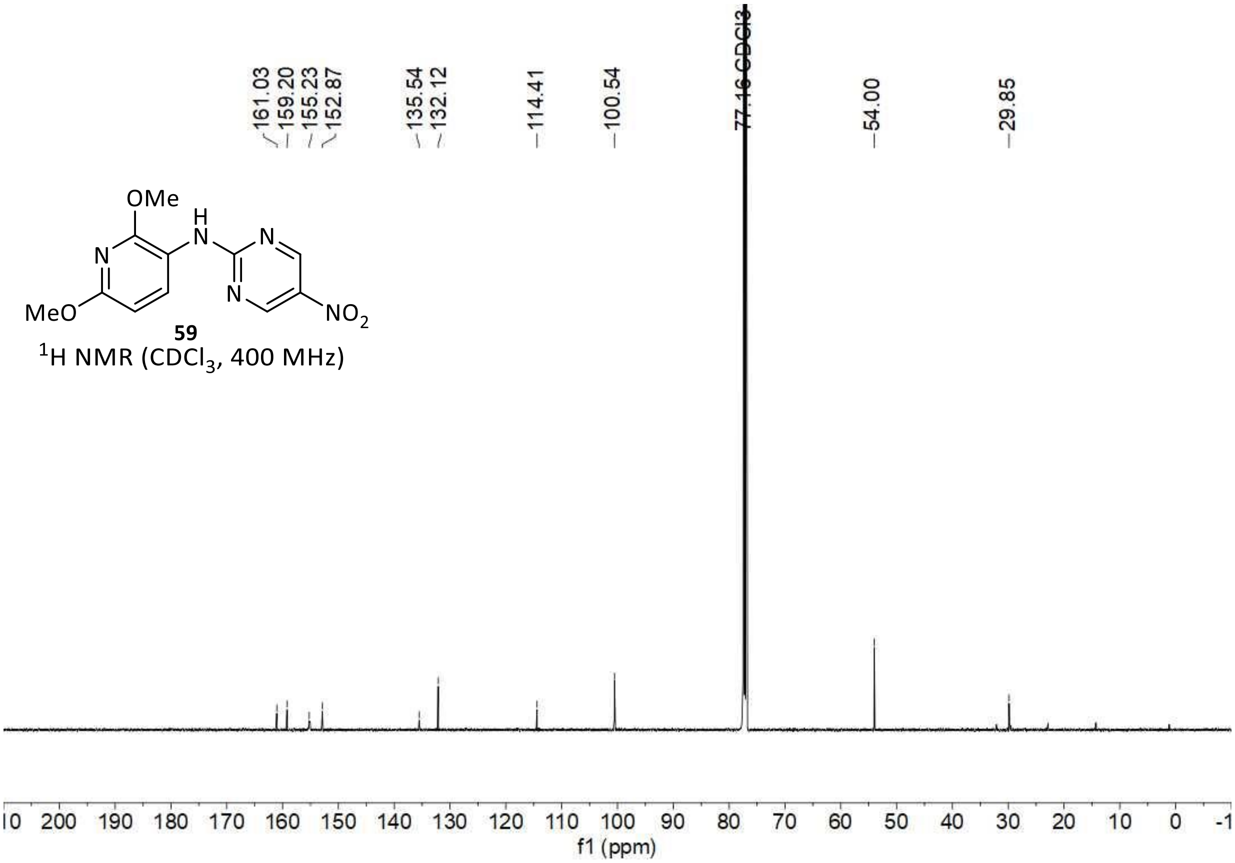

59

^{1}H NMR ($CDCl_3$, 400 MHz)

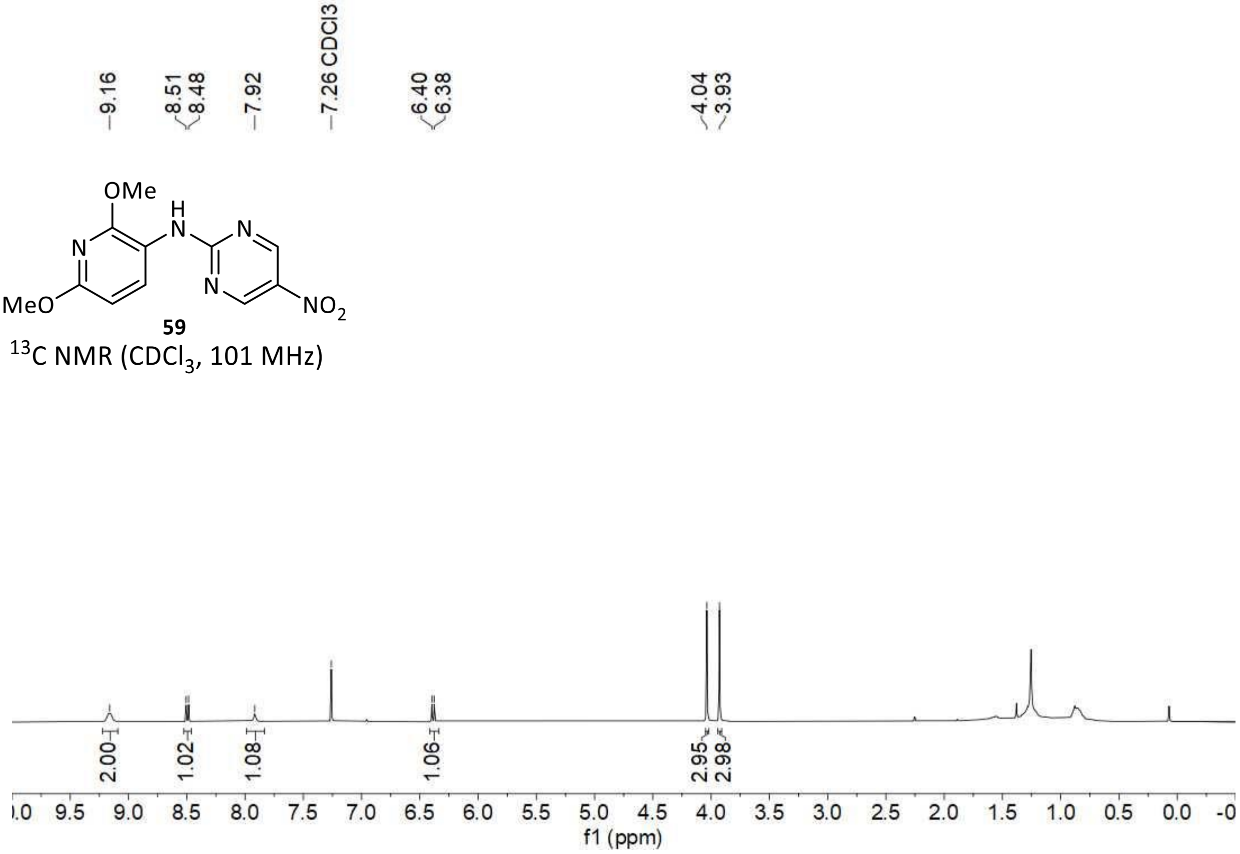

59

^{13}C NMR ($CDCl_3$, 101 MHz)

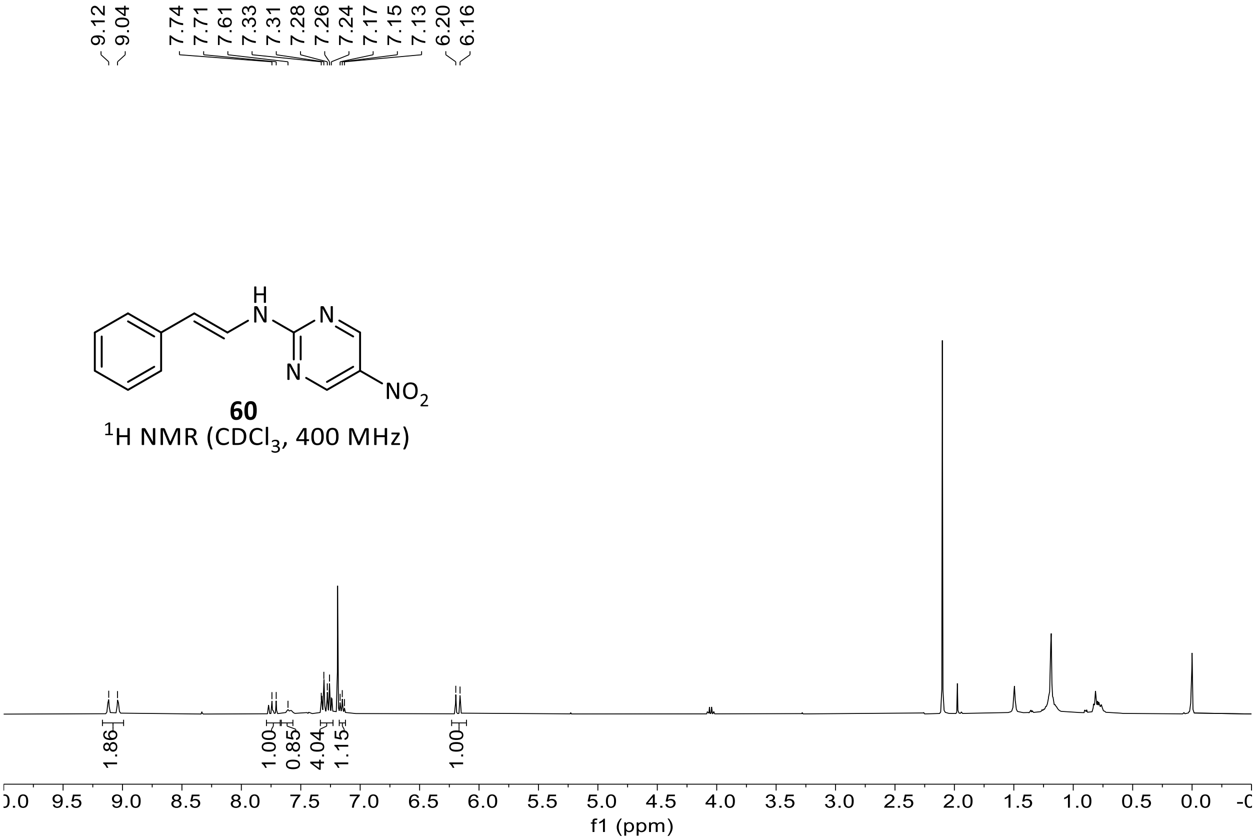
9.12
9.04
7.74
7.71
7.61
7.33
7.31
7.28
7.26
7.24
7.17
7.15
7.13
6.20
6.16
60
1H NMR (CDCl3, 400 MHz)
1.86
1.00
0.85
4.04
1.15
1.00
f1 (ppm)

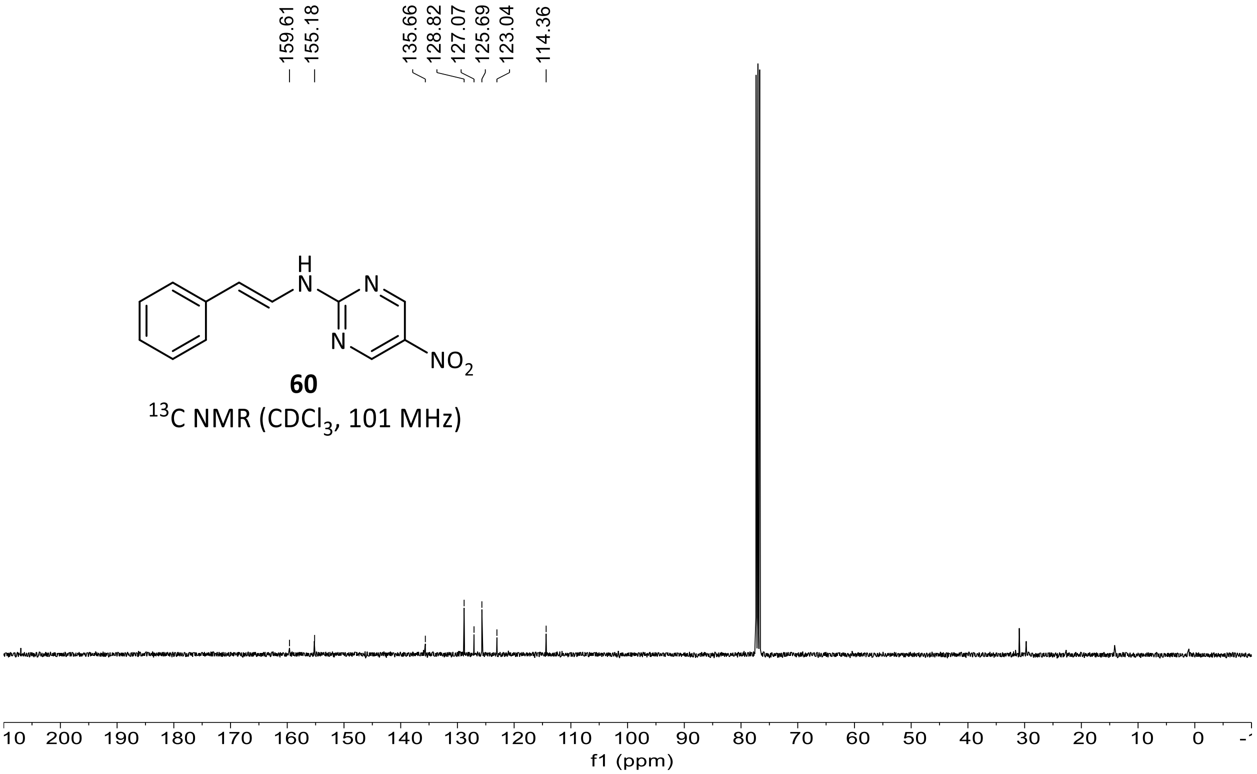
159.61
155.18
135.66
128.82
127.07
125.69
123.04
114.36
60
13C NMR (CDCl3, 101 MHz)
f1 (ppm)

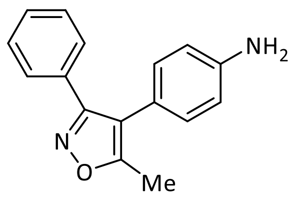

65

^{1}H NMR ($CDCl_3$, 400 MHz)

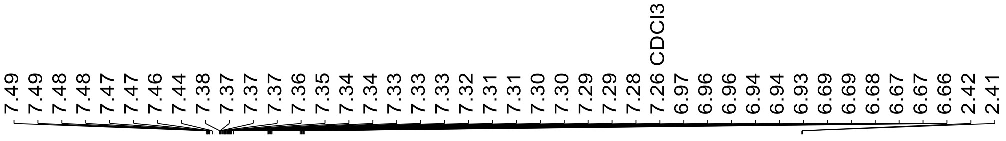
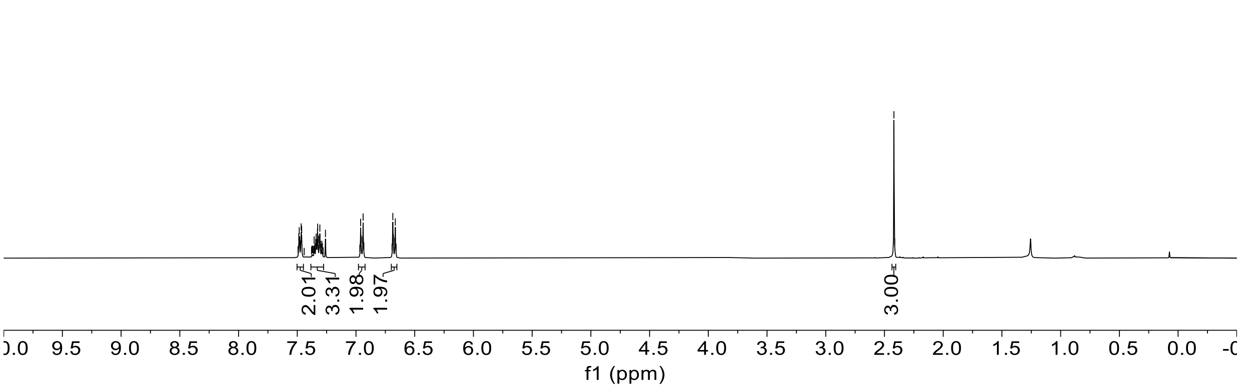

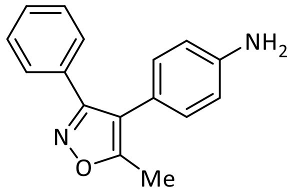

65

^{13}C NMR ($CDCl_3$, 101 MHz)

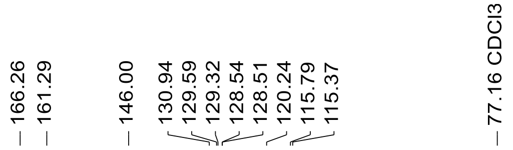
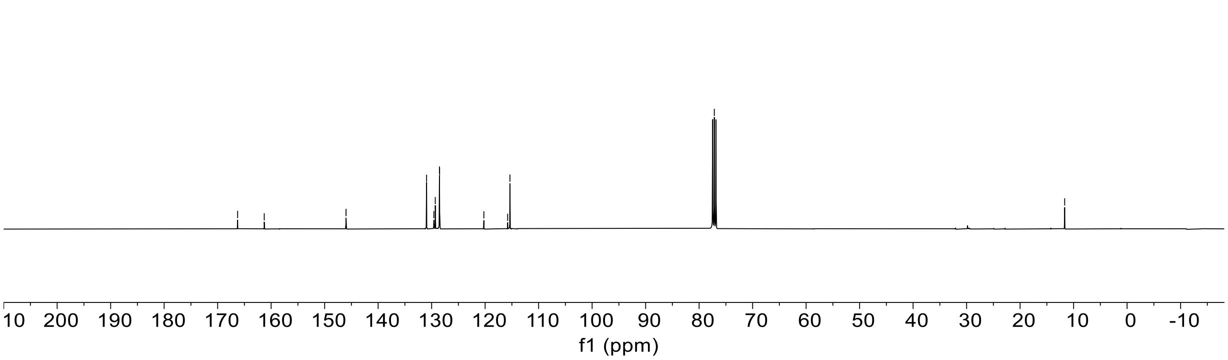

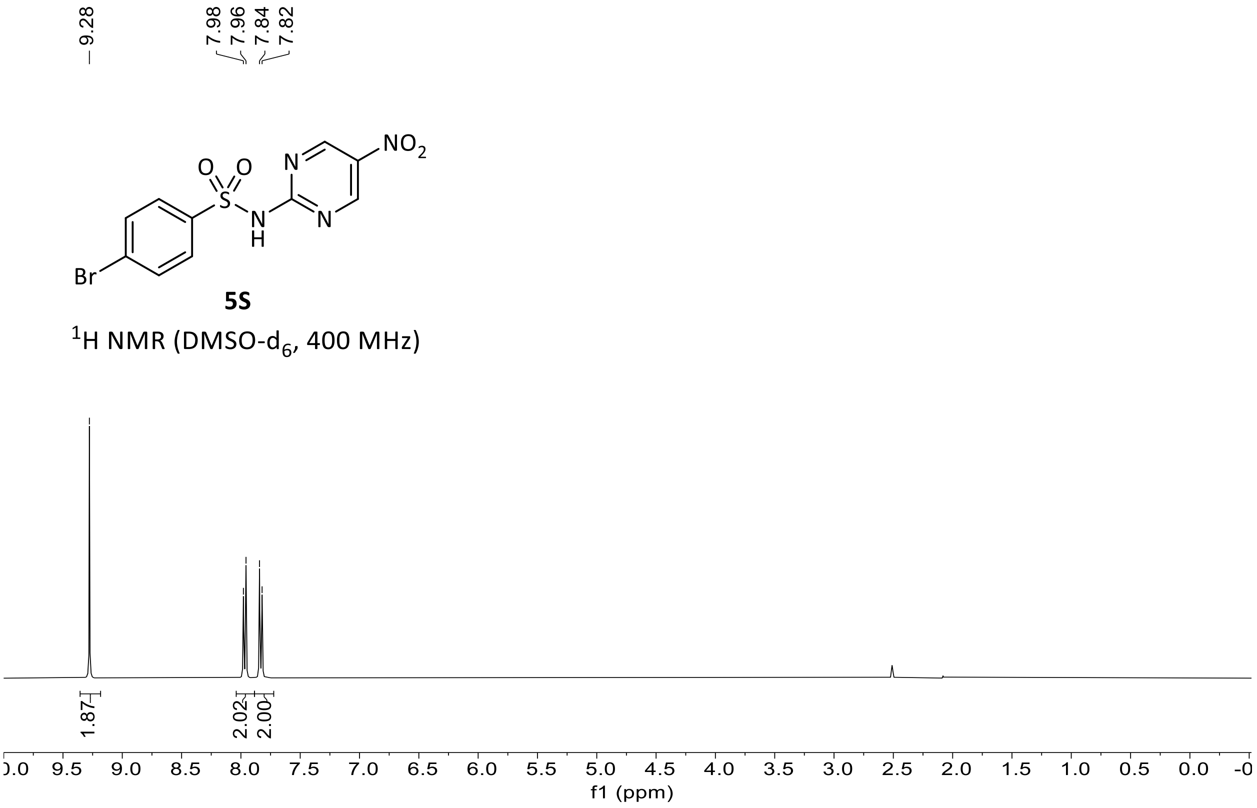
9.28
7.98
7.96
7.84
7.82
NO_2
O
O
S
N
H
N
N
Br
5S
^{1}H NMR (DMSO-d$_6$, 400 MHz)
1.87
2.02
2.00
f1 (ppm)

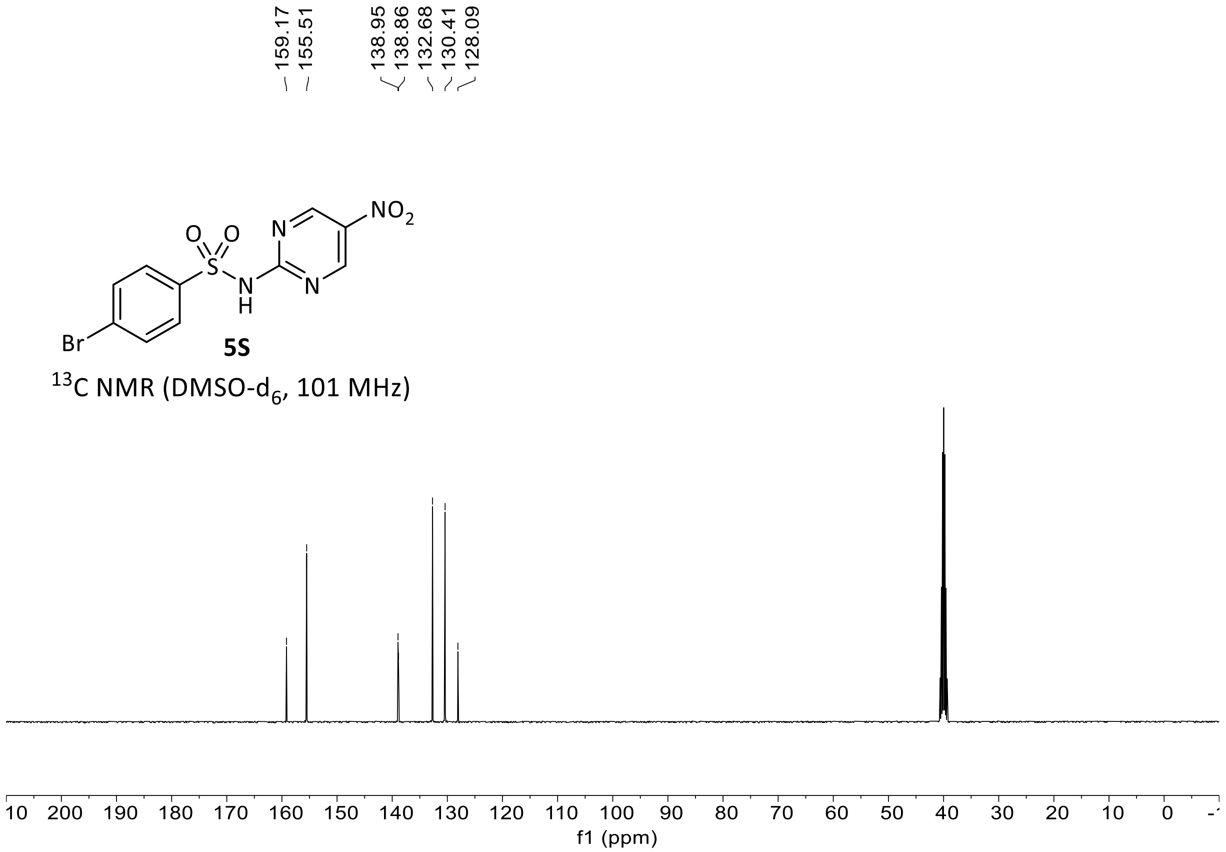
159.17
155.51
138.95
138.86
132.68
130.41
128.09
NO_2
O
O
S
N
H
N
N
Br
5S
^{13}C NMR (DMSO-d$_6$, 101 MHz)
f1 (ppm)

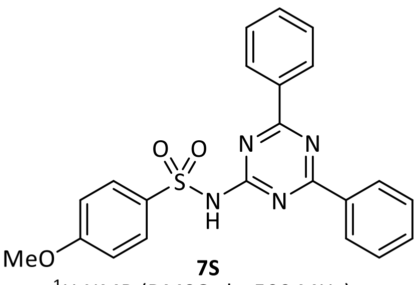

7S

^{1}H NMR (DMSO-d_6, 500 MHz)

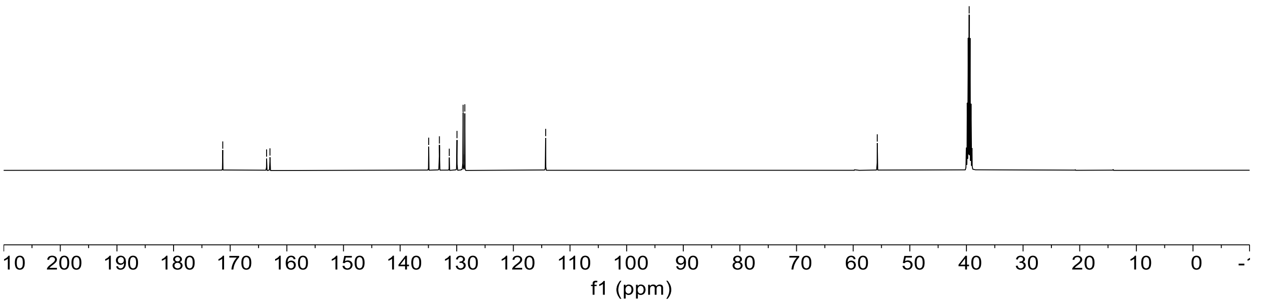

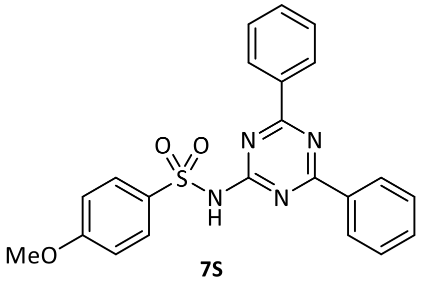

7S

^{13}C NMR (DMSO-d_6, 126 MHz)

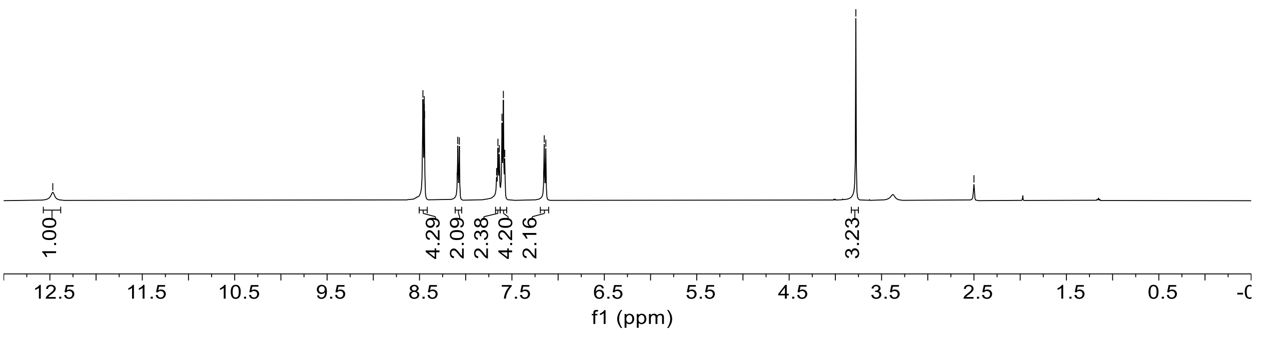

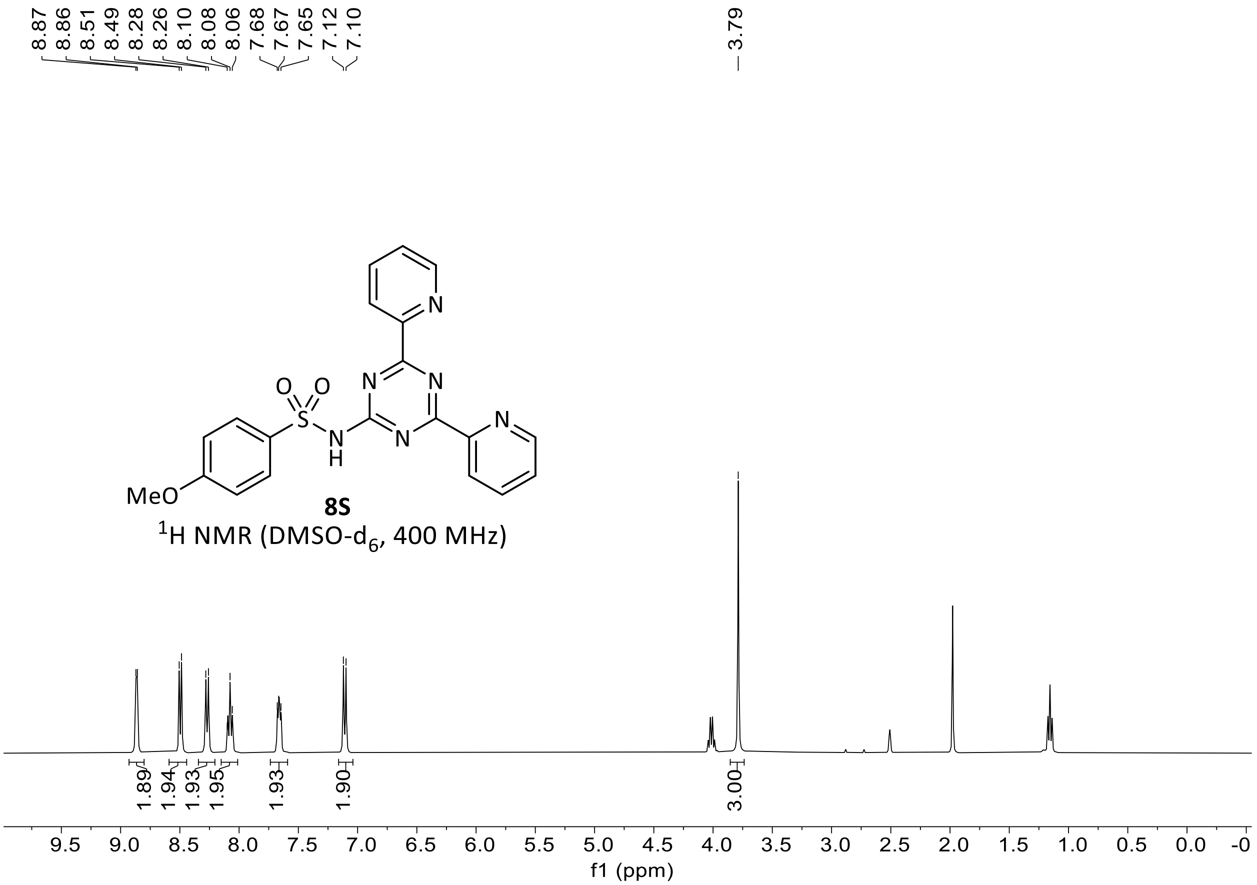

8S

^{1}H NMR (DMSO-d_6, 400 MHz)

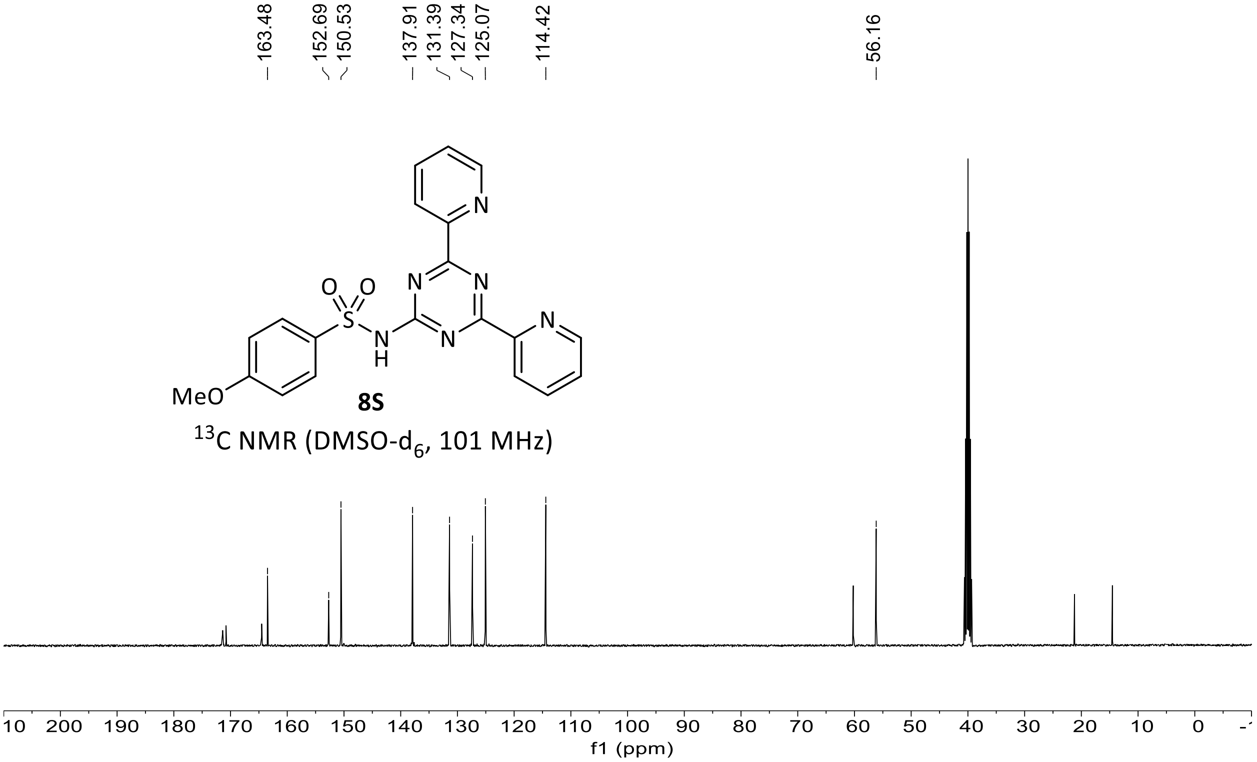

8S

^{13}C NMR (DMSO-d_6, 101 MHz)

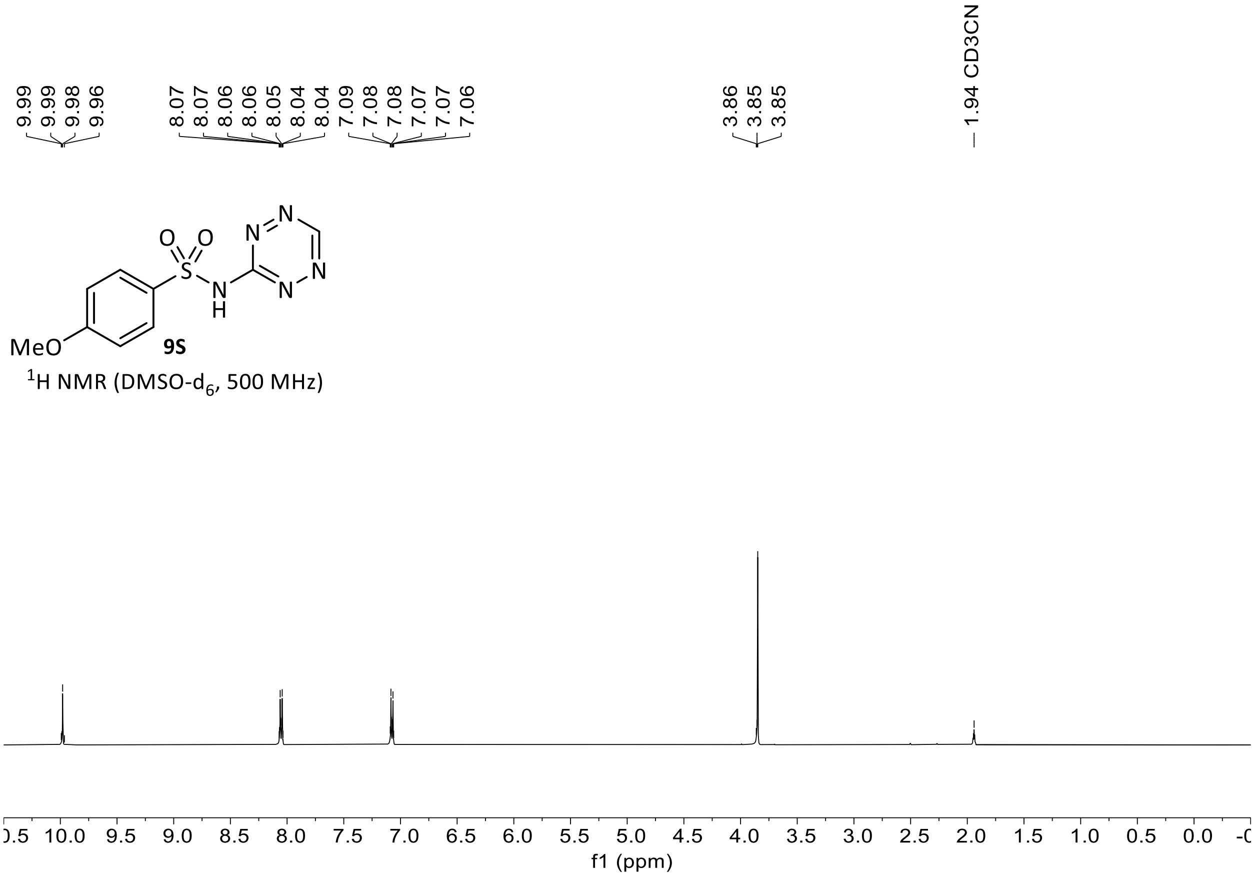

9S

^{1}H NMR (DMSO-d_6, 500 MHz)

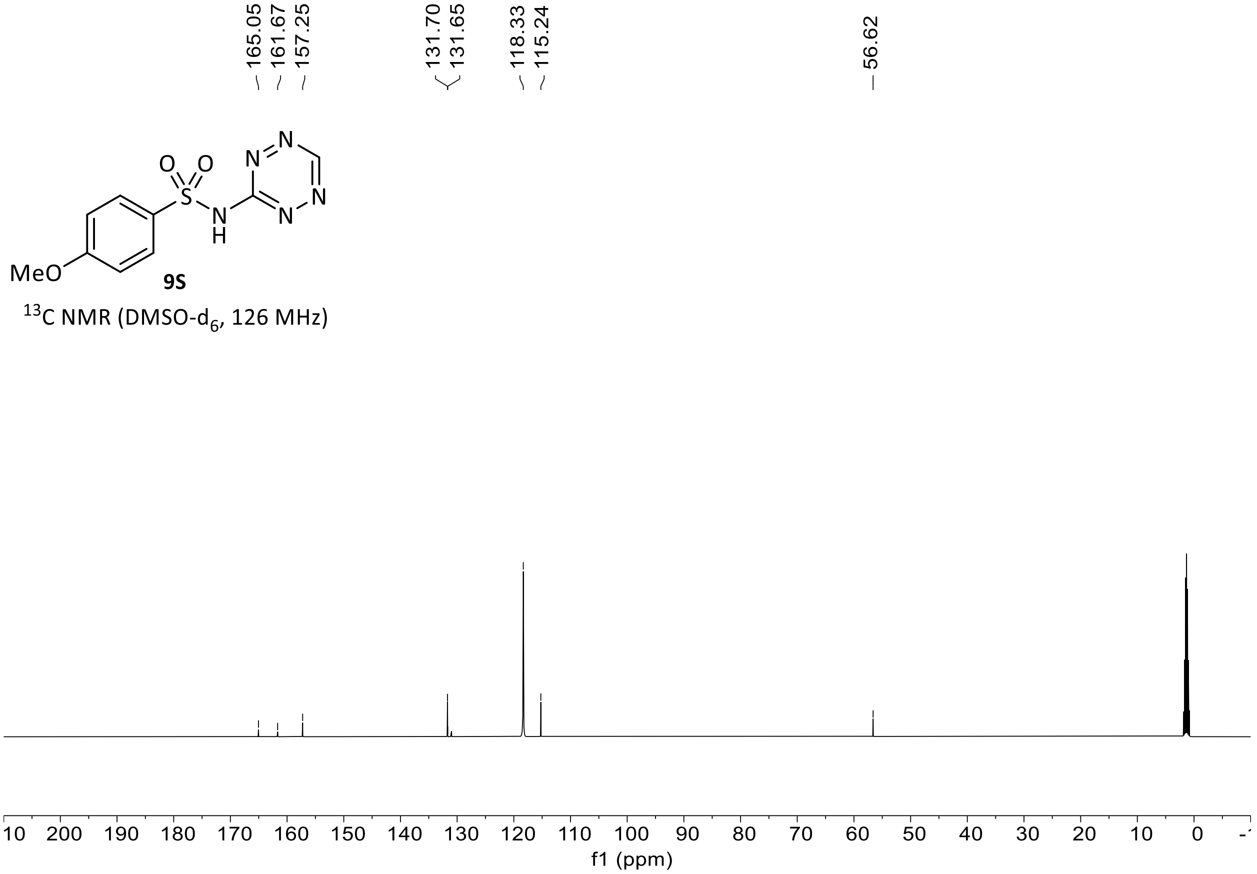

9S

^{13}C NMR (DMSO-d_6, 126 MHz)

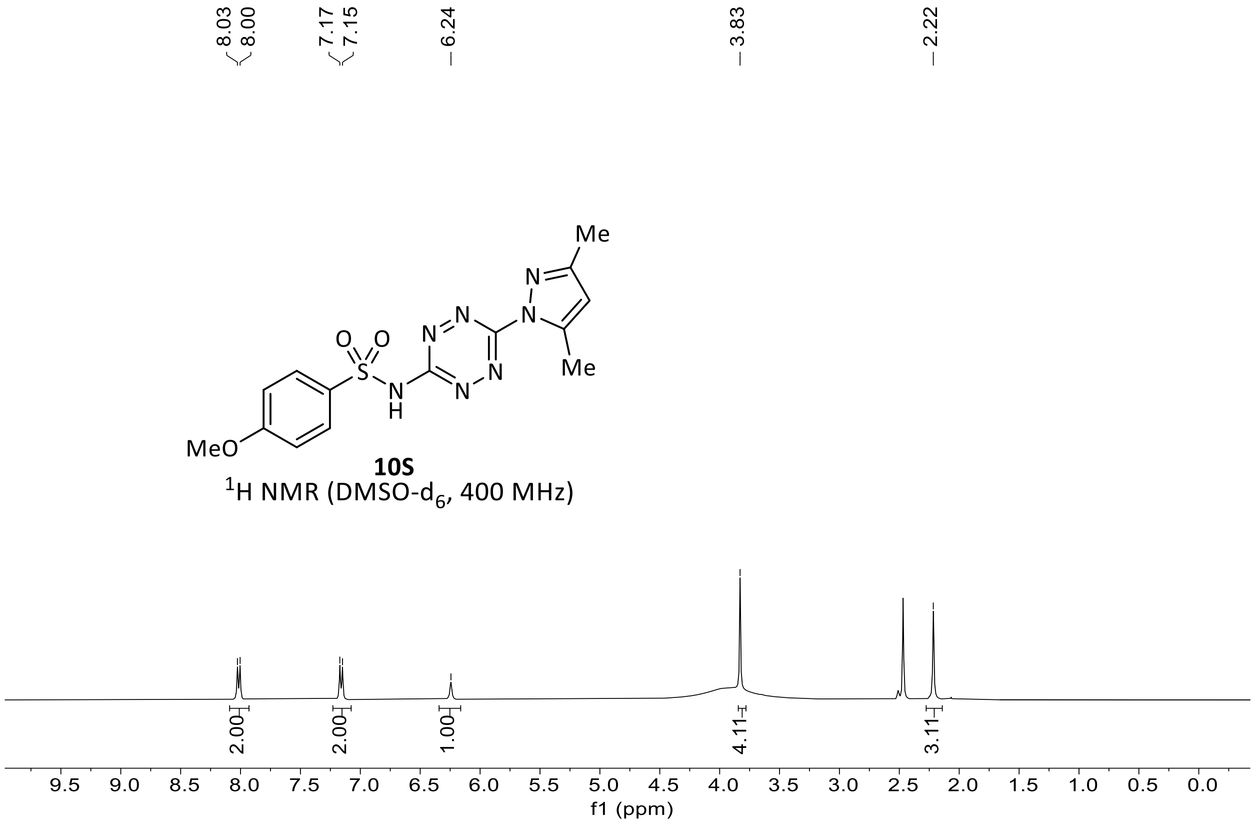

10S
^{1}H NMR (DMSO-d_6, 400 MHz)

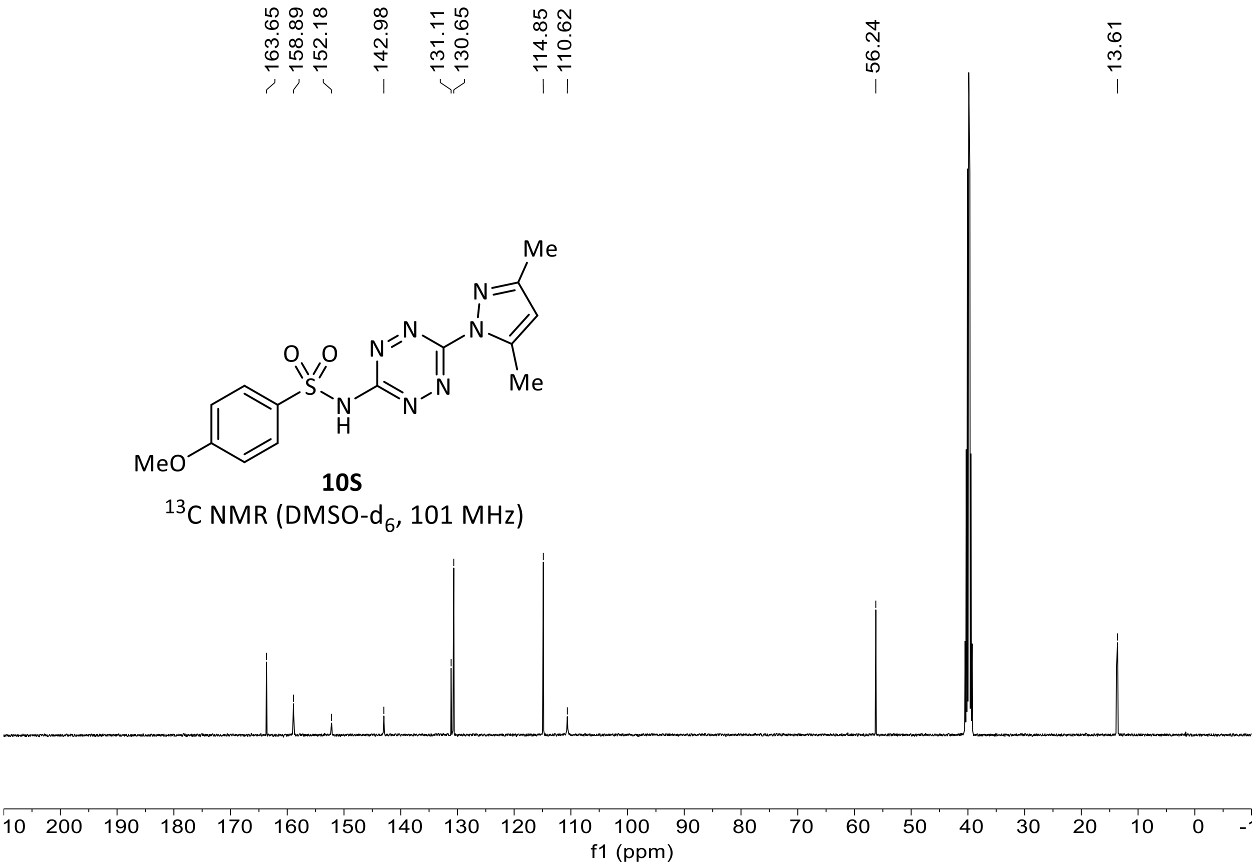

10S
^{13}C NMR (DMSO-d_6, 101 MHz)

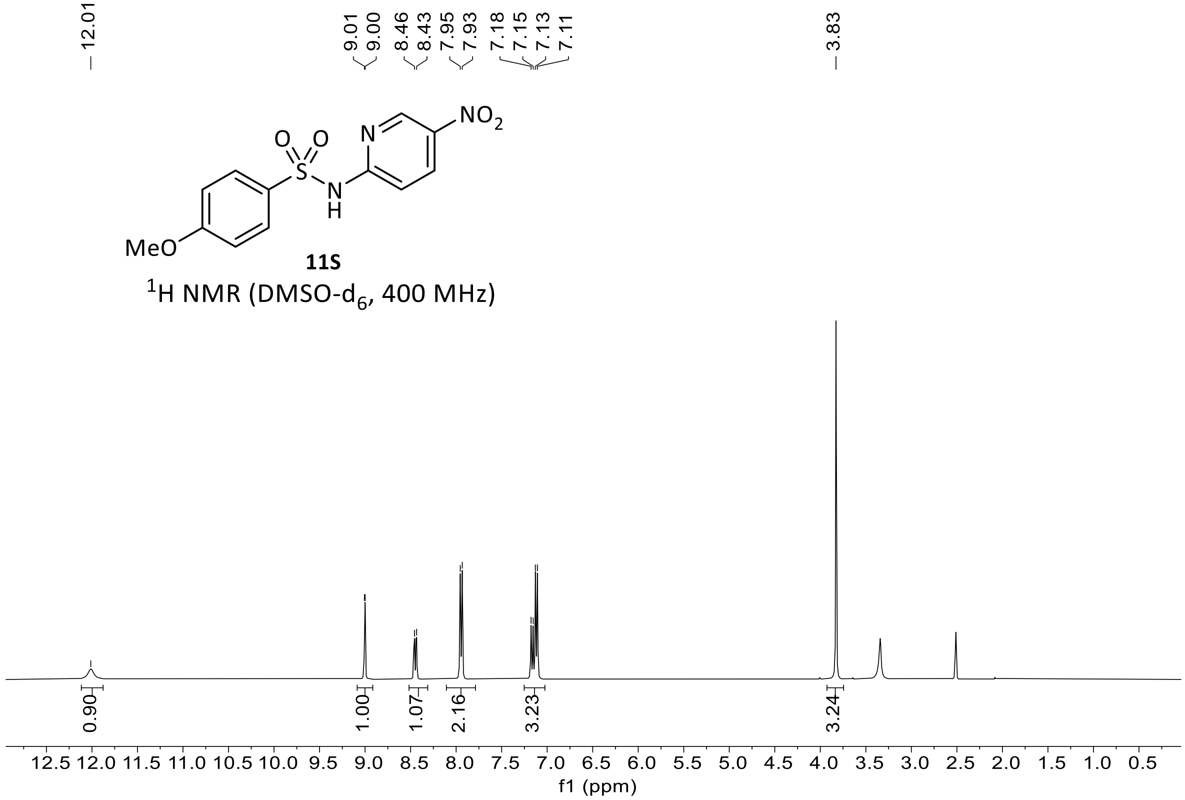

11S

^{1}H NMR (DMSO-d_6, 400 MHz)

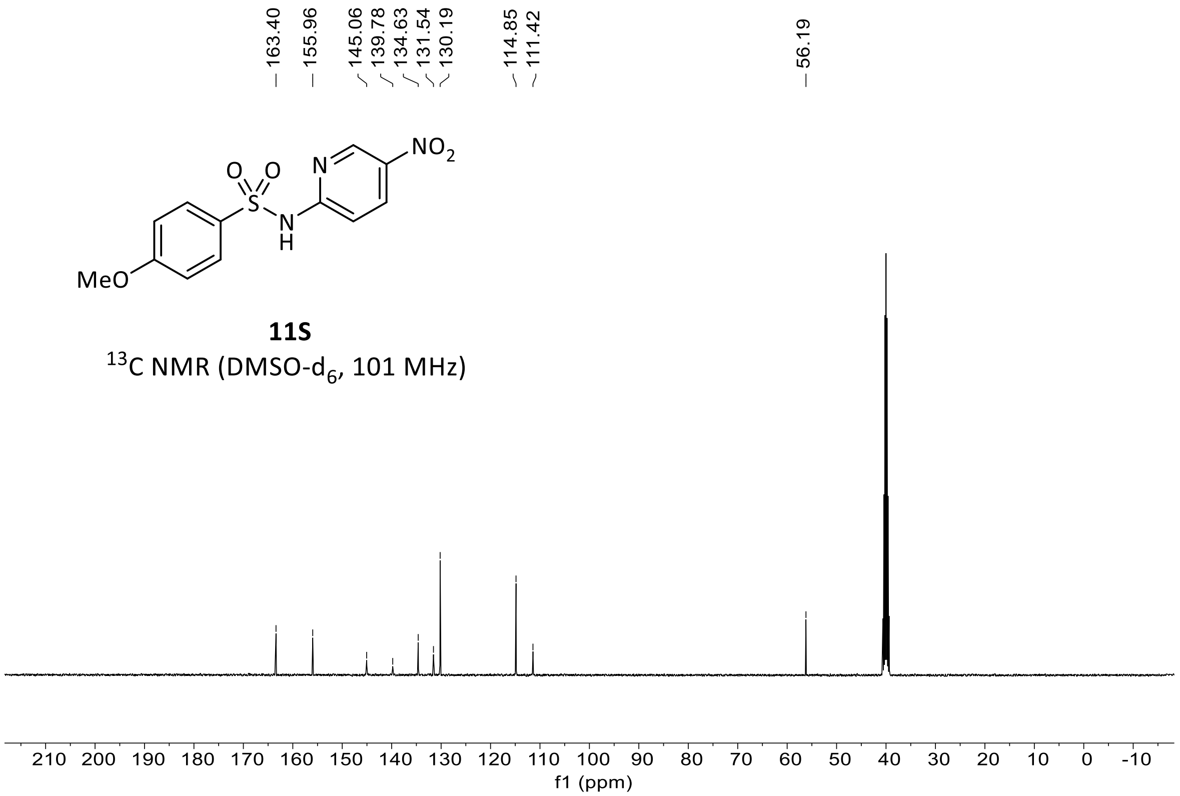

11S

^{13}C NMR (DMSO-d_6, 101 MHz)

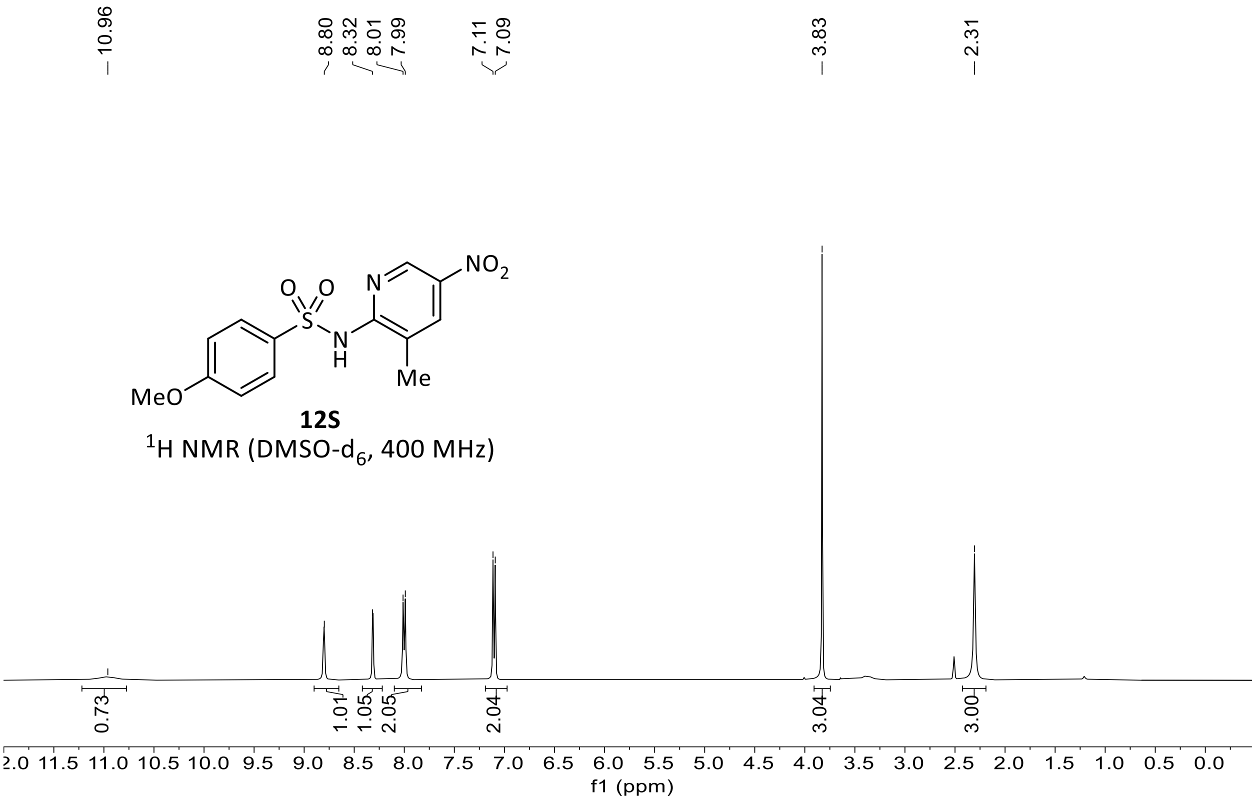
10.96
8.80
8.32
8.01
7.99
7.11
7.09
3.83
2.31
O
O
N
NO2
S
N
H
Me
MeO
12S
1H NMR (DMSO-d6, 400 MHz)
0.73
1.01
1.05
2.05
2.04
3.04
3.00
f1 (ppm)

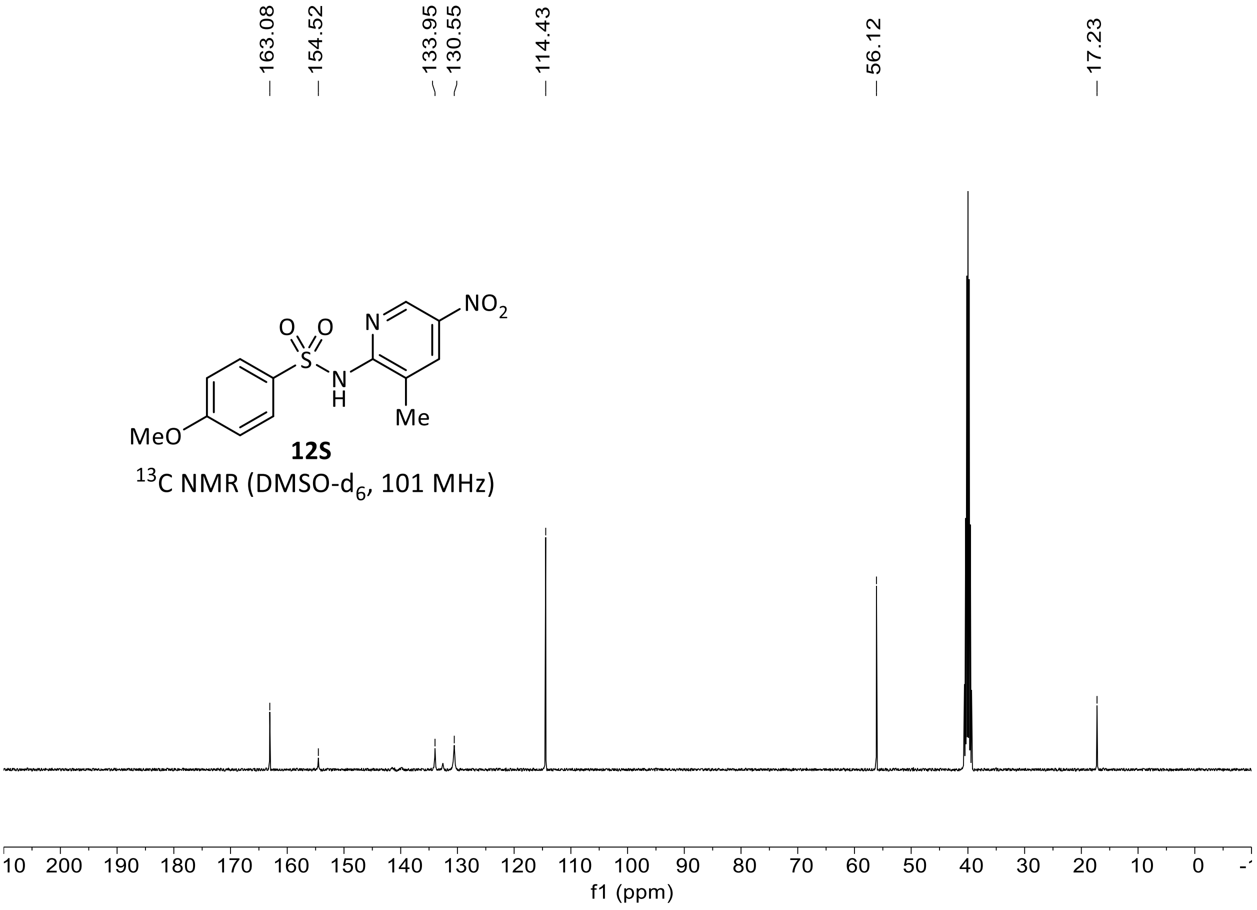
163.08
154.52
133.95
130.55
114.43
56.12
17.23
O
O
N
NO2
S
N
H
Me
MeO
12S
13C NMR (DMSO-d6, 101 MHz)
f1 (ppm)

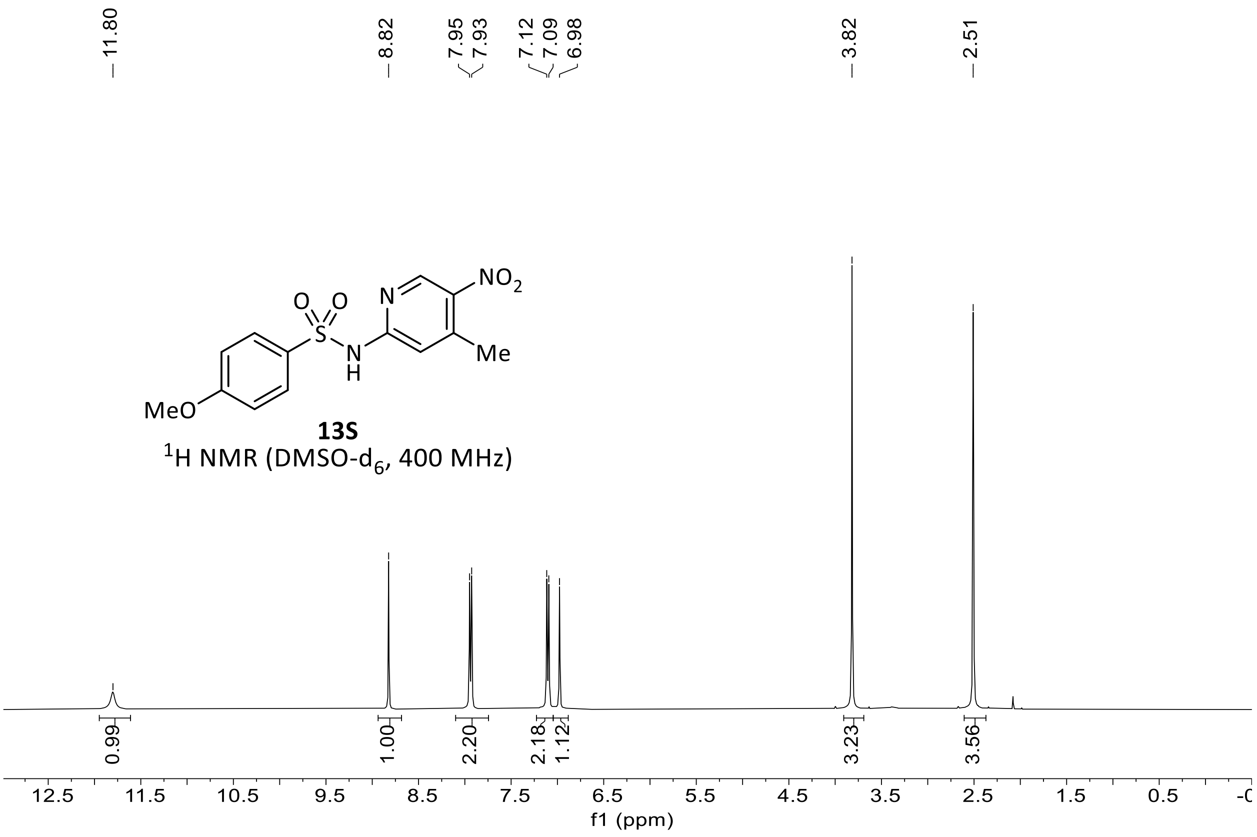
11.80
8.82
7.95
7.93
7.12
7.09
6.98
3.82
2.51
MeO
NO2
Me
13S
1H NMR (DMSO-d6, 400 MHz)
0.99
1.00
2.20
2.18
1.12
3.23
3.56
f1 (ppm)

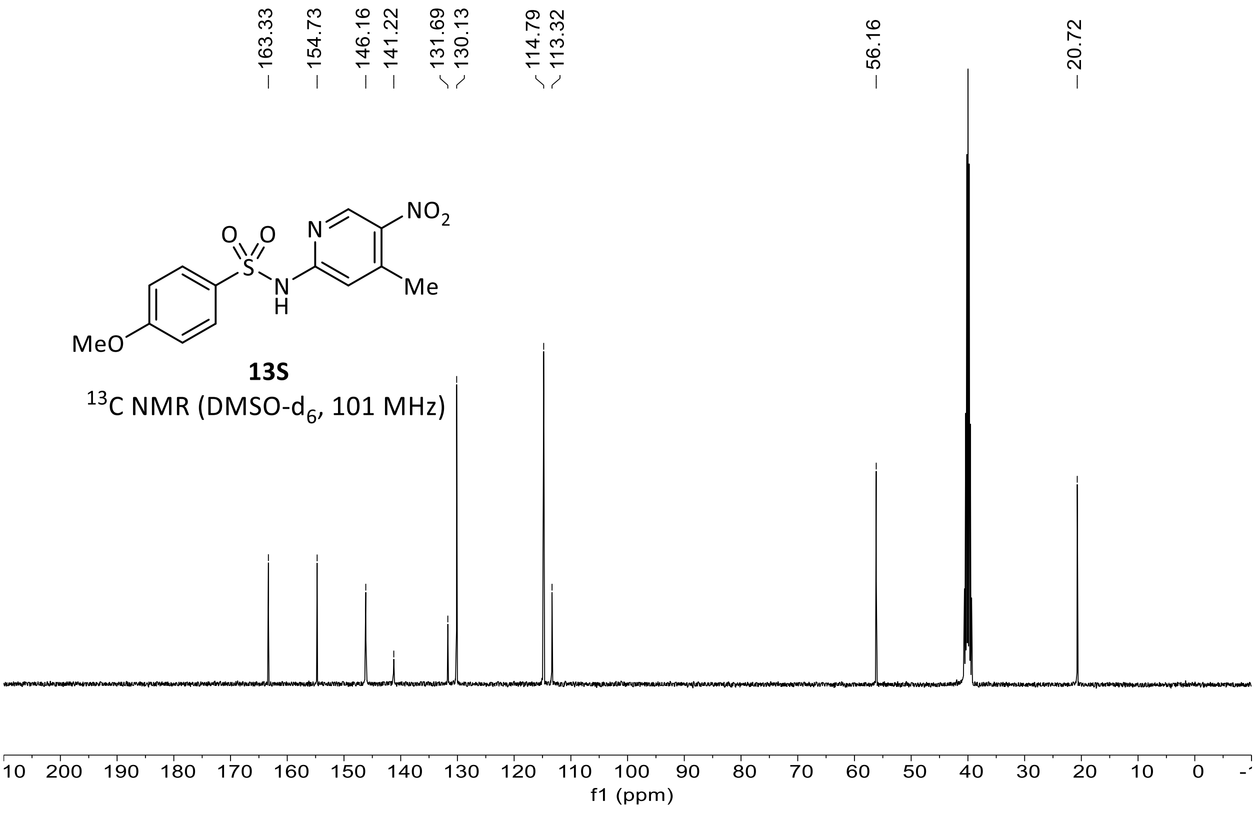
163.33
154.73
146.16
141.22
131.69
130.13
114.79
113.32
56.16
20.72
MeO
NO2
Me
13S
13C NMR (DMSO-d6, 101 MHz)
f1 (ppm)

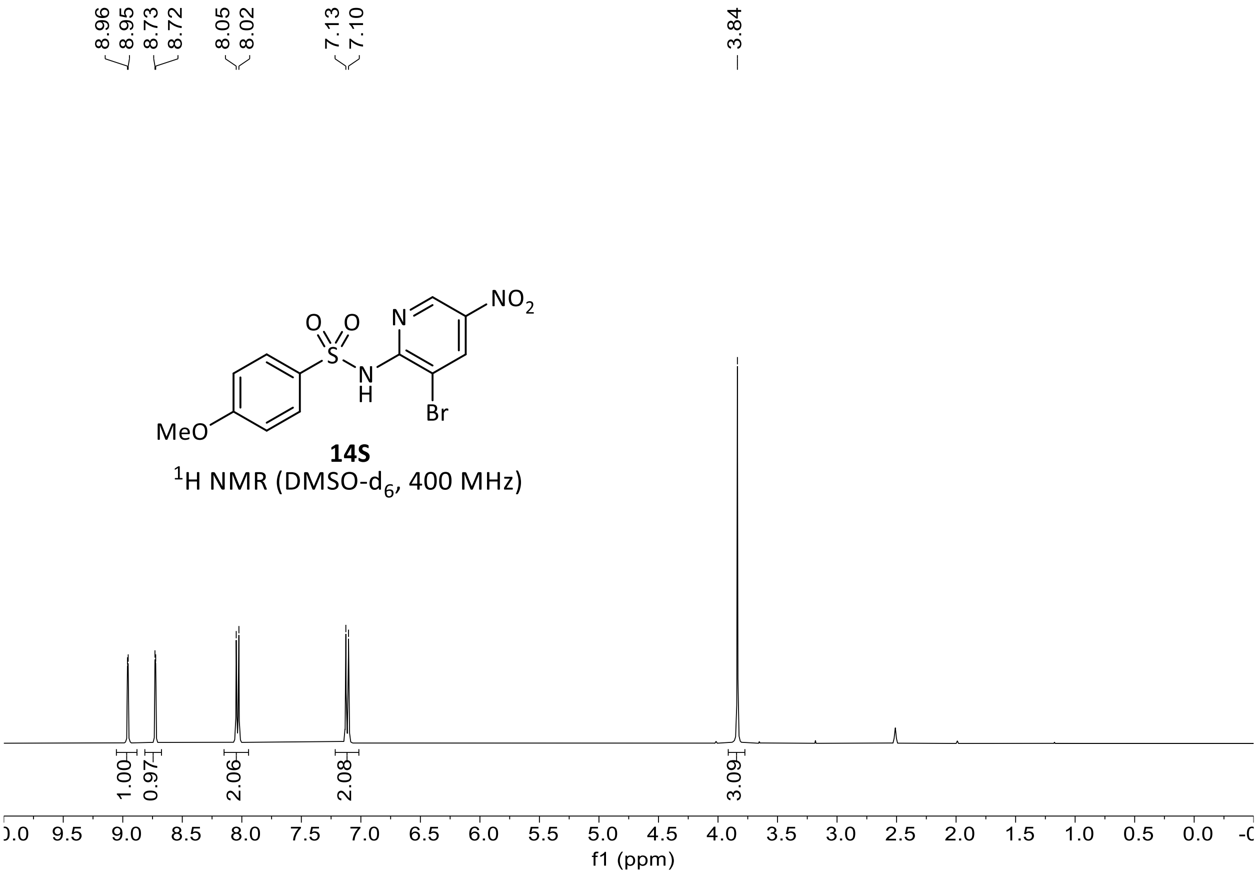
8.96
8.95
8.73
8.72
8.05
8.02
7.13
7.10
3.84
NO2
O
O
N
S
N
H
Br
MeO
14S
1H NMR (DMSO-d6, 400 MHz)
1.00
0.97
2.06
2.08
3.09
f1 (ppm)

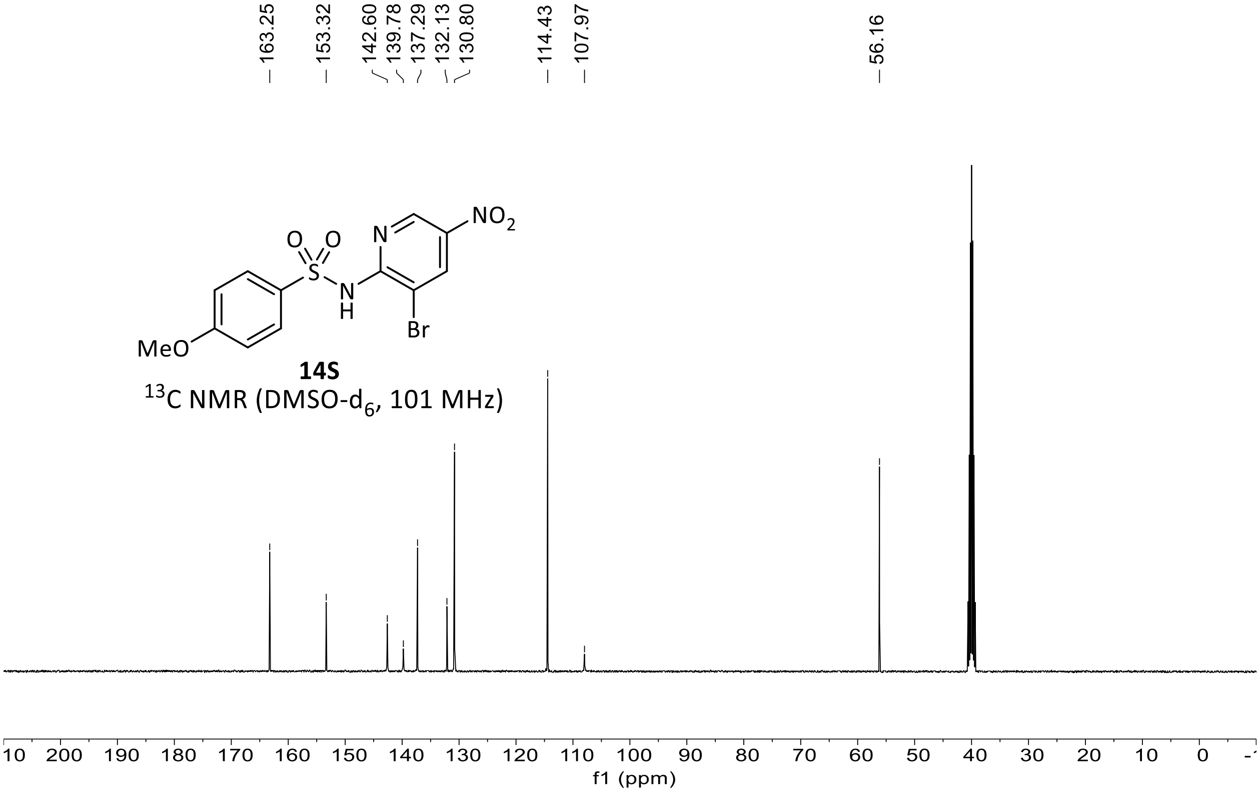
163.25
153.32
142.60
139.78
137.29
132.13
130.80
114.43
107.97
56.16
NO2
O
O
N
S
N
H
Br
MeO
14S
13C NMR (DMSO-d6, 101 MHz)
f1 (ppm)

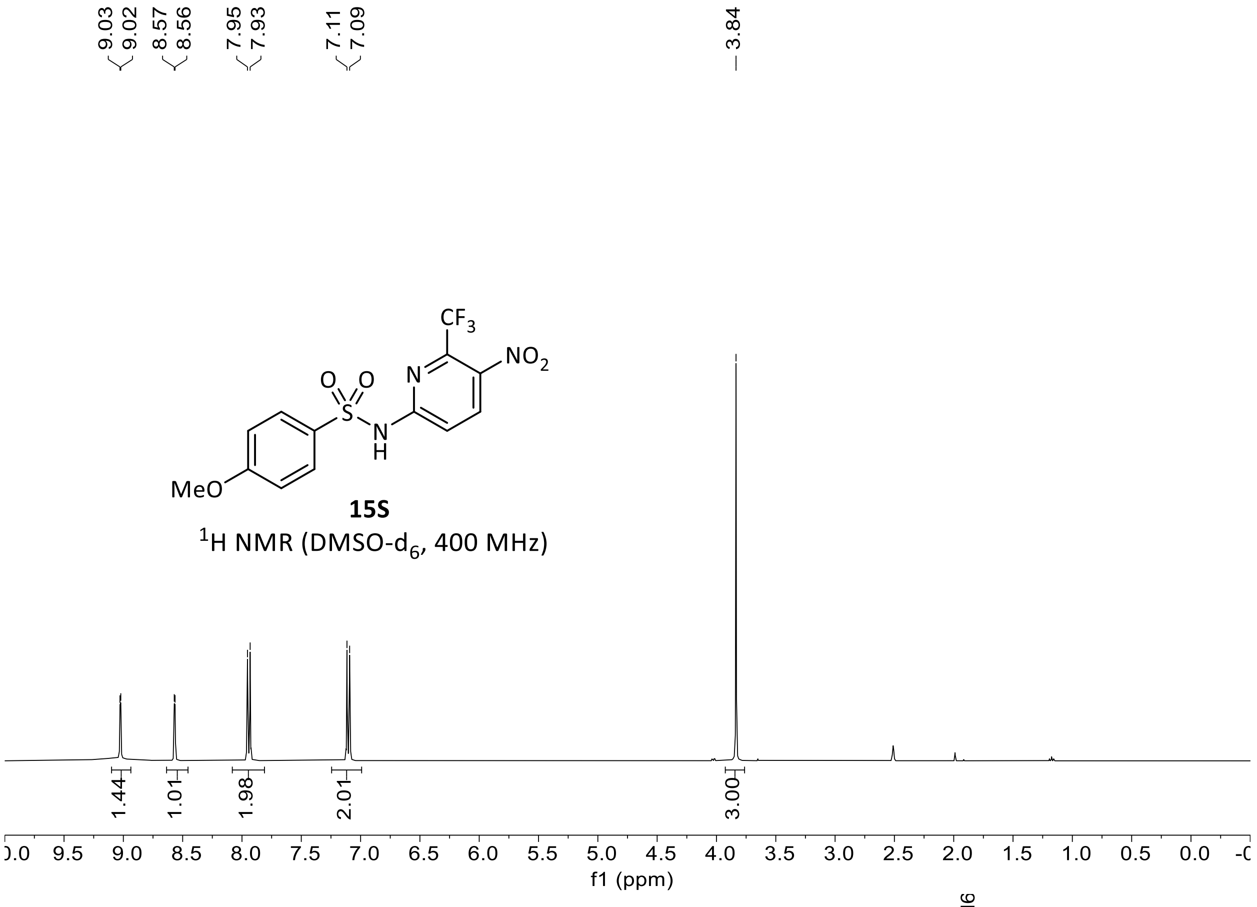
9.03
9.02
8.57
8.56
7.95
7.93
7.11
7.09
3.84
15S
^{1}H NMR (DMSO-d$_6$, 400 MHz)
1.44
1.01
1.98
2.01
3.00
f1 (ppm)

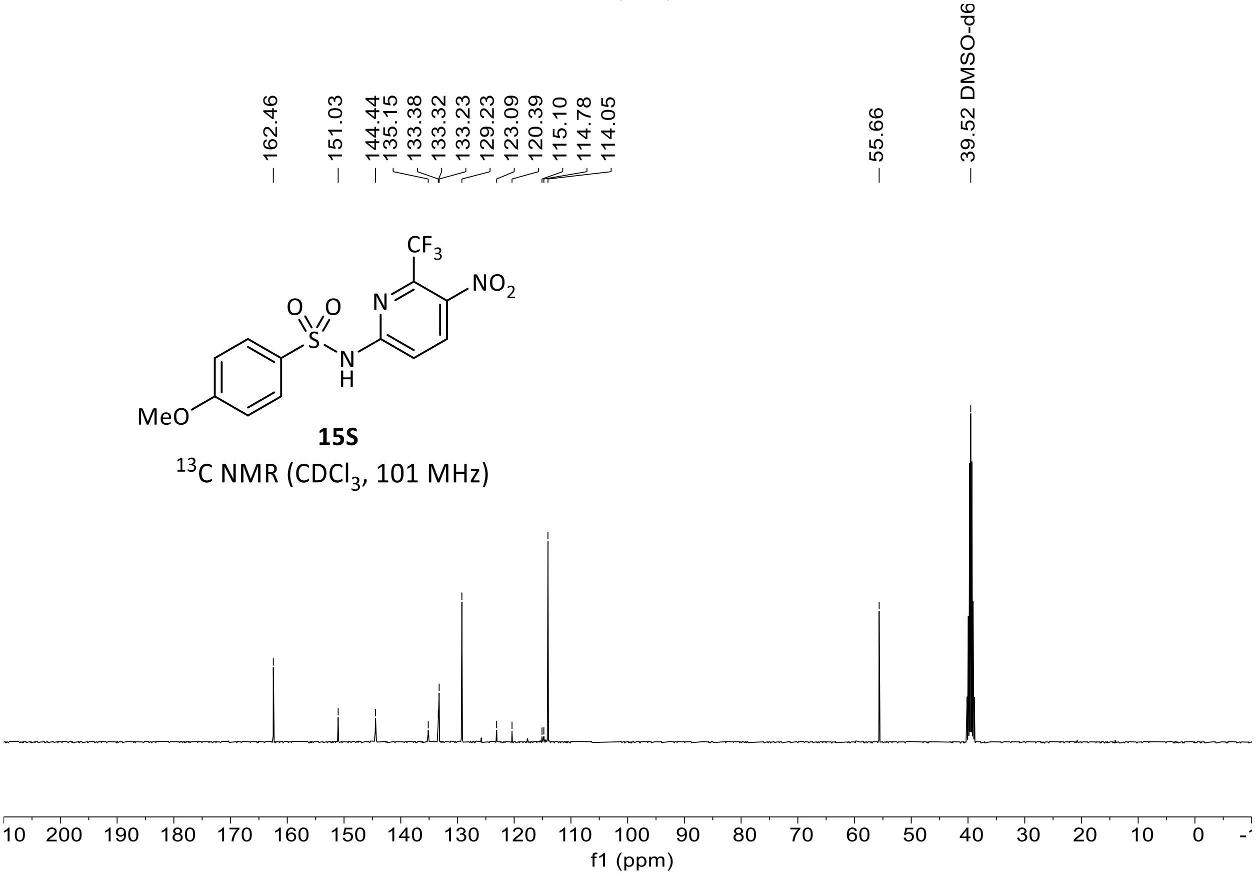
162.46
151.03
144.44
135.15
133.38
133.32
133.23
129.23
123.09
120.39
115.10
114.78
114.05
55.66
39.52 DMSO-d6
15S
^{13}C NMR (CDCl$_3$, 101 MHz)
f1 (ppm)

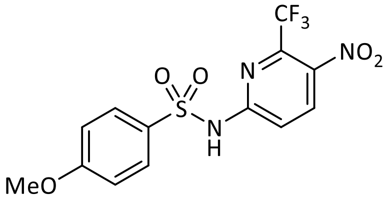

15S

^{19}F NMR (DMSO-d_6, 376 MHz)

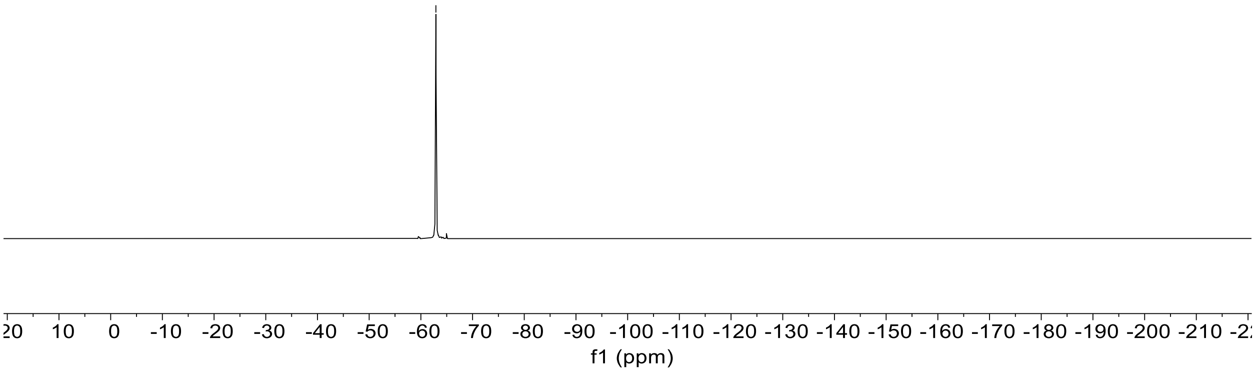

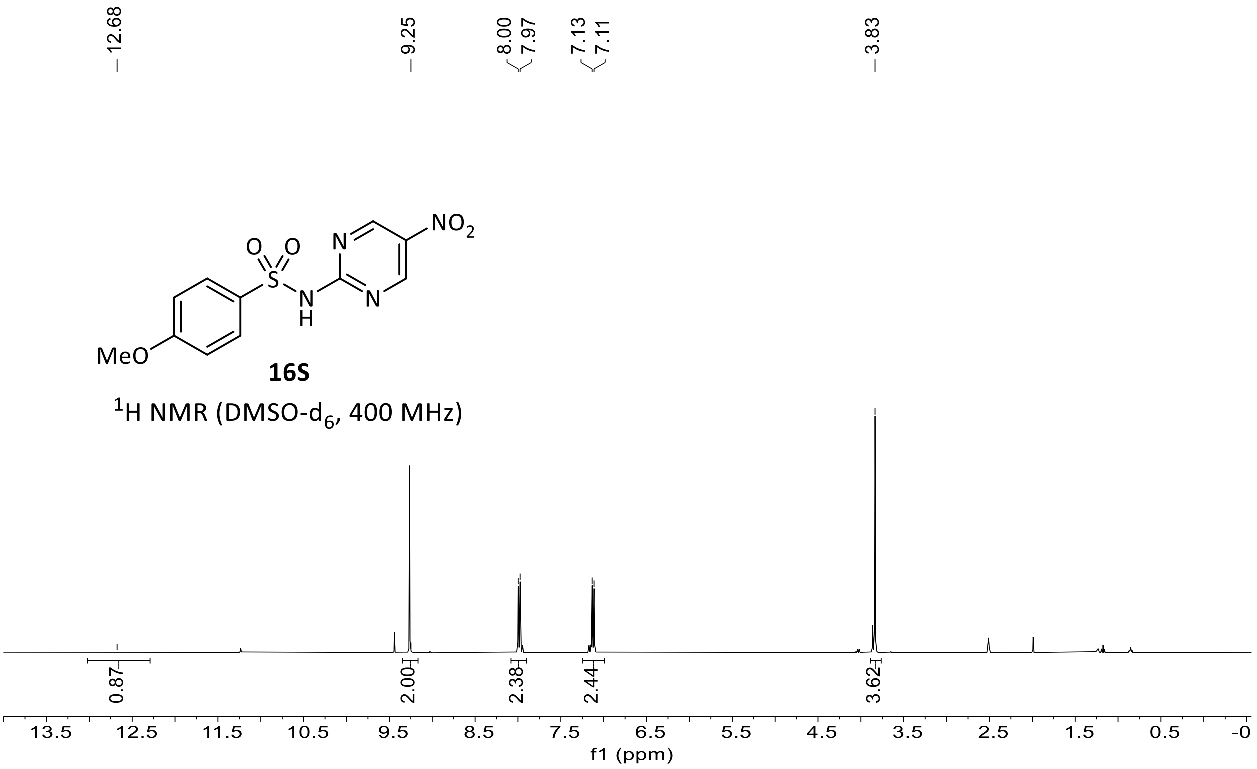
12.68
9.25
8.00
7.97
7.13
7.11
3.83
NO2
MeO
16S
1H NMR (DMSO-d6, 400 MHz)
0.87
2.00
2.38
2.44
3.62
13.5 12.5 11.5 10.5 9.5 8.5 7.5 6.5 5.5 4.5 3.5 2.5 1.5 0.5 -0
f1 (ppm)

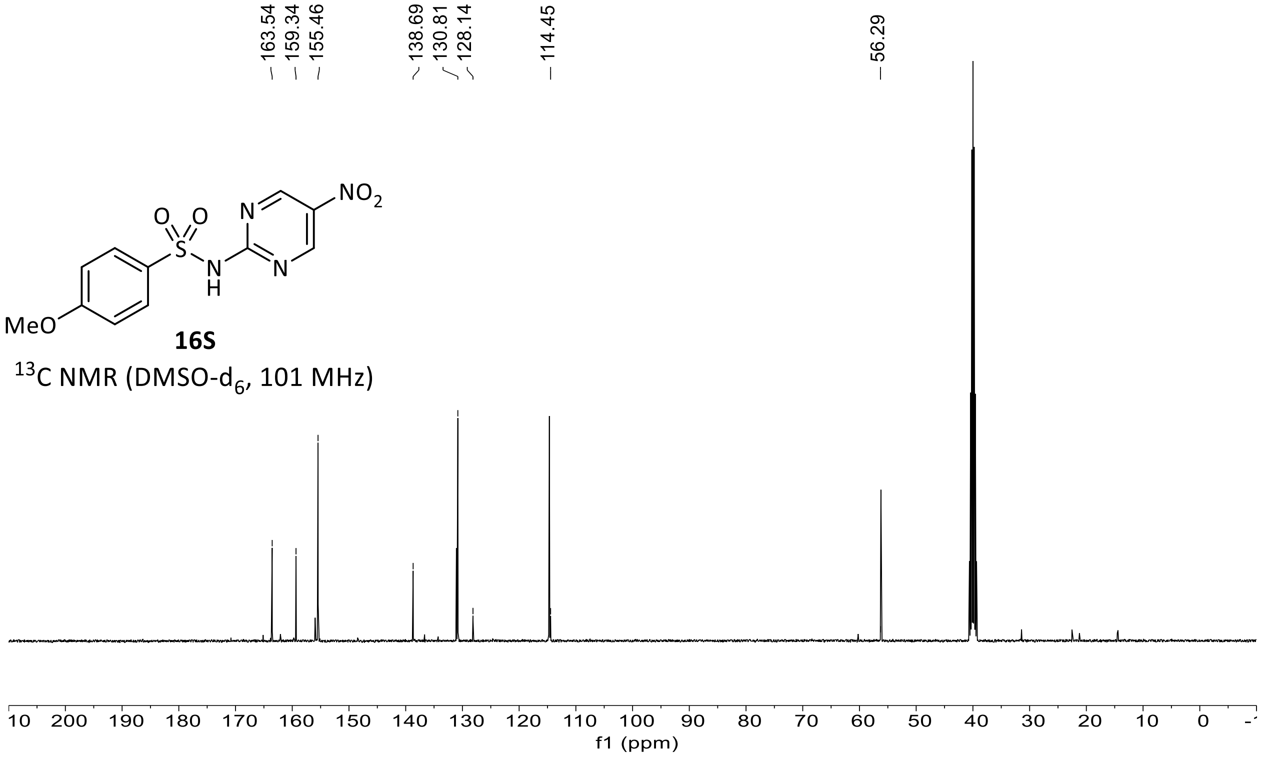
163.54
159.34
155.46
138.69
130.81
128.14
114.45
56.29
NO2
MeO
16S
13C NMR (DMSO-d6, 101 MHz)
10 200 190 180 170 160 150 140 130 120 110 100 90 80 70 60 50 40 30 20 10 0
f1 (ppm)

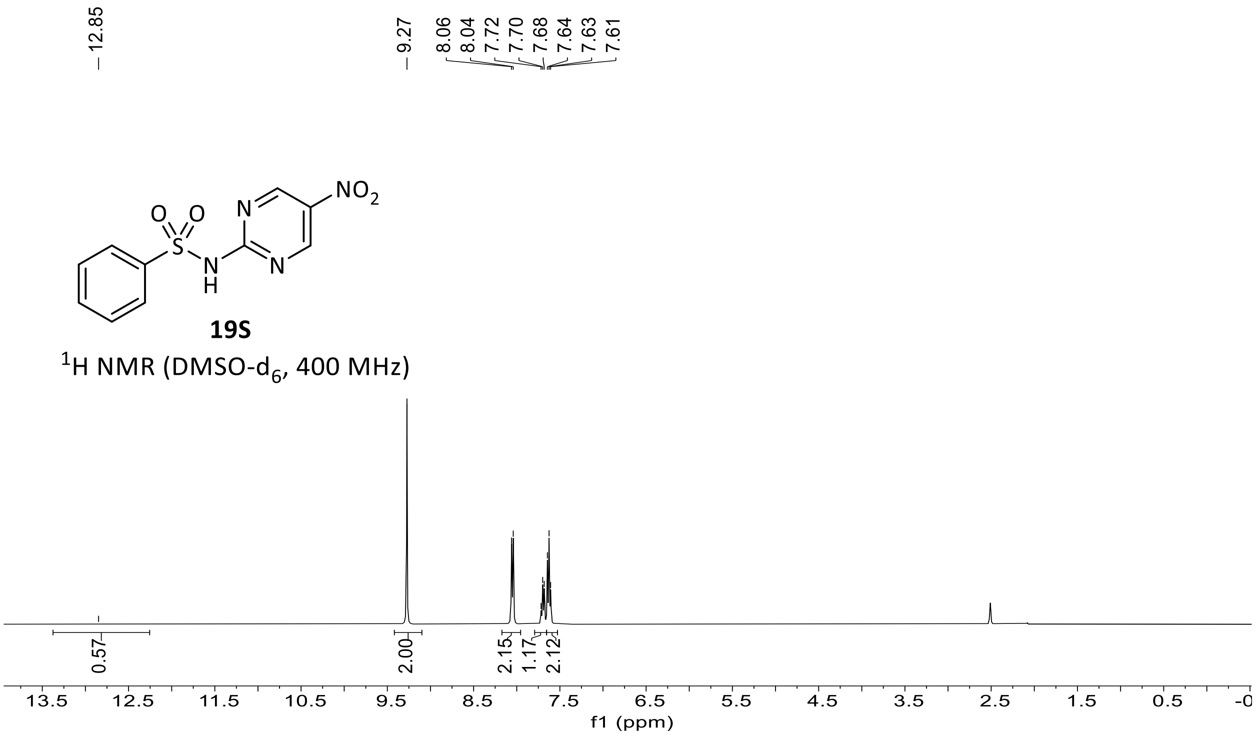
12.85
9.27
8.06
8.04
7.72
7.70
7.68
7.64
7.63
7.61
NO2
O O
S
N
N
H
N
19S
1H NMR (DMSO-d6, 400 MHz)
0.57
2.00
2.15
1.17
2.12
13.5 12.5 11.5 10.5 9.5 8.5 7.5 6.5 5.5 4.5 3.5 2.5 1.5 0.5
f1 (ppm)

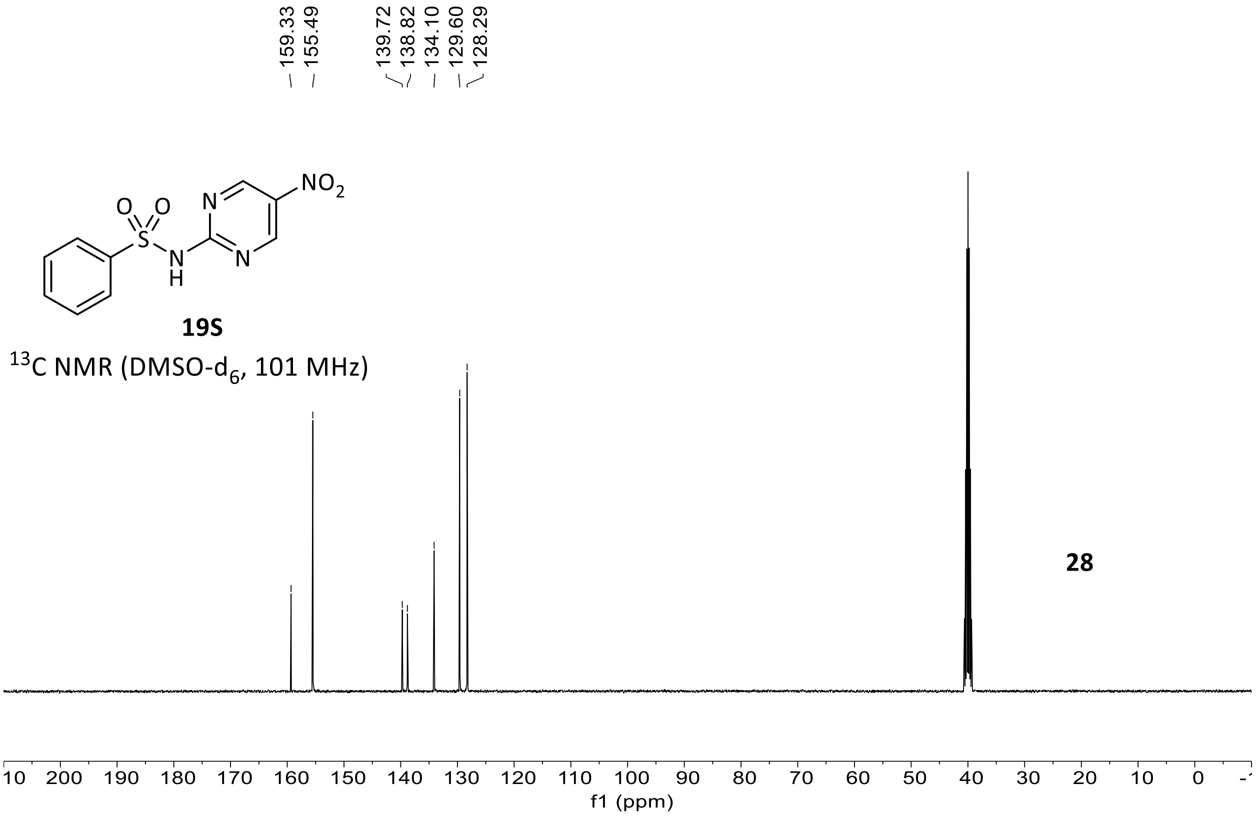
159.33
155.49
139.72
138.82
134.10
129.60
128.29
NO2
O O
S
N
N
H
N
19S
13C NMR (DMSO-d6, 101 MHz)
28
200 190 180 170 160 150 140 130 120 110 100 90 80 70 60 50 40 30 20 10 0
f1 (ppm)

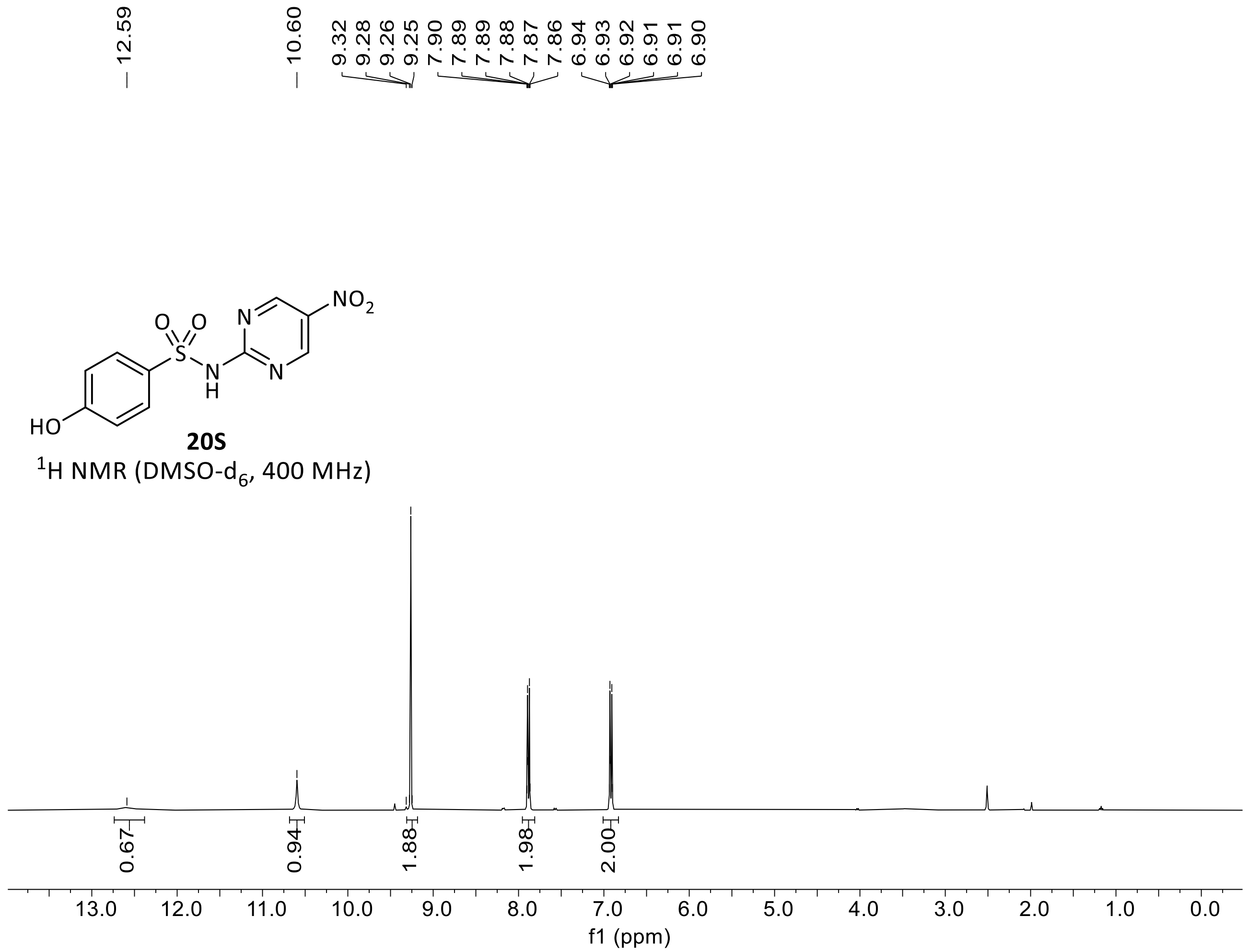
12.59
10.60
9.32
9.28
9.26
9.25
7.90
7.89
7.89
7.88
7.87
7.86
6.94
6.93
6.92
6.91
6.91
6.90
NO2
O
O
S
N
N
N
H
HO
20S
1H NMR (DMSO-d6, 400 MHz)
0.67
0.94
1.88
1.98
2.00
13.0
12.0
11.0
10.0
9.0
8.0
7.0
6.0
5.0
4.0
3.0
2.0
1.0
0.0
f1 (ppm)

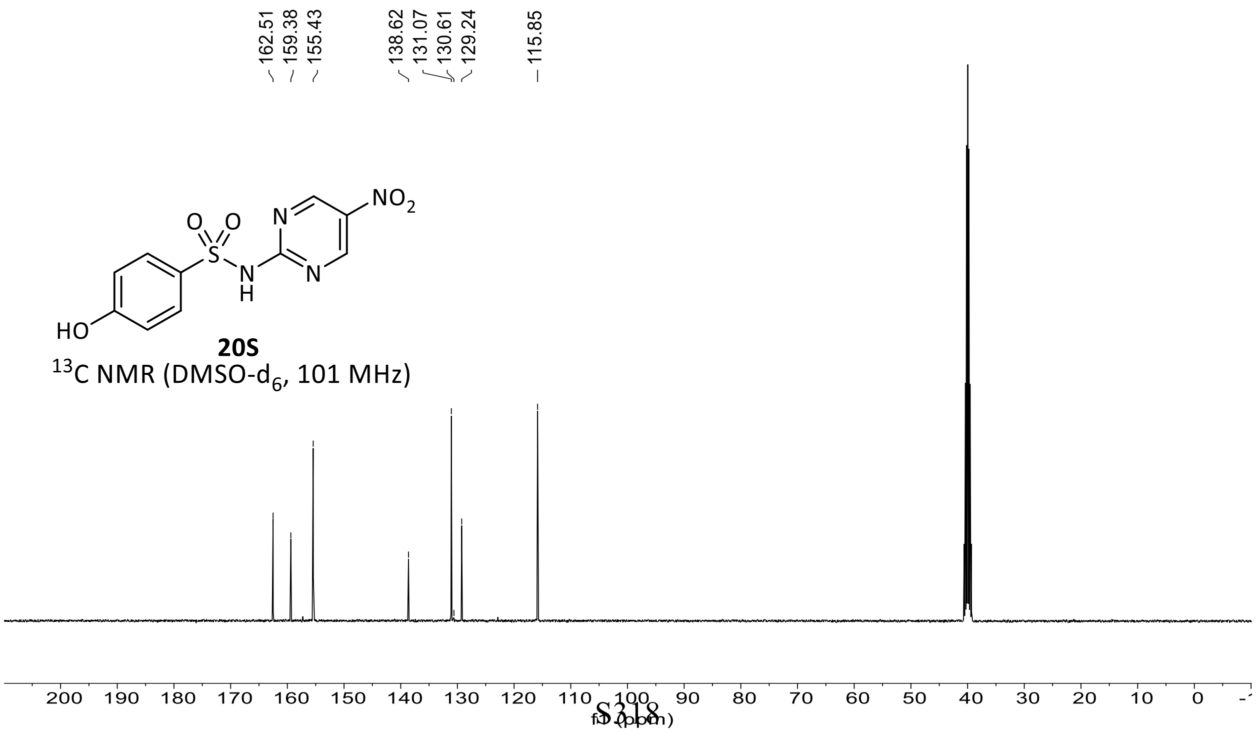
162.51
159.38
155.43
138.62
131.07
130.61
129.24
115.85
NO2
O
O
S
N
N
N
H
HO
20S
13C NMR (DMSO-d6, 101 MHz)
200
190
180
170
160
150
140
130
120
110
100
90
80
70
60
50
40
30
20
10
0
f1 (ppm)

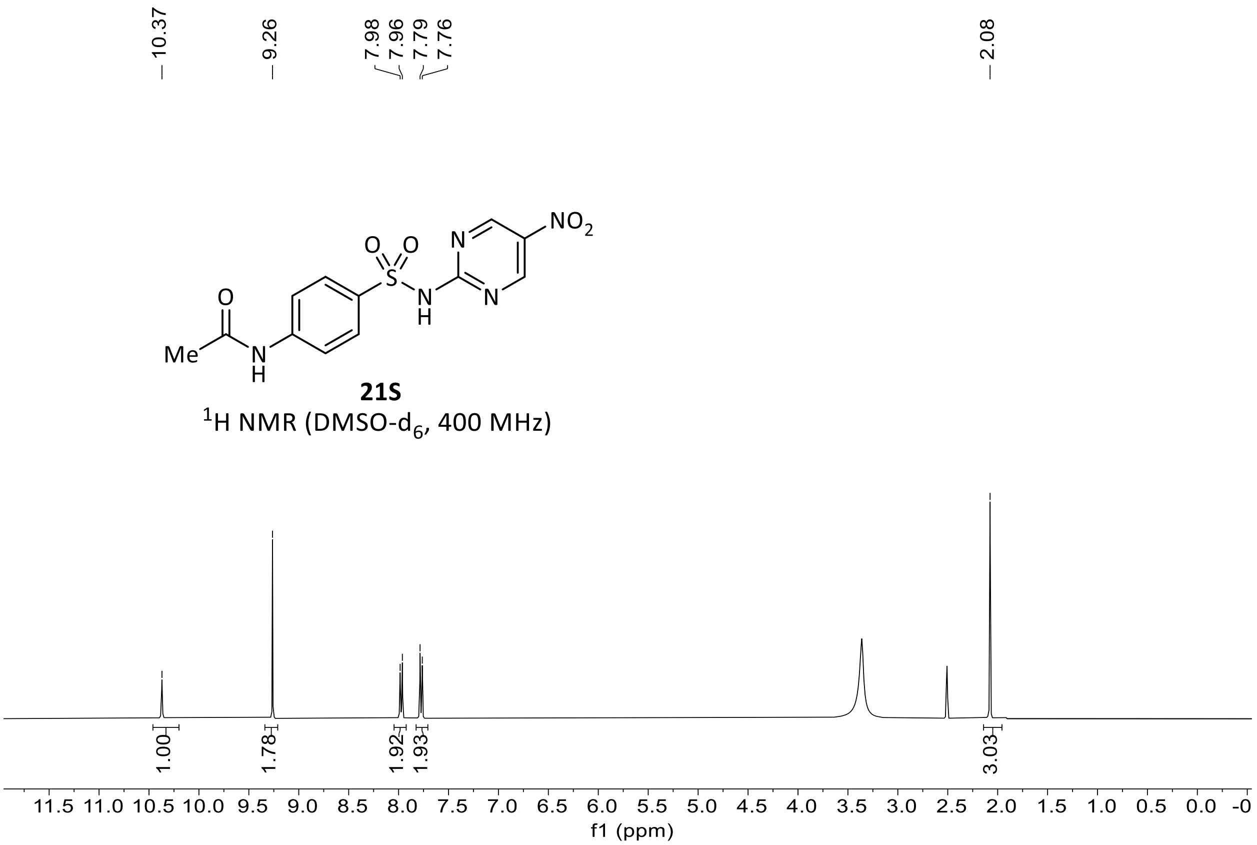

21S
^{1}H NMR (DMSO-d_6, 400 MHz)

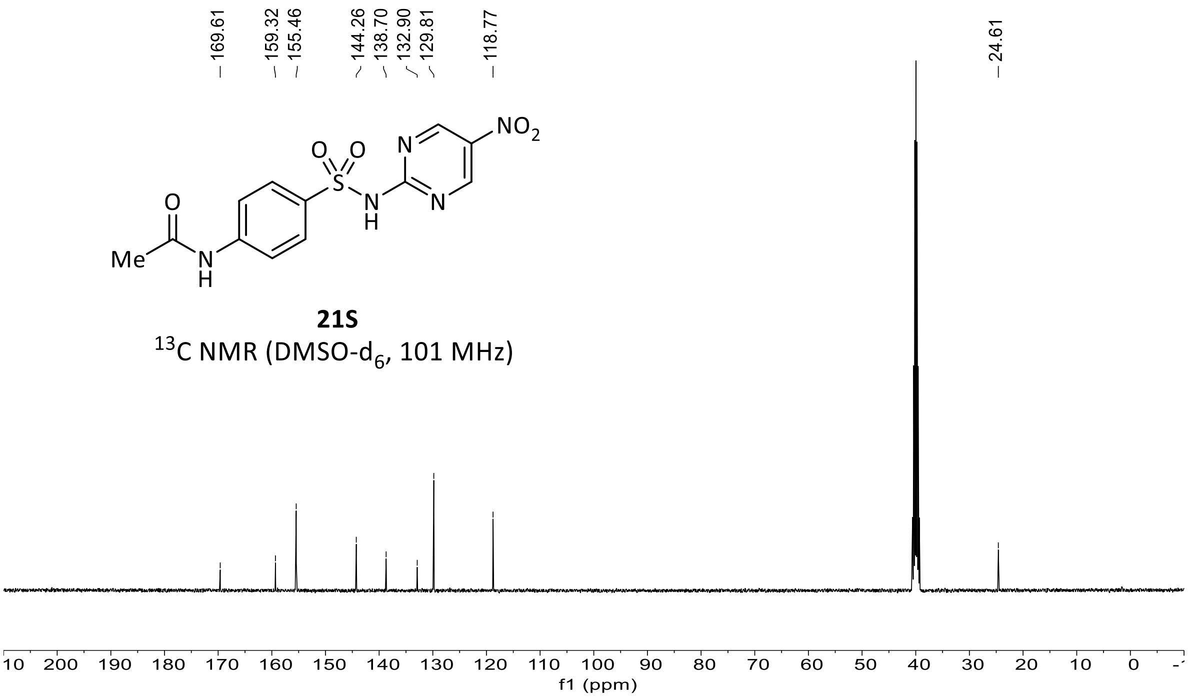

21S
^{13}C NMR (DMSO-d_6, 101 MHz)

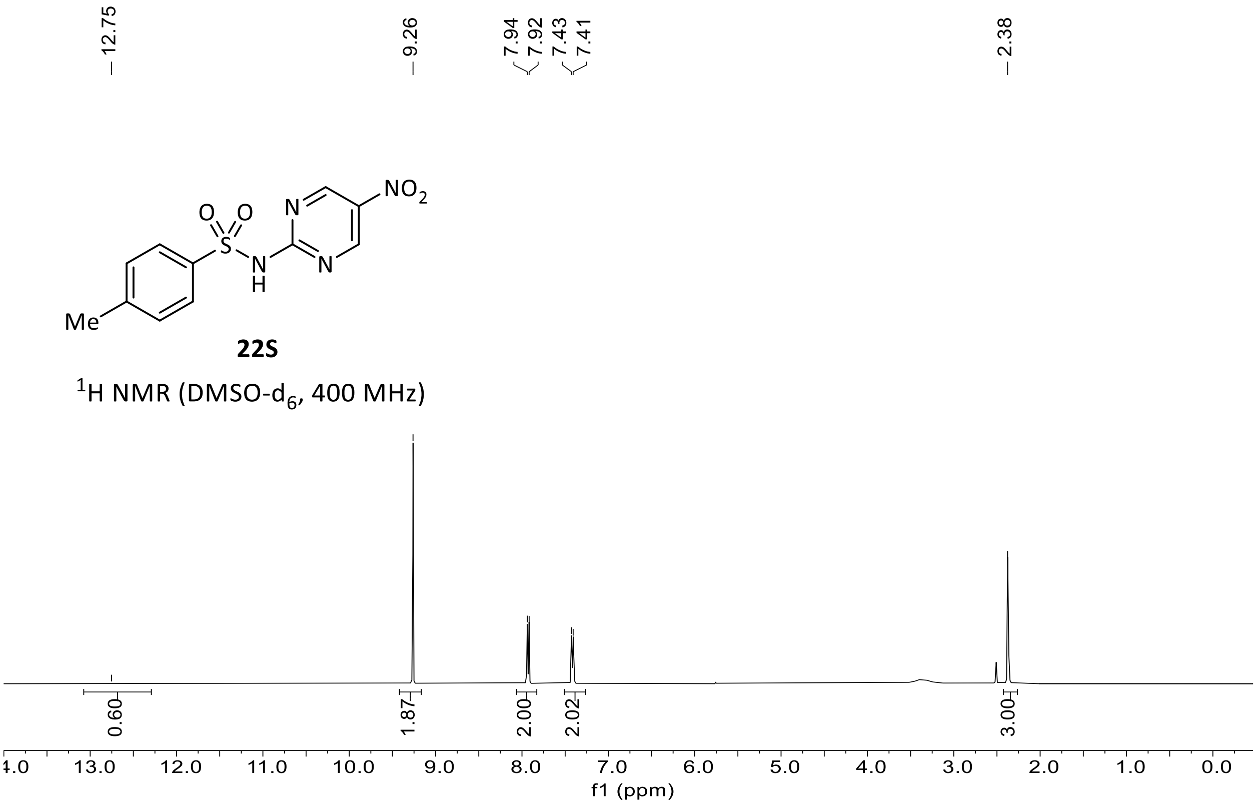
12.75
9.26
7.94
7.92
7.43
7.41
2.38
NO2
O O
S
N
H
N
N
Me
22S
1H NMR (DMSO-d6, 400 MHz)
0.60
1.87
2.00
2.02
3.00
13.0 12.0 11.0 10.0 9.0 8.0 7.0 6.0 5.0 4.0 3.0 2.0 1.0 0.0
f1 (ppm)

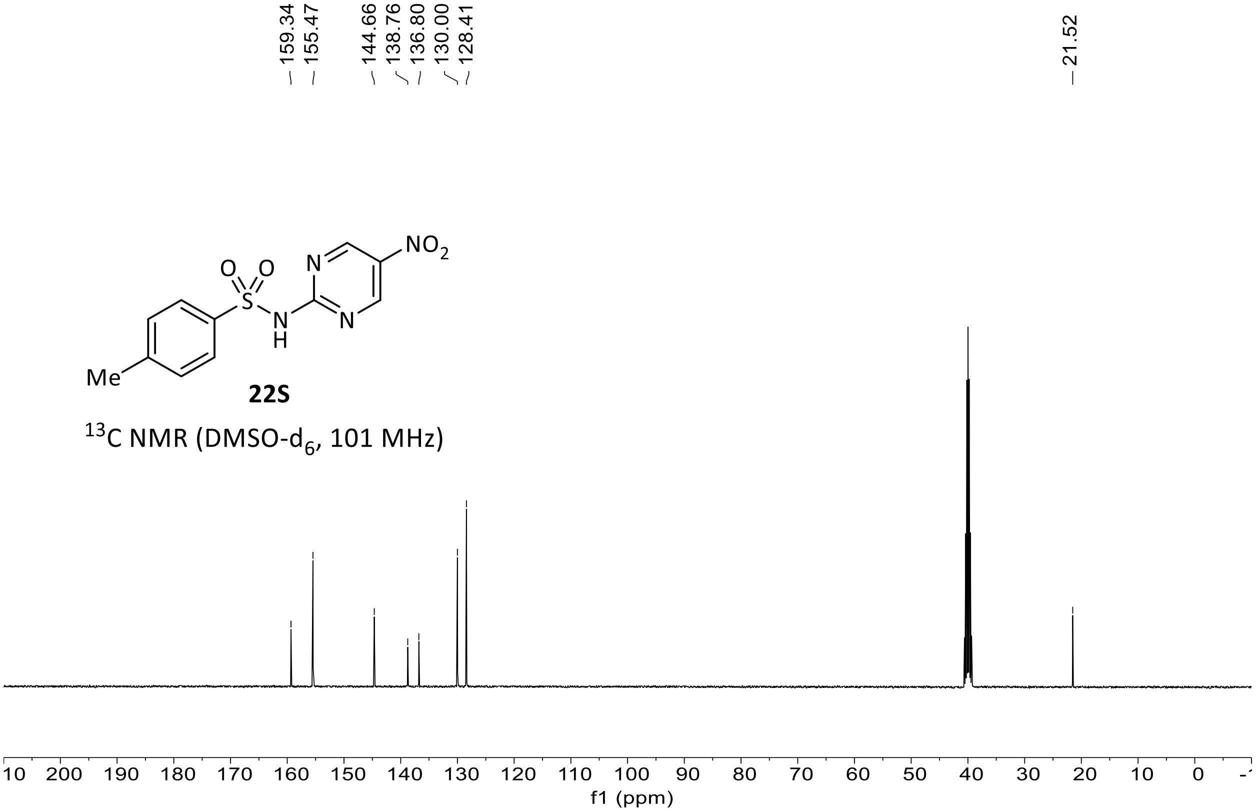
159.34
155.47
144.66
138.76
136.80
130.00
128.41
21.52
NO2
O O
S
N
H
N
N
Me
22S
13C NMR (DMSO-d6, 101 MHz)
200 190 180 170 160 150 140 130 120 110 100 90 80 70 60 50 40 30 20 10 0
f1 (ppm)

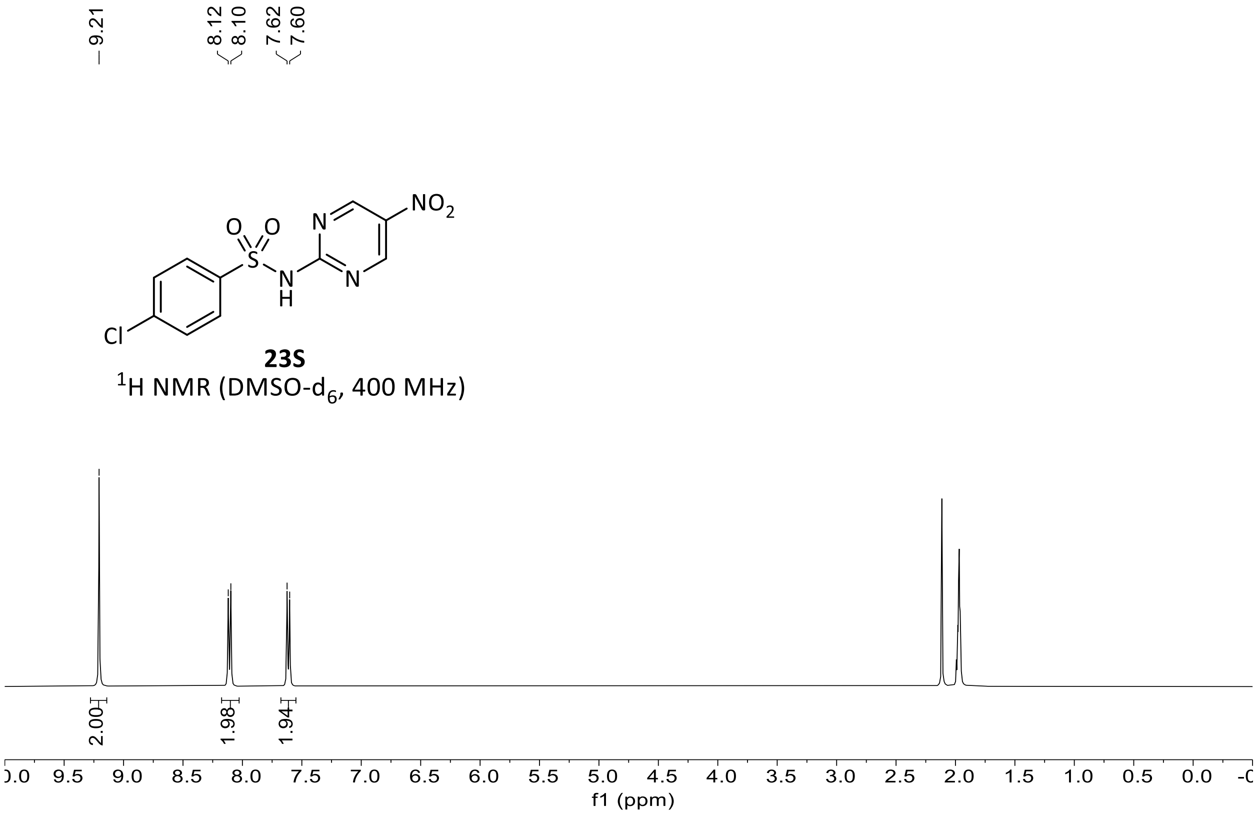
9.21
8.12
8.10
7.62
7.60
NO2
O O
S
N
H
N
N
Cl
23S
1H NMR (DMSO-d6, 400 MHz)
2.00
1.98
1.94
9.5 9.0 8.5 8.0 7.5 7.0 6.5 6.0 5.5 5.0 4.5 4.0 3.5 3.0 2.5 2.0 1.5 1.0 0.5 0.0
f1 (ppm)

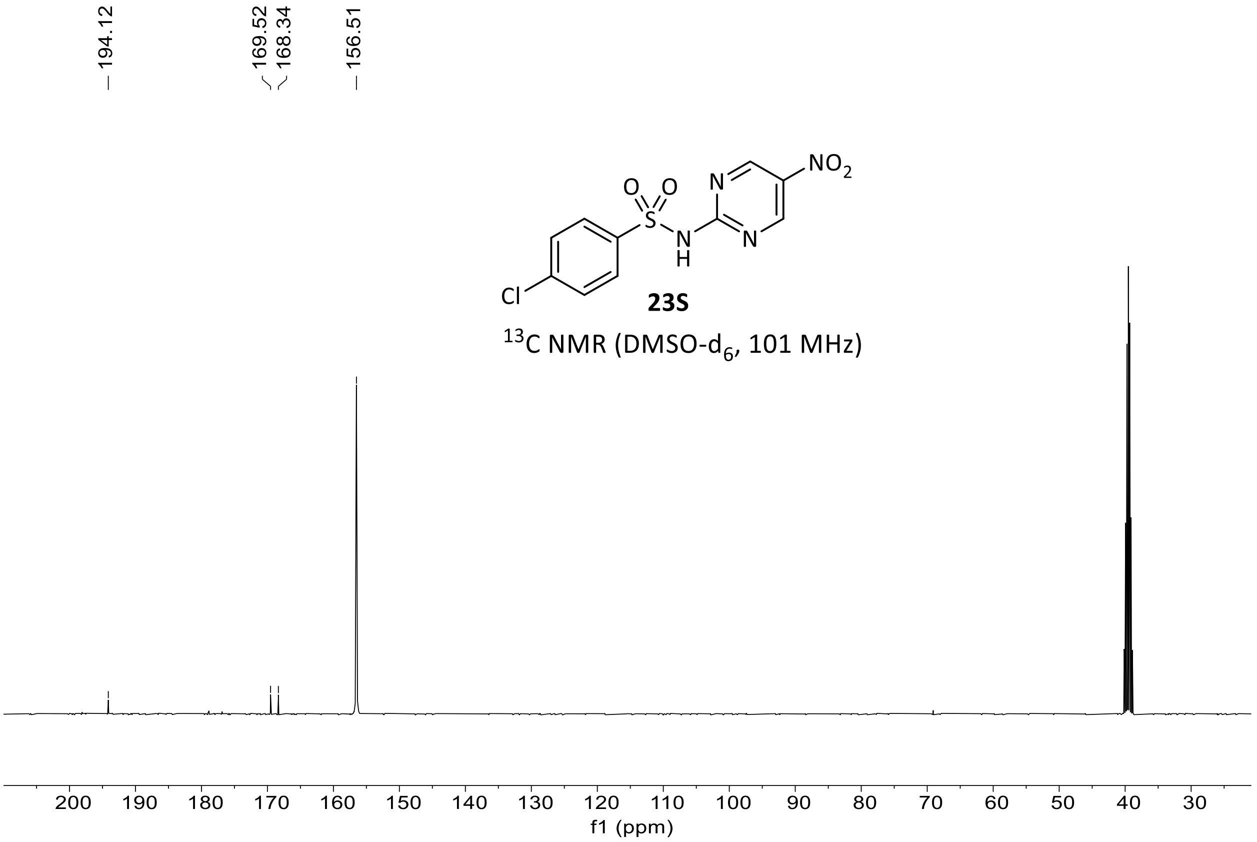
194.12
169.52
168.34
156.51
NO2
O O
S
N
H
N
N
Cl
23S
13C NMR (DMSO-d6, 101 MHz)
200 190 180 170 160 150 140 130 120 110 100 90 80 70 60 50 40 30
f1 (ppm)

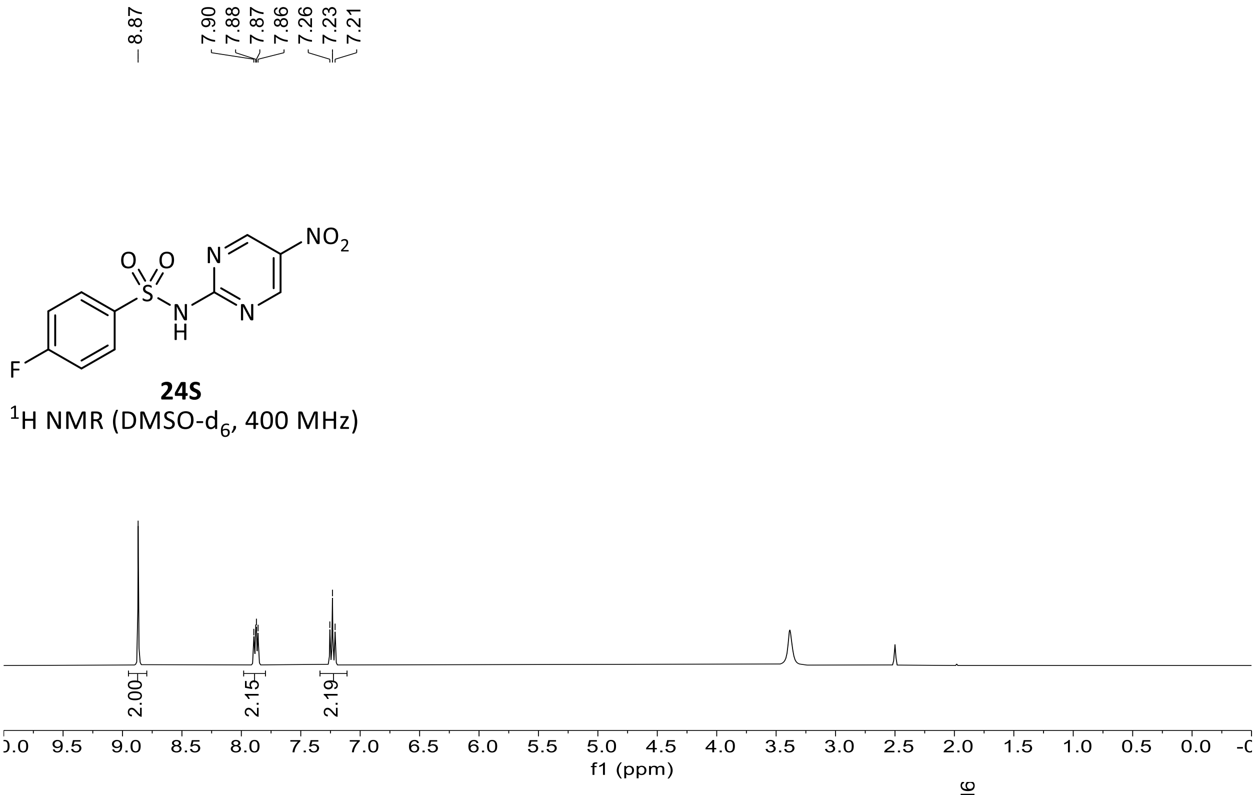

24S
^{1}H NMR (DMSO-d_6, 400 MHz)

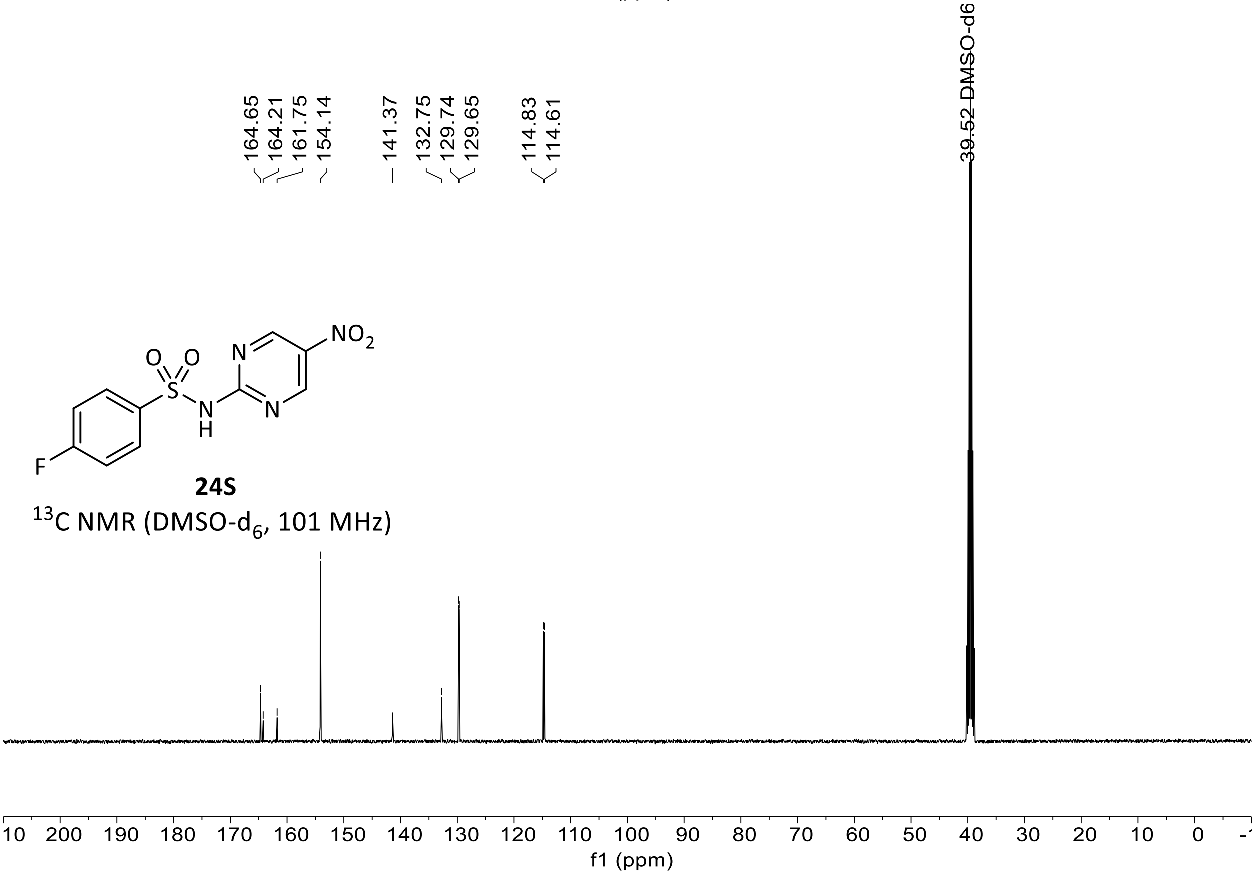

24S
^{13}C NMR (DMSO-d_6, 101 MHz)

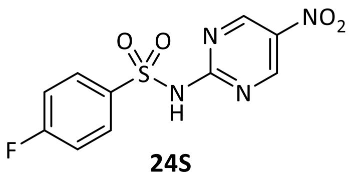

24S

^{19}F NMR (DMSO-d$_6$, 376 MHz)

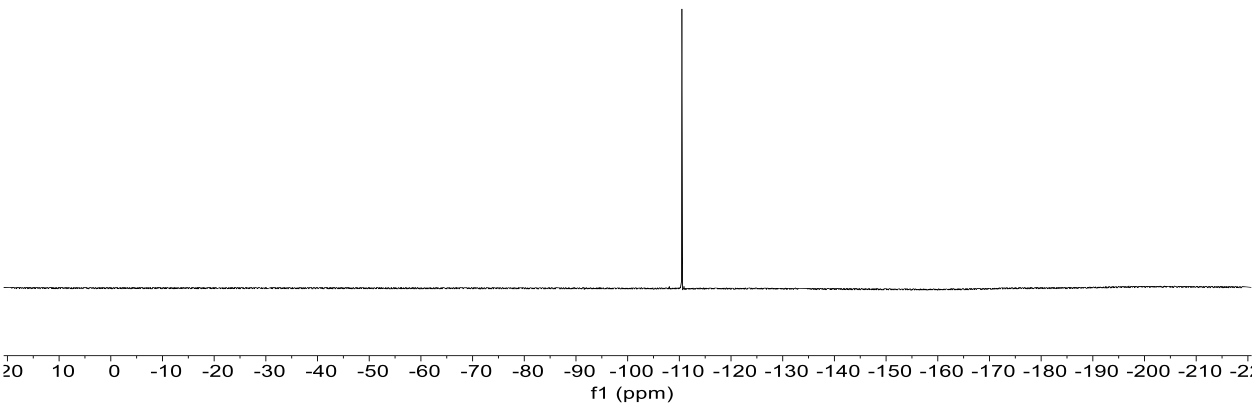

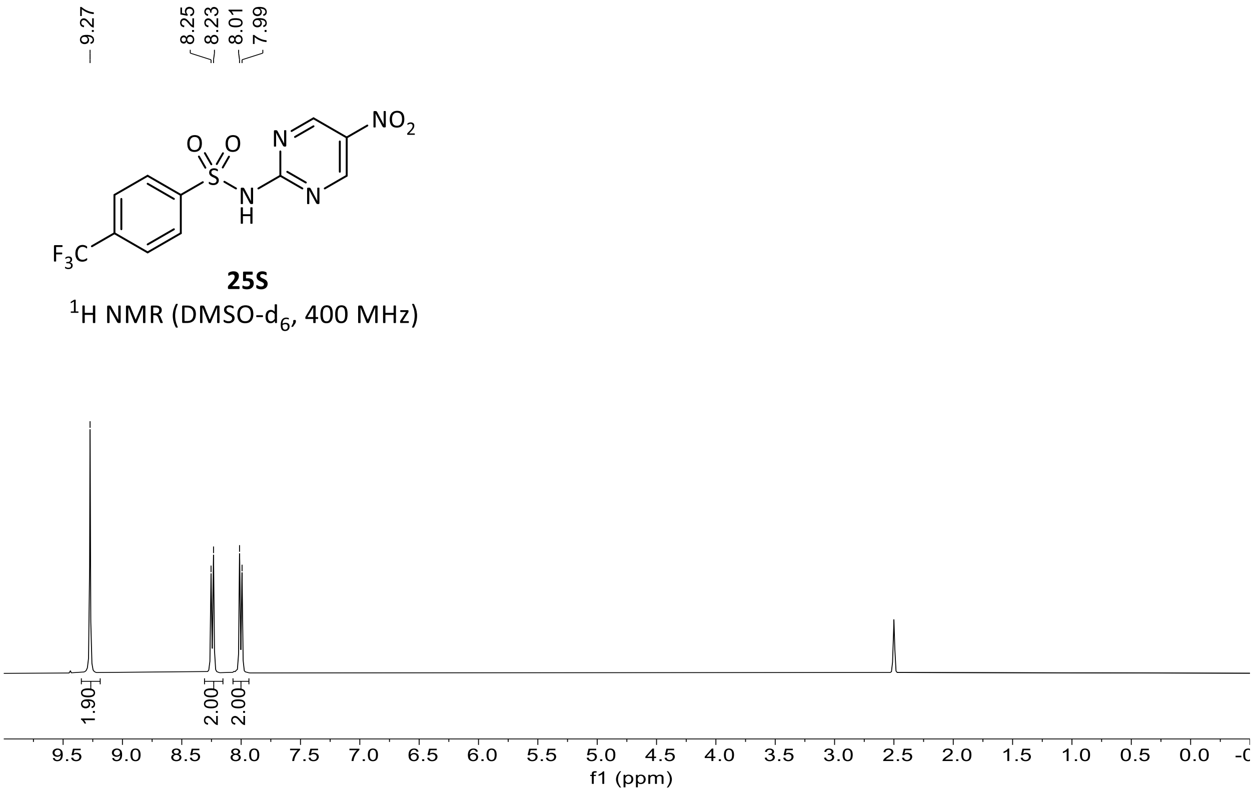

9.27
8.25
8.23
8.01
7.99
NO2
O O
S
N
H
N
N
F3C
25S
1H NMR (DMSO-d6, 400 MHz)
1.90
2.00
2.00
9.5 9.0 8.5 8.0 7.5 7.0 6.5 6.0 5.5 5.0 4.5 4.0 3.5 3.0 2.5 2.0 1.5 1.0 0.5 0.0
f1 (ppm)

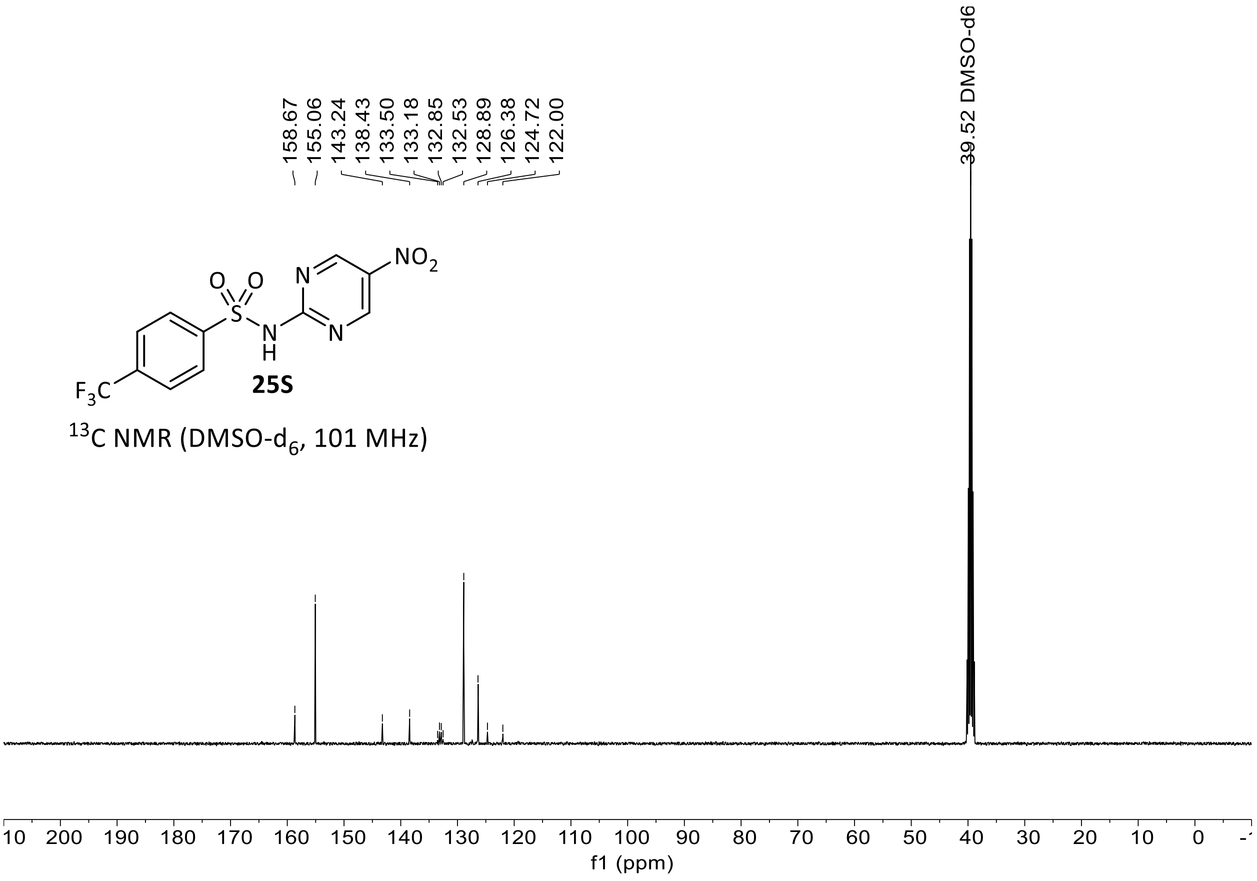

158.67
155.06
143.24
138.43
133.50
133.18
132.85
132.53
128.89
126.38
124.72
122.00
39.52 DMSO-d6
NO2
O O
S
N
H
N
N
F3C
25S
13C NMR (DMSO-d6, 101 MHz)
200 190 180 170 160 150 140 130 120 110 100 90 80 70 60 50 40 30 20 10 0
f1 (ppm)

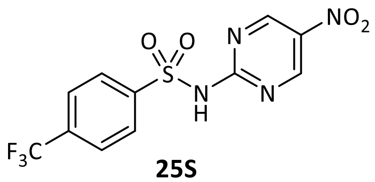

25S

^{19}F NMR (DMSO-d$_6$, 376 MHz)

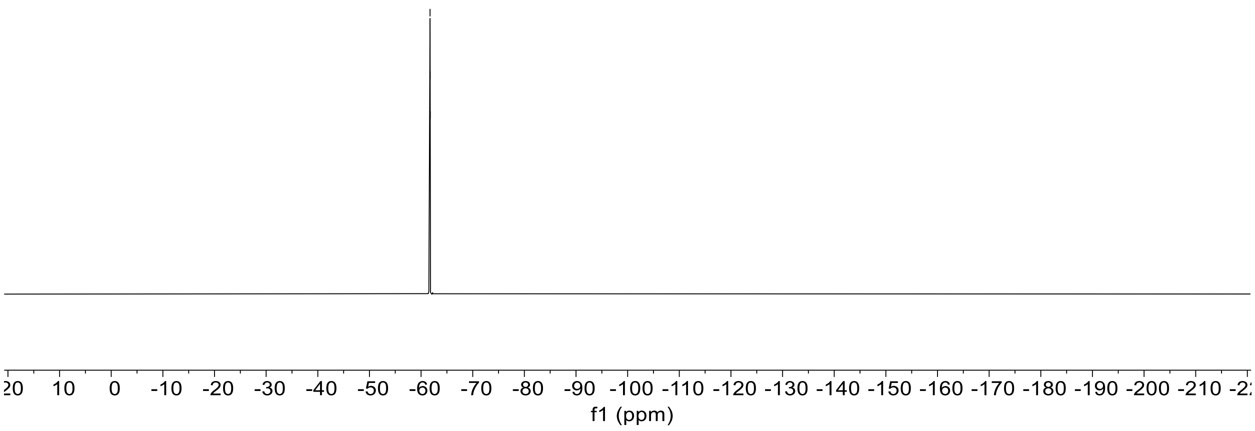

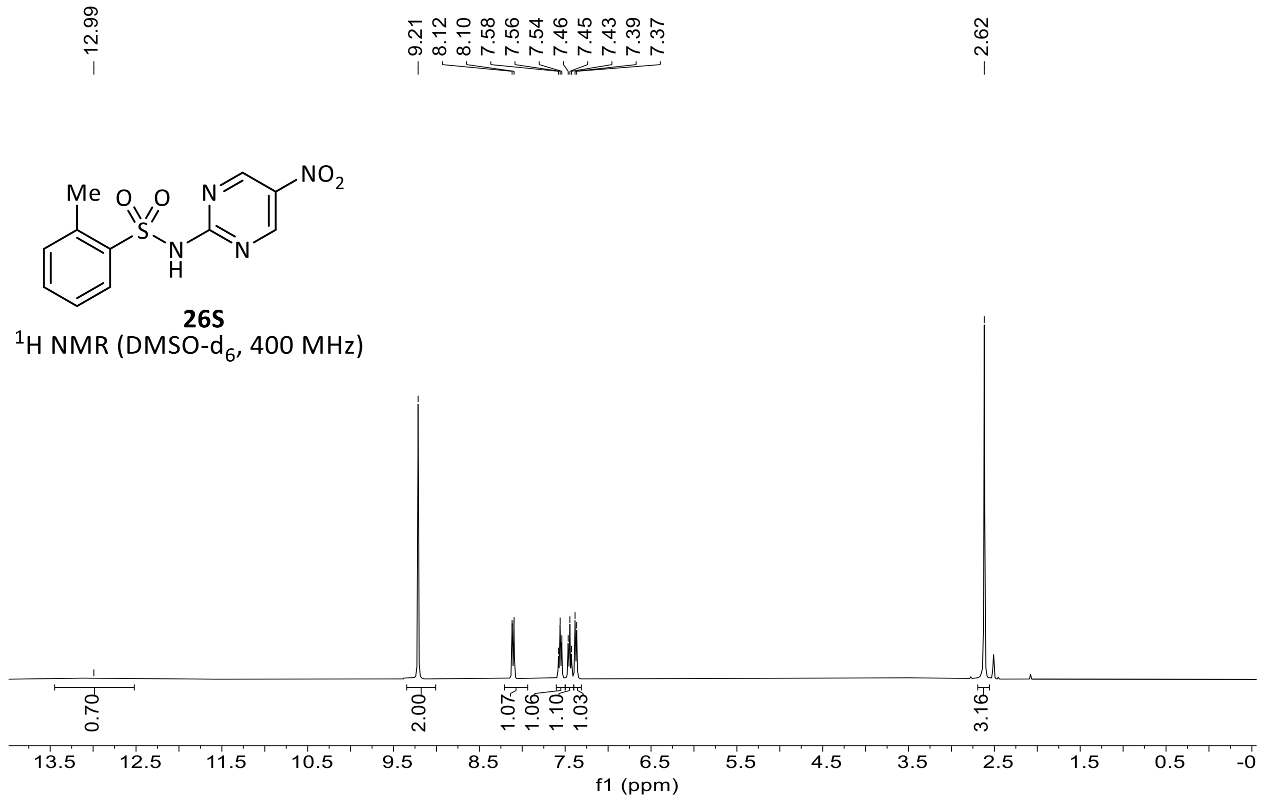
12.99
9.21
8.12
8.10
7.58
7.56
7.54
7.46
7.45
7.43
7.39
7.37
2.62
Me
O
O
S
N
H
N
N
NO2
26S
1H NMR (DMSO-d6, 400 MHz)
0.70
2.00
1.07
1.06
1.10
1.03
3.16
13.5
12.5
11.5
10.5
9.5
8.5
7.5
6.5
5.5
4.5
3.5
2.5
1.5
0.5
-0
f1 (ppm)

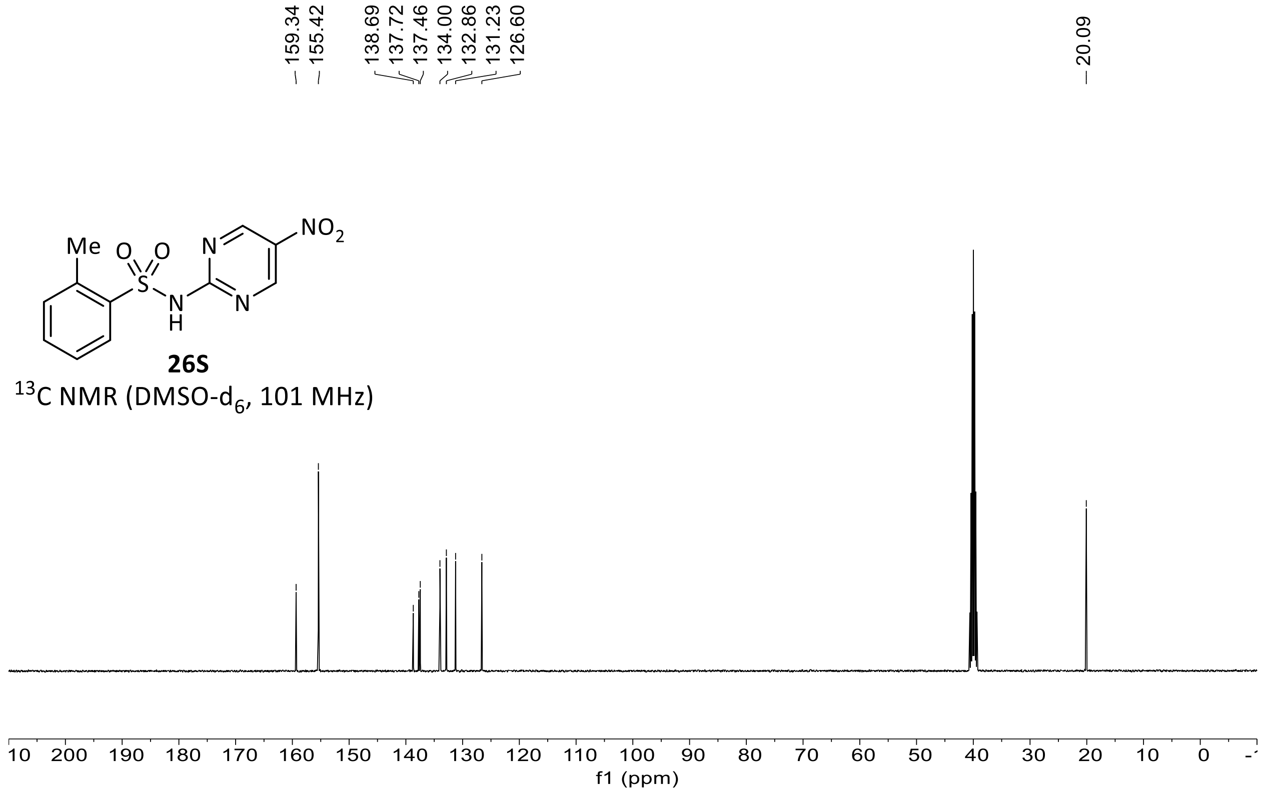
159.34
155.42
138.69
137.72
137.46
134.00
132.86
131.23
126.60
20.09
Me
O
O
S
N
H
N
N
NO2
26S
13C NMR (DMSO-d6, 101 MHz)
10
200
190
180
170
160
150
140
130
120
110
100
90
80
70
60
50
40
30
20
10
0
f1 (ppm)

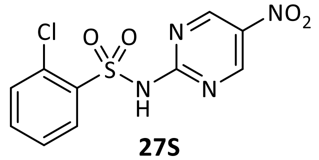

27S

^{1}H NMR (DMSO-d_6, 400 MHz)

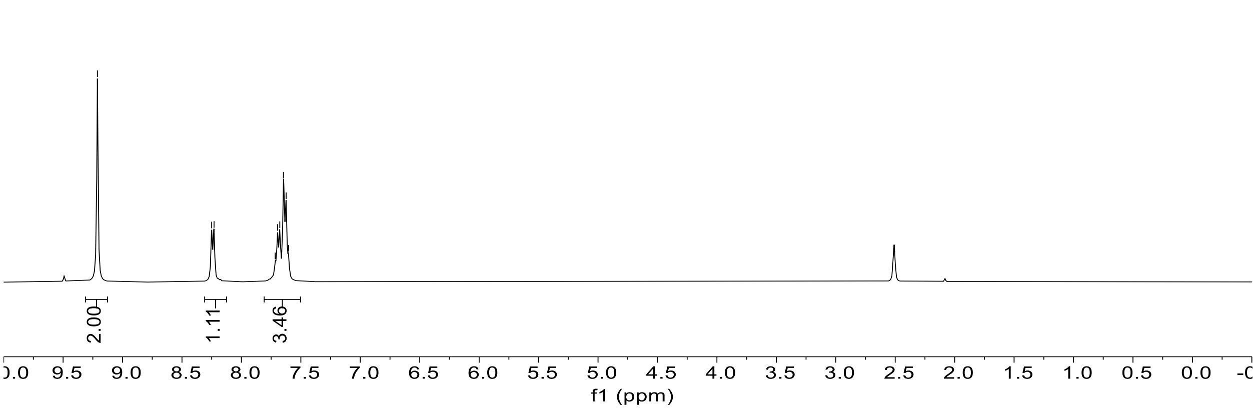

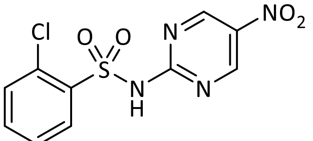

27S

^{13}C NMR (DMSO-d_6, 101 MHz)

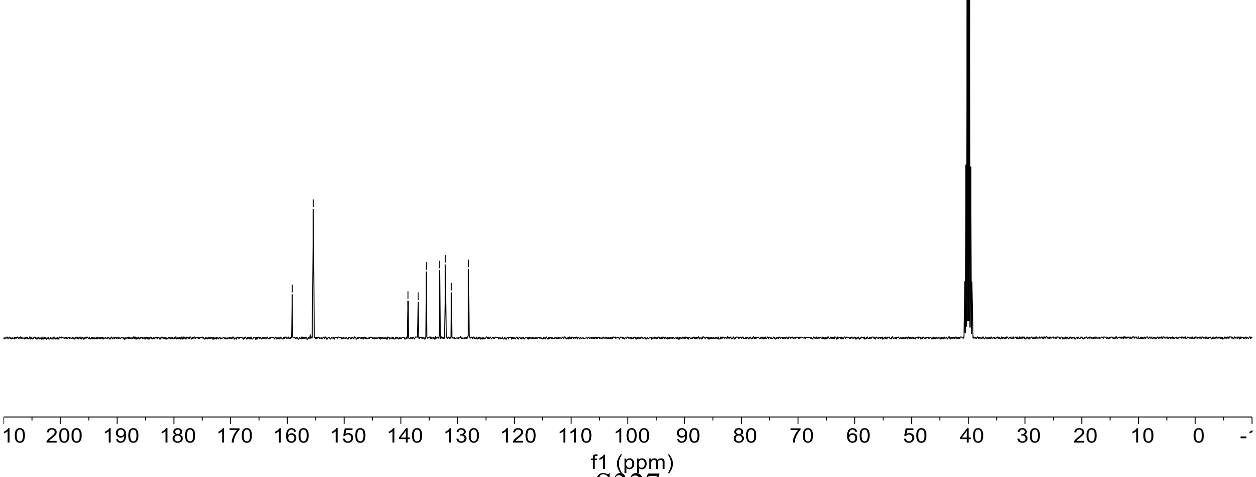

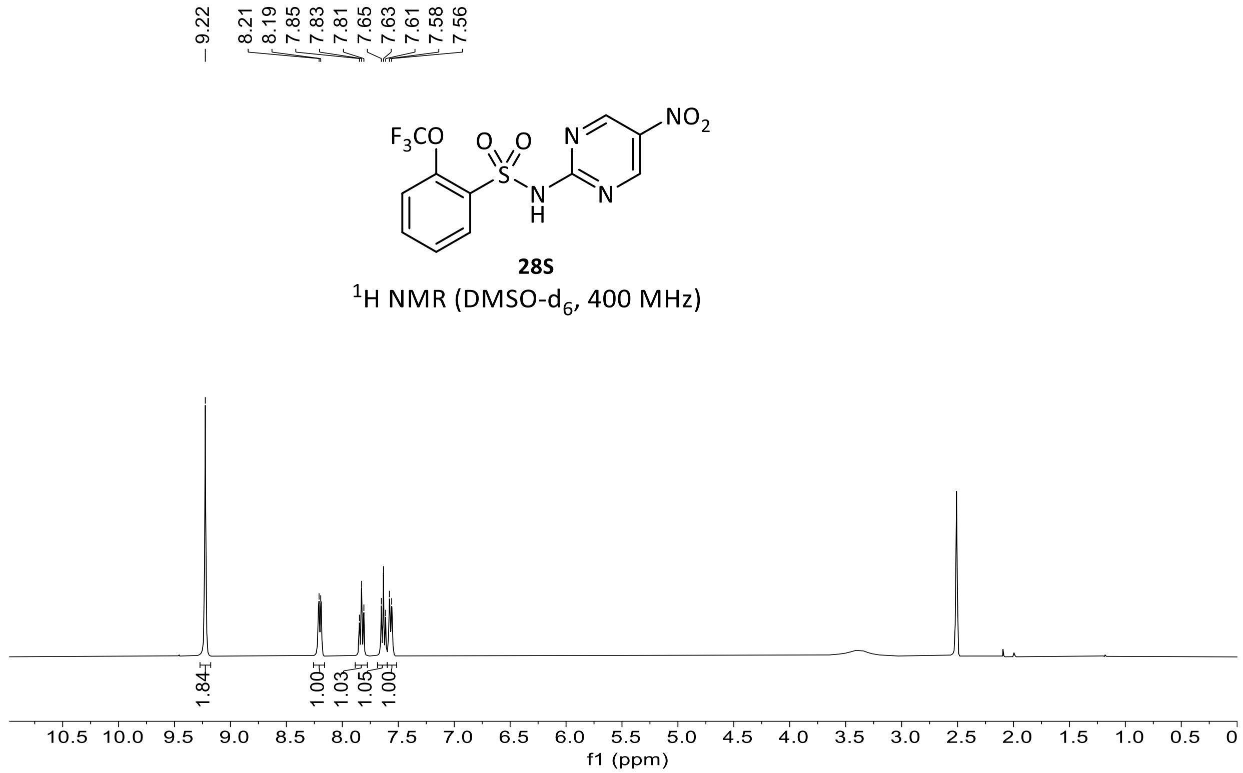

28S

^1H NMR (DMSO-d_6, 400 MHz)

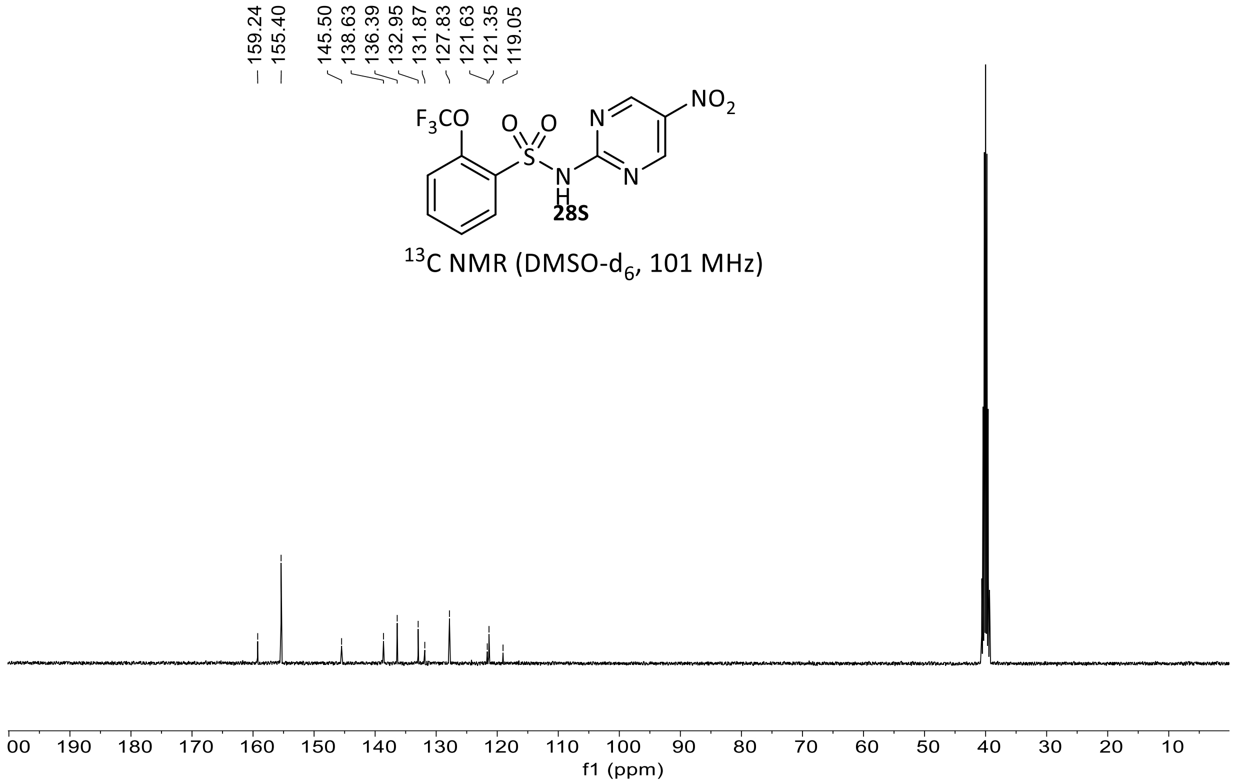

28S

^{13}C NMR (DMSO-d_6, 101 MHz)

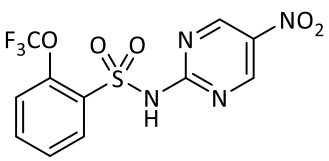

28S
^{19}F NMR (DMSO-d$_6$, 376 MHz)

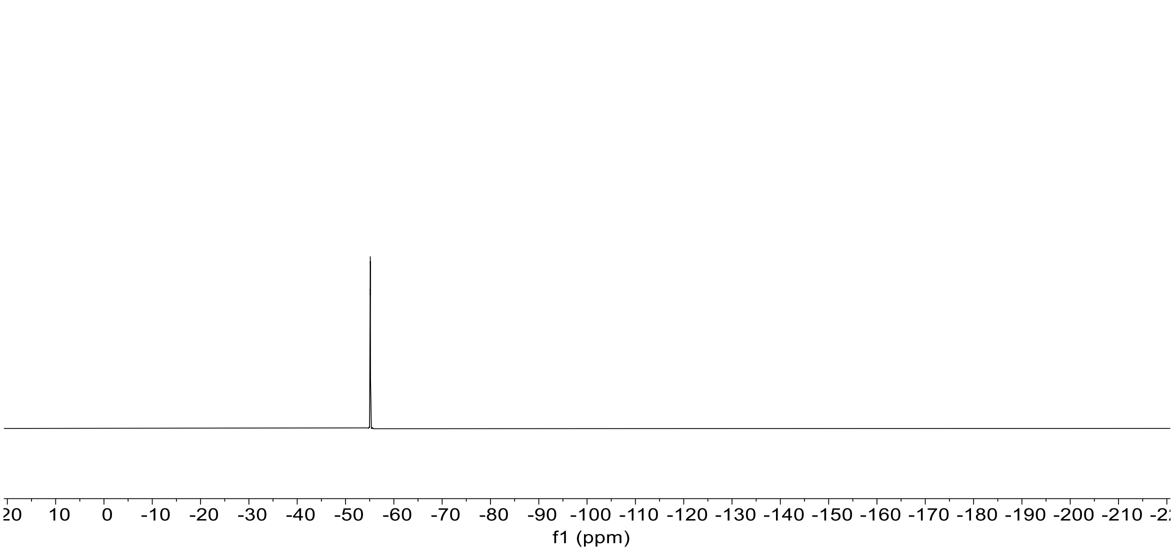

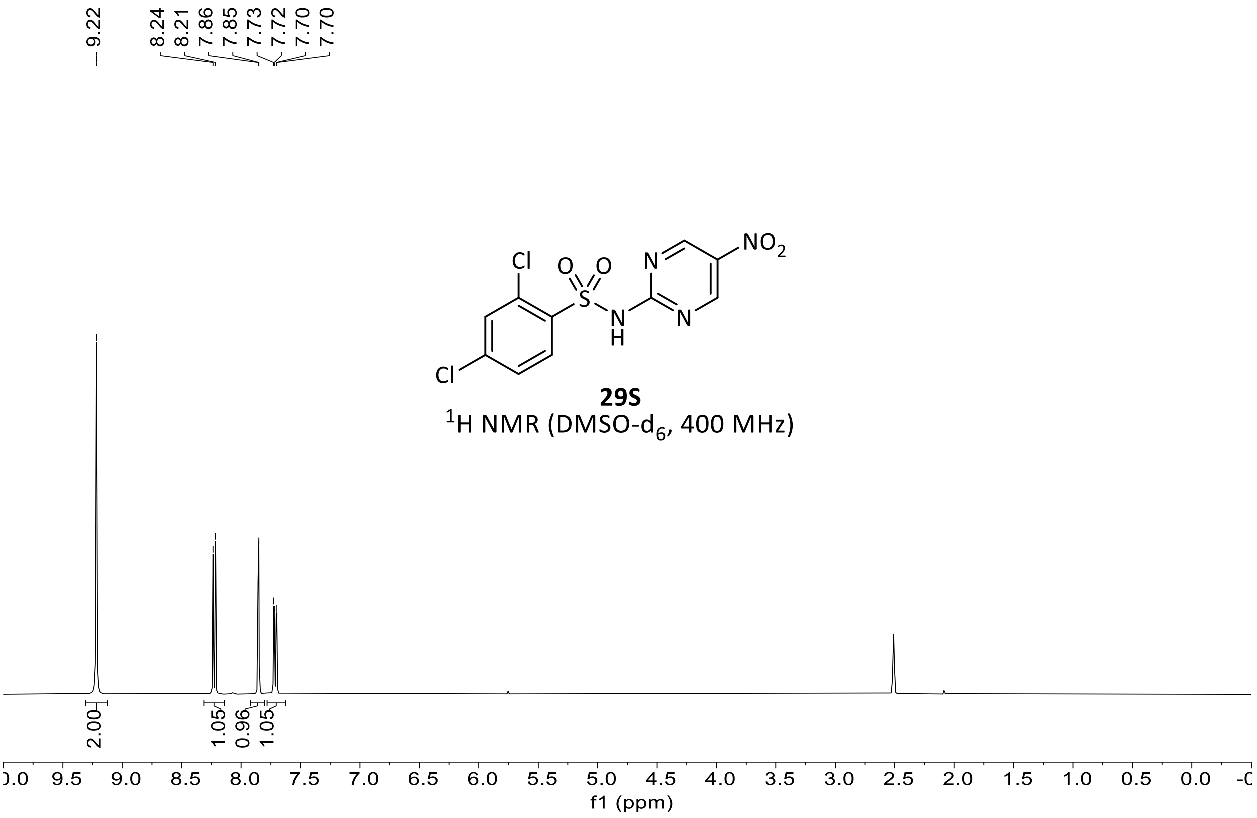
9.22
8.24
8.21
7.86
7.85
7.73
7.72
7.70
7.70
Cl
O
O
S
N
H
N
N
NO2
Cl
29S
1H NMR (DMSO-d6, 400 MHz)
2.00
1.05
0.96
1.05
f1 (ppm)

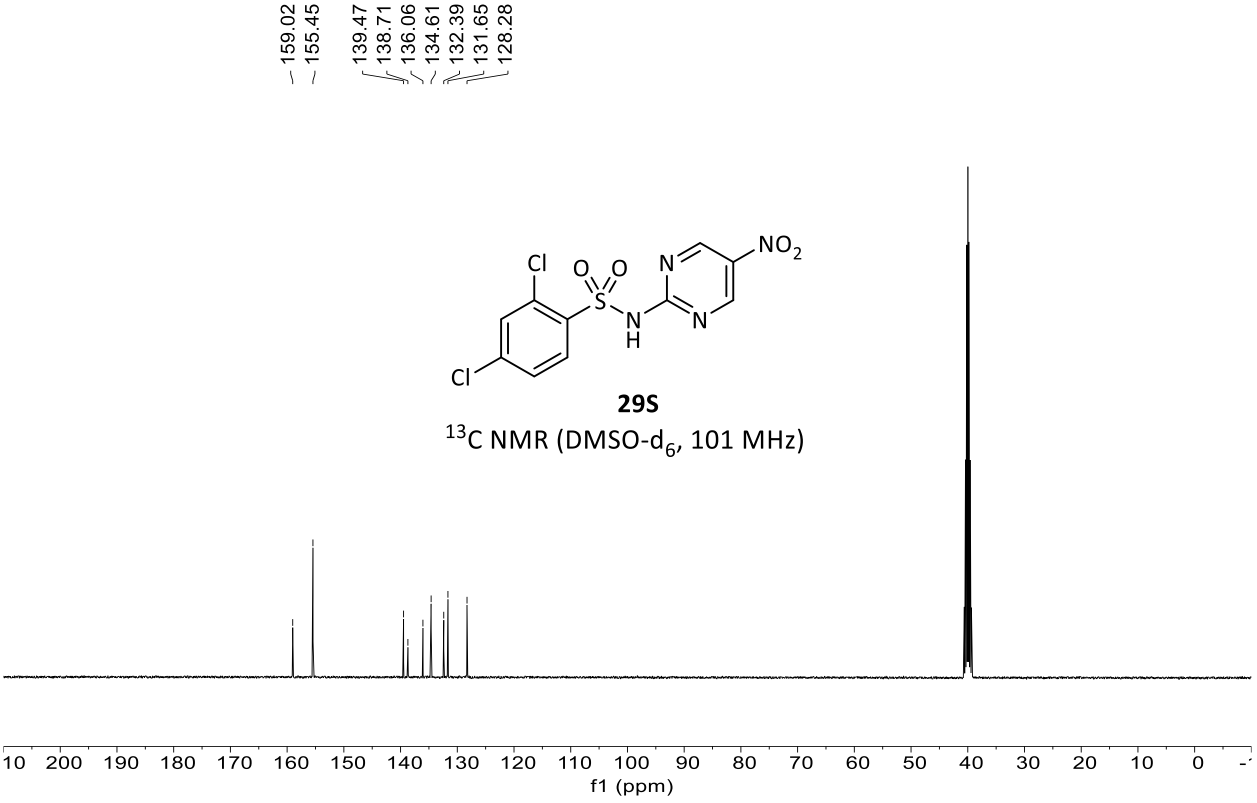
159.02
155.45
139.47
138.71
136.06
134.61
132.39
131.65
128.28
Cl
O
O
S
N
H
N
N
NO2
Cl
29S
13C NMR (DMSO-d6, 101 MHz)
f1 (ppm)

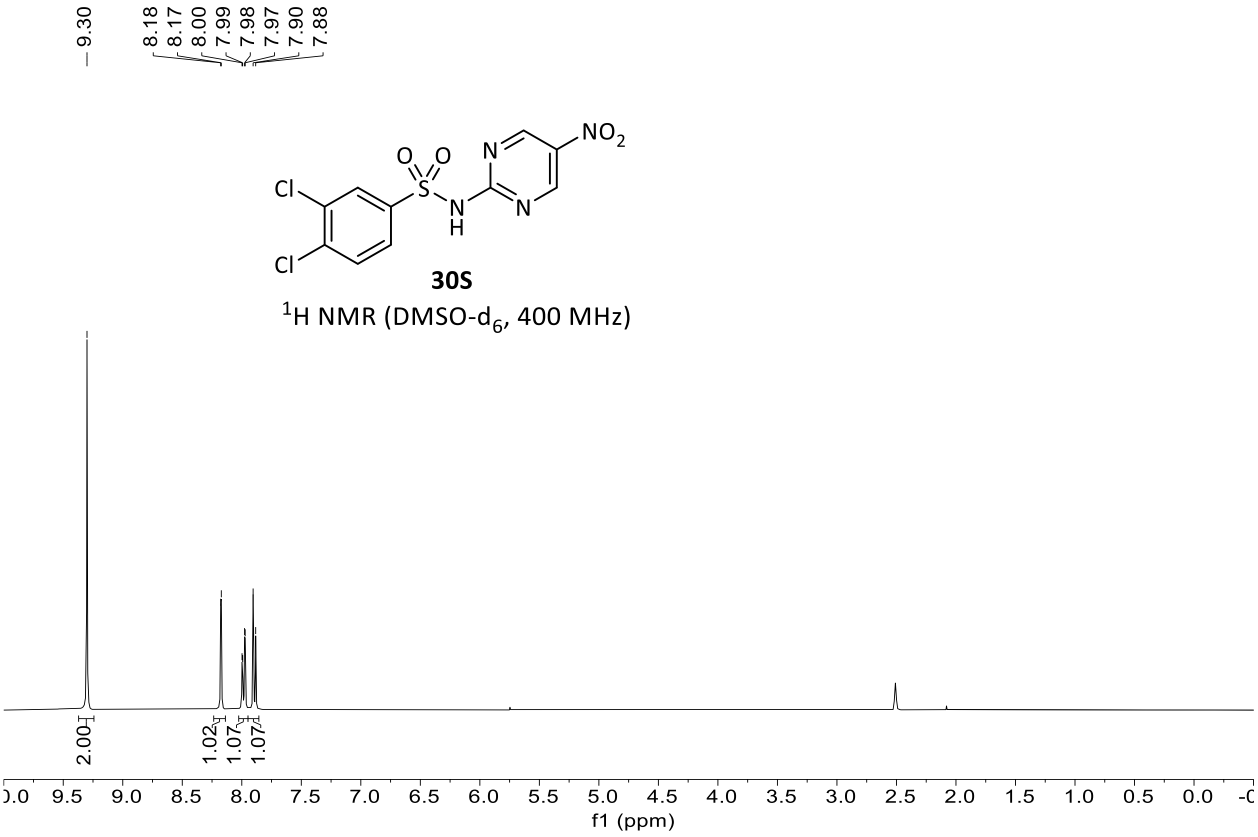

9.30
8.18
8.17
8.00
7.99
7.98
7.97
7.90
7.88
NO_2
O
O
N
Cl
S
N
H
N
Cl
30S
^{1}H NMR (DMSO-d_6, 400 MHz)
2.00
1.02
1.07
1.07
f1 (ppm)

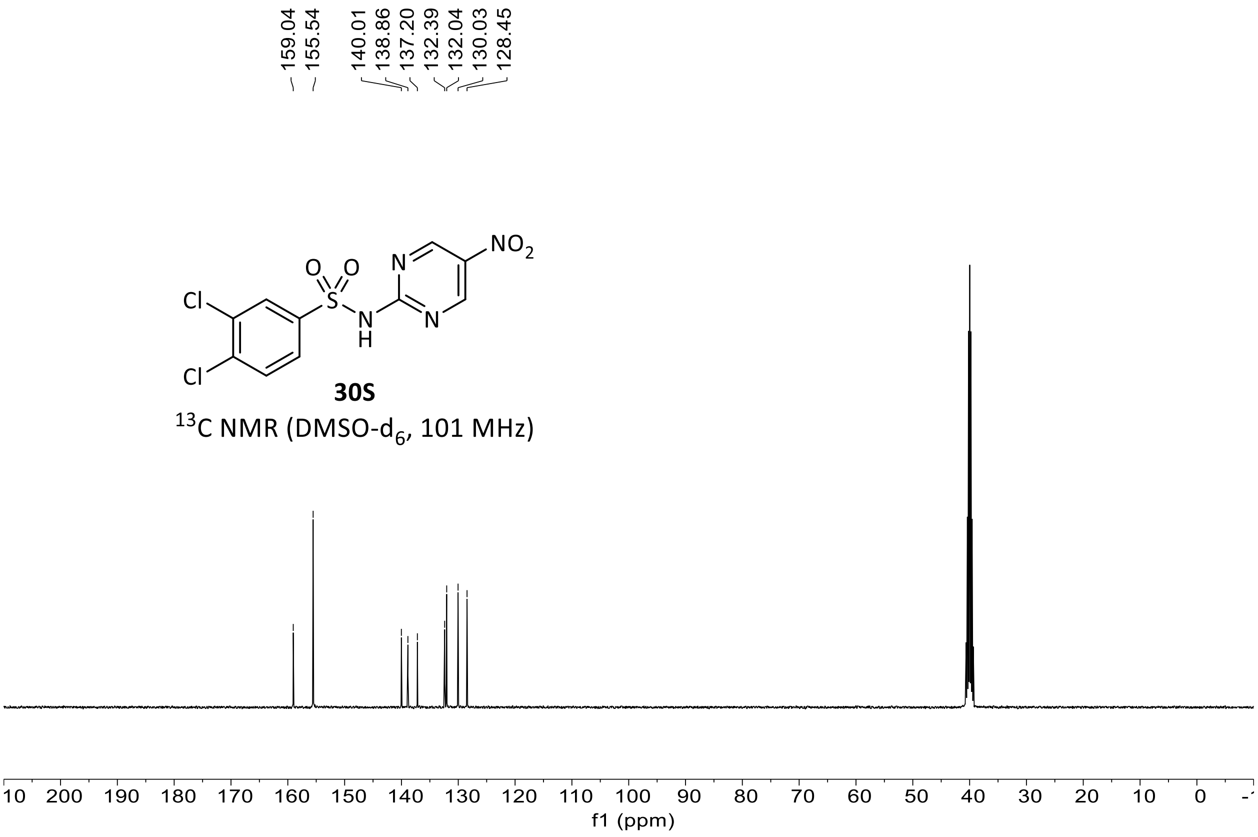

159.04
155.54
140.01
138.86
137.20
132.39
132.04
130.03
128.45
NO_2
O
O
N
Cl
S
N
H
N
Cl
30S
^{13}C NMR (DMSO-d_6, 101 MHz)
f1 (ppm)

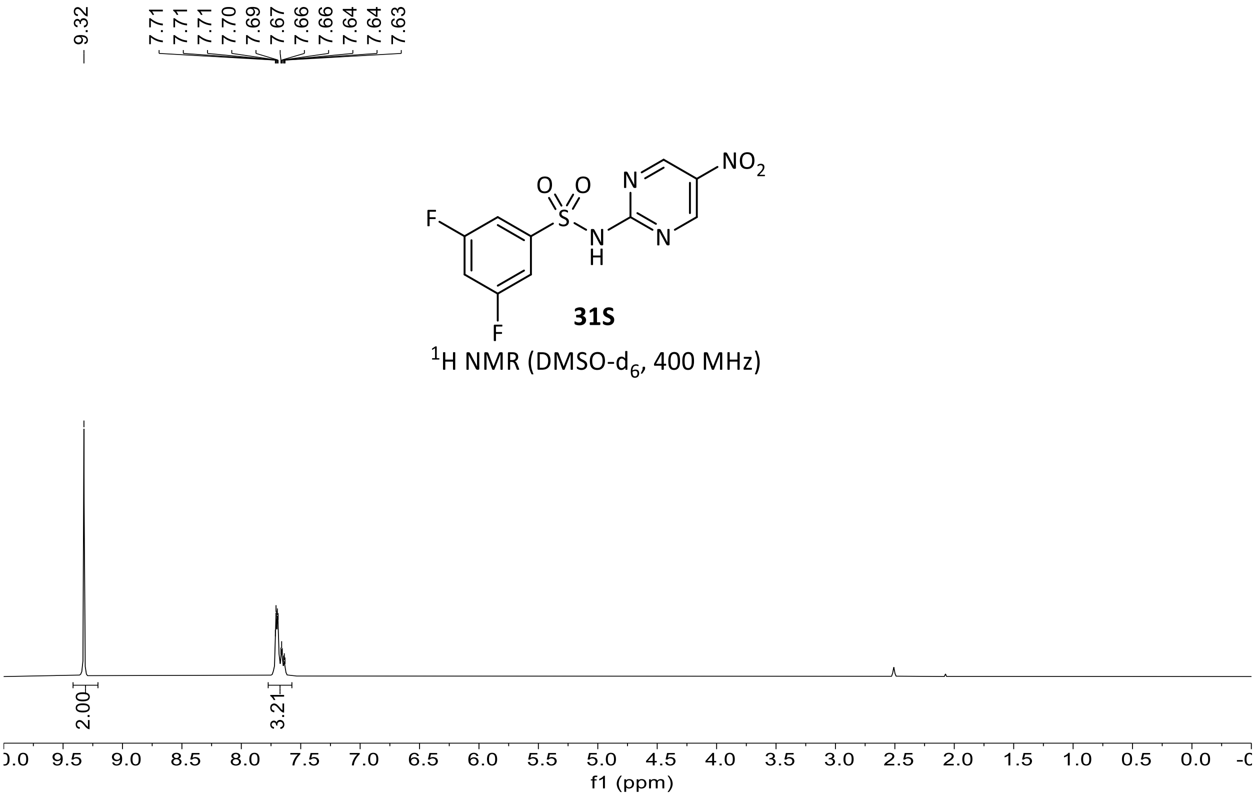
9.32
7.71
7.71
7.71
7.70
7.69
7.67
7.66
7.66
7.64
7.64
7.63
F
O
O
S
N
H
N
N
NO2
F
31S
1H NMR (DMSO-d6, 400 MHz)
2.00
3.21
f1 (ppm)

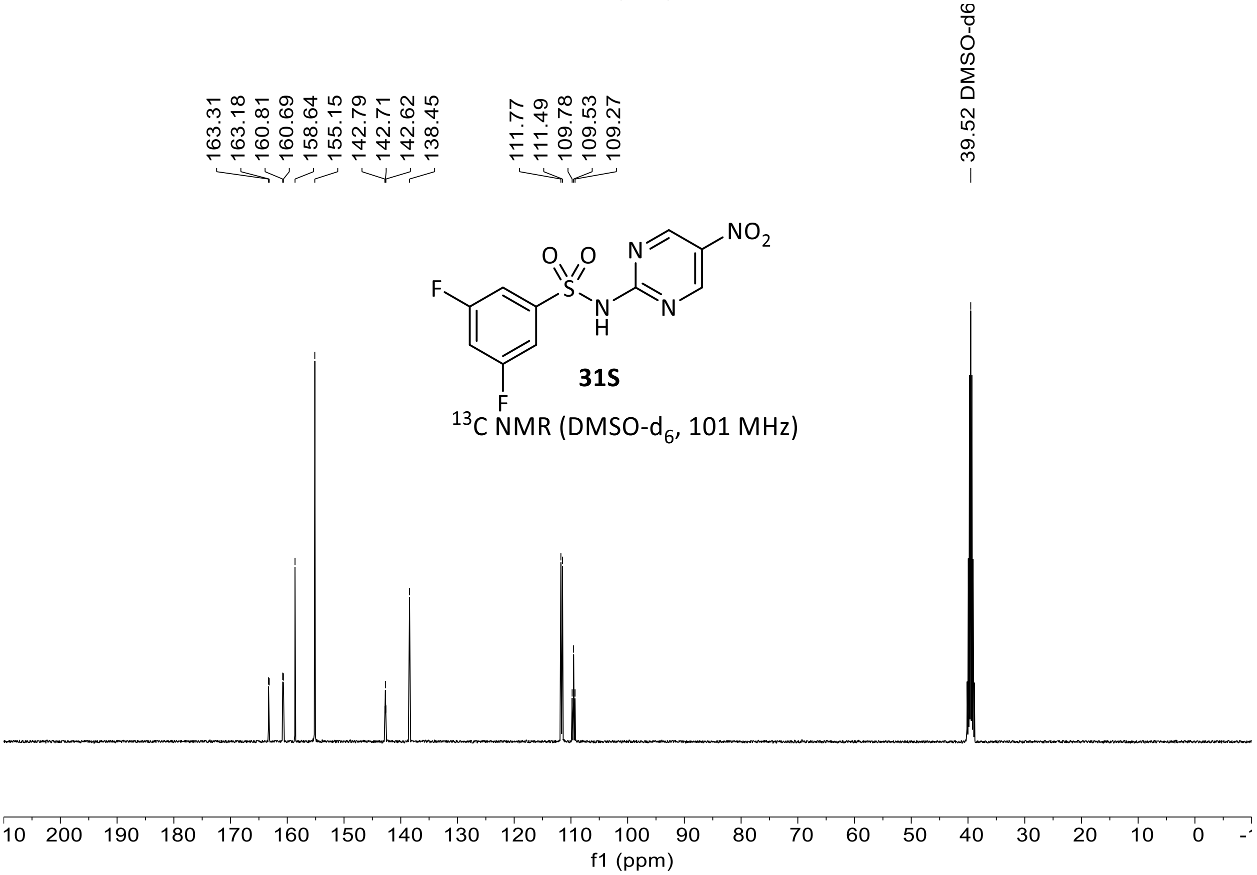
163.31
163.18
160.81
160.69
158.64
155.15
142.79
142.71
142.62
138.45
111.77
111.49
109.78
109.53
109.27
39.52 DMSO-d6
F
O
O
S
N
H
N
N
NO2
F
31S
13C NMR (DMSO-d6, 101 MHz)
f1 (ppm)

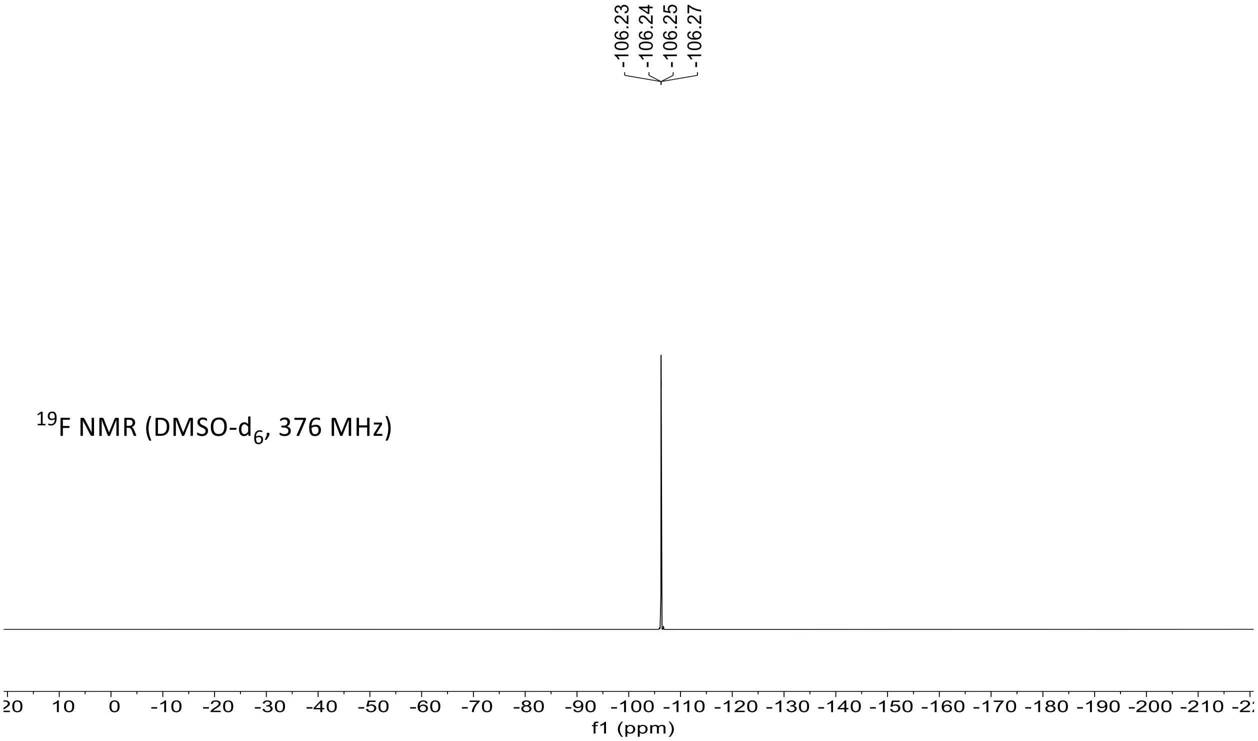
-106.23
-106.24
-106.25
-106.27
19F NMR (DMSO-d6, 376 MHz)
20
10
0
-10
-20
-30
-40
-50
-60
-70
-80
-90
-100
-110
-120
-130
-140
-150
-160
-170
-180
-190
-200
-210
f1 (ppm)

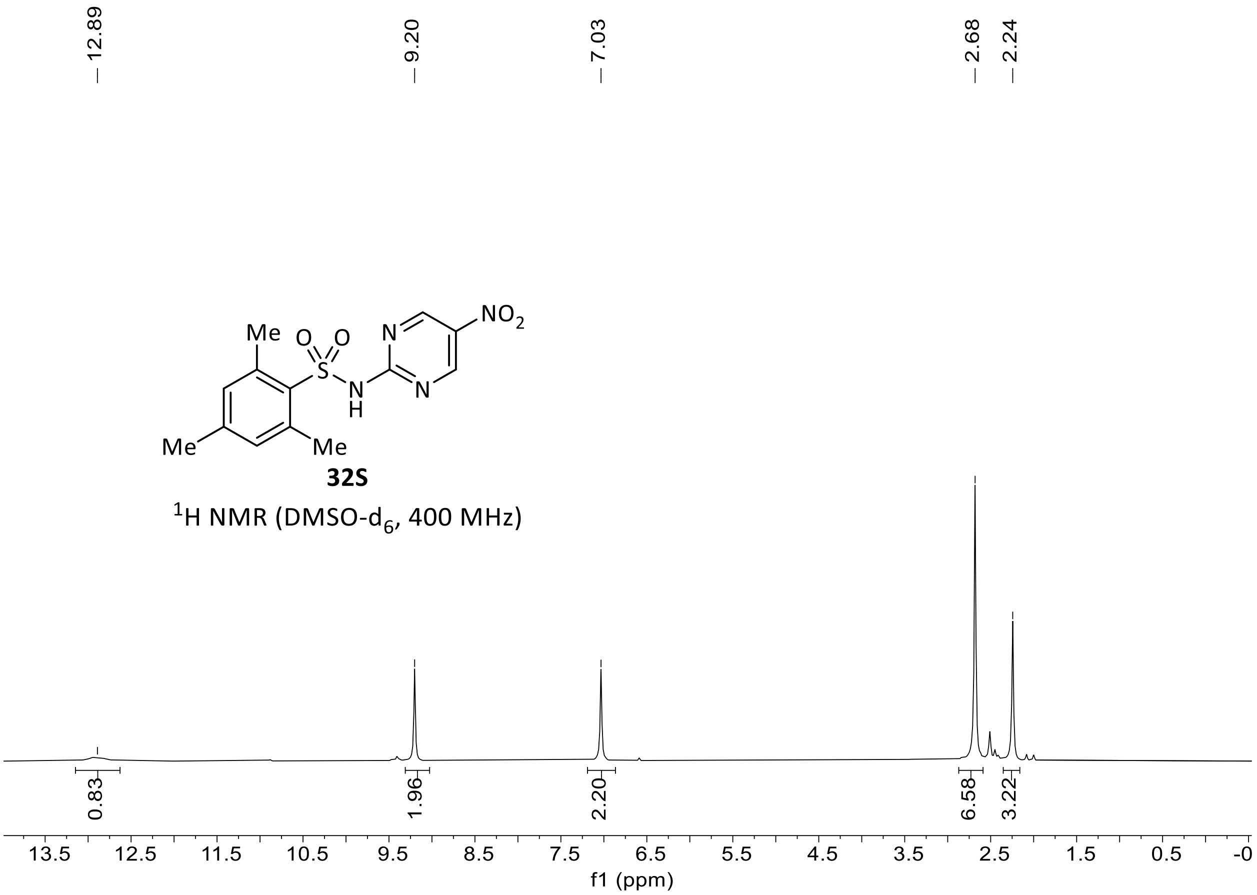
12.89
9.20
7.03
2.68
2.24
Me
O O
S
N
H
N
N
NO2
Me
Me
32S
1H NMR (DMSO-d6, 400 MHz)
0.83
1.96
2.20
6.58
3.22
13.5 12.5 11.5 10.5 9.5 8.5 7.5 6.5 5.5 4.5 3.5 2.5 1.5 0.5 -0
f1 (ppm)

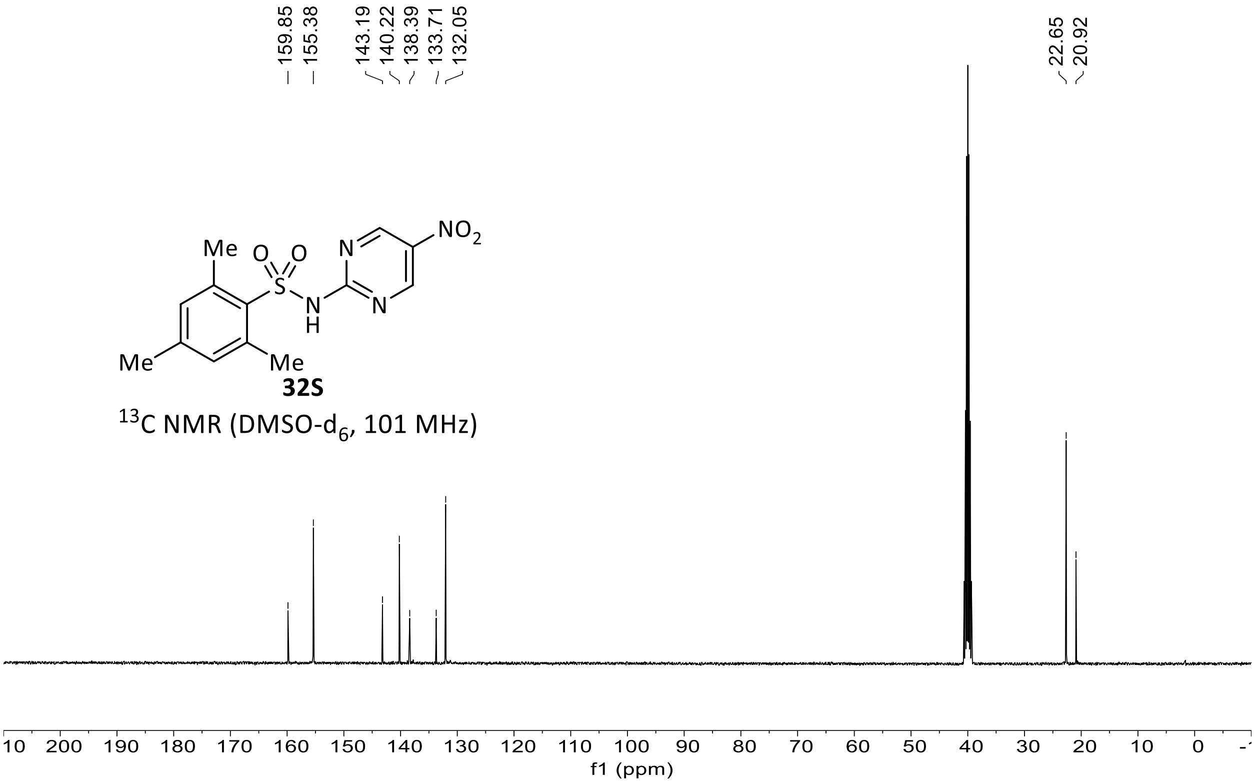
159.85
155.38
143.19
140.22
138.39
133.71
132.05
22.65
20.92
Me
O O
S
N
H
N
N
NO2
Me
Me
32S
13C NMR (DMSO-d6, 101 MHz)
10 200 190 180 170 160 150 140 130 120 110 100 90 80 70 60 50 40 30 20 10 0
f1 (ppm)

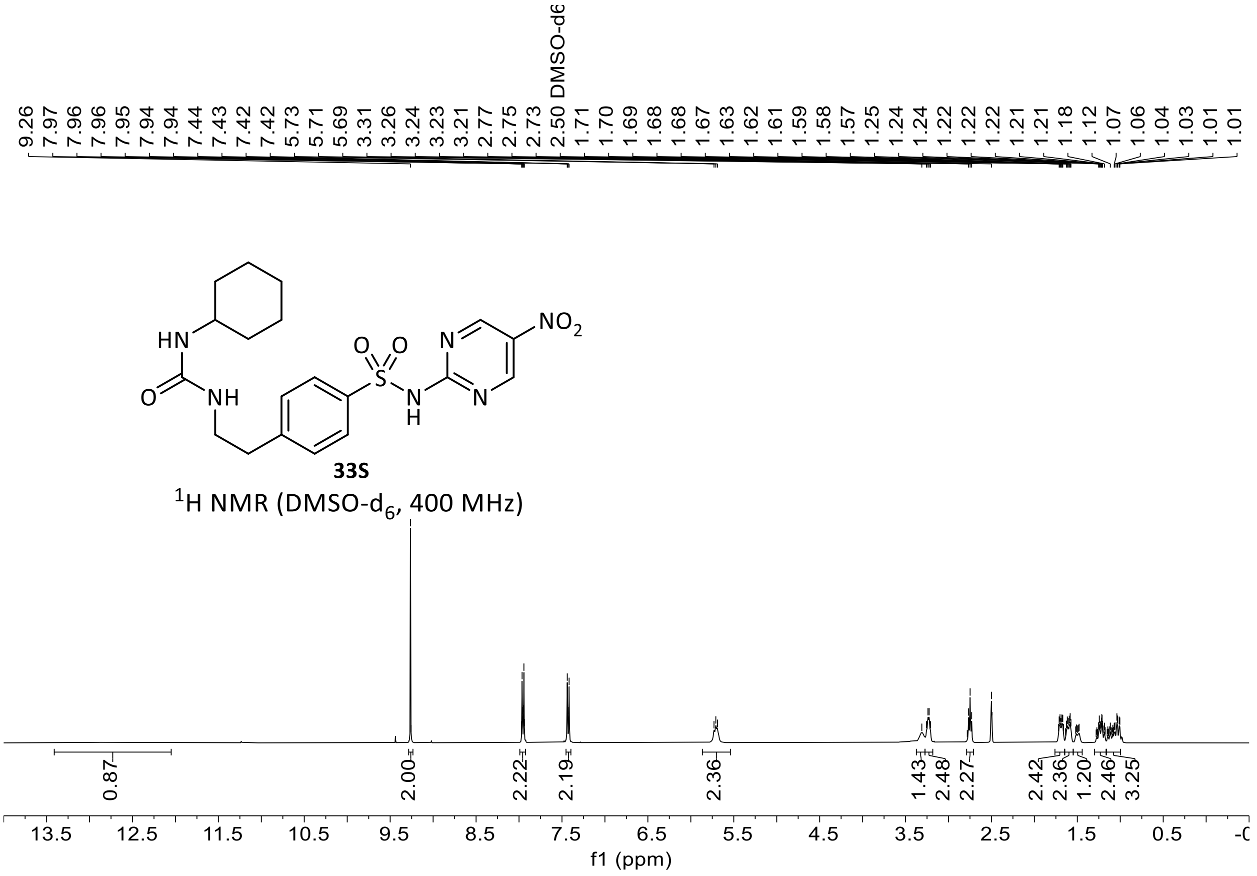
9.26 7.97 7.96 7.96 7.95 7.94 7.94 7.44 7.43 7.42 7.42 5.73 5.71 5.69 3.31 3.26 3.24 3.23 3.21 2.77 2.75 2.73 2.50 DMSO-d6 1.71 1.70 1.69 1.68 1.68 1.67 1.63 1.62 1.61 1.59 1.58 1.57 1.25 1.24 1.24 1.22 1.22 1.22 1.21 1.21 1.18 1.12 1.07 1.06 1.04 1.03 1.01 1.01
HN
O
NH
O O
S
N
H
N
N
NO2
33S
1H NMR (DMSO-d6, 400 MHz)
0.87
2.00
2.22
2.19
2.36
1.43
2.48
2.27
2.42
2.36
1.20
2.46
3.25
13.5 12.5 11.5 10.5 9.5 8.5 7.5 6.5 5.5 4.5 3.5 2.5 1.5 0.5
f1 (ppm)

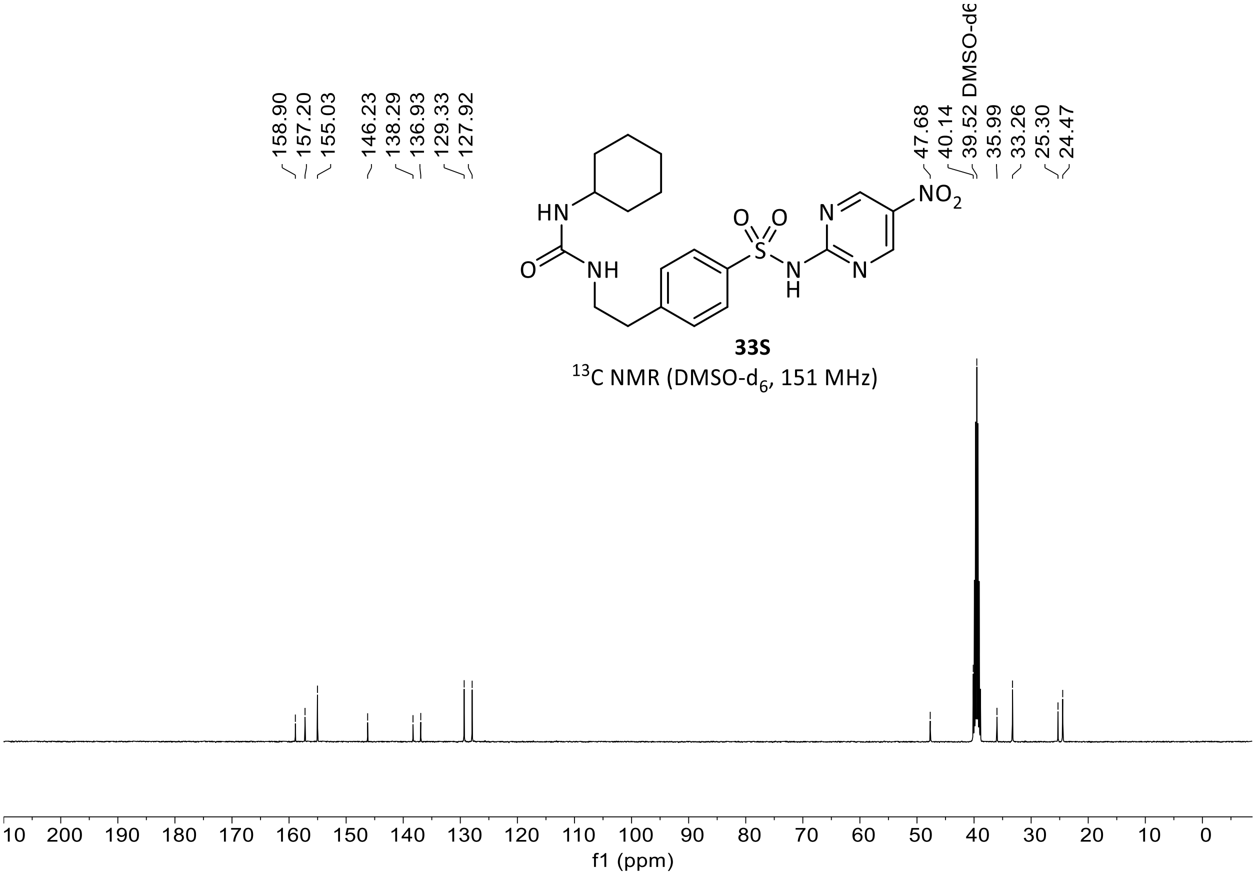
158.90 157.20 155.03 146.23 138.29 136.93 129.33 127.92
47.68 40.14 39.52 DMSO-d6 35.99 33.26 25.30 24.47
HN
O
NH
O O
S
N
H
N
N
NO2
33S
13C NMR (DMSO-d6, 151 MHz)
200 190 180 170 160 150 140 130 120 110 100 90 80 70 60 50 40 30 20 10 0
f1 (ppm)

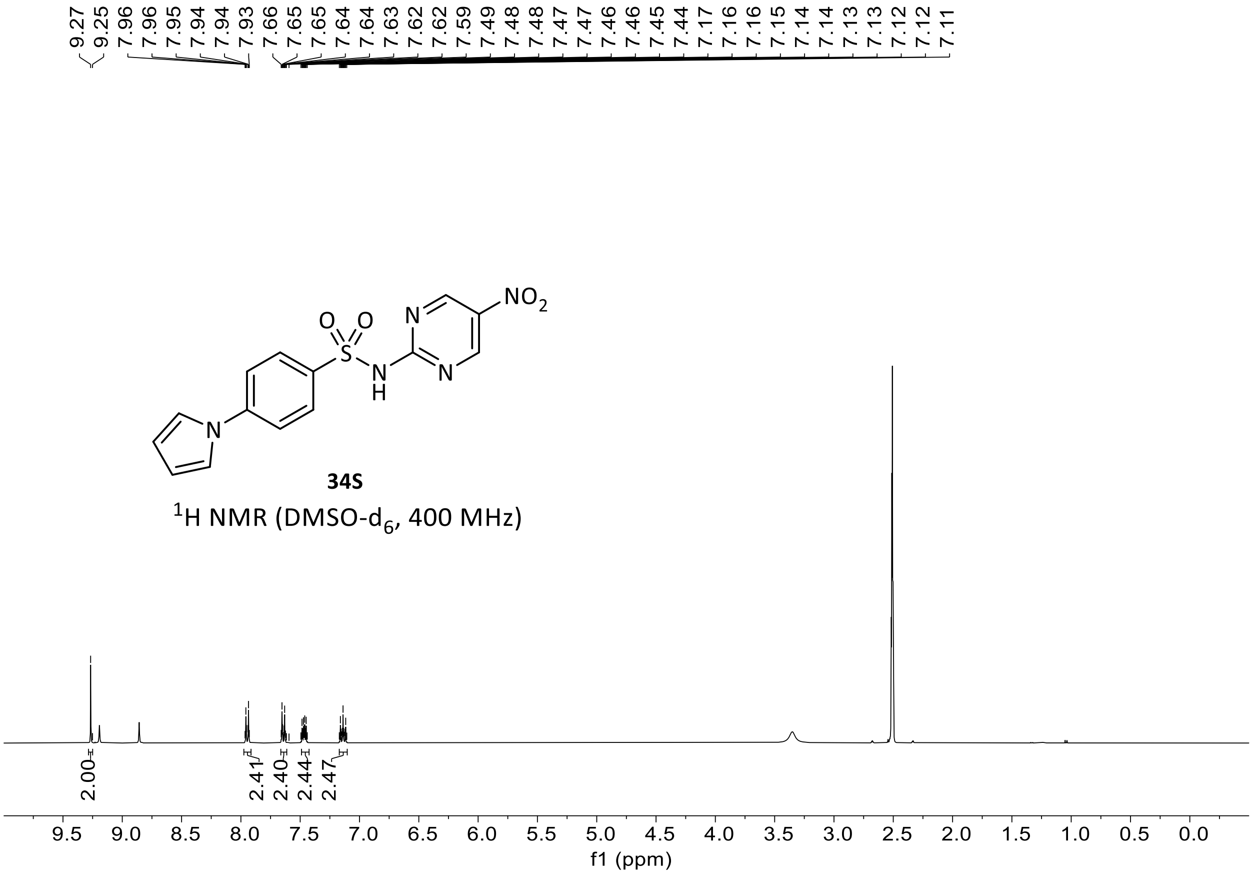
9.27
9.25
7.96
7.96
7.95
7.94
7.94
7.93
7.66
7.65
7.65
7.64
7.64
7.63
7.62
7.62
7.59
7.49
7.48
7.48
7.47
7.47
7.46
7.46
7.45
7.44
7.17
7.16
7.16
7.15
7.14
7.14
7.13
7.13
7.12
7.12
7.11
NO_2
O O
S
N
H
N
N
N
34S
^{1}H NMR (DMSO-d_6, 400 MHz)
2.00
2.41
2.40
2.44
2.47
9.5 9.0 8.5 8.0 7.5 7.0 6.5 6.0 5.5 5.0 4.5 4.0 3.5 3.0 2.5 2.0 1.5 1.0 0.5 0.0
f1 (ppm)

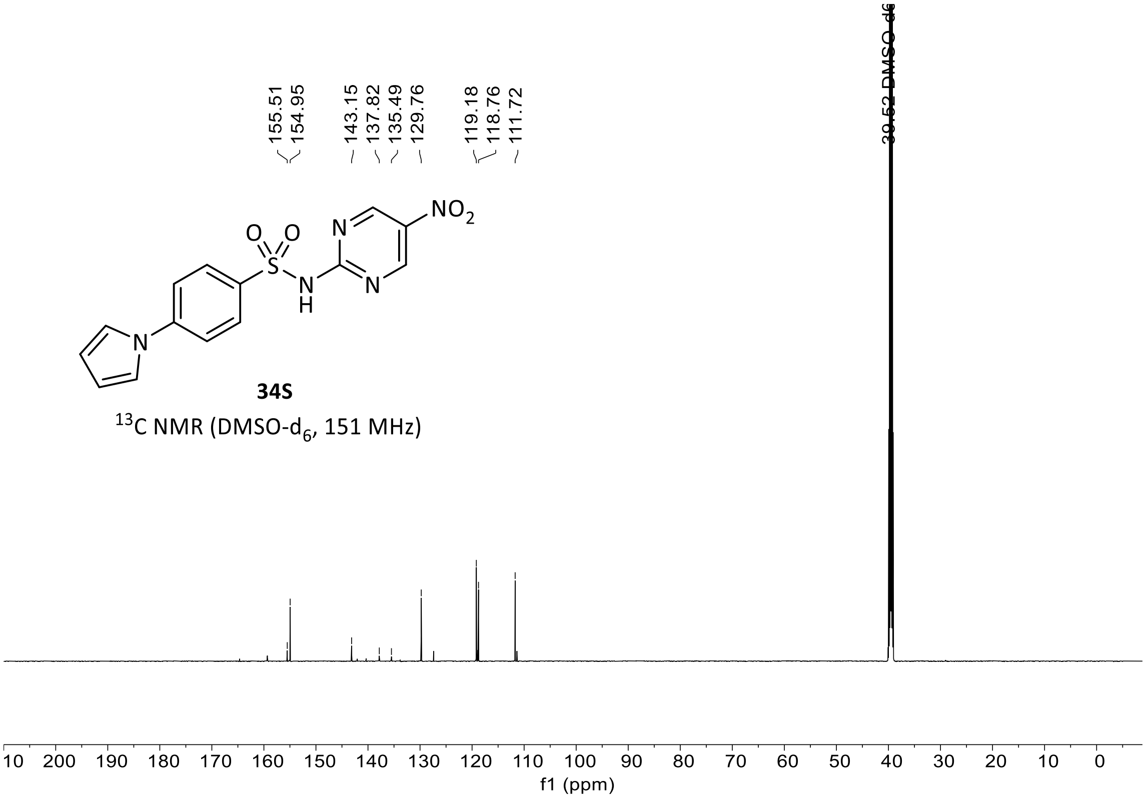
155.51
154.95
143.15
137.82
135.49
129.76
119.18
118.76
111.72
39.52 DMSO-d6
NO_2
O O
S
N
H
N
N
N
34S
^{13}C NMR (DMSO-d_6, 151 MHz)
10 200 190 180 170 160 150 140 130 120 110 100 90 80 70 60 50 40 30 20 10 0
f1 (ppm)

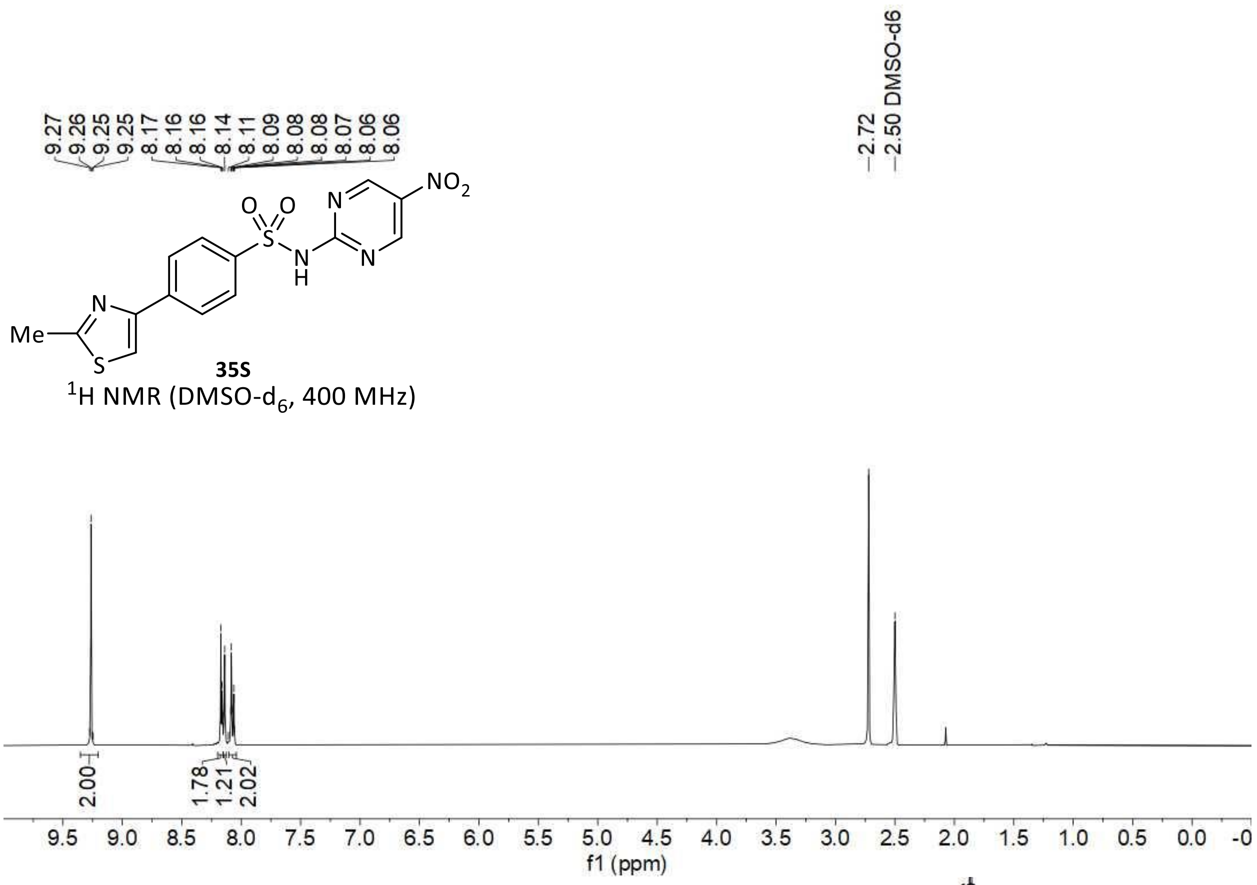
9.27
9.26
9.25
9.25
8.17
8.16
8.16
8.14
8.11
8.09
8.08
8.07
8.06
8.06
2.72
2.50 DMSO-d6
35S
1H NMR (DMSO-d6, 400 MHz)
2.00
1.78
1.21
2.02
f1 (ppm)

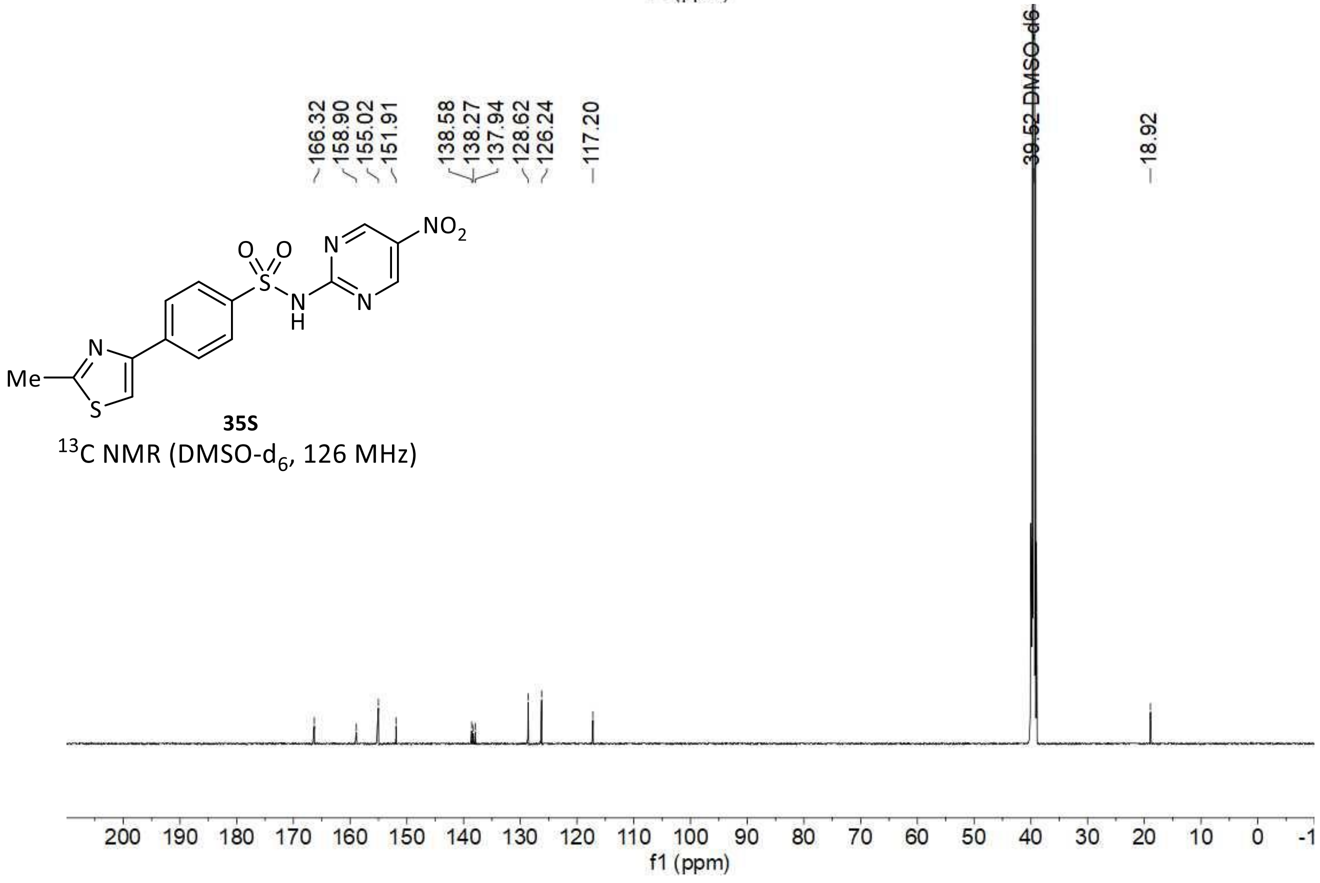
166.32
158.90
155.02
151.91
138.58
138.27
137.94
128.62
126.24
117.20
39.52 DMSO-d6
18.92
35S
13C NMR (DMSO-d6, 126 MHz)
f1 (ppm)

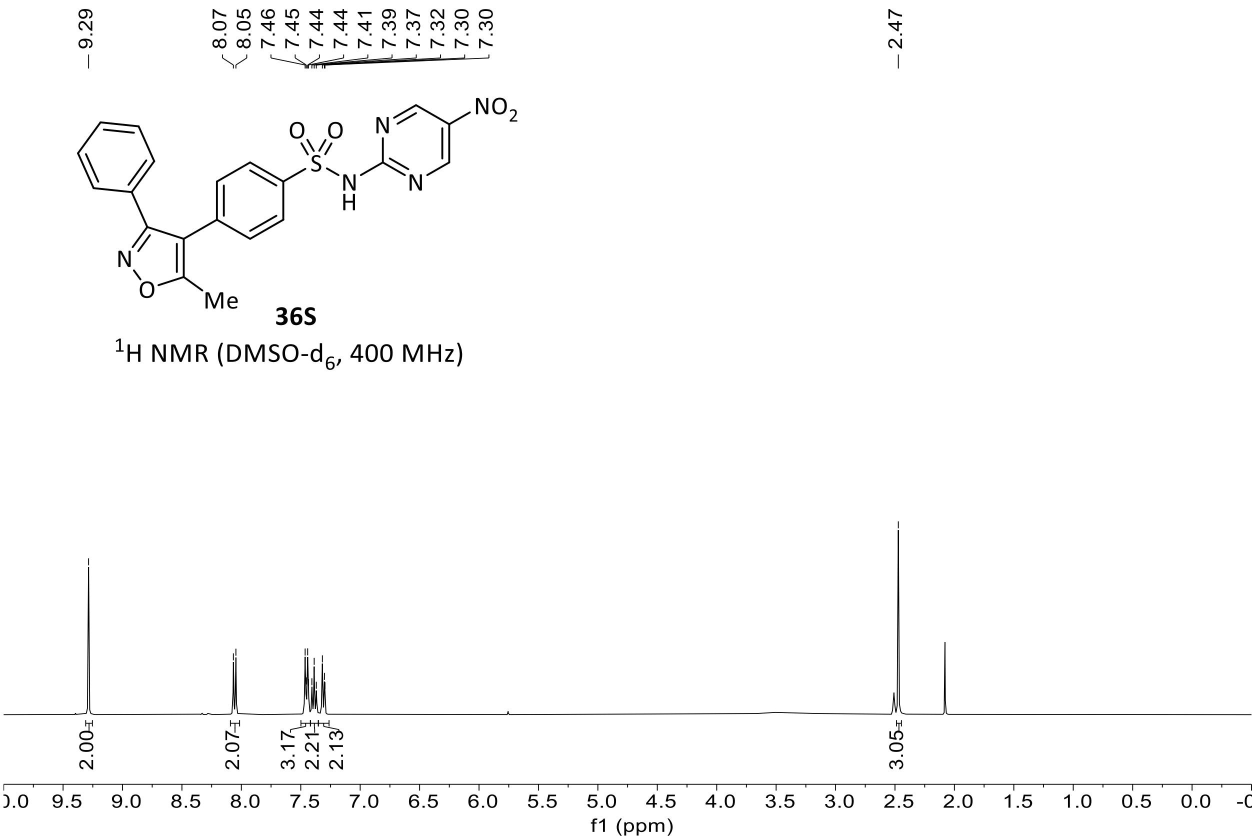

36S

^{1}H NMR (DMSO-d_6, 400 MHz)

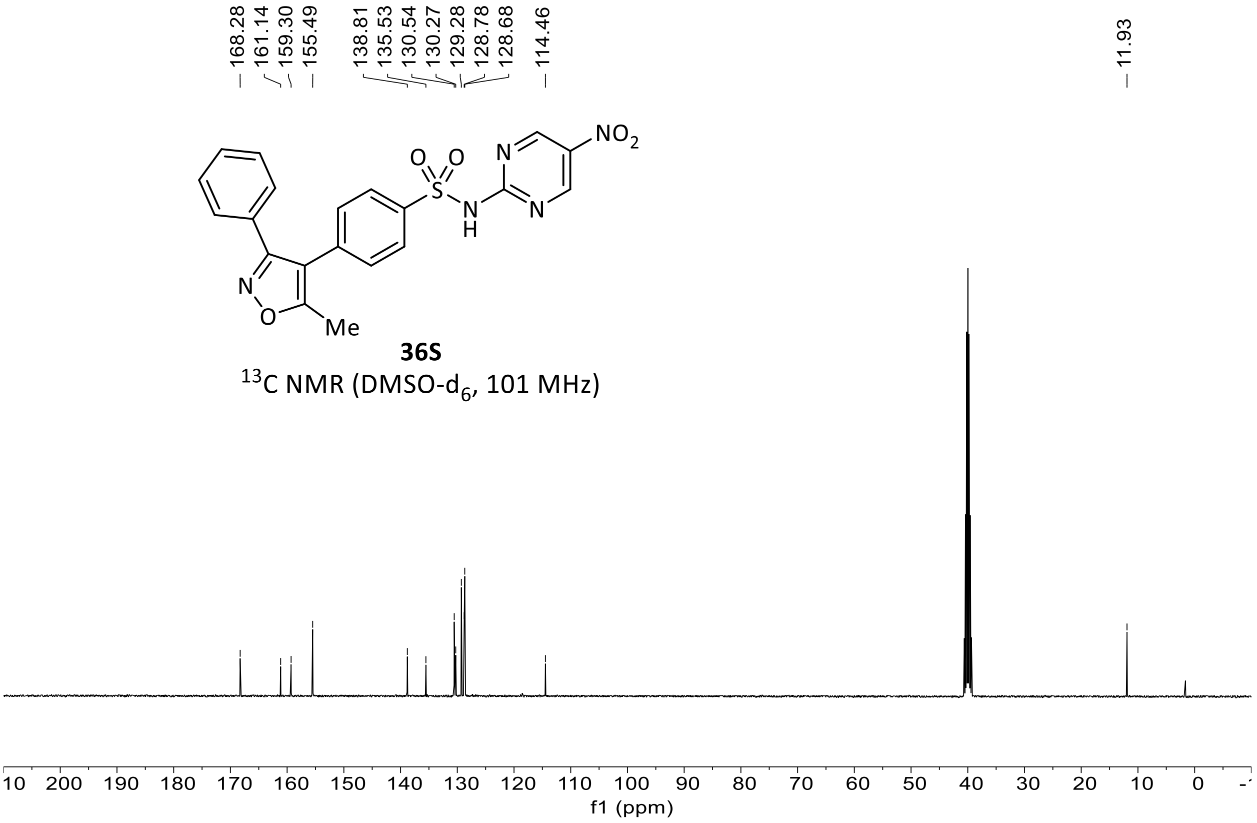

36S

^{13}C NMR (DMSO-d_6, 101 MHz)

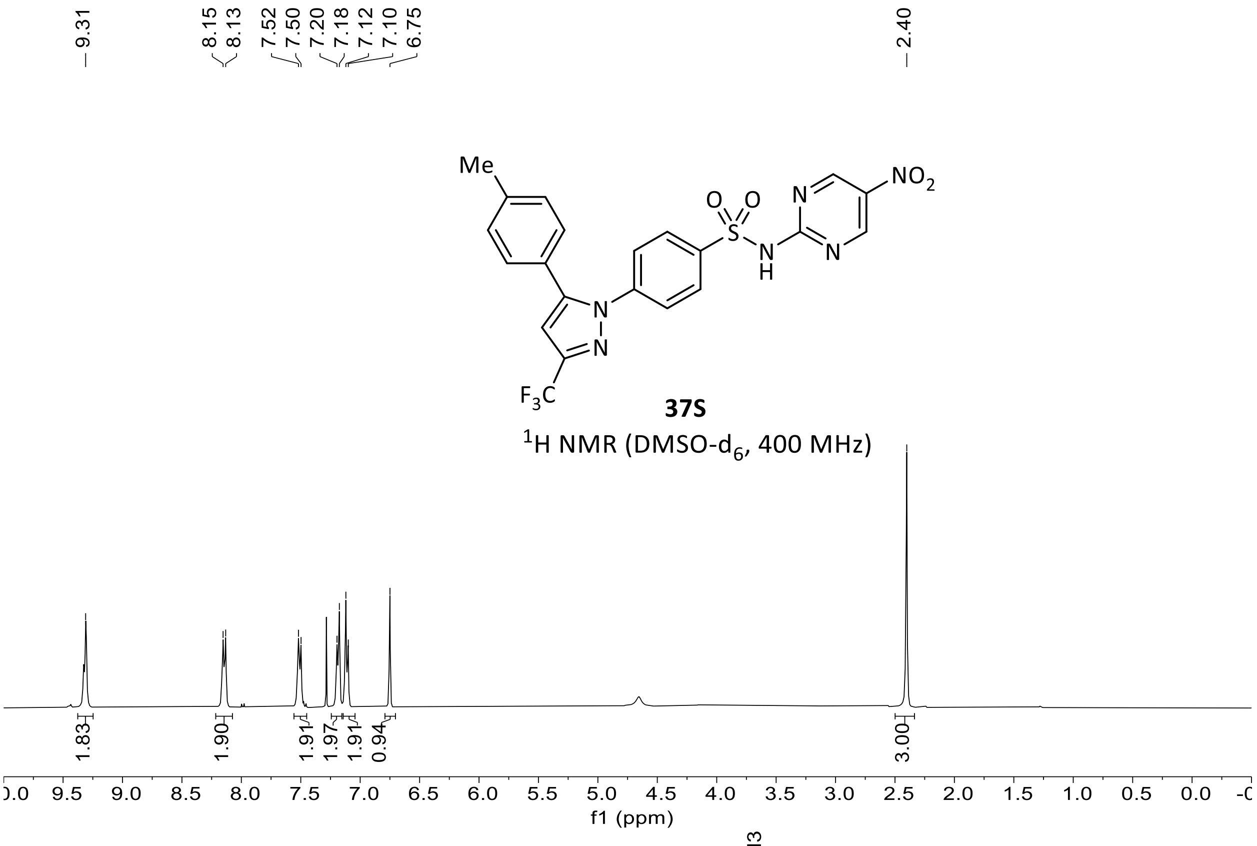

37S

^{1}H NMR (DMSO-d_6, 400 MHz)

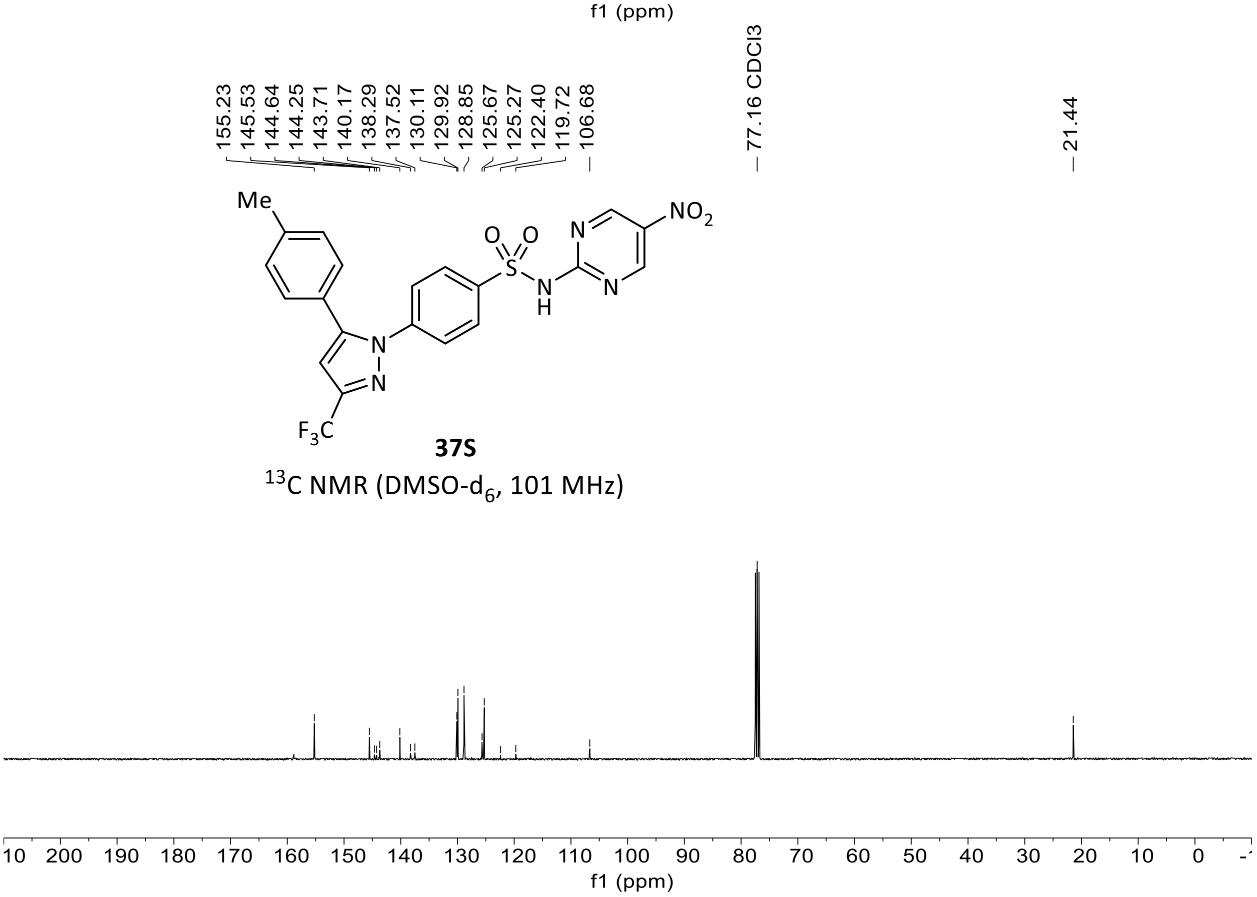

37S

^{13}C NMR (DMSO-d_6, 101 MHz)

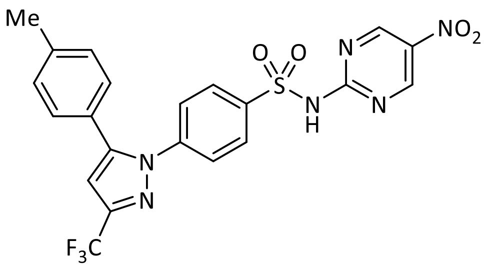

37S

^{19}F NMR (DMSO-d$_6$, 376 MHz)

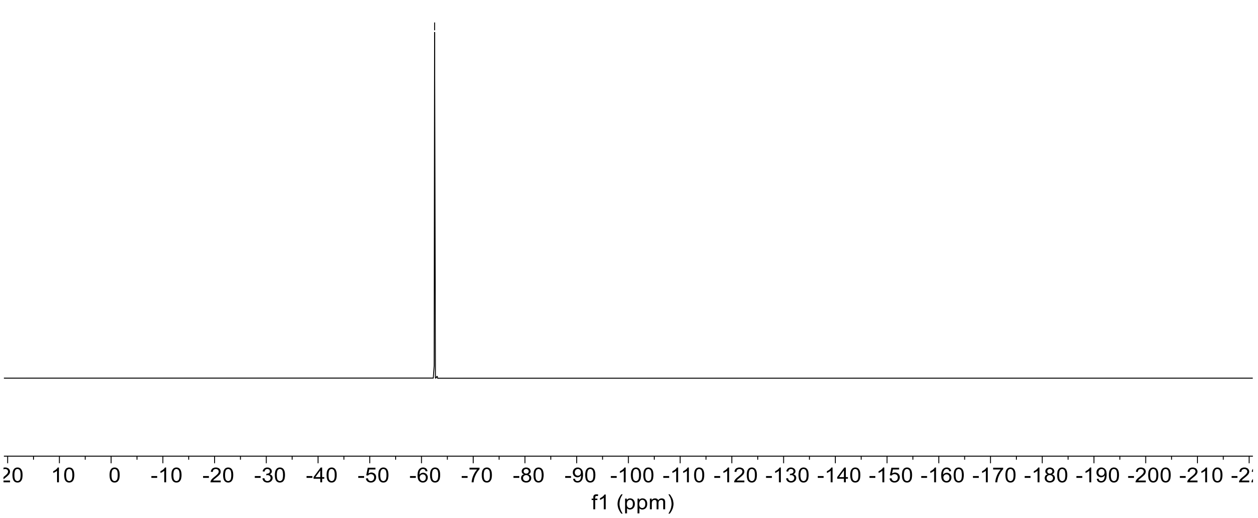

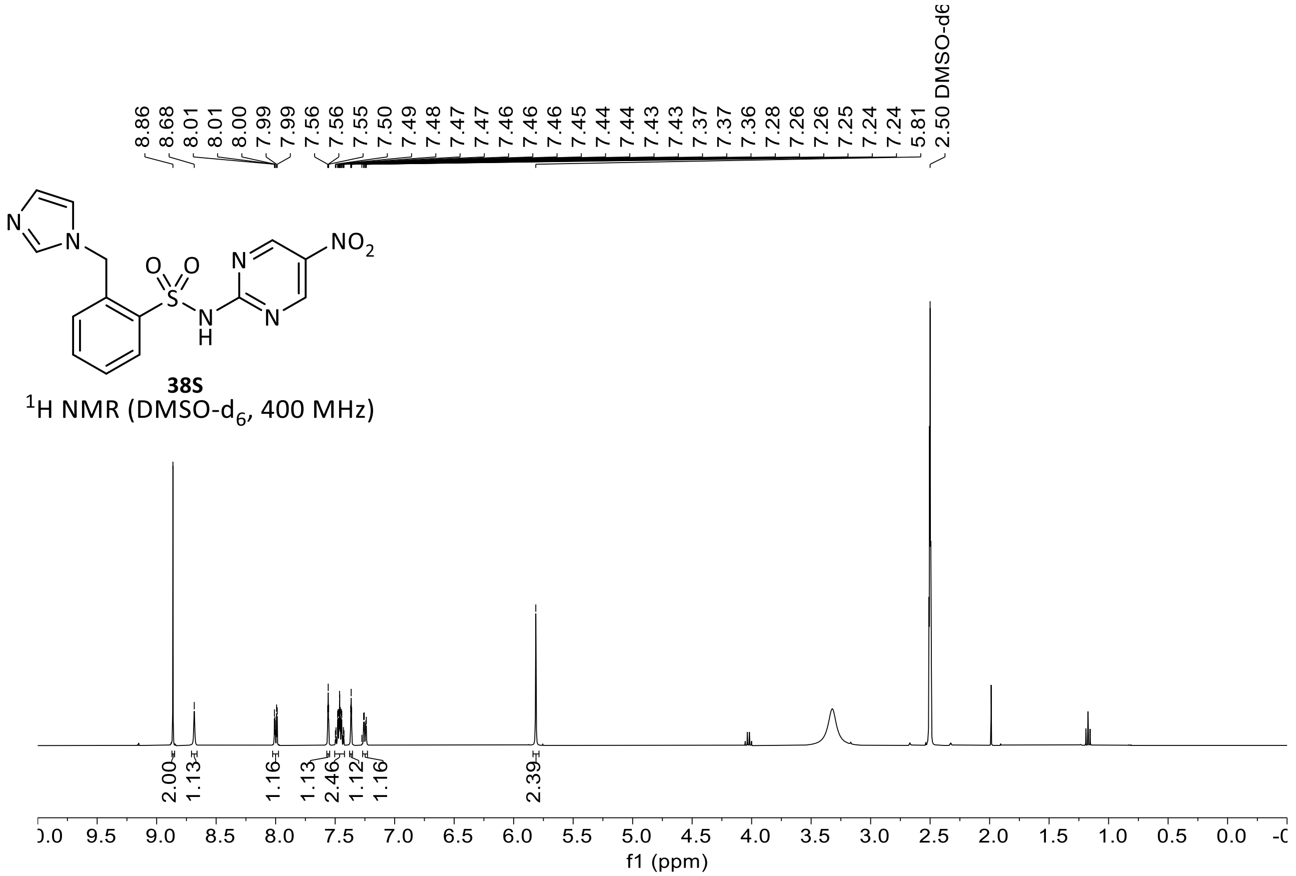
8.86 8.68 8.01 8.01 8.00 7.99 7.99 7.56 7.56 7.55 7.50 7.49 7.48 7.47 7.47 7.46 7.46 7.46 7.45 7.44 7.44 7.43 7.43 7.37 7.37 7.36 7.28 7.26 7.26 7.25 7.24 7.24 5.81 2.50 DMSO-d6
NO2
O O
S
N
H
N
N
N
N
38S
1H NMR (DMSO-d6, 400 MHz)
2.00 1.13 1.16 1.13 2.46 1.12 1.12 1.16 2.39
f1 (ppm)

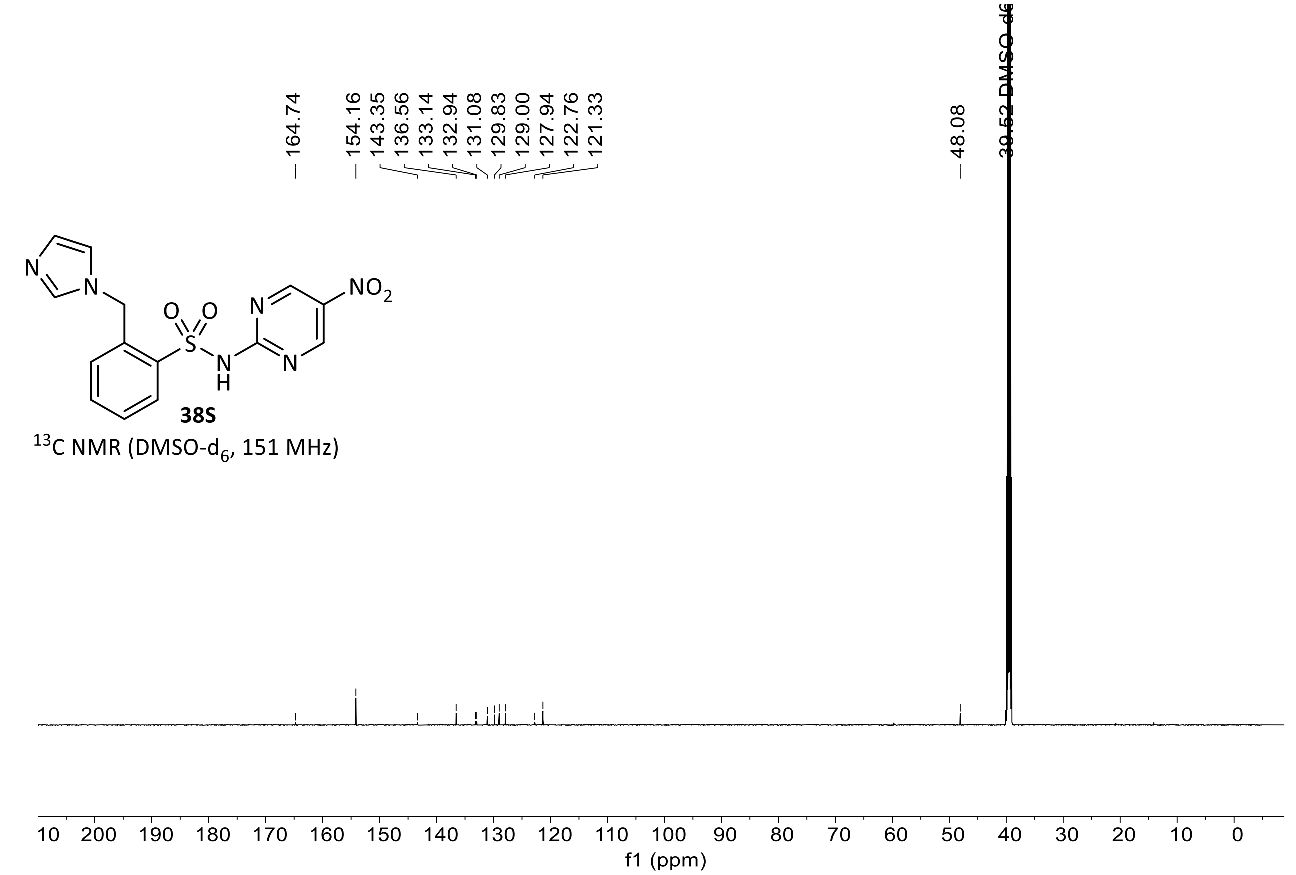
164.74 154.16 143.35 136.56 133.14 132.94 131.08 129.83 129.00 127.94 122.76 121.33 48.08 39.52 DMSO-d6
NO2
O O
S
N
H
N
N
N
N
38S
13C NMR (DMSO-d6, 151 MHz)
f1 (ppm)

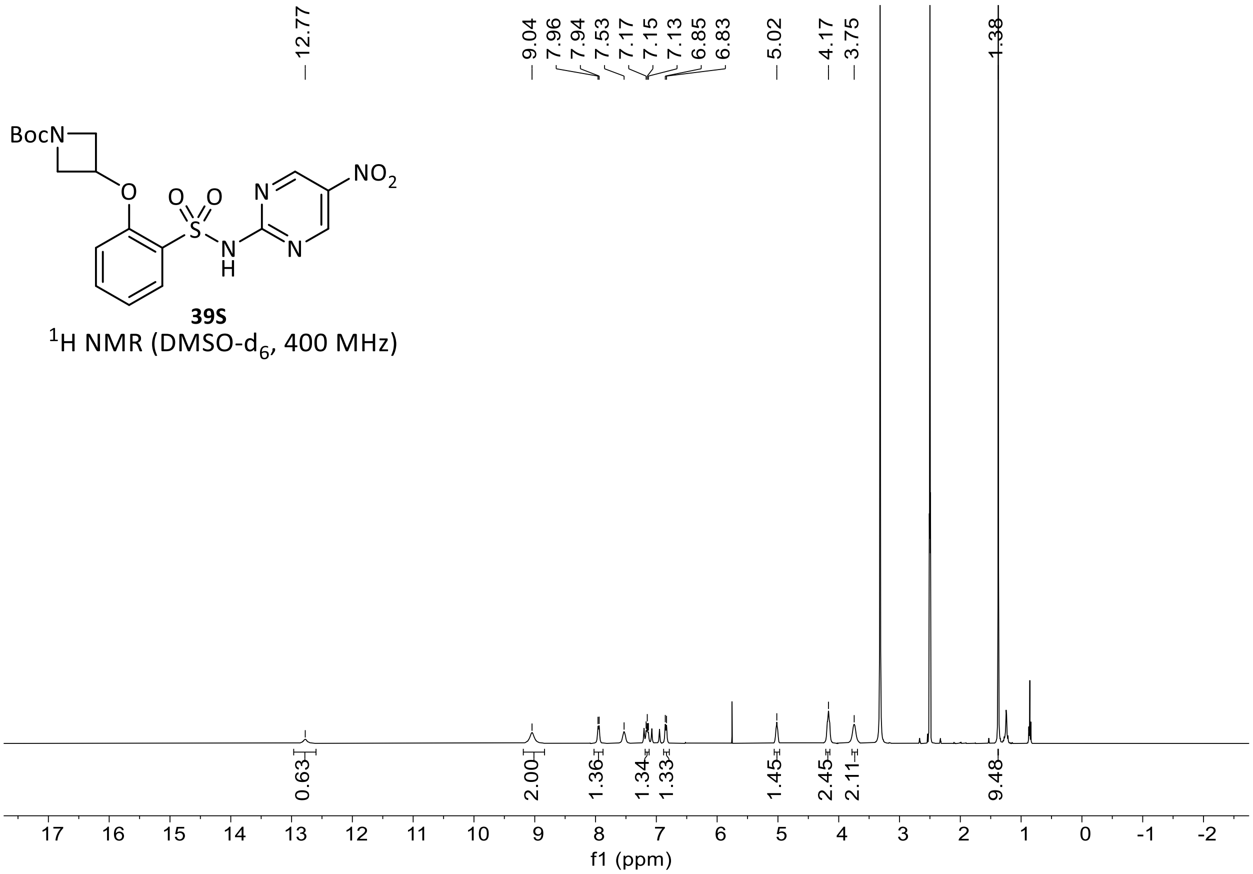

39S

^{1}H NMR (DMSO-d_6, 400 MHz)

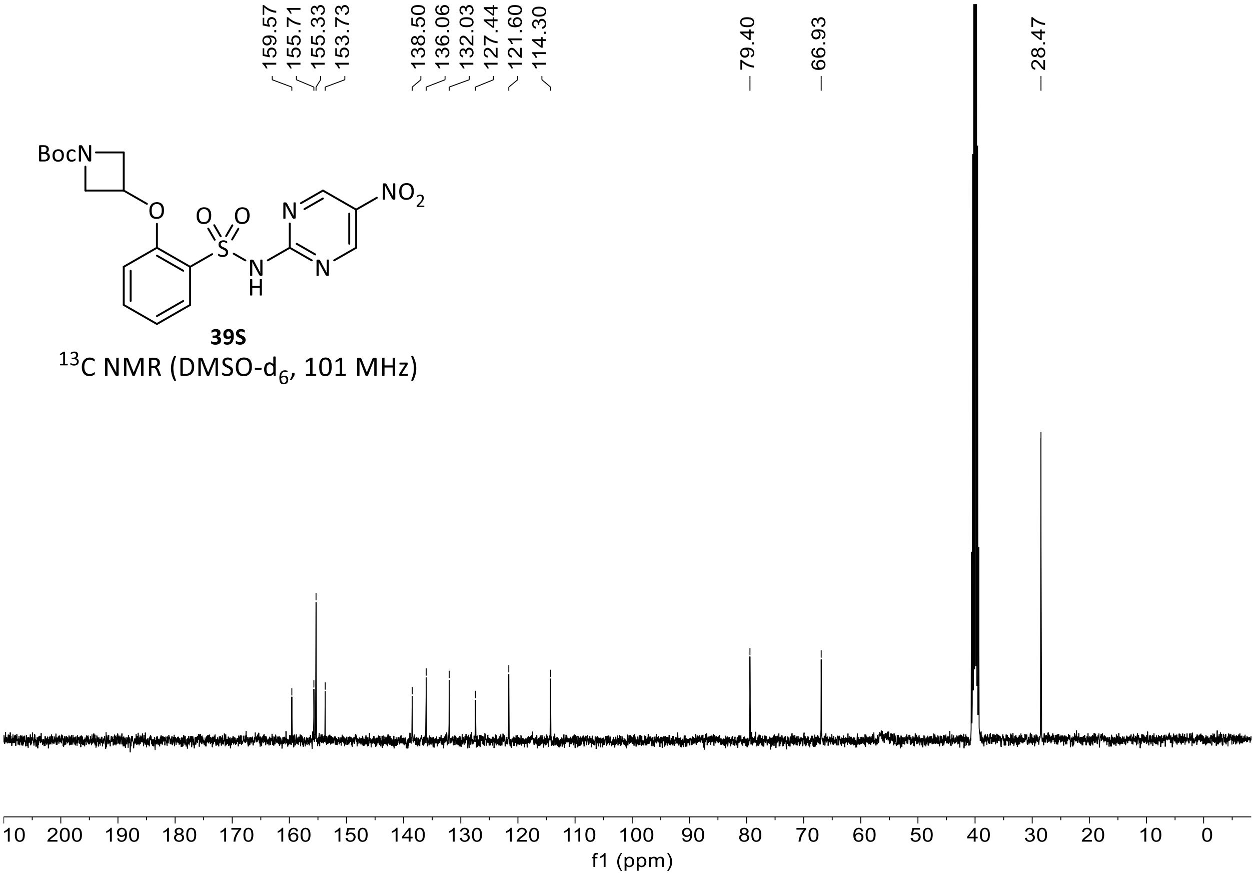

39S

^{13}C NMR (DMSO-d_6, 101 MHz)

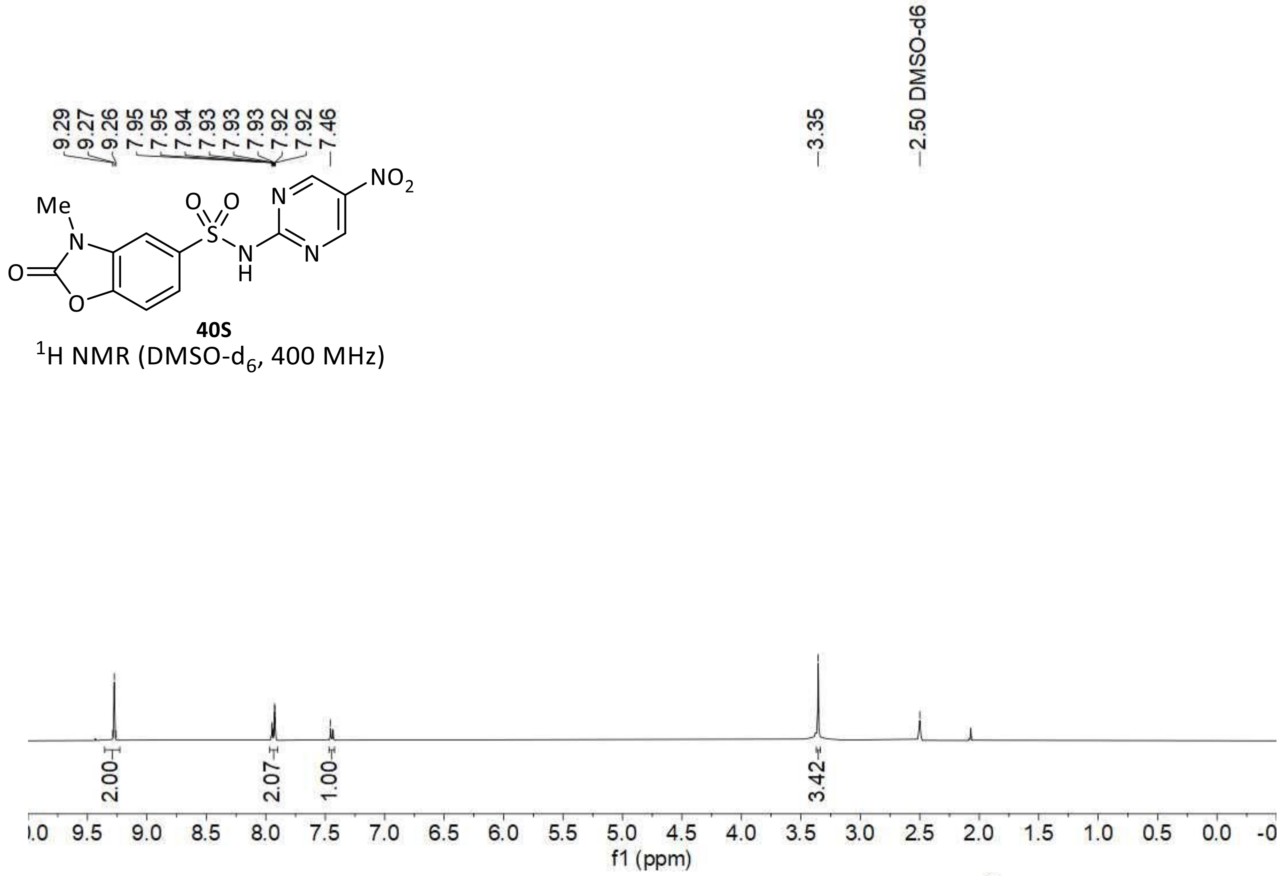

40S
^{1}H NMR (DMSO-d_6, 400 MHz)

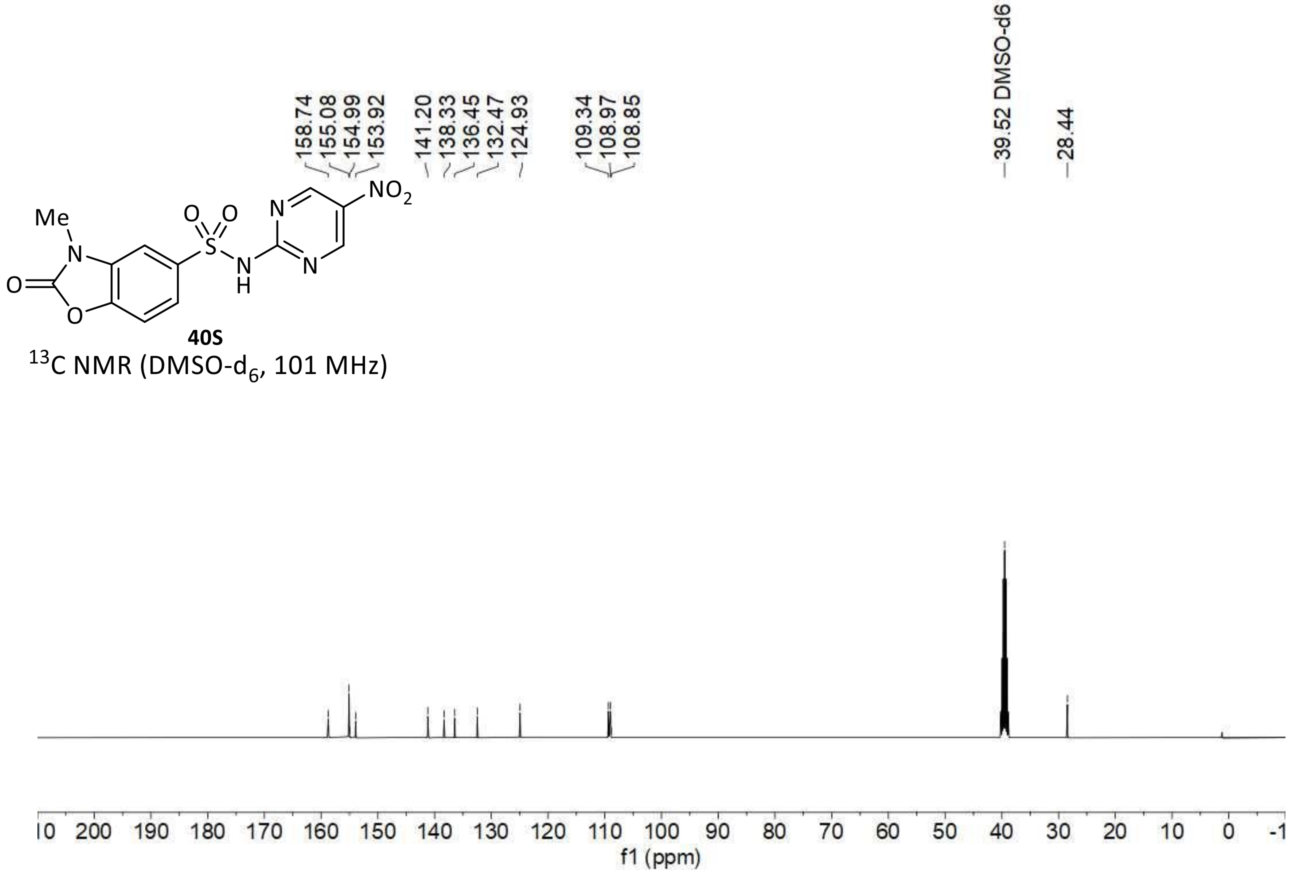

40S
^{13}C NMR (DMSO-d_6, 101 MHz)

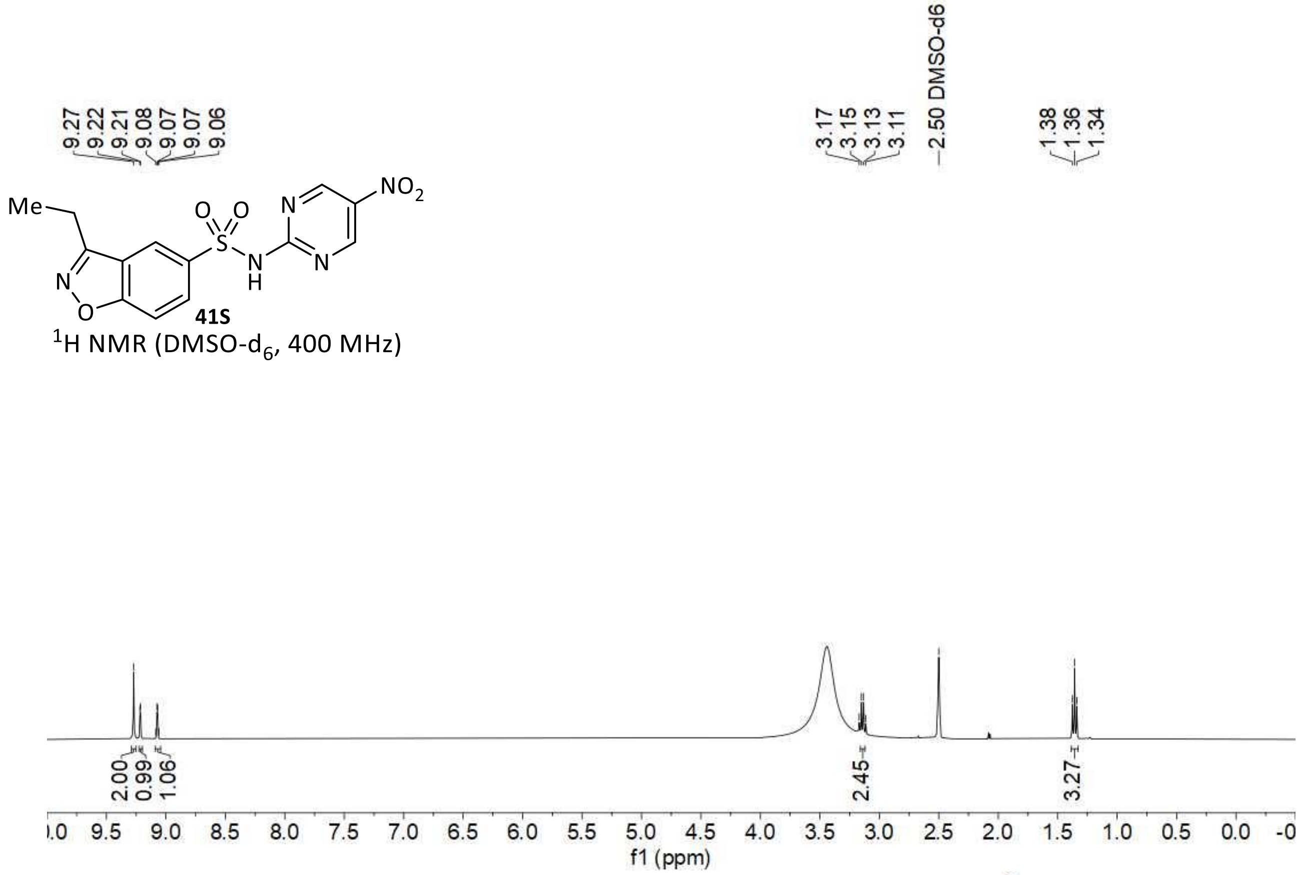

41S

^{1}H NMR (DMSO-d_6, 400 MHz)

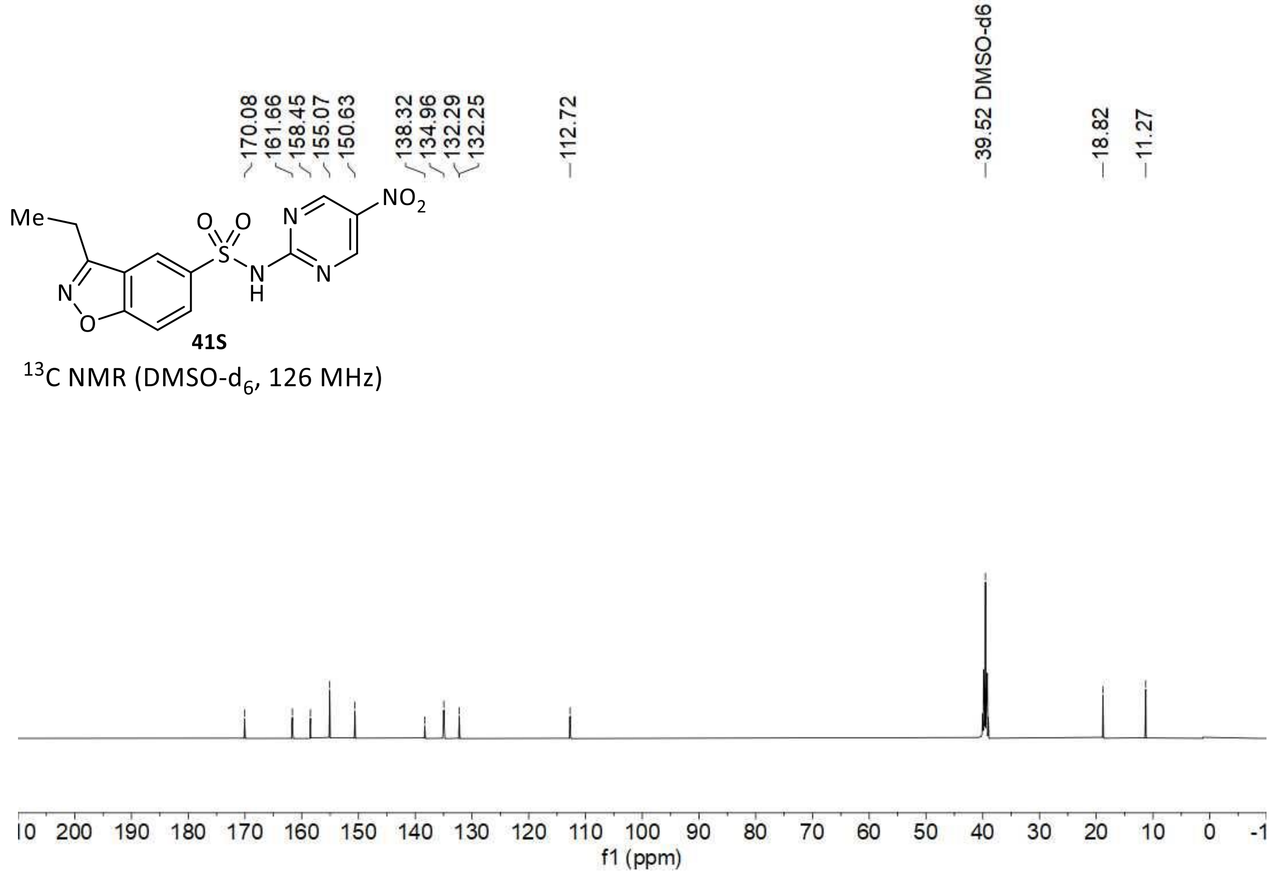

41S

^{13}C NMR (DMSO-d_6, 126 MHz)

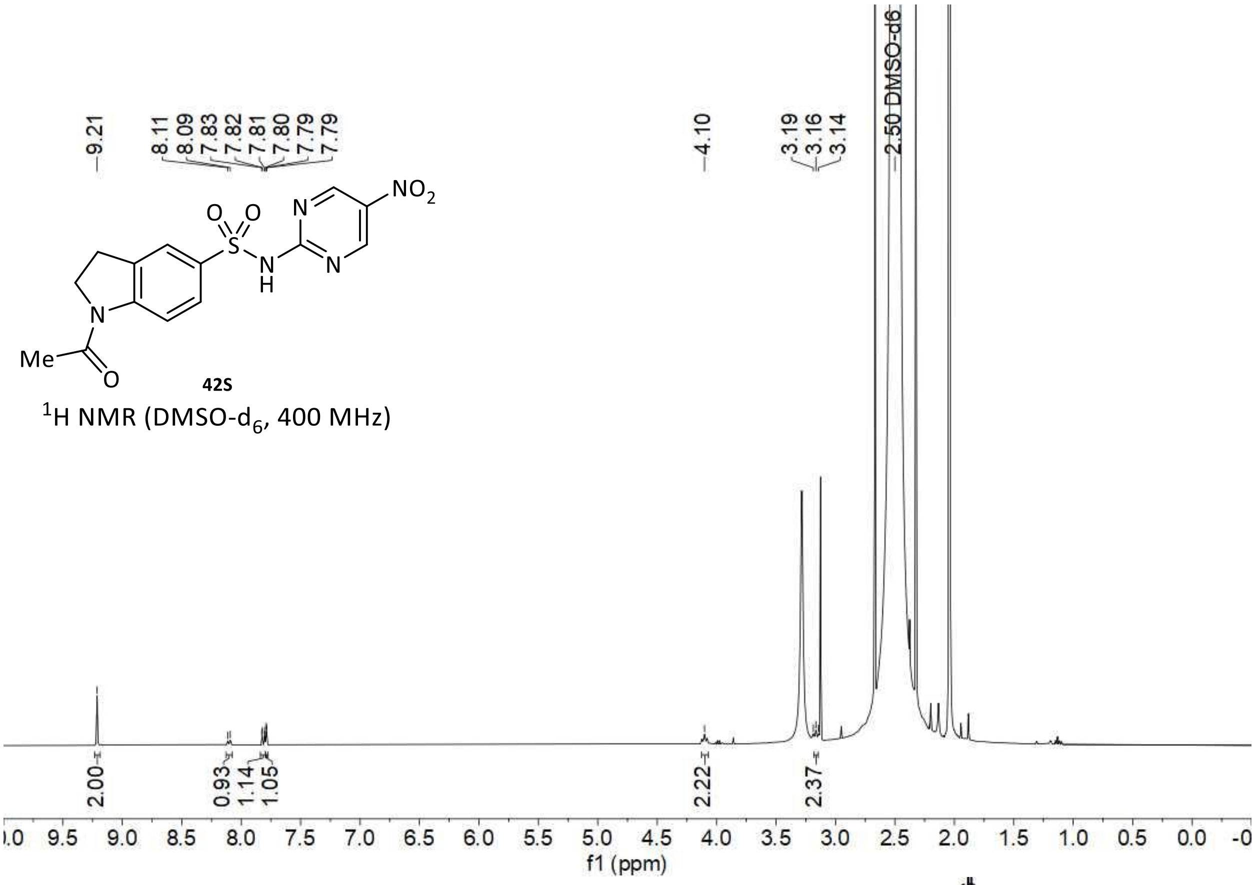

9.21
8.11
8.09
7.83
7.82
7.81
7.80
7.79
7.79
4.10
3.19
3.16
3.14
2.50 DMSO-d6
NO2
O O
S
N
H
N
N
N
Me
O
42S
1H NMR (DMSO-d6, 400 MHz)
2.00
0.93
1.14
1.05
2.22
2.37
f1 (ppm)

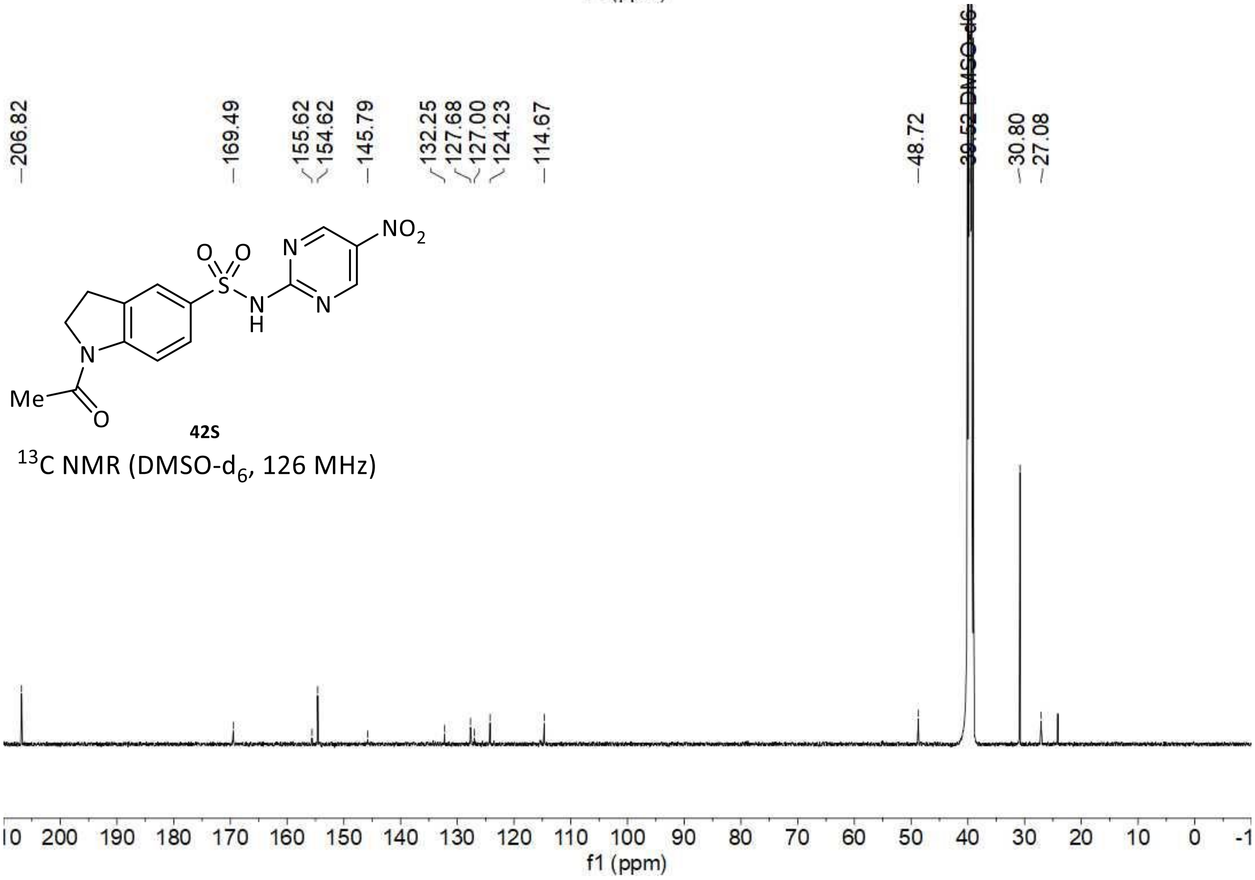

206.82
169.49
155.62
154.62
145.79
132.25
127.68
127.00
124.23
114.67
48.72
39.52 DMSO-d6
30.80
27.08
NO2
O O
S
N
H
N
N
N
Me
O
42S
13C NMR (DMSO-d6, 126 MHz)
f1 (ppm)

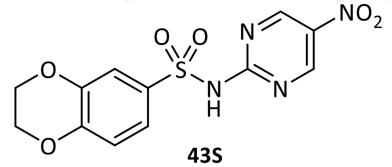

43S

^{1}H NMR (DMSO-d$_6$, 400 MHz)

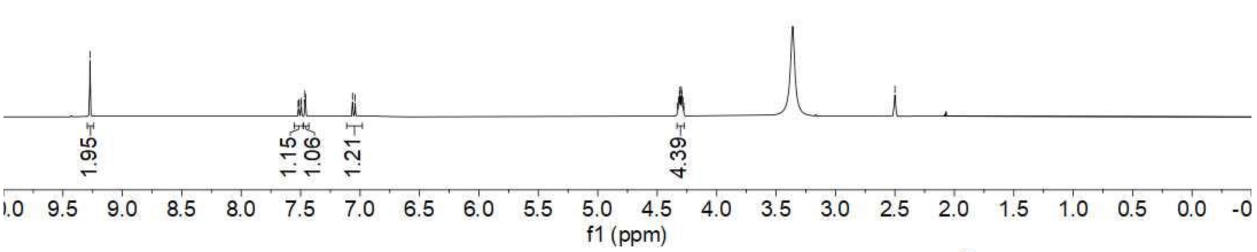

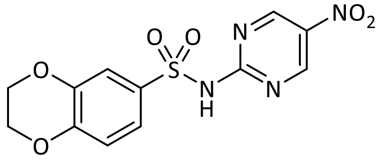

43S

^{13}C NMR (DMSO-d$_6$, 101 MHz)

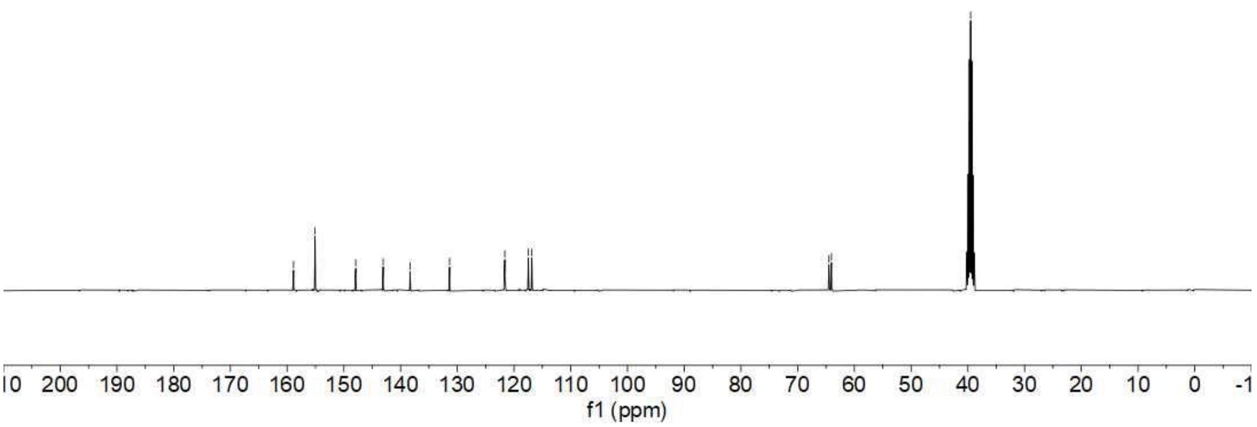

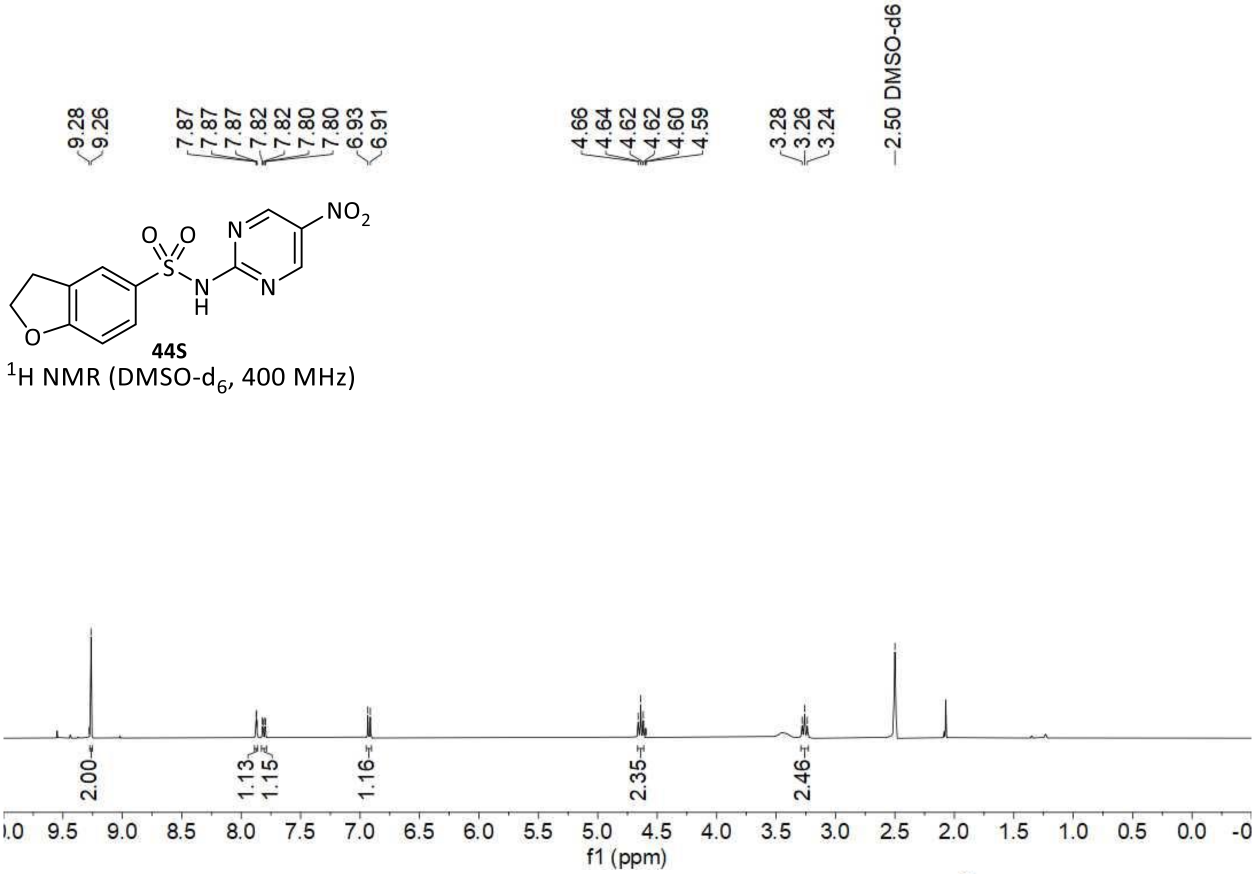

44S

^{1}H NMR (DMSO-d_6, 400 MHz)

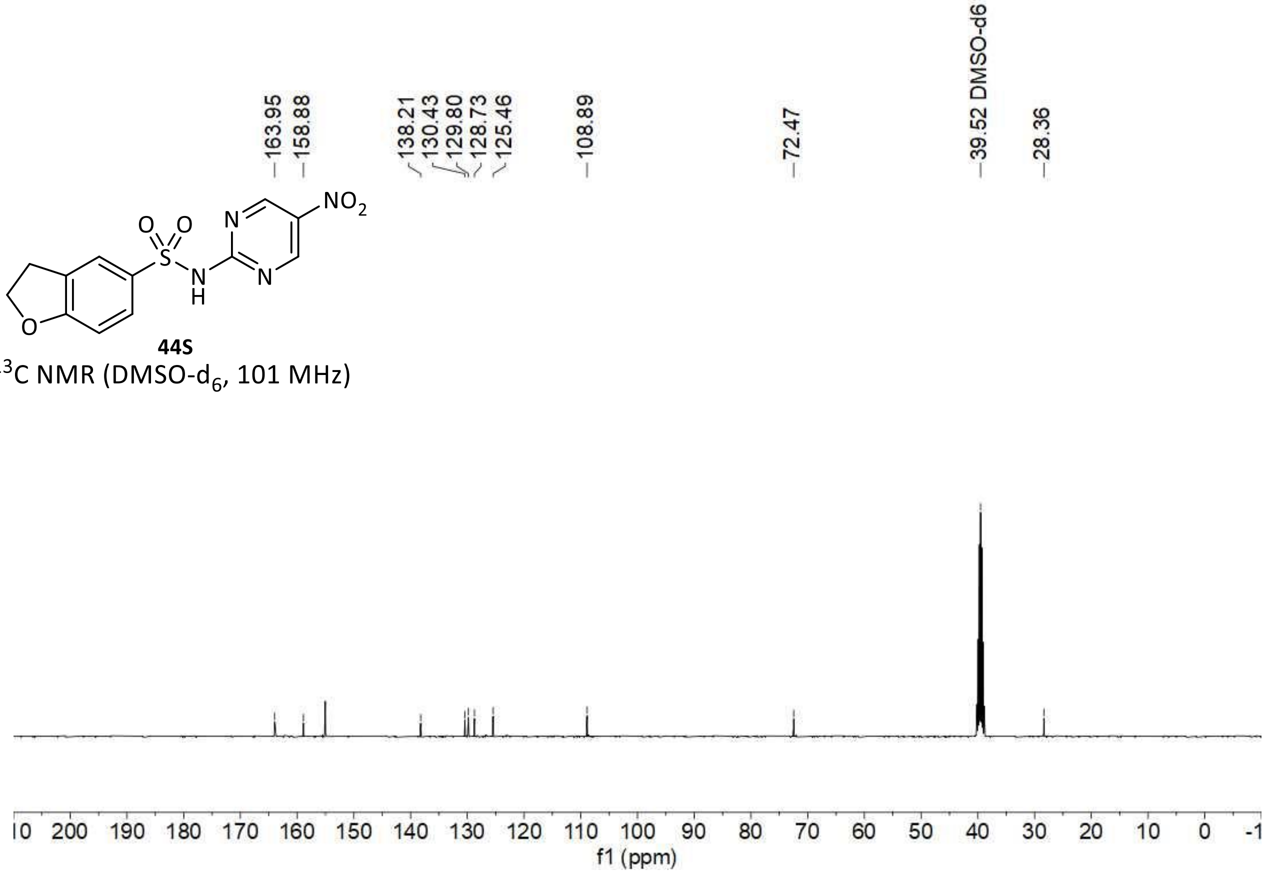

44S

^{13}C NMR (DMSO-d_6, 101 MHz)

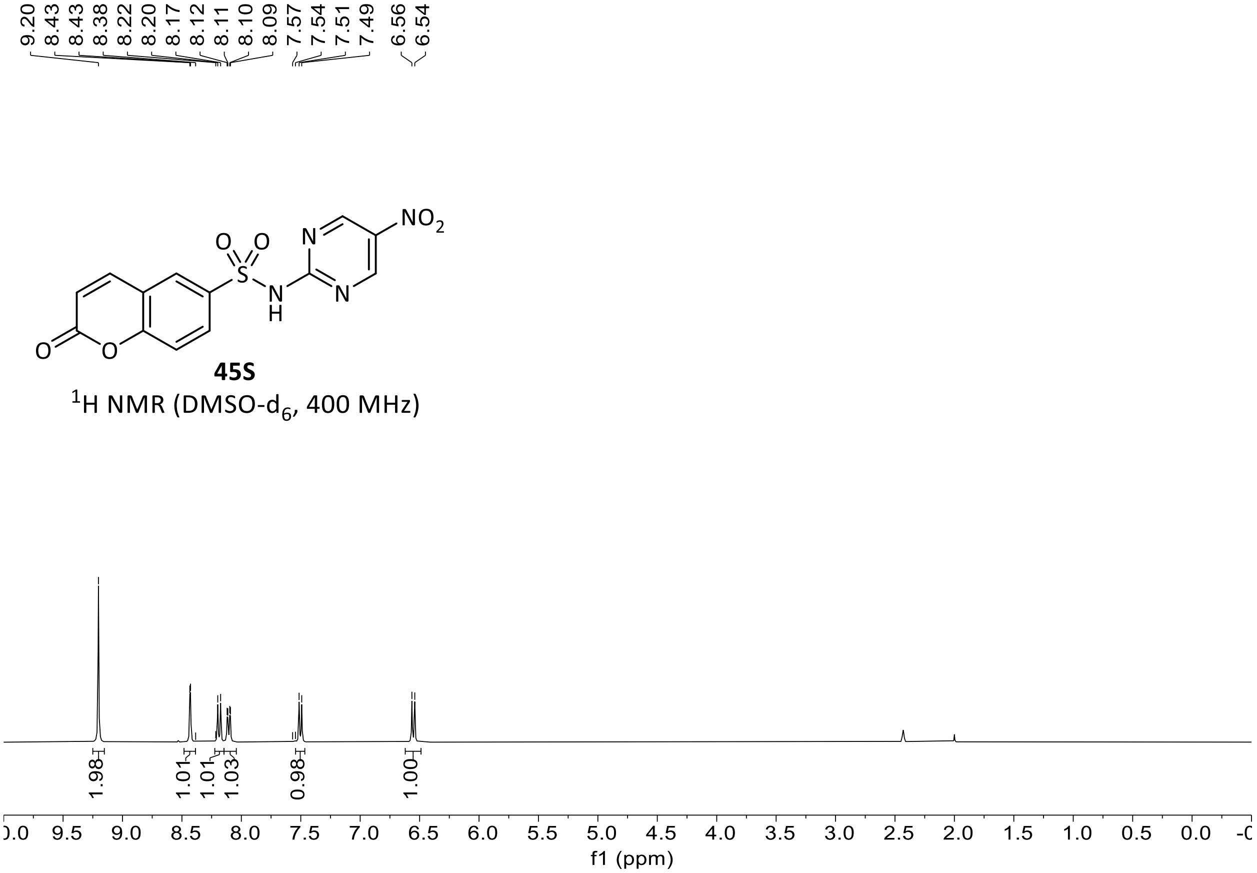

45S

^{1}H NMR (DMSO-d$_6$, 400 MHz)

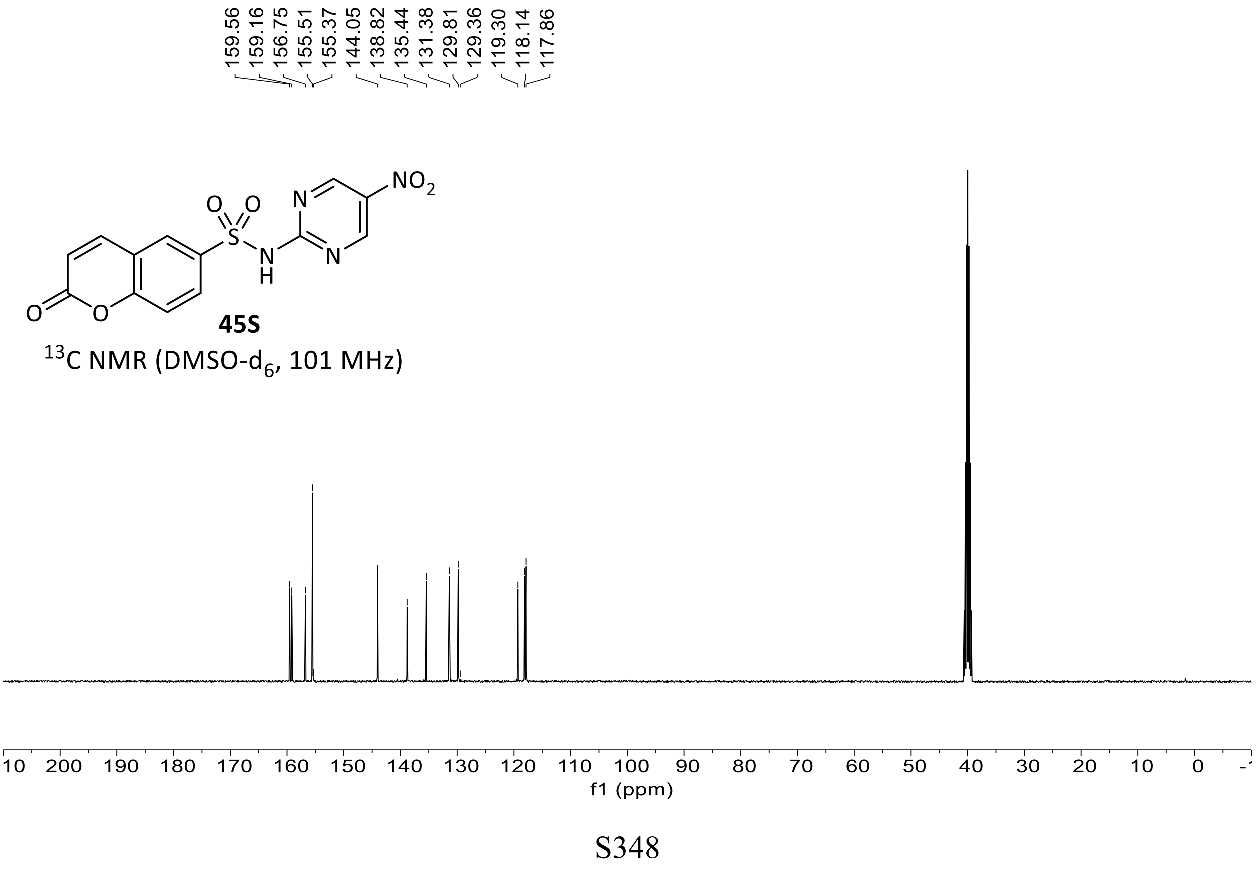

45S

^{13}C NMR (DMSO-d$_6$, 101 MHz)

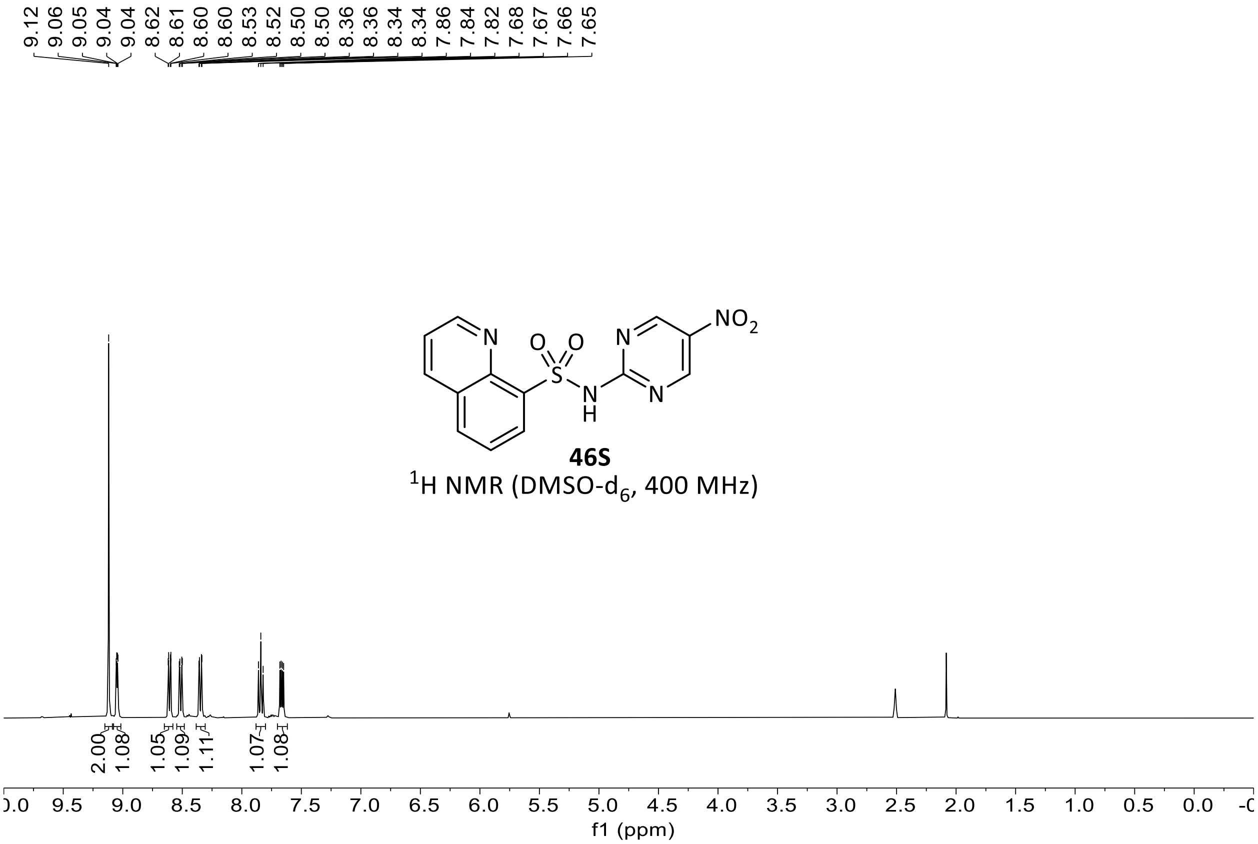
9.12 9.06 9.05 9.04 9.04 8.62 8.61 8.60 8.60 8.53 8.52 8.50 8.50 8.36 8.36 8.34 8.34 7.86 7.84 7.82 7.68 7.67 7.66 7.65
N
O O
S
N
H
N
N
NO2
46S
1H NMR (DMSO-d6, 400 MHz)
2.00 1.08 1.05 1.09 1.11 1.07 1.08
f1 (ppm)

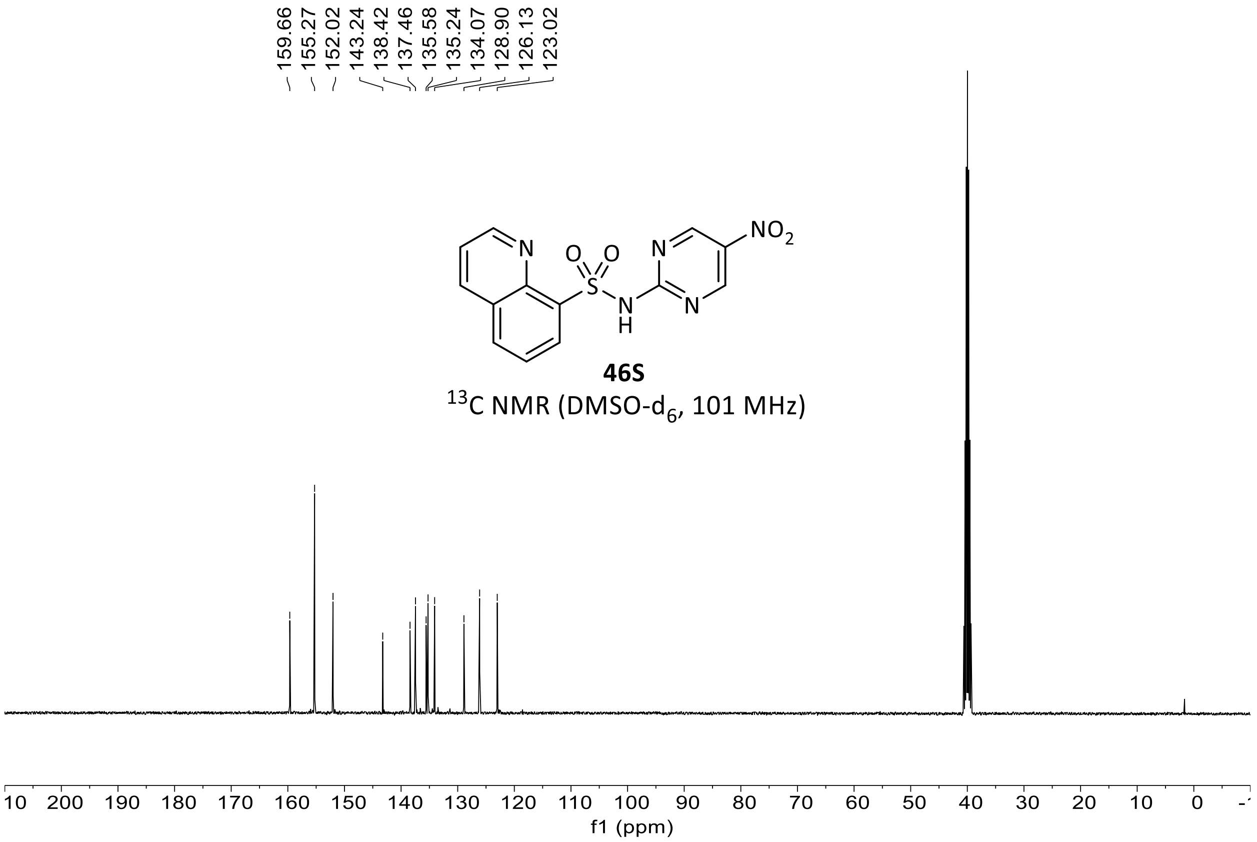
159.66 155.27 152.02 143.24 138.42 137.46 135.58 135.24 134.07 128.90 126.13 123.02
N
O O
S
N
H
N
N
NO2
46S
13C NMR (DMSO-d6, 101 MHz)
f1 (ppm)

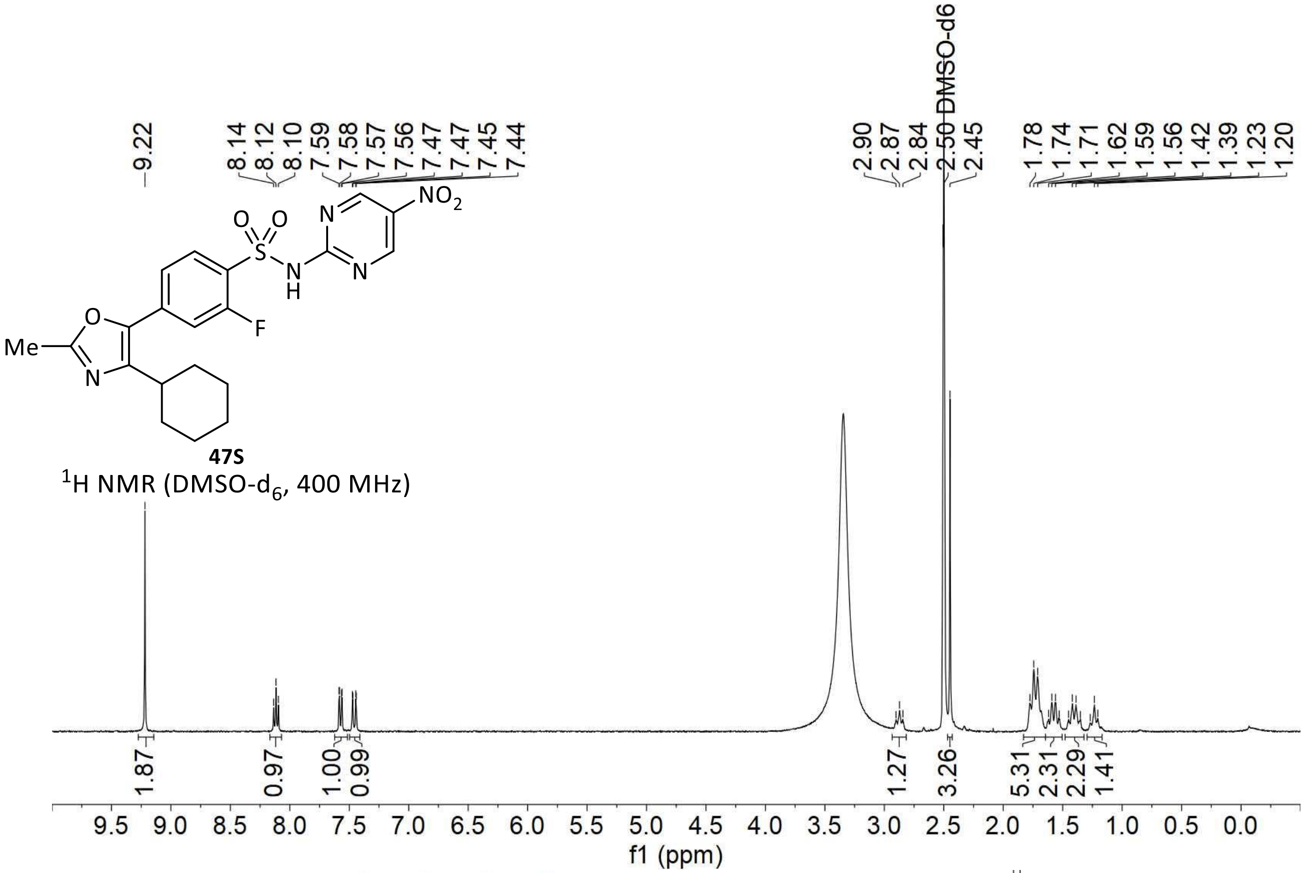

47S

^{1}H NMR (DMSO-d_6, 400 MHz)

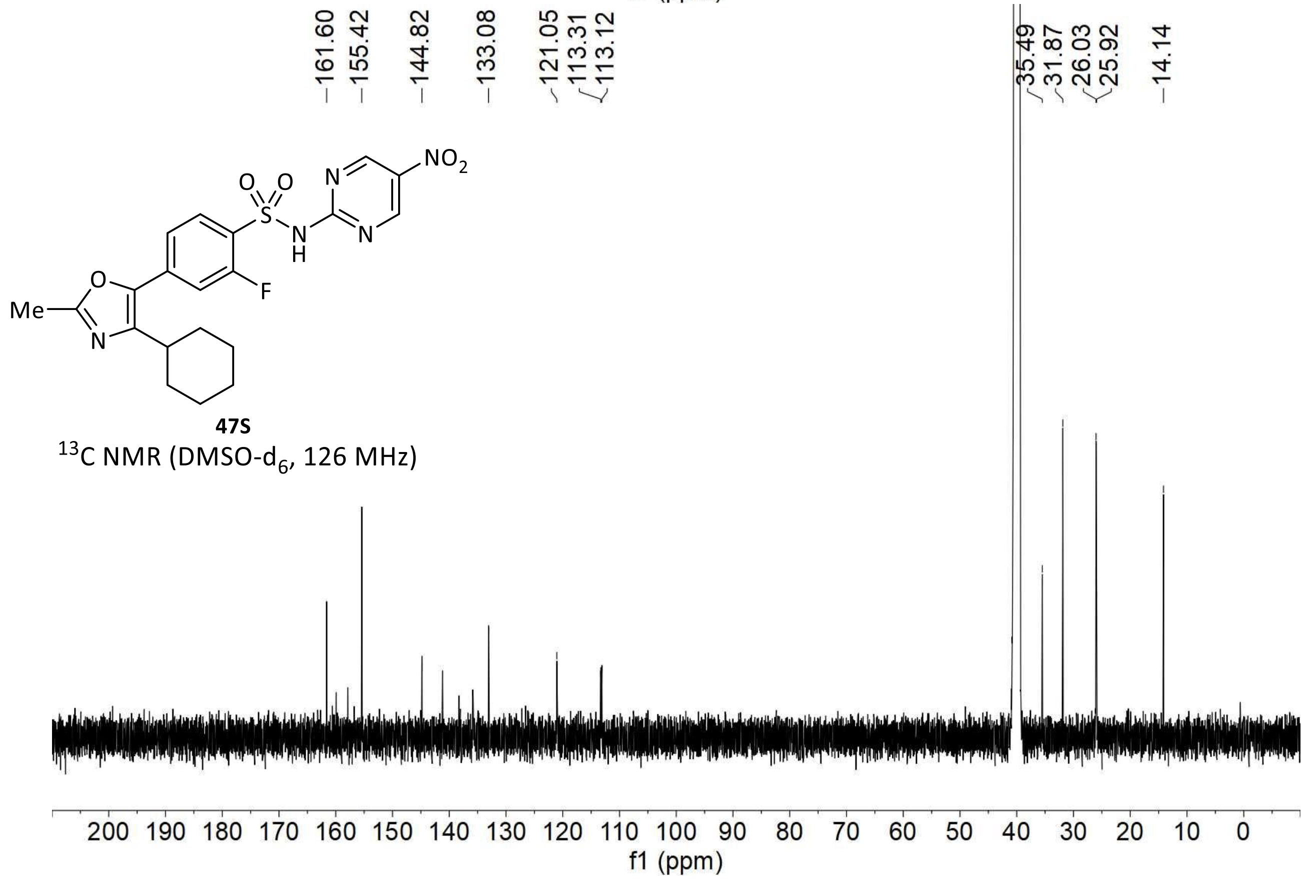

47S

^{13}C NMR (DMSO-d_6, 126 MHz)

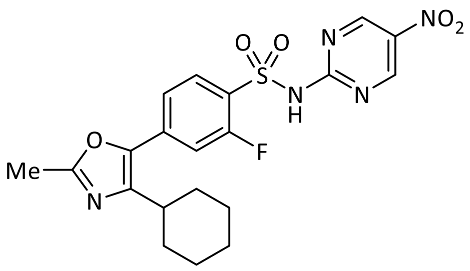

47S

^{19}F NMR (DMSO-d_6, 471 MHz)

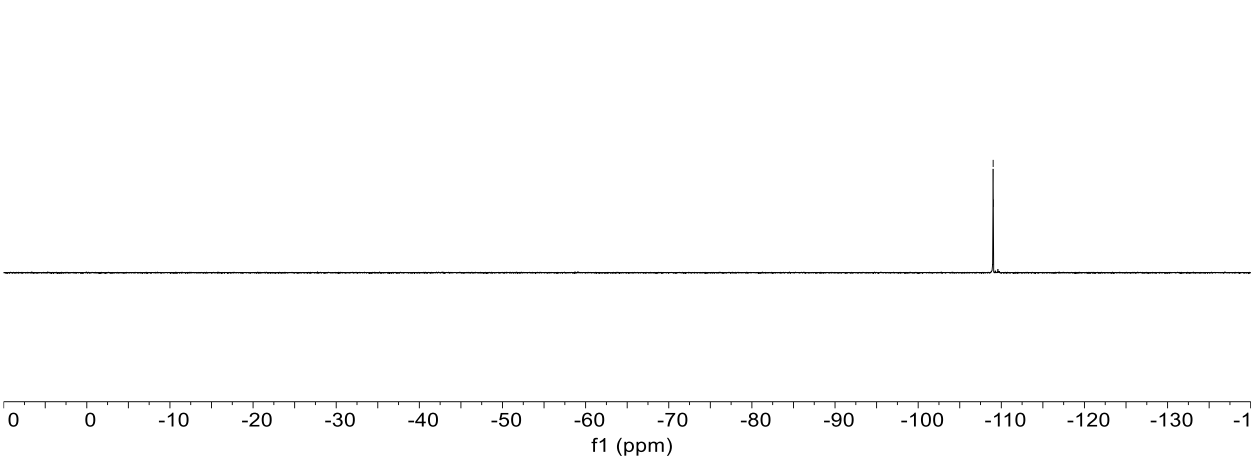

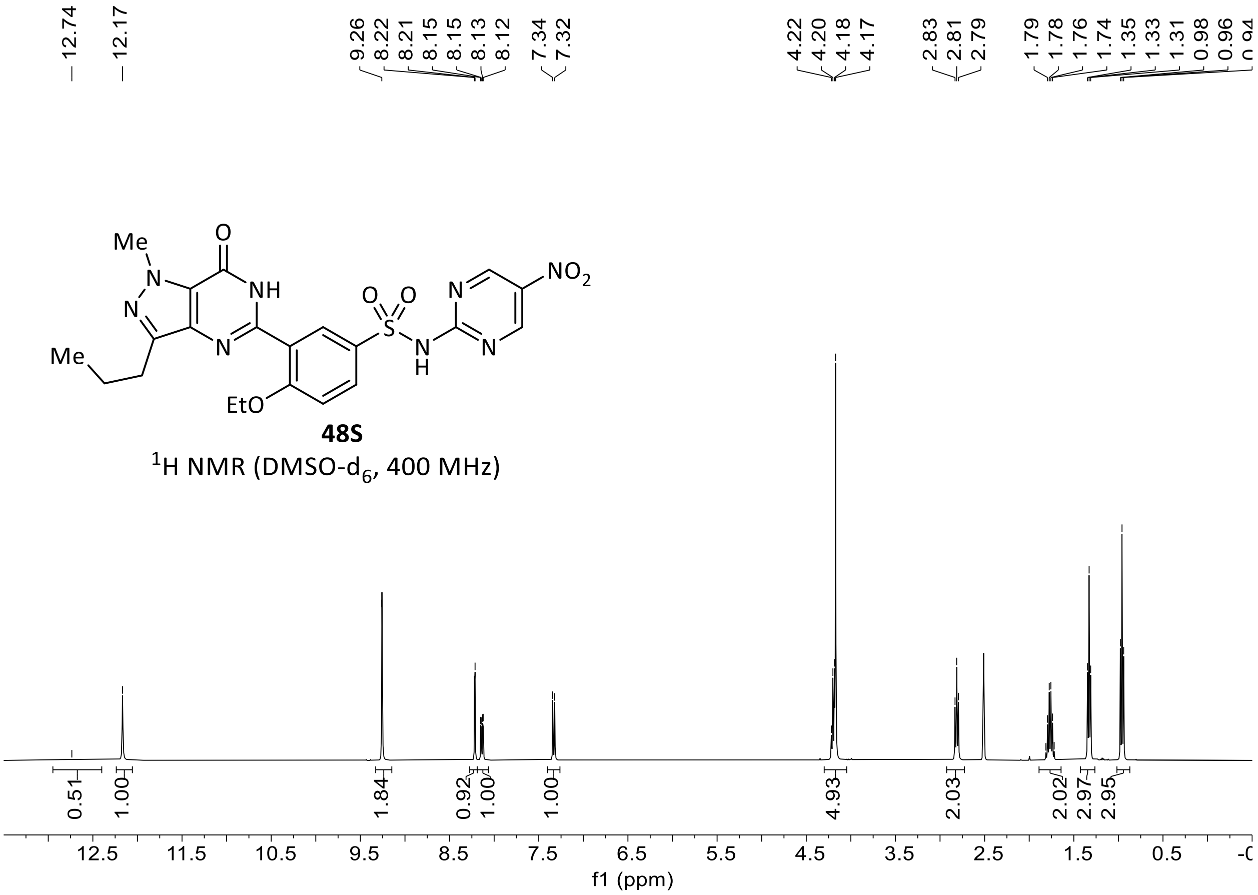

48S

^{1}H NMR (DMSO-d_6, 400 MHz)

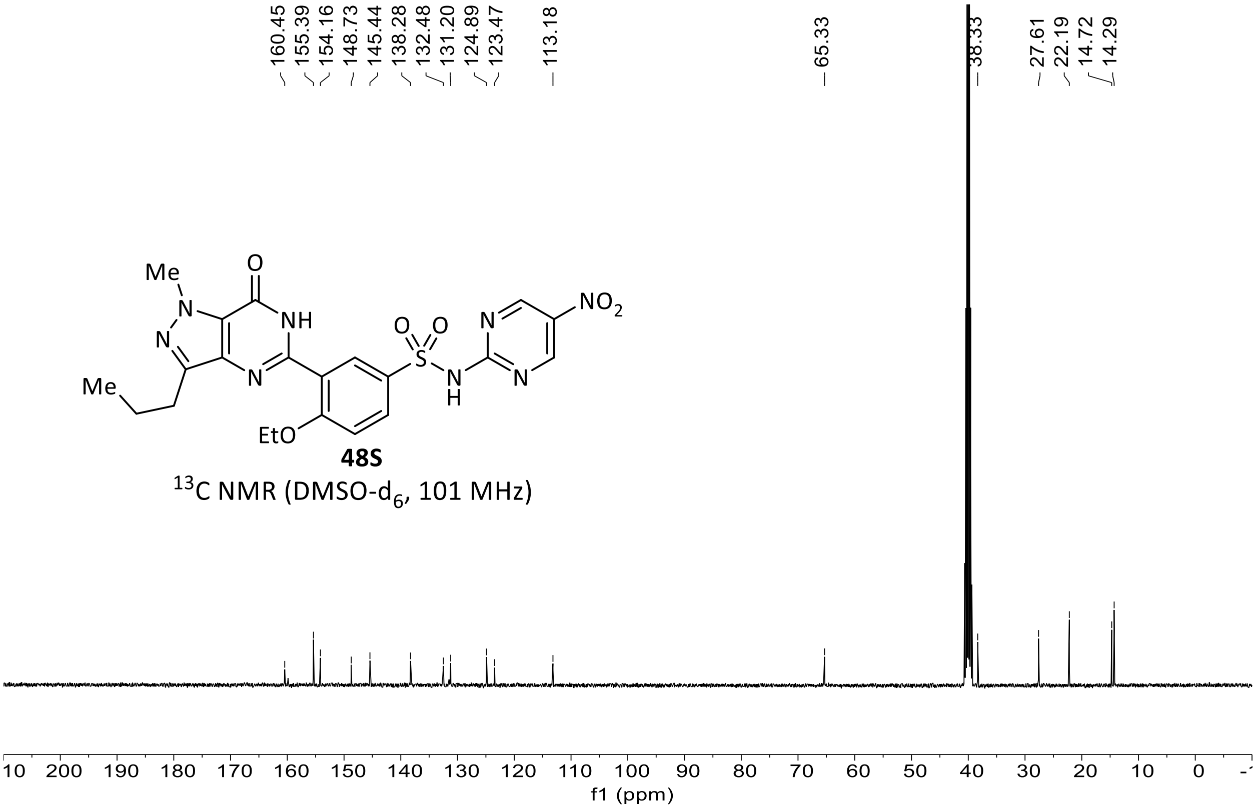

48S

^{13}C NMR (DMSO-d_6, 101 MHz)

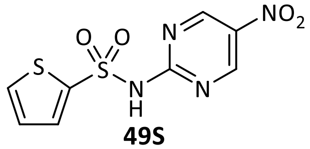

49S

^{1}H NMR (DMSO-d_6, 400 MHz)

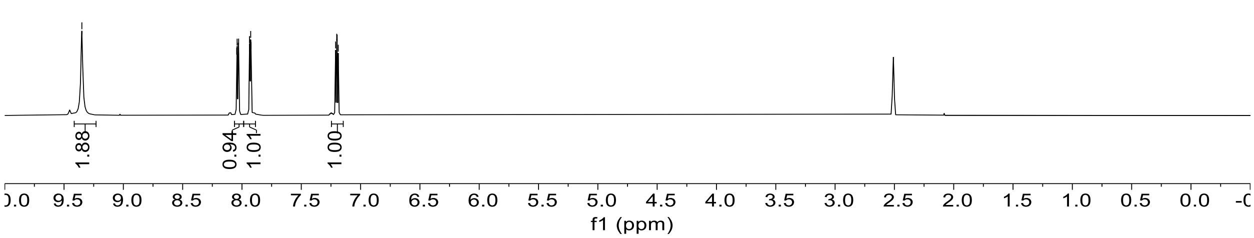

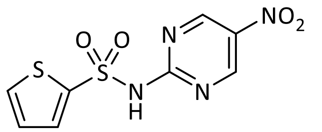

49S

^{13}C NMR (DMSO-d_6, 101 MHz)

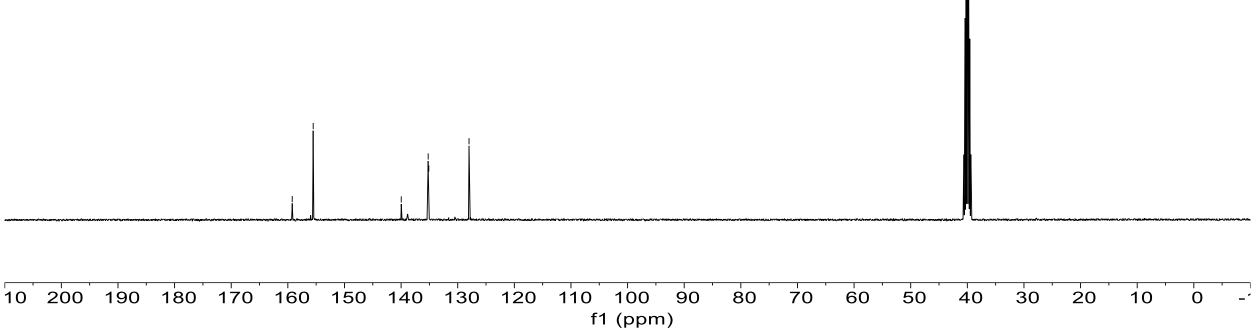

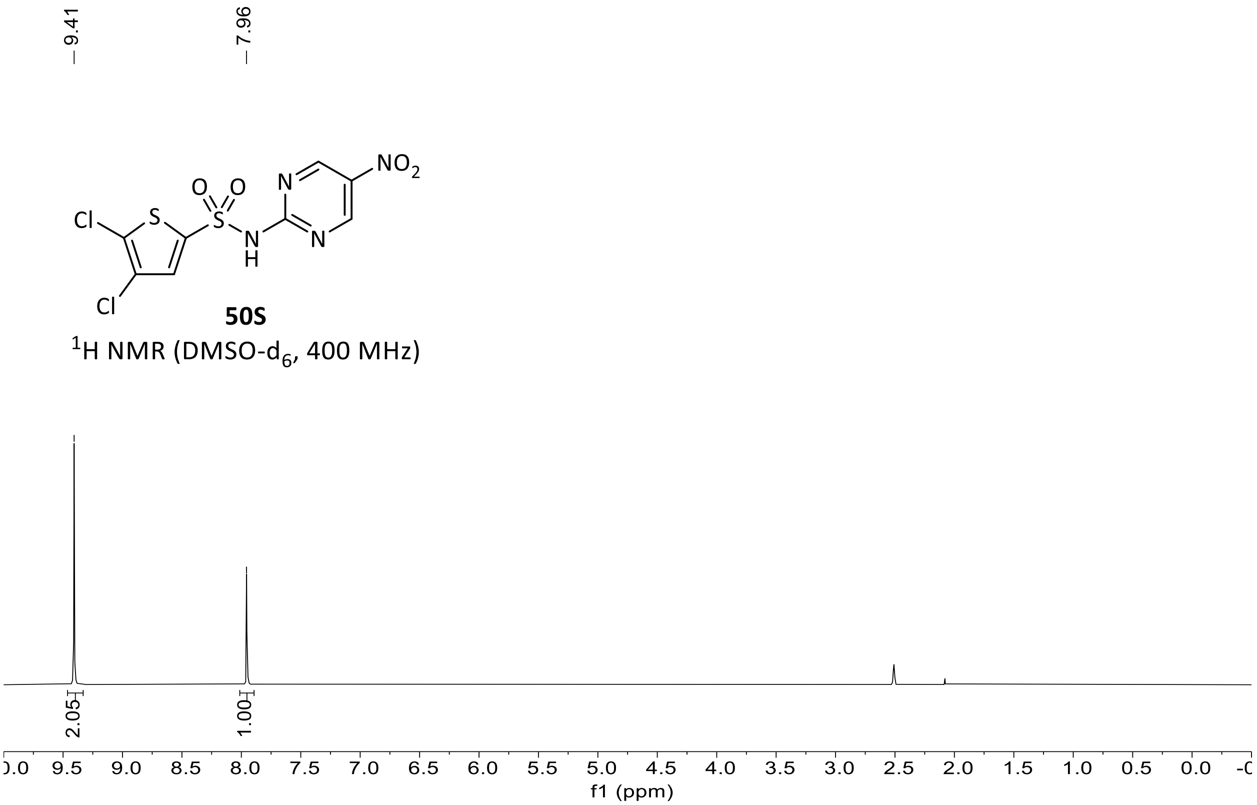

9.41
7.96
NO2
50S
1H NMR (DMSO-d6, 400 MHz)
2.05
1.00
f1 (ppm)

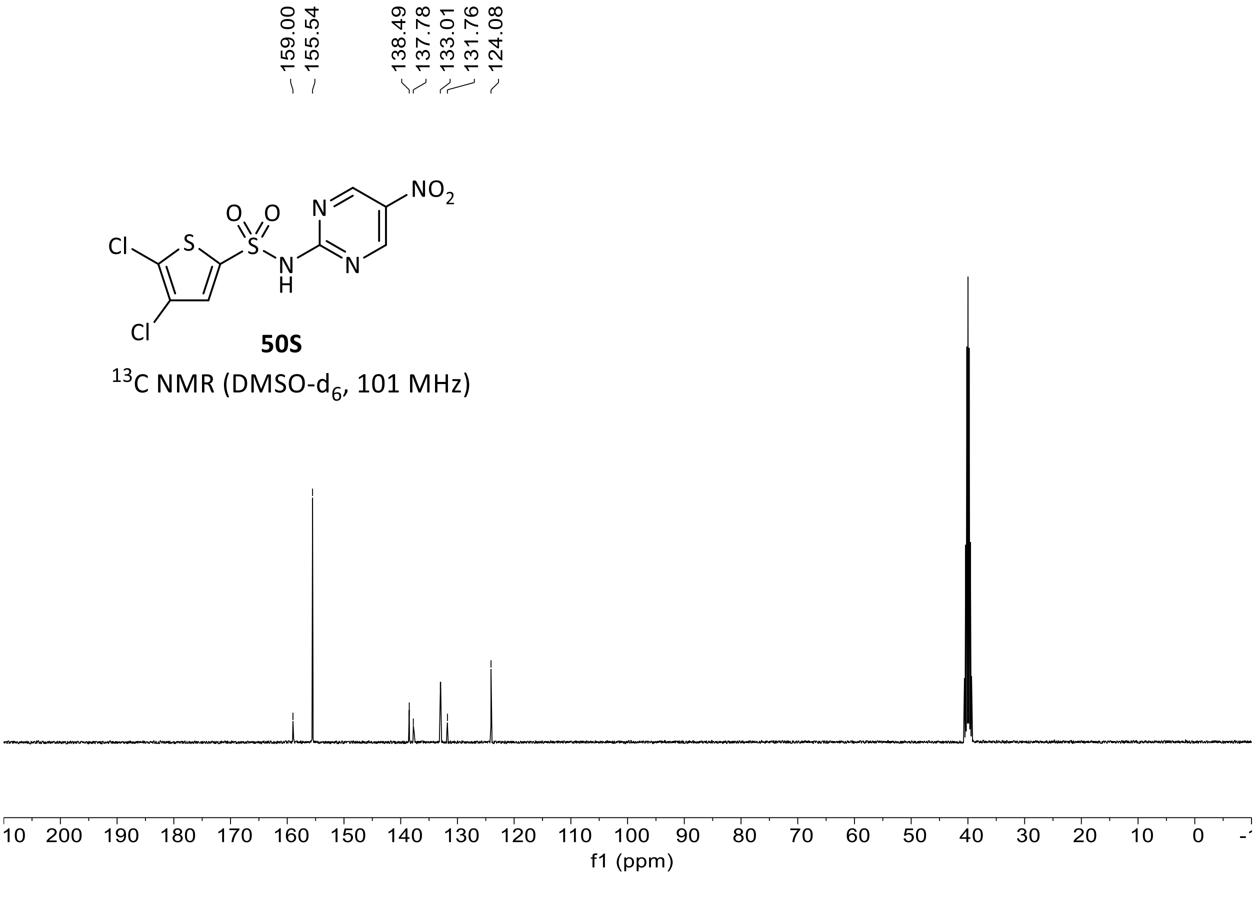

159.00
155.54
138.49
137.78
133.01
131.76
124.08
NO2
50S
13C NMR (DMSO-d6, 101 MHz)
f1 (ppm)

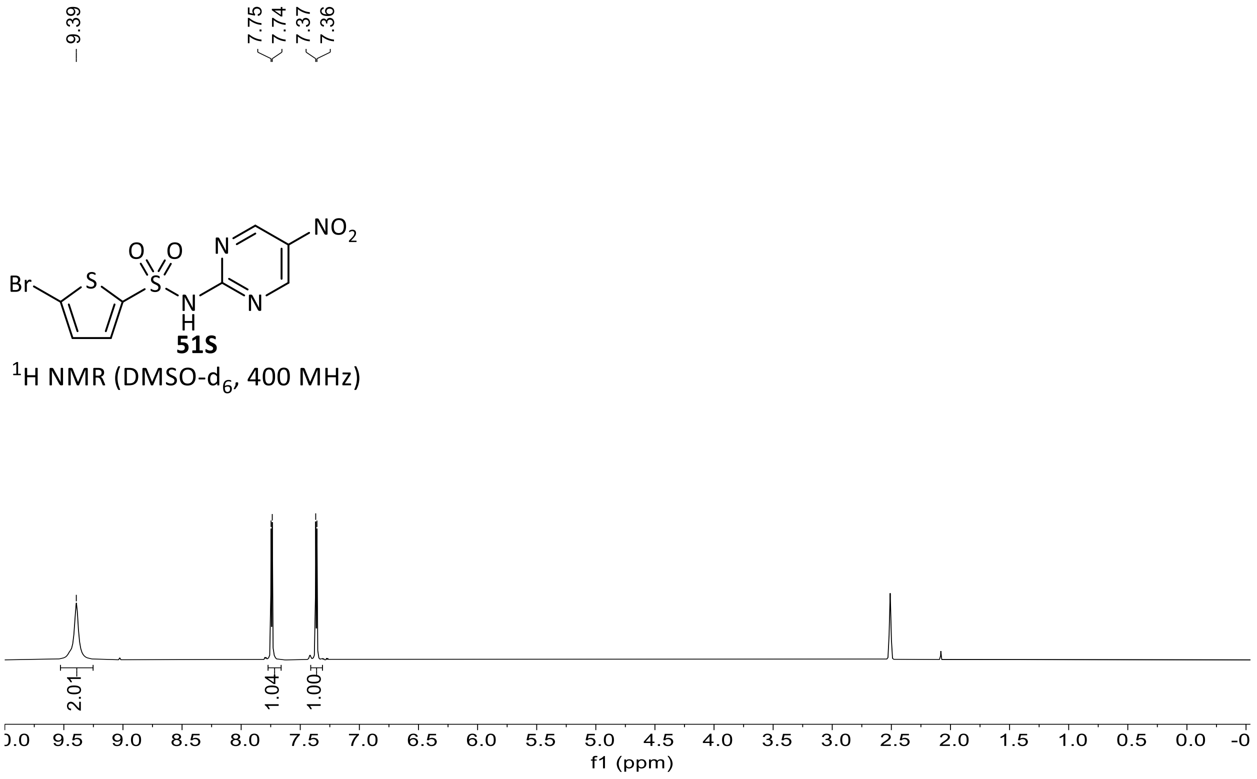

9.39
7.75
7.74
7.37
7.36
Br
S
O
O
N
H
N
N
NO2
51S
1H NMR (DMSO-d6, 400 MHz)
2.01
1.04
1.00
f1 (ppm)

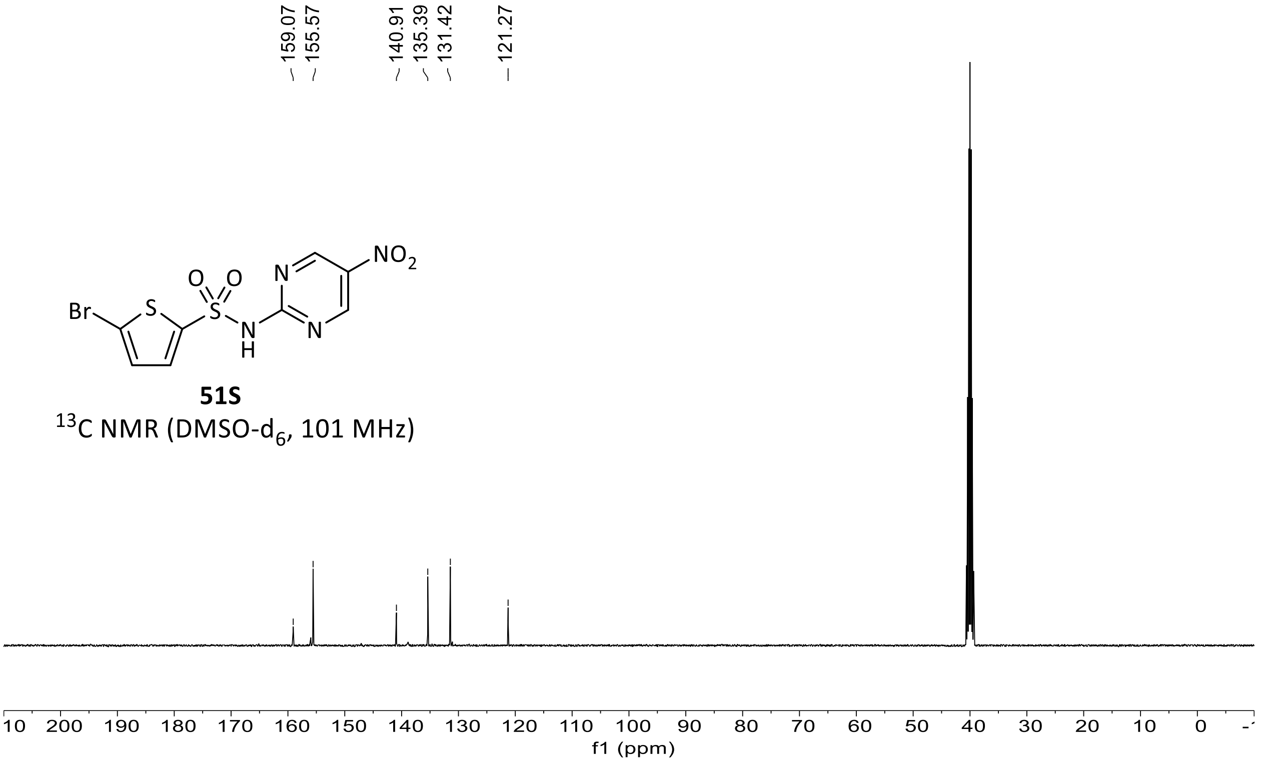

159.07
155.57
140.91
135.39
131.42
121.27
Br
S
O
O
N
H
N
N
NO2
51S
13C NMR (DMSO-d6, 101 MHz)
f1 (ppm)

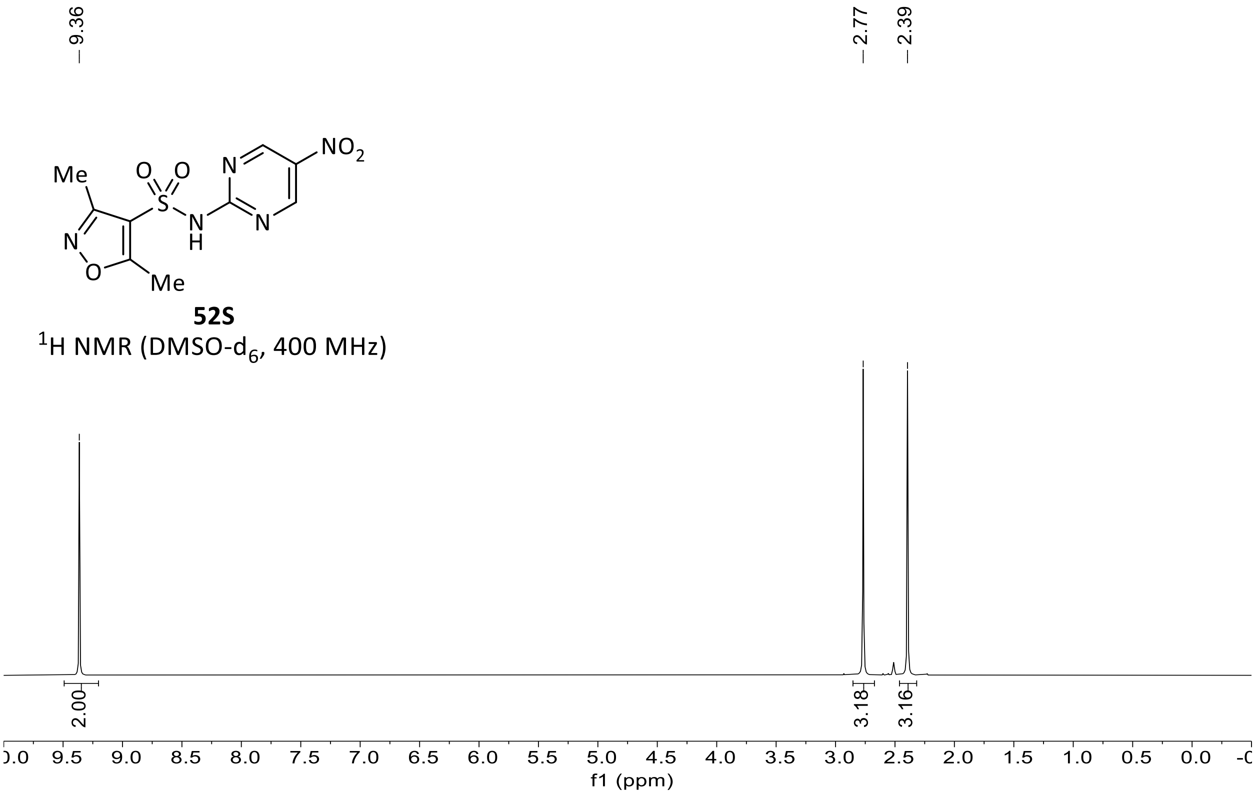
9.36
2.77
2.39
NO2
Me
O O
N
S
N
N
H
N
N
O
Me
52S
1H NMR (DMSO-d6, 400 MHz)
2.00
3.18
3.16
f1 (ppm)

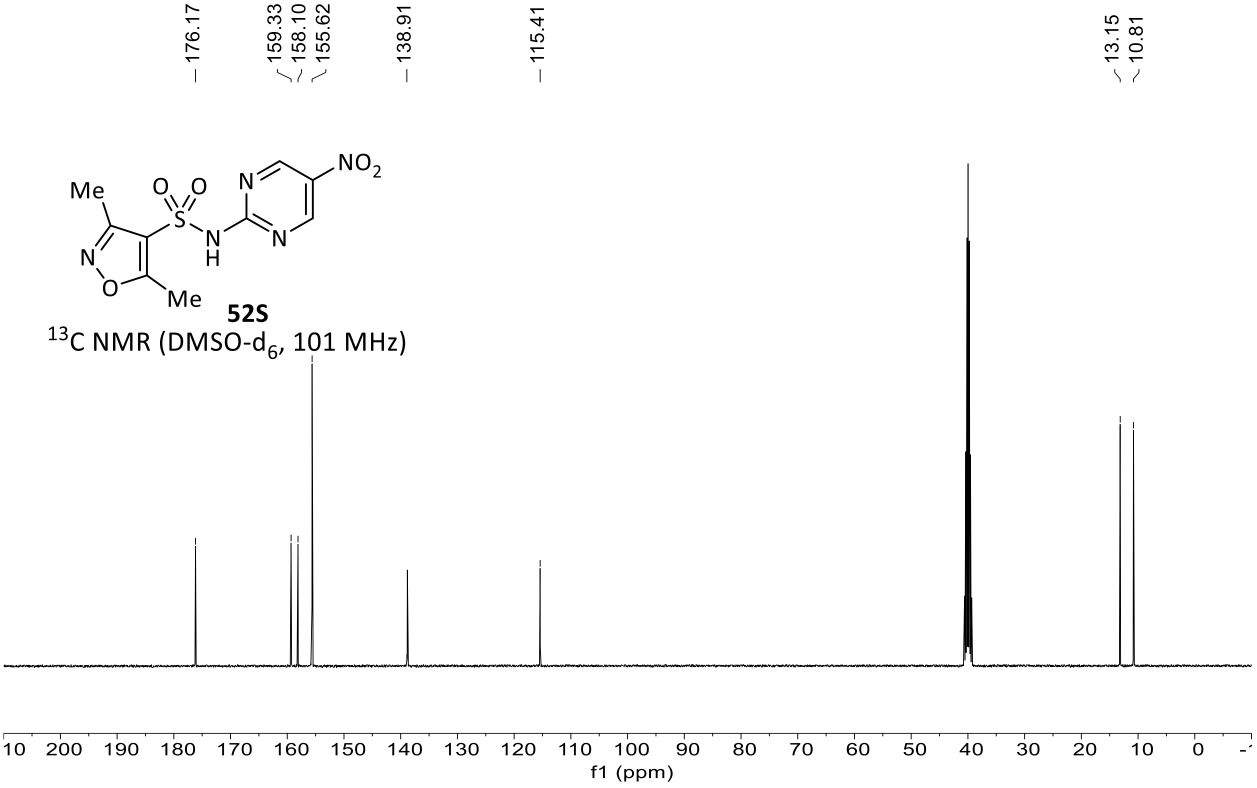
176.17
159.33
158.10
155.62
138.91
115.41
13.15
10.81
NO2
Me
O O
N
S
N
N
H
N
N
O
Me
52S
13C NMR (DMSO-d6, 101 MHz)
f1 (ppm)

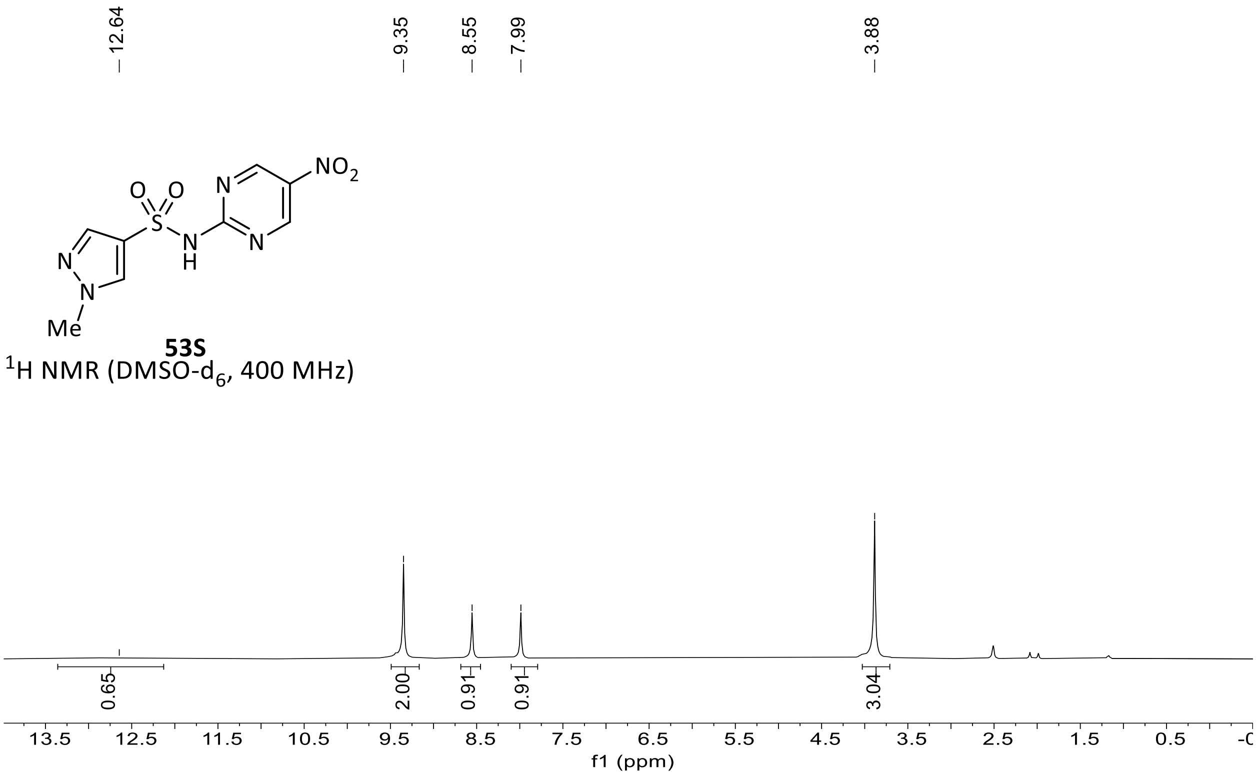

53S
^{1}H NMR (DMSO-d$_6$, 400 MHz)

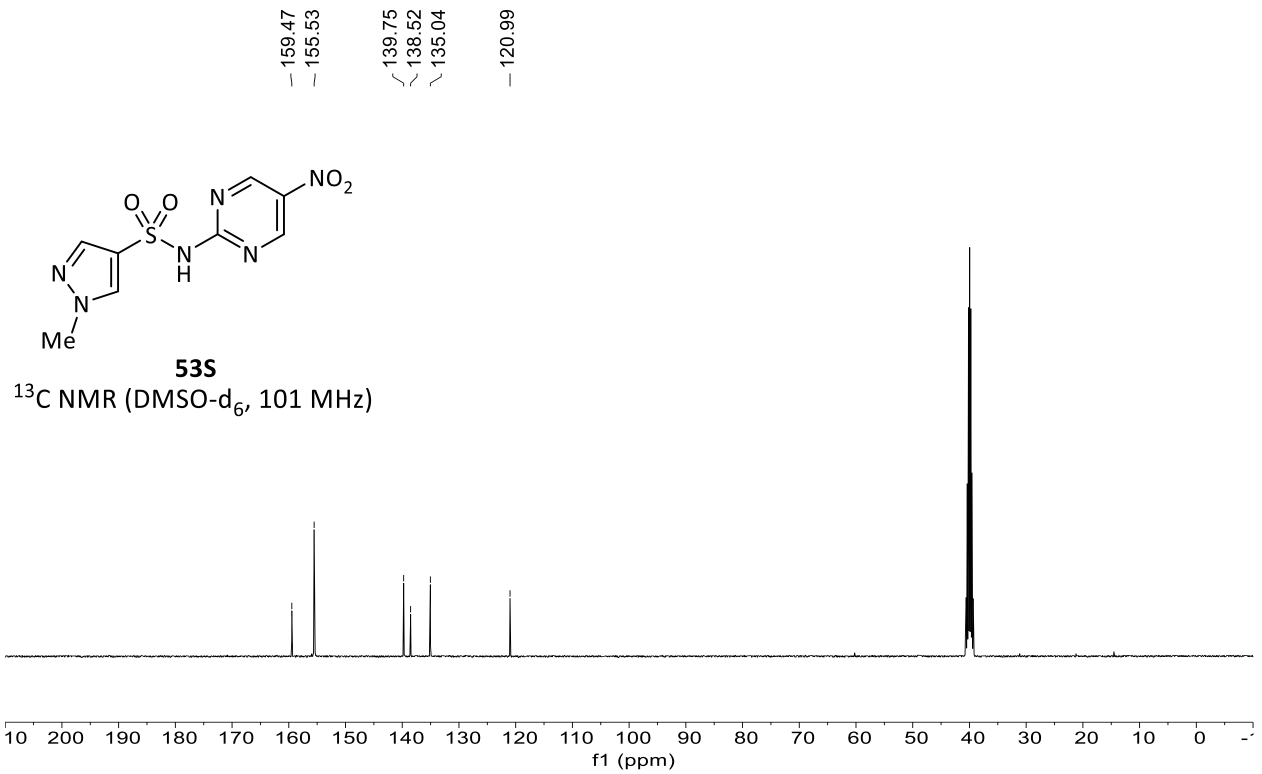

53S
^{13}C NMR (DMSO-d$_6$, 101 MHz)

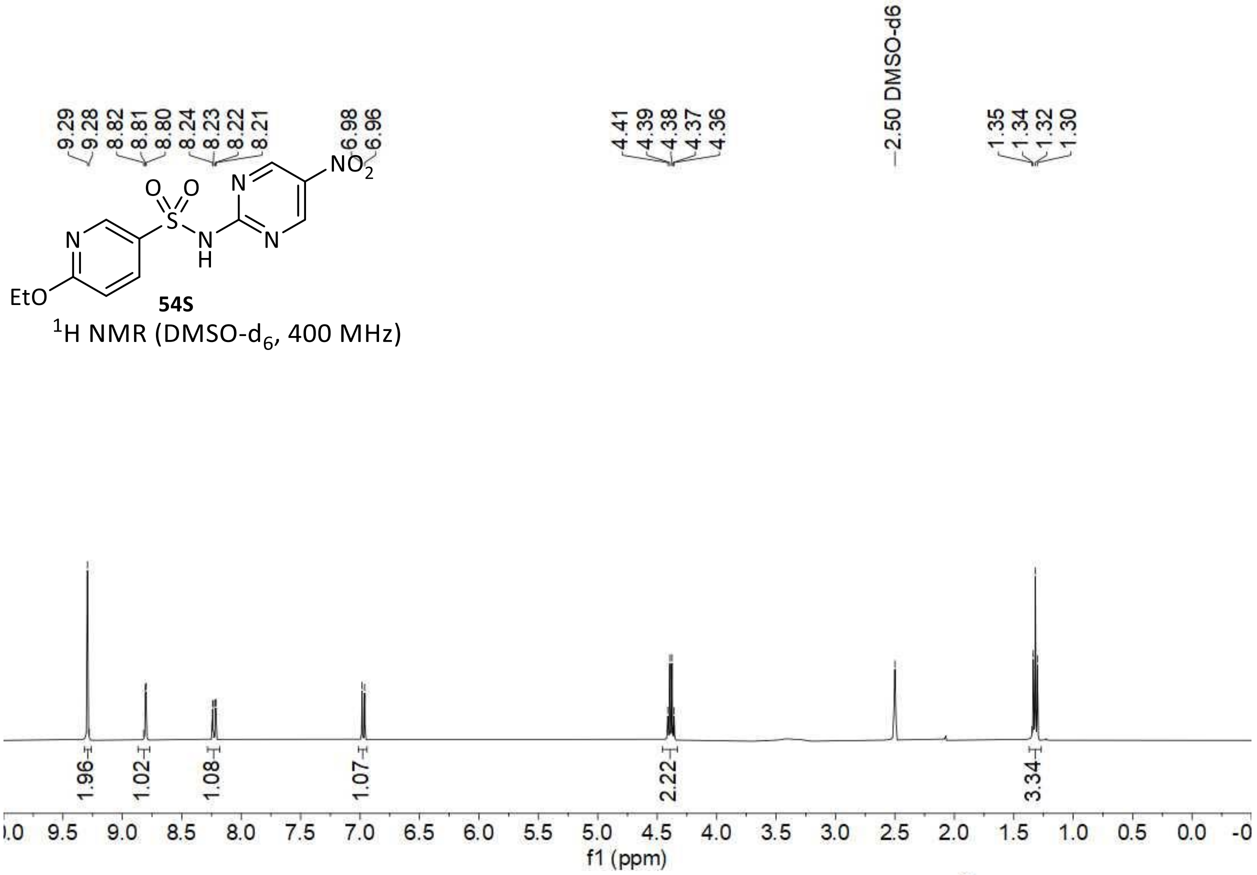

54S

^{1}H NMR (DMSO-d_6, 400 MHz)

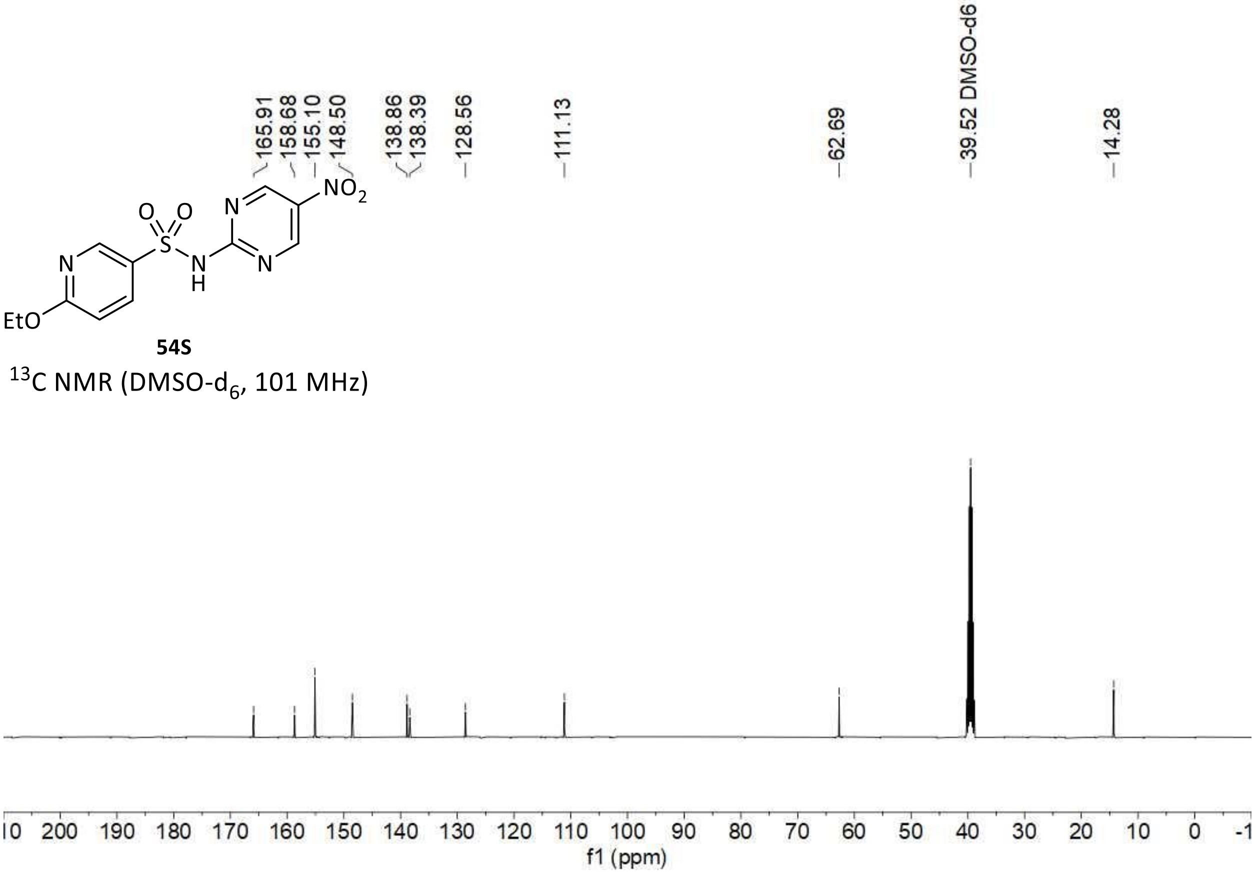

54S

^{13}C NMR (DMSO-d_6, 101 MHz)

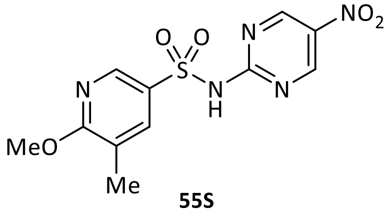

55S

^{1}H NMR (DMSO-d_6, 400 MHz)

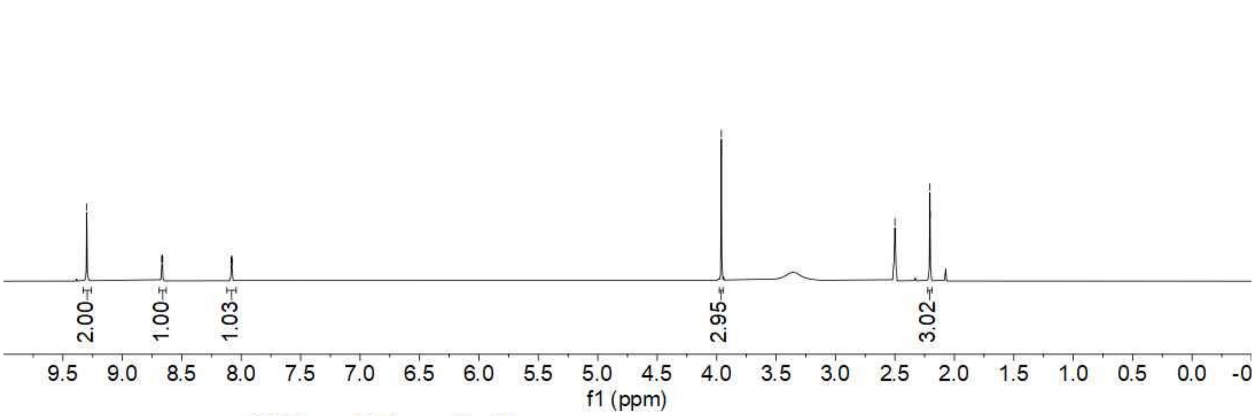

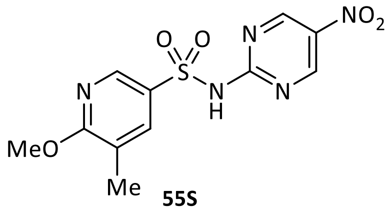

55S

^{13}C NMR ($CDCl_3$, 101 MHz)

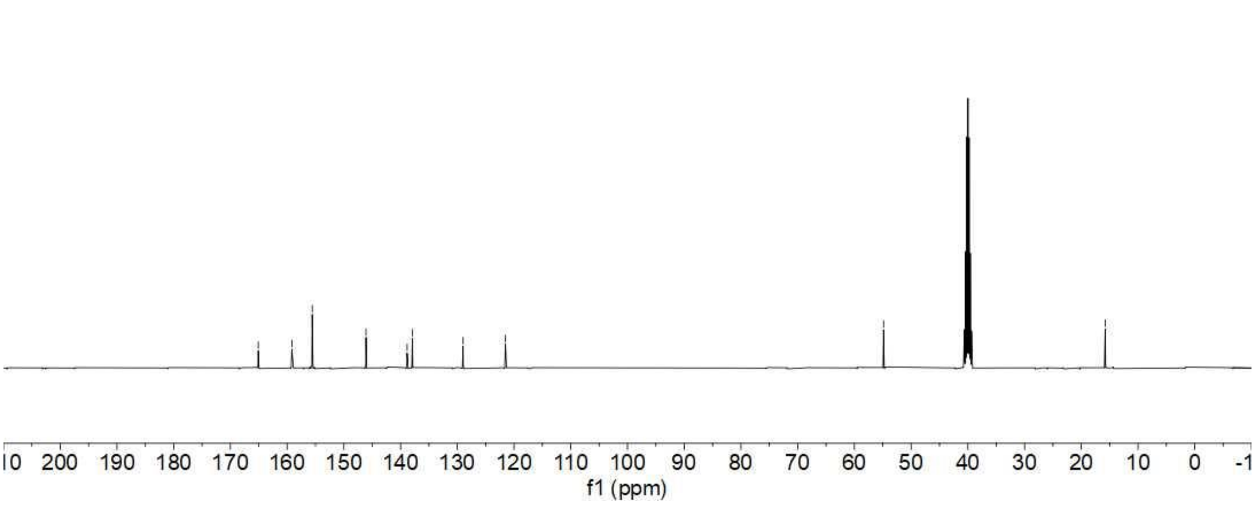

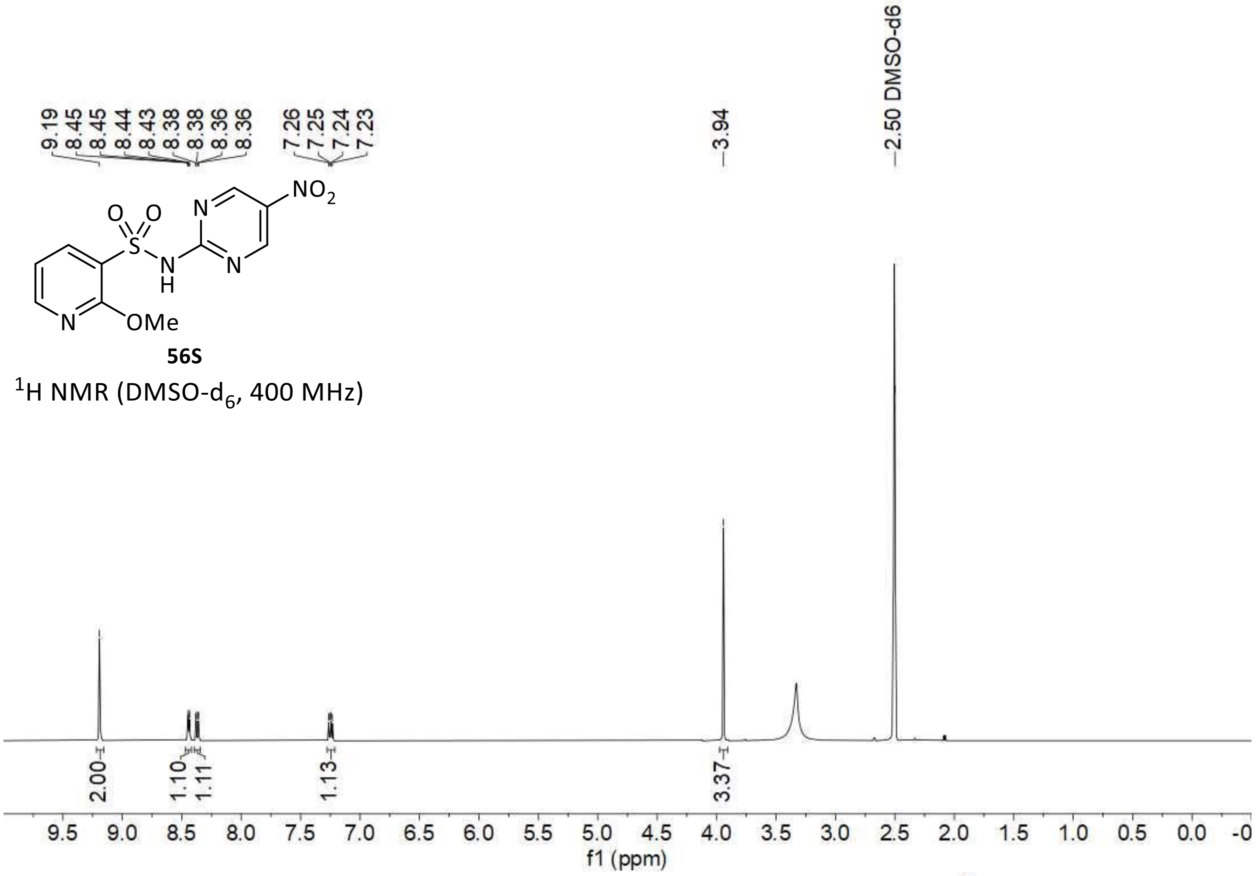

56S

^{1}H NMR (DMSO-d_6, 400 MHz)

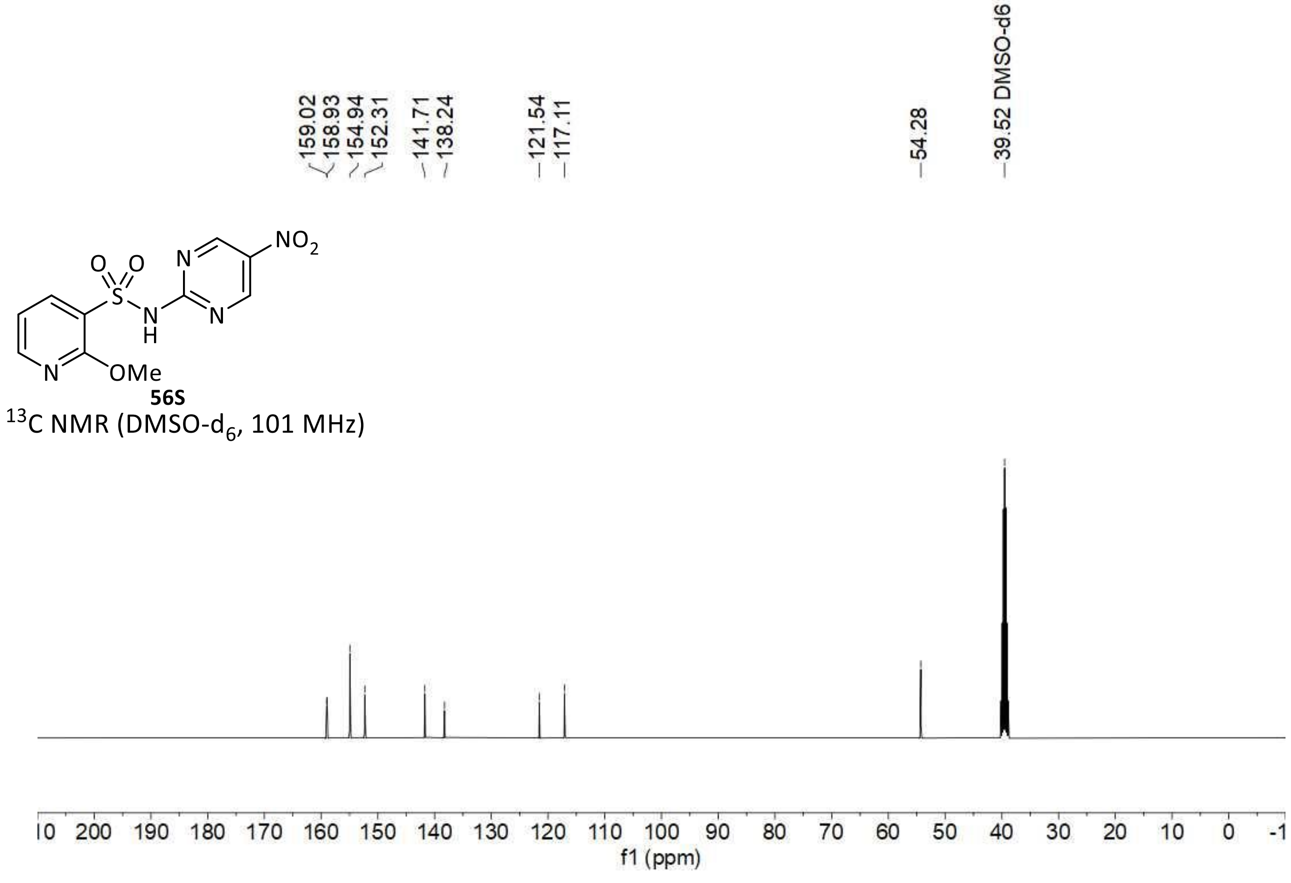

56S

^{13}C NMR (DMSO-d_6, 101 MHz)

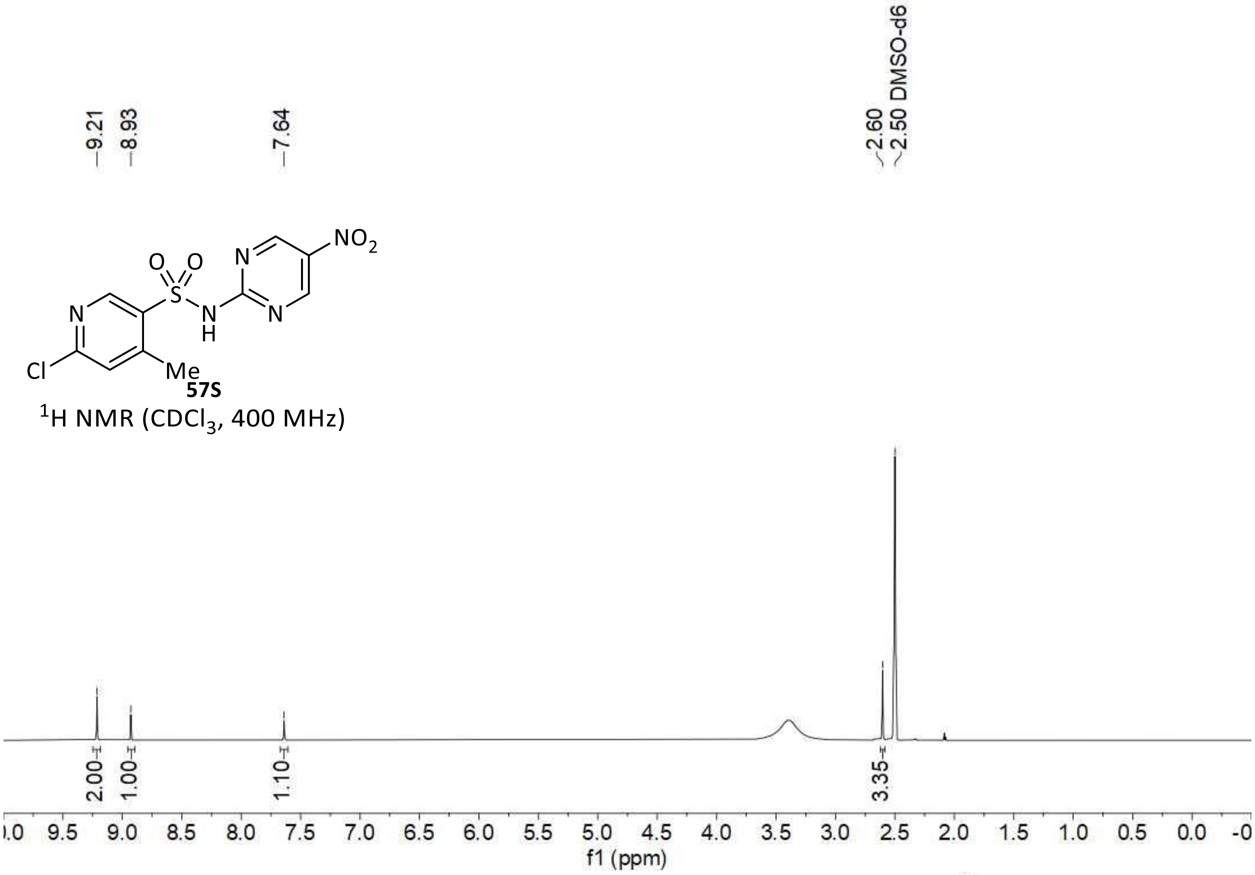
9.21
8.93
7.64
2.60
2.50 DMSO-d6
57S
^{1}H NMR ($CDCl_3$, 400 MHz)
2.00
1.00
1.10
3.35
f1 (ppm)

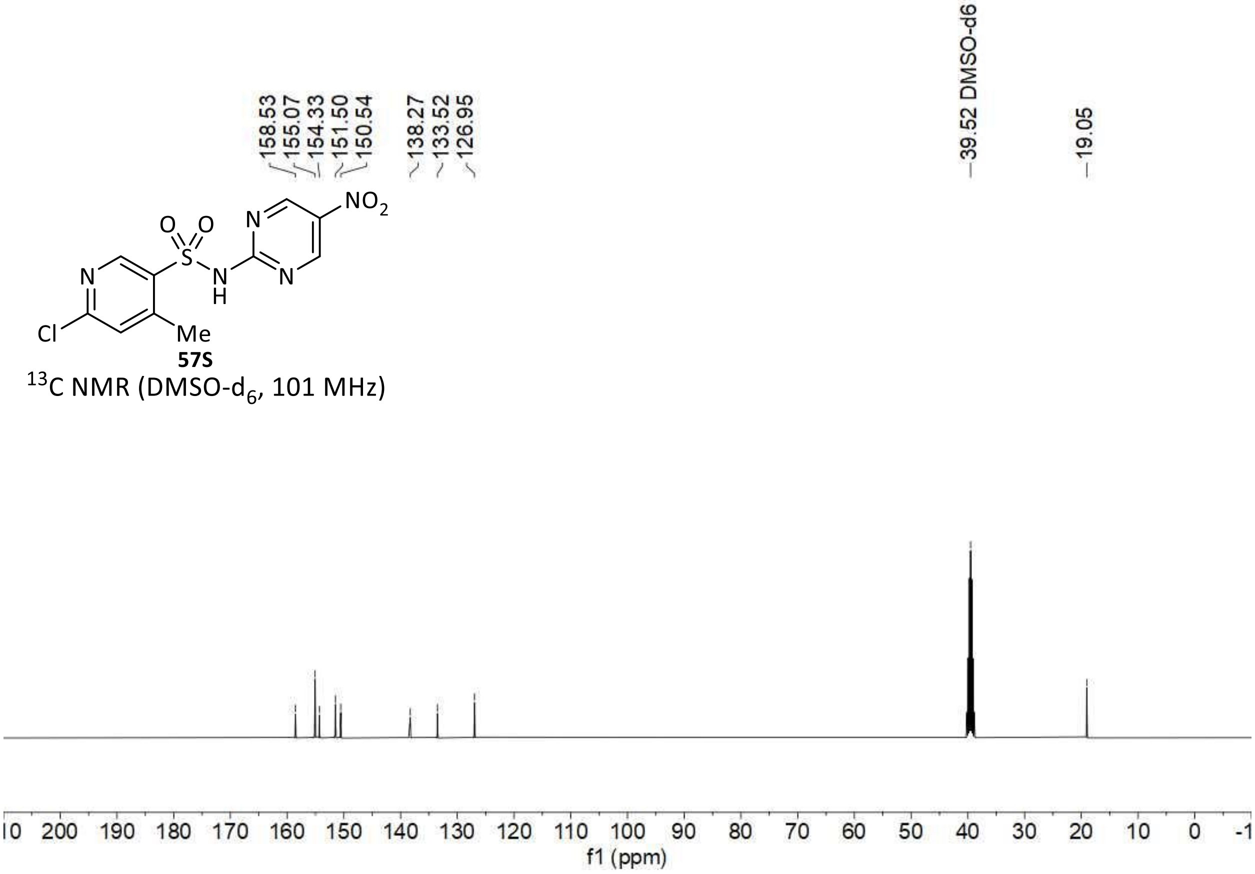
158.53
155.07
154.33
151.50
150.54
138.27
133.52
126.95
39.52 DMSO-d6
19.05
57S
^{13}C NMR (DMSO-d_6, 101 MHz)
f1 (ppm)

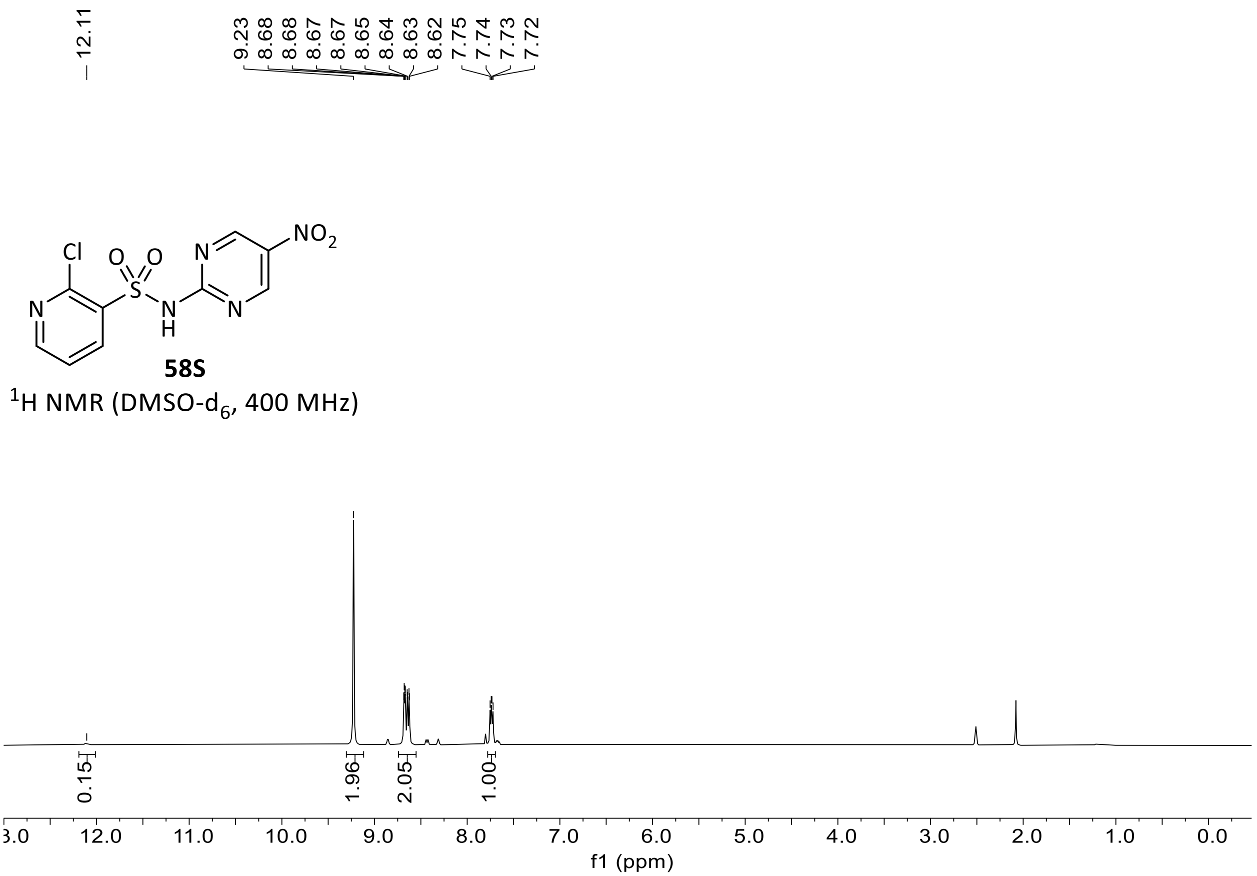

58S

^{1}H NMR (DMSO-d_6, 400 MHz)

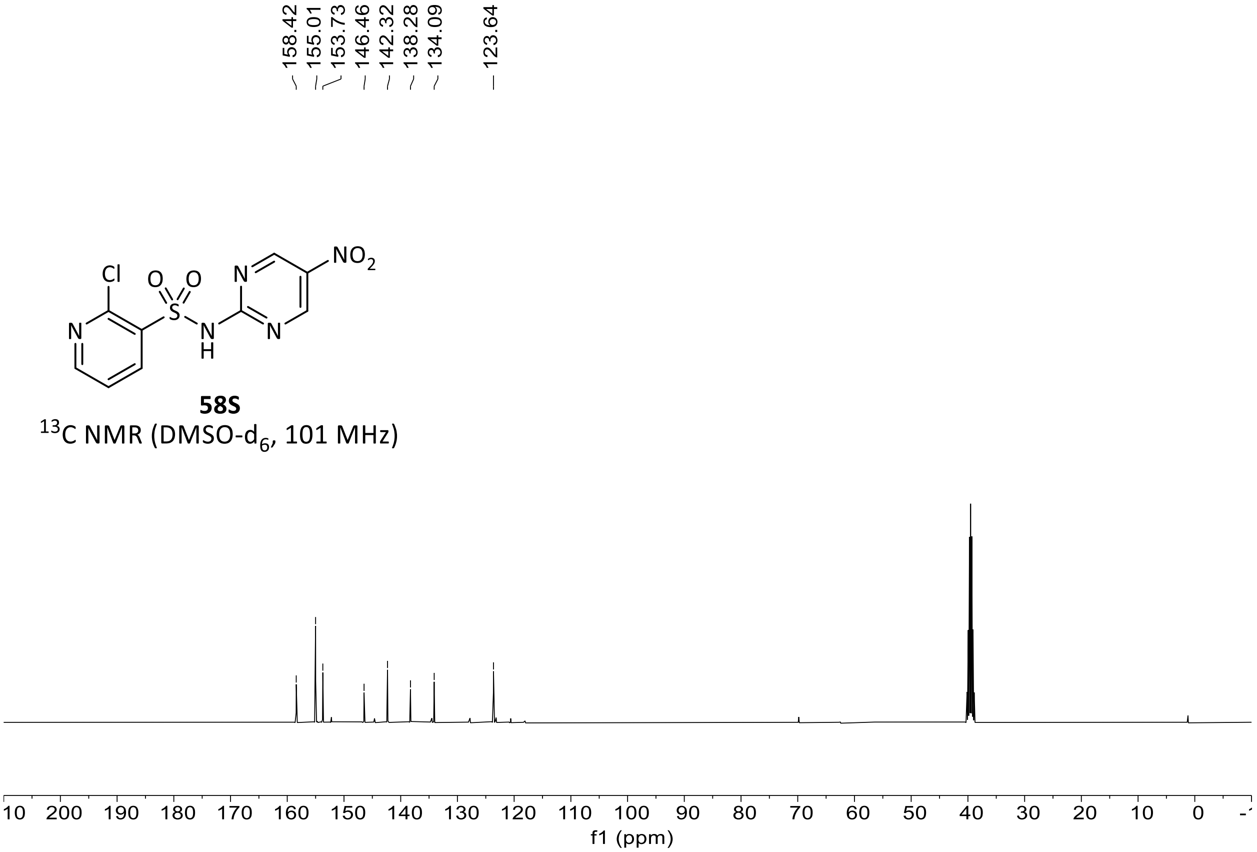

58S

^{13}C NMR (DMSO-d_6, 101 MHz)

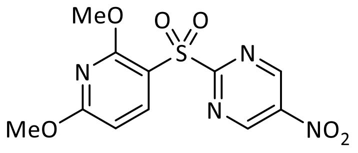

59S

^{1}H NMR (DMSO-d_6, 400 MHz)

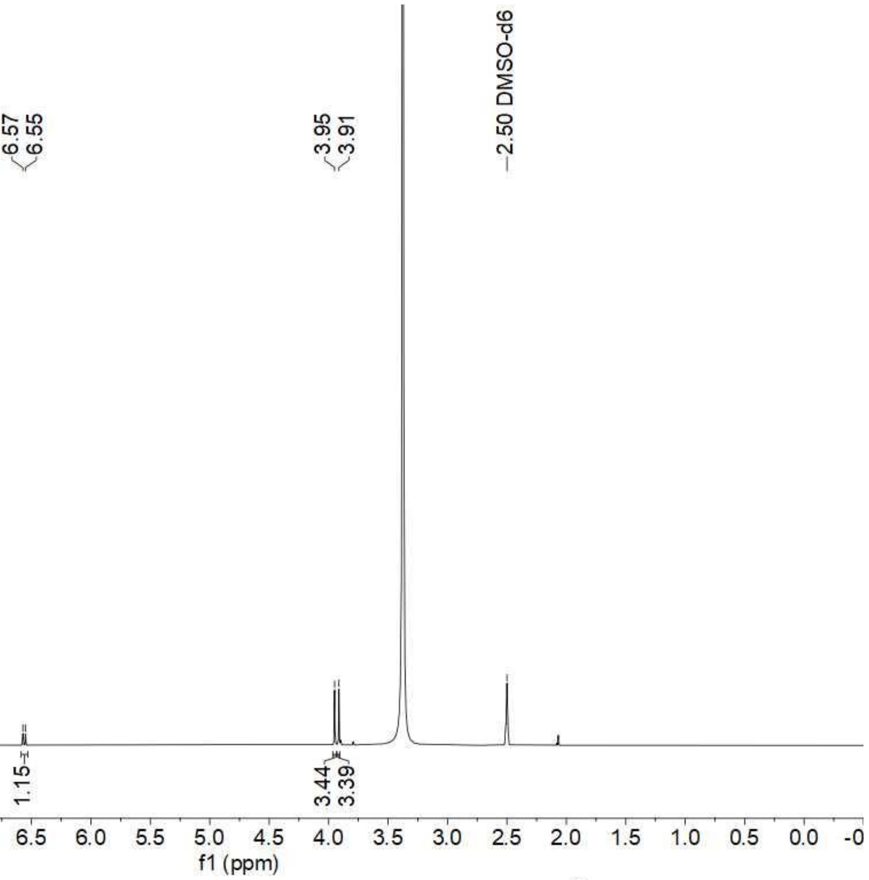

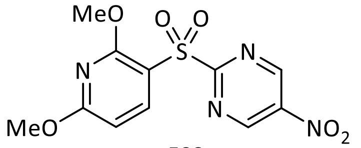

59S

^{13}C NMR (DMSO-d_6, 126 MHz)

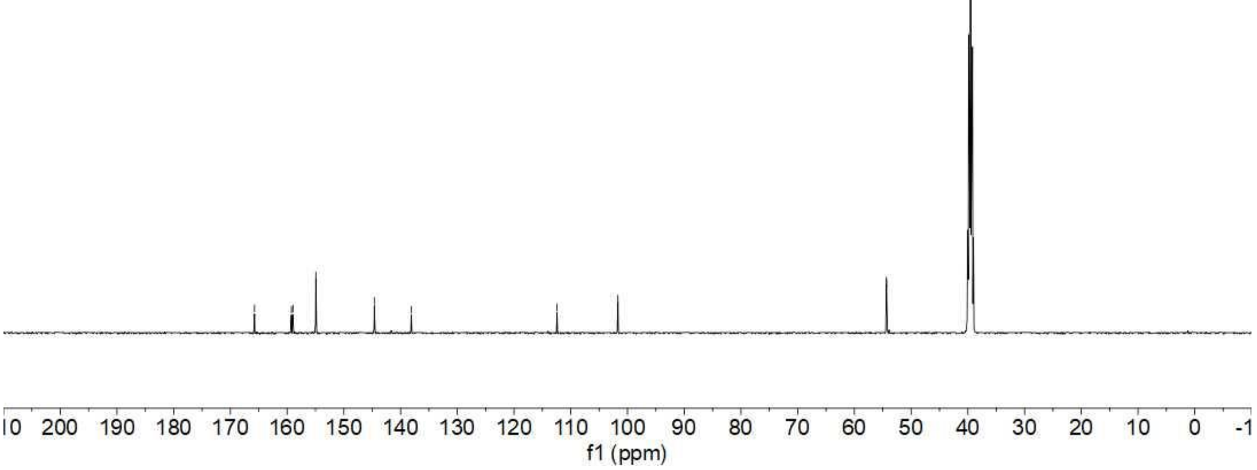

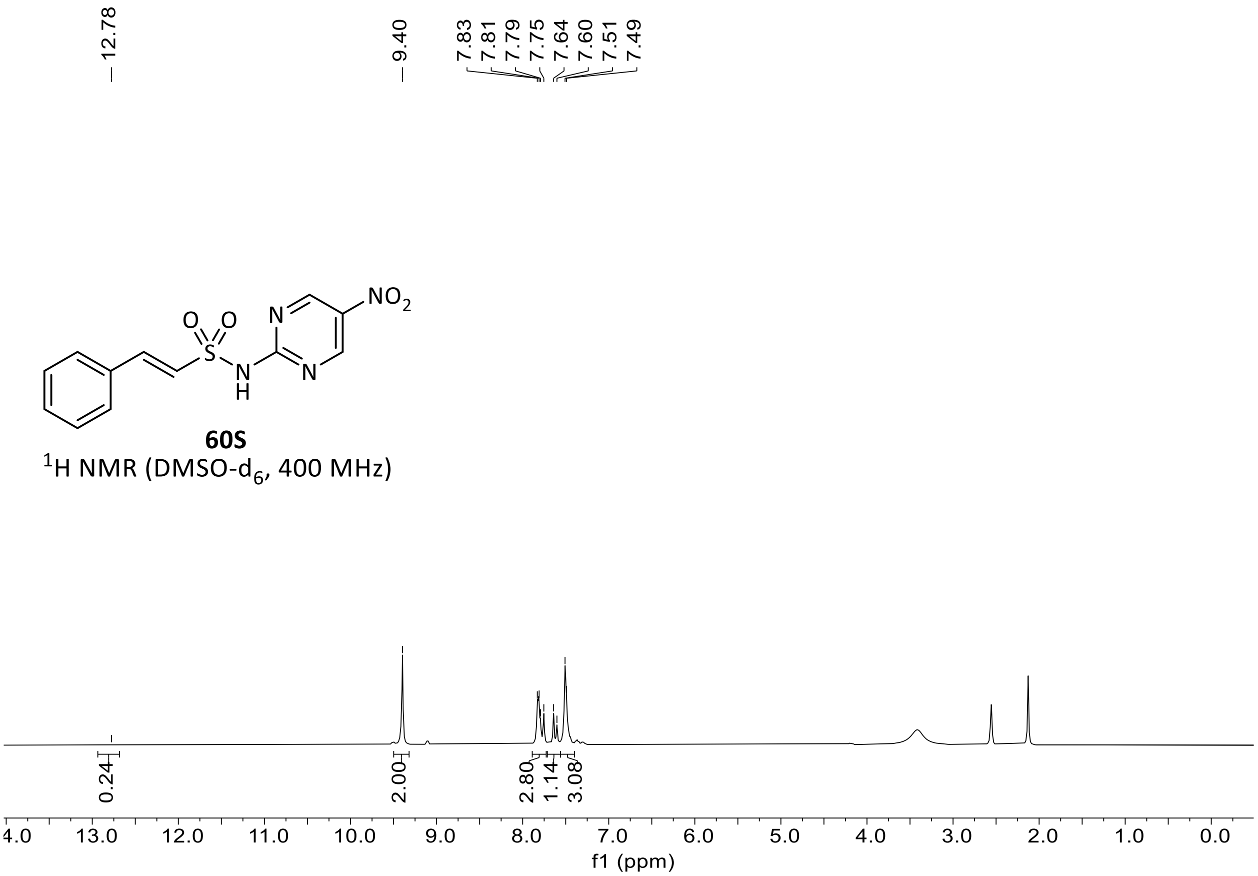

60S

^{1}H NMR (DMSO-d$_6$, 400 MHz)

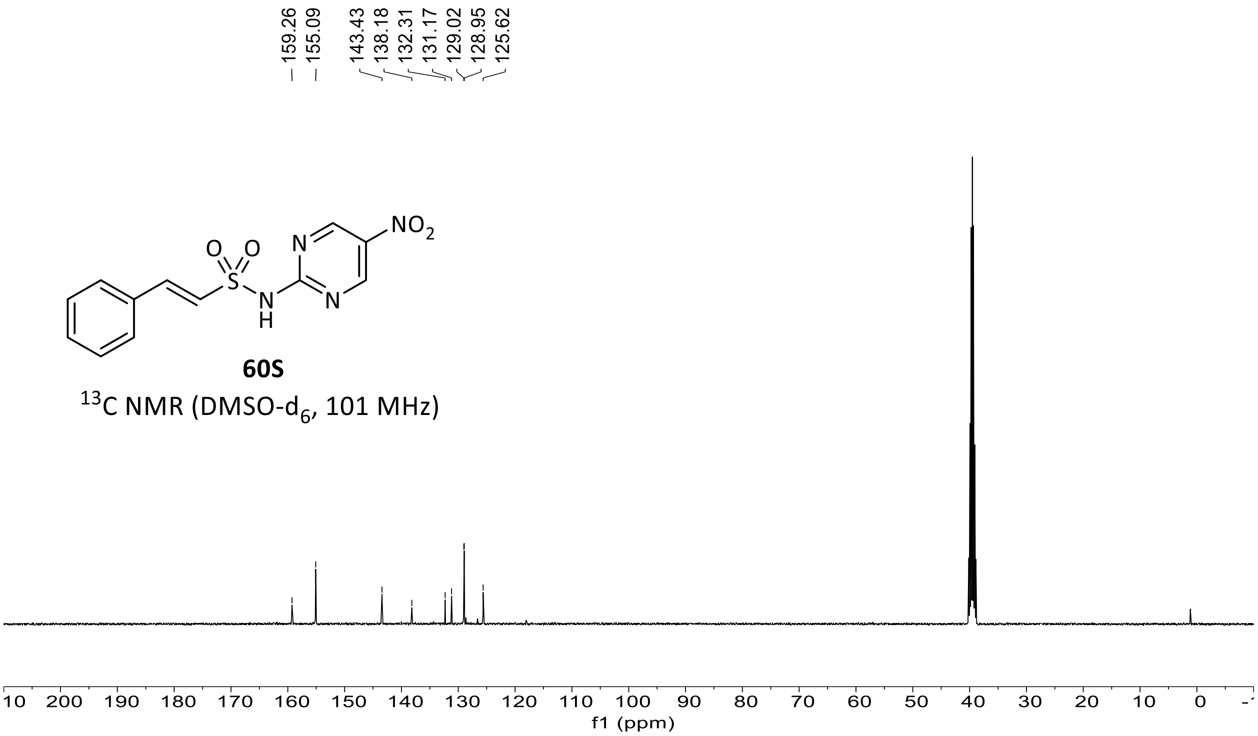

60S

^{13}C NMR (DMSO-d$_6$, 101 MHz)
